# Low Level of Antifungal Resistance in Iranian Isolates of Candida glabrata Recovered from Blood Samples in a Multicenter Study from 2015 to 2018 and Potential Prognostic Values of Genotyping and Sequencing of *PDR1*

**DOI:** 10.1128/AAC.02503-18

**Published:** 2019-06-24

**Authors:** Amir Arastehfar, Farnaz Daneshnia, Kamiar Zomorodian, Mohammad Javad Najafzadeh, Sadegh Khodavaisy, Hossein Zarrinfar, Ferry Hagen, Zahra Zare Shahrabadi, Michaela Lackner, Hossein Mirhendi, Mohammadreza Salehi, Maryam Roudbary, Weihua Pan, Markus Kostrzewa, Teun Boekhout

**Affiliations:** a Westerdijk Fungal Biodiversity Institute, Utrecht, Netherlands; b Basic Sciences in Infectious Diseases Research Center, Shiraz University of Medical Sciences, Shiraz, Iran; c Department of Medical Mycology and Parasitology, School of Medicine, Shiraz University of Medical Sciences, Shiraz, Iran; d Department of Parasitology and Mycology, Faculty of Medicine, Mashhad University of Medical Sciences, Mashhad, Iran; e Zoonoses Research Center, Kurdistan University of Medical Sciences, Sanandaj, Iran; f Department of Medical Parasitology and Mycology, Tehran University of Medical Sciences, Tehran, Iran; g Allergy Research Center, Mashhad University of Medical Sciences, Mashhad, Iran; h Division of Hygiene and Medical Microbiology, Medical University of Innsbruck, Innsbruck, Austria; i Department of Medical Parasitology and Mycology, School of Medicine, Isfahan University of Medical Sciences, Isfahan, Iran; j Department of Infectious Diseases and Tropical Medicine, Faculty of Medicine, Tehran University of Medical Sciences, Tehran, Iran; k Department of Medical Mycology and Parasitology, School of Medicine, Iran University of Medical Sciences, Tehran, Iran; l Department of Dermatology, Shanghai Key Laboratory of Molecular Medical Mycology, Shanghai Institute of Medical Mycology, Shanghai Changzheng Hospital, Second Military Medical University, Shanghai, China; m Bruker Daltonik GmbH, Bremen, Germany; n Institute of Biodiversity and Ecosystem Dynamics, University of Amsterdam, Amsterdam, Netherlands

**Keywords:** antifungal susceptibility testing, Candida glabrata, candidemia, Cg*PDR1*, *ERG11*, genotyping, HS1 of *FKS1* and *FKS2*, Iran

## Abstract

Establishing an effective empirical antifungal therapy requires that national surveillance studies be conducted. Herein, we report the clinical outcome of infections with and the microbiological features of Iranian isolates of Candida glabrata derived from patients suffering from candidemia. C. glabrata isolates were retrospectively collected from four major cities in Iran; identified by a 21-plex PCR, matrix-assisted laser desorption ionization–time of flight mass spectrometry, and large subunit of ribosomal DNA sequencing; and genotyped by amplified fragment length polymorphism (AFLP). Mutations in *PDR1*, *ERG11*, and hot spot 1 (HS1) of *FKS1* and *FKS2* were investigated, and antifungal susceptibility testing (AFST) was performed (by the CLSI M27-A3 and M27-S4 methods). Seventy isolates of C. glabrata were collected from 65 patients with a median age of 58 years. Fluconazole was the most widely used (29.23%) and least effective antifungal agent. The overall crude mortality rate was 35.4%. Only one strain was resistant to fluconazole, and 57.7% and 37.5% of the isolates were non-wild type (non-WT) for susceptibility to caspofungin and voriconazole, respectively. All isolates showed the WT phenotype for amphotericin B, posaconazole, and itraconazole. HS1 of *FKS1* and *FKS2* did not harbor any mutations, while numerous missense mutations were observed in *PDR1* and *ERG11*. AFLP clustered our isolates into nine genotypes; among them, genotypes 1 and 2 were significantly associated with a higher mortality rate (*P* = 0.034 and *P* = 0.022, α < 0.05). Moreover, 83.3% of patients infected with strains harboring a single new mutation in *PDR1*, T745A, died despite treatment with fluconazole or caspofungin. Overall, Iranian isolates of C. glabrata were susceptible to the major antifungal drugs. Application of genotyping techniques and sequencing of a specific gene (*PDR1*) might have prognostic implications.

## Abstract (precis)

Establishing an effective empirical antifungal therapy requires that national surveillance studies be conducted. Herein, we report the clinical outcome of infections with and the microbiological features of Iranian isolates of Candida glabrata derived from patients suffering from candidemia.

## INTRODUCTION

Candida glabrata is considered the second most common cause of candidemia in the United States and some European countries (1–4) and the third most common cause in Spain (5). Patients infected with C. glabrata require higher expenses for health care and longer stays in the hospital than those infected with C. albicans (6). The emergence of strains resistant to fluconazole (FLC) (7), echinocandins, and/or other antifungals (multidrug-resistant [MDR] strains) (8, 9), along with the limited number of antifungal drugs, has created a therapeutic challenge.

Although gain-of-function mutations in the transactivating transcription factor of C. glabrata *PDR1* (Cg*PDR1*) have been considered the main cause of azole resistance in C. glabrata (10), some mutations in *ERG11* are linked to MDR strains highly resistant to FLC, voriconazole (VRC), and amphotericin B (AMB) (11). Resistance to echinocandins is mainly mediated by mutations in hot spot 1 (HS1) of *FKS1* and *FKS2* (12), which are considered independent factors for the prediction of the therapeutic failure of echinocandins (13).

Although C. glabrata is recognized to be an asexual *Candida* species, genomic studies showed a high genetic variability of the clinical isolates of C. glabrata obtained from various countries (14). Moreover, it has been known that a higher mortality rate is attributable to some genotypes (15), and it might even be hypothesized that some genotypes are more virulent and resistant (15). Hence, utilization of genotyping techniques, such as multilocus sequence typing (MLST) (15), microsatellite typing (9), pulsed-field gel electrophoresis (16), amplified fragment length polymorphism (AFLP) analysis (17), and polymorphic locus sequence typing (18), is relevant for infection control. Although MLST has been extensively used for the genotyping of clinical isolates of C. glabrata, AFLP showed a higher resolution (19), and it is also a preferred typing method for C. auris (20) and Aspergillus terreus (21).

Determination of the antifungal susceptibility pattern on a national level is a prerequisite to understanding the evolving susceptibility profile of C. glabrata. A lack of systematic and nationwide microbiological and clinical data for Iranian isolates of C. glabrata recovered from blood samples prompted us to conduct the present study. Clinical isolates of C. glabrata were retrospectively collected from four major cities in Iran from 2015 to 2018. Antifungal susceptibility testing was performed according to the CLSI M27-A3 and M27-S4 methods (22, 23), characterization of genotypes was carried out by AFLP, and the presence of mutations in genes conferring resistance to azoles (*PDR1* and *ERG11*) and echinocandins (HS1 of *FKS1* and *FKS2*) was explored. Moreover, important clinical data were mined from the history of infected patients and are presented.

## RESULTS

### Clinical outcomes.

The clinical data used in this study are listed in Table S2 in the supplemental material (in the form of an Excel file). In total, 70 isolates of C. glabrata were recovered from 65 patients with a median age of 58 years. Among them, 47.7% (*n* = 31) were female and 52.3% (*n* = 34) were male. The majority of the isolates (86.1%; *n* = 56) were recovered from blood, followed by central venous catheters and abdominal fluids, each at 3.08% (*n* = 2 each), and abdominal wounds, dialysis fluid, cerebrospinal fluid (CSF), double lumen (DL), and triple lumen (TL), each at 1.54% (*n* = 1 each) (Table S2). Intensive care units, coronary care units, neonatal intensive care units, and pediatric intensive care units accommodated the majority of the patients (47.69%), followed by other hospital units, including surgery (18.46%), emergency (15.38%), internal medicine (12.31%), pediatric (3.08%), and infectious diseases (1.54%) units and general units for men (1.54%). Regarding underlying conditions, other infections and tumors were observed in 47.7% of patients, followed by conditions related to trauma and surgery (20.00%), metabolic disorders (9.23%), blood-associated disease (7.69%), autoimmune disease and liver and kidney dysfunctions (each at 4.62%), gastrointestinal bleeding (GIB; 3.08%); and poisoning (1.54%). The majority of patients were treated with fluconazole (29.23%), followed by caspofungin (CAS; 18.46%), AMB (10.77%), voriconazole (3.08%), and clotrimazole ointment (1.54%). Patients treated with caspofungin showed the highest rate of survival (83.3%), followed by those treated with AMB (71.43%) and fluconazole (52.63%). Twenty-four (36.92%) patients did not receive any treatment, and 9 of them (37.50%) died and 15 (62.50%) survived. The overall crude mortality rate among patients infected with C. glabrata was 35.4% (*n *=* *23).

### Screening for mutations in *PDR1*, *ERG11*, and HS1 of *FKS1* and *FKS2*.

Sequencing for mutations in *PDR1* showed that 54.92% (*n *=* *39) of the isolates contained nonsynonymous mutations (Table 1, Tables S3 and S4, and Fig. S3), 45.08% (*n *=* *39) of the isolates were wild type (WT), and 64.78% (*n *=* *39) harbored silent mutations (Table S4). Twenty-eight percent of the mutations were located in the region between the binding and middle homology domains and found in isolates that showed the highest MIC values for fluconazole (≥32 and 64 μg/ml). Regarding the association of the occurrence of mutations in *PDR1* and voriconazole MIC values, 45.1% of the isolates with wild-type *PDR1* and 30.7% of the isolates with non-WT *PDR1* (carrying various nonsynonymous mutations) had MIC values higher than the epidemiological cutoff value (ECV) (MIC ≥ 0.5 μg/ml) (Table 2). Among strains with nonsynonymous mutations in *PDR1*, K67N (MIC = 2 μg/ml), G128E + G493A (MIC = 0.5 μg/ml), K430M + T745A (MIC = 0.5 μg/ml), E555K (MIC = 4 μg/ml), and T745 + C930R (MIC = 0.5 μg/ml) exclusively occurred in strains with a voriconazole MIC greater than the ECV (Table 2). Regarding *ERG11*, 36.6% (*n *=* *26) of the isolates showed nonsynonymous mutations, 63.38% (*n *=* *26) were wild type, and 81.69% (*n *=* *58) harbored silent mutations (Table 3, Tables S3 and S4, and Fig. S3). Almost 22.53% (*n *=* *16) of the isolates simultaneously contained mutations in both the *PDR1* and *ERG11* genes (Table S4). The hot spot 1 regions of both *FKS1* and *FKS2* were devoid of any mutations. Isolates simultaneously harboring mutations in both *PDR1* and *ERG11* and those harboring a mutation in either gene did not show significantly higher MIC values than the wild types. Surprisingly, five out of six patients infected with strains containing a single mutation of T745A in *PDR1* died, despite treatment with fluconazole, caspofungin, or a combination of both drugs. These strains were found in two cities, Mashhad (*n *=* *5) and Shiraz (*n *=* *1), and as determined using AFLP, they were clustered into five distinguishing genotypes (two strains from Mashhad shared the same genotype).

**TABLE 1:** Frequency of resistance to fluconazole in wild-type and mutated strains for *PDR1*

Polymorphism(s) in *PDR1*	No. of isolates with the following MIC (μg/ml):										
	≤0.5	1	2	4	8	16	32	64	128	≥256	Total
WT				5	15	10	1				32
K67N					1						1
P68S, P135T, D235N						1	1				2
P76S, P145T, D243N					3	1	1	1^*a*^			6
P117S				1							1
G128E						1					1
G128E, G493A					1						1
N162S						1					1
N162S, F944S					1						1
G189V					1						1
Y285N, T286A, K430M, T745A					1						1
K430M				2							2
K430M, E441K						1					1
K430M, L454P						1					1
K430M, T745A						1					1
K430M, G493A, T745A						1					1
E555K						1					1
G574S					1						1
T745A			1		3	2					6
T745A, C930R						1					1
A828T							1				
C930R				2		3	1				6
A1004C					1						1

a Only one of the isolates with this mutation (P76S, P145T, D243N) was resistant to fluconazole, and the rest of the isolates were 100% SDD to this drug.

**TABLE 2:** Frequency of isolates with wild-type and mutated *PDR1* profile along with their MIC values for voriconazole

Polymorphism(s) in *PDR1*	% of isolates with MIC:		No. of isolates along with the following MIC (μg/ml):										
	Less than ECV	Greater than ECV	≤0.0625	0.125	0.25	0.5	1	2	4	8	16	≥32	Total
WT	54.9	45.1	1	7	9	6	4	3			1		31
K67N	0.00	100						1					1
P68S, P135T, D235N	100	0.00		2									2
P76S, P145T, D243N	67.67	33.33		2	2	2							6
P117S	100	0.00		1									1
G128E	100	0.00			1								1
G128E, G493A	0.00	100				1							1
N162S	100	0.00			1								1
N162S, F944S	100	0.00		1									1
G189V	100	0.00			1								1
Y285N, T286A, K430M, T745A	100	0.00			1								1
K430M	100	0.00		1	1								2
K430M, E441K	100	0.00			1								1
K430M, L454P	100	0.00		1									1
K430M, T745A	0.00	100				1							1
K430M, G493A, T745A	100	0.00		1									1
E555K	0.00	100							1				1
G574S	100	0.00			1								1
T745A	50	50	1	1	1	2	1						6
T745A, C930R	0.00	100				1							1
A828T	0.00	100				1							1
C930R	67.67	33.33		2	2	1	1						6
A1004C	100	0.00			1								1

**TABLE 3:** Frequency of resistance to fluconazole in wild-type and mutated strains for *ERG11*

Polymorphism(s) in *ERG11*	No. of isolates along with the following MIC (μg/ml):										
	≤0.5	1	2	4	8	16	32	64	128	≥256	Total
WT			1	7	18	13	5	1^*a*^			45
D196N					1						
N368T				2	3	7					12
N368T, H430P					1	1					2
N368T, K456R, G457C, V458F						1					1
N425I				1							1
H430P				1	4	2					7
K456R, G457C, V458F					1						1

a Only one of the *ERG11* wild-type isolates was fluconazole resistant, and the rest of the wild-type isolates and the isolates with *ERG11* mutations were 100% SDD to this drug.

### Genotyping of isolates using AFLP.

**FIG 1:**
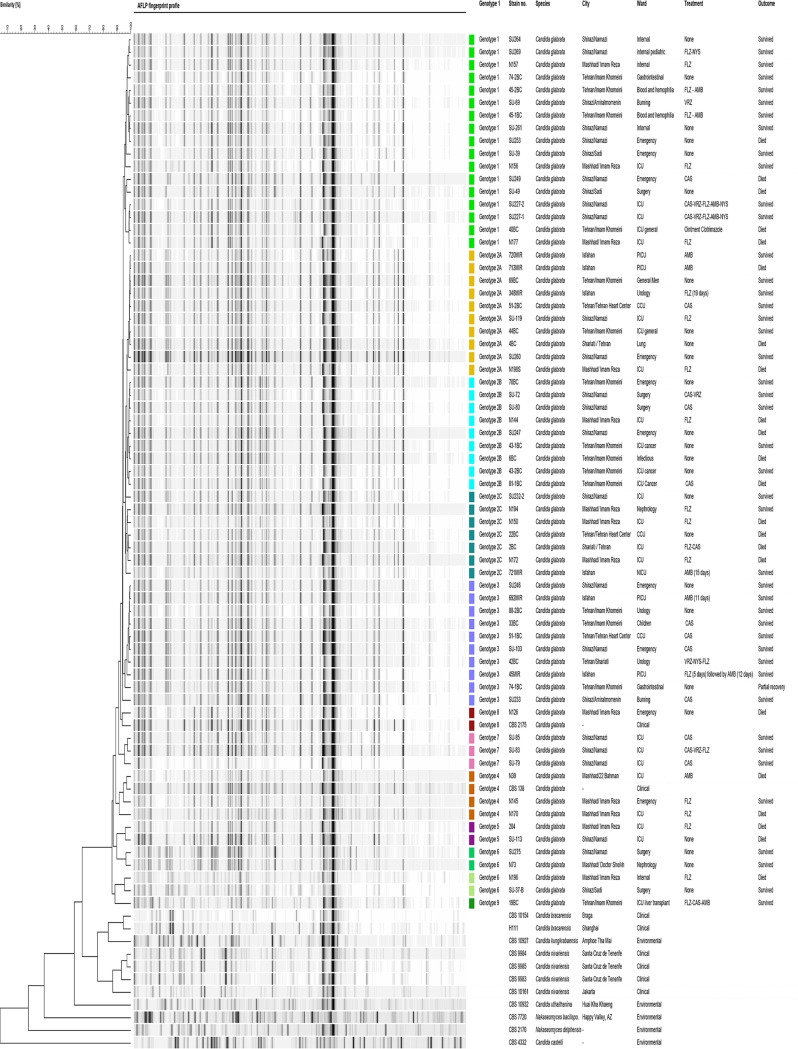
AFLP genotyping for the studied strains of C. glabrata. Our isolates were clustered into nine genotypes using AFLP, and each genotype is distinctively color coded. ICU, intensive care unit; CCU, coronary care unit; NICU, neonatal intensive care unit; PICU, pediatric intensive care unit; FLZ, fluconazole; NYS, nystatin; AMB, amphotericin B; VRZ, voriconazole; CAS, caspofungin.

AFLP divided our isolates into 9 distinct clusters (genotype 1 [G1] to G9), and genotype 2 was comprised of three subgenotypes, G2A, G2B, and G2C (Fig. 1). Two isolates, collected from Tehran and Isfahan, showed a bizarre banding pattern compared to the rest of the C. glabrata isolates, and they clustered with C. nivariensis and C. uthaithanina. Subsequently, the respective DNA samples were subjected to the 21-plex PCR, and two bands representing C. glabrata and C. parapsilosis were revealed, indicating that the DNA samples contained a mixture of the DNA of both of these species. As a result, the DNA samples obtained from these two isolates were excluded from downstream genotyping analysis. There was no significant difference between the resistance profile and the genotype clusters (Table 4). The associations of various genotypes with the profile of resistance to fluconazole are summarized in Table 4. Although by the chi-square test (two-tailed) the clinical outcome was significantly associated only with G3 (*P *=* *0.025), logistic regression and path analysis showed that G1 (*P *=* *0.034) and G2 (*P *=* *0.022) were significantly associated with a higher rate of mortality (α* *<* *0.05), while G3 was significantly associated with survival (*P *=* *0.001, α* *<* *0.05) (see the “Statistical analysis” section in the supplemental material). Moreover, by the chi-square test (two-tailed) there was no significant association between the clinical outcome and the VRZ resistance profile (*P *=* *0.555). Additionally, multivariate logistic regression analysis did not show a significant association between the clinical outcome and the hospitalization duration (*P *=* *0.291) (see the “Statistical analysis” section in the supplemental material).

**TABLE 4:** MIC distribution of fluconazole among C. glabrata isolates of different genotypes

Genotype	No. of isolates along with the following MIC (μg/ml):										
	≤0.5	1	2	4	8	16	32	64	128	≥256	Total
G1				2	8	4	2	1^*a*^			17
G2 (B and C)				4	9	12	1				26
G3				2	4	3	1				10
G4				1		2					3
G5					1	1					2
G6			1		3						4
G7				1	1	1					3
G8					1						1
G9					1						1

a Only one of the isolates within genotype 1 (G1) was resistant to fluconazole, and the rest of the isolates were SDD to this drug.

### Antifungal susceptibility pattern.

All of the MIC values obtained in this study are summarized in Table 5 and Table S3. Resistance to fluconazole (MIC ≥ 64 μg/ml) was noted in only one isolate (1.4%), and the rest were susceptible dose dependent (SDD), while 36.43% (*n *=* *28) of the isolates showed MIC values higher than the ECV for voriconazole (MIC ≥ 0.5 μg/ml) and all of the isolates showed the WT phenotype for posaconazole (PSC; MIC ≥ 2 μg/ml) and itraconazole (ITC; MIC ≥ 4 μg/ml). No cross-resistance between azole drugs was observed. For caspofungin, 57.74% of the isolates (*n *=* *41) showed MICs above the ECV (≥0.5 μg/ml), while for AMB, none of the isolates showed an MIC greater than the ECV (AMB ECV > 2 μg/ml) (24). Although resistance to echinocandins is noted when resistance to at least two antifungal agents in this class is observed (12, 25), caspofungin was the only echinocandin agent that was available in our study. Moreover, due to the interlaboratory variation observed for caspofungin (26), the MIC values of caspofungin were combined with the sequence data for HS1 of *FKS1* and *FKS2* as a surrogate for the caspofungin MIC. Almost 24% (*n *=* *17) of the isolates simultaneously had MIC values higher than the ECVs for both caspofungin and voriconazole (MIC ≥ 0.5 μg/ml), and among these isolates, 35.29% (*n *=* *6) had MIC values of ≥1 μg/ml and ≥0.5 μg/ml for voriconazole and caspofungin, respectively. Fluconazole showed the highest geometric mean value (10.31), followed by amphotericin B (0.57), itraconazole (0.51), caspofungin (0.41), posaconazole (0.41), and voriconazole (0.32).

**TABLE 5:** Antifungal susceptibility data derived from C. glabrata isolates in this study

Antifungal drug	No. of isolates along with the following MIC (μg/ml):													MIC range (μg/ml)	GM^*a*^ mean MIC (μg/ml)
	≤0.016	0.032	0.064	0.125	0.25	0.5	1	2	4	8	16	32	≥64		
FLC								1	11	28	24	5	1	2–64	10.11
VRC			2	20	21	16	6	4	1		1			0.064–16	0.32
PSC		1	1	1	15	27	26							0.032–1	0.41
ITC			2	3	21	34	10	1						0.064–2	0.51
CAS				8	22	22	19							0.125–1	0.41
AMB					3	52	15	1						0.25–2	0.57

a GM, geometric mean.

## DISCUSSION

The steady increase in the incidence of candidemia due to C. glabrata, along with a concerning development of resistance to azoles and echinocandins and even the emergence of strains with MDR traits, highlights the importance of studying antifungal susceptibility, determining the subcellular mechanisms of resistance involved, and genotyping clinical isolates of C. glabrata (9, 12). Previously, studies conducted in China (9, 27), South Korea (15), India (28), and the United States (12) investigated the aforementioned aspects of clinical isolates of C. glabrata and showed variability in the rate of resistance to azoles and echinocandins in those countries. As this information for Iranian isolates of C. glabrata is lacking on a nationwide scale, we conducted a multicenter study to investigate the clinical and microbiological features of this species.

In our study, no difference in the occurrence of candidemia due to C. glabrata was observed between males and females. Consistent with the findings of other studies, infections due to C. glabrata were mainly observed in elderly individuals (6, 29), with the median age being 58 years. Moreover, the underlying conditions observed for our patients, namely, the extensive use of broad-spectrum antibiotics, cancer, other infections, and surgery, are recognized risk factors for the development of candidemia (6, 29). Although clinical guidelines consider echinocandins to be the frontline therapy for C. glabrata (30), in our study, caspofungin ranked as the second treatment option, and patients treated with caspofungin showed a higher rate of survival than those treated with fluconazole. The lower rate of utilization of echinocandins than azoles in developing countries might reflect the higher costs associated with these drugs (28). Unlike other studies, in which the mortality rate was reported to be 58% to 61% (31), in our study, approximately 35% of our patients died, similar to the rate reported from the United States (6).

As no mutations were observed in HS1 of *FKS1* and *FKS2*, none of our isolates were categorized as echinocandin resistant. Due to the unreliability of the MIC values of caspofungin (26) and the superiority of the presence of mutations in HS1 of *FKS1* and *FKS2* for the detection of resistance to echinocandins (32), resistance to echinocandins was inferred only based on the presence of a mutation in HS1 of the aforementioned genes. This is in line with our findings, where the vast majority of the isolates (57.74%) had an MIC greater than the ECV (0.5 μg/ml), while there were no mutations in HS1 of *FKS1* and *FKS2*. Contrary to the echinocandin resistance rate in the United States, which is up to 13% (12), the lack of echinocandin resistance in our study is similar to that in other Asian countries, including South Korea (0%), India (0%), China (1.9%), and Turkey (2%) (15, 27, 28, 33), and European and South American countries (31, 34–37). This variation in the rate of resistance to echinocandins likely reflects the variation in the therapeutic regimens implemented in a specific region/country (28) and the genetic differences between isolates of C. glabrata (15).

A low level of resistance to fluconazole was observed (one isolate, 1.4%), and the rest of the isolates were categorized to have the SDD phenotype. This rate of resistance to fluconazole is similar to what is observed in the other Asian and South American countries, where the incidence of fluconazole resistance varies from 0% to 8.9% (15, 27, 28, 33, 34). As strains harboring mutations in *PDR1* or *ERG11* did not exhibit higher MIC values than wild-type strains (Tables 3 and 5), it could be inferred that those mutations are not engaged in resistance. The fluconazole-resistant isolate carried previously described mutations (P76S, P145T, D243N) (27) that were also found in isolates with the SDD phenotype (Table 3). Although in some other *Candida* species, such as C. albicans (38), fluconazole and voriconazole resistance are governed by the same mechanism, none of our strains showed concurrent cross-resistance/a non-WT phenotype for FLZ and VRC. Moreover, the majority of strains with nonsynonymous mutations occurring in *PDR1* (*n* =* *26; 66.6%) had a VRZ MIC less than the ECV, and among those with an MIC greater than the ECV, only one-third were exclusively found among VRZ non-WT strains (strains with the K67N, G128E + G493A, K430M + T745A, E555K, and T745 + C930R mutations). Besides, *PDR1* WT strains had a higher proportion with a non-WT phenotype for VRZ than non-WT strains (45.1% for WT strains versus 30.7% for non-WT strains) (Table 2). Collectively, these observations point to the fact that in C. glabrata, resistance to fluconazole and voriconazole might not be controlled by the same mechanism. For *ERG11*, all nonsynonymous mutations occurred in fluconazole SDD strains. X-ray crystallography studies of *ERG11* of Saccharomyces cerevisiae (39) and homology modeling in C. glabrata (40) showed that missense mutations in residues 132, 140, 143, and 464 and residues 146, 243, and 246, respectively, are linked to azole resistance. On the contrary, in our study none of the isolates with substitutions in the neighborhood of those residues (residues 196, 425, 430, and 456 to 458) showed resistance to fluconazole. Moreover, unlike S. cerevisiae (39), the occurrence of a mutation in residue 315 (G315D) of a clinical strain of C. glabrata caused multidrug resistance to fluconazole (MIC > 256 μg/ml), voriconazole (MIC > 256 μg/ml), and AMB (MIC > 32 μg/ml) (11). None of the isolates showed MIC values higher than the ECV (MIC > 2 μg/ml) for AMB. The low level or a lack of resistance to azoles and AMB or echinocandins in this study might be explained by the fact that none of our patients experienced previous and prolonged exposure to these antifungals (41, 42).

Although mutations in the *MSH2* (DNA mismatch repair pathway) gene correspond to hypermutable phenotypes of C. glabrata that can facilitate the development of azole-resistant and MDR strains (8), studies from India (28), France (43), and China (27) found that mutations in this gene are more associated with rare and specific genotypes. Therefore, we did not include this gene in our study.

The observation of hypervariation in the virulence patterns for each strain of C. glabrata (44), along with the association of certain genotypes with a higher rate of mortality (15), revealed the importance of the use of genotyping techniques in clinical settings. In line with these findings, in our study, two genotypes, G1 and G2, showed a significant association with a higher rate of mortality (α* *<* *0.05, *P *=* *0.034 and *P *=* *0.022, respectively), while G3 was significantly associated with survival (α* *<* *0.05, *P *=* *0.001). Additionally, it has been shown that mutations in *PDR1* have implications for virulence, and strains carrying certain mutations showed reduced adherence to macrophages and increased adhesion to epithelial cells (10). Interestingly, we noticed that five out of six patients infected with strains carrying the single T745A mutation in *PDR1* (not in combination with the other mutations in *PDR1*) died despite treatment with either fluconazole, caspofungin, or a combination of both drugs. Five of those isolates belonging to four genotypes (two strains shared the same genotype) were found in the same city (Mashhad) and the same hospital in which 80% of infected patients died (*n *=* *4). The other isolate belonging to a different genotype was found in Shiraz, and the infected patient died. This conclusion is drawn based on the findings for a small number of strains, and so it is not conclusive, due to the pleiotropic functions of *PDR1*, and this specific mutation (T745A) might deserve further *in vivo* studies. Surprisingly, in our study isolates of each genotype of C. glabrata were recovered from patients hospitalized in different cities. Admittedly, AFLP might not have the genotyping resolution of whole-genome sequencing platforms, but this observation might be indicative of the nosocomial transmission of C. glabrata isolates. Although it is rarely reported, some studies have shown the nosocomial transmission of C. glabrata isolates in clinical settings (18, 45).

## MATERIALS AND METHODS

### Collection of isolates and ethical approval.

Isolates of C. glabrata were retrospectively collected from the Iranian cities of Tehran, Isfahan, Shiraz, and Mashhad from 2015 to 2018 (see Fig. S1 in the supplemental material). The procedure of study in each center was evaluated by regional ethical committee members, and accordingly, they were provided with ethical codes (IR.SUMS.REC.1397.365, IR.MUMS.fm.REC.1397.268, and IR.TUMS.SPH.REC.1396.4195). Prior to studying the isolates and analyzing the clinical data, each patient and the isolates derived from them were designated with specific codes to prevent exposing the patients’ personal data.

### Identification.

Isolates were preliminarily identified by a 21-plex PCR (46). Isolates were serially coded from 1 to 70. They were reidentified by matrix-assisted laser desorption ionization–time of flight mass spectrometry (MALDI Biotyper; Bruker Daltonik GmbH, Bremen, Germany) (47) and sequencing of domain 1 (D1) and D2 of the large subunit of ribosomal DNA (LSU rDNA) (48).

### DNA extraction.

DNA samples were extracted by the cetyltrimethylammonium bromide (CTAB) method (100 mM Tris-HCl, pH 8,4, 1.4 M NaCl, 25 mM EDTA, pH 8.0, 2% CTAB) (49). The quality of the DNA samples was assessed by use of a NanoDrop spectrophotometer (Thermo Fisher Scientific Corporation, Waltham, MA, USA) and the running of 5 μl of DNA sample on a 0.7% agarose gel, and the quality and quantity were evaluated by use of a QuBit double-stranded DNA BR assay kit (Thermo Fisher Scientific Corporation, Waltham, MA, USA).

### Primer design, PCR, and sequencing for *FKS1*, *FKS2*, *PDR1*, and *ERG11*.

The DNA sequences of HS1 of *FKS1* and *FKS2*, *PDR1*, and *ERG11* were determined and screened for the presence of mutations. Fourteen primers comprising 2 external primers and 12 internal primers were used to sequence *PDR1*, and 8 primers, including 2 external primers and 6 internal primers, were used to sequence *ERG11* (Table S1 and Fig. S2). Primers were synthesized by Integrated DNA Technology (Leuven, Belgium).

PCR mixtures for *FKS1*, *FKS2*, *PDR1*, and *ERG11* were prepared in a volume of 50 μl and were as follows: 5 μl 10× buffer (10× NH_4_, no MgCl_2_), 2 mM MgCl_2_, 0.2 mM deoxynucleoside triphosphate (dNTP) mix (dNTP mix, 100 mM; Biolab), 5 pmol of primers (primers FKS1-F, FKS1R, FKS2F, FKS2R, PDR1Fex, PDR1Rex, ERG11Fex, and ERG11Rex), and 2.5 units of *Taq* polymerase enzyme (Bio *Taq* DNA polymerase; Biolab). Milli-Q water was used to adjust the volume to 50 μl.

All PCRs were set at the same annealing temperature, but variable incubation times were used for the extension phase. PCR programs contained the following steps: 95°C for 5 min, followed by 95°C for 30 s, 58°C for 30 s, and 72°C for 30 s (*FKS1*), 1 min (*FKS2*), 2 min (*ERG11*), or 3 min (*PDR1*), followed by 72°C for 8 min. The PCR products were run on a 2% agarose gel.

### Sequencing and analysis of sequences.

The primers presented in Table S1 were used for bidirectional dideoxy chain-terminated Sanger sequencing. Contigs were assembled and edited by the use of SeqMan software (DNAStar, Madison, WI, USA), and the sequences obtained were aligned by the use of MEGA software (v.7.0; Temple University, Philadelphia, PA). The sequences with GenBank accession numbers FJ550269.1 (10) and XM_445876 (50) were used as the WT references for the *PDR1* and *ERG11* sequences, respectively.

### Genotyping using AFLP.

The amplified fragment length polymorphism (AFLP) method suggested by Marchetta et al. (51) was employed to evaluate the genotypic patterns of our isolates of C. glabrata. AFLP data were analyzed by the use of BioNumerics software (v7.6; Applied Math Inc., Austin, TX, USA). The reference and type strains of C. glabrata (strains CBS 138 and CBS 2175, respectively) and the other closely related species, including C. nivariensis (CBS 9983 to CBS 9985 and CBS 10161), C. bracarensis (CBS 10154), C. uthaithanina (CBS 10932), C. kungkrabaensis (CBS 10927), Nakaseomyces delphensis (CBS 2170), and Nakaseomyces bacillisporus (CBS 7720) and a clinical isolate of C. bracarensis (generously provided by W. Liao, Shanghai, China), were included in the AFLP experiment.

### Antifungal susceptibility testing.

The MIC values of the antifungal drugs were determined by a broth microdilution procedure as described in CLSI document M27-A3 (22). The following antifungal drugs were included: fluconazole (Pfizer, New York, NY, USA), voriconazole (Pfizer, New York, NY, USA), itraconazole (Santa Cruz Biotech, Dallas, TX, USA), posaconazole (MSD, Kenilworth, NJ, USA), caspofungin (Merck & Co., Inc.), and amphotericin B (Sigma Chemical Corporation, St. Louis, MO). For quality control purposes, C. parapsilosis (CBS 604) and C. krusei (CBS 5147) were used. Species-specific breakpoints were adopted from CLSI document M27-S4 (23). The MIC was read visually after 24 h and noted as the lowest concentration of fluconazole (FLZ) and caspofungin (CAS) resulting in at least a 50% reduction of growth compared to that of the control. Resistance to FLZ and CAS was noted when the MIC values were ≥64 μg/ml and ≥0.5 μg/ml, respectively. The MIC values of other azole drugs, including voriconazole (VRC) (≥ 1 μg/ml), posaconazole (PSC) (≥4 μg/ml), and itraconazole (ITC) (≥4 μg/ml), were interpreted according to epidemiological cutoff values (23, 52). The MIC values of AMB were noted to be the lowest concentration of the drug that showed a 100% reduction of growth of the test strain compared to that of a control strain grown without AMB, and isolates for which MIC values were >2.0 μg/ml were considered to be potentially resistant (23, 24, 52).

### Statistical analysis.

Logistic regression and path analyses were performed to evaluate statistical significance and the association between the genotypes and death or survival. As multivariate logistic regression analysis does not consider the indirect influence of independent variables on dependent ones, path analysis was used to overcome this problem. Using path analysis, the association with mortality and survival was individually assessed for genotypes 1 to 3. Moreover, the chi-square test (two-tailed) was used to find the association between the clinical outcome and genotypes, voriconazole susceptibility profile (susceptible or resistance), and hospitalization duration for all patients. *P* values of <0.05 were considered statistically significant. All statistical analyses were performed with SPSS software (v.24 for Windows; SPSS, Inc., Chicago, IL, USA) (see the “Statistical analysis” section in the supplemental material).

### Data availability.

All the isolates of C. glabrata studied in this project were deposited in the culture collection the Westerdijk Fungal Biodiversity Institute, and they were designated with the following CBS numbers: CBS 15665 to CBS 15720, CBS 15722 to CBS 15733, and CBS 15744. Sequences obtained for *PDR1*, *ERG11*, and HS1 of *FKS1* and *FKS2* were deposited in GenBank under the following accession numbers: MK847567 to MK847637, MK847780 to MK847850, MK847638 to MK847708, and MK847709 to MK847779, respectively.

## Supplementary Material

Supplemental file 1

Supplemental file 2
