# Supplementary material for: Low Level of Antifungal Resistance in Iranian Isolates of Candida glabrata Recovered from Blood Samples in a Multicenter Study from 2015 to 2018 and Potential Prognostic Values of Genotyping and Sequencing of PDR1
Source: Antimicrob Agents Chemother. 2019 Jun 24;63(7):e02503-18. doi: 10.1128/AAC.02503-18 (PMC6591624; doi:10.1128/AAC.02503-18)
Supplement: Supplemental file 1 [file AAC.02503-18-s0001.pdf]

Supplementary Table 1. List of primers used for PCR and sequencing of target genes conferring resistance to azoles and echinocandins

**Oligo Name**	**Sequence**	**Target gene/Purpose**	**PCR product sizes**	**Reference**
Cg-PDR1-Fexternal	AACAAGCATAGAGGCGCTGT	Cg*PDR1*/PCR and sequencing	3616bps	(1)
Cg-PDR1-R1	AGACCTCTATGGCATTGA	Cg*PDR1*/sequencing	---	This study
Cg-PDR1-F1	GCTAATATTCATGTTGGTC	Cg*PDR1*/sequencing	---	This study
Cg-PDR1-R2	TCTGCAAGACTAAATCCAC	Cg*PDR1*/sequencing	---	This study
Cg-PDR1-F2	AGATTAATGAAAGTGCCACC	Cg*PDR1*/sequencing	---	This study
Cg-PDR1-R3	TTCGCCTTATAGACTTACA	Cg*PDR1*/sequencing	---	This study
Cg-PDR1-F3	GATAATAGAGAGAATACCG	Cg*PDR1*/sequencing	---	This study
Cg-PDR1-R4	CACCCTATCCAGATAGCCA	Cg*PDR1*/sequencing	---	This study
Cg-PDR1-F4	AAGAGGCGGCTACTATGG	Cg*PDR1*/sequencing	---	This study
Cg-PDR1-R5	TTGAATAATCGTTGTCCAT	Cg*PDR1*/sequencing	---	This study
Cg-PDR1-F5	GGTGAAACCAAAATCAGATT	Cg*PDR1*/sequencing	---	This study
Cg-PDR1-R6	GCTAAATTCACCATTGCAC	Cg*PDR1*/sequencing	---	This study
Cg-PDR1-F6	CCTCCGATGCTTCTAGATC	Cg*PDR1*/sequencing	---	This study
Cg-PDR1-Rexternal	TGAGGTAGTCTAAGTCTCATG	Cg*PDR1*/PCR and sequencing	3616bps	(1)
Fex-ERG11	ATATTCCACCTCGAAGAACCC	Cg*ERG11*/PCR and sequencing	2043bps	This study
R1-ERG11	TTCCATTAGTCTGTGGTT	Cg*ERG11*/ sequencing	---	This study
F1-ERG11	TTATGACTGTCTACTTGG	Cg*ERG11*/ sequencing	---	This study
R2-ERG11	TCATCTTAGTACCATCCTT	Cg*ERG11*/ sequencing	---	This study
F2-ERG11	ACAGAAAGAGAGATCATG	Cg*ERG11*/ sequencing	---	This study
R3-ERG11	CGATACATCTGTGTCTAC	Cg*ERG11*/ sequencing	---	This study
F3-ERG11	TGATGCTGCTTCTTCCAG	Cg*ERG11*/ sequencing	---	This study
Rex-ERG11	TGTCATATGCTTGCACTGC	Cg*ERG11*/PCR and sequencing	2043bps	This study
FKS2-HS1-F	Not published data	Cg*FKS2*/PCR and sequencing	718bps	Not published data
FKS2-HS1-R	Not published data	Cg*FKS2*/PCR and sequencing	718bps	Not published data
FKS1-HS1-F	Not published data	Cg*FKS1*/PCR and sequencing	389bps	Not published data
FKS1-HS1-R	Not published data	Cg*FKS1*/PCR and sequencing	389bps	Not published data

Supplementary table 3. MIC values of tested antifungal drugs for each isolates along with mutations found in ERG11, PDR1, and HS1 of FKS1 and FKS2. The last two columns show the antifungal drugs that patients were treated with and the resultant outcomes.

	MIC Values (µg/ml)						ERG11 amino acid (nucleic acid)	PDR1amino acid (nucleic acid)	FKS1 HS1	FKS2 HS2	Treatment option	Outcome
	FLC	VRC	ITC	PSC	CASP	AMB						
**SU-264**	8.0	1.0	1.0	1.0	0.25	0.5	---	---	---	---	No Information	Survived
**SU-269**	16.0	0.5	0.5	0.5	0.5	0.5	---	---	---	---	FLC+NST	Survived
**N157**	8.0	0.125	0.5	0.25	0.25	0.5	---	---	---	---	Not Treated	Survived
**74-2BC**	16.0	0.25	1.0	1.0	0.25	0.5	---	---	---	---	No	Partial recovery
**45-2BC**	16.0	0.125	1.0	0.5	0.5	0.5	---	C930R (T2788Y)	---	---	FLC+AMB	Survived
**SU-69**	4.0	0.25	0.25	0.25	0.125	1.0	---	K430M (A1289W)	---	---	VRC	Survived
**45-1BC**	32.0	0.25	1.0	0.5	1.0	0.5	---	C930R (T2788Y)	---	---	FLC+AMB	Survived
**Su 261**	32.0	0.5	0.25	0.5	0.5	1.0	---	A828T (G2482R)			No Information	Survived
**SU-253**	4.0	0.25	0.25	1.0	0.25	0.5	---	---	---	---	Not Treated	Died
**SU-39**	8.0	0.125	0.25	0.25	0.5	0.5	H430P (A1289M	G189V (G566T)	---	---	Not treated	Survived
**N156**	8.0	0.062	0.062	0.031	0.25	0.25	---	---	---	---	FLC	Survived
**SU-249**	8.0	0.125	0.5	0.5	1.0	0.5	---	T745A (A2234G)	---	---	CASP	Died
**SU-49**	8.0	0.5	0.25	0.25	0.25	0.5	---	---	---	---	Not treated	Died
**SU-227(1)**	64.0	0.125	1.0	1.0	0.25	0.5	---	P76S(C226T)<br>P143T(C426A)<br>D243N (G727A)	---	---	CASP+VRC+AMB+NST	Survived
**SU-227(2)**	8.0	0.25	0.5	0.5	1.0	0.5	---	P76S(C226T)<br>P143T(C426A)<br>D243N (G727A)	---	---	CASP+VRC+AMB+NST	Survived
**40BC**	16.0	0.125	0.5	0.5	1.0	0.5	---	P68S (C202T)<br>P135T (C403A)<br>D235N (G703A)	---	---	Ointment Clotrimazole	Died
**N177**	8.0	0.125	1.0	0.5	1.0	0.5	---	P76S(C226T)<br>P143T(C426A)<br>D243N (G727A)	---	---	FLC	Died
**720MIR**	8.0	0.25	0.5	0.5	0.25	0.5	N368T (C1194T)<br>H430P (A1289M)	---	---	---		Survived
**713MIR**	32.0	16.0	1.0	1.0	0.25	1.0	---	---	---	---	AMB	Died
**69BC**	4.0	0.25	0.25	1.0	0.25	0.5	N368T (C1194T)	C930R (T2788Y)	---	---	No	Partial recovery
**348MIR**	16.0	2.0	0.5	1.0	1.0	1.0	N368T (C1194T)	---	---	---	FLC	Survived
**51-2BC**	16.0	0.25	0.5	0.5	0.25	0.5	---	N162S (A485G)	---	---	CASP	Survived
**SU-119**	4.0	0.125	0.25	0.125	0.125	1.0	N368T (C1194T)	K430M (A1289W)	---	---	FLC	Survived
**44BC**	16.0	0.125	0.5	0.5	1.0	0.5	N368T (C1194T)	C930R (T2788Y)	---	---	Not Treated	Survived
**4BC**	8.0	0.5	0.5	1.0	0.5	0.5	---	---	---	---	NO	Died
**SU-260**	16.0	0.5	0.5	0.5	0.5	0.5	N368T (C1194T)	---	---	---	No Information	Survived
**N198s**	8.0	0.5	0.5	0.5	0.5	0.5	N368T (C1194T)	T745A (A2234G)	---	---	FLC	Died
**70BC**	16.0	0.25	0.5	1.0	0.5	1.0	N368T (C1194T)<br>H430P (A1289M)	---	---	---	No	Survived
**SU-72**	8.0	0.5	0.5	0.5	1.0	1.0	H430P (A1289M)	G128E (G383R), G493A (G1478S)	---	---	CASP andVRC	Survived

**SU-80**	16.0	0.25	0.25	0.5	0.5	1.0	H430P (A1289M)	G128E (G383R)	---	---	CASP	Survived
**N144**	4.0	0.25	0.25	1.0	0.5	0.5	---	---	---	---	FLC	Died
**SU-247**	8.0	0.5	0.5	0.25	1.0	0.5	---	---	---	---	Not treated	Died
**43-1BC**	16.0	2.0	0.5	0.5	1.0	0.5	---	---	---	---	Not Treated	Survived
**43-2BC**	16.0	2.0	0.25	1.0	0.25	0.5	---	---	---	---	Not Treated	Survived
**6BC**	16.0	0.125	0.25	0.25	0.125	0.25	N368T (C1194T)<br>K456R (A1367R)<br>G457C (G1369K)<br>V458F (G1372K)	---	---	---	NO	Died
**81-1BC**	8.0	0.125	0.5	0.25	0.25	0.5	N368T (C1194T)	---	---	---	CASP	Died
**SU-232(2)**	8.0	0.25	0.5	0.5	1.0	0.5	D196N (G586A)	A1004S (G3011T)	---	---	Not Information	Survived
**N194**	8.0	0.5	0.25	1.0	0.5	0.5	N368T (C1194T)	T745A (A2234G)	---	---	FLC	Survived
**N150**	4.0	0.5	0.125	0.062	0.25	0.5	N425I (A1274T)<br>(heterozygous W)	---	---	---	FLC	Died
**2BC**	16.0	0.5	0.5	1.0	0.5	0.5	N368T (C1194T)	T745A (A2234G)<br>C930R (T2789Y)	---	---	FLC+CASP	Died
**N172**	16.0	1.0	1.0	0.5	0.5	0.5	N368T (C1194T)	T745A<br>(A2234G)	---	---	FLC	Died
**721MIR**	16.0	1.0	0.5	0.5	0.5	0.5	N368T (C1194T)	---	---	---	AMB	survived
**22BC**	8.0	0.125	0.5	0.25	1.0	0.5	K456R (A1367R)<br>G457C (G1369K)<br>V458F (G1372K)	N162S (A485G)<br>F944S (T2831Y)	---	---	Not Treated	Died
**SU-246**	8.0	0.25	0.5	1.0	0.5	1.0	---	---	---	---	Not Information	Survived
**692MIR**	8.0	2.0	0.25	1.0	0.25	1.0	---	K67N (A201C)	---	---	AMB	Survived
**88-2BC**	16.0	1.0	0.062	0.5	1.0	0.5	---	C930R (T2788Y)	---	---	No	Survived
**33BC**	8.0	0.25	0.5	0.5	1.0	0.5	---	G574S (G1720A)	---	---	CASP	Survived
**51-1BC**	4.0	0.125	0.5	0.5	0.5	0.5	---	P117S (C350Y)	---	---	CASP	Survived
**SU-103**	16.0	0.125	0.25	0.25	0.5	0.5	---	K430M (A1289W), L454P<br>(T1361Y)	---	---	CASP	Survived
**42BC**	32.0	0.125	0.5	0.5	1.0	0.5	---	P68S (C202T)<br>P135T (C403A)<br>D235N (G703R)	---	---	VRC+Nyst+FLC	Partial recovery
**74-1BC**	16.0	1.0	0.25	1.0	0.25	1.0	---	---	---	---	No	Partial recovery
**45MIR**	4.0	0.25	0.25	0.5	1.0	0.5	---	---	---	---	FLC+AMB	Survived
**SU-233**	8.0	0.125	0.5	0.5	1.0	0.5	---	---	---	---	CASP	Survived
**N126**	8.0	0.25	0.5	0.5	0.5	0.5	---	---	---	---	Not Treated	Died
**SU-79**	4.0	0.5	0.125	0.25	0.125	1.0	H430P (A1289M)	K430M (A1289W), T745A<br>(A2234G)	---	---	CASP	Survived
**SU-83**	8.0	0.25	0.125	0.25	0.125	1.0	H430P (A1289M)	Y285N (T853W), T286A<br>(A856R), K430M (A1289W),<br>T745A (A2234G)	---	---	CASP, VRC, and FLC	Survived
**SU-85**	16.0	0.125	0.25	0.25	0.125	1.0	H430P (A1289M)	K430M (A1289W), G493A<br>(G1478S), T745A (A2234G)	---	---	CASP	Survived
**N39**	4.0	0.25	0.25	1.0	0.25	0.5	---	---	---	---	AMB	Died
**N170**	16.0	0.25	0.5	1.0	0.25	0.5	N368T (C1194T)	T745A (A2234G)	---	---	FLC	Died
**N145**	16.0	0.5	1.0	1.0	0.5	0.5	---	P76S(C226T)<br>P143T(C426A)<br>D243N (G727A)	---	---	FLC	Survived
**N204**	8.0	1.0	0.5	1.0	0.5	0.5	---	---	---	---	FLC	Died
**SU-113**	16.0	0.25	0.5	0.5	0.5	2.0	---	K430M (A1289W), E441K<br>(G1321R)	---	---	No	Died

**N73**	8.0	0.25	0.5	1.0	0.5	0.5	---	P76S(C226T)<br>P143T(C427A)<br>D243N (G727A)	---	---	NO	Survived
**SU-275**	8.0	0.125	0.5	1.0	1.0	0.5	---	---	---	---	Not Treated	survived
**N196**	2.0	0.062	0.25	0.25	0.125	0.25	---	T745A (A2234G)	---	---	FLC	Died
**SU-37-B**	8.0	0.125	2.0	0.25	0.125	1.0	H430P (A1289M		---	---	Not treated	Survived
**19BC**	8.0	0.125	1.0	0.25	0.25	0.5	---	---	---	---	FLC+CASP+AMB	Partial recovery
**159MIR**	16.0	4.0	0.5	1.0	1.0	0.5	---	E555K (G1663A)	---	---	AMB	Survived
**63BC**	4.0	0.5	0.25	1.0	0.25	0.5	---	C930R (T2788Y)	---	---	No	Died
**52BC**	32.0	0.5	0.5	1.0	0.25	0.5	---	P76S(C226T)<br>P143T(C426A)<br>D243N (G727A)	---	---	AMB+CASP	Partial recovery

*52BC oimitted from AFLP due to the lack of pure DNA. Cells were directly subjected to PCR and sequencing of target genes.

*159Mir and 63BC were mixed of *C. glabrata* and *C. parapsilosis*, hence, Omitted from AFLP figure.

Supplementary Table 4. Summary of silent mutations found within ERG11, PDR1, and hotspots of FKS1 and FKS2 for isolates of *C. glabrata* recovered from patients suffering from candidemia.

**Strain number**	**PDR1**	**ERG11**	**FKS1**	**DFKS2**
**1** **N150**	C309A	---	---	---
**2** **N156**	---	G87A	---	---
**3** **N172**	C309A	T300C	---	---
**4** **N177**	T162C, T705C, T765C, C871T, T1749C, T2320A, C2579T, C2995T, A3157G	C678T, T1275C	---	---
**5** **N204**	---	C423T, C834T, T1275C	---	---
**6** **N198s**	C309A	T300C	---	---
**7** **N196**	C309A	---	---	---
**8** **N194**	C309A	T300C	---	---
**9** **N170**	C309A	T300C,	---	---
**10** **N73**	T162C, T705C, T765C, C871T, T1749C, T2320A, C2579T, C2995T, A3157G	C678T, T1275C	---	---
**11** **N39**	---	G87A, C192T	---	---
**12** **N145**	T162C, T705C, T765C, C871T, T1749C, T2320A, C2579T, C2995T, A3157G	C678T, T1276C	---	---
**13** **N144**	C310A	---	---	---
**14** **N157**	---	G87A, C192T	---	---
**15** **N126**	C309A, G936A, G1478A, C1993T	---	---	---
**16** **SU-275**	---	C423T, C834T, T1275C	---	---
**17** **SU-249**	C309A, G3070A	C834T, C849T, T1275C	---	---
**18** **SU-253**	---	G87A, C192T	---	---
**19** **SU-264**	---	G87A, C192T	---	---

**20**<br>**SU-269**	---	G87A, C192T	---	---
**21**<br>**SU-232(2)**	A489G, T1749C	---	---	---
**22**<br>**SU-246**	A489G	A399G, C834T, T1275C	---	---
**23**<br>**SU-233**	A489G	A399G, C834T, T1275C	---	---
**24**<br>**SU-227(1)**	T162C, T705C, T765C, C871T, T1749C, T2320A, C2579T, C2995T, A3157G	C678T, T1275C	---	---
**25**<br>**SU-227(2)**	T162C, T705C, T765C, C871T, T1749C, T2320A, C2579T, C2995T, A3157G	C678T, T1275C	---	---
**26**<br>**SU-260**	---	T300C<br>T1374G (heterozygous K)	---	---
**SU-261**	G87A	---		
**27**<br>**SU-247**	C309A	---	---	---
**71**<br>**SU-37-B**	A489G	A399G, C834T, T1275C	---	---
**72**<br>**SU-39**	A225R, A771R	---	---	---
**73**<br>**SU-49**	C837T, A3157G, T3229C	---	---	---
**74**<br>**SU-69**	C1563T	G87A, C192T	---	---
**75**<br>**SU-72**	C309A		---	---
**76**<br>**SU-79**	C309A, G3070A	C192T, C834T, C849T, T1275C	---	---
**77**<br>**SU-80**	C309A	---	---	---
**78**<br>**SU-83**	C309A, G3070A	C192T, C834T, C849T, T1275C	---	---
**79**<br>**SU-85**	C309A, G3070A	C192T, C834T, C849T, T1275C	---	---
**80**<br>**SU-103**	A489G	A399G, C834T, T1275C	---	---
**81**<br>**SU-113**	T1320W, A2338M	C423T, C834T, T1275C	---	---
**82**<br>**SU-119**	C1391Y, C2428Y	---	---	---
**28**<br>**2BC**	C309A	T300C,	---	---
**29**<br>**4BC**	C2160T	C588T, C834T, C918T	---	---
**30**<br>**6BC**	---	T300C, C1194T, A1367R, G1369K, G1372K	---	---
**31**	---	G87A, C192T, C834T	---	---

**19BC**				
**32**<br>**22BC**	T1080C	C597T, C600A, T1275C, A1367R, G1369K, G1372K	---	---
**33**<br>**33BC**		G87A, C192T	---	---
**34**<br>**40BC**	T138C, T681C, T741C, C847T, T1725C, T2295A, C2554T C2970T, A3132G	C678T, T1275C	---	---
**35**<br>**42BC**	T138Y, C349Y, A465G, T681Y, T741Y, T1725Y A3132R	C678T<br>T1275C	---	---
**36**<br>**43-1BC**	---	---	---	---
**37**<br>**43-2BC**	---	---	---	---
**38**<br>**44BC**		T300C,	---	---
**39**<br>**45-1BC**		G88A, C192T	---	---
**40**<br>**45-2BC**		G87A, C192T	---	---
**41**<br>**51-1BC**	C349Y, A465G	A399G, C834T, T1275C	---	---
**42**<br>**51-2BC**	---	A399G, C834T, T1275C	---	---
**43**<br>**52BC**	T163C, T705C, T765C, C871T, T1749C, T2319A, C2578T, C2994T<br>A3156G	C678T, T1275C	---	---
**44**<br>**63BC**	---	G87A, C192T	---	---
**45**<br>**69BC**	---	T300C, C1194T	---	---
**46**<br>**70BC**	---	T300C, C1194T	---	---
**47**<br>**74-1BC**	C2160T	C588T, C834T, C918T	---	---
**48**<br>**74-2BC**	C2160T	C588T, C834T, C918T	---	---
**49**<br>**81-1BC**	---	T300C, C1194T	---	---
**51**<br>**88-2BC**	A489G, C2427Y	A399G, C834T, T1275C	---	---
**52**	A489G	A399G, C834T, T1275C	---	---

**45MIR**				
**53 159MIR**	C837T	C834T, G927A	---	---
**54 348MIR**	---	T300C, C1194T	---	---
**55 692MIR**	A465G	A400G, C834T	---	---
**56 713MIR**	---	T300C, T1276C	---	---
**57 720MIR**	---	T300C, C1194T	---	---
**58 721MIR**	---	T300C, C1194T	---	---

Supplementary Table 5. Frequency of resistance to fluconazole when both *PDR1* and *ERG11* are both mutated compared to wild type of both genes and mutation in either of *pdr1* and *ERG11*.

**Polymorphism in ERG11**	**Polymorphism in PDR1**	**SDD%**	**R%**	**# of isolates along with their MIC values (µg/ml)**										**Total**
				**≤0.5**	**1**	**2**	**4**	**8**	**16**	**32**	**64**	**128**	**≥256**	
WT and/or mutation in either of genes						1	8	21	18	5	1			54
N368T	T745A							2	2					4
D196N	A1004S							1						1
H430P	G189V							1						1
H430P	G128E, G493A							1						1
H430P	K430M, T745A						1							1
H430P	G128E								1					1
H430P	Y285N, T286A, K430M, T745A							1						1
H430P	K430M, G493A, T745A								1					1
N368T	K430M						1							1
N368T	T745A, C930R								1					1
K456R, G457C, V458F	N162S, F944S							1						1
N368T	C930R						1		1					2

Supplementary Figure 1. Clinical centers involved in this study

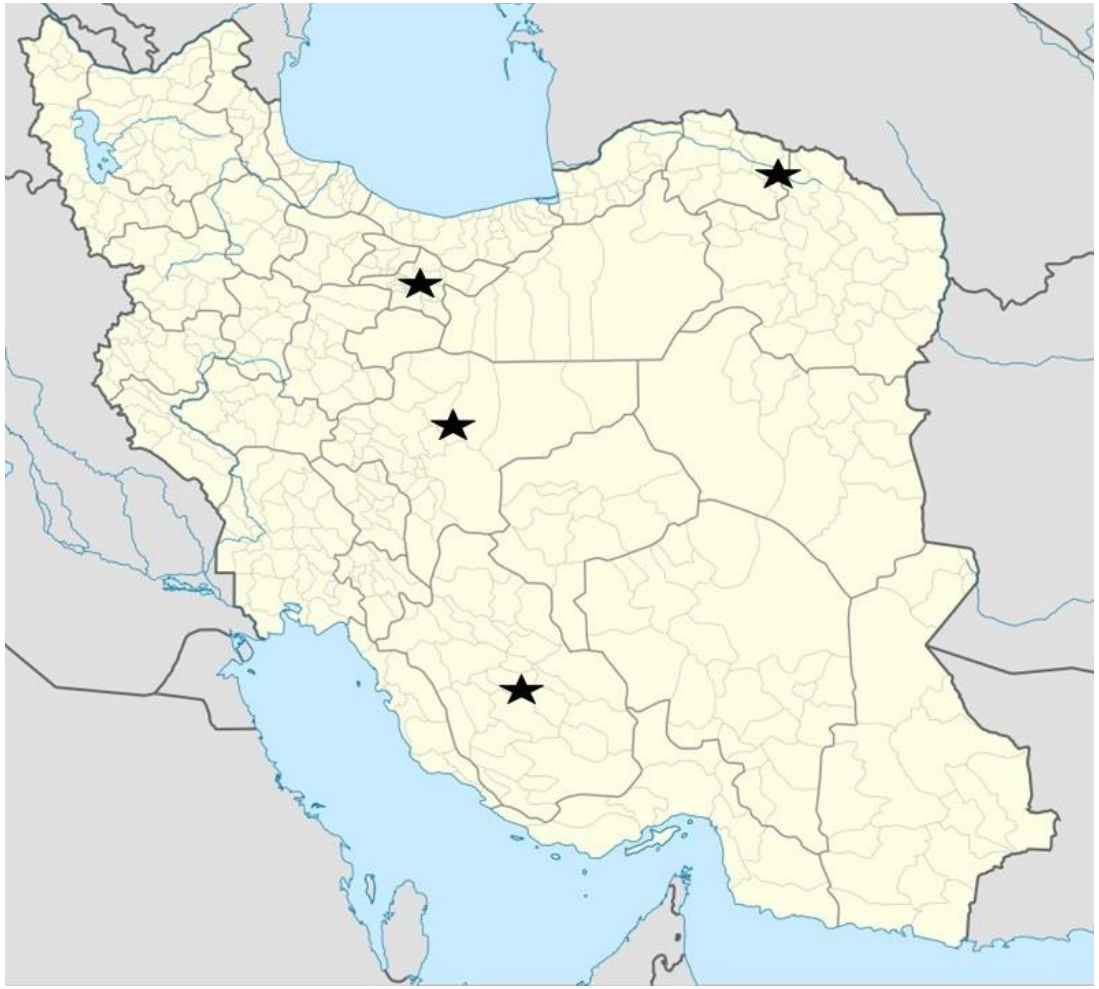

Supplementary Figure 2. Amplified PCR products of target genes

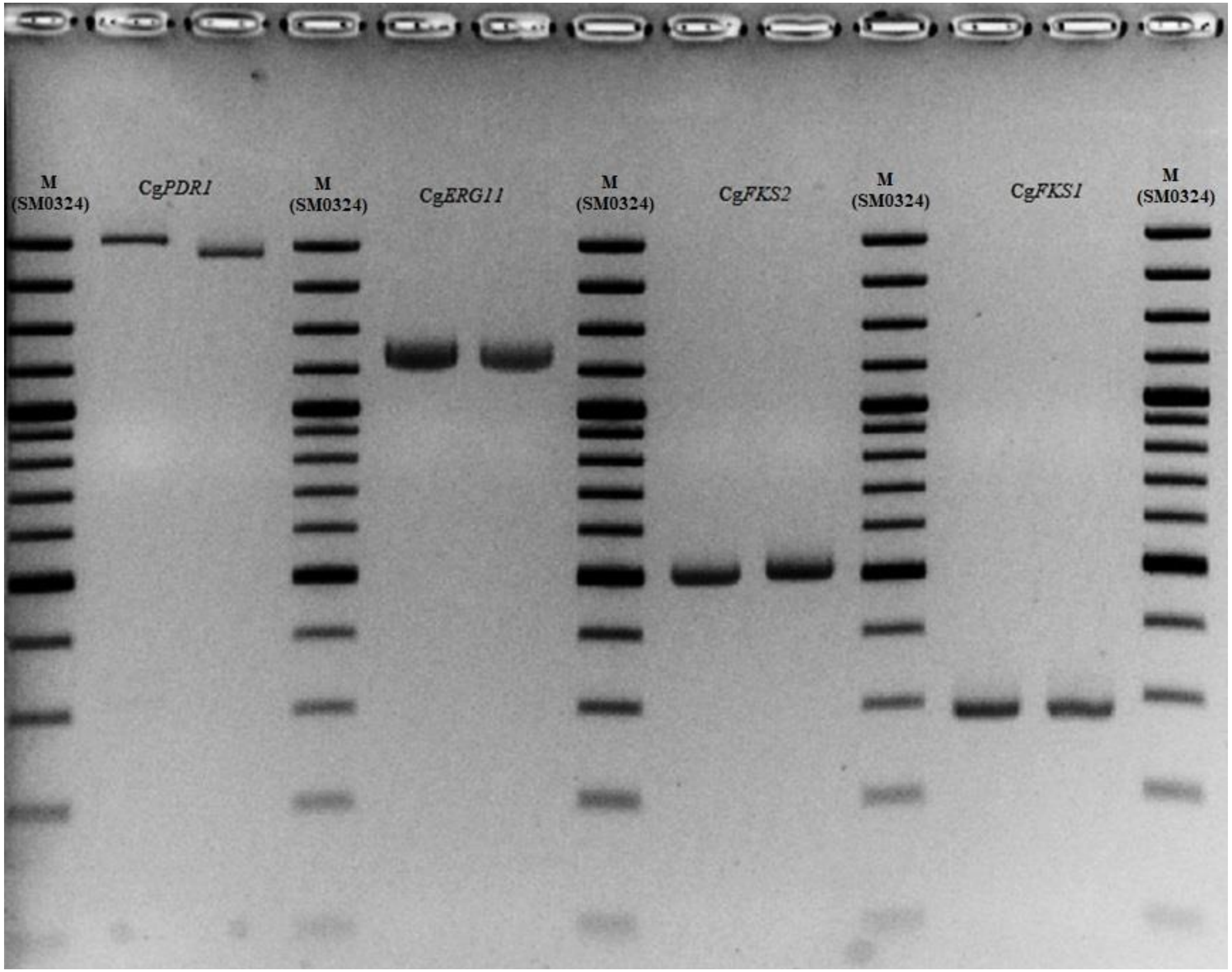

Supplemenatry Figure 3. Position of mutations in target genes

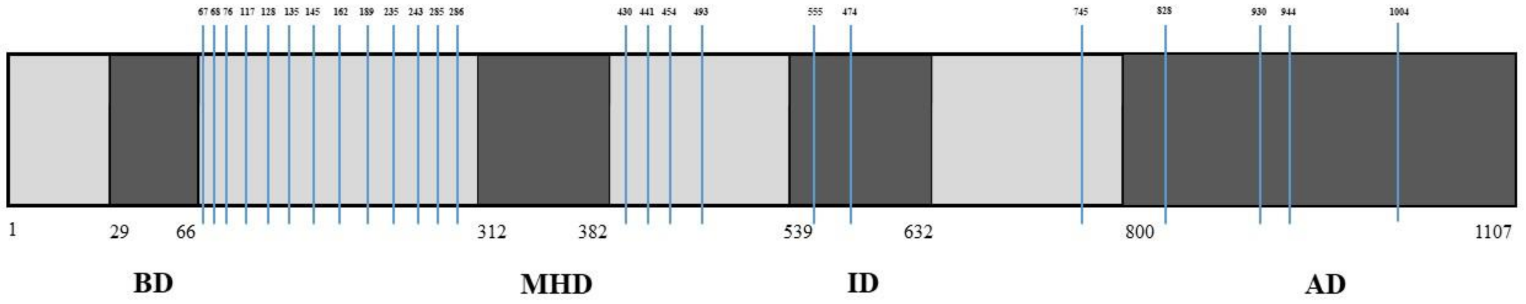

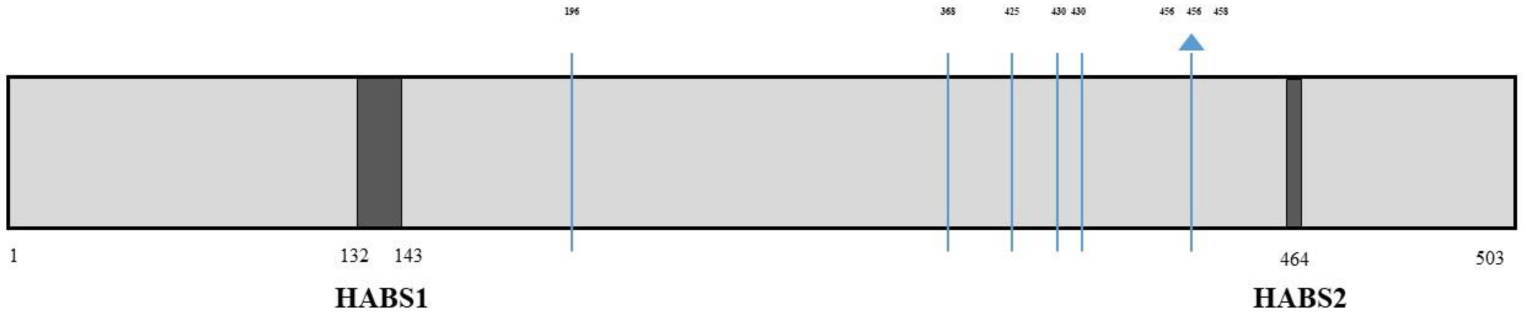

BD, Binding domain, MHD, Middle homology domain, ID, Inhibitory domain, AD, activatory domain, HABS, Hypothetical azole binding sites

Reference

1. Healey KR, Zhao Y, Perez WB, Lockhart SR, Sobel JD, Farmakiotis D, Kontoyiannis DP, Sanglard D, Taj-Aldeen SJ, Alexander BD, Jimenez-Ortigosa C, Shor E, Perlin DS. 2016. Prevalent mutator genotype identified in fungal pathogen Candida glabrata promotes multi-drug resistance. Nat Commun 7:11128.

G1=15, G2=26, G3=10, G4=3, G5=2, G6=4, G7=3, G8=1, G9=1

The following repetitive isolates due to being placed in different genotypes were deleted; 74-1BC, 74-2BC, 51-1BC, 51-2BC

Patient number	VRZ MIC	Clinical failure	Hospitalization duration stay (days)	Genotype	Outcome
**SU-264**	R	No	21	G1	Survived
**SU-269**	R	No	10	G1	Survived
**N157**	S	No	35	G1	Survived
**45-2BC**	S	Yes	174	G1	Survived
**SU-69**	S	No	44	G1	Survived
**45-1BC**	S	Yes	174	G1	Survived
**Su 261**	R	No	20	G1	Survived
**SU-253**	S	Yes	16	G1	Died
**SU-39**	S	No	10	G1	Survived
**N156**	S	No	33	G1	Survived
**SU-249**	S	No	2	G1	Died
**SU-49**	R	No	156	G1	Died
**SU-227(1)**	S	Yes	32	G1	Survived
**40BC**	S	No	42	G1	Died
**N177**	S	No	19	G1	Died
**720MIR**	S	No	23	G2	Survived
**713MIR**	R	No	21	G2	Died
**69BC**	S	No	23	G2	Survived
**348MIR**	R	No	49	G2	Survived
**SU-119**	S	No	53	G2	Survived
**44BC**	S	No	15	G2	Survived
**4BC**	R	No	14	G2	Died
**SU-260**	R	No	6	G2	Survived
**N198s**	R	Yes	81	G2	Died
**70BC**	S	No	3	G2	Survived
**SU-72**	R	No	81	G2	Survived
**SU-80**	S	No	81	G2	Survived
**N144**	S	Yes	54	G2	Died
**SU-247**	R	No	6	G2	Died
**43-1BC**	R	No	39	G2	Survived
**6BC**	S	No	43	G2	Died

**81-1BC**	S	Yes	16	G2	Died
**SU-232(2)**	S	No	25	G2	Survived
**N194**	R	No	24	G2	Survived
**N150**	R	Yes	32	G2	Died
**2BC**	R	Yes	28	G2	Died
**N172**	R	No	14	G2	Died
**721MIR**	R	No	41	G2	survived
**22BC**	S	No	47	G2	Died
**SU-246**	S	No	18	G3	Survived
**692MIR**	R	No	29	G3	Survived
**88-2BC**	R	No	7	G3	Survived
**33BC**	S	No	20	G3	Survived
**SU-103**	S	No	26	G3	Survived
**42BC**	S	No	126	G3	Survived
**45MIR**	S	Yes	58	G3	Survived
**SU-233**	S	No	26	G3	Survived
**N126**	S	Not treated	22	G8	Died
**SU-79**	R	Yes	28	G7	Survived
**SU-83**	S	Yes	28	G7	Survived
**SU-85**	S	Yes	28	G7	Survived
**N39**	S	No	42	G4	Died
**N170**	S	No	7	G4	Died
**N145**	R	No	12	G4	Survived
**N204**	R	No	11	G5	Died
**SU-113**	S	No	4	G5	Died
**N73**	S	Not treated	48	G6	Survived
**SU-275**	S	No	18	G6	survived
**N196**	S	No	10	G6	Died
**SU-37-B**	S	No	35	G6	Survived
**19BC**	S	Yes	60	G9	Survived
**159MIR**	R	No	36	Not assigned	Survived
**63BC**	R	No	16	Not assigned	Died
**52BC**	R	No	24	Not assigned	Survived

VRZ susceptibility pattern and clinical outcome association

Crosstab

			outcome		
			Died	Survive	Total
vrz	1	Count	10	17	27
		% within vrz	37.0%	63.0%	100.0%
	2	Count	13	30	43
		% within vrz	30.2%	69.8%	100.0%
Total		Count	23	47	70
		% within vrz	32.9%	67.1%	100.0%

Chi-Square Tests

	Value	df	Asymp. Sig. (2-sided)	Exact Sig. (2-sided)	Exact Sig. (1-sided)
Pearson Chi-Square	.348[a]	1	.555	.607	.369
Continuity Correction[b]	.108	1	.742		
Likelihood Ratio	.346	1	.556	.607	.369

Fisher's Exact Test					.607	.369
N of Valid Cases	70					

a. 0 cells (0.0%) have expected count less than 5. The minimum expected count is 8.87.

b. Computed only for a 2x2 table

Chi-square test showed no significant association between clinical outcome and VRZ susceptibility profile (P value 0.555, α<0.05).

Repetitive isolates of SU-227 and 43BC were regarded as a single case/genotype. Moreover, G4-G8 (highlighted in red color) due to limited numbers of cases were not considered for further analysis.

**Genotype**	**Outcome**
G1	Survived
G1	Survived
G1	Survived
G1	Survived
G1	Survived
G1	Survived
G1	Survived
G1	Died
G1	Survived
G1	Survived
G1	Died
G1	Died
G1	Survived
G1	Died
G1	Died
G2	Survived
G2	Died
G2	Survived

G2	Survived
G2	Survived
G2	Survived
G2	Died
G2	Survived
G2	Died
G2	Survived
G2	Survived
G2	Survived
G2	Died
G2	Died
G2	Survived
G2	Died
G2	Died
G2	Survived
G2	Survived
G2	Died
G2	Died
G2	Died
G2	survived
G2	Died
G3	Survived
G3	Survived
G3	Survived
G3	Survived
G3	Survived
G3	Survived
G3	Survived
G3	Survived
G8	Died
G7	Survived
G7	Survived
G7	Survived
G4	Died
G4	Died
G4	Survived
G5	Died

G5	Died
G6	Survived
G6	survived
G6	Died
G6	Survived
G9	Survived
Not assigned	Survived
Not assigned	Died
Not assigned	Survivedz

Clinical outcome and genotype Crosstabulation (3-by-2)

Count

		Outcome		
		Died	Survived	Total
Genotype	G1	5	10	15
	G2	11	13	24
	G3	0	8	8
Total		16	31	47

Chi-Square Tests

	Value	df	Asymptotic Significance (2-sided)
Pearson Chi-Square	5.618[a]	2	.060
Likelihood Ratio	8.084	2	.018
Linear-by-Linear Association	1.360	1	.244
N of Valid Cases	47		

a. 1 cells (16.7%) have expected count less than 5. The minimum expected count is 2.72.

Clinical outcome and G1

Crosstab

Count

		G1		
		no	yes	Total
Outcome	Died	11	5	16
	Survived	21	10	31
Total		32	15	47

Chi-Square Tests

	Value	df	Asymptotic Significance (2-sided)	Exact Sig. (2-sided)	Exact Sig. (1-sided)
Pearson Chi-Square	.005[a]	1	.944		
Continuity Correction[b]	.000	1	1.000		
Likelihood Ratio	.005	1	.944		
Fisher's Exact Test				1.000	.607
Linear-by-Linear Association	.005	1	.945		
N of Valid Cases	47				

a. 0 cells (0.0%) have expected count less than 5. The minimum expected count is 5.11.

b. Computed only for a 2x2 table

Clinical outcome and G2

Crosstab

Count

		G2		
		no	yes	Total
Outcome	Died	5	11	16
	Survived	18	13	31
Total		23	24	47

Chi-Square Tests

	Value	df	Asymptotic Significance (2-sided)	Exact Sig. (2-sided)	Exact Sig. (1-sided)
Pearson Chi-Square	3.037[a]	1	.081		
Continuity Correction[b]	2.058	1	.151		
Likelihood Ratio	3.095	1	.079		
Fisher's Exact Test				.125	.075
Linear-by-Linear Association	2.972	1	.085		
N of Valid Cases	47				

a. 0 cells (0.0%) have expected count less than 5. The minimum expected count is 7.83.

b. Computed only for a 2x2 table

Clinical outcome and G3

Crosstab

Count

		G3		
		no	yes	Total
Outcome	Died	16	0	16
	Survived	23	8	31
Total		39	8	47

Chi-Square Tests

	Value	df	Asymptotic Significance (2-sided)	Exact Sig. (2-sided)	Exact Sig. (1-sided)
Pearson Chi-Square	4.976[a]	1	.026		
Continuity Correction[b]	3.317	1	.069		
Likelihood Ratio	7.482	1	.006		
Fisher's Exact Test				.038	.025
Linear-by-Linear Association	4.870	1	.027		
N of Valid Cases	47				

Logistic regression and path analysis to find the association between mortality and survival

Multivariate logistic regression analysis was used to find/predict a linear association between independent and dependent variables. However, multivariate logistic regression analysis can only predict the direct influence of independent variables over dependent variables and it is not possible to assess the indirect influence of independent variables over dependent ones. In these situations, establishing association between independent variables is not possible. In order to overcome this problem the path analysis was used, in which the association between genotypes (G1, G2, and G3) was individually assessed with mortality and survival.

			Score	df	Sig.
Step 0	Variables	G1	.005	1	.042
		G2	0.337	1	.031
		G3	0.076	1	.026

1. First mortality was considered as the dependent variable, which showed a significant association with G1 and G2.

Variables in the Equation

		B	S.E.	Wald	df	Sig.	Exp(B)	95% C.I.for EXP(B)	
								Lower	Upper
Step 1[a]	G1	-22.746	.793	. 921	1	.034	2.510	. 349	10.271
	G2	-20.612	.789	. 582	1	.022	1.831	. 431	7.811
	G3	16.920	.604	. 403	1	.181	.669		

a. Variable(s) entered on step 1: G1, G2, G3

2. In the next step, survival was considered as the dependent variable, which was significantly associated with G3.

Variables in the Equation

		B	S.E.	Wald	df	Sig.	Exp(B)	95% C.I.for EXP(B)	
								Lower	Upper
Step 1[a]	G1	-19.281	1.618	.481	1	.073	2.412	.	
	G2	-17.281	2.491	.539	1	.122	1.759		
	G3	10.405	2.815	.859	1	.001	.667	.531	9.718

a. Variable(s) entered on step 1: G1, G2, G3

Hospitalization duration and mortality

Following repetitive isolates were presented as a single case, 43BC, 51BC, 74BC, and SU-227.

Patient number	Hospitalization duration stay (days)	Outcome
**SU-264**	21	Survived
**SU-269**	10	Survived
**51BC**	115	Survived
**74BC**	7	Survived
**N157**	35	Survived
**45-2BC**	174	Survived
**SU-69**	44	Survived
**45-1BC**	174	Survived
**Su 261**	20	Survived

**SU-253**	16	Died
**SU-39**	10	Survived
**N156**	33	Survived
**SU-249**	2	Died
**SU-49**	156	Died
**SU-227(1)**	32	Survived
**40BC**	42	Died
**N177**	19	Died
**720MIR**	23	Survived
**713MIR**	21	Died
**69BC**	23	Survived
**348MIR**	49	Survived
**SU-119**	53	Survived
**44BC**	15	Survived
**4BC**	14	Died
**SU-260**	6	Survived
**N198s**	81	Died
**70BC**	3	Survived
**SU-72**	81	Survived
**SU-80**	81	Survived
**N144**	54	Died
**SU-247**	6	Died
**43-1BC**	39	Survived
**6BC**	43	Died
**81-1BC**	16	Died
**SU-232(2)**	25	Survived
**N194**	24	Survived
**N150**	32	Died
**2BC**	28	Died
**N172**	14	Died
**721MIR**	41	survived
**22BC**	47	Died
**SU-246**	18	Survived
**692MIR**	29	Survived
**88-2BC**	7	Survived
**33BC**	20	Survived
**SU-103**	26	Survived

**42BC**	126	Survived
**45MIR**	58	Survived
**SU-233**	26	Survived
**N126**	22	Died
**SU-79**	28	Survived
**SU-83**	28	Survived
**SU-85**	28	Survived
**N39**	42	Died
**N170**	7	Died
**N145**	12	Survived
**N204**	11	Died
**SU-113**	4	Died
**N73**	48	Survived
**SU-275**	18	survived
**N196**	10	Died
**SU-37-B**	35	Survived
**19BC**	60	Survived
**159MIR**	36	Survived
**63BC**	16	Died
**52BC**	24	Survived

Logistic regression revealed lack of association between hospitalization duration and survival.

Variables in the Equation

		B	S.E.	Wald	df	Sig.	Exp(B)
Step 1[a]	Hosdur	-.009	.008	1.114	1	.291	.991
	Constant	-.314	.379	.685	1	.408	.731

a. Variable(s) entered on step 1: Hosdur.

Two-step correlation coefficient test showed lack of significant association between hospitalization duration and clinical outcome.

Symmetric Measures

	Value	Asymptotic Standard Error[a]	Approximate T[b]	Approximate Significance
**point- biserial correlation coefficient**	-.219	.137	-1.601	.109
N of Valid Cases	66			

Supplementary text file 6-9. Sequences of target genes obtained for this study

ERG11 Sequences

>1

ATGTCCACTGAAAACACTTCTTTGGTCGTTGAACTATTGGAGTACGTGAAGCTTGGTCTTTCGTACTTCCAAGCTCTGCCATTGGCGCAGAGAGTGTCTATTATGGTCGCCTTGC
CATTTGTGTACACCATCACATGGCAATTGCTTTACTCCTTGAGAAAGGACAGACCACCACTTGTGTTCTACTGGATCCCATGGGTCGGCTCTGCTATCCCATACGGTACCAAGCC
ATACGAGTTCTTCGAAGACTGCCAAAAGAAATACGGTGATATCTTCTCTTTCATGCTATTGGGTAGAATTATGACTGTCTACTTGGGTCCAAAGGGTCACGAATTCATCTTCAAC
GCCAAGTTGGCCGATGTTTCCGCTGAAGCTGCTTACTCCCACTTGACCACCCCAGTGTTCGGTAAAGGTGTTATCTACGATTGTCCAAACCACAGACTAATGGAACAAAAGAAG
TTTGTCAAGGGTGCTTTGACTAAGGAAGCCTTTGTCAGATACGTTCCATTGATCGCTGAGGAAATCTACAAGTACTTCAGAAACTCCAAGAACTTCAAGATCAACGAAAACAAC
TCCGGTATCGTCGACGTTATGGTCTCCCAACCTGAAATGACTATCTTCACTGCTTCCAGATCCTTGCTAGGTAAGGAAATGAGAGACAAGTTGGACACCGACTTCGCTTACTTGT
ACAGTGACTTGGACAAGGGTTTCACCCCAATTAACTTCGTCTTCCCTAACTTGCCTCTAGAACACTACAGAAAGAGAGACCATGCCCAACAAGCTATCTCTGGTACTTACATGTC
CTTGATTAAGGAAAGACGTGAGAAGAACGACATCCAAAACCGTGACTTGATTGATGAATTGATGAAGAACTCCACTTACAAGGATGGTACTAAGATGACCGACCAAGAAATT
GCCAACCTATTGATTGGTGTCTTGATGGGTGGTCAACATACTTCCGCTGCTACCTCCGCTTGGTGTCTATTGCATTTGGCTGAAAGACCAGATGTCCAAGAAGAATTGTACCAA

GAACAAATGCGCGTCTTGAACAACGATACCAAGGAATTGACTTACGATGACCTACAAAACATGCCTCTATTGAACCAAATGATCAAGGAAACTTTGAGATTGCACCACCCATTG
CACTCTTTGTTCCGTAAAGTCATGAGAGATGTCGCTATTCCAAACACTTCCTACGTTGTCCCAAGGGACTACCACGTTCTAGTCTCCCCAGGTTACACTCACTTGCAAGAAGAATT
CTTCCCTAAGCCAATTGAATTCAACATCCACCGTTGGGACGGTGATGCTGCTTCTTCCAGTGCTGCTGGTGGTGACGAAGTTGATTACGGTTTCGGTGCTATCTCCAAGGGTGTT
TCCTCTCCATACTTGCCATTCGGTGGTGGTAGACACAGATGTATCGGTGAATTGTTCGCTTACTGTCAATTGGGTGTGTTGATGTCCATTTTCATCAGAACCATGAAATGGCGTT
ACCCAACTGAAGGTGAAACTGTCCCACCATCTGACTTCACCTCCATGGTCACCCTACCAACTGCCCCAGCTAAGATCTACTGGGAAAAGAGACATCCAGAACAAAAGTACTAG-

>2

ATGTCCACTGAAAACACTTCTTTGGTCGTTGAACTATTGGAGTACGTGAAGCTTGGTCTTTCGTACTTCCAAGCTCTGCCATTGGCACAGAGAGTGTCTATTATGGTCGCCTTGC
CATTTGTGTACACCATCACATGGCAATTGCTTTACTCCTTGAGAAAGGACAGACCACCACTTGTGTTCTACTGGATTCCATGGGTCGGCTCTGCTATCCCATACGGTACCAAGCC
ATACGAGTTCTTCGAAGACTGCCAAAAGAAATACGGTGATATCTTCTCTTTCATGCTATTGGGTAGAATTATGACTGTCTACTTGGGTCCAAAGGGTCACGAATTCATCTTCAAC
GCCAAGTTGGCCGATGTTTCCGCTGAAGCTGCTTACTCCCACTTGACCACCCCAGTGTTCGGTAAAGGTGTTATCTACGATTGTCCAAACCACAGACTAATGGAACAAAAGAAG
TTTGTCAAGGGTGCTTTGACTAAGGAAGCCTTTGTCAGATACGTTCCATTGATCGCTGAGGAAATCTACAAGTACTTCAGAAACTCCAAGAACTTCAAGATCAACGAAAACAAC
TCCGGTATCGTCGACGTTATGGTCTCCCAACCTGAAATGACTATCTTCACTGCTTCCAGATCCTTGCTAGGTAAGGAAATGAGAGACAAGTTGGACACCGACTTCGCTTACTTGT
ACAGTGACTTGGACAAGGGTTTCACCCCAATTAACTTCGTCTTCCCTAACTTGCCTCTAGAACACTACAGAAAGAGAGACCATGCCCAACAAGCTATCTCTGGTACTTACATGTC
CTTGATTAAGGAAAGACGTGAGAAGAACGACATCCAAAACCGTGACTTGATTGATGAATTGATGAAGAACTCCACTTACAAGGATGGTACTAAGATGACCGACCAAGAAATT
GCCAACCTATTGATTGGTGTCTTGATGGGTGGTCAACATACTTCCGCTGCTACCTCCGCTTGGTGTCTATTGCATTTGGCTGAAAGACCAGATGTCCAAGAAGAATTGTACCAA
GAACAAATGCGCGTCTTGAACAACGATACCAAGGAATTGACTTACGATGACCTACAAAACATGCCTCTATTGAACCAAATGATCAAGGAAACTTTGAGATTGCACCACCCATTG
CACTCTTTGTTCCGTAAAGTCATGAGAGATGTCGCTATTCCAAACACTTCCTACGTTGTCCCAAGGGACTACCACGTTCTAGTCTCCCCAGGTTACACTCACTTGCAAGAAGAATT
CTTCCCTAAGCCAAATGAATTCAACATCCACCGTTGGGACGGTGATGCTGCTTCTTCCAGTGCTGCTGGTGGTGACGAAGTTGATTACGGTTTCGGTGCTATCTCCAAGGGTGT
TTCCTCTCCATACTTGCCATTCGGTGGTGGTAGACACAGATGTATCGGTGAATTGTTCGCTTACTGTCAATTGGGTGTGTTGATGTCCATTTTCATCAGAACCATGAAATGGCGT
TACCCAACTGAAGGTGAAACTGTCCCACCATCTGACTTCACCTCCATGGTCACCCTACCAACTGCCCCAGCTAAGATCTACTGGGAAAAGAGACATCCAGAACAAAAGTACTAG
-

>3

ATGTCCACTGAAAACACTTCTTTGGTCGTTGAACTATTGGAGTACGTGAAGCTTGGTCTTTCGTACTTCCAAGCTCTGCCATTGGCGCAGAGAGTGTCTATTATGGTCGCCTTGC
CATTTGTGTACACCATCACATGGCAATTGCTTTACTCCTTGAGAAAGGACAGACCACCACTTGTGTTCTACTGGATCCCATGGGTCGGCTCTGCTATCCCATACGGTACCAAGCC
ATACGAGTTCTTCGAAGACTGCCAAAAGAAATACGGTGATATCTTCTCTTTCATGCTATTGGGTAGAATCATGACTGTCTACTTGGGTCCAAAGGGTCACGAATTCATCTTCAAC
GCCAAGTTGGCCGATGTTTCCGCTGAAGCTGCTTACTCCCACTTGACCACCCCAGTGTTCGGTAAAGGTGTTATCTACGATTGTCCAAACCACAGACTAATGGAACAAAAGAAG
TTTGTCAAGGGTGCTTTGACTAAGGAAGCCTTTGTCAGATACGTTCCATTGATCGCTGAGGAAATCTACAAGTACTTCAGAAACTCCAAGAACTTCAAGATCAACGAAAACAAC
TCCGGTATCGTCGACGTTATGGTCTCCCAACCTGAAATGACTATCTTCACTGCTTCCAGATCCTTGCTAGGTAAGGAAATGAGAGACAAGTTGGACACCGACTTCGCTTACTTGT
ACAGTGACTTGGACAAGGGTTTCACCCCAATTAACTTCGTCTTCCCTAACTTGCCTCTAGAACACTACAGAAAGAGAGACCATGCCCAACAAGCTATCTCTGGTACTTACATGTC

CTTGATTAAGGAAAGACGTGAGAAGAACGACATCCAAAACCGTGACTTGATTGATGAATTGATGAAGAACTCCACTTACAAGGATGGTACTAAGATGACCGACCAAGAAATT
GCCAACCTATTGATTGGTGTCTTGATGGGTGGTCAACATACTTCCGCTGCTACCTCCGCTTGGTGTCTATTGCATTTGGCTGAAAGACCAGATGTCCAAGAAGAATTGTACCAA
GAACAAATGCGCGTCTTGAACAACGATACCAAGGAATTGACTTACGATGACCTACAAAACATGCCTCTATTGAACCAAATGATCAAGGAAACTTTGAGATTGCACCACCCATTG
CACTCTTTGTTCCGTAAAGTCATGAGAGATGTCGCTATTCCAAACACTTCTTACGTTGTCCCAAGGGACTACCACGTTCTAGTCTCCCCAGGTTACACTCACTTGCAAGAAGAATT
CTTCCCTAAGCCAAATGAATTCAACATCCACCGTTGGGACGGTGATGCTGCTTCTTCCAGTGCTGCTGGTGGTGACGAAGTTGATTACGGTTTCGGTGCTATCTCCAAGGGTGT
TTCCTCTCCATACTTGCCATTCGGTGGTGGTAGACACAGATGTATCGGTGAATTGTTCGCTTACTGTCAATTGGGTGTGTTGATGTCCATTTTCATCAGAACCATGAAATGGCGT
TACCCAACTGAAGGTGAAACTGTCCCACCATCTGACTTCACCTCCATGGTCACCCTACCAACTGCCCCAGCTAAGATCTACTGGGAAAAGAGACATCCAGAACAAAAGTACTAG
-

>6

ATGTCCACTGAAAACACTTCTTTGGTCGTTGAACTATTGGAGTACGTGAAGCTTGGTCTTTCGTACTTCCAAGCTCTGCCATTGGCGCAGAGAGTGTCTATTATGGTCGCCTTGC
CATTTGTGTACACCATCACATGGCAATTGCTTTACTCCTTGAGAAAGGACAGACCACCACTTGTGTTCTACTGGATCCCATGGGTCGGCTCTGCTATCCCATACGGTACCAAGCC
ATACGAGTTCTTCGAAGACTGCCAAAAGAAATACGGTGATATCTTCTCTTTCATGCTATTGGGTAGAATCATGACTGTCTACTTGGGTCCAAAGGGTCACGAATTCATCTTCAAC
GCCAAGTTGGCCGATGTTTCCGCTGAAGCTGCTTACTCCCACTTGACCACCCCAGTGTTCGGTAAAGGTGTTATCTACGATTGTCCAAACCACAGACTAATGGAACAAAAGAAG
TTTGTCAAGGGTGCTTTGACTAAGGAAGCCTTTGTCAGATACGTTCCATTGATCGCTGAGGAAATCTACAAGTACTTCAGAAACTCCAAGAACTTCAAGATCAACGAAAACAAC
TCCGGTATCGTCGACGTTATGGTCTCCCAACCTGAAATGACTATCTTCACTGCTTCCAGATCCTTGCTAGGTAAGGAAATGAGAGACAAGTTGGACACCGACTTCGCTTACTTGT
ACAGTGACTTGGACAAGGGTTTCACCCCAATTAACTTCGTCTTCCCTAACTTGCCTCTAGAACACTACAGAAAGAGAGACCATGCCCAACAAGCTATCTCTGGTACTTACATGTC
CTTGATTAAGGAAAGACGTGAGAAGAACGACATCCAAAACCGTGACTTGATTGATGAATTGATGAAGAACTCCACTTACAAGGATGGTACTAAGATGACCGACCAAGAAATT
GCCAACCTATTGATTGGTGTCTTGATGGGTGGTCAACATACTTCCGCTGCTACCTCCGCTTGGTGTCTATTGCATTTGGCTGAAAGACCAGATGTCCAAGAAGAATTGTACCAA
GAACAAATGCGCGTCTTGAACAACGATACCAAGGAATTGACTTACGATGACCTACAAAACATGCCTCTATTGAACCAAATGATCAAGGAAACTTTGAGATTGCACCACCCATTG
CACTCTTTGTTCCGTAAAGTCATGAGAGATGTCGCTATTCCAAACACTTCTTACGTTGTCCCAAGGGACTACCACGTTCTAGTCTCCCCAGGTTACACTCACTTGCAAGAAGAATT
CTTCCCTAAGCCAAATGAATTCAACATCCACCGTTGGGACGGTGATGCTGCTTCTTCCAGTGCTGCTGGTGGTGACGAAGTTGATTACGGTTTCGGTGCTATCTCCAAGGGTGT
TTCCTCTCCATACTTGCCATTCGGTGGTGGTAGACACAGATGTATCGGTGAATTGTTCGCTTACTGTCAATTGGGTGTGTTGATGTCCATTTTCATCAGAACCATGAAATGGCGT
TACCCAACTGAAGGTGAAACTGTCCCACCATCTGACTTCACCTCCATGGTCACCCTACCAACTGCCCCAGCTAAGATCTACTGGGAAAAGAGACATCCAGAACAAAAGTACTAG
-

>8

ATGTCCACTGAAAACACTTCTTTGGTCGTTGAACTATTGGAGTACGTGAAGCTTGGTCTTTCGTACTTCCAAGCTCTGCCATTGGCGCAGAGAGTGTCTATTATGGTCGCCTTGC
CATTTGTGTACACCATCACATGGCAATTGCTTTACTCCTTGAGAAAGGACAGACCACCACTTGTGTTCTACTGGATCCCATGGGTCGGCTCTGCTATCCCATACGGTACCAAGCC
ATACGAGTTCTTCGAAGACTGCCAAAAGAAATACGGTGATATCTTCTCTTTCATGCTATTGGGTAGAATCATGACTGTCTACTTGGGTCCAAAGGGTCACGAATTCATCTTCAAC
GCCAAGTTGGCCGATGTTTCCGCTGAAGCTGCTTACTCCCACTTGACCACCCCAGTGTTCGGTAAAGGTGTTATCTACGATTGTCCAAACCACAGACTAATGGAACAAAAGAAG

TTTGTCAAGGGTGCTTTGACTAAGGAAGCCTTTGTCAGATACGTTCCATTGATCGCTGAGGAAATCTACAAGTACTTCAGAAACTCCAAGAACTTCAAGATCAACGAAAACAAC
TCCGGTATCGTCGACGTTATGGTCTCCCAACCTGAAATGACTATCTTCACTGCTTCCAGATCCTTGCTAGGTAAGGAAATGAGAGACAAGTTGGACACCGACTTCGCTTACTTGT
ACAGTGACTTGGACAAGGGTTTCACCCCAATTAACTTCGTCTTCCCTAACTTGCCTCTAGAACACTACAGAAAGAGAGACCATGCCCAACAAGCTATCTCTGGTACTTACATGTC
CTTGATTAAGGAAAGACGTGAGAAGAACGACATCCAAAACCGTGACTTGATTGATGAATTGATGAAGAACTCCACTTACAAGGATGGTACTAAGATGACCGACCAAGAAATT
GCCAACCTATTGATTGGTGTCTTGATGGGTGGTCAACATACTTCCGCTGCTACCTCCGCTTGGTGTCTATTGCATTTGGCTGAAAGACCAGATGTCCAAGAAGAATTGTACCAA
GAACAAATGCGCGTCTTGAACAACGATACCAAGGAATTGACTTACGATGACCTACAAAACATGCCTCTATTGAACCAAATGATCAAGGAAACTTTGAGATTGCACCACCCATTG
CACTCTTTGTTCCGTAAAGTCATGAGAGATGTCGCTATTCCAAACACTTCTTACGTTGTCCCAAGGGACTACCACGTTCTAGTCTCCCCAGGTTACACTCACTTGCAAGAAGAATT
CTTCCCTAAGCCAAATGAATTCAACATCCACCGTTGGGACGGTGATGCTGCTTCTTCCAGTGCTGCTGGTGGTGACGAAGTTGATTACGGTTTCGGTGCTATCTCCAAGGGTGT
TTCCTCTCCATACTTGCCATTCGGTGGTGGTAGACACAGATGTATCGGTGAATTGTTCGCTTACTGTCAATTGGGTGTGTTGATGTCCATTTTCATCAGAACCATGAAATGGCGT
TACCCAACTGAAGGTGAAACTGTCCCACCATCTGACTTCACCTCCATGGTCACCCTACCAACTGCCCCAGCTAAGATCTACTGGGAAAAGAGACATCCAGAACAAAAGTACTAG
-

>9

ATGTCCACTGAAAACACTTCTTTGGTCGTTGAACTATTGGAGTACGTGAAGCTTGGTCTTTCGTACTTCCAAGCTCTGCCATTGGCGCAGAGAGTGTCTATTATGGTCGCCTTGC
CATTTGTGTACACCATCACATGGCAATTGCTTTACTCCTTGAGAAAGGACAGACCACCACTTGTGTTCTACTGGATCCCATGGGTCGGCTCTGCTATCCCATACGGTACCAAGCC
ATACGAGTTCTTCGAAGACTGCCAAAAGAAATACGGTGATATCTTCTCTTTCATGCTATTGGGTAGAATCATGACTGTCTACTTGGGTCCAAAGGGTCACGAATTCATCTTCAAC
GCCAAGTTGGCCGATGTTTCCGCTGAAGCTGCTTACTCCCACTTGACCACCCCAGTGTTCGGTAAAGGTGTTATCTACGATTGTCCAAACCACAGACTAATGGAACAAAAGAAG
TTTGTCAAGGGTGCTTTGACTAAGGAAGCCTTTGTCAGATACGTTCCATTGATCGCTGAGGAAATCTACAAGTACTTCAGAAACTCCAAGAACTTCAAGATCAACGAAAACAAC
TCCGGTATCGTCGACGTTATGGTCTCCCAACCTGAAATGACTATCTTCACTGCTTCCAGATCCTTGCTAGGTAAGGAAATGAGAGACAAGTTGGACACCGACTTCGCTTACTTGT
ACAGTGACTTGGACAAGGGTTTCACCCCAATTAACTTCGTCTTCCCTAACTTGCCTCTAGAACACTACAGAAAGAGAGACCATGCCCAACAAGCTATCTCTGGTACTTACATGTC
CTTGATTAAGGAAAGACGTGAGAAGAACGACATCCAAAACCGTGACTTGATTGATGAATTGATGAAGAACTCCACTTACAAGGATGGTACTAAGATGACCGACCAAGAAATT
GCCAACCTATTGATTGGTGTCTTGATGGGTGGTCAACATACTTCCGCTGCTACCTCCGCTTGGTGTCTATTGCATTTGGCTGAAAGACCAGATGTCCAAGAAGAATTGTACCAA
GAACAAATGCGCGTCTTGAACAACGATACCAAGGAATTGACTTACGATGACCTACAAAACATGCCTCTATTGAACCAAATGATCAAGGAAACTTTGAGATTGCACCACCCATTG
CACTCTTTGTTCCGTAAAGTCATGAGAGATGTCGCTATTCCAAACACTTCTTACGTTGTCCCAAGGGACTACCACGTTCTAGTCTCCCCAGGTTACACTCACTTGCAAGAAGAATT
CTTCCCTAAGCCAAATGAATTCAACATCCACCGTTGGGACGGTGATGCTGCTTCTTCCAGTGCTGCTGGTGGTGACGAAGTTGATTACGGTTTCGGTGCTATCTCCAAGGGTGT
TTCCTCTCCATACTTGCCATTCGGTGGTGGTAGACACAGATGTATCGGTGAATTGTTCGCTTACTGTCAATTGGGTGTGTTGATGTCCATTTTCATCAGAACCATGAAATGGCGT
TACCCAACTGAAGGTGAAACTGTCCCACCATCTGACTTCACCTCCATGGTCACCCTACCAACTGCCCCAGCTAAGATCTACTGGGAAAAGAGACATCCAGAACAAAAGTACTAG
-

>10

ATGTCCACTGAAAACACTTCTTTGGTCGTTGAACTATTGGAGTACGTGAAGCTTGGTCTTTCGTACTTCCAAGCTCTGCCATTGGCGCAGAGAGTGTCTATTATGGTCGCCTTGC
CATTTGTGTACACCATCACATGGCAATTGCTTTACTCCTTGAGAAAGGACAGACCACCACTTGTGTTCTACTGGATCCCATGGGTCGGCTCTGCTATCCCATACGGTACCAAGCC
ATACGAGTTCTTCGAAGACTGCCAAAAGAAATACGGTGATATCTTCTCTTTCATGCTATTGGGTAGAATTATGACTGTCTACTTGGGTCCAAAGGGTCACGAATTCATCTTCAAC
GCCAAGTTGGCCGATGTTTCCGCTGAAGCTGCTTACTCCCACTTGACCACCCCAGTGTTCGGTAAAGGTGTTATCTACGATTGTCCAAACCACAGACTAATGGAACAAAAGAAG
TTTGTCAAGGGTGCTTTGACTAAGGAAGCCTTTGTCAGATACGTTCCATTGATCGCTGAGGAAATCTACAAGTACTTCAGAAACTCCAAGAACTTCAAGATCAACGAAAACAAC
TCCGGTATCGTCGACGTTATGGTCTCCCAACCTGAAATGACTATCTTCACTGCTTCCAGATCCTTGCTAGGTAAGGAAATGAGAGACAAGTTGGACACCGACTTTGCTTACTTGT
ACAGTGACTTGGACAAGGGTTTCACCCCAATTAACTTCGTCTTCCCTAACTTGCCTCTAGAACACTACAGAAAGAGAGACCATGCCCAACAAGCTATCTCTGGTACTTACATGTC
CTTGATTAAGGAAAGACGTGAGAAGAACGACATCCAAAACCGTGACTTGATTGATGAATTGATGAAGAACTCCACTTACAAGGATGGTACTAAGATGACCGACCAAGAAATT
GCCAACCTATTGATTGGTGTCTTGATGGGTGGTCAACATACTTCCGCTGCTACCTCCGCTTGGTGTCTATTGCATTTGGCTGAAAGACCAGATGTCCAAGAAGAATTGTACCAA
GAACAAATGCGCGTCTTGAACAACGATACCAAGGAATTGACTTACGATGACCTACAAAACATGCCTCTATTGAACCAAATGATCAAGGAAACTTTGAGATTGCACCACCCATTG
CACTCTTTGTTCCGTAAAGTCATGAGAGATGTCGCTATTCCAAACACTTCCTACGTTGTCCCAAGGGACTACCACGTTCTAGTCTCCCCAGGTTACACTCACTTGCAAGAAGAATT
CTTCCCTAAGCCAAACGAATTCAACATCCACCGTTGGGACGGTGATGCTGCTTCTTCCAGTGCTGCTGGTGGTGACGAAGTTGATTACGGTTTCGGTGCTATCTCCAAGGGTGT
TTCCTCTCCATACTTGCCATTCGGTGGTGGTAGACACAGATGTATCGGTGAATTGTTCGCTTACTGTCAATTGGGTGTGTTGATGTCCATTTTCATCAGAACCATGAAATGGCGT
TACCCAACTGAAGGTGAAACTGTCCCACCATCTGACTTCACCTCCATGGTCACCCTACCAACTGCCCCAGCTAAGATCTACTGGGAAAAGAGACATCCAGAACAAAAGTACTAG
-

>12

ATGTCCACTGAAAACACTTCTTTGGTCGTTGAACTATTGGAGTACGTGAAGCTTGGTCTTTCGTACTTCCAAGCTCTGCCATTGGCGCAGAGAGTGTCTATTATGGTCGCCTTGC
CATTTGTGTACACCATCACATGGCAATTGCTTTACTCCTTGAGAAAGGACAGACCACCACTTGTGTTCTACTGGATCCCATGGGTCGGCTCTGCTATCCCATACGGTACCAAGCC
ATACGAGTTCTTCGAAGACTGCCAAAAGAAATACGGTGATATCTTCTCTTTCATGCTATTGGGTAGAATTATGACTGTCTACTTGGGTCCAAAGGGTCACGAATTCATCTTCAAC
GCCAAGTTGGCCGATGTTTCCGCTGAAGCTGCTTACTCCCACTTGACCACCCCAGTGTTCGGTAAAGGTGTTATCTACGATTGTCCAAACCACAGACTAATGGAACAAAAGAAG
TTTGTCAAGGGTGCTTTGACTAAGGAAGCCTTTGTCAGATACGTTCCATTGATCGCTGAGGAAATCTACAAGTACTTCAGAAACTCCAAGAACTTCAAGATCAACGAAAACAAC
TCCGGTATCGTCGACGTTATGGTCTCCCAACCTGAAATGACTATCTTCACTGCTTCCAGATCCTTGCTAGGTAAGGAAATGAGAGACAAGTTGGACACCGACTTTGCTTACTTGT
ACAGTGACTTGGACAAGGGTTTCACCCCAATTAACTTCGTCTTCCCTAACTTGCCTCTAGAACACTACAGAAAGAGAGACCATGCCCAACAAGCTATCTCTGGTACTTACATGTC
CTTGATTAAGGAAAGACGTGAGAAGAACGACATCCAAAACCGTGACTTGATTGATGAATTGATGAAGAACTCCACTTACAAGGATGGTACTAAGATGACCGACCAAGAAATT
GCCAACCTATTGATTGGTGTCTTGATGGGTGGTCAACATACTTCCGCTGCTACCTCCGCTTGGTGTCTATTGCATTTGGCTGAAAGACCAGATGTCCAAGAAGAATTGTACCAA
GAACAAATGCGCGTCTTGAACAACGATACCAAGGAATTGACTTACGATGACCTACAAAACATGCCTCTATTGAACCAAATGATCAAGGAAACTTTGAGATTGCACCACCCATTG
CACTCTTTGTTCCGTAAAGTCATGAGAGATGTCGCTATTCCAAACACTTCCTACGTTGTCCCAAGGGACTACCACGTTCTAGTCTCCCCAGGTTACACTCACTTGCAAGAAGAATT
CTTCCCTAAGCCAAACGAATTCAACATCCACCGTTGGGACGGTGATGCTGCTTCTTCCAGTGCTGCTGGTGGTGACGAAGTTGATTACGGTTTCGGTGCTATCTCCAAGGGTGT
TTCCTCTCCATACTTGCCATTCGGTGGTGGTAGACACAGATGTATCGGTGAATTGTTCGCTTACTGTCAATTGGGTGTGTTGATGTCCATTTTCATCAGAACCATGAAATGGCGT

TACCCAACTGAAGGTGAAACTGTCCCACCATCTGACTTCACCTCCATGGTCACCCTACCAACTGCCCCAGCTAAGATCTACTGGGAAAAGAGACATCCAGAACAAAAGTACTAG
-

>13

ATGTCCACTGAAAACACTTCTTTGGTCGTTGAACTATTGGAGTACGTGAAGCTTGGTCTTTCGTACTTCCAAGCTCTGCCATTGGCGCAGAGAGTGTCTATTATGGTCGCCTTGC
CATTTGTGTACACCATCACATGGCAATTGCTTTACTCCTTGAGAAAGGACAGACCACCACTTGTGTTCTACTGGATCCCATGGGTCGGCTCTGCTATCCCATACGGTACCAAGCC
ATACGAGTTCTTCGAAGACTGCCAAAAGAAATACGGTGATATCTTCTCTTTCATGCTATTGGGTAGAATTATGACTGTCTACTTGGGTCCAAAGGGTCACGAATTCATCTTCAAC
GCCAAGTTGGCCGATGTTTCCGCTGAAGCTGCTTACTCCCACTTGACCACCCCAGTGTTCGGTAAAGGTGTTATCTACGATTGTCCAAACCACAGACTAATGGAACAAAAGAAG
TTTGTCAAGGGTGCTTTGACTAAGGAAGCCTTTGTCAGATACGTTCCATTGATCGCTGAGGAAATCTACAAGTACTTCAGAAACTCCAAGAACTTCAAGATCAACGAAAACAAC
TCCGGTATCGTCGACGTTATGGTCTCCCAACCTGAAATGACTATCTTCACTGCTTCCAGATCCTTGCTAGGTAAGGAAATGAGAGACAAGTTGGACACCGACTTCGCTTACTTGT
ACAGTGACTTGGACAAGGGTTTCACCCCAATTAACTTCGTCTTCCCTAACTTGCCTCTAGAACACTACAGAAAGAGAGACCATGCCCAACAAGCTATCTCTGGTACTTACATGTC
CTTGATTAAGGAAAGACGTGAGAAGAACGACATCCAAAACCGTGACTTGATTGATGAATTGATGAAGAACTCCACTTACAAGGATGGTACTAAGATGACCGACCAAGAAATT
GCCAACCTATTGATTGGTGTCTTGATGGGTGGTCAACATACTTCCGCTGCTACCTCCGCTTGGTGTCTATTGCATTTGGCTGAAAGACCAGATGTCCAAGAAGAATTGTACCAA
GAACAAATGCGCGTCTTGAACAACGATACCAAGGAATTGACTTACGATGACCTACAAAACATGCCTCTATTGAACCAAATGATCAAGGAAACTTTGAGATTGCACCACCCATTG
CACTCTTTGTTCCGTAAAGTCATGAGAGATGTCGCTATTCCAAACACTTCCTACGTTGTCCCAAGGGACTACCACGTTCTAGTCTCCCCAGGTTACACTCACTTGCAAGAAGAATT
CTTCCCTAAGCCAAATGAATTCAACATCCACCGTTGGGACGGTGATGCTGCTTCTTCCAGTGCTGCTGGTGGTGACGAAGTTGATTACGGTTTCGGTGCTATCTCCAAGGGTGT
TTCCTCTCCATACTTGCCATTCGGTGGTGGTAGACACAGATGTATCGGTGAATTGTTCGCTTACTGTCAATTGGGTGTGTTGATGTCCATTTTCATCAGAACCATGAAATGGCGT
TACCCAACTGAAGGTGAAACTGTCCCACCATCTGACTTCACCTCCATGGTCACCCTACCAACTGCCCCAGCTAAGATCTACTGGGAAAAGAGACATCCAGAACAAAAGTACTAG
-

>7

ATGTCCACTGAAAACACTTCTTTGGTCGTTGAACTATTGGAGTACGTGAAGCTTGGTCTTTCGTACTTCCAAGCTCTGCCATTGGCGCAGAGAGTGTCTATTATGGTCGCCTTGC
CATTTGTGTACACCATCACATGGCAATTGCTTTACTCCTTGAGAAAGGACAGACCACCACTTGTGTTCTACTGGATCCCATGGGTCGGCTCTGCTATCCCATACGGTACCAAGCC
ATACGAGTTCTTCGAAGACTGCCAAAAGAAATACGGTGATATCTTCTCTTTCATGCTATTGGGTAGAATTATGACTGTCTACTTGGGTCCAAAGGGTCACGAATTCATCTTCAAC
GCCAAGTTGGCCGATGTTTCCGCTGAAGCTGCTTACTCCCACTTGACCACCCCAGTGTTCGGTAAAGGTGTTATCTACGATTGTCCAAACCACAGACTAATGGAACAAAAGAAG
TTTGTCAAGGGTGCTTTGACTAAGGAAGCCTTTGTCAGATACGTTCCATTGATCGCTGAGGAAATCTACAAGTACTTCAGAAACTCCAAGAACTTCAAGATCAACGAAAACAAC
TCCGGTATCGTCGACGTTATGGTCTCCCAACCTGAAATGACTATCTTCACTGCTTCCAGATCCTTGCTAGGTAAGGAAATGAGAGACAAGTTGGACACCGACTTCGCTTACTTGT
ACAGTGACTTGGACAAGGGTTTCACCCCAATTAACTTCGTCTTCCCTAACTTGCCTCTAGAACACTACAGAAAGAGAGACCATGCCCAACAAGCTATCTCTGGTACTTACATGTC
CTTGATTAAGGAAAGACGTGAGAAGAACGACATCCAAAACCGTGACTTGATTGATGAATTGATGAAGAACTCCACTTACAAGGATGGTACTAAGATGACCGACCAAGAAATT
GCCAACCTATTGATTGGTGTCTTGATGGGTGGTCAACATACTTCCGCTGCTACCTCCGCTTGGTGTCTATTGCATTTGGCTGAAAGACCAGATGTCCAAGAAGAATTGTACCAA
GAACAAATGCGCGTCTTGAACAACGATACCAAGGAATTGACTTACGATGACCTACAAAACATGCCTCTATTGAACCAAATGATCAAGGAAACTTTGAGATTGCACCACCCATTG

CACTCTTTGTTCCGTAAAGTCATGAGAGATGTCGCTATTCCAAACACTTCCTACGTTGTCCCAAGGGACTACCACGTTCTAGTCTCCCCAGGTTACACTCACTTGCAAGAAGAATT
CTTCCCTAAGCCAAATGAATTCAACATCCACCGTTGGGACGGTGATGCTGCTTCTTCCAGTGCTGCTGGTGGTGACGAAGTTGATTACGGTTTCGGTGCTATCTCCAAGGGTGT
TTCCTCTCCATACTTGCCATTCGGTGGTGGTAGACACAGATGTATCGGTGAATTGTTCGCTTACTGTCAATTGGGTGTGTTGATGTCCATTTTCATCAGAACCATGAAATGGCGT
TACCCAACTGAAGGTGAAACTGTCCCACCATCTGACTTCACCTCCATGGTCACCCTACCAACTGCCCCAGCTAAGATCTACTGGGAAAAGAGACATCCAGAACAAAAGTACTAG
-

>11

ATGTCCACTGAAAACACTTCTTTGGTCGTTGAACTATTGGAGTACGTGAAGCTTGGTCTTTCGTACTTCCAAGCTCTGCCATTGGCACAGAGAGTGTCTATTATGGTCGCCTTGC
CATTTGTGTACACCATCACATGGCAATTGCTTTACTCCTTGAGAAAGGACAGACCACCACTTGTGTTCTACTGGATTCCATGGGTCGGCTCTGCTATCCCATACGGTACCAAGCC
ATACGAGTTCTTCGAAGACTGCCAAAAGAAATACGGTGATATCTTCTCTTTCATGCTATTGGGTAGAATTATGACTGTCTACTTGGGTCCAAAGGGTCACGAATTCATCTTCAAC
GCCAAGTTGGCCGATGTTTCCGCTGAAGCTGCTTACTCCCACTTGACCACCCCAGTGTTCGGTAAAGGTGTTATCTACGATTGTCCAAACCACAGACTAATGGAACAAAAGAAG
TTTGTCAAGGGTGCTTTGACTAAGGAAGCCTTTGTCAGATACGTTCCATTGATCGCTGAGGAAATCTACAAGTACTTCAGAAACTCCAAGAACTTCAAGATCAACGAAAACAAC
TCCGGTATCGTCGACGTTATGGTCTCCCAACCTGAAATGACTATCTTCACTGCTTCCAGATCCTTGCTAGGTAAGGAAATGAGAGACAAGTTGGACACCGACTTCGCTTACTTGT
ACAGTGACTTGGACAAGGGTTTCACCCCAATTAACTTCGTCTTCCCTAACTTGCCTCTAGAACACTACAGAAAGAGAGACCATGCCCAACAAGCTATCTCTGGTACTTACATGTC
CTTGATTAAGGAAAGACGTGAGAAGAACGACATCCAAAACCGTGACTTGATTGATGAATTGATGAAGAACTCCACTTACAAGGATGGTACTAAGATGACCGACCAAGAAATT
GCCAACCTATTGATTGGTGTCTTGATGGGTGGTCAACATACTTCCGCTGCTACCTCCGCTTGGTGTCTATTGCATTTGGCTGAAAGACCAGATGTCCAAGAAGAATTGTACCAA
GAACAAATGCGCGTCTTGAACAACGATACCAAGGAATTGACTTACGATGACCTACAAAACATGCCTCTATTGAACCAAATGATCAAGGAAACTTTGAGATTGCACCACCCATTG
CACTCTTTGTTCCGTAAAGTCATGAGAGATGTCGCTATTCCAAACACTTCCTACGTTGTCCCAAGGGACTACCACGTTCTAGTCTCCCCAGGTTACACTCACTTGCAAGAAGAATT
CTTCCCTAAGCCAAATGAATTCAACATCCACCGTTGGGACGGTGATGCTGCTTCTTCCAGTGCTGCTGGTGGTGACGAAGTTGATTACGGTTTCGGTGCTATCTCCAAGGGTGT
TTCCTCTCCATACTTGCCATTCGGTGGTGGTAGACACAGATGTATCGGTGAATTGTTCGCTTACTGTCAATTGGGTGTGTTGATGTCCATTTTCATCAGAACCATGAAATGGCGT
TACCCAACTGAAGGTGAAACTGTCCCACCATCTGACTTCACCTCCATGGTCACCCTACCAACTGCCCCAGCTAAGATCTACTGGGAAAAGAGACATCCAGAACAAAAGTACTAG
-

>15

ATGTCCACTGAAAACACTTCTTTGGTCGTTGAACTATTGGAGTACGTGAAGCTTGGTCTTTCGTACTTCCAAGCTCTGCCATTGGCGCAGAGAGTGTCTATTATGGTCGCCTTGC
CATTTGTGTACACCATCACATGGCAATTGCTTTACTCCTTGAGAAAGGACAGACCACCACTTGTGTTCTACTGGATCCCATGGGTCGGCTCTGCTATCCCATACGGTACCAAGCC
ATACGAGTTCTTCGAAGACTGCCAAAAGAAATACGGTGATATCTTCTCTTTCATGCTATTGGGTAGAATTATGACTGTCTACTTGGGTCCAAAGGGTCACGAATTCATCTTCAAC
GCCAAGTTGGCCGATGTTTCCGCTGAAGCTGCTTACTCCCACTTGACCACCCCAGTGTTCGGTAAAGGTGTTATCTACGATTGTCCAAACCACAGACTAATGGAACAAAAGAAG
TTTGTCAAGGGTGCTTTGACTAAGGAAGCCTTTGTCAGATACGTTCCATTGATCGCTGAGGAAATCTACAAGTACTTCAGAAACTCCAAGAACTTCAAGATCAACGAAAACAAC
TCCGGTATCGTCGACGTTATGGTCTCCCAACCTGAAATGACTATCTTCACTGCTTCCAGATCCTTGCTAGGTAAGGAAATGAGAGACAAGTTGGACACCGACTTCGCTTACTTGT
ACAGTGACTTGGACAAGGGTTTCACCCCAATTAACTTCGTCTTCCCTAACTTGCCTCTAGAACACTACAGAAAGAGAGACCATGCCCAACAAGCTATCTCTGGTACTTACATGTC

CTTGATTAAGGAAAGACGTGAGAAGAACGACATCCAAAACCGTGACTTGATTGATGAATTGATGAAGAACTCCACTTACAAGGATGGTACTAAGATGACCGACCAAGAAATT
GCCAACCTATTGATTGGTGTCTTGATGGGTGGTCAACATACTTCCGCTGCTACCTCCGCTTGGTGTCTATTGCATTTGGCTGAAAGACCAGATGTCCAAGAAGAATTGTACCAA
GAACAAATGCGCGTCTTGAACAACGATACCAAGGAATTGACTTACGATGACCTACAAAACATGCCTCTATTGAACCAAATGATCAAGGAAACTTTGAGATTGCACCACCCATTG
CACTCTTTGTTCCGTAAAGTCATGAGAGATGTCGCTATTCCAAACACTTCCTACGTTGTCCCAAGGGACTACCACGTTCTAGTCTCCCCAGGTTACACTCACTTGCAAGAAGAATT
CTTCCCTAAGCCAAATGAATTCAACATCCACCGTTGGGACGGTGATGCTGCTTCTTCCAGTGCTGCTGGTGGTGACGAAGTTGATTACGGTTTCGGTGCTATCTCCAAGGGTGT
TTCCTCTCCATACTTGCCATTCGGTGGTGGTAGACACAGATGTATCGGTGAATTGTTCGCTTACTGTCAATTGGGTGTGTTGATGTCCATTTTCATCAGAACCATGAAATGGCGT
TACCCAACTGAAGGTGAAACTGTCCCACCATCTGACTTCACCTCCATGGTCACCCTACCAACTGCCCCAGCTAAGATCTACTGGGAAAAGAGACATCCAGAACAAAAGTACTAG
-

>17

ATGTCCACTGAAAACACTTCTTTGGTCGTTGAACTATTGGAGTACGTGAAGCTTGGTCTTTCGTACTTCCAAGCTCTGCCATTGGCGCAGAGAGTGTCTATTATGGTCGCCTTGC
CATTTGTGTACACCATCACATGGCAATTGCTTTACTCCTTGAGAAAGGACAGACCACCACTTGTGTTCTACTGGATTCCATGGGTCGGCTCTGCTATCCCATACGGTACCAAGCC
ATACGAGTTCTTCGAAGACTGCCAAAAGAAATACGGTGATATCTTCTCTTTCATGCTATTGGGTAGAATTATGACTGTCTACTTGGGTCCAAAGGGTCACGAATTCATCTTCAAC
GCCAAGTTGGCCGATGTTTCCGCTGAAGCTGCTTACTCCCACTTGACCACCCCAGTGTTCGGTAAAGGTGTTATCTACGATTGTCCAAACCACAGACTAATGGAACAAAAGAAG
TTTGTCAAGGGTGCTTTGACTAAGGAAGCCTTTGTCAGATACGTTCCATTGATCGCTGAGGAAATCTACAAGTACTTCAGAAACTCCAAGAACTTCAAGATCAACGAAAACAAC
TCCGGTATCGTCGACGTTATGGTCTCCCAACCTGAAATGACTATCTTCACTGCTTCCAGATCCTTGCTAGGTAAGGAAATGAGAGACAAGTTGGACACCGACTTCGCTTACTTGT
ACAGTGACTTGGACAAGGGTTTCACCCCAATTAACTTCGTCTTCCCTAACTTGCCTCTAGAACACTACAGAAAGAGAGACCATGCCCAACAAGCTATCTCTGGTACTTACATGTC
CTTGATTAAGGAAAGACGTGAGAAGAACGATATCCAAAACCGTGATTTGATTGATGAATTGATGAAGAACTCCACTTACAAGGATGGTACTAAGATGACCGACCAAGAAATTG
CCAACCTATTGATTGGTGTCTTGATGGGTGGTCAACATACTTCCGCTGCTACCTCCGCTTGGTGTCTATTGCATTTGGCTGAAAGACCAGATGTCCAAGAAGAATTGTACCAAG
AACAAATGCGCGTCTTGAACAACGATACCAAGGAATTGACTTACGATGACCTACAAAACATGCCTCTATTGAACCAAATGATCAAGGAAACTTTGAGATTGCACCACCCATTGC
ACTCTTTGTTCCGTAAAGTCATGAGAGATGTCGCTATTCCAAACACTTCCTACGTTGTCCCAAGGGACTACCACGTTCTAGTCTCCCCAGGTTACACTCACTTGCAAGAAGAATTC
TTCCCTAAGCCAAACGAATTCAACATCCACCGTTGGGACGGTGATGCTGCTTCTTCCAGTGCTGCTGGTGGTGACGAAGTTGATTACGGTTTCGGTGCTATCTCCAAGGGTGTT
TCCTCTCCATACTTGCCATTCGGTGGTGGTAGACACAGATGTATCGGTGAATTGTTCGCTTACTGTCAATTGGGTGTGTTGATGTCCATTTTCATCAGAACCATGAAATGGCGTT
ACCCAACTGAAGGTGAAACTGTCCCACCATCTGACTTCACCTCCATGGTCACCCTACCAACTGCCCCAGCTAAGATCTACTGGGAAAAGAGACATCCAGAACAAAAGTACTAG-

>18

ATGTCCACTGAAAACACTTCTTTGGTCGTTGAACTATTGGAGTACGTGAAGCTTGGTCTTTCGTACTTCCAAGCTCTGCCATTGGCACAGAGAGTGTCTATTATGGTCGCCTTGC
CATTTGTGTACACCATCACATGGCAATTGCTTTACTCCTTGAGAAAGGACAGACCACCACTTGTGTTCTACTGGATTCCATGGGTCGGCTCTGCTATCCCATACGGTACCAAGCC
ATACGAGTTCTTCGAAGACTGCCAAAAGAAATACGGTGATATCTTCTCTTTCATGCTATTGGGTAGAATTATGACTGTCTACTTGGGTCCAAAGGGTCACGAATTCATCTTCAAC
GCCAAGTTGGCCGATGTTTCCGCTGAAGCTGCTTACTCCCACTTGACCACCCCAGTGTTCGGTAAAGGTGTTATCTACGATTGTCCAAACCACAGACTAATGGAACAAAAGAAG
TTTGTCAAGGGTGCTTTGACTAAGGAAGCCTTTGTCAGATACGTTCCATTGATCGCTGAGGAAATCTACAAGTACTTCAGAAACTCCAAGAACTTCAAGATCAACGAAAACAAC

TCCGGTATCGTCGACGTTATGGTCTCCCAACCTGAAATGACTATCTTCACTGCTTCCAGATCCTTGCTAGGTAAGGAAATGAGAGACAAGTTGGACACCGACTTCGCTTACTTGT
ACAGTGACTTGGACAAGGGTTTCACCCCAATTAACTTCGTCTTCCCTAACTTGCCTCTAGAACACTACAGAAAGAGAGACCATGCCCAACAAGCTATCTCTGGTACTTACATGTC
CTTGATTAAGGAAAGACGTGAGAAGAACGACATCCAAAACCGTGACTTGATTGATGAATTGATGAAGAACTCCACTTACAAGGATGGTACTAAGATGACCGACCAAGAAATT
GCCAACCTATTGATTGGTGTCTTGATGGGTGGTCAACATACTTCCGCTGCTACCTCCGCTTGGTGTCTATTGCATTTGGCTGAAAGACCAGATGTCCAAGAAGAATTGTACCAA
GAACAAATGCGCGTCTTGAACAACGATACCAAGGAATTGACTTACGATGACCTACAAAACATGCCTCTATTGAACCAAATGATCAAGGAAACTTTGAGATTGCACCACCCATTG
CACTCTTTGTTCCGTAAAGTCATGAGAGATGTCGCTATTCCAAACACTTCCTACGTTGTCCCAAGGGACTACCACGTTCTAGTCTCCCCAGGTTACACTCACTTGCAAGAAGAATT
CTTCCCTAAGCCAAATGAATTCAACATCCACCGTTGGGACGGTGATGCTGCTTCTTCCAGTGCTGCTGGTGGTGACGAAGTTGATTACGGTTTCGGTGCTATCTCCAAGGGTGT
TTCCTCTCCATACTTGCCATTCGGTGGTGGTAGACACAGATGTATCGGTGAATTGTTCGCTTACTGTCAATTGGGTGTGTTGATGTCCATTTTCATCAGAACCATGAAATGGCGT
TACCCAACTGAAGGTGAAACTGTCCCACCATCTGACTTCACCTCCATGGTCACCCTACCAACTGCCCCAGCTAAGATCTACTGGGAAAAGAGACATCCAGAACAAAAGTACTAG
-

>20

ATGTCCACTGAAAACACTTCTTTGGTCGTTGAACTATTGGAGTACGTGAAGCTTGGTCTTTCGTACTTCCAAGCTCTGCCATTGGCACAGAGAGTGTCTATTATGGTCGCCTTGC
CATTTGTGTACACCATCACATGGCAATTGCTTTACTCCTTGAGAAAGGACAGACCACCACTTGTGTTCTACTGGATTCCATGGGTCGGCTCTGCTATCCCATACGGTACCAAGCC
ATACGAGTTCTTCGAAGACTGCCAAAAGAAATACGGTGATATCTTCTCTTTCATGCTATTGGGTAGAATTATGACTGTCTACTTGGGTCCAAAGGGTCACGAATTCATCTTCAAC
GCCAAGTTGGCCGATGTTTCCGCTGAAGCTGCTTACTCCCACTTGACCACCCCAGTGTTCGGTAAAGGTGTTATCTACGATTGTCCAAACCACAGACTAATGGAACAAAAGAAG
TTTGTCAAGGGTGCTTTGACTAAGGAAGCCTTTGTCAGATACGTTCCATTGATCGCTGAGGAAATCTACAAGTACTTCAGAAACTCCAAGAACTTCAAGATCAACGAAAACAAC
TCCGGTATCGTCGACGTTATGGTCTCCCAACCTGAAATGACTATCTTCACTGCTTCCAGATCCTTGCTAGGTAAGGAAATGAGAGACAAGTTGGACACCGACTTCGCTTACTTGT
ACAGTGACTTGGACAAGGGTTTCACCCCAATTAACTTCGTCTTCCCTAACTTGCCTCTAGAACACTACAGAAAGAGAGACCATGCCCAACAAGCTATCTCTGGTACTTACATGTC
CTTGATTAAGGAAAGACGTGAGAAGAACGACATCCAAAACCGTGACTTGATTGATGAATTGATGAAGAACTCCACTTACAAGGATGGTACTAAGATGACCGACCAAGAAATT
GCCAACCTATTGATTGGTGTCTTGATGGGTGGTCAACATACTTCCGCTGCTACCTCCGCTTGGTGTCTATTGCATTTGGCTGAAAGACCAGATGTCCAAGAAGAATTGTACCAA
GAACAAATGCGCGTCTTGAACAACGATACCAAGGAATTGACTTACGATGACCTACAAAACATGCCTCTATTGAACCAAATGATCAAGGAAACTTTGAGATTGCACCACCCATTG
CACTCTTTGTTCCGTAAAGTCATGAGAGATGTCGCTATTCCAAACACTTCCTACGTTGTCCCAAGGGACTACCACGTTCTAGTCTCCCCAGGTTACACTCACTTGCAAGAAGAATT
CTTCCCTAAGCCAAATGAATTCAACATCCACCGTTGGGACGGTGATGCTGCTTCTTCCAGTGCTGCTGGTGGTGACGAAGTTGATTACGGTTTCGGTGCTATCTCCAAGGGTGT
TTCCTCTCCATACTTGCCATTCGGTGGTGGTAGACACAGATGTATCGGTGAATTGTTCGCTTACTGTCAATTGGGTGTGTTGATGTCCATTTTCATCAGAACCATGAAATGGCGT
TACCCAACTGAAGGTGAAACTGTCCCACCATCTGACTTCACCTCCATGGTCACCCTACCAACTGCCCCAGCTAAGATCTACTGGGAAAAGAGACATCCAGAACAAAAGTACTAG
-

>22

ATGTCCACTGAAAACACTTCTTTGGTCGTTGAACTATTGGAGTACGTGAAGCTTGGTCTTTCGTACTTCCAAGCTCTGCCATTGGCGCAGAGAGTGTCTATTATGGTCGCCTTGC
CATTTGTGTACACCATCACATGGCAATTGCTTTACTCCTTGAGAAAGGACAGACCACCACTTGTGTTCTACTGGATCCCATGGGTCGGCTCTGCTATCCCATACGGTACCAAGCC

ATACGAGTTCTTCGAAGACTGCCAAAAGAAATACGGTGATATCTTCTCTTTCATGCTATTGGGTAGAATTATGACTGTCTACTTGGGTCCAAAGGGTCACGAATTCATCTTCAAC
GCCAAGTTGGCCGATGTTTCCGCTGAAGCTGCTTACTCCCACTTGACCACCCCGGTGTTCGGTAAAGGTGTTATCTACGATTGTCCAAACCACAGACTAATGGAACAAAAGAAG
TTTGTCAAGGGTGCTTTGACTAAGGAAGCCTTTGTCAGATACGTTCCATTGATCGCTGAGGAAATCTACAAGTACTTCAGAAACTCCAAGAACTTCAAGATCAACGAAAACAAC
TCCGGTATCGTCGACGTTATGGTCTCCCAACCTGAAATGACTATCTTCACTGCTTCCAGATCCTTGCTAGGTAAGGAAATGAGAGACAAGTTGGACACCGACTTCGCTTACTTGT
ACAGTGACTTGGACAAGGGTTTCACCCCAATTAACTTCGTCTTCCCTAACTTGCCTCTAGAACACTACAGAAAGAGAGACCATGCCCAACAAGCTATCTCTGGTACTTACATGTC
CTTGATTAAGGAAAGACGTGAGAAGAACGATATCCAAAACCGTGACTTGATTGATGAATTGATGAAGAACTCCACTTACAAGGATGGTACTAAGATGACCGACCAAGAAATTG
CCAACCTATTGATTGGTGTCTTGATGGGTGGTCAACATACTTCCGCTGCTACCTCCGCTTGGTGTCTATTGCATTTGGCTGAAAGACCAGATGTCCAAGAAGAATTGTACCAAG
AACAAATGCGCGTCTTGAACAACGATACCAAGGAATTGACTTACGATGACCTACAAAACATGCCTCTATTGAACCAAATGATCAAGGAAACTTTGAGATTGCACCACCCATTGC
ACTCTTTGTTCCGTAAAGTCATGAGAGATGTCGCTATTCCAAACACTTCCTACGTTGTCCCAAGGGACTACCACGTTCTAGTCTCCCCAGGTTACACTCACTTGCAAGAAGAATTC
TTCCCTAAGCCAAACGAATTCAACATCCACCGTTGGGACGGTGATGCTGCTTCTTCCAGTGCTGCTGGTGGTGACGAAGTTGATTACGGTTTCGGTGCTATCTCCAAGGGTGTT
TCCTCTCCATACTTGCCATTCGGTGGTGGTAGACACAGATGTATCGGTGAATTGTTCGCTTACTGTCAATTGGGTGTGTTGATGTCCATTTTCATCAGAACCATGAAATGGCGTT
ACCCAACTGAAGGTGAAACTGTCCCACCATCTGACTTCACCTCCATGGTCACCCTACCAACTGCCCCAGCTAAGATCTACTGGGAAAAGAGACATCCAGAACAAAAGTACTAG-

>23

ATGTCCACTGAAAACACTTCTTTGGTCGTTGAACTATTGGAGTACGTGAAGCTTGGTCTTTCGTACTTCCAAGCTCTGCCATTGGCGCAGAGAGTGTCTATTATGGTCGCCTTGC
CATTTGTGTACACCATCACATGGCAATTGCTTTACTCCTTGAGAAAGGACAGACCACCACTTGTGTTCTACTGGATCCCATGGGTCGGCTCTGCTATCCCATACGGTACCAAGCC
ATACGAGTTCTTCGAAGACTGCCAAAAGAAATACGGTGATATCTTCTCTTTCATGCTATTGGGTAGAATTATGACTGTCTACTTGGGTCCAAAGGGTCACGAATTCATCTTCAAC
GCCAAGTTGGCCGATGTTTCCGCTGAAGCTGCTTACTCCCACTTGACCACCCCGGTGTTCGGTAAAGGTGTTATCTACGATTGTCCAAACCACAGACTAATGGAACAAAAGAAG
TTTGTCAAGGGTGCTTTGACTAAGGAAGCCTTTGTCAGATACGTTCCATTGATCGCTGAGGAAATCTACAAGTACTTCAGAAACTCCAAGAACTTCAAGATCAACGAAAACAAC
TCCGGTATCGTCGACGTTATGGTCTCCCAACCTGAAATGACTATCTTCACTGCTTCCAGATCCTTGCTAGGTAAGGAAATGAGAGACAAGTTGGACACCGACTTCGCTTACTTGT
ACAGTGACTTGGACAAGGGTTTCACCCCAATTAACTTCGTCTTCCCTAACTTGCCTCTAGAACACTACAGAAAGAGAGACCATGCCCAACAAGCTATCTCTGGTACTTACATGTC
CTTGATTAAGGAAAGACGTGAGAAGAACGATATCCAAAACCGTGACTTGATTGATGAATTGATGAAGAACTCCACTTACAAGGATGGTACTAAGATGACCGACCAAGAAATTG
CCAACCTATTGATTGGTGTCTTGATGGGTGGTCAACATACTTCCGCTGCTACCTCCGCTTGGTGTCTATTGCATTTGGCTGAAAGACCAGATGTCCAAGAAGAATTGTACCAAG
AACAAATGCGCGTCTTGAACAACGATACCAAGGAATTGACTTACGATGACCTACAAAACATGCCTCTATTGAACCAAATGATCAAGGAAACTTTGAGATTGCACCACCCATTGC
ACTCTTTGTTCCGTAAAGTCATGAGAGATGTCGCTATTCCAAACACTTCCTACGTTGTCCCAAGGGACTACCACGTTCTAGTCTCCCCAGGTTACACTCACTTGCAAGAAGAATTC
TTCCCTAAGCCAAACGAATTCAACATCCACCGTTGGGACGGTGATGCTGCTTCTTCCAGTGCTGCTGGTGGTGACGAAGTTGATTACGGTTTCGGTGCTATCTCCAAGGGTGTT
TCCTCTCCATACTTGCCATTCGGTGGTGGTAGACACAGATGTATCGGTGAATTGTTCGCTTACTGTCAATTGGGTGTGTTGATGTCCATTTTCATCAGAACCATGAAATGGCGTT
ACCCAACTGAAGGTGAAACTGTCCCACCATCTGACTTCACCTCCATGGTCACCCTACCAACTGCCCCAGCTAAGATCTACTGGGAAAAGAGACATCCAGAACAAAAGTACTAG-

>24

ATGTCCACTGAAAACACTTCTTTGGTCGTTGAACTATTGGAGTACGTGAAGCTTGGTCTTTCGTACTTCCAAGCTCTGCCATTGGCGCAGAGAGTGTCTATTATGGTCGCCTTGC
CATTTGTGTACACCATCACATGGCAATTGCTTTACTCCTTGAGAAAGGACAGACCACCACTTGTGTTCTACTGGATCCCATGGGTCGGCTCTGCTATCCCATACGGTACCAAGCC
ATACGAGTTCTTCGAAGACTGCCAAAAGAAATACGGTGATATCTTCTCTTTCATGCTATTGGGTAGAATTATGACTGTCTACTTGGGTCCAAAGGGTCACGAATTCATCTTCAAC
GCCAAGTTGGCCGATGTTTCCGCTGAAGCTGCTTACTCCCACTTGACCACCCCAGTGTTCGGTAAAGGTGTTATCTACGATTGTCCAAACCACAGACTAATGGAACAAAAGAAG
TTTGTCAAGGGTGCTTTGACTAAGGAAGCCTTTGTCAGATACGTTCCATTGATCGCTGAGGAAATCTACAAGTACTTCAGAAACTCCAAGAACTTCAAGATCAACGAAAACAAC
TCCGGTATCGTCGACGTTATGGTCTCCCAACCTGAAATGACTATCTTCACTGCTTCCAGATCCTTGCTAGGTAAGGAAATGAGAGACAAGTTGGACACCGACTTTGCTTACTTGT
ACAGTGACTTGGACAAGGGTTTCACCCCAATTAACTTCGTCTTCCCTAACTTGCCTCTAGAACACTACAGAAAGAGAGACCATGCCCAACAAGCTATCTCTGGTACTTACATGTC
CTTGATTAAGGAAAGACGTGAGAAGAACGACATCCAAAACCGTGACTTGATTGATGAATTGATGAAGAACTCCACTTACAAGGATGGTACTAAGATGACCGACCAAGAAATT
GCCAACCTATTGATTGGTGTCTTGATGGGTGGTCAACATACTTCCGCTGCTACCTCCGCTTGGTGTCTATTGCATTTGGCTGAAAGACCAGATGTCCAAGAAGAATTGTACCAA
GAACAAATGCGCGTCTTGAACAACGATACCAAGGAATTGACTTACGATGACCTACAAAACATGCCTCTATTGAACCAAATGATCAAGGAAACTTTGAGATTGCACCACCCATTG
CACTCTTTGTTCCGTAAAGTCATGAGAGATGTCGCTATTCCAAACACTTCCTACGTTGTCCCAAGGGACTACCACGTTCTAGTCTCCCCAGGTTACACTCACTTGCAAGAAGAATT
CTTCCCTAAGCCAAACGAATTCAACATCCACCGTTGGGACGGTGATGCTGCTTCTTCCAGTGCTGCTGGTGGTGACGAAGTTGATTACGGTTTCGGTGCTATCTCCAAGGGTGT
TTCCTCTCCATACTTGCCATTCGGTGGTGGTAGACACAGATGTATCGGTGAATTGTTCGCTTACTGTCAATTGGGTGTGTTGATGTCCATTTTCATCAGAACCATGAAATGGCGT
TACCCAACTGAAGGTGAAACTGTCCCACCATCTGACTTCACCTCCATGGTCACCCTACCAACTGCCCCAGCTAAGATCTACTGGGAAAAGAGACATCCAGAACAAAAGTACTAG
-

>25

ATGTCCACTGAAAACACTTCTTTGGTCGTTGAACTATTGGAGTACGTGAAGCTTGGTCTTTCGTACTTCCAAGCTCTGCCATTGGCGCAGAGAGTGTCTATTATGGTCGCCTTGC
CATTTGTGTACACCATCACATGGCAATTGCTTTACTCCTTGAGAAAGGACAGACCACCACTTGTGTTCTACTGGATCCCATGGGTCGGCTCTGCTATCCCATACGGTACCAAGCC
ATACGAGTTCTTCGAAGACTGCCAAAAGAAATACGGTGATATCTTCTCTTTCATGCTATTGGGTAGAATTATGACTGTCTACTTGGGTCCAAAGGGTCACGAATTCATCTTCAAC
GCCAAGTTGGCCGATGTTTCCGCTGAAGCTGCTTACTCCCACTTGACCACCCCAGTGTTCGGTAAAGGTGTTATCTACGATTGTCCAAACCACAGACTAATGGAACAAAAGAAG
TTTGTCAAGGGTGCTTTGACTAAGGAAGCCTTTGTCAGATACGTTCCATTGATCGCTGAGGAAATCTACAAGTACTTCAGAAACTCCAAGAACTTCAAGATCAACGAAAACAAC
TCCGGTATCGTCGACGTTATGGTCTCCCAACCTGAAATGACTATCTTCACTGCTTCCAGATCCTTGCTAGGTAAGGAAATGAGAGACAAGTTGGACACCGACTTTGCTTACTTGT
ACAGTGACTTGGACAAGGGTTTCACCCCAATTAACTTCGTCTTCCCTAACTTGCCTCTAGAACACTACAGAAAGAGAGACCATGCCCAACAAGCTATCTCTGGTACTTACATGTC
CTTGATTAAGGAAAGACGTGAGAAGAACGACATCCAAAACCGTGACTTGATTGATGAATTGATGAAGAACTCCACTTACAAGGATGGTACTAAGATGACCGACCAAGAAATT
GCCAACCTATTGATTGGTGTCTTGATGGGTGGTCAACATACTTCCGCTGCTACCTCCGCTTGGTGTCTATTGCATTTGGCTGAAAGACCAGATGTCCAAGAAGAATTGTACCAA
GAACAAATGCGCGTCTTGAACAACGATACCAAGGAATTGACTTACGATGACCTACAAAACATGCCTCTATTGAACCAAATGATCAAGGAAACTTTGAGATTGCACCACCCATTG
CACTCTTTGTTCCGTAAAGTCATGAGAGATGTCGCTATTCCAAACACTTCCTACGTTGTCCCAAGGGACTACCACGTTCTAGTCTCCCCAGGTTACACTCACTTGCAAGAAGAATT
CTTCCCTAAGCCAAACGAATTCAACATCCACCGTTGGGACGGTGATGCTGCTTCTTCCAGTGCTGCTGGTGGTGACGAAGTTGATTACGGTTTCGGTGCTATCTCCAAGGGTGT
TTCCTCTCCATACTTGCCATTCGGTGGTGGTAGACACAGATGTATCGGTGAATTGTTCGCTTACTGTCAATTGGGTGTGTTGATGTCCATTTTCATCAGAACCATGAAATGGCGT

TACCCAACTGAAGGTGAAACTGTCCCACCATCTGACTTCACCTCCATGGTCACCCTACCAACTGCCCCAGCTAAGATCTACTGGGAAAAGAGACATCCAGAACAAAAGTACTAG
-

>27

ATGTCCACTGAAAACACTTCTTTGGTCGTTGAACTATTGGAGTACGTGAAGCTTGGTCTTTCGTACTTCCAAGCTCTGCCATTGGCGCAGAGAGTGTCTATTATGGTCGCCTTGC
CATTTGTGTACACCATCACATGGCAATTGCTTTACTCCTTGAGAAAGGACAGACCACCACTTGTGTTCTACTGGATCCCATGGGTCGGCTCTGCTATCCCATACGGTACCAAGCC
ATACGAGTTCTTCGAAGACTGCCAAAAGAAATACGGTGATATCTTCTCTTTCATGCTATTGGGTAGAATTATGACTGTCTACTTGGGTCCAAAGGGTCACGAATTCATCTTCAAC
GCCAAGTTGGCCGATGTTTCCGCTGAAGCTGCTTACTCCCACTTGACCACCCCAGTGTTCGGTAAAGGTGTTATCTACGATTGTCCAAACCACAGACTAATGGAACAAAAGAAG
TTTGTCAAGGGTGCTTTGACTAAGGAAGCCTTTGTCAGATACGTTCCATTGATCGCTGAGGAAATCTACAAGTACTTCAGAAACTCCAAGAACTTCAAGATCAACGAAAACAAC
TCCGGTATCGTCGACGTTATGGTCTCCCAACCTGAAATGACTATCTTCACTGCTTCCAGATCCTTGCTAGGTAAGGAAATGAGAGACAAGTTGGACACCGACTTCGCTTACTTGT
ACAGTGACTTGGACAAGGGTTTCACCCCAATTAACTTCGTCTTCCCTAACTTGCCTCTAGAACACTACAGAAAGAGAGACCATGCCCAACAAGCTATCTCTGGTACTTACATGTC
CTTGATTAAGGAAAGACGTGAGAAGAACGACATCCAAAACCGTGACTTGATTGATGAATTGATGAAGAACTCCACTTACAAGGATGGTACTAAGATGACCGACCAAGAAATT
GCCAACCTATTGATTGGTGTCTTGATGGGTGGTCAACATACTTCCGCTGCTACCTCCGCTTGGTGTCTATTGCATTTGGCTGAAAGACCAGATGTCCAAGAAGAATTGTACCAA
GAACAAATGCGCGTCTTGAACAACGATACCAAGGAATTGACTTACGATGACCTACAAAACATGCCTCTATTGAACCAAATGATCAAGGAAACTTTGAGATTGCACCACCCATTG
CACTCTTTGTTCCGTAAAGTCATGAGAGATGTCGCTATTCCAAACACTTCCTACGTTGTCCCAAGGGACTACCACGTTCTAGTCTCCCCAGGTTACACTCACTTGCAAGAAGAATT
CTTCCCTAAGCCAAATGAATTCAACATCCACCGTTGGGACGGTGATGCTGCTTCTTCCAGTGCTGCTGGTGGTGACGAAGTTGATTACGGTTTCGGTGCTATCTCCAAGGGTGT
TTCCTCTCCATACTTGCCATTCGGTGGTGGTAGACACAGATGTATCGGTGAATTGTTCGCTTACTGTCAATTGGGTGTGTTGATGTCCATTTTCATCAGAACCATGAAATGGCGT
TACCCAACTGAAGGTGAAACTGTCCCACCATCTGACTTCACCTCCATGGTCACCCTACCAACTGCCCCAGCTAAGATCTACTGGGAAAAGAGACATCCAGAACAAAAGTACTAG
-

>4

ATGTCCACTGAAAACACTTCTTTGGTCGTTGAACTATTGGAGTACGTGAAGCTTGGTCTTTCGTACTTCCAAGCTCTGCCATTGGCGCAGAGAGTGTCTATTATGGTCGCCTTGC
CATTTGTGTACACCATCACATGGCAATTGCTTTACTCCTTGAGAAAGGACAGACCACCACTTGTGTTCTACTGGATCCCATGGGTCGGCTCTGCTATCCCATACGGTACCAAGCC
ATACGAGTTCTTCGAAGACTGCCAAAAGAAATACGGTGATATCTTCTCTTTCATGCTATTGGGTAGAATTATGACTGTCTACTTGGGTCCAAAGGGTCACGAATTCATCTTCAAC
GCCAAGTTGGCCGATGTTTCCGCTGAAGCTGCTTACTCCCACTTGACCACCCCAGTGTTCGGTAAAGGTGTTATCTACGATTGTCCAAACCACAGACTAATGGAACAAAAGAAG
TTTGTCAAGGGTGCTTTGACTAAGGAAGCCTTTGTCAGATACGTTCCATTGATCGCTGAGGAAATCTACAAGTACTTCAGAAACTCCAAGAACTTCAAGATCAACGAAAACAAC
TCCGGTATCGTCGACGTTATGGTCTCCCAACCTGAAATGACTATCTTCACTGCTTCCAGATCCTTGCTAGGTAAGGAAATGAGAGACAAGTTGGACACCGACTTTGCTTACTTGT
ACAGTGACTTGGACAAGGGTTTCACCCCAATTAACTTCGTCTTCCCTAACTTGCCTCTAGAACACTACAGAAAGAGAGACCGTGCCCAACAAGCTATCTCTGGTACTTACATGTC
CTTGATTAAGGAAAGACGTGAGAAGAACGACATCCAAAACCGTGACTTGATTGATGAATTGATGAAGAACTCCACTTACAAGGATGGTACTAAGATGACCGACCAAGAAATT
GCCAACCTATTGATTGGTGTCTTGATGGGTGGTCAACATACTTCCGCTGCTACCTCCGCTTGGTGTCTATTGCATTTGGCTGAAAGACCAGATGTCCAAGAAGAATTGTACCAA
GAACAAATGCGCGTCTTGAACAACGATACCAAGGAATTGACTTACGATGACCTACAAAACATGCCTCTATTGAACCAAATGATCAAGGAAACTTTGAGATTGCACCACCCATTG

CACTCTTTGTTCCGTAAAGTCATGAGAGATGTCGCTATTCCAAACACTTCCTACGTTGTCCCAAGGGACTACCACGTTCTAGTCTCCCCAGGTTACACTCACTTGCAAGAAGAATT
CTTCCCTAAGCCAAACGAATTCAACATCCACCGTTGGGACGGTGATGCTGCTTCTTCCAGTGCTGCTGGTGGTGACGAAGTTGATTACGGTTTCGGTGCTATCTCCAAGGGTGT
TTCCTCTCCATACTTGCCATTCGGTGGTGGTAGACACAGATGTATCGGTGAATTGTTCGCTTACTGTCAATTGGGTGTGTTGATGTCCATTTTCATCAGAACCATGAAATGGCGT
TACCCAACTGAAGGTGAAACTGTCCCACCATCTGACTTCACCTCCATGGTCACCCTACCAACTGCCCCAGCTAAGATCTACTGGGAAAAGAGACATCCAGAACAAAAGTACTAG
-

>5

ATGTCCACTGAAAACACTTCTTTGGTCGTTGAACTATTGGAGTACGTGAAGCTTGGTCTTTCGTACTTCCAAGCTCTGCCATTGGCGCAGAGAGTGTCTATTATGGTCGCCTTGC
CATTTGTGTACACCATCACATGGCAATTGCTTTACTCCTTGAGAAAGGACAGACCACCACTTGTGTTCTACTGGATCCCATGGGTCGGCTCTGCTATCCCATACGGTACCAAGCC
ATACGAGTTCTTCGAAGACTGCCAAAAGAAATACGGTGATATCTTCTCTTTCATGCTATTGGGTAGAATTATGACTGTCTACTTGGGTCCAAAGGGTCACGAATTCATCTTCAAC
GCCAAGTTGGCCGATGTTTCCGCTGAAGCTGCTTACTCCCACTTGACCACCCCAGTGTTCGGTAAAGGTGTTATCTATGATTGTCCAAACCACAGACTAATGGAACAAAAGAAG
TTTGTCAAGGGTGCTTTGACTAAGGAAGCCTTTGTCAGATACGTTCCATTGATCGCTGAGGAAATCTACAAGTACTTCAGAAACTCCAAGAACTTCAAGATCAACGAAAACAAC
TCCGGTATCGTCGACGTTATGGTCTCCCAACCTGAAATGACTATCTTCACTGCTTCCAGATCCTTGCTAGGTAAGGAAATGAGAGACAAGTTGGACACCGACTTCGCTTACTTGT
ACAGTGACTTGGACAAGGGTTTCACCCCAATTAACTTCGTCTTCCCTAACTTGCCTCTAGAACACTACAGAAAGAGAGACCATGCCCAACAAGCTATCTCTGGTACTTACATGTC
CTTGATTAAGGAAAGACGTGAGAAGAACGATATCCAAAACCGTGACTTGATTGATGAATTGATGAAGAACTCCACTTACAAGGATGGTACTAAGATGACCGACCAAGAAATTG
CCAACCTATTGATTGGTGTCTTGATGGGTGGTCAACATACTTCCGCTGCTACCTCCGCTTGGTGTCTATTGCATTTGGCTGAAAGACCAGATGTCCAAGAAGAATTGTACCAAG
AACAAATGCGCGTCTTGAACAACGATACCAAGGAATTGACTTACGATGACCTACAAAACATGCCTCTATTGAACCAAATGATCAAGGAAACTTTGAGATTGCACCACCCATTGC
ACTCTTTGTTCCGTAAAGTCATGAGAGATGTCGCTATTCCAAACACTTCCTACGTTGTCCCAAGGGACTACCACGTTCTAGTCTCCCCAGGTTACACTCACTTGCAAGAAGAATTC
TTCCCTAAGCCAAACGAATTCAACATCCACCGTTGGGACGGTGATGCTGCTTCTTCCAGTGCTGCTGGTGGTGACGAAGTTGATTACGGTTTCGGTGCTATCTCCAAGGGTGTT
TCCTCTCCATACTTGCCATTCGGTGGTGGTAGACACAGATGTATCGGTGAATTGTTCGCTTACTGTCAATTGGGTGTGTTGATGTCCATTTTCATCAGAACCATGAAATGGCGTT
ACCCAACTGAAGGTGAAACTGTCCCACCATCTGACTTCACCTCCATGGTCACCCTACCAACTGCCCCAGCTAAGATCTACTGGGAAAAGAGACATCCAGAACAAAAGTACTAG-

>14

ATGTCCACTGAAAACACTTCTTTGGTCGTTGAACTATTGGAGTACGTGAAGCTTGGTCTTTCGTACTTCCAAGCTCTGCCATTGGCACAGAGAGTGTCTATTATGGTCGCCTTGC
CATTTGTGTACACCATCACATGGCAATTGCTTTACTCCTTGAGAAAGGACAGACCACCACTTGTGTTCTACTGGATTCCATGGGTCGGCTCTGCTATCCCATACGGTACCAAGCC
ATACGAGTTCTTCGAAGACTGCCAAAAGAAATACGGTGATATCTTCTCTTTCATGCTATTGGGTAGAATTATGACTGTCTACTTGGGTCCAAAGGGTCACGAATTCATCTTCAAC
GCCAAGTTGGCCGATGTTTCCGCTGAAGCTGCTTACTCCCACTTGACCACCCCAGTGTTCGGTAAAGGTGTTATCTACGATTGTCCAAACCACAGACTAATGGAACAAAAGAAG
TTTGTCAAGGGTGCTTTGACTAAGGAAGCCTTTGTCAGATACGTTCCATTGATCGCTGAGGAAATCTACAAGTACTTCAGAAACTCCAAGAACTTCAAGATCAACGAAAACAAC
TCCGGTATCGTCGACGTTATGGTCTCCCAACCTGAAATGACTATCTTCACTGCTTCCAGATCCTTGCTAGGTAAGGAAATGAGAGACAAGTTGGACACCGACTTCGCTTACTTGT
ACAGTGACTTGGACAAGGGTTTCACCCCAATTAACTTCGTCTTCCCTAACTTGCCTCTAGAACACTACAGAAAGAGAGACCATGCCCAACAAGCTATCTCTGGTACTTACATGTC
CTTGATTAAGGAAAGACGTGAGAAGAACGACATCCAAAACCGTGACTTGATTGATGAATTGATGAAGAACTCCACTTACAAGGATGGTACTAAGATGACCGACCAAGAAATT

GCCAACCTATTGATTGGTGTCTTGATGGGTGGTCAACATACTTCCGCTGCTACCTCCGCTTGGTGTCTATTGCATTTGGCTGAAAGACCAGATGTCCAAGAAGAATTGTACCAA
GAACAAATGCGCGTCTTGAACAACGATACCAAGGAATTGACTTACGATGACCTACAAAACATGCCTCTATTGAACCAAATGATCAAGGAAACTTTGAGATTGCACCACCCATTG
CACTCTTTGTTCCGTAAAGTCATGAGAGATGTCGCTATTCCAAACACTTCCTACGTTGTCCCAAGGGACTACCACGTTCTAGTCTCCCCAGGTTACACTCACTTGCAAGAAGAATT
CTTCCCTAAGCCAAATGAATTCAACATCCACCGTTGGGACGGTGATGCTGCTTCTTCCAGTGCTGCTGGTGGTGACGAAGTTGATTACGGTTTCGGTGCTATCTCCAAGGGTGT
TTCCTCTCCATACTTGCCATTCGGTGGTGGTAGACACAGATGTATCGGTGAATTGTTCGCTTACTGTCAATTGGGTGTGTTGATGTCCATTTTCATCAGAACCATGAAATGGCGT
TACCCAACTGAAGGTGAAACTGTCCCACCATCTGACTTCACCTCCATGGTCACCCTACCAACTGCCCCAGCTAAGATCTACTGGGAAAAGAGACATCCAGAACAAAAGTACTAG
-

>16

ATGTCCACTGAAAACACTTCTTTGGTCGTTGAACTATTGGAGTACGTGAAGCTTGGTCTTTCGTACTTCCAAGCTCTGCCATTGGCGCAGAGAGTGTCTATTATGGTCGCCTTGC
CATTTGTGTACACCATCACATGGCAATTGCTTTACTCCTTGAGAAAGGACAGACCACCACTTGTGTTCTACTGGATCCCATGGGTCGGCTCTGCTATCCCATACGGTACCAAGCC
ATACGAGTTCTTCGAAGACTGCCAAAAGAAATACGGTGATATCTTCTCTTTCATGCTATTGGGTAGAATTATGACTGTCTACTTGGGTCCAAAGGGTCACGAATTCATCTTCAAC
GCCAAGTTGGCCGATGTTTCCGCTGAAGCTGCTTACTCCCACTTGACCACCCCAGTGTTCGGTAAAGGTGTTATCTATGATTGTCCAAACCACAGACTAATGGAACAAAAGAAG
TTTGTCAAGGGTGCTTTGACTAAGGAAGCCTTTGTCAGATACGTTCCATTGATCGCTGAGGAAATCTACAAGTACTTCAGAAACTCCAAGAACTTCAAGATCAACGAAAACAAC
TCCGGTATCGTCGACGTTATGGTCTCCCAACCTGAAATGACTATCTTCACTGCTTCCAGATCCTTGCTAGGTAAGGAAATGAGAGACAAGTTGGACACCGACTTCGCTTACTTGT
ACAGTGACTTGGACAAGGGTTTCACCCCAATTAACTTCGTCTTCCCTAACTTGCCTCTAGAACACTACAGAAAGAGAGACCATGCCCAACAAGCTATCTCTGGTACTTACATGTC
CTTGATTAAGGAAAGACGTGAGAAGAACGATATCCAAAACCGTGACTTGATTGATGAATTGATGAAGAACTCCACTTACAAGGATGGTACTAAGATGACCGACCAAGAAATTG
CCAACCTATTGATTGGTGTCTTGATGGGTGGTCAACATACTTCCGCTGCTACCTCCGCTTGGTGTCTATTGCATTTGGCTGAAAGACCAGATGTCCAAGAAGAATTGTACCAAG
AACAAATGCGCGTCTTGAACAACGATACCAAGGAATTGACTTACGATGACCTACAAAACATGCCTCTATTGAACCAAATGATCAAGGAAACTTTGAGATTGCACCACCCATTGC
ACTCTTTGTTCCGTAAAGTCATGAGAGATGTCGCTATTCCAAACACTTCCTACGTTGTCCCAAGGGACTACCACGTTCTAGTCTCCCCAGGTTACACTCACTTGCAAGAAGAATTC
TTCCCTAAGCCAAACGAATTCAACATCCACCGTTGGGACGGTGATGCTGCTTCTTCCAGTGCTGCTGGTGGTGACGAAGTTGATTACGGTTTCGGTGCTATCTCCAAGGGTGTT
TCCTCTCCATACTTGCCATTCGGTGGTGGTAGACACAGATGTATCGGTGAATTGTTCGCTTACTGTCAATTGGGTGTGTTGATGTCCATTTTCATCAGAACCATGAAATGGCGTT
ACCCAACTGAAGGTGAAACTGTCCCACCATCTGACTTCACCTCCATGGTCACCCTACCAACTGCCCCAGCTAAGATCTACTGGGAAAAGAGACATCCAGAACAAAAGTACTAG-

>21

ATGTCCACTGAAAACACTTCTTTGGTCGTTGAACTATTGGAGTACGTGAAGCTTGGTCTTTCGTACTTCCAAGCTCTGCCATTGGCGCAGAGAGTGTCTATTATGGTCGCCTTGC
CATTTGTGTACACCATCACATGGCAATTGCTTTACTCCTTGAGAAAGGACAGACCACCACTTGTGTTCTACTGGATCCCATGGGTCGGCTCTGCTATCCCATACGGTACCAAGCC
ATACGAGTTCTTCGAAGACTGCCAAAAGAAATACGGTGATATCTTCTCTTTCATGCTATTGGGTAGAATTATGACTGTCTACTTGGGTCCAAAGGGTCACGAATTCATCTTCAAC
GCCAAGTTGGCCGATGTTTCCGCTGAAGCTGCTTACTCCCACTTGACCACCCCAGTGTTCGGTAAAGGTGTTATCTACGATTGTCCAAACCACAGACTAATGGAACAAAAGAAG
TTTGTCAAGGGTGCTTTGACTAAGGAAGCCTTTGTCAGATACGTTCCATTGATCGCTGAGGAAATCTACAAGTACTTCAGAAACTCCAAGAACTTCAAGATCAACGAAAACAAC
TCCGGTATCGTCAACGTTATGGTCTCCCAACCTGAAATGACTATCTTCACTGCTTCCAGATCCTTGCTAGGTAAGGAAATGAGAGACAAGTTGGACACCGACTTCGCTTACTTGT

ACAGTGACTTGGACAAGGGTTTCACCCCAATTAACTTCGTCTTCCCTAACTTGCCTCTAGAACACTACAGAAAGAGAGACCATGCCCAACAAGCTATCTCTGGTACTTACATGTC
CTTGATTAAGGAAAGACGTGAGAAGAACGACATCCAAAACCGTGACTTGATTGATGAATTGATGAAGAACTCCACTTACAAGGATGGTACTAAGATGACCGACCAAGAAATT
GCCAACCTATTGATTGGTGTCTTGATGGGTGGTCAACATACTTCCGCTGCTACCTCCGCTTGGTGTCTATTGCATTTGGCTGAAAGACCAGATGTCCAAGAAGAATTGTACCAA
GAACAAATGCGCGTCTTGAACAACGATACCAAGGAATTGACTTACGATGACCTACAAAACATGCCTCTATTGAACCAAATGATCAAGGAAACTTTGAGATTGCACCACCCATTG
CACTCTTTGTTCCGTAAAGTCATGAGAGATGTCGCTATTCCAAACACTTCCTACGTTGTCCCAAGGGACTACCACGTTCTAGTCTCCCCAGGTTACACTCACTTGCAAGAAGAATT
CTTCCCTAAGCCAAATGAATTCAACATCCACCGTTGGGACGGTGATGCTGCTTCTTCCAGTGCTGCTGGTGGTGACGAAGTTGATTACGGTTTCGGTGCTATCTCCAAGGGTGT
TTCCTCTCCATACTTGCCATTCGGTGGTGGTAGACACAGATGTATCGGTGAATTGTTCGCTTACTGTCAATTGGGTGTGTTGATGTCCATTTTCATCAGAACCATGAAATGGCGT
TACCCAACTGAAGGTGAAACTGTCCCACCATCTGACTTCACCTCCATGGTCACCCTACCAACTGCCCCAGCTAAGATCTACTGGGAAAAGAGACATCCAGAACAAAAGTACTAG
-

>19

ATGTCCACTGAAAACACTTCTTTGGTCGTTGAACTATTGGAGTACGTGAAGCTTGGTCTTTCGTACTTCCAAGCTCTGCCATTGGCACAGAGAGTGTCTATTATGGTCGCCTTGC
CATTTGTGTACACCATCACATGGCAATTGCTTTACTCCTTGAGAAAGGACAGACCACCACTTGTGTTCTACTGGATTCCATGGGTCGGCTCTGCTATCCCATACGGTACCAAGCC
ATACGAGTTCTTCGAAGACTGCCAAAAGAAATACGGTGATATCTTCTCTTTCATGCTATTGGGTAGAATTATGACTGTCTACTTGGGTCCAAAGGGTCACGAATTCATCTTCAAC
GCCAAGTTGGCCGATGTTTCCGCTGAAGCTGCTTACTCCCACTTGACCACCCCAGTGTTCGGTAAAGGTGTTATCTACGATTGTCCAAACCACAGACTAATGGAACAAAAGAAG
TTTGTCAAGGGTGCTTTGACTAAGGAAGCCTTTGTCAGATACGTTCCATTGATCGCTGAGGAAATCTACAAGTACTTCAGAAACTCCAAGAACTTCAAGATCAACGAAAACAAC
TCCGGTATCGTCGACGTTATGGTCTCCCAACCTGAAATGACTATCTTCACTGCTTCCAGATCCTTGCTAGGTAAGGAAATGAGAGACAAGTTGGACACCGACTTCGCTTACTTGT
ACAGTGACTTGGACAAGGGTTTCACCCCAATTAACTTCGTCTTCCCTAACTTGCCTCTAGAACACTACAGAAAGAGAGACCATGCCCAACAAGCTATCTCTGGTACTTACATGTC
CTTGATTAAGGAAAGACGTGAGAAGAACGACATCCAAAACCGTGACTTGATTGATGAATTGATGAAGAACTCCACTTACAAGGATGGTACTAAGATGACCGACCAAGAAATT
GCCAACCTATTGATTGGTGTCTTGATGGGTGGTCAACATACTTCCGCTGCTACCTCCGCTTGGTGTCTATTGCATTTGGCTGAAAGACCAGATGTCCAAGAAGAATTGTACCAA
GAACAAATGCGCGTCTTGAACAACGATACCAAGGAATTGACTTACGATGACCTACAAAACATGCCTCTATTGAACCAAATGATCAAGGAAACTTTGAGATTGCACCACCCATTG
CACTCTTTGTTCCGTAAAGTCATGAGAGATGTCGCTATTCCAAACACTTCCTACGTTGTCCCAAGGGACTACCACGTTCTAGTCTCCCCAGGTTACACTCACTTGCAAGAAGAATT
CTTCCCTAAGCCAAATGAATTCAACATCCACCGTTGGGACGGTGATGCTGCTTCTTCCAGTGCTGCTGGTGGTGACGAAGTTGATTACGGTTTCGGTGCTATCTCCAAGGGTGT
TTCCTCTCCATACTTGCCATTCGGTGGTGGTAGACACAGATGTATCGGTGAATTGTTCGCTTACTGTCAATTGGGTGTGTTGATGTCCATTTTCATCAGAACCATGAAATGGCGT
TACCCAACTGAAGGTGAAACTGTCCCACCATCTGACTTCACCTCCATGGTCACCCTACCAACTGCCCCAGCTAAGATCTACTGGGAAAAGAGACATCCAGAACAAAAGTACTAG
-

>26

ATGTCCACTGAAAACACTTCTTTGGTCGTTGAACTATTGGAGTACGTGAAGCTTGGTCTTTCGTACTTCCAAGCTCTGCCATTGGCGCAGAGAGTGTCTATTATGGTCGCCTTGC
CATTTGTGTACACCATCACATGGCAATTGCTTTACTCCTTGAGAAAGGACAGACCACCACTTGTGTTCTACTGGATCCCATGGGTCGGCTCTGCTATCCCATACGGTACCAAGCC
ATACGAGTTCTTCGAAGACTGCCAAAAGAAATACGGTGATATCTTCTCTTTCATGCTATTGGGTAGAATCATGACTGTCTACTTGGGTCCAAAGGGTCACGAATTCATCTTCAAC

GCCAAGTTGGCCGATGTTTCCGCTGAAGCTGCTTACTCCCACTTGACCACCCCAGTGTTCGGTAAAGGTGTTATCTACGATTGTCCAAACCACAGACTAATGGAACAAAAGAAG
TTTGTCAAGGGTGCTTTGACTAAGGAAGCCTTTGTCAGATACGTTCCATTGATCGCTGAGGAAATCTACAAGTACTTCAGAAACTCCAAGAACTTCAAGATCAACGAAAACAAC
TCCGGTATCGTCGACGTTATGGTCTCCCAACCTGAAATGACTATCTTCACTGCTTCCAGATCCTTGCTAGGTAAGGAAATGAGAGACAAGTTGGACACCGACTTCGCTTACTTGT
ACAGTGACTTGGACAAGGGTTTCACCCCAATTAACTTCGTCTTCCCTAACTTGCCTCTAGAACACTACAGAAAGAGAGACCATGCCCAACAAGCTATCTCTGGTACTTACATGTC
CTTGATTAAGGAAAGACGTGAGAAGAACGACATCCAAAACCGTGACTTGATTGATGAATTGATGAAGAACTCCACTTACAAGGATGGTACTAAGATGACCGACCAAGAAATT
GCCAACCTATTGATTGGTGTCTTGATGGGTGGTCAACATACTTCCGCTGCTACCTCCGCTTGGTGTCTATTGCATTTGGCTGAAAGACCAGATGTCCAAGAAGAATTGTACCAA
GAACAAATGCGCGTCTTGAACAACGATACCAAGGAATTGACTTACGATGACCTACAAAACATGCCTCTATTGAACCAAATGATCAAGGAAACTTTGAGATTGCACCACCCATTG
CACTCTTTGTTCCGTAAAGTCATGAGAGATGTCGCTATTCCAAACACTTCTTACGTTGTCCCAAGGGACTACCACGTTCTAGTCTCCCCAGGTTACACTCACTTGCAAGAAGAATT
CTTCCCTAAGCCAAATGAATTCAACATCCACCGTTGGGACGGTGATGCTGCTTCTTCCAGTGCTGCTGGTGGTGACGAAGTTGATTACGGTTTCGGTGCTATCTCCAAGGGTGT
GTCCTCTCCATACTTGCCATTCGGTGGTGGTAGACACAGATGTATCGGTGAATTGTTCGCTTACTGTCAATTGGGTGTGTTGATGTCCATTTTCATCAGAACCATGAAATGGCGT
TACCCAACTGAAGGTGAAACTGTCCCACCATCTGACTTCACCTCCATGGTCACCCTACCAACTGCCCCAGCTAAGATCTACTGGGAAAAGAGACATCCAGAACAAAAGTACTAG
-

>31

ATGTCCACTGAAAACACTTCTTTGGTCGTTGAACTATTGGAGTACGTGAAGCTTGGTCTTTCGTACTTCCAAGCTCTGCCATTGGCACAGAGAGTGTCTATTATGGTCGCCTTGC
CATTTGTGTACACCATCACATGGCAATTGCTTTACTCCTTGAGAAAGGACAGACCACCACTTGTGTTCTACTGGATTCCATGGGTCGGCTCTGCTATCCCATACGGTACCAAGCC
ATACGAGTTCTTCGAAGACTGCCAAAAGAAATACGGTGATATCTTCTCTTTCATGCTATTGGGTAGAATTATGACTGTCTACTTGGGTCCAAAGGGTCACGAATTCATCTTCAAC
GCCAAGTTGGCCGATGTTTCCGCTGAAGCTGCTTACTCCCACTTGACCACCCCAGTGTTCGGTAAAGGTGTTATCTACGATTGTCCAAACCACAGACTAATGGAACAAAAGAAG
TTTGTCAAGGGTGCTTTGACTAAGGAAGCCTTTGTCAGATACGTTCCATTGATCGCTGAGGAAATCTACAAGTACTTCAGAAACTCCAAGAACTTCAAGATCAACGAAAACAAC
TCCGGTATCGTCGACGTTATGGTCTCCCAACCTGAAATGACTATCTTCACTGCTTCCAGATCCTTGCTAGGTAAGGAAATGAGAGACAAGTTGGACACCGACTTCGCTTACTTGT
ACAGTGACTTGGACAAGGGTTTCACCCCAATTAACTTCGTCTTCCCTAACTTGCCTCTAGAACACTACAGAAAGAGAGACCATGCCCAACAAGCTATCTCTGGTACTTACATGTC
CTTGATTAAGGAAAGACGTGAGAAGAACGACATCCAAAACCGTGACTTGATTGATGAATTGATGAAGAACTCCACTTACAAGGATGGTACTAAGATGACCGACCAAGAAATT
GCCAACCTATTGATTGGTGTCTTGATGGGTGGTCAACATACTTCCGCTGCTACCTCCGCTTGGTGTCTATTGCATTTGGCTGAAAGACCAGATGTCCAAGAAGAATTGTACCAA
GAACAAATGCGCGTCTTGAACAACGATACCAAGGAATTGACTTACGATGACCTACAAAACATGCCTCTATTGAACCAAATGATCAAGGAAACTTTGAGATTGCACCACCCATTG
CACTCTTTGTTCCGTAAAGTCATGAGAGATGTCGCTATTCCAAACACTTCCTACGTTGTCCCAAGGGACTACCACGTTCTAGTCTCCCCAGGTTACACTCACTTGCAAGAAGAATT
CTTCCCTAAGCCAAATGAATTCAACATCCACCGTTGGGACGGTGATGCTGCTTCTTCCAGTGCTGCTGGTGGTGACGAAGTTGATTACGGTTTCGGTGCTATCTCCAAGGGTGT
TTCCTCTCCATACTTGCCATTCGGTGGTGGTAGACACAGATGTATCGGTGAATTGTTCGCTTACTGTCAATTGGGTGTGTTGATGTCCATTTTCATCAGAACCATGAAATGGCGT
TACCCAACTGAAGGTGAAACTGTCCCACCATCTGACTTCACCTCCATGGTCACCCTACCAACTGCCCCAGCTAAGATCTACTGGGAAAAGAGACATCCAGAACAAAAGTACTAG
-

>33

ATGTCCACTGAAAACACTTCTTTGGTCGTTGAACTATTGGAGTACGTGAAGCTTGGTCTTTCGTACTTCCAAGCTCTGCCATTGGCACAGAGAGTGTCTATTATGGTCGCCTTGC
CATTTGTGTACACCATCACATGGCAATTGCTTTACTCCTTGAGAAAGGACAGACCACCACTTGTGTTCTACTGGATTCCATGGGTCGGCTCTGCTATCCCATACGGTACCAAGCC
ATACGAGTTCTTCGAAGACTGCCAAAAGAAATACGGTGATATCTTCTCTTTCATGCTATTGGGTAGAATTATGACTGTCTACTTGGGTCCAAAGGGTCACGAATTCATCTTCAAC
GCCAAGTTGGCCGATGTTTCCGCTGAAGCTGCTTACTCCCACTTGACCACCCCAGTGTTCGGTAAAGGTGTTATCTACGATTGTCCAAACCACAGACTAATGGAACAAAAGAAG
TTTGTCAAGGGTGCTTTGACTAAGGAAGCCTTTGTCAGATACGTTCCATTGATCGCTGAGGAAATCTACAAGTACTTCAGAAACTCCAAGAACTTCAAGATCAACGAAAACAAC
TCCGGTATCGTCGACGTTATGGTCTCCCAACCTGAAATGACTATCTTCACTGCTTCCAGATCCTTGCTAGGTAAGGAAATGAGAGACAAGTTGGACACCGACTTCGCTTACTTGT
ACAGTGACTTGGACAAGGGTTTCACCCCAATTAACTTCGTCTTCCCTAACTTGCCTCTAGAACACTACAGAAAGAGAGACCATGCCCAACAAGCTATCTCTGGTACTTACATGTC
CTTGATTAAGGAAAGACGTGAGAAGAACGACATCCAAAACCGTGACTTGATTGATGAATTGATGAAGAACTCCACTTACAAGGATGGTACTAAGATGACCGACCAAGAAATT
GCCAACCTATTGATTGGTGTCTTGATGGGTGGTCAACATACTTCCGCTGCTACCTCCGCTTGGTGTCTATTGCATTTGGCTGAAAGACCAGATGTCCAAGAAGAATTGTACCAA
GAACAAATGCGCGTCTTGAACAACGATACCAAGGAATTGACTTACGATGACCTACAAAACATGCCTCTATTGAACCAAATGATCAAGGAAACTTTGAGATTGCACCACCCATTG
CACTCTTTGTTCCGTAAAGTCATGAGAGATGTCGCTATTCCAAACACTTCCTACGTTGTCCCAAGGGACTACCACGTTCTAGTCTCCCCAGGTTACACTCACTTGCAAGAAGAATT
CTTCCCTAAGCCAAATGAATTCAACATCCACCGTTGGGACGGTGATGCTGCTTCTTCCAGTGCTGCTGGTGGTGACGAAGTTGATTACGGTTTCGGTGCTATCTCCAAGGGTGT
TTCCTCTCCATACTTGCCATTCGGTGGTGGTAGACACAGATGTATCGGTGAATTGTTCGCTTACTGTCAATTGGGTGTGTTGATGTCCATTTTCATCAGAACCATGAAATGGCGT
TACCCAACTGAAGGTGAAACTGTCCCACCATCTGACTTCACCTCCATGGTCACCCTACCAACTGCCCCAGCTAAGATCTACTGGGAAAAGAGACATCCAGAACAAAAGTACTAG
-

>35

ATGTCCACTGAAAACACTTCTTTGGTCGTTGAACTATTGGAGTACGTGAAGCTTGGTCTTTCGTACTTCCAAGCTCTGCCATTGGCGCAGAGAGTGTCTATTATGGTCGCCTTGC
CATTTGTGTACACCATCACATGGCAATTGCTTTACTCCTTGAGAAAGGACAGACCACCACTTGTGTTCTACTGGATCCCATGGGTCGGCTCTGCTATCCCATACGGTACCAAGCC
ATACGAGTTCTTCGAAGACTGCCAAAAGAAATACGGTGATATCTTCTCTTTCATGCTATTGGGTAGAATTATGACTGTCTACTTGGGTCCAAAGGGTCACGAATTCATCTTCAAC
GCCAAGTTGGCCGATGTTTCCGCTGAAGCTGCTTACTCCCACTTGACCACCCCAGTGTTCGGTAAAGGTGTTATCTACGATTGTCCAAACCACAGACTAATGGAACAAAAGAAG
TTTGTCAAGGGTGCTTTGACTAAGGAAGCCTTTGTCAGATACGTTCCATTGATCGCTGAGGAAATCTACAAGTACTTCAGAAACTCCAAGAACTTCAAGATCAACGAAAACAAC
TCCGGTATCGTCGACGTTATGGTCTCCCAACCTGAAATGACTATCTTCACTGCTTCCAGATCCTTGCTAGGTAAGGAAATGAGAGACAAGTTGGACACCGACTTTGCTTACTTGT
ACAGTGACTTGGACAAGGGTTTCACCCCAATTAACTTCGTCTTCCCTAACTTGCCTCTAGAACACTACAGAAAGAGAGACCATGCCCAACAAGCTATCTCTGGTACTTACATGTC
CTTGATTAAGGAAAGACGTGAGAAGAACGACATCCAAAACCGTGACTTGATTGATGAATTGATGAAGAACTCCACTTACAAGGATGGTACTAAGATGACCGACCAAGAAATT
GCCAACCTATTGATTGGTGTCTTGATGGGTGGTCAACATACTTCCGCTGCTACCTCCGCTTGGTGTCTATTGCATTTGGCTGAAAGACCAGATGTCCAAGAAGAATTGTACCAA
GAACAAATGCGCGTCTTGAACAACGATACCAAGGAATTGACTTACGATGACCTACAAAACATGCCTCTATTGAACCAAATGATCAAGGAAACTTTGAGATTGCACCACCCATTG
CACTCTTTGTTCCGTAAAGTCATGAGAGATGTCGCTATTCCAAACACTTCCTACGTTGTCCCAAGGGACTACCACGTTCTAGTCTCCCCAGGTTACACTCACTTGCAAGAAGAATT
CTTCCCTAAGCCAAACGAATTCAACATCCACCGTTGGGACGGTGATGCTGCTTCTTCCAGTGCTGCTGGTGGTGACGAAGTTGATTACGGTTTCGGTGCTATCTCCAAGGGTGT
TTCCTCTCCATACTTGCCATTCGGTGGTGGTAGACACAGATGTATCGGTGAATTGTTCGCTTACTGTCAATTGGGTGTGTTGATGTCCATTTTCATCAGAACCATGAAATGGCGT

TACCCAACTGAAGGTGAAACTGTCCCACCATCTGACTTCACCTCCATGGTCACCCTACCAACTGCCCCAGCTAAGATCTACTGGGAAAAGAGACATCCAGAACAAAAGTACTAG
-

>40

ATGTCCACTGAAAACACTTCTTTGGTCGTTGAACTATTGGAGTACGTGAAGCTTGGTCTTTCGTACTTCCAAGCTCTGCCATTGGCACAGAGAGTGTCTATTATGGTCGCCTTGC
CATTTGTGTACACCATCACATGGCAATTGCTTTACTCCTTGAGAAAGGACAGACCACCACTTGTGTTCTACTGGATTCCATGGGTCGGCTCTGCTATCCCATACGGTACCAAGCC
ATACGAGTTCTTCGAAGACTGCCAAAAGAAATACGGTGATATCTTCTCTTTCATGCTATTGGGTAGAATTATGACTGTCTACTTGGGTCCAAAGGGTCACGAATTCATCTTCAAC
GCCAAGTTGGCCGATGTTTCCGCTGAAGCTGCTTACTCCCACTTGACCACCCCAGTGTTCGGTAAAGGTGTTATCTACGATTGTCCAAACCACAGACTAATGGAACAAAAGAAG
TTTGTCAAGGGTGCTTTGACTAAGGAAGCCTTTGTCAGATACGTTCCATTGATCGCTGAGGAAATCTACAAGTACTTCAGAAACTCCAAGAACTTCAAGATCAACGAAAACAAC
TCCGGTATCGTCGACGTTATGGTCTCCCAACCTGAAATGACTATCTTCACTGCTTCCAGATCCTTGCTAGGTAAGGAAATGAGAGACAAGTTGGACACCGACTTCGCTTACTTGT
ACAGTGACTTGGACAAGGGTTTCACCCCAATTAACTTCGTCTTCCCTAACTTGCCTCTAGAACACTACAGAAAGAGAGACCATGCCCAACAAGCTATCTCTGGTACTTACATGTC
CTTGATTAAGGAAAGACGTGAGAAGAACGACATCCAAAACCGTGACTTGATTGATGAATTGATGAAGAACTCCACTTACAAGGATGGTACTAAGATGACCGACCAAGAAATT
GCCAACCTATTGATTGGTGTCTTGATGGGTGGTCAACATACTTCCGCTGCTACCTCCGCTTGGTGTCTATTGCATTTGGCTGAAAGACCAGATGTCCAAGAAGAATTGTACCAA
GAACAAATGCGCGTCTTGAACAACGATACCAAGGAATTGACTTACGATGACCTACAAAACATGCCTCTATTGAACCAAATGATCAAGGAAACTTTGAGATTGCACCACCCATTG
CACTCTTTGTTCCGTAAAGTCATGAGAGATGTCGCTATTCCAAACACTTCCTACGTTGTCCCAAGGGACTACCACGTTCTAGTCTCCCCAGGTTACACTCACTTGCAAGAAGAATT
CTTCCCTAAGCCAAATGAATTCAACATCCACCGTTGGGACGGTGATGCTGCTTCTTCCAGTGCTGCTGGTGGTGACGAAGTTGATTACGGTTTCGGTGCTATCTCCAAGGGTGT
TTCCTCTCCATACTTGCCATTCGGTGGTGGTAGACACAGATGTATCGGTGAATTGTTCGCTTACTGTCAATTGGGTGTGTTGATGTCCATTTTCATCAGAACCATGAAATGGCGT
TACCCAACTGAAGGTGAAACTGTCCCACCATCTGACTTCACCTCCATGGTCACCCTACCAACTGCCCCAGCTAAGATCTACTGGGAAAAGAGACATCCAGAACAAAAGTACTAG
-

>41

ATGTCCACTGAAAACACTTCTTTGGTCGTTGAACTATTGGAGTACGTGAAGCTTGGTCTTTCGTACTTCCAAGCTCTGCCATTGGCGCAGAGAGTGTCTATTATGGTCGCCTTGC
CATTTGTGTACACCATCACATGGCAATTGCTTTACTCCTTGAGAAAGGACAGACCACCACTTGTGTTCTACTGGATCCCATGGGTCGGCTCTGCTATCCCATACGGTACCAAGCC
ATACGAGTTCTTCGAAGACTGCCAAAAGAAATACGGTGATATCTTCTCTTTCATGCTATTGGGTAGAATTATGACTGTCTACTTGGGTCCAAAGGGTCACGAATTCATCTTCAAC
GCCAAGTTGGCCGATGTTTCCGCTGAAGCTGCTTACTCCCACTTGACCACCCCGGTGTTCGGTAAAGGTGTTATCTACGATTGTCCAAACCACAGACTAATGGAACAAAAGAAG
TTTGTCAAGGGTGCTTTGACTAAGGAAGCCTTTGTCAGATACGTTCCATTGATCGCTGAGGAAATCTACAAGTACTTCAGAAACTCCAAGAACTTCAAGATCAACGAAAACAAC
TCCGGTATCGTCGACGTTATGGTCTCCCAACCTGAAATGACTATCTTCACTGCTTCCAGATCCTTGCTAGGTAAGGAAATGAGAGACAAGTTGGACACCGACTTCGCTTACTTGT
ACAGTGACTTGGACAAGGGTTTCACCCCAATTAACTTCGTCTTCCCTAACTTGCCTCTAGAACACTACAGAAAGAGAGACCATGCCCAACAAGCTATCTCTGGTACTTACATGTC
CTTGATTAAGGAAAGACGTGAGAAGAACGATATCCAAAACCGTGACTTGATTGATGAATTGATGAAGAACTCCACTTACAAGGATGGTACTAAGATGACCGACCAAGAAATTG
CCAACCTATTGATTGGTGTCTTGATGGGTGGTCAACATACTTCCGCTGCTACCTCCGCTTGGTGTCTATTGCATTTGGCTGAAAGACCAGATGTCCAAGAAGAATTGTACCAAG
AACAAATGCGCGTCTTGAACAACGATACCAAGGAATTGACTTACGATGACCTACAAAACATGCCTCTATTGAACCAAATGATCAAGGAAACTTTGAGATTGCACCACCCATTGC

ACTCTTTGTTCCGTAAAGTCATGAGAGATGTCGCTATTCCAAACACTTCCTACGTTGTCCCAAGGGACTACCACGTTCTAGTCTCCCCAGGTTACACTCACTTGCAAGAAGAATTC
TTCCCTAAGCCAAACGAATTCAACATCCACCGTTGGGACGGTGATGCTGCTTCTTCCAGTGCTGCTGGTGGTGACGAAGTTGATTACGGTTTCGGTGCTATCTCCAAGGGTGTT
TCCTCTCCATACTTGCCATTCGGTGGTGGTAGACACAGATGTATCGGTGAATTGTTCGCTTACTGTCAATTGGGTGTGTTGATGTCCATTTTCATCAGAACCATGAAATGGCGTT
ACCCAACTGAAGGTGAAACTGTCCCACCATCTGACTTCACCTCCATGGTCACCCTACCAACTGCCCCAGCTAAGATCTACTGGGAAAAGAGACATCCAGAACAAAAGTACTAG-

>42

ATGTCCACTGAAAACACTTCTTTGGTCGTTGAACTATTGGAGTACGTGAAGCTTGGTCTTTCGTACTTCCAAGCTCTGCCATTGGCGCAGAGAGTGTCTATTATGGTCGCCTTGC
CATTTGTGTACACCATCACATGGCAATTGCTTTACTCCTTGAGAAAGGACAGACCACCACTTGTGTTCTACTGGATCCCATGGGTCGGCTCTGCTATCCCATACGGTACCAAGCC
ATACGAGTTCTTCGAAGACTGCCAAAAGAAATACGGTGATATCTTCTCTTTCATGCTATTGGGTAGAATTATGACTGTCTACTTGGGTCCAAAGGGTCACGAATTCATCTTCAAC
GCCAAGTTGGCCGATGTTTCCGCTGAAGCTGCTTACTCCCACTTGACCACCCCGGTGTTCGGTAAAGGTGTTATCTACGATTGTCCAAACCACAGACTAATGGAACAAAAGAAG
TTTGTCAAGGGTGCTTTGACTAAGGAAGCCTTTGTCAGATACGTTCCATTGATCGCTGAGGAAATCTACAAGTACTTCAGAAACTCCAAGAACTTCAAGATCAACGAAAACAAC
TCCGGTATCGTCGACGTTATGGTCTCCCAACCTGAAATGACTATCTTCACTGCTTCCAGATCCTTGCTAGGTAAGGAAATGAGAGACAAGTTGGACACCGACTTCGCTTACTTGT
ACAGTGACTTGGACAAGGGTTTCACCCCAATTAACTTCGTCTTCCCTAACTTGCCTCTAGAACACTACAGAAAGAGAGACCATGCCCAACAAGCTATCTCTGGTACTTACATGTC
CTTGATTAAGGAAAGACGTGAGAAGAACGATATCCAAAACCGTGACTTGATTGATGAATTGATGAAGAACTCCACTTACAAGGATGGTACTAAGATGACCGACCAAGAAATTG
CCAACCTATTGATTGGTGTCTTGATGGGTGGTCAACATACTTCCGCTGCTACCTCCGCTTGGTGTCTATTGCATTTGGCTGAAAGACCAGATGTCCAAGAAGAATTGTACCAAG
AACAAATGCGCGTCTTGAACAACGATACCAAGGAATTGACTTACGATGACCTACAAAACATGCCTCTATTGAACCAAATGATCAAGGAAACTTTGAGATTGCACCACCCATTGC
ACTCTTTGTTCCGTAAAGTCATGAGAGATGTCGCTATTCCAAACACTTCCTACGTTGTCCCAAGGGACTACCACGTTCTAGTCTCCCCAGGTTACACTCACTTGCAAGAAGAATTC
TTCCCTAAGCCAAACGAATTCAACATCCACCGTTGGGACGGTGATGCTGCTTCTTCCAGTGCTGCTGGTGGTGACGAAGTTGATTACGGTTTCGGTGCTATCTCCAAGGGTGTT
TCCTCTCCATACTTGCCATTCGGTGGTGGTAGACACAGATGTATCGGTGAATTGTTCGCTTACTGTCAATTGGGTGTGTTGATGTCCATTTTCATCAGAACCATGAAATGGCGTT
ACCCAACTGAAGGTGAAACTGTCCCACCATCTGACTTCACCTCCATGGTCACCCTACCAACTGCCCCAGCTAAGATCTACTGGGAAAAGAGACATCCAGAACAAAAGTACTAG-

>44

ATGTCCACTGAAAACACTTCTTTGGTCGTTGAACTATTGGAGTACGTGAAGCTTGGTCTTTCGTACTTCCAAGCTCTGCCATTGGCACAGAGAGTGTCTATTATGGTCGCCTTGC
CATTTGTGTACACCATCACATGGCAATTGCTTTACTCCTTGAGAAAGGACAGACCACCACTTGTGTTCTACTGGATTCCATGGGTCGGCTCTGCTATCCCATACGGTACCAAGCC
ATACGAGTTCTTCGAAGACTGCCAAAAGAAATACGGTGATATCTTCTCTTTCATGCTATTGGGTAGAATTATGACTGTCTACTTGGGTCCAAAGGGTCACGAATTCATCTTCAAC
GCCAAGTTGGCCGATGTTTCCGCTGAAGCTGCTTACTCCCACTTGACCACCCCAGTGTTCGGTAAAGGTGTTATCTACGATTGTCCAAACCACAGACTAATGGAACAAAAGAAG
TTTGTCAAGGGTGCTTTGACTAAGGAAGCCTTTGTCAGATACGTTCCATTGATCGCTGAGGAAATCTACAAGTACTTCAGAAACTCCAAGAACTTCAAGATCAACGAAAACAAC
TCCGGTATCGTCGACGTTATGGTCTCCCAACCTGAAATGACTATCTTCACTGCTTCCAGATCCTTGCTAGGTAAGGAAATGAGAGACAAGTTGGACACCGACTTCGCTTACTTGT
ACAGTGACTTGGACAAGGGTTTCACCCCAATTAACTTCGTCTTCCCTAACTTGCCTCTAGAACACTACAGAAAGAGAGACCATGCCCAACAAGCTATCTCTGGTACTTACATGTC
CTTGATTAAGGAAAGACGTGAGAAGAACGACATCCAAAACCGTGACTTGATTGATGAATTGATGAAGAACTCCACTTACAAGGATGGTACTAAGATGACCGACCAAGAAATT
GCCAACCTATTGATTGGTGTCTTGATGGGTGGTCAACATACTTCCGCTGCTACCTCCGCTTGGTGTCTATTGCATTTGGCTGAAAGACCAGATGTCCAAGAAGAATTGTACCAA

GAACAAATGCGCGTCTTGAACAACGATACCAAGGAATTGACTTACGATGACCTACAAAACATGCCTCTATTGAACCAAATGATCAAGGAAACTTTGAGATTGCACCACCCATTG
CACTCTTTGTTCCGTAAAGTCATGAGAGATGTCGCTATTCCAAACACTTCCTACGTTGTCCCAAGGGACTACCACGTTCTAGTCTCCCCAGGTTACACTCACTTGCAAGAAGAATT
CTTCCCTAAGCCAAATGAATTCAACATCCACCGTTGGGACGGTGATGCTGCTTCTTCCAGTGCTGCTGGTGGTGACGAAGTTGATTACGGTTTCGGTGCTATCTCCAAGGGTGT
TTCCTCTCCATACTTGCCATTCGGTGGTGGTAGACACAGATGTATCGGTGAATTGTTCGCTTACTGTCAATTGGGTGTGTTGATGTCCATTTTCATCAGAACCATGAAATGGCGT
TACCCAACTGAAGGTGAAACTGTCCCACCATCTGACTTCACCTCCATGGTCACCCTACCAACTGCCCCAGCTAAGATCTACTGGGAAAAGAGACATCCAGAACAAAAGTACTAG
-

>47

ATGTCCACTGAAAACACTTCTTTGGTCGTTGAACTATTGGAGTACGTGAAGCTTGGTCTTTCGTACTTCCAAGCTCTGCCATTGGCGCAGAGAGTGTCTATTATGGTCGCCTTGC
CATTTGTGTACACCATCACATGGCAATTGCTTTACTCCTTGAGAAAGGACAGACCACCACTTGTGTTCTACTGGATCCCATGGGTCGGCTCTGCTATCCCATACGGTACCAAGCC
ATACGAGTTCTTCGAAGACTGCCAAAAGAAATACGGTGATATCTTCTCTTTCATGCTATTGGGTAGAATTATGACTGTCTACTTGGGTCCAAAGGGTCACGAATTCATCTTCAAC
GCCAAGTTGGCCGATGTTTCCGCTGAAGCTGCTTACTCCCACTTGACCACCCCAGTGTTCGGTAAAGGTGTTATCTACGATTGTCCAAACCACAGACTAATGGAACAAAAGAAG
TTTGTCAAGGGTGCTTTGACTAAGGAAGCCTTTGTCAGATACGTTCCATTGATCGCTGAGGAAATCTACAAGTACTTCAGAAACTCCAAGAACTTCAAGATCAACGAAAACAAC
TCCGGTATCGTCGATGTTATGGTCTCCCAACCTGAAATGACTATCTTCACTGCTTCCAGATCCTTGCTAGGTAAGGAAATGAGAGACAAGTTGGACACCGACTTCGCTTACTTGT
ACAGTGACTTGGACAAGGGTTTCACCCCAATTAACTTCGTCTTCCCTAACTTGCCTCTAGAACACTACAGAAAGAGAGACCATGCCCAACAAGCTATCTCTGGTACTTACATGTC
CTTGATTAAGGAAAGACGTGAGAAGAACGATATCCAAAACCGTGACTTGATTGATGAATTGATGAAGAACTCCACTTACAAGGATGGTACTAAGATGACCGACCAAGAAATTG
CTAACCTATTGATTGGTGTCTTGATGGGTGGTCAACATACTTCCGCTGCTACCTCCGCTTGGTGTCTATTGCATTTGGCTGAAAGACCAGATGTCCAAGAAGAATTGTACCAAGA
ACAAATGCGCGTCTTGAACAACGATACCAAGGAATTGACTTACGATGACCTACAAAACATGCCTCTATTGAACCAAATGATCAAGGAAACTTTGAGATTGCACCACCCATTGCA
CTCTTTGTTCCGTAAAGTCATGAGAGATGTCGCTATTCCAAACACTTCCTACGTTGTCCCAAGGGACTACCACGTTCTAGTCTCCCCAGGTTACACTCACTTGCAAGAAGAATTCT
TCCCTAAGCCAAATGAATTCAACATCCACCGTTGGGACGGTGATGCTGCTTCTTCCAGTGCTGCTGGTGGTGACGAAGTTGATTACGGTTTCGGTGCTATCTCCAAGGGTGTTT
CCTCTCCATACTTGCCATTCGGTGGTGGTAGACACAGATGTATCGGTGAATTGTTCGCTTACTGTCAATTGGGTGTGTTGATGTCCATTTTCATCAGAACCATGAAATGGCGTTA
CCCAACTGAAGGTGAAACTGTCCCACCATCTGACTTCACCTCCATGGTCACCCTACCAACTGCCCCAGCTAAGATCTACTGGGAAAAGAGACATCCAGAACAAAAGTACTAG-

>48

ATGTCCACTGAAAACACTTCTTTGGTCGTTGAACTATTGGAGTACGTGAAGCTTGGTCTTTCGTACTTCCAAGCTCTGCCATTGGCGCAGAGAGTGTCTATTATGGTCGCCTTGC
CATTTGTGTACACCATCACATGGCAATTGCTTTACTCCTTGAGAAAGGACAGACCACCACTTGTGTTCTACTGGATCCCATGGGTCGGCTCTGCTATCCCATACGGTACCAAGCC
ATACGAGTTCTTCGAAGACTGCCAAAAGAAATACGGTGATATCTTCTCTTTCATGCTATTGGGTAGAATTATGACTGTCTACTTGGGTCCAAAGGGTCACGAATTCATCTTCAAC
GCCAAGTTGGCCGATGTTTCCGCTGAAGCTGCTTACTCCCACTTGACCACCCCAGTGTTCGGTAAAGGTGTTATCTACGATTGTCCAAACCACAGACTAATGGAACAAAAGAAG
TTTGTCAAGGGTGCTTTGACTAAGGAAGCCTTTGTCAGATACGTTCCATTGATCGCTGAGGAAATCTACAAGTACTTCAGAAACTCCAAGAACTTCAAGATCAACGAAAACAAC
TCCGGTATCGTCGATGTTATGGTCTCCCAACCTGAAATGACTATCTTCACTGCTTCCAGATCCTTGCTAGGTAAGGAAATGAGAGACAAGTTGGACACCGACTTCGCTTACTTGT
ACAGTGACTTGGACAAGGGTTTCACCCCAATTAACTTCGTCTTCCCTAACTTGCCTCTAGAACACTACAGAAAGAGAGACCATGCCCAACAAGCTATCTCTGGTACTTACATGTC

CTTGATTAAGGAAAGACGTGAGAAGAACGATATCCAAAACCGTGACTTGATTGATGAATTGATGAAGAACTCCACTTACAAGGATGGTACTAAGATGACCGACCAAGAAATTG
CTAACCTATTGATTGGTGTCTTGATGGGTGGTCAACATACTTCCGCTGCTACCTCCGCTTGGTGTCTATTGCATTTGGCTGAAAGACCAGATGTCCAAGAAGAATTGTACCAAGA
ACAAATGCGCGTCTTGAACAACGATACCAAGGAATTGACTTACGATGACCTACAAAACATGCCTCTATTGAACCAAATGATCAAGGAAACTTTGAGATTGCACCACCCATTGCA
CTCTTTGTTCCGTAAAGTCATGAGAGATGTCGCTATTCCAAACACTTCCTACGTTGTCCCAAGGGACTACCACGTTCTAGTCTCCCCAGGTTACACTCACTTGCAAGAAGAATTCT
TCCCTAAGCCAAATGAATTCAACATCCACCGTTGGGACGGTGATGCTGCTTCTTCCAGTGCTGCTGGTGGTGACGAAGTTGATTACGGTTTCGGTGCTATCTCCAAGGGTGTTT
CCTCTCCATACTTGCCATTCGGTGGTGGTAGACACAGATGTATCGGTGAATTGTTCGCTTACTGTCAATTGGGTGTGTTGATGTCCATTTTCATCAGAACCATGAAATGGCGTTA
CCCAACTGAAGGTGAAACTGTCCCACCATCTGACTTCACCTCCATGGTCACCCTACCAACTGCCCCAGCTAAGATCTACTGGGAAAAGAGACATCCAGAACAAAAGTACTAG-

>49

ATGTCCACTGAAAACACTTCTTTGGTCGTTGAACTATTGGAGTACGTGAAGCTTGGTCTTTCGTACTTCCAAGCTCTGCCATTGGCGCAGAGAGTGTCTATTATGGTCGCCTTGC
CATTTGTGTACACCATCACATGGCAATTGCTTTACTCCTTGAGAAAGGACAGACCACCACTTGTGTTCTACTGGATCCCATGGGTCGGCTCTGCTATCCCATACGGTACCAAGCC
ATACGAGTTCTTCGAAGACTGCCAAAAGAAATACGGTGATATCTTCTCTTTCATGCTATTGGGTAGAATCATGACTGTCTACTTGGGTCCAAAGGGTCACGAATTCATCTTCAAC
GCCAAGTTGGCCGATGTTTCCGCTGAAGCTGCTTACTCCCACTTGACCACCCCAGTGTTCGGTAAAGGTGTTATCTACGATTGTCCAAACCACAGACTAATGGAACAAAAGAAG
TTTGTCAAGGGTGCTTTGACTAAGGAAGCCTTTGTCAGATACGTTCCATTGATCGCTGAGGAAATCTACAAGTACTTCAGAAACTCCAAGAACTTCAAGATCAACGAAAACAAC
TCCGGTATCGTCGACGTTATGGTCTCCCAACCTGAAATGACTATCTTCACTGCTTCCAGATCCTTGCTAGGTAAGGAAATGAGAGACAAGTTGGACACCGACTTCGCTTACTTGT
ACAGTGACTTGGACAAGGGTTTCACCCCAATTAACTTCGTCTTCCCTAACTTGCCTCTAGAACACTACAGAAAGAGAGACCATGCCCAACAAGCTATCTCTGGTACTTACATGTC
CTTGATTAAGGAAAGACGTGAGAAGAACGACATCCAAAACCGTGACTTGATTGATGAATTGATGAAGAACTCCACTTACAAGGATGGTACTAAGATGACCGACCAAGAAATT
GCCAACCTATTGATTGGTGTCTTGATGGGTGGTCAACATACTTCCGCTGCTACCTCCGCTTGGTGTCTATTGCATTTGGCTGAAAGACCAGATGTCCAAGAAGAATTGTACCAA
GAACAAATGCGCGTCTTGAACAACGATACCAAGGAATTGACTTACGATGACCTACAAAACATGCCTCTATTGAACCAAATGATCAAGGAAACTTTGAGATTGCACCACCCATTG
CACTCTTTGTTCCGTAAAGTCATGAGAGATGTCGCTATTCCAAACACTTCTTACGTTGTCCCAAGGGACTACCACGTTCTAGTCTCCCCAGGTTACACTCACTTGCAAGAAGAATT
CTTCCCTAAGCCAAATGAATTCAACATCCACCGTTGGGACGGTGATGCTGCTTCTTCCAGTGCTGCTGGTGGTGACGAAGTTGATTACGGTTTCGGTGCTATCTCCAAGGGTGT
TTCCTCTCCATACTTGCCATTCGGTGGTGGTAGACACAGATGTATCGGTGAATTGTTCGCTTACTGTCAATTGGGTGTGTTGATGTCCATTTTCATCAGAACCATGAAATGGCGT
TACCCAACTGAAGGTGAAACTGTCCCACCATCTGACTTCACCTCCATGGTCACCCTACCAACTGCCCCAGCTAAGATCTACTGGGAAAAGAGACATCCAGAACAAAAGTACTAG
-

>52

ATGTCCACTGAAAACACTTCTTTGGTCGTTGAACTATTGGAGTACGTGAAGCTTGGTCTTTCGTACTTCCAAGCTCTGCCATTGGCGCAGAGAGTGTCTATTATGGTCGCCTTGC
CATTTGTGTACACCATCACATGGCAATTGCTTTACTCCTTGAGAAAGGACAGACCACCACTTGTGTTCTACTGGATCCCATGGGTCGGCTCTGCTATCCCATACGGTACCAAGCC
ATACGAGTTCTTCGAAGACTGCCAAAAGAAATACGGTGATATCTTCTCTTTCATGCTATTGGGTAGAATTATGACTGTCTACTTGGGTCCAAAGGGTCACGAATTCATCTTCAAC
GCCAAGTTGGCCGATGTTTCCGCTGAAGCTGCTTACTCCCACTTGACCACCCCGGTGTTCGGTAAAGGTGTTATCTACGATTGTCCAAACCACAGACTAATGGAACAAAAGAAG
TTTGTCAAGGGTGCTTTGACTAAGGAAGCCTTTGTCAGATACGTTCCATTGATCGCTGAGGAAATCTACAAGTACTTCAGAAACTCCAAGAACTTCAAGATCAACGAAAACAAC

TCCGGTATCGTCGACGTTATGGTCTCCCAACCTGAAATGACTATCTTCACTGCTTCCAGATCCTTGCTAGGTAAGGAAATGAGAGACAAGTTGGACACCGACTTCGCTTACTTGT
ACAGTGACTTGGACAAGGGTTTCACCCCAATTAACTTCGTCTTCCCTAACTTGCCTCTAGAACACTACAGAAAGAGAGACCATGCCCAACAAGCTATCTCTGGTACTTACATGTC
CTTGATTAAGGAAAGACGTGAGAAGAACGATATCCAAAACCGTGACTTGATTGATGAATTGATGAAGAACTCCACTTACAAGGATGGTACTAAGATGACCGACCAAGAAATTG
CCAACCTATTGATTGGTGTCTTGATGGGTGGTCAACATACTTCCGCTGCTACCTCCGCTTGGTGTCTATTGCATTTGGCTGAAAGACCAGATGTCCAAGAAGAATTGTACCAAG
AACAAATGCGCGTCTTGAACAACGATACCAAGGAATTGACTTACGATGACCTACAAAACATGCCTCTATTGAACCAAATGATCAAGGAAACTTTGAGATTGCACCACCCATTGC
ACTCTTTGTTCCGTAAAGTCATGAGAGATGTCGCTATTCCAAACACTTCCTACGTTGTCCCAAGGGACTACCACGTTCTAGTCTCCCCAGGTTACACTCACTTGCAAGAAGAATTC
TTCCCTAAGCCAAACGAATTCAACATCCACCGTTGGGACGGTGATGCTGCTTCTTCCAGTGCTGCTGGTGGTGACGAAGTTGATTACGGTTTCGGTGCTATCTCCAAGGGTGTT
TCCTCTCCATACTTGCCATTCGGTGGTGGTAGACACAGATGTATCGGTGAATTGTTCGCTTACTGTCAATTGGGTGTGTTGATGTCCATTTTCATCAGAACCATGAAATGGCGTT
ACCCAACTGAAGGTGAAACTGTCCCACCATCTGACTTCACCTCCATGGTCACCCTACCAACTGCCCCAGCTAAGATCTACTGGGAAAAGAGACATCCAGAACAAAAGTACTAG-

>55

ATGTCCACTGAAAACACTTCTTTGGTCGTTGAACTATTGGAGTACGTGAAGCTTGGTCTTTCGTACTTCCAAGCTCTGCCATTGGCGCAGAGAGTGTCTATTATGGTCGCCTTGC
CATTTGTGTACACCATCACATGGCAATTGCTTTACTCCTTGAGAAAGGACAGACCACCACTTGTGTTCTACTGGATCCCATGGGTCGGCTCTGCTATCCCATACGGTACCAAGCC
ATACGAGTTCTTCGAAGACTGCCAAAAGAAATACGGTGATATCTTCTCTTTCATGCTATTGGGTAGAATTATGACTGTCTACTTGGGTCCAAAGGGTCACGAATTCATCTTCAAC
GCCAAGTTGGCCGATGTTTCCGCTGAAGCTGCTTACTCCCACTTGACCACCCCGGTGTTCGGTAAAGGTGTTATCTACGATTGTCCAAACCACAGACTAATGGAACAAAAGAAG
TTTGTCAAGGGTGCTTTGACTAAGGAAGCCTTTGTCAGATACGTTCCATTGATCGCTGAGGAAATCTACAAGTACTTCAGAAACTCCAAGAACTTCAAGATCAACGAAAACAAC
TCCGGTATCGTCGACGTTATGGTCTCCCAACCTGAAATGACTATCTTCACTGCTTCCAGATCCTTGCTAGGTAAGGAAATGAGAGACAAGTTGGACACCGACTTCGCTTACTTGT
ACAGTGACTTGGACAAGGGTTTCACCCCAATTAACTTCGTCTTCCCTAACTTGCCTCTAGAACACTACAGAAAGAGAGACCATGCCCAACAAGCTATCTCTGGTACTTACATGTC
CTTGATTAAGGAAAGACGTGAGAAGAACGATATCCAAAACCGTGACTTGATTGATGAATTGATGAAGAACTCCACTTACAAGGATGGTACTAAGATGACCGACCAAGAAATTG
CCAACCTATTGATTGGTGTCTTGATGGGTGGTCAACATACTTCCGCTGCTACCTCCGCTTGGTGTCTATTGCATTTGGCTGAAAGACCAGATGTCCAAGAAGAATTGTACCAAG
AACAAATGCGCGTCTTGAACAACGATACCAAGGAATTGACTTACGATGACCTACAAAACATGCCTCTATTGAACCAAATGATCAAGGAAACTTTGAGATTGCACCACCCATTGC
ACTCTTTGTTCCGTAAAGTCATGAGAGATGTCGCTATTCCAAACACTTCCTACGTTGTCCCAAGGGACTACCACGTTCTAGTCTCCCCAGGTTACACTCACTTGCAAGAAGAATTC
TTCCCTAAGCCAAACGAATTCAACATCCACCGTTGGGACGGTGATGCTGCTTCTTCCAGTGCTGCTGGTGGTGACGAAGTTGATTACGGTTTCGGTGCTATCTCCAAGGGTGTT
TCCTCTCCATACTTGCCATTCGGTGGTGGTAGACACAGATGTATCGGTGAATTGTTCGCTTACTGTCAATTGGGTGTGTTGATGTCCATTTTCATCAGAACCATGAAATGGCGTT
ACCCAACTGAAGGTGAAACTGTCCCACCATCTGACTTCACCTCCATGGTCACCCTACCAACTGCCCCAGCTAAGATCTACTGGGAAAAGAGACATCCAGAACAAAAGTACTAG-

>53

ATGTCCACTGAAAACACTTCTTTGGTCGTTGAACTATTGGAGTACGTGAAGCTTGGTCTTTCGTACTTCCAAGCTCTGCCATTGGCGCAGAGAGTGTCTATTATGGTCGCCTTGC
CATTTGTGTACACCATCACATGGCAATTGCTTTACTCCTTGAGAAAGGACAGACCACCACTTGTGTTCTACTGGATCCCATGGGTCGGCTCTGCTATCCCATACGGTACCAAGCC
ATACGAGTTCTTCGAAGACTGCCAAAAGAAATACGGTGATATCTTCTCTTTCATGCTATTGGGTAGAATTATGACTGTCTACTTGGGTCCAAAGGGTCACGAATTCATCTTCAAC
GCCAAGTTGGCCGATGTTTCCGCTGAAGCTGCTTACTCCCACTTGACCACCCCAGTGTTCGGTAAAGGTGTTATCTACGATTGTCCAAACCACAGACTAATGGAACAAAAGAAG

TTTGTCAAGGGTGCTTTGACTAAGGAAGCCTTTGTCAGATACGTTCCATTGATCGCTGAGGAAATCTACAAGTACTTCAGAAACTCCAAGAACTTCAAGATCAACGAAAACAAC
TCCGGTATCGTCGACGTTATGGTCTCCCAACCTGAAATGACTATCTTCACTGCTTCCAGATCCTTGCTAGGTAAGGAAATGAGAGACAAGTTGGACACCGACTTCGCTTACTTGT
ACAGTGACTTGGACAAGGGTTTCACCCCAATTAACTTCGTCTTCCCTAACTTGCCTCTAGAACACTACAGAAAGAGAGACCATGCCCAACAAGCTATCTCTGGTACTTACATGTC
CTTGATTAAGGAAAGACGTGAGAAGAACGATATCCAAAACCGTGACTTGATTGATGAATTGATGAAGAACTCCACTTACAAGGATGGTACTAAGATGACCGACCAAGAAATTG
CCAACCTATTAATTGGTGTCTTGATGGGTGGTCAACATACTTCCGCTGCTACCTCCGCTTGGTGTCTATTGCATTTGGCTGAAAGACCAGATGTCCAAGAAGAATTGTACCAAGA
ACAAATGCGCGTCTTGAACAACGATACCAAGGAATTGACTTACGATGACCTACAAAACATGCCTCTATTGAACCAAATGATCAAGGAAACTTTGAGATTGCACCACCCATTGCA
CTCTTTGTTCCGTAAAGTCATGAGAGATGTCGCTATTCCAAACACTTCCTACGTTGTCCCAAGGGACTACCACGTTCTAGTCTCCCCAGGTTACACTCACTTGCAAGAAGAATTCT
TCCCTAAGCCAAATGAATTCAACATCCACCGTTGGGACGGTGATGCTGCTTCTTCCAGTGCTGCTGGTGGTGACGAAGTTGATTACGGTTTCGGTGCTATCTCCAAGGGTGTTT
CCTCTCCATACTTGCCATTCGGTGGTGGTAGACACAGATGTATCGGTGAATTGTTCGCTTACTGTCAATTGGGTGTGTTGATGTCCATTTTCATCAGAACCATGAAATGGCGTTA
CCCAACTGAAGGTGAAACTGTCCCACCATCTGACTTCACCTCCATGGTCACCCTACCAACTGCCCCAGCTAAGATCTACTGGGAAAAGAGACATCCAGAACAAAAGTACTAG-

>56

ATGTCCACTGAAAACACTTCTTTGGTCGTTGAACTATTGGAGTACGTGAAGCTTGGTCTTTCGTACTTCCAAGCTCTGCCATTGGCGCAGAGAGTGTCTATTATGGTCGCCTTGC
CATTTGTGTACACCATCACATGGCAATTGCTTTACTCCTTGAGAAAGGACAGACCACCACTTGTGTTCTACTGGATCCCATGGGTCGGCTCTGCTATCCCATACGGTACCAAGCC
ATACGAGTTCTTCGAAGACTGCCAAAAGAAATACGGTGATATCTTCTCTTTCATGCTATTGGGTAGAATCATGACTGTCTACTTGGGTCCAAAGGGTCACGAATTCATCTTCAAC
GCCAAGTTGGCCGATGTTTCCGCTGAAGCTGCTTACTCCCACTTGACCACCCCAGTGTTCGGTAAAGGTGTTATCTACGATTGTCCAAACCACAGACTAATGGAACAAAAGAAG
TTTGTCAAGGGTGCTTTGACTAAGGAAGCCTTTGTCAGATACGTTCCATTGATCGCTGAGGAAATCTACAAGTACTTCAGAAACTCCAAGAACTTCAAGATCAACGAAAACAAC
TCCGGTATCGTCGACGTTATGGTCTCCCAACCTGAAATGACTATCTTCACTGCTTCCAGATCCTTGCTAGGTAAGGAAATGAGAGACAAGTTGGACACCGACTTCGCTTACTTGT
ACAGTGACTTGGACAAGGGTTTCACCCCAATTAACTTCGTCTTCCCTAACTTGCCTCTAGAACACTACAGAAAGAGAGACCATGCCCAACAAGCTATCTCTGGTACTTACATGTC
CTTGATTAAGGAAAGACGTGAGAAGAACGACATCCAAAACCGTGACTTGATTGATGAATTGATGAAGAACTCCACTTACAAGGATGGTACTAAGATGACCGACCAAGAAATT
GCCAACCTATTGATTGGTGTCTTGATGGGTGGTCAACATACTTCCGCTGCTACCTCCGCTTGGTGTCTATTGCATTTGGCTGAAAGACCAGATGTCCAAGAAGAATTGTACCAA
GAACAAATGCGCGTCTTGAACAACGATACCAAGGAATTGACTTACGATGACCTACAAAACATGCCTCTATTGAACCAAATGATCAAGGAAACTTTGAGATTGCACCACCCATTG
CACTCTTTGTTCCGTAAAGTCATGAGAGATGTCGCTATTCCAAACACTTCTTACGTTGTCCCAAGGGACTACCACGTTCTAGTCTCCCCAGGTTACACTCACTTGCAAGAAGAATT
CTTCCCTAAGCCAAATGAATTCAACATCCACCGTTGGGACGGTGATGCTGCTTCTTCCAGTGCTGCTGGTGGTGACGAAGTTGATTACGGTTTCGGTGCTATCTCCAAGGGTGT
TTCCTCTCCATACTTGCCATTCGGTGGTGGTAGACACAGATGTATCGGTGAATTGTTCGCTTACTGTCAATTGGGTGTGTTGATGTCCATTTTCATCAGAACCATGAAATGGCGT
TACCCAACTGAAGGTGAAACTGTCCCACCATCTGACTTCACCTCCATGGTCACCCTACCAACTGCCCCAGCTAAGATCTACTGGGAAAAGAGACATCCAGAACAAAAGTACTAG
-

>28

ATGTCCACTGAAAACACTTCTTTGGTCGTTGAACTATTGGAGTACGTGAAGCTTGGTCTTTCGTACTTCCAAGCTCTGCCATTGGCGCAGAGAGTGTCTATTATGGTCGCCTTGC
CATTTGTGTACACCATCACATGGCAATTGCTTTACTCCTTGAGAAAGGACAGACCACCACTTGTGTTCTACTGGATCCCATGGGTCGGCTCTGCTATCCCATACGGTACCAAGCC

ATACGAGTTCTTCGAAGACTGCCAAAAGAAATACGGTGATATCTTCTCTTTCATGCTATTGGGTAGAATCATGACTGTCTACTTGGGTCCAAAGGGTCACGAATTCATCTTCAAC
GCCAAGTTGGCCGATGTTTCCGCTGAAGCTGCTTACTCCCACTTGACCACCCCAGTGTTCGGTAAAGGTGTTATCTACGATTGTCCAAACCACAGACTAATGGAACAAAAGAAG
TTTGTCAAGGGTGCTTTGACTAAGGAAGCCTTTGTCAGATACGTTCCATTGATCGCTGAGGAAATCTACAAGTACTTCAGAAACTCCAAGAACTTCAAGATCAACGAAAACAAC
TCCGGTATCGTCGACGTTATGGTCTCCCAACCTGAAATGACTATCTTCACTGCTTCCAGATCCTTGCTAGGTAAGGAAATGAGAGACAAGTTGGACACCGACTTCGCTTACTTGT
ACAGTGACTTGGACAAGGGTTTCACCCCAATTAACTTCGTCTTCCCTAACTTGCCTCTAGAACACTACAGAAAGAGAGACCATGCCCAACAAGCTATCTCTGGTACTTACATGTC
CTTGATTAAGGAAAGACGTGAGAAGAACGACATCCAAAACCGTGACTTGATTGATGAATTGATGAAGAACTCCACTTACAAGGATGGTACTAAGATGACCGACCAAGAAATT
GCCAACCTATTGATTGGTGTCTTGATGGGTGGTCAACATACTTCCGCTGCTACCTCCGCTTGGTGTCTATTGCATTTGGCTGAAAGACCAGATGTCCAAGAAGAATTGTACCAA
GAACAAATGCGCGTCTTGAACAACGATACCAAGGAATTGACTTACGATGACCTACAAAACATGCCTCTATTGAACCAAATGATCAAGGAAACTTTGAGATTGCACCACCCATTG
CACTCTTTGTTCCGTAAAGTCATGAGAGATGTCGCTATTCCAAACACTTCTTACGTTGTCCCAAGGGACTACCACGTTCTAGTCTCCCCAGGTTACACTCACTTGCAAGAAGAATT
CTTCCCTAAGCCAAATGAATTCAACATCCACCGTTGGGACGGTGATGCTGCTTCTTCCAGTGCTGCTGGTGGTGACGAAGTTGATTACGGTTTCGGTGCTATCTCCAAGGGTGT
TTCCTCTCCATACTTGCCATTCGGTGGTGGTAGACACAGATGTATCGGTGAATTGTTCGCTTACTGTCAATTGGGTGTGTTGATGTCCATTTTCATCAGAACCATGAAATGGCGT
TACCCAACTGAAGGTGAAACTGTCCCACCATCTGACTTCACCTCCATGGTCACCCTACCAACTGCCCCAGCTAAGATCTACTGGGAAAAGAGACATCCAGAACAAAAGTACTAG
-

>29

ATGTCCACTGAAAACACTTCTTTGGTCGTTGAACTATTGGAGTACGTGAAGCTTGGTCTTTCGTACTTCCAAGCTCTGCCATTGGCGCAGAGAGTGTCTATTATGGTCGCCTTGC
CATTTGTGTACACCATCACATGGCAATTGCTTTACTCCTTGAGAAAGGACAGACCACCACTTGTGTTCTACTGGATCCCATGGGTCGGCTCTGCTATCCCATACGGTACCAAGCC
ATACGAGTTCTTCGAAGACTGCCAAAAGAAATACGGTGATATCTTCTCTTTCATGCTATTGGGTAGAATTATGACTGTCTACTTGGGTCCAAAGGGTCACGAATTCATCTTCAAC
GCCAAGTTGGCCGATGTTTCCGCTGAAGCTGCTTACTCCCACTTGACCACCCCAGTGTTCGGTAAAGGTGTTATCTACGATTGTCCAAACCACAGACTAATGGAACAAAAGAAG
TTTGTCAAGGGTGCTTTGACTAAGGAAGCCTTTGTCAGATACGTTCCATTGATCGCTGAGGAAATCTACAAGTACTTCAGAAACTCCAAGAACTTCAAGATCAACGAAAACAAC
TCCGGTATCGTCGATGTTATGGTCTCCCAACCTGAAATGACTATCTTCACTGCTTCCAGATCCTTGCTAGGTAAGGAAATGAGAGACAAGTTGGACACCGACTTCGCTTACTTGT
ACAGTGACTTGGACAAGGGTTTCACCCCAATTAACTTCGTCTTCCCTAACTTGCCTCTAGAACACTACAGAAAGAGAGACCATGCCCAACAAGCTATCTCTGGTACTTACATGTC
CTTGATTAAGGAAAGACGTGAGAAGAACGATATCCAAAACCGTGACTTGATTGATGAATTGATGAAGAACTCCACTTACAAGGATGGTACTAAGATGACCGACCAAGAAATTG
CTAACCTATTGATTGGTGTCTTGATGGGTGGTCAACATACTTCCGCTGCTACCTCCGCTTGGTGTCTATTGCATTTGGCTGAAAGACCAGATGTCCAAGAAGAATTGTACCAAGA
ACAAATGCGCGTCTTGAACAACGATACCAAGGAATTGACTTACGATGACCTACAAAACATGCCTCTATTGAACCAAATGATCAAGGAAACTTTGAGATTGCACCACCCATTGCA
CTCTTTGTTCCGTAAAGTCATGAGAGATGTCGCTATTCCAAACACTTCCTACGTTGTCCCAAGGGACTACCACGTTCTAGTCTCCCCAGGTTACACTCACTTGCAAGAAGAATTCT
TCCCTAAGCCAAATGAATTCAACATCCACCGTTGGGACGGTGATGCTGCTTCTTCCAGTGCTGCTGGTGGTGACGAAGTTGATTACGGTTTCGGTGCTATCTCCAAGGGTGTTT
CCTCTCCATACTTGCCATTCGGTGGTGGTAGACACAGATGTATCGGTGAATTGTTCGCTTACTGTCAATTGGGTGTGTTGATGTCCATTTTCATCAGAACCATGAAATGGCGTTA
CCCAACTGAAGGTGAAACTGTCCCACCATCTGACTTCACCTCCATGGTCACCCTACCAACTGCCCCAGCTAAGATCTACTGGGAAAAGAGACATCCAGAACAAAAGTACTAG-

>30

ATGTCCACTGAAAACACTTCTTTGGTCGTTGAACTATTGGAGTACGTGAAGCTTGGTCTTTCGTACTTCCAAGCTCTGCCATTGGCGCAGAGAGTGTCTATTATGGTCGCCTTGC
CATTTGTGTACACCATCACATGGCAATTGCTTTACTCCTTGAGAAAGGACAGACCACCACTTGTGTTCTACTGGATCCCATGGGTCGGCTCTGCTATCCCATACGGTACCAAGCC
ATACGAGTTCTTCGAAGACTGCCAAAAGAAATACGGTGATATCTTCTCTTTCATGCTATTGGGTAGAATCATGACTGTCTACTTGGGTCCAAAGGGTCACGAATTCATCTTCAAC
GCCAAGTTGGCCGATGTTTCCGCTGAAGCTGCTTACTCCCACTTGACCACCCCAGTGTTCGGTAAAGGTGTTATCTACGATTGTCCAAACCACAGACTAATGGAACAAAAGAAG
TTTGTCAAGGGTGCTTTGACTAAGGAAGCCTTTGTCAGATACGTTCCATTGATCGCTGAGGAAATCTACAAGTACTTCAGAAACTCCAAGAACTTCAAGATCAACGAAAACAAC
TCCGGTATCGTCGACGTTATGGTCTCCCAACCTGAAATGACTATCTTCACTGCTTCCAGATCCTTGCTAGGTAAGGAAATGAGAGACAAGTTGGACACCGACTTCGCTTACTTGT
ACAGTGACTTGGACAAGGGTTTCACCCCAATTAACTTCGTCTTCCCTAACTTGCCTCTAGAACACTACAGAAAGAGAGACCATGCCCAACAAGCTATCTCTGGTACTTACATGTC
CTTGATTAAGGAAAGACGTGAGAAGAACGACATCCAAAACCGTGACTTGATTGATGAATTGATGAAGAACTCCACTTACAAGGATGGTACTAAGATGACCGACCAAGAAATT
GCCAACCTATTGATTGGTGTCTTGATGGGTGGTCAACATACTTCCGCTGCTACCTCCGCTTGGTGTCTATTGCATTTGGCTGAAAGACCAGATGTCCAAGAAGAATTGTACCAA
GAACAAATGCGCGTCTTGAACAACGATACCAAGGAATTGACTTACGATGACCTACAAAACATGCCTCTATTGAACCAAATGATCAAGGAAACTTTGAGATTGCACCACCCATTG
CACTCTTTGTTCCGTAAAGTCATGAGAGATGTCGCTATTCCAAACACTTCTTACGTTGTCCCAAGGGACTACCACGTTCTAGTCTCCCCAGGTTACACTCACTTGCAAGAAGAATT
CTTCCCTAAGCCAAATGAATTCAACATCCACCGTTGGGACGGTGATGCTGCTTCTTCCAGTGCTGCTGGTGGTGACGAAGTTGATTACGGTTTCGGTGCTATCTCCARGKGTKTT
TCCTCTCCATACTTGCCATTCGGTGGTGGTAGACACAGATGTATCGGTGAATTGTTCGCTTACTGTCAATTGGGTGTGTTGATGTCCATTTTCATCAGAACCATGAAATGGCGTT
ACCCAACTGAAGGTGAAACTGTCCCACCATCTGACTTCACCTCCATGGTCACCCTACCAACTGCCCCAGCTAAGATCTACTGGGAAAAGAGACATCCAGAACAAAAGTACTAG-

>32

ATGTCCACTGAAAACACTTCTTTGGTCGTTGAACTATTGGAGTACGTGAAGCTTGGTCTTTCGTACTTCCAAGCTCTGCCATTGGCGCAGAGAGTGTCTATTATGGTCGCCTTGC
CATTTGTGTACACCATCACATGGCAATTGCTTTACTCCTTGAGAAAGGACAGACCACCACTTGTGTTCTACTGGATCCCATGGGTCGGCTCTGCTATCCCATACGGTACCAAGCC
ATACGAGTTCTTCGAAGACTGCCAAAAGAAATACGGTGATATCTTCTCTTTCATGCTATTGGGTAGAATTATGACTGTCTACTTGGGTCCAAAGGGTCACGAATTCATCTTCAAC
GCCAAGTTGGCCGATGTTTCCGCTGAAGCTGCTTACTCCCACTTGACCACCCCAGTGTTCGGTAAAGGTGTTATCTACGATTGTCCAAACCACAGACTAATGGAACAAAAGAAG
TTTGTCAAGGGTGCTTTGACTAAGGAAGCCTTTGTCAGATACGTTCCATTGATCGCTGAGGAAATCTACAAGTACTTCAGAAACTCCAAGAACTTCAAGATCAACGAAAACAAC
TCCGGTATCGTCGACGTTATGGTTTCACAACCTGAAATGACTATCTTCACTGCTTCCAGATCCTTGCTAGGTAAGGAAATGAGAGACAAGTTGGACACCGACTTCGCTTACTTGT
ACAGTGACTTGGACAAGGGTTTCACCCCAATTAACTTCGTCTTCCCTAACTTGCCTCTAGAACACTACAGAAAGAGAGACCATGCCCAACAAGCTATCTCTGGTACTTACATGTC
CTTGATTAAGGAAAGACGTGAGAAGAACGATATCCAAAACCGTGACTTGATTGATGAATTGATGAAGAACTCCACTTACAAGGATGGTACTAAGATGACCGACCAAGAAATTG
CCAACCTATTGATTGGTGTCTTGATGGGTGGTCAACATACTTCCGCTGCTACCTCCGCTTGGTGTCTATTGCATTTGGCTGAAAGACCAGATGTCCAAGAAGAATTGTACCAAG
AACAAATGCGCGTCTTGAACAACGATACCAAGGAATTGACTTACGATGACCTACAAAACATGCCTCTATTGAACCAAATGATCAAGGAAACTTTGAGATTGCACCACCCATTGC
ACTCTTTGTTCCGTAAAGTCATGAGAGATGTCGCTATTCCAAACACTTCCTACGTTGTCCCAAGGGACTACCACGTTCTAGTCTCCCCAGGTTACACTCACTTGCAAGAAGAATTC
TTCCCTAAGCCAAACGAATTCAACATCCACCGTTGGGACGGTGATGCTGCTTCTTCCAGTGCTGCTGGTGGTGACGAAGTTGATTACGGTTTCGGTGCTATCTCCARGKGTKTTT
CCTCTCCATACTTGCCATTCGGTGGTGGTAGACACAGATGTATCGGTGAATTGTTCGCTTACTGTCAATTGGGTGTGTTGATGTCCATTTTCATCAGAACCATGAAATGGCGTTA
CCCAACTGAAGGTGAAACTGTCCCACCATCTGACTTCACCTCCATGGTCACCCTACCAACTGCCCCAGCTAAGATCTACTGGGAAAAGAGACATCCAGAACAAAAGTACTAG-

>34

ATGTCCACTGAAAACACTTCTTTGGTCGTTGAACTATTGGAGTACGTGAAGCTTGGTCTTTCGTACTTCCAAGCTCTGCCATTGGCGCAGAGAGTGTCTATTATGGTCGCCTTGC
CATTTGTGTACACCATCACATGGCAATTGCTTTACTCCTTGAGAAAGGACAGACCACCACTTGTGTTCTACTGGATCCCATGGGTCGGCTCTGCTATCCCATACGGTACCAAGCC
ATACGAGTTCTTCGAAGACTGCCAAAAGAAATACGGTGATATCTTCTCTTTCATGCTATTGGGTAGAATTATGACTGTCTACTTGGGTCCAAAGGGTCACGAATTCATCTTCAAC
GCCAAGTTGGCCGATGTTTCCGCTGAAGCTGCTTACTCCCACTTGACCACCCCAGTGTTCGGTAAAGGTGTTATCTACGATTGTCCAAACCACAGACTAATGGAACAAAAGAAG
TTTGTCAAGGGTGCTTTGACTAAGGAAGCCTTTGTCAGATACGTTCCATTGATCGCTGAGGAAATCTACAAGTACTTCAGAAACTCCAAGAACTTCAAGATCAACGAAAACAAC
TCCGGTATCGTCGACGTTATGGTCTCCCAACCTGAAATGACTATCTTCACTGCTTCCAGATCCTTGCTAGGTAAGGAAATGAGAGACAAGTTGGACACCGACTTTGCTTACTTGT
ACAGTGACTTGGACAAGGGTTTCACCCCAATTAACTTCGTCTTCCCTAACTTGCCTCTAGAACACTACAGAAAGAGAGACCATGCCCAACAAGCTATCTCTGGTACTTACATGTC
CTTGATTAAGGAAAGACGTGAGAAGAACGACATCCAAAACCGTGACTTGATTGATGAATTGATGAAGAACTCCACTTACAAGGATGGTACTAAGATGACCGACCAAGAAATT
GCCAACCTATTGATTGGTGTCTTGATGGGTGGTCAACATACTTCCGCTGCTACCTCCGCTTGGTGTCTATTGCATTTGGCTGAAAGACCAGATGTCCAAGAAGAATTGTACCAA
GAACAAATGCGCGTCTTGAACAACGATACCAAGGAATTGACTTACGATGACCTACAAAACATGCCTCTATTGAACCAAATGATCAAGGAAACTTTGAGATTGCACCACCCATTG
CACTCTTTGTTCCGTAAAGTCATGAGAGATGTCGCTATTCCAAACACTTCCTACGTTGTCCCAAGGGACTACCACGTTCTAGTCTCCCCAGGTTACACTCACTTGCAAGAAGAATT
CTTCCCTAAGCCAAACGAATTCAACATCCACCGTTGGGACGGTGATGCTGCTTCTTCCAGTGCTGCTGGTGGTGACGAAGTTGATTACGGTTTCGGTGCTATCTCCAAGGGTGT
TTCCTCTCCATACTTGCCATTCGGTGGTGGTAGACACAGATGTATCGGTGAATTGTTCGCTTACTGTCAATTGGGTGTGTTGATGTCCATTTTCATCAGAACCATGAAATGGCGT
TACCCAACTGAAGGTGAAACTGTCCCACCATCTGACTTCACCTCCATGGTCACCCTACCAACTGCCCCAGCTAAGATCTACTGGGAAAAGAGACATCCAGAACAAAAGTACTAG
-

>36

ATGTCCACTGAAAACACTTCTTTGGTCGTTGAACTATTGGAGTACGTGAAGCTTGGTCTTTCGTACTTCCAAGCTCTGCCATTGGCGCAGAGAGTGTCTATTATGGTCGCCTTGC
CATTTGTGTACACCATCACATGGCAATTGCTTTACTCCTTGAGAAAGGACAGACCACCACTTGTGTTCTACTGGATCCCATGGGTCGGCTCTGCTATCCCATACGGTACCAAGCC
ATACGAGTTCTTCGAAGACTGCCAAAAGAAATACGGTGATATCTTCTCTTTCATGCTATTGGGTAGAATTATGACTGTCTACTTGGGTCCAAAGGGTCACGAATTCATCTTCAAC
GCCAAGTTGGCCGATGTTTCCGCTGAAGCTGCTTACTCCCACTTGACCACCCCAGTGTTCGGTAAAGGTGTTATCTACGATTGTCCAAACCACAGACTAATGGAACAAAAGAAG
TTTGTCAAGGGTGCTTTGACTAAGGAAGCCTTTGTCAGATACGTTCCATTGATCGCTGAGGAAATCTACAAGTACTTCAGAAACTCCAAGAACTTCAAGATCAACGAAAACAAC
TCCGGTATCGTCGACGTTATGGTCTCCCAACCTGAAATGACTATCTTCACTGCTTCCAGATCCTTGCTAGGTAAGGAAATGAGAGACAAGTTGGACACCGACTTCGCTTACTTGT
ACAGTGACTTGGACAAGGGTTTCACCCCAATTAACTTCGTCTTCCCTAACTTGCCTCTAGAACACTACAGAAAGAGAGACCATGCCCAACAAGCTATCTCTGGTACTTACATGTC
CTTGATTAAGGAAAGACGTGAGAAGAACGACATCCAAAACCGTGACTTGATTGATGAATTGATGAAGAACTCCACTTACAAGGATGGTACTAAGATGACCGACCAAGAAATT
GCCAACCTATTGATTGGTGTCTTGATGGGTGGTCAACATACTTCCGCTGCTACCTCCGCTTGGTGTCTATTGCATTTGGCTGAAAGACCAGATGTCCAAGAAGAATTGTACCAA
GAACAAATGCGCGTCTTGAACAACGATACCAAGGAATTGACTTACGATGACCTACAAAACATGCCTCTATTGAACCAAATGATCAAGGAAACTTTGAGATTGCACCACCCATTG
CACTCTTTGTTCCGTAAAGTCATGAGAGATGTCGCTATTCCAAACACTTCCTACGTTGTCCCAAGGGACTACCACGTTCTAGTCTCCCCAGGTTACACTCACTTGCAAGAAGAATT
CTTCCCTAAGCCAAATGAATTCAACATCCACCGTTGGGACGGTGATGCTGCTTCTTCCAGTGCTGCTGGTGGTGACGAAGTTGATTACGGTTTCGGTGCTATCTCCAAGGGTGT

TTCCTCTCCATACTTGCCATTCGGTGGTGGTAGACACAGATGTATCGGTGAATTGTTCGCTTACTGTCAATTGGGTGTGTTGATGTCCATTTTCATCAGAACCATGAAATGGCGT
TACCCAACTGAAGGTGAAACTGTCCCACCATCTGACTTCACCTCCATGGTCACCCTACCAACTGCCCCAGCTAAGATCTACTGGGAAAAGAGACATCCAGAACAAAAGTACTAG
-

>37

ATGTCCACTGAAAACACTTCTTTGGTCGTTGAACTATTGGAGTACGTGAAGCTTGGTCTTTCGTACTTCCAAGCTCTGCCATTGGCGCAGAGAGTGTCTATTATGGTCGCCTTGC
CATTTGTGTACACCATCACATGGCAATTGCTTTACTCCTTGAGAAAGGACAGACCACCACTTGTGTTCTACTGGATCCCATGGGTCGGCTCTGCTATCCCATACGGTACCAAGCC
ATACGAGTTCTTCGAAGACTGCCAAAAGAAATACGGTGATATCTTCTCTTTCATGCTATTGGGTAGAATTATGACTGTCTACTTGGGTCCAAAGGGTCACGAATTCATCTTCAAC
GCCAAGTTGGCCGATGTTTCCGCTGAAGCTGCTTACTCCCACTTGACCACCCCAGTGTTCGGTAAAGGTGTTATCTACGATTGTCCAAACCACAGACTAATGGAACAAAAGAAG
TTTGTCAAGGGTGCTTTGACTAAGGAAGCCTTTGTCAGATACGTTCCATTGATCGCTGAGGAAATCTACAAGTACTTCAGAAACTCCAAGAACTTCAAGATCAACGAAAACAAC
TCCGGTATCGTCGACGTTATGGTCTCCCAACCTGAAATGACTATCTTCACTGCTTCCAGATCCTTGCTAGGTAAGGAAATGAGAGACAAGTTGGACACCGACTTCGCTTACTTGT
ACAGTGACTTGGACAAGGGTTTCACCCCAATTAACTTCGTCTTCCCTAACTTGCCTCTAGAACACTACAGAAAGAGAGACCATGCCCAACAAGCTATCTCTGGTACTTACATGTC
CTTGATTAAGGAAAGACGTGAGAAGAACGACATCCAAAACCGTGACTTGATTGATGAATTGATGAAGAACTCCACTTACAAGGATGGTACTAAGATGACCGACCAAGAAATT
GCCAACCTATTGATTGGTGTCTTGATGGGTGGTCAACATACTTCCGCTGCTACCTCCGCTTGGTGTCTATTGCATTTGGCTGAAAGACCAGATGTCCAAGAAGAATTGTACCAA
GAACAAATGCGCGTCTTGAACAACGATACCAAGGAATTGACTTACGATGACCTACAAAACATGCCTCTATTGAACCAAATGATCAAGGAAACTTTGAGATTGCACCACCCATTG
CACTCTTTGTTCCGTAAAGTCATGAGAGATGTCGCTATTCCAAACACTTCCTACGTTGTCCCAAGGGACTACCACGTTCTAGTCTCCCCAGGTTACACTCACTTGCAAGAAGAATT
CTTCCCTAAGCCAAATGAATTCAACATCCACCGTTGGGACGGTGATGCTGCTTCTTCCAGTGCTGCTGGTGGTGACGAAGTTGATTACGGTTTCGGTGCTATCTCCAAGGGTGT
TTCCTCTCCATACTTGCCATTCGGTGGTGGTAGACACAGATGTATCGGTGAATTGTTCGCTTACTGTCAATTGGGTGTGTTGATGTCCATTTTCATCAGAACCATGAAATGGCGT
TACCCAACTGAAGGTGAAACTGTCCCACCATCTGACTTCACCTCCATGGTCACCCTACCAACTGCCCCAGCTAAGATCTACTGGGAAAAGAGACATCCAGAACAAAAGTACTAG
-

>38

ATGTCCACTGAAAACACTTCTTTGGTCGTTGAACTATTGGAGTACGTGAAGCTTGGTCTTTCGTACTTCCAAGCTCTGCCATTGGCGCAGAGAGTGTCTATTATGGTCGCCTTGC
CATTTGTGTACACCATCACATGGCAATTGCTTTACTCCTTGAGAAAGGACAGACCACCACTTGTGTTCTACTGGATCCCATGGGTCGGCTCTGCTATCCCATACGGTACCAAGCC
ATACGAGTTCTTCGAAGACTGCCAAAAGAAATACGGTGATATCTTCTCTTTCATGCTATTGGGTAGAATCATGACTGTCTACTTGGGTCCAAAGGGTCACGAATTCATCTTCAAC
GCCAAGTTGGCCGATGTTTCCGCTGAAGCTGCTTACTCCCACTTGACCACCCCAGTGTTCGGTAAAGGTGTTATCTACGATTGTCCAAACCACAGACTAATGGAACAAAAGAAG
TTTGTCAAGGGTGCTTTGACTAAGGAAGCCTTTGTCAGATACGTTCCATTGATCGCTGAGGAAATCTACAAGTACTTCAGAAACTCCAAGAACTTCAAGATCAACGAAAACAAC
TCCGGTATCGTCGACGTTATGGTCTCCCAACCTGAAATGACTATCTTCACTGCTTCCAGATCCTTGCTAGGTAAGGAAATGAGAGACAAGTTGGACACCGACTTCGCTTACTTGT
ACAGTGACTTGGACAAGGGTTTCACCCCAATTAACTTCGTCTTCCCTAACTTGCCTCTAGAACACTACAGAAAGAGAGACCATGCCCAACAAGCTATCTCTGGTACTTACATGTC
CTTGATTAAGGAAAGACGTGAGAAGAACGACATCCAAAACCGTGACTTGATTGATGAATTGATGAAGAACTCCACTTACAAGGATGGTACTAAGATGACCGACCAAGAAATT
GCCAACCTATTGATTGGTGTCTTGATGGGTGGTCAACATACTTCCGCTGCTACCTCCGCTTGGTGTCTATTGCATTTGGCTGAAAGACCAGATGTCCAAGAAGAATTGTACCAA

GAACAAATGCGCGTCTTGAACAACGATACCAAGGAATTGACTTACGATGACCTACAAAACATGCCTCTATTGAACCAAATGATCAAGGAAACTTTGAGATTGCACCACCCATTG
CACTCTTTGTTCCGTAAAGTCATGAGAGATGTCGCTATTCCAAACACTTCTTACGTTGTCCCAAGGGACTACCACGTTCTAGTCTCCCCAGGTTACACTCACTTGCAAGAAGAATT
CTTCCCTAAGCCAAATGAATTCAACATCCACCGTTGGGACGGTGATGCTGCTTCTTCCAGTGCTGCTGGTGGTGACGAAGTTGATTACGGTTTCGGTGCTATCTCCAAGGGTGT
TTCCTCTCCATACTTGCCATTCGGTGGTGGTAGACACAGATGTATCGGTGAATTGTTCGCTTACTGTCAATTGGGTGTGTTGATGTCCATTTTCATCAGAACCATGAAATGGCGT
TACCCAACTGAAGGTGAAACTGTCCCACCATCTGACTTCACCTCCATGGTCACCCTACCAACTGCCCCAGCTAAGATCTACTGGGAAAAGAGACATCCAGAACAAAAGTACTAG
-

>39

ATGTCCACTGAAAACACTTCTTTGGTCGTTGAACTATTGGAGTACGTGAAGCTTGGTCTTTCGTACTTCCAAGCTCTGCCATTGGCACAGAGAGTGTCTATTATGGTCGCCTTGC
CATTTGTGTACACCATCACATGGCAATTGCTTTACTCCTTGAGAAAGGACAGACCACCACTTGTGTTCTACTGGATTCCATGGGTCGGCTCTGCTATCCCATACGGTACCAAGCC
ATACGAGTTCTTCGAAGACTGCCAAAAGAAATACGGTGATATCTTCTCTTTCATGCTATTGGGTAGAATTATGACTGTCTACTTGGGTCCAAAGGGTCACGAATTCATCTTCAAC
GCCAAGTTGGCCGATGTTTCCGCTGAAGCTGCTTACTCCCACTTGACCACCCCAGTGTTCGGTAAAGGTGTTATCTACGATTGTCCAAACCACAGACTAATGGAACAAAAGAAG
TTTGTCAAGGGTGCTTTGACTAAGGAAGCCTTTGTCAGATACGTTCCATTGATCGCTGAGGAAATCTACAAGTACTTCAGAAACTCCAAGAACTTCAAGATCAACGAAAACAAC
TCCGGTATCGTCGACGTTATGGTCTCCCAACCTGAAATGACTATCTTCACTGCTTCCAGATCCTTGCTAGGTAAGGAAATGAGAGACAAGTTGGACACCGACTTCGCTTACTTGT
ACAGTGACTTGGACAAGGGTTTCACCCCAATTAACTTCGTCTTCCCTAACTTGCCTCTAGAACACTACAGAAAGAGAGACCATGCCCAACAAGCTATCTCTGGTACTTACATGTC
CTTGATTAAGGAAAGACGTGAGAAGAACGACATCCAAAACCGTGACTTGATTGATGAATTGATGAAGAACTCCACTTACAAGGATGGTACTAAGATGACCGACCAAGAAATT
GCCAACCTATTGATTGGTGTCTTGATGGGTGGTCAACATACTTCCGCTGCTACCTCCGCTTGGTGTCTATTGCATTTGGCTGAAAGACCAGATGTCCAAGAAGAATTGTACCAA
GAACAAATGCGCGTCTTGAACAACGATACCAAGGAATTGACTTACGATGACCTACAAAACATGCCTCTATTGAACCAAATGATCAAGGAAACTTTGAGATTGCACCACCCATTG
CACTCTTTGTTCCGTAAAGTCATGAGAGATGTCGCTATTCCAAACACTTCCTACGTTGTCCCAAGGGACTACCACGTTCTAGTCTCCCCAGGTTACACTCACTTGCAAGAAGAATT
CTTCCCTAAGCCAAATGAATTCAACATCCACCGTTGGGACGGTGATGCTGCTTCTTCCAGTGCTGCTGGTGGTGACGAAGTTGATTACGGTTTCGGTGCTATCTCCAAGGGTGT
TTCCTCTCCATACTTGCCATTCGGTGGTGGTAGACACAGATGTATCGGTGAATTGTTCGCTTACTGTCAATTGGGTGTGTTGATGTCCATTTTCATCAGAACCATGAAATGGCGT
TACCCAACTGAAGGTGAAACTGTCCCACCATCTGACTTCACCTCCATGGTCACCCTACCAACTGCCCCAGCTAAGATCTACTGGGAAAAGAGACATCCAGAACAAAAGTACTAG
-

>43

ATGTCCACTGAAAACACTTCTTTGGTCGTTGAACTATTGGAGTACGTGAAGCTTGGTCTTTCGTACTTCCAAGCTCTGCCATTGGCGCAGAGAGTGTCTATTATGGTCGCCTTGC
CATTTGTGTACACCATCACATGGCAATTGCTTTACTCCTTGAGAAAGGACAGACCACCACTTGTGTTCTACTGGATCCCATGGGTCGGCTCTGCTATCCCATACGGTACCAAGCC
ATACGAGTTCTTCGAAGACTGCCAAAAGAAATACGGTGATATCTTCTCTTTCATGCTATTGGGTAGAATTATGACTGTCTACTTGGGTCCAAAGGGTCACGAATTCATCTTCAAC
GCCAAGTTGGCCGATGTTTCCGCTGAAGCTGCTTACTCCCACTTGACCACCCCAGTGTTCGGTAAAGGTGTTATCTACGATTGTCCAAACCACAGACTAATGGAACAAAAGAAG
TTTGTCAAGGGTGCTTTGACTAAGGAAGCCTTTGTCAGATACGTTCCATTGATCGCTGAGGAAATCTACAAGTACTTCAGAAACTCCAAGAACTTCAAGATCAACGAAAACAAC
TCCGGTATCGTCGACGTTATGGTCTCCCAACCTGAAATGACTATCTTCACTGCTTCCAGATCCTTGCTAGGTAAGGAAATGAGAGACAAGTTGGACACCGACTTTGCTTACTTGT

ACAGTGACTTGGACAAGGGTTTCACCCCAATTAACTTCGTCTTCCCTAACTTGCCTCTAGAACACTACAGAAAGAGAGACCATGCCCAACAAGCTATCTCTGGTACTTACATGTC
CTTGATTAAGGAAAGACGTGAGAAGAACGACATCCAAAACCGTGACTTGATTGATGAATTGATGAAGAACTCCACTTACAAGGATGGTACTAAGATGACCGACCAAGAAATT
GCCAACCTATTGATTGGTGTCTTGATGGGTGGTCAACATACTTCCGCTGCTACCTCCGCTTGGTGTCTATTGCATTTGGCTGAAAGACCAGATGTCCAAGAAGAATTGTACCAA
GAACAAATGCGCGTCTTGAACAACGATACCAAGGAATTGACTTACGATGACCTACAAAACATGCCTCTATTGAACCAAATGATCAAGGAAACTTTGAGATTGCACCACCCATTG
CACTCTTTGTTCCGTAAAGTCATGAGAGATGTCGCTATTCCAAACACTTCCTACGTTGTCCCAAGGGACTACCACGTTCTAGTCTCCCCAGGTTACACTCACTTGCAAGAAGAATT
CTTCCCTAAGCCAAACGAATTCAACATCCACCGTTGGGACGGTGATGCTGCTTCTTCCAGTGCTGCTGGTGGTGACGAAGTTGATTACGGTTTCGGTGCTATCTCCAAGGGTGT
TTCCTCTCCATACTTGCCATTCGGTGGTGGTAGACACAGATGTATCGGTGAATTGTTCGCTTACTGTCAATTGGGTGTGTTGATGTCCATTTTCATCAGAACCATGAAATGGCGT
TACCCAACTGAAGGTGAAACTGTCCCACCATCTGACTTCACCTCCATGGTCACCCTACCAACTGCCCCAGCTAAGATCTACTGGGAAAAGAGACATCCAGAACAAAAGTACTAG
-

>45

ATGTCCACTGAAAACACTTCTTTGGTCGTTGAACTATTGGAGTACGTGAAGCTTGGTCTTTCGTACTTCCAAGCTCTGCCATTGGCGCAGAGAGTGTCTATTATGGTCGCCTTGC
CATTTGTGTACACCATCACATGGCAATTGCTTTACTCCTTGAGAAAGGACAGACCACCACTTGTGTTCTACTGGATCCCATGGGTCGGCTCTGCTATCCCATACGGTACCAAGCC
ATACGAGTTCTTCGAAGACTGCCAAAAGAAATACGGTGATATCTTCTCTTTCATGCTATTGGGTAGAATCATGACTGTCTACTTGGGTCCAAAGGGTCACGAATTCATCTTCAAC
GCCAAGTTGGCCGATGTTTCCGCTGAAGCTGCTTACTCCCACTTGACCACCCCAGTGTTCGGTAAAGGTGTTATCTACGATTGTCCAAACCACAGACTAATGGAACAAAAGAAG
TTTGTCAAGGGTGCTTTGACTAAGGAAGCCTTTGTCAGATACGTTCCATTGATCGCTGAGGAAATCTACAAGTACTTCAGAAACTCCAAGAACTTCAAGATCAACGAAAACAAC
TCCGGTATCGTCGACGTTATGGTCTCCCAACCTGAAATGACTATCTTCACTGCTTCCAGATCCTTGCTAGGTAAGGAAATGAGAGACAAGTTGGACACCGACTTCGCTTACTTGT
ACAGTGACTTGGACAAGGGTTTCACCCCAATTAACTTCGTCTTCCCTAACTTGCCTCTAGAACACTACAGAAAGAGAGACCATGCCCAACAAGCTATCTCTGGTACTTACATGTC
CTTGATTAAGGAAAGACGTGAGAAGAACGACATCCAAAACCGTGACTTGATTGATGAATTGATGAAGAACTCCACTTACAAGGATGGTACTAAGATGACCGACCAAGAAATT
GCCAACCTATTGATTGGTGTCTTGATGGGTGGTCAACATACTTCCGCTGCTACCTCCGCTTGGTGTCTATTGCATTTGGCTGAAAGACCAGATGTCCAAGAAGAATTGTACCAA
GAACAAATGCGCGTCTTGAACAACGATACCAAGGAATTGACTTACGATGACCTACAAAACATGCCTCTATTGAACCAAATGATCAAGGAAACTTTGAGATTGCACCACCCATTG
CACTCTTTGTTCCGTAAAGTCATGAGAGATGTCGCTATTCCAAACACTTCTTACGTTGTCCCAAGGGACTACCACGTTCTAGTCTCCCCAGGTTACACTCACTTGCAAGAAGAATT
CTTCCCTAAGCCAAATGAATTCAACATCCACCGTTGGGACGGTGATGCTGCTTCTTCCAGTGCTGCTGGTGGTGACGAAGTTGATTACGGTTTCGGTGCTATCTCCAAGGGTGT
TTCCTCTCCATACTTGCCATTCGGTGGTGGTAGACACAGATGTATCGGTGAATTGTTCGCTTACTGTCAATTGGGTGTGTTGATGTCCATTTTCATCAGAACCATGAAATGGCGT
TACCCAACTGAAGGTGAAACTGTCCCACCATCTGACTTCACCTCCATGGTCACCCTACCAACTGCCCCAGCTAAGATCTACTGGGAAAAGAGACATCCAGAACAAAAGTACTAG
-

>46

ATGTCCACTGAAAACACTTCTTTGGTCGTTGAACTATTGGAGTACGTGAAGCTTGGTCTTTCGTACTTCCAAGCTCTGCCATTGGCGCAGAGAGTGTCTATTATGGTCGCCTTGC
CATTTGTGTACACCATCACATGGCAATTGCTTTACTCCTTGAGAAAGGACAGACCACCACTTGTGTTCTACTGGATCCCATGGGTCGGCTCTGCTATCCCATACGGTACCAAGCC
ATACGAGTTCTTCGAAGACTGCCAAAAGAAATACGGTGATATCTTCTCTTTCATGCTATTGGGTAGAATCATGACTGTCTACTTGGGTCCAAAGGGTCACGAATTCATCTTCAAC

GCCAAGTTGGCCGATGTTTCCGCTGAAGCTGCTTACTCCCACTTGACCACCCCAGTGTTCGGTAAAGGTGTTATCTACGATTGTCCAAACCACAGACTAATGGAACAAAAGAAG
TTTGTCAAGGGTGCTTTGACTAAGGAAGCCTTTGTCAGATACGTTCCATTGATCGCTGAGGAAATCTACAAGTACTTCAGAAACTCCAAGAACTTCAAGATCAACGAAAACAAC
TCCGGTATCGTCGACGTTATGGTCTCCCAACCTGAAATGACTATCTTCACTGCTTCCAGATCCTTGCTAGGTAAGGAAATGAGAGACAAGTTGGACACCGACTTCGCTTACTTGT
ACAGTGACTTGGACAAGGGTTTCACCCCAATTAACTTCGTCTTCCCTAACTTGCCTCTAGAACACTACAGAAAGAGAGACCATGCCCAACAAGCTATCTCTGGTACTTACATGTC
CTTGATTAAGGAAAGACGTGAGAAGAACGACATCCAAAACCGTGACTTGATTGATGAATTGATGAAGAACTCCACTTACAAGGATGGTACTAAGATGACCGACCAAGAAATT
GCCAACCTATTGATTGGTGTCTTGATGGGTGGTCAACATACTTCCGCTGCTACCTCCGCTTGGTGTCTATTGCATTTGGCTGAAAGACCAGATGTCCAAGAAGAATTGTACCAA
GAACAAATGCGCGTCTTGAACAACGATACCAAGGAATTGACTTACGATGACCTACAAAACATGCCTCTATTGAACCAAATGATCAAGGAAACTTTGAGATTGCACCACCCATTG
CACTCTTTGTTCCGTAAAGTCATGAGAGATGTCGCTATTCCAAACACTTCTTACGTTGTCCCAAGGGACTACCACGTTCTAGTCTCCCCAGGTTACACTCACTTGCAAGAAGAATT
CTTCCCTAAGCCAAATGAATTCAACATCCMCCGTTGGGACGGTGATGCTGCTTCTTCCAGTGCTGCTGGTGGTGACGAAGTTGATTACGGTTTCGGTGCTATCTCCAAGGGTGT
TTCCTCTCCATACTTGCCATTCGGTGGTGGTAGACACAGATGTATCGGTGAATTGTTCGCTTACTGTCAATTGGGTGTGTTGATGTCCATTTTCATCAGAACCATGAAATGGCGT
TACCCAACTGAAGGTGAAACTGTCCCACCATCTGACTTCACCTCCATGGTCACCCTACCAACTGCCCCAGCTAAGATCTACTGGGAAAAGAGACATCCAGAACAAAAGTACTAG
-

>50

ATGTCCACTGAAAACACTTCTTTGGTCGTTGAACTATTGGAGTACGTGAAGCTTGGTCTTTCGTACTTCCAAGCTCTGCCATTGGCGCAGAGAGTGTCTATTATGGTCGCCTTGC
CATTTGTGTACACCATCACATGGCAATTGCTTTACTCCTTGAGAAAGGACAGACCACCACTTGTGTTCTACTGGATCCCATGGGTCGGCTCTGCTATCCCATACGGTACCAAGCC
ATACGAGTTCTTCGAAGACTGCCAAAAGAAATACGGTGATATCTTCTCTTTCATGCTATTGGGTAGAATCATGACTGTCTACTTGGGTCCAAAGGGTCACGAATTCATCTTCAAC
GCCAAGTTGGCCGATGTTTCCGCTGAAGCTGCTTACTCCCACTTGACCACCCCAGTGTTCGGTAAAGGTGTTATCTACGATTGTCCAAACCACAGACTAATGGAACAAAAGAAG
TTTGTCAAGGGTGCTTTGACTAAGGAAGCCTTTGTCAGATACGTTCCATTGATCGCTGAGGAAATCTACAAGTACTTCAGAAACTCCAAGAACTTCAAGATCAACGAAAACAAC
TCCGGTATCGTCGACGTTATGGTCTCCCAACCTGAAATGACTATCTTCACTGCTTCCAGATCCTTGCTAGGTAAGGAAATGAGAGACAAGTTGGACACCGACTTCGCTTACTTGT
ACAGTGACTTGGACAAGGGTTTCACCCCAATTAACTTCGTCTTCCCTAACTTGCCTCTAGAACACTACAGAAAGAGAGACCATGCCCAACAAGCTATCTCTGGTACTTACATGTC
CTTGATTAAGGAAAGACGTGAGAAGAACGACATCCAAAACCGTGACTTGATTGATGAATTGATGAAGAACTCCACTTACAAGGATGGTACTAAGATGACCGACCAAGAAATT
GCCAACCTATTGATTGGTGTCTTGATGGGTGGTCAACATACTTCCGCTGCTACCTCCGCTTGGTGTCTATTGCATTTGGCTGAAAGACCAGATGTCCAAGAAGAATTGTACCAA
GAACAAATGCGCGTCTTGAACAACGATACCAAGGAATTGACTTACGATGACCTACAAAACATGCCTCTATTGAACCAAATGATCAAGGAAACTTTGAGATTGCACCACCCATTG
CACTCTTTGTTCCGTAAAGTCATGAGAGATGTCGCTATTCCAAACACTTCTTACGTTGTCCCAAGGGACTACCACGTTCTAGTCTCCCCAGGTTACACTCACTTGCAAGAAGAATT
CTTCCCTAAGCCAAATGAATTCAACATCCACCGTTGGGACGGTGATGCTGCTTCTTCCAGTGCTGCTGGTGGTGACGAAGTTGATTACGGTTTCGGTGCTATCTCCAAGGGTGT
TTCCTCTCCATACTTGCCATTCGGTGGTGGTAGACACAGATGTATCGGTGAATTGTTCGCTTACTGTCAATTGGGTGTGTTGATGTCCATTTTCATCAGAACCATGAAATGGCGT
TACCCAACTGAAGGTGAAACTGTCCCACCATCTGACTTCACCTCCATGGTCACCCTACCAACTGCCCCAGCTAAGATCTACTGGGAAAAGAGACATCCAGAACAAAAGTACTAG
-

>51

ATGTCCACTGAAAACACTTCTTTGGTCGTTGAACTATTGGAGTACGTGAAGCTTGGTCTTTCGTACTTCCAAGCTCTGCCATTGGCGCAGAGAGTGTCTATTATGGTCGCCTTGC
CATTTGTGTACACCATCACATGGCAATTGCTTTACTCCTTGAGAAAGGACAGACCACCACTTGTGTTCTACTGGATCCCATGGGTCGGCTCTGCTATCCCATACGGTACCAAGCC
ATACGAGTTCTTCGAAGACTGCCAAAAGAAATACGGTGATATCTTCTCTTTCATGCTATTGGGTAGAATTATGACTGTCTACTTGGGTCCAAAGGGTCACGAATTCATCTTCAAC
GCCAAGTTGGCCGATGTTTCCGCTGAAGCTGCTTACTCCCACTTGACCACCCCGGTGTTCGGTAAAGGTGTTATCTACGATTGTCCAAACCACAGACTAATGGAACAAAAGAAG
TTTGTCAAGGGTGCTTTGACTAAGGAAGCCTTTGTCAGATACGTTCCATTGATCGCTGAGGAAATCTACAAGTACTTCAGAAACTCCAAGAACTTCAAGATCAACGAAAACAAC
TCCGGTATCGTCGACGTTATGGTCTCCCAACCTGAAATGACTATCTTCACTGCTTCCAGATCCTTGCTAGGTAAGGAAATGAGAGACAAGTTGGACACCGACTTCGCTTACTTGT
ACAGTGACTTGGACAAGGGTTTCACCCCAATTAACTTCGTCTTCCCTAACTTGCCTCTAGAACACTACAGAAAGAGAGACCATGCCCAACAAGCTATCTCTGGTACTTACATGTC
CTTGATTAAGGAAAGACGTGAGAAGAACGATATCCAAAACCGTGACTTGATTGATGAATTGATGAAGAACTCCACTTACAAGGATGGTACTAAGATGACCGACCAAGAAATTG
CCAACCTATTGATTGGTGTCTTGATGGGTGGTCAACATACTTCCGCTGCTACCTCCGCTTGGTGTCTATTGCATTTGGCTGAAAGACCAGATGTCCAAGAAGAATTGTACCAAG
AACAAATGCGCGTCTTGAACAACGATACCAAGGAATTGACTTACGATGACCTACAAAACATGCCTCTATTGAACCAAATGATCAAGGAAACTTTGAGATTGCACCACCCATTGC
ACTCTTTGTTCCGTAAAGTCATGAGAGATGTCGCTATTCCAAACACTTCCTACGTTGTCCCAAGGGACTACCACGTTCTAGTCTCCCCAGGTTACACTCACTTGCAAGAAGAATTC
TTCCCTAAGCCAAACGAATTCAACATCCACCGTTGGGACGGTGATGCTGCTTCTTCCAGTGCTGCTGGTGGTGACGAAGTTGATTACGGTTTCGGTGCTATCTCCAAGGGTGTT
TCCTCTCCATACTTGCCATTCGGTGGTGGTAGACACAGATGTATCGGTGAATTGTTCGCTTACTGTCAATTGGGTGTGTTGATGTCCATTTTCATCAGAACCATGAAATGGCGTT
ACCCAACTGAAGGTGAAACTGTCCCACCATCTGACTTCACCTCCATGGTCACCCTACCAACTGCCCCAGCTAAGATCTACTGGGAAAAGAGACATCCAGAACAAAAGTACTAG-

>54

ATGTCCACTGAAAACACTTCTTTGGTCGTTGAACTATTGGAGTACGTGAAGCTTGGTCTTTCGTACTTCCAAGCTCTGCCATTGGCGCAGAGAGTGTCTATTATGGTCGCCTTGC
CATTTGTGTACACCATCACATGGCAATTGCTTTACTCCTTGAGAAAGGACAGACCACCACTTGTGTTCTACTGGATCCCATGGGTCGGCTCTGCTATCCCATACGGTACCAAGCC
ATACGAGTTCTTCGAAGACTGCCAAAAGAAATACGGTGATATCTTCTCTTTCATGCTATTGGGTAGAATCATGACTGTCTACTTGGGTCCAAAGGGTCACGAATTCATCTTCAAC
GCCAAGTTGGCCGATGTTTCCGCTGAAGCTGCTTACTCCCACTTGACCACCCCAGTGTTCGGTAAAGGTGTTATCTACGATTGTCCAAACCACAGACTAATGGAACAAAAGAAG
TTTGTCAAGGGTGCTTTGACTAAGGAAGCCTTTGTCAGATACGTTCCATTGATCGCTGAGGAAATCTACAAGTACTTCAGAAACTCCAAGAACTTCAAGATCAACGAAAACAAC
TCCGGTATCGTCGACGTTATGGTCTCCCAACCTGAAATGACTATCTTCACTGCTTCCAGATCCTTGCTAGGTAAGGAAATGAGAGACAAGTTGGACACCGACTTCGCTTACTTGT
ACAGTGACTTGGACAAGGGTTTCACCCCAATTAACTTCGTCTTCCCTAACTTGCCTCTAGAACACTACAGAAAGAGAGACCATGCCCAACAAGCTATCTCTGGTACTTACATGTC
CTTGATTAAGGAAAGACGTGAGAAGAACGACATCCAAAACCGTGACTTGATTGATGAATTGATGAAGAACTCCACTTACAAGGATGGTACTAAGATGACCGACCAAGAAATT
GCCAACCTATTGATTGGTGTCTTGATGGGTGGTCAACATACTTCCGCTGCTACCTCCGCTTGGTGTCTATTGCATTTGGCTGAAAGACCAGATGTCCAAGAAGAATTGTACCAA
GAACAAATGCGCGTCTTGAACAACGATACCAAGGAATTGACTTACGATGACCTACAAAACATGCCTCTATTGAACCAAATGATCAAGGAAACTTTGAGATTGCACCACCCATTG
CACTCTTTGTTCCGTAAAGTCATGAGAGATGTCGCTATTCCAAACACTTCTTACGTTGTCCCAAGGGACTACCACGTTCTAGTCTCCCCAGGTTACACTCACTTGCAAGAAGAATT
CTTCCCTAAGCCAAATGAATTCAACATCCACCGTTGGGACGGTGATGCTGCTTCTTCCAGTGCTGCTGGTGGTGACGAAGTTGATTACGGTTTCGGTGCTATCTCCAAGGGTGT
TTCCTCTCCATACTTGCCATTCGGTGGTGGTAGACACAGATGTATCGGTGAATTGTTCGCTTACTGTCAATTGGGTGTGTTGATGTCCATTTTCATCAGAACCATGAAATGGCGT
TACCCAACTGAAGGTGAAACTGTCCCACCATCTGACTTCACCTCCATGGTCACCCTACCAACTGCCCCAGCTAAGATCTACTGGGAAAAGAGACATCCAGAACAAAAGTACTAG
-

>57

ATGTCCACTGAAAACACTTCTTTGGTCGTTGAACTATTGGAGTACGTGAAGCTTGGTCTTTCGTACTTCCAAGCTCTGCCATTGGCGCAGAGAGTGTCTATTATGGTCGCCTTGC
CATTTGTGTACACCATCACATGGCAATTGCTTTACTCCTTGAGAAAGGACAGACCACCACTTGTGTTCTACTGGATCCCATGGGTCGGCTCTGCTATCCCATACGGTACCAAGCC
ATACGAGTTCTTCGAAGACTGCCAAAAGAAATACGGTGATATCTTCTCTTTCATGCTATTGGGTAGAATCATGACTGTCTACTTGGGTCCAAAGGGTCACGAATTCATCTTCAAC
GCCAAGTTGGCCGATGTTTCCGCTGAAGCTGCTTACTCCCACTTGACCACCCCAGTGTTCGGTAAAGGTGTTATCTACGATTGTCCAAACCACAGACTAATGGAACAAAAGAAG
TTTGTCAAGGGTGCTTTGACTAAGGAAGCCTTTGTCAGATACGTTCCATTGATCGCTGAGGAAATCTACAAGTACTTCAGAAACTCCAAGAACTTCAAGATCAACGAAAACAAC
TCCGGTATCGTCGACGTTATGGTCTCCCAACCTGAAATGACTATCTTCACTGCTTCCAGATCCTTGCTAGGTAAGGAAATGAGAGACAAGTTGGACACCGACTTCGCTTACTTGT
ACAGTGACTTGGACAAGGGTTTCACCCCAATTAACTTCGTCTTCCCTAACTTGCCTCTAGAACACTACAGAAAGAGAGACCATGCCCAACAAGCTATCTCTGGTACTTACATGTC
CTTGATTAAGGAAAGACGTGAGAAGAACGACATCCAAAACCGTGACTTGATTGATGAATTGATGAAGAACTCCACTTACAAGGATGGTACTAAGATGACCGACCAAGAAATT
GCCAACCTATTGATTGGTGTCTTGATGGGTGGTCAACATACTTCCGCTGCTACCTCCGCTTGGTGTCTATTGCATTTGGCTGAAAGACCAGATGTCCAAGAAGAATTGTACCAA
GAACAAATGCGCGTCTTGAACAACGATACCAAGGAATTGACTTACGATGACCTACAAAACATGCCTCTATTGAACCAAATGATCAAGGAAACTTTGAGATTGCACCACCCATTG
CACTCTTTGTTCCGTAAAGTCATGAGAGATGTCGCTATTCCAAACACTTCTTACGTTGTCCCAAGGGACTACCACGTTCTAGTCTCCCCAGGTTACACTCACTTGCAAGAAGAATT
CTTCCCTAAGCCAAATGAATTCAACATCCMCCGTTGGGACGGTGATGCTGCTTCTTCCAGTGCTGCTGGTGGTGACGAAGTTGATTACGGTTTCGGTGCTATCTCCAAGGGTGT
TTCCTCTCCATACTTGCCATTCGGTGGTGGTAGACACAGATGTATCGGTGAATTGTTCGCTTACTGTCAATTGGGTGTGTTGATGTCCATTTTCATCAGAACCATGAAATGGCGT
TACCCAACTGAAGGTGAAACTGTCCCACCATCTGACTTCACCTCCATGGTCACCCTACCAACTGCCCCAGCTAAGATCTACTGGGAAAAGAGACATCCAGAACAAAAGTACTAG
-

>58

ATGTCCACTGAAAACACTTCTTTGGTCGTTGAACTATTGGAGTACGTGAAGCTTGGTCTTTCGTACTTCCAAGCTCTGCCATTGGCGCAGAGAGTGTCTATTATGGTCGCCTTGC
CATTTGTGTACACCATCACATGGCAATTGCTTTACTCCTTGAGAAAGGACAGACCACCACTTGTGTTCTACTGGATCCCATGGGTCGGCTCTGCTATCCCATACGGTACCAAGCC
ATACGAGTTCTTCGAAGACTGCCAAAAGAAATACGGTGATATCTTCTCTTTCATGCTATTGGGTAGAATCATGACTGTCTACTTGGGTCCAAAGGGTCACGAATTCATCTTCAAC
GCCAAGTTGGCCGATGTTTCCGCTGAAGCTGCTTACTCCCACTTGACCACCCCAGTGTTCGGTAAAGGTGTTATCTACGATTGTCCAAACCACAGACTAATGGAACAAAAGAAG
TTTGTCAAGGGTGCTTTGACTAAGGAAGCCTTTGTCAGATACGTTCCATTGATCGCTGAGGAAATCTACAAGTACTTCAGAAACTCCAAGAACTTCAAGATCAACGAAAACAAC
TCCGGTATCGTCGACGTTATGGTCTCCCAACCTGAAATGACTATCTTCACTGCTTCCAGATCCTTGCTAGGTAAGGAAATGAGAGACAAGTTGGACACCGACTTCGCTTACTTGT
ACAGTGACTTGGACAAGGGTTTCACCCCAATTAACTTCGTCTTCCCTAACTTGCCTCTAGAACACTACAGAAAGAGAGACCATGCCCAACAAGCTATCTCTGGTACTTACATGTC
CTTGATTAAGGAAAGACGTGAGAAGAACGACATCCAAAACCGTGACTTGATTGATGAATTGATGAAGAACTCCACTTACAAGGATGGTACTAAGATGACCGACCAAGAAATT
GCCAACCTATTGATTGGTGTCTTGATGGGTGGTCAACATACTTCCGCTGCTACCTCCGCTTGGTGTCTATTGCATTTGGCTGAAAGACCAGATGTCCAAGAAGAATTGTACCAA
GAACAAATGCGCGTCTTGAACAACGATACCAAGGAATTGACTTACGATGACCTACAAAACATGCCTCTATTGAACCAAATGATCAAGGAAACTTTGAGATTGCACCACCCATTG
CACTCTTTGTTCCGTAAAGTCATGAGAGATGTCGCTATTCCAAACACTTCTTACGTTGTCCCAAGGGACTACCACGTTCTAGTCTCCCCAGGTTACACTCACTTGCAAGAAGAATT
CTTCCCTAAGCCAAATGAATTCAACATCCACCGTTGGGACGGTGATGCTGCTTCTTCCAGTGCTGCTGGTGGTGACGAAGTTGATTACGGTTTCGGTGCTATCTCCAAGGGTGT

TTCCTCTCCATACTTGCCATTCGGTGGTGGTAGACACAGATGTATCGGTGAATTGTTCGCTTACTGTCAATTGGGTGTGTTGATGTCCATTTTCATCAGAACCATGAAATGGCGT
TACCCAACTGAAGGTGAAACTGTCCCACCATCTGACTTCACCTCCATGGTCACCCTACCAACTGCCCCAGCTAAGATCTACTGGGAAAAGAGACATCCAGAACAAAAGTACTAG
-

>71

ATGTCCACTGAAAACACTTCTTTGGTCGTTGAACTATTGGAGTACGTGAAGCTTGGTCTTTCGTACTTCCAAGCTCTGCCATTGGCGCAGAGAGTGTCTATTATGGTCGCCTTGC
CATTTGTGTACACCATCACATGGCAATTGCTTTACTCCTTGAGAAAGGACAGACCACCACTTGTGTTCTACTGGATCCCATGGGTCGGCTCTGCTATCCCATACGGTACCAAGCC
ATACGAGTTCTTCGAAGACTGCCAAAAGAAATACGGTGATATCTTCTCTTTCATGCTATTGGGTAGAATTATGACTGTCTACTTGGGTCCAAAGGGTCACGAATTCATCTTCAAC
GCCAAGTTGGCCGATGTTTCCGCTGAAGCTGCTTACTCCCACTTGACCACCCCGGTGTTCGGTAAAGGTGTTATCTACGATTGTCCAAACCACAGACTAATGGAACAAAAGAAG
TTTGTCAAGGGTGCTTTGACTAAGGAAGCCTTTGTCAGATACGTTCCATTGATCGCTGAGGAAATCTACAAGTACTTCAGAAACTCCAAGAACTTCAAGATCAACGAAAACAAC
TCCGGTATCGTCGACGTTATGGTCTCCCAACCTGAAATGACTATCTTCACTGCTTCCAGATCCTTGCTAGGTAAGGAAATGAGAGACAAGTTGGACACCGACTTCGCTTACTTGT
ACAGTGACTTGGACAAGGGTTTCACCCCAATTAACTTCGTCTTCCCTAACTTGCCTCTAGAACACTACAGAAAGAGAGACCATGCCCAACAAGCTATCTCTGGTACTTACATGTC
CTTGATTAAGGAAAGACGTGAGAAGAACGATATCCAAAACCGTGACTTGATTGATGAATTGATGAAGAACTCCACTTACAAGGATGGTACTAAGATGACCGACCAAGAAATTG
CCAACCTATTGATTGGTGTCTTGATGGGTGGTCAACATACTTCCGCTGCTACCTCCGCTTGGTGTCTATTGCATTTGGCTGAAAGACCAGATGTCCAAGAAGAATTGTACCAAG
AACAAATGCGCGTCTTGAACAACGATACCAAGGAATTGACTTACGATGACCTACAAAACATGCCTCTATTGAACCAAATGATCAAGGAAACTTTGAGATTGCACCACCCATTGC
ACTCTTTGTTCCGTAAAGTCATGAGAGATGTCGCTATTCCAAACACTTCCTACGTTGTCCCAAGGGACTACCACGTTCTAGTCTCCCCAGGTTACACTCACTTGCAAGAAGAATTC
TTCCCTAAGCCAAACGAATTCAACATCCMCCGTTGGGACGGTGATGCTGCTTCTTCCAGTGCTGCTGGTGGTGACGAAGTTGATTACGGTTTCGGTGCTATCTCCAAGGGTGTT
TCCTCTCCATACTTGCCATTCGGTGGTGGTAGACACAGATGTATCGGTGAATTGTTCGCTTACTGTCAATTGGGTGTGTTGATGTCCATTTTCATCAGAACCATGAAATGGCGTT
ACCCAACTGAAGGTGAAACTGTCCCACCATCTGACTTCACCTCCATGGTCACCCTACCAACTGCCCCAGCTAAGATCTACTGGGAAAAGAGACATCCAGAACAAAAGTACTAG-

>72

ATGTCCACTGAAAACACTTCTTTGGTCGTTGAACTATTGGAGTACGTGAAGCTTGGTCTTTCGTACTTCCAAGCTCTGCCATTGGCGCAGAGAGTGTCTATTATGGTCGCCTTGC
CATTTGTGTACACCATCACATGGCAATTGCTTTACTCCTTGAGAAAGGACAGACCACCACTTGTGTTCTACTGGATCCCATGGGTCGGCTCTGCTATCCCATACGGTACCAAGCC
ATACGAGTTCTTCGAAGACTGCCAAAAGAAATACGGTGATATCTTCTCTTTCATGCTATTGGGTAGAATTATGACTGTCTACTTGGGTCCAAAGGGTCACGAATTCATCTTCAAC
GCCAAGTTGGCCGATGTTTCCGCTGAAGCTGCTTACTCCCACTTGACCACCCCAGTGTTCGGTAAAGGTGTTATCTACGATTGTCCAAACCACAGACTAATGGAACAAAAGAAG
TTTGTCAAGGGTGCTTTGACTAAGGAAGCCTTTGTCAGATACGTTCCATTGATCGCTGAGGAAATCTACAAGTACTTCAGAAACTCCAAGAACTTCAAGATCAACGAAAACAAC
TCCGGTATCGTCGACGTTATGGTCTCCCAACCTGAAATGACTATCTTCACTGCTTCCAGATCCTTGCTAGGTAAGGAAATGAGAGACAAGTTGGACACCGACTTCGCTTACTTGT
ACAGTGACTTGGACAAGGGTTTCACCCCAATTAACTTCGTCTTCCCTAACTTGCCTCTAGAACACTACAGAAAGAGAGACCATGCCCAACAAGCTATCTCTGGTACTTACATGTC
CTTGATTAAGGAAAGACGTGAGAAGAACGACATCCAAAACCGTGACTTGATTGATGAATTGATGAAGAACTCCACTTACAAGGATGGTACTAAGATGACCGACCAAGAAATT
GCCAACCTATTGATTGGTGTCTTGATGGGTGGTCAACATACTTCCGCTGCTACCTCCGCTTGGTGTCTATTGCATTTGGCTGAAAGACCAGATGTCCAAGAAGAATTGTACCAA
GAACAAATGCGCGTCTTGAACAACGATACCAAGGAATTGACTTACGATGACCTACAAAACATGCCTCTATTGAACCAAATGATCAAGGAAACTTTGAGATTGCACCACCCATTG

CACTCTTTGTTCCGTAAAGTCATGAGAGATGTCGCTATTCCAAACACTTCCTACGTTGTCCCAAGGGACTACCACGTTCTAGTCTCCCCAGGTTACACTCACTTGCAAGAAGAATT
CTTCCCTAAGCCAAATGAATTCAACATCCMCCGTTGGGACGGTGATGCTGCTTCTTCCAGTGCTGCTGGTGGTGACGAAGTTGATTACGGTTTCGGTGCTATCTCCAAGGGTGT
TTCCTCTCCATACTTGCCATTCGGTGGTGGTAGACACAGATGTATCGGTGAATTGTTCGCTTACTGTCAATTGGGTGTGTTGATGTCCATTTTCATCAGAACCATGAAATGGCGT
TACCCAACTGAAGGTGAAACTGTCCCACCATCTGACTTCACCTCCATGGTCACCCTACCAACTGCCCCAGCTAAGATCTACTGGGAAAAGAGACATCCAGAACAAAAGTACTAG
-

>73

ATGTCCACTGAAAACACTTCTTTGGTCGTTGAACTATTGGAGTACGTGAAGCTTGGTCTTTCGTACTTCCAAGCTCTGCCATTGGCGCAGAGAGTGTCTATTATGGTCGCCTTGC
CATTTGTGTACACCATCACATGGCAATTGCTTTACTCCTTGAGAAAGGACAGACCACCACTTGTGTTCTACTGGATCCCATGGGTCGGCTCTGCTATCCCATACGGTACCAAGCC
ATACGAGTTCTTCGAAGACTGCCAAAAGAAATACGGTGATATCTTCTCTTTCATGCTATTGGGTAGAATTATGACTGTCTACTTGGGTCCAAAGGGTCACGAATTCATCTTCAAC
GCCAAGTTGGCCGATGTTTCCGCTGAAGCTGCTTACTCCCACTTGACCACCCCAGTGTTCGGTAAAGGTGTTATCTACGATTGTCCAAACCACAGACTAATGGAACAAAAGAAG
TTTGTCAAGGGTGCTTTGACTAAGGAAGCCTTTGTCAGATACGTTCCATTGATCGCTGAGGAAATCTACAAGTACTTCAGAAACTCCAAGAACTTCAAGATCAACGAAAACAAC
TCCGGTATCGTCGACGTTATGGTCTCCCAACCTGAAATGACTATCTTCACTGCTTCCAGATCCTTGCTAGGTAAGGAAATGAGAGACAAGTTGGACACCGACTTCGCTTACTTGT
ACAGTGACTTGGACAAGGGTTTCACCCCAATTAACTTCGTCTTCCCTAACTTGCCTCTAGAACACTACAGAAAGAGAGACCATGCCCAACAAGCTATCTCTGGTACTTACATGTC
CTTGATTAAGGAAAGACGTGAGAAGAACGACATCCAAAACCGTGACTTGATTGATGAATTGATGAAGAACTCCACTTACAAGGATGGTACTAAGATGACCGACCAAGAAATT
GCCAACCTATTGATTGGTGTCTTGATGGGTGGTCAACATACTTCCGCTGCTACCTCCGCTTGGTGTCTATTGCATTTGGCTGAAAGACCAGATGTCCAAGAAGAATTGTACCAA
GAACAAATGCGCGTCTTGAACAACGATACCAAGGAATTGACTTACGATGACCTACAAAACATGCCTCTATTGAACCAAATGATCAAGGAAACTTTGAGATTGCACCACCCATTG
CACTCTTTGTTCCGTAAAGTCATGAGAGATGTCGCTATTCCAAACACTTCCTACGTTGTCCCAAGGGACTACCACGTTCTAGTCTCCCCAGGTTACACTCACTTGCAAGAAGAATT
CTTCCCTAAGCCAAATGAATTCAACATCCACCGTTGGGACGGTGATGCTGCTTCTTCCAGTGCTGCTGGTGGTGACGAAGTTGATTACGGTTTCGGTGCTATCTCCAAGGGTGT
TTCCTCTCCATACTTGCCATTCGGTGGTGGTAGACACAGATGTATCGGTGAATTGTTCGCTTACTGTCAATTGGGTGTGTTGATGTCCATTTTCATCAGAACCATGAAATGGCGT
TACCCAACTGAAGGTGAAACTGTCCCACCATCTGACTTCACCTCCATGGTCACCCTACCAACTGCCCCAGCTAAGATCTACTGGGAAAAGAGACATCCAGAACAAAAGTACTAG
-

>74

ATGTCCACTGAAAACACTTCTTTGGTCGTTGAACTATTGGAGTACGTGAAGCTTGGTCTTTCGTACTTCCAAGCTCTGCCATTGGCACAGAGAGTGTCTATTATGGTCGCCTTGC
CATTTGTGTACACCATCACATGGCAATTGCTTTACTCCTTGAGAAAGGACAGACCACCACTTGTGTTCTACTGGATTCCATGGGTCGGCTCTGCTATCCCATACGGTACCAAGCC
ATACGAGTTCTTCGAAGACTGCCAAAAGAAATACGGTGATATCTTCTCTTTCATGCTATTGGGTAGAATTATGACTGTCTACTTGGGTCCAAAGGGTCACGAATTCATCTTCAAC
GCCAAGTTGGCCGATGTTTCCGCTGAAGCTGCTTACTCCCACTTGACCACCCCAGTGTTCGGTAAAGGTGTTATCTACGATTGTCCAAACCACAGACTAATGGAACAAAAGAAG
TTTGTCAAGGGTGCTTTGACTAAGGAAGCCTTTGTCAGATACGTTCCATTGATCGCTGAGGAAATCTACAAGTACTTCAGAAACTCCAAGAACTTCAAGATCAACGAAAACAAC
TCCGGTATCGTCGACGTTATGGTCTCCCAACCTGAAATGACTATCTTCACTGCTTCCAGATCCTTGCTAGGTAAGGAAATGAGAGACAAGTTGGACACCGACTTCGCTTACTTGT
ACAGTGACTTGGACAAGGGTTTCACCCCAATTAACTTCGTCTTCCCTAACTTGCCTCTAGAACACTACAGAAAGAGAGACCATGCCCAACAAGCTATCTCTGGTACTTACATGTC

CTTGATTAAGGAAAGACGTGAGAAGAACGACATCCAAAACCGTGACTTGATTGATGAATTGATGAAGAACTCCACTTACAAGGATGGTACTAAGATGACCGACCAAGAAATT
GCCAACCTATTGATTGGTGTCTTGATGGGTGGTCAACATACTTCCGCTGCTACCTCCGCTTGGTGTCTATTGCATTTGGCTGAAAGACCAGATGTCCAAGAAGAATTGTACCAA
GAACAAATGCGCGTCTTGAACAACGATACCAAGGAATTGACTTACGATGACCTACAAAACATGCCTCTATTGAACCAAATGATCAAGGAAACTTTGAGATTGCACCACCCATTG
CACTCTTTGTTCCGTAAAGTCATGAGAGATGTCGCTATTCCAAACACTTCCTACGTTGTCCCAAGGGACTACCACGTTCTAGTCTCCCCAGGTTACACTCACTTGCAAGAAGAATT
CTTCCCTAAGCCAAATGAATTCAACATCCACCGTTGGGACGGTGATGCTGCTTCTTCCAGTGCTGCTGGTGGTGACGAAGTTGATTACGGTTTCGGTGCTATCTCCAAGGGTGT
TTCCTCTCCATACTTGCCATTCGGTGGTGGTAGACACAGATGTATCGGTGAATTGTTCGCTTACTGTCAATTGGGTGTGTTGATGTCCATTTTCATCAGAACCATGAAATGGCGT
TACCCAACTGAAGGTGAAACTGTCCCACCATCTGACTTCACCTCCATGGTCACCCTACCAACTGCCCCAGCTAAGATCTACTGGGAAAAGAGACATCCAGAACAAAAGTACTAG
-

>75

ATGTCCACTGAAAACACTTCTTTGGTCGTTGAACTATTGGAGTACGTGAAGCTTGGTCTTTCGTACTTCCAAGCTCTGCCATTGGCGCAGAGAGTGTCTATTATGGTCGCCTTGC
CATTTGTGTACACCATCACATGGCAATTGCTTTACTCCTTGAGAAAGGACAGACCACCACTTGTGTTCTACTGGATCCCATGGGTCGGCTCTGCTATCCCATACGGTACCAAGCC
ATACGAGTTCTTCGAAGACTGCCAAAAGAAATACGGTGATATCTTCTCTTTCATGCTATTGGGTAGAATTATGACTGTCTACTTGGGTCCAAAGGGTCACGAATTCATCTTCAAC
GCCAAGTTGGCCGATGTTTCCGCTGAAGCTGCTTACTCCCACTTGACCACCCCAGTGTTCGGTAAAGGTGTTATCTACGATTGTCCAAACCACAGACTAATGGAACAAAAGAAG
TTTGTCAAGGGTGCTTTGACTAAGGAAGCCTTTGTCAGATACGTTCCATTGATCGCTGAGGAAATCTACAAGTACTTCAGAAACTCCAAGAACTTCAAGATCAACGAAAACAAC
TCCGGTATCGTCGACGTTATGGTCTCCCAACCTGAAATGACTATCTTCACTGCTTCCAGATCCTTGCTAGGTAAGGAAATGAGAGACAAGTTGGACACCGACTTCGCTTACTTGT
ACAGTGACTTGGACAAGGGTTTCACCCCAATTAACTTCGTCTTCCCTAACTTGCCTCTAGAACACTACAGAAAGAGAGACCATGCCCAACAAGCTATCTCTGGTACTTACATGTC
CTTGATTAAGGAAAGACGTGAGAAGAACGACATCCAAAACCGTGACTTGATTGATGAATTGATGAAGAACTCCACTTACAAGGATGGTACTAAGATGACCGACCAAGAAATT
GCCAACCTATTGATTGGTGTCTTGATGGGTGGTCAACATACTTCCGCTGCTACCTCCGCTTGGTGTCTATTGCATTTGGCTGAAAGACCAGATGTCCAAGAAGAATTGTACCAA
GAACAAATGCGCGTCTTGAACAACGATACCAAGGAATTGACTTACGATGACCTACAAAACATGCCTCTATTGAACCAAATGATCAAGGAAACTTTGAGATTGCACCACCCATTG
CACTCTTTGTTCCGTAAAGTCATGAGAGATGTCGCTATTCCAAACACTTCCTACGTTGTCCCAAGGGACTACCACGTTCTAGTCTCCCCAGGTTACACTCACTTGCAAGAAGAATT
CTTCCCTAAGCCAAATGAATTCAACATCCMCCGTTGGGACGGTGATGCTGCTTCTTCCAGTGCTGCTGGTGGTGACGAAGTTGATTACGGTTTCGGTGCTATCTCCAAGGGTGT
TTCCTCTCCATACTTGCCATTCGGTGGTGGTAGACACAGATGTATCGGTGAATTGTTCGCTTACTGTCAATTGGGTGTGTTGATGTCCATTTTCATCAGAACCATGAAATGGCGT
TACCCAACTGAAGGTGAAACTGTCCCACCATCTGACTTCACCTCCATGGTCACCCTACCAACTGCCCCAGCTAAGATCTACTGGGAAAAGAGACATCCAGAACAAAAGTACTAG
-

>76

ATGTCCACTGAAAACACTTCTTTGGTCGTTGAACTATTGGAGTACGTGAAGCTTGGTCTTTCGTACTTCCAAGCTCTGCCATTGGCGCAGAGAGTGTCTATTATGGTCGCCTTGC
CATTTGTGTACACCATCACATGGCAATTGCTTTACTCCTTGAGAAAGGACAGACCACCACTTGTGTTCTACTGGATTCCATGGGTCGGCTCTGCTATCCCATACGGTACCAAGCC
ATACGAGTTCTTCGAAGACTGCCAAAAGAAATACGGTGATATCTTCTCTTTCATGCTATTGGGTAGAATTATGACTGTCTACTTGGGTCCAAAGGGTCACGAATTCATCTTCAAC
GCCAAGTTGGCCGATGTTTCCGCTGAAGCTGCTTACTCCCACTTGACCACCCCAGTGTTCGGTAAAGGTGTTATCTACGATTGTCCAAACCACAGACTAATGGAACAAAAGAAG

TTTGTCAAGGGTGCTTTGACTAAGGAAGCCTTTGTCAGATACGTTCCATTGATCGCTGAGGAAATCTACAAGTACTTCAGAAACTCCAAGAACTTCAAGATCAACGAAAACAAC
TCCGGTATCGTCGACGTTATGGTCTCCCAACCTGAAATGACTATCTTCACTGCTTCCAGATCCTTGCTAGGTAAGGAAATGAGAGACAAGTTGGACACCGACTTCGCTTACTTGT
ACAGTGACTTGGACAAGGGTTTCACCCCAATTAACTTCGTCTTCCCTAACTTGCCTCTAGAACACTACAGAAAGAGAGACCATGCCCAACAAGCTATCTCTGGTACTTACATGTC
CTTGATTAAGGAAAGACGTGAGAAGAACGATATCCAAAACCGTGATTTGATTGATGAATTGATGAAGAACTCCACTTACAAGGATGGTACTAAGATGACCGACCAAGAAATTG
CCAACCTATTGATTGGTGTCTTGATGGGTGGTCAACATACTTCCGCTGCTACCTCCGCTTGGTGTCTATTGCATTTGGCTGAAAGACCAGATGTCCAAGAAGAATTGTACCAAG
AACAAATGCGCGTCTTGAACAACGATACCAAGGAATTGACTTACGATGACCTACAAAACATGCCTCTATTGAACCAAATGATCAAGGAAACTTTGAGATTGCACCACCCATTGC
ACTCTTTGTTCCGTAAAGTCATGAGAGATGTCGCTATTCCAAACACTTCCTACGTTGTCCCAAGGGACTACCACGTTCTAGTCTCCCCAGGTTACACTCACTTGCAAGAAGAATTC
TTCCCTAAGCCAAACGAATTCAACATCCMCCGTTGGGACGGTGATGCTGCTTCTTCCAGTGCTGCTGGTGGTGACGAAGTTGATTACGGTTTCGGTGCTATCTCCAAGGGTGTT
TCCTCTCCATACTTGCCATTCGGTGGTGGTAGACACAGATGTATCGGTGAATTGTTCGCTTACTGTCAATTGGGTGTGTTGATGTCCATTTTCATCAGAACCATGAAATGGCGTT
ACCCAACTGAAGGTGAAACTGTCCCACCATCTGACTTCACCTCCATGGTCACCCTACCAACTGCCCCAGCTAAGATCTACTGGGAAAAGAGACATCCAGAACAAAAGTACTAG-

>77

ATGTCCACTGAAAACACTTCTTTGGTCGTTGAACTATTGGAGTACGTGAAGCTTGGTCTTTCGTACTTCCAAGCTCTGCCATTGGCGCAGAGAGTGTCTATTATGGTCGCCTTGC
CATTTGTGTACACCATCACATGGCAATTGCTTTACTCCTTGAGAAAGGACAGACCACCACTTGTGTTCTACTGGATCCCATGGGTCGGCTCTGCTATCCCATACGGTACCAAGCC
ATACGAGTTCTTCGAAGACTGCCAAAAGAAATACGGTGATATCTTCTCTTTCATGCTATTGGGTAGAATTATGACTGTCTACTTGGGTCCAAAGGGTCACGAATTCATCTTCAAC
GCCAAGTTGGCCGATGTTTCCGCTGAAGCTGCTTACTCCCACTTGACCACCCCAGTGTTCGGTAAAGGTGTTATCTACGATTGTCCAAACCACAGACTAATGGAACAAAAGAAG
TTTGTCAAGGGTGCTTTGACTAAGGAAGCCTTTGTCAGATACGTTCCATTGATCGCTGAGGAAATCTACAAGTACTTCAGAAACTCCAAGAACTTCAAGATCAACGAAAACAAC
TCCGGTATCGTCGACGTTATGGTCTCCCAACCTGAAATGACTATCTTCACTGCTTCCAGATCCTTGCTAGGTAAGGAAATGAGAGACAAGTTGGACACCGACTTCGCTTACTTGT
ACAGTGACTTGGACAAGGGTTTCACCCCAATTAACTTCGTCTTCCCTAACTTGCCTCTAGAACACTACAGAAAGAGAGACCATGCCCAACAAGCTATCTCTGGTACTTACATGTC
CTTGATTAAGGAAAGACGTGAGAAGAACGACATCCAAAACCGTGACTTGATTGATGAATTGATGAAGAACTCCACTTACAAGGATGGTACTAAGATGACCGACCAAGAAATT
GCCAACCTATTGATTGGTGTCTTGATGGGTGGTCAACATACTTCCGCTGCTACCTCCGCTTGGTGTCTATTGCATTTGGCTGAAAGACCAGATGTCCAAGAAGAATTGTACCAA
GAACAAATGCGCGTCTTGAACAACGATACCAAGGAATTGACTTACGATGACCTACAAAACATGCCTCTATTGAACCAAATGATCAAGGAAACTTTGAGATTGCACCACCCATTG
CACTCTTTGTTCCGTAAAGTCATGAGAGATGTCGCTATTCCAAACACTTCCTACGTTGTCCCAAGGGACTACCACGTTCTAGTCTCCCCAGGTTACACTCACTTGCAAGAAGAATT
CTTCCCTAAGCCAAATGAATTCAACATCCMCCGTTGGGACGGTGATGCTGCTTCTTCCAGTGCTGCTGGTGGTGACGAAGTTGATTACGGTTTCGGTGCTATCTCCAAGGGTGT
TTCCTCTCCATACTTGCCATTCGGTGGTGGTAGACACAGATGTATCGGTGAATTGTTCGCTTACTGTCAATTGGGTGTGTTGATGTCCATTTTCATCAGAACCATGAAATGGCGT
TACCCAACTGAAGGTGAAACTGTCCCACCATCTGACTTCACCTCCATGGTCACCCTACCAACTGCCCCAGCTAAGATCTACTGGGAAAAGAGACATCCAGAACAAAAGTACTAG

>78

ATGTCCACTGAAAACACTTCTTTGGTCGTTGAACTATTGGAGTACGTGAAGCTTGGTCTTTCGTACTTCCAAGCTCTGCCATTGGCGCAGAGAGTGTCTATTATGGTCGCCTTGC
CATTTGTGTACACCATCACATGGCAATTGCTTTACTCCTTGAGAAAGGACAGACCACCACTTGTGTTCTACTGGATTCCATGGGTCGGCTCTGCTATCCCATACGGTACCAAGCC
ATACGAGTTCTTCGAAGACTGCCAAAAGAAATACGGTGATATCTTCTCTTTCATGCTATTGGGTAGAATTATGACTGTCTACTTGGGTCCAAAGGGTCACGAATTCATCTTCAAC

GCCAAGTTGGCCGATGTTTCCGCTGAAGCTGCTTACTCCCACTTGACCACCCCAGTGTTCGGTAAAGGTGTTATCTACGATTGTCCAAACCACAGACTAATGGAACAAAAGAAG
TTTGTCAAGGGTGCTTTGACTAAGGAAGCCTTTGTCAGATACGTTCCATTGATCGCTGAGGAAATCTACAAGTACTTCAGAAACTCCAAGAACTTCAAGATCAACGAAAACAAC
TCCGGTATCGTCGACGTTATGGTCTCCCAACCTGAAATGACTATCTTCACTGCTTCCAGATCCTTGCTAGGTAAGGAAATGAGAGACAAGTTGGACACCGACTTCGCTTACTTGT
ACAGTGACTTGGACAAGGGTTTCACCCCAATTAACTTCGTCTTCCCTAACTTGCCTCTAGAACACTACAGAAAGAGAGACCATGCCCAACAAGCTATCTCTGGTACTTACATGTC
CTTGATTAAGGAAAGACGTGAGAAGAACGATATCCAAAACCGTGATTTGATTGATGAATTGATGAAGAACTCCACTTACAAGGATGGTACTAAGATGACCGACCAAGAAATTG
CCAACCTATTGATTGGTGTCTTGATGGGTGGTCAACATACTTCCGCTGCTACCTCCGCTTGGTGTCTATTGCATTTGGCTGAAAGACCAGATGTCCAAGAAGAATTGTACCAAG
AACAAATGCGCGTCTTGAACAACGATACCAAGGAATTGACTTACGATGACCTACAAAACATGCCTCTATTGAACCAAATGATCAAGGAAACTTTGAGATTGCACCACCCATTGC
ACTCTTTGTTCCGTAAAGTCATGAGAGATGTCGCTATTCCAAACACTTCCTACGTTGTCCCAAGGGACTACCACGTTCTAGTCTCCCCAGGTTACACTCACTTGCAAGAAGAATTC
TTCCCTAAGCCAAACGAATTCAACATCCMCCGTTGGGACGGTGATGCTGCTTCTTCCAGTGCTGCTGGTGGTGACGAAGTTGATTACGGTTTCGGTGCTATCTCCAAGGGTGTT
TCCTCTCCATACTTGCCATTCGGTGGTGGTAGACACAGATGTATCGGTGAATTGTTCGCTTACTGTCAATTGGGTGTGTTGATGTCCATTTTCATCAGAACCATGAAATGGCGTT
ACCCAACTGAAGGTGAAACTGTCCCACCATCTGACTTCACCTCCATGGTCACCCTACCAACTGCCCCAGCTAAGATCTACTGGGAAAAGAGACATCCAGAACAAAAGTACTAG-

>79

ATGTCCACTGAAAACACTTCTTTGGTCGTTGAACTATTGGAGTACGTGAAGCTTGGTCTTTCGTACTTCCAAGCTCTGCCATTGGCGCAGAGAGTGTCTATTATGGTCGCCTTGC
CATTTGTGTACACCATCACATGGCAATTGCTTTACTCCTTGAGAAAGGACAGACCACCACTTGTGTTCTACTGGATTCCATGGGTCGGCTCTGCTATCCCATACGGTACCAAGCC
ATACGAGTTCTTCGAAGACTGCCAAAAGAAATACGGTGATATCTTCTCTTTCATGCTATTGGGTAGAATTATGACTGTCTACTTGGGTCCAAAGGGTCACGAATTCATCTTCAAC
GCCAAGTTGGCCGATGTTTCCGCTGAAGCTGCTTACTCCCACTTGACCACCCCAGTGTTCGGTAAAGGTGTTATCTACGATTGTCCAAACCACAGACTAATGGAACAAAAGAAG
TTTGTCAAGGGTGCTTTGACTAAGGAAGCCTTTGTCAGATACGTTCCATTGATCGCTGAGGAAATCTACAAGTACTTCAGAAACTCCAAGAACTTCAAGATCAACGAAAACAAC
TCCGGTATCGTCGACGTTATGGTCTCCCAACCTGAAATGACTATCTTCACTGCTTCCAGATCCTTGCTAGGTAAGGAAATGAGAGACAAGTTGGACACCGACTTCGCTTACTTGT
ACAGTGACTTGGACAAGGGTTTCACCCCAATTAACTTCGTCTTCCCTAACTTGCCTCTAGAACACTACAGAAAGAGAGACCATGCCCAACAAGCTATCTCTGGTACTTACATGTC
CTTGATTAAGGAAAGACGTGAGAAGAACGATATCCAAAACCGTGATTTGATTGATGAATTGATGAAGAACTCCACTTACAAGGATGGTACTAAGATGACCGACCAAGAAATTG
CCAACCTATTGATTGGTGTCTTGATGGGTGGTCAACATACTTCCGCTGCTACCTCCGCTTGGTGTCTATTGCATTTGGCTGAAAGACCAGATGTCCAAGAAGAATTGTACCAAG
AACAAATGCGCGTCTTGAACAACGATACCAAGGAATTGACTTACGATGACCTACAAAACATGCCTCTATTGAACCAAATGATCAAGGAAACTTTGAGATTGCACCACCCATTGC
ACTCTTTGTTCCGTAAAGTCATGAGAGATGTCGCTATTCCAAACACTTCCTACGTTGTCCCAAGGGACTACCACGTTCTAGTCTCCCCAGGTTACACTCACTTGCAAGAAGAATTC
TTCCCTAAGCCAAACGAATTCAACATCCMCCGTTGGGACGGTGATGCTGCTTCTTCCAGTGCTGCTGGTGGTGACGAAGTTGATTACGGTTTCGGTGCTATCTCCAAGGGTGTT
TCCTCTCCATACTTGCCATTCGGTGGTGGTAGACACAGATGTATCGGTGAATTGTTCGCTTACTGTCAATTGGGTGTGTTGATGTCCATTTTCATCAGAACCATGAAATGGCGTT
ACCCAACTGAAGGTGAAACTGTCCCACCATCTGACTTCACCTCCATGGTCACCCTACCAACTGCCCCAGCTAAGATCTACTGGGAAAAGAGACATCCAGAACAAAAGTACTAG-

>80

ATGTCCACTGAAAACACTTCTTTGGTCGTTGAACTATTGGAGTACGTGAAGCTTGGTCTTTCGTACTTCCAAGCTCTGCCATTGGCGCAGAGAGTGTCTATTATGGTCGCCTTGC
CATTTGTGTACACCATCACATGGCAATTGCTTTACTCCTTGAGAAAGGACAGACCACCACTTGTGTTCTACTGGATCCCATGGGTCGGCTCTGCTATCCCATACGGTACCAAGCC

ATACGAGTTCTTCGAAGACTGCCAAAAGAAATACGGTGATATCTTCTCTTTCATGCTATTGGGTAGAATTATGACTGTCTACTTGGGTCCAAAGGGTCACGAATTCATCTTCAAC
GCCAAGTTGGCCGATGTTTCCGCTGAAGCTGCTTACTCCCACTTGACCACCCCGGTGTTCGGTAAAGGTGTTATCTACGATTGTCCAAACCACAGACTAATGGAACAAAAGAAG
TTTGTCAAGGGTGCTTTGACTAAGGAAGCCTTTGTCAGATACGTTCCATTGATCGCTGAGGAAATCTACAAGTACTTCAGAAACTCCAAGAACTTCAAGATCAACGAAAACAAC
TCCGGTATCGTCGACGTTATGGTCTCCCAACCTGAAATGACTATCTTCACTGCTTCCAGATCCTTGCTAGGTAAGGAAATGAGAGACAAGTTGGACACCGACTTCGCTTACTTGT
ACAGTGACTTGGACAAGGGTTTCACCCCAATTAACTTCGTCTTCCCTAACTTGCCTCTAGAACACTACAGAAAGAGAGACCATGCCCAACAAGCTATCTCTGGTACTTACATGTC
CTTGATTAAGGAAAGACGTGAGAAGAACGATATCCAAAACCGTGACTTGATTGATGAATTGATGAAGAACTCCACTTACAAGGATGGTACTAAGATGACCGACCAAGAAATTG
CCAACCTATTGATTGGTGTCTTGATGGGTGGTCAACATACTTCCGCTGCTACCTCCGCTTGGTGTCTATTGCATTTGGCTGAAAGACCAGATGTCCAAGAAGAATTGTACCAAG
AACAAATGCGCGTCTTGAACAACGATACCAAGGAATTGACTTACGATGACCTACAAAACATGCCTCTATTGAACCAAATGATCAAGGAAACTTTGAGATTGCACCACCCATTGC
ACTCTTTGTTCCGTAAAGTCATGAGAGATGTCGCTATTCCAAACACTTCCTACGTTGTCCCAAGGGACTACCACGTTCTAGTCTCCCCAGGTTACACTCACTTGCAAGAAGAATTC
TTCCCTAAGCCAAACGAATTCAACATCCACCGTTGGGACGGTGATGCTGCTTCTTCCAGTGCTGCTGGTGGTGACGAAGTTGATTACGGTTTCGGTGCTATCTCCAAGGGTGTT
TCCTCTCCATACTTGCCATTCGGTGGTGGTAGACACAGATGTATCGGTGAATTGTTCGCTTACTGTCAATTGGGTGTGTTGATGTCCATTTTCATCAGAACCATGAAATGGCGTT
ACCCAACTGAAGGTGAAACTGTCCCACCATCTGACTTCACCTCCATGGTCACCCTACCAACTGCCCCAGCTAAGATCTACTGGGAAAAGAGACATCCAGAACAAAAGTACTAG-

>81

ATGTCCACTGAAAACACTTCTTTGGTCGTTGAACTATTGGAGTACGTGAAGCTTGGTCTTTCGTACTTCCAAGCTCTGCCATTGGCGCAGAGAGTGTCTATTATGGTCGCCTTGC
CATTTGTGTACACCATCACATGGCAATTGCTTTACTCCTTGAGAAAGGACAGACCACCACTTGTGTTCTACTGGATCCCATGGGTCGGCTCTGCTATCCCATACGGTACCAAGCC
ATACGAGTTCTTCGAAGACTGCCAAAAGAAATACGGTGATATCTTCTCTTTCATGCTATTGGGTAGAATTATGACTGTCTACTTGGGTCCAAAGGGTCACGAATTCATCTTCAAC
GCCAAGTTGGCCGATGTTTCCGCTGAAGCTGCTTACTCCCACTTGACCACCCCAGTGTTCGGTAAAGGTGTTATCTATGATTGTCCAAACCACAGACTAATGGAACAAAAGAAG
TTTGTCAAGGGTGCTTTGACTAAGGAAGCCTTTGTCAGATACGTTCCATTGATCGCTGAGGAAATCTACAAGTACTTCAGAAACTCCAAGAACTTCAAGATCAACGAAAACAAC
TCCGGTATCGTCGACGTTATGGTCTCCCAACCTGAAATGACTATCTTCACTGCTTCCAGATCCTTGCTAGGTAAGGAAATGAGAGACAAGTTGGACACCGACTTCGCTTACTTGT
ACAGTGACTTGGACAAGGGTTTCACCCCAATTAACTTCGTCTTCCCTAACTTGCCTCTAGAACACTACAGAAAGAGAGACCATGCCCAACAAGCTATCTCTGGTACTTACATGTC
CTTGATTAAGGAAAGACGTGAGAAGAACGATATCCAAAACCGTGACTTGATTGATGAATTGATGAAGAACTCCACTTACAAGGATGGTACTAAGATGACCGACCAAGAAATTG
CCAACCTATTGATTGGTGTCTTGATGGGTGGTCAACATACTTCCGCTGCTACCTCCGCTTGGTGTCTATTGCATTTGGCTGAAAGACCAGATGTCCAAGAAGAATTGTACCAAG
AACAAATGCGCGTCTTGAACAACGATACCAAGGAATTGACTTACGATGACCTACAAAACATGCCTCTATTGAACCAAATGATCAAGGAAACTTTGAGATTGCACCACCCATTGC
ACTCTTTGTTCCGTAAAGTCATGAGAGATGTCGCTATTCCAAACACTTCCTACGTTGTCCCAAGGGACTACCACGTTCTAGTCTCCCCAGGTTACACTCACTTGCAAGAAGAATTC
TTCCCTAAGCCAAACGAATTCAACATCCACCGTTGGGACGGTGATGCTGCTTCTTCCAGTGCTGCTGGTGGTGACGAAGTTGATTACGGTTTCGGTGCTATCTCCAAGGGTGTT
TCCTCTCCATACTTGCCATTCGGTGGTGGTAGACACAGATGTATCGGTGAATTGTTCGCTTACTGTCAATTGGGTGTGTTGATGTCCATTTTCATCAGAACCATGAAATGGCGTT
ACCCAACTGAAGGTGAAACTGTCCCACCATCTGACTTCACCTCCATGGTCACCCTACCAACTGCCCCAGCTAAGATCTACTGGGAAAAGAGACATCCAGAACAAAAGTACTAG-

>82

ATGTCCACTGAAAACACTTCTTTGGTCGTTGAACTATTGGAGTACGTGAAGCTTGGTCTTTCGTACTTCCAAGCTCTGCCATTGGCGCAGAGAGTGTCTATTATGGTCGCCTTGC
CATTTGTGTACACCATCACATGGCAATTGCTTTACTCCTTGAGAAAGGACAGACCACCACTTGTGTTCTACTGGATCCCATGGGTCGGCTCTGCTATCCCATACGGTACCAAGCC
ATACGAGTTCTTCGAAGACTGCCAAAAGAAATACGGTGATATCTTCTCTTTCATGCTATTGGGTAGAATCATGACTGTCTACTTGGGTCCAAAGGGTCACGAATTCATCTTCAAC
GCCAAGTTGGCCGATGTTTCCGCTGAAGCTGCTTACTCCCACTTGACCACCCCAGTGTTCGGTAAAGGTGTTATCTACGATTGTCCAAACCACAGACTAATGGAACAAAAGAAG
TTTGTCAAGGGTGCTTTGACTAAGGAAGCCTTTGTCAGATACGTTCCATTGATCGCTGAGGAAATCTACAAGTACTTCAGAAACTCCAAGAACTTCAAGATCAACGAAAACAAC
TCCGGTATCGTCGACGTTATGGTCTCCCAACCTGAAATGACTATCTTCACTGCTTCCAGATCCTTGCTAGGTAAGGAAATGAGAGACAAGTTGGACACCGACTTCGCTTACTTGT
ACAGTGACTTGGACAAGGGTTTCACCCCAATTAACTTCGTCTTCCCTAACTTGCCTCTAGAACACTACAGAAAGAGAGACCATGCCCAACAAGCTATCTCTGGTACTTACATGTC
CTTGATTAAGGAAAGACGTGAGAAGAACGACATCCAAAACCGTGACTTGATTGATGAATTGATGAAGAACTCCACTTACAAGGATGGTACTAAGATGACCGACCAAGAAATT
GCCAACCTATTGATTGGTGTCTTGATGGGTGGTCAACATACTTCCGCTGCTACCTCCGCTTGGTGTCTATTGCATTTGGCTGAAAGACCAGATGTCCAAGAAGAATTGTACCAA
GAACAAATGCGCGTCTTGAACAACGATACCAAGGAATTGACTTACGATGACCTACAAAACATGCCTCTATTGAACCAAATGATCAAGGAAACTTTGAGATTGCACCACCCATTG
CACTCTTTGTTCCGTAAAGTCATGAGAGATGTCGCTATTCCAAACACTTCTTACGTTGTCCCAAGGGACTACCACGTTCTAGTCTCCCCAGGTTACACTCACTTGCAAGAAGAATT
CTTCCCTAAGCCAAATGAATTCAACATCCACCGTTGGGACGGTGATGCTGCTTCTTCCAGTGCTGCTGGTGGTGACGAAGTTGATTACGGTTTCGGTGCTATCTCCAAGGGTGT
TTCCTCTCCATACTTGCCATTCGGTGGTGGTAGACACAGATGTATCGGTGAATTGTTCGCTTACTGTCAATTGGGTGTGTTGATGTCCATTTTCATCAGAACCATGAAATGGCGT
TACCCAACTGAAGGTGAAACTGTCCCACCATCTGACTTCACCTCCATGGTCACCCTACCAACTGCCCCAGCTAAGATCTACTGGGAAAAGAGACATCCAGAACAAAAGTACTAG
-

>83

ATGTCCACTGAAAACACTTCTTTGGTCGTTGAACTATTGGAGTACGTGAAGCTTGGTCTTTCGTACTTCCAAGCTCTGCCATTGGCACAGAGAGTGTCTATTATGGTCGCCTTGC
CATTTGTGTACACCATCACATGGCAATTGCTTTACTCCTTGAGAAAGGACAGACCACCACTTGTGTTCTACTGGATTCCATGGGTCGGCTCTGCTATCCCATACGGTACCAAGCC
ATACGAGTTCTTCGAAGACTGCCAAAAGAAATACGGTGATATCTTCTCTTTCATGCTATTGGGTAGAATTATGACTGTCTACTTGGGTCCAAAGGGTCACGAATTCATCTTCAAC
GCCAAGTTGGCCGATGTTTCCGCTGAAGCTGCTTACTCCCACTTGACCACCCCAGTGTTCGGTAAAGGTGTTATCTACGATTGTCCAAACCACAGACTAATGGAACAAAAGAAG
TTTGTCAAGGGTGCTTTGACTAAGGAAGCCTTTGTCAGATACGTTCCATTGATCGCTGAGGAAATCTACAAGTACTTCAGAAACTCCAAGAACTTCAAGATCAACGAAAACAAC
TCCGGTATCGTCGACGTTATGGTCTCCCAACCTGAAATGACTATCTTCACTGCTTCCAGATCCTTGCTAGGTAAGGAAATGAGAGACAAGTTGGACACCGACTTCGCTTACTTGT
ACAGTGACTTGGACAAGGGTTTCACCCCAATTAACTTCGTCTTCCCTAACTTGCCTCTAGAACACTACAGAAAGAGAGACCATGCCCAACAAGCTATCTCTGGTACTTACATGTC
CTTGATTAAGGAAAGACGTGAGAAGAACGACATCCAAAACCGTGACTTGATTGATGAATTGATGAAGAACTCCACTTACAAGGATGGTACTAAGATGACCGACCAAGAAATT
GCCAACCTATTGATTGGTGTCTTGATGGGTGGTCAACATACTTCCGCTGCTACCTCCGCTTGGTGTCTATTGCATTTGGCTGAAAGACCAGATGTCCAAGAAGAATTGTACCAA
GAACAAATGCGCGTCTTGAACAACGATACCAAGGAATTGACTTACGATGACCTACAAAACATGCCTCTATTGAACCAAATGATCAAGGAAACTTTGAGATTGCACCACCCATTG
CACTCTTTGTTCCGTAAAGTCATGAGAGATGTCGCTATTCCAAACACTTCCTACGTTGTCCCAAGGGACTACCACGTTCTAGTCTCCCCAGGTTACACTCACTTGCAAGAAGAATT
CTTCCCTAAGCCAAATGAATTCAACATCCACCGTTGGGACGGTGATGCTGCTTCTTCCAGTGCTGCTGGTGGTGACGAAGTTGATTACGGTTTCGGTGCTATCTCCAAGGGTGT
TTCCTCTCCATACTTGCCATTCGGTGGTGGTAGACACAGATGTATCGGTGAATTGTTCGCTTACTGTCAATTGGGTGTGTTGATGTCCATTTTCATCAGAACCATGAAATGGCGT
TACCCAACTGAAGGTGAAACTGTCCCACCATCTGACTTCACCTCCATGGTCACCCTACCAACTGCCCCAGCTAAGATCTACTGGGAAAAGAGACATCCAGAACAAAAGTACTAG

PDR1 Sequences

>1

ATGCAAACATTAGAAACTACATCAAAATCAAATCCAGGGGAAGTCAAAGCACAGAAGCCTAGTACAAGAAGAACAAAAGTTGGAAAAGCTTGTGATAGCTGTAGAAGGAGG
AAAATAAAATGTAATGGGCTAAAACCTTGTCCATCTTGTACAATCTATGGTTGTGAATGTACATATACTGATGCAAAATCGACAAAAAATCTCAAATCAAATGATGCAGGTAAA
CCAAAACCAACAGGGAGAGTATCAAAGAATAAAGAAACTACTAGAATCGACAAAGATATTAGGAAATCAGAGCAGCAGTATGTACCTATTAATGCTAATATTCATGTTGGTCC
CAGGTTCCCCTCCGAGAATATATTGAATGGATATCCACAATGTGGAGCACCACAGAACAATGTTGTGGGTAATCCACTAGCGGTTAATCCTCAATGCCATAGAGGTCTTTCTGA
AACTCCTATGTCCTCAACATTCAAAGAATCTAACTTAAGAGATGATCGGCTACTACAGTCATCAGATACAGATGATATGAGGAATGGTGACTCGGAAGAAAGGGACTTGAAAG
GGAGTGACAGCGAGAATGTCAAAAGTAAAGACAATAAAAGTGATCCTTTGATTATATACAAAGATGATACACATATTGAAAGCACGGTTAATAAACTAACACAGGCAGTTAAT
GAACTCAAATCACTTCAAAATGCACCTAGTTCGATAAAATCATCCATTGACGCCATTGAGTTACAACTTAGAAACATTTTAGACAATTGGAAACCAGAGGTAGATTTCGAGAAA
GCAAAGATTAATGAAAGTGCCACCACTAAGTCACTTGAAACAAACTTGCTGAGGAATAAATACACTAATCACGTTCATCTAACAAGATTTAGGATATGGATAGATTATAAAAAT
GCGAACAAAAACAATCATTTTATGGGAGAGTGTGGATTTAGTCTTGCAGAATCTTTTTTTGCTTCTAATCAGCCATTGGTCGATGAATTGTTTGGGTTGTATTCCCAGGTAGAGG
CCTTTTCTTTGCAAGGTCTTGGTTACTGTGTTCACCTTTATGAGCCATATATGAAAACTGAGGAAGCGATAAAACTGATGAAAGAGACCTTATATATTATACTACGGTTTATTGA
TATATGTGTTCACCATATCAATGAAGAGTCGATATCGATTGCCAACCCGTTAGAAACATATTTACGAAAAAAACATCTAATGCCTATGACTCCTACACCAAGGTCGTCCTATGGA
AGTCCACAAAGTGCTAGTACAAAGAGCTTGGTAAGTAAGATAATAGAGAGAATACCGCAACCGTTTATTGAGAGTGTAACTAATGTGTCGAGTCTTCAACTATTAGATCTTCGA
GATGACGAGTCAAAAATGTTTGGAACATTGCTGAACATGTGTAAGTCTATAAGGCGAAAATTTGACTCTGTTATGAGCGATTACGATTCCATTGTCACAGAAAAATCCGAAGG
CGAACAAAATGATGGTAAAGTAACTGTAGCTGAGTTCACATCTTTGTGTGAAGCGGAAGAAATGCTCTTAGCATTATGCTATAACTATTATAATCTGACGTTATACAGTTTCTTT
GAATTTGGGACTAATATTGAATACATGGAACATCTGTTGCTTCTTCTTGAAGAACAGCTTGCTCTCGACGAATACTATGGTTTTGAAAAGGTCTTGAATGTAGCTGTTGCAAATG
CTAAAAAAATGGGTTTCCACCGTTGGGAGTTTTACGTCGGTTATGAAGAGTCGACTGCTGAAAAGAGGCGGCTACTATGGTGGAAGTTATACAATTATGAAAAAGCCAGTACT
ATGAAGAAGGGTTTTTTTTCTGTGATTGATGATGCTACTGTCAACTGTTTATTACCTAAGATTTTTAGAAACTTTGGCTATCTGGATAGGGTGGAGTTTCTAGAAAATATTCAAA
AGCCAATGGATCTTAGTGTGTTTTCCGATGTTCCAATTTCTGTCCTTTGTAAATACGGTGAGTTGGCCCTTACAATAGTTACCAGTGAGTTTCATGAAAAATTTTTATATGCTGAT
AGATACACTTCTATTCGAAATTCCGCGAAACCGCCGACATTAAAAAACCAATTAATTAAGGAAATTGTGGATGGTATAGCTTATACAGAGACATCATATGAGGCAATCAGAAA
GCAAACTGCAAAACTATGGGATATTGCATTAGGTAAGGTGACCAAAGATAAAATCAATAAAGAAGATACAGCAGCAGCTAGCAAATTTACTTTGAGTTATGAATATCACAGAT
TCAGGCTAATCAATATGGCAGACAATTTAATTGCTAGACTTATGGTGAAACCAAAATCAGATTGGCTAATATCAGTCATGAAGGGGCATCTTAACAGACTATATGAGCACTGGA
AAGTAATGAATGAAATTATCCTAAGTATGGACAACGATTATTCAATTGCAACAACGTTCGAATATTATGCACCATCATGTCTGTGTTTAGCTACGCAGACTTTCCTTATTGTGAG
GAATATGGAAATGGATGATGTCAAGATGATGGTTGCAGTATATAAAAGATTTCTTAACCTAGGAATGTTTCTGCAGAGTGCCAAAGTATGCAGCCTTGCCGATAGTCATACATT
CAGAGATTTTTCTAGATCTTTTTCCTTTATTACGATAATTTCAAGATTGATGATAATCGAATTTATGCAAATTAAAGAATTGACGAAGGTAGAGTTTATTGAGAAGTTTTCTGAAG
TATGCCCTGACCTTGCAGATCTACCTCCGATGCTTCTAGATCCAAACTCTTGCTTATATTTTTCATTGTTACAGCAGATTAAGAAATCTGGTTTTACGTTGTCATTCAAAAAAAATTC
TTGAAGACGCTAGAATGATGGACTTCAATTACGACCGCAATTTGGACTCAGAGGCCATTAAAAAGTGCAATGGTGAATTTAGCAAGTCAATGCCTTCCTGTACCAATGTCTCAG

ATACCACCACCGCTGTTTCTGACAACAGTGCTAAGAAGAAAGCTTCAATGGGGTCGGCGAGGGTAAATTCAACTGATACACTAACTGCATCTCCCTTATCGGGCTTAAGGAATC
AAACGCAGTTGGATTCTAAAGACAGTGTTCCATCTCTCGAGGCTTATACACCAATTGATTCTGTCTCTGACGTACCCACTGGGGAGATCAACGTTCCATTCCCTCCTGTTTATAAT
CAAAATGGATTGGATCAGCAAACCACTTATAATTTGGGAACTTTAGATGAGTTTGTTAACAAGGGAGATTTGAATGAACTCTATAATAGCCTATGGGGTGACCTATTTTCTGAT
GTTTACTTGTGA

>2

ATGCAAACATTAGAAACTACATCAAAATCAAATCCAGGGGAAGTCAAAGCACAGAAGCCTAGTACAAGAAGAACAAAAGTTGGAAAAGCTTGTGATAGCTGTAGAAGGAGG
AAAATAAAATGTAATGGGCTAAAACCTTGTCCATCTTGTACAATCTATGGTTGTGAATGTACATATACTGATGCAAAATCGACAAAAAATCTCAAATCAAATGATGCAGGTAAA
CCAAAACCAACAGGGAGAGTATCAAAGAATAAAGAAACTACTAGAATCGACAAAGATATTAGGAAATCAGAGCAGCAGTATGTCCCTATTAATGCTAATATTCATGTTGGTCC
CAGGTTCCCCTCCGAGAATATATTGAATGGATATCCACAATGTGGAGCACCACAGAACAATGTTGTGGGTAATCCACTAGCGGTTAATCCTCAATGCCATAGAGGTCTTTCTGA
AACTCCTATGTCCTCAACATTCAAAGAATCTAACTTAAGAGATGATCGGCTACTACAGTCATCAGATACAGATGATATGAGGAATGGTGACTCGGAAGAAAGGGACTTGAAAG
GGAGTGACAGCGAGAATGTCAAAAGTAAAGACAATAAAAGTGATCCTTTGATTATATACAAAGATGATACACATATTGAAAGCACGGTTAATAAACTAACACAGGCAGTTAAT
GAACTCAAATCACTTCAAAATGCACCTAGTTCGATAAAATCATCCATTGACGCCATTGAGTTACAACTTAGAAACATTTTAGACAATTGGAAACCAGAGGTAGATTTCGAGAAA
GCAAAGATTAATGAAAGTGCCACCACTAAGTCACTTGAAACAAACTTGCTGAGGAATAAATACACTAATCACGTTCATCTAACAAGATTTAGGATATGGATAGATTATAAAAAT
GCGAACAAAAACAATCATTTTATGGGAGAGTGTGGATTTAGTCTTGCAGAATCTTTTTTTGCTTCTAATCAGCCATTGGTCGATGAATTGTTTGGGTTGTATTCCCAGGTAGAGG
CCTTTTCTTTGCAAGGTCTTGGTTACTGTGTTCACCTTTATGAGCCATATATGAAAACTGAGGAAGCGATAAAACTGATGAAAGAGACCTTATATATTATACTACGGTTTATTGA
TATATGTGTTCACCATATCAATGAAGAGTCGATATCGATTGCCAACCCGTTAGAAACATATTTACGAAAAAAACATCTAATGCCTATGACTCCTACACCAAGGTCGTCCTATGGA
AGTCCACAAAGTGCTAGTACAAAGAGCTTGGTAAGTAAGATAATAGAGAGAATACCGCAACCGTTTATTGAGAGTGTAACTAATGTGTCGAGTCTTCAACTATTAGATCTTCGA
GATGACGAGTCAAAAATGTTTGGAACATTGCTGAACATGTGTAAGTCTATAAGGCGAAAATTTGACTCTGTTATGAGCGATTACGATTCCATTGTCACAGAAAAATCCGAAGG
CGAACAAAATGATGGTAAAGTAACTGTAGCTGAGTTCACATCTTTGTGTGAAGCGGAAGAAATGCTCTTAGCATTATGCTATAACTATTATAATCTGACGTTATACAGTTTCTTT
GAATTTGGGACTAATATTGAATACATGGAACATCTGTTGCTTCTTCTTGAAGAACAGCTTGCTCTCGACGAATACTATGGTTTTGAAAAGGTCTTGAATGTAGCTGTTGCAAATG
CTAAAAAAATGGGTTTCCACCGTTGGGAGTTTTACGTCGGTTATGAAGAGTCGACTGCTGAAAAGAGGCGGCTACTATGGTGGAAGTTATACAATTATGAAAAAGCCAGTACT
ATGAAGAAGGGTTTTTTTTCTGTGATTGATGATGCTACTGTCAACTGTTTATTACCTAAGATTTTTAGAAACTTTGGCTATCTGGATAGGGTGGAGTTTCTAGAAAATATTCAAA
AGCCAATGGATCTTAGTGTGTTTTCCGATGTTCCAATTTCTGTCCTTTGTAAATACGGTGAGTTGGCCCTTACAATAGTTACCAGTGAGTTTCATGAAAAATTTTTATATGCTGAT
AGATACACTTCTATTCGAAATTCCGCGAAACCGCCGACATTAAAAAACCAATTAATTAAGGAAATTGTGGATGGTATAGCTTATACAGAGACATCATATGAGGCAATCAGAAA
GCAAACTGCAAAACTATGGGATATTGCATTAGGTAAGGTGACCAAAGATAAAATCAATAAAGAAGATACAGCAGCAGCTAGCAAATTTACTTTGAGTTATGAATATCACAGAT
TCAGGCTAATCAATATGGCAGACAATTTAATTGCTAGACTTATGGTGAAACCAAAATCAGATTGGCTAATATCAGTCATGAAGGGGCATCTTAACAGACTATATGAGCACTGGA
AAGTAATGAATGAAATTATCCTAAGTATGGACAACGATTATTCAATTGCAACAACGTTCGAATATTATGCACCATCATGTCTGTGTTTAGCTACGCAGACTTTCCTTATTGTGAG
GAATATGGAAATGGATGATGTCAAGATGATGGTTGCAGTATATAAAAGATTTCTTAACCTAGGAATGTTTCTGCAGAGTGCCAAAGTATGCAGCCTTGCCGATAGTCATACATT
CAGAGATTTTTCTAGATCTTTTTCCTTTATTACGATAATTTCAAGATTGATGATAATCGAATTTATGCAAATTAAAGAATTGACGAAGGTAGAGTTTATTGAGAAGTTTTCTGAAG
TATGCCCTGACCTTGCAGATCTACCTCCGATGCTTCTAGATCCAAACTCTTGCTTATATTTTTCATTGTTACAGCAGATTAAGAAATCTGGTTTTACGTTGTCATTCAAAAAAAATTC

TTGAAGACGCTAGAATGATGGACTTCAATTACGACCGCAATTTGGACTCAGAGGCCATTAAAAAGTGCAATGGTGAATTTAGCAAGTCAATGCCTTCCTGTACCAATGTCTCAG
ATACCACCACCGCTGTTTCTGACAACAGTGCTAAGAAGAAAGCTTCAATGGGGTCGGCGAGGGTAAATTCAACTGATACACTAACTGCATCTCCCTTATCGGGCTTAAGGAATC
AAACGCAGTTGGATTCTAAAGACAGTGTTCCATCTCTCGAGGCTTATACACCAATTGATTCTGTCTCTGACGTACCCACTGGGGAGATCAACGTTCCATTCCCTCCTGTTTATAAT
CAAAATGGATTGGATCAGCAAACCACTTATAATTTGGGAACTTTAGATGAGTTTGTTAACAAGGGAGATTTGAATGAACTCTATAATAGCCTATGGGGTGACCTATTTTCTGAT
GTTTACTTGTGA

>3

ATGCAAACATTAGAAACTACATCAAAATCAAATCCAGGGGAAGTCAAAGCACAGAAGCCTAGTACAAGAAGAACAAAAGTTGGAAAAGCTTGTGATAGCTGTAGAAGGAGG
AAAATAAAATGTAATGGGCTAAAACCTTGTCCATCTTGTACAATCTATGGTTGTGAATGTACATATACTGATGCAAAATCGACAAAAAATCTCAAATCAAATGATGCAGGTAAA
CCAAAACCAACAGGGAGAGTATCAAAGAATAAAGAAACTACTAGAATCGACAAAGATATTAGGAAATCAGAGCAGCAGTATGTACCTATTAATGCTAATATTCATGTTGGTCC
CAGGTTCCCCTCCGAGAATATATTGAATGGATATCCACAATGTGGAGCACCACAGAACAATGTTGTGGGTAATCCACTAGCGGTTAATCCTCAATGCCATAGAGGTCTTTCTGA
AACTCCTATGTCCTCAACATTCAAAGAATCTAACTTAAGAGATGATCGGCTACTACAGTCATCAGATACAGATGATATGAGGAATGGTGACTCGGAAGAAAGGGACTTGAAAG
GGAGTGACAGCGAGAATGTCAAAAGTAAAGACAATAAAAGTGATCCTTTGATTATATACAAAGATGATACACATATTGAAAGCACGGTTAATAAACTAACACAGGCAGTTAAT
GAACTCAAATCACTTCAAAATGCACCTAGTTCGATAAAATCATCCATTGACGCCATTGAGTTACAACTTAGAAACATTTTAGACAATTGGAAACCAGAGGTAGATTTCGAGAAA
GCAAAGATTAATGAAAGTGCCACCACTAAGTCACTTGAAACAAACTTGCTGAGGAATAAATACACTAATCACGTTCATCTAACAAGATTTAGGATATGGATAGATTATAAAAAT
GCGAACAAAAACAATCATTTTATGGGAGAGTGTGGATTTAGTCTTGCAGAATCTTTTTTTGCTTCTAATCAGCCATTGGTCGATGAATTGTTTGGGTTGTATTCCCAGGTAGAGG
CCTTTTCTTTGCAAGGTCTTGGTTACTGTGTTCACCTTTATGAGCCATATATGAAAACTGAGGAAGCGATAAAACTGATGAAAGAGACCTTATATATTATACTACGGTTTATTGA
TATATGTGTTCACCATATCAATGAAGAGTCGATATCGATTGCCAACCCGTTAGAAACATATTTACGAAAAAAACATCTAATGCCTATGACTCCTACACCAAGGTCGTCCTATGGA
AGTCCACAAAGTGCTAGTACAAAGAGCTTGGTAAGTAAGATAATAGAGAGAATACCGCAACCGTTTATTGAGAGTGTAACTAATGTGTCGAGTCTTCAACTATTAGATCTTCGA
GATGACGAGTCAAAAATGTTTGGAACATTGCTGAACATGTGTAAGTCTATAAGGCGAAAATTTGACTCTGTTATGAGCGATTACGATTCCATTGTCACAGAAAAATCCGAAGG
CGAACAAAATGATGGTAAAGTAACTGTAGCTGAGTTCACATCTTTGTGTGAAGCGGAAGAAATGCTCTTAGCATTATGCTATAACTATTATAATCTGACGTTATACAGTTTCTTT
GAATTTGGGACTAATATTGAATACATGGAACATCTGTTGCTTCTTCTTGAAGAACAGCTTGCTCTCGACGAATACTATGGTTTTGAAAAGGTCTTGAATGTAGCTGTTGCAAATG
CTAAAAAAATGGGTTTCCACCGTTGGGAGTTTTACGTCGGTTATGAAGAGTCGACTGCTGAAAAGAGGCGGCTACTATGGTGGAAGTTATACAATTATGAAAAAGCCAGTACT
ATGAAGAAGGGTTTTTTTTCTGTGATTGATGATGCTACTGTCAACTGTTTATTACCTAAGATTTTTAGAAACTTTGGCTATCTGGATAGGGTGGAGTTTCTAGAAAATATTCAAA
AGCCAATGGATCTTAGTGTGTTTTCCGATGTTCCAATTTCTGTCCTTTGTAAATACGGTGAGTTGGCCCTTACAATAGTTACCAGTGAGTTTCATGAAAAATTTTTATATGCTGAT
AGATACACTTCTATTCGAAATTCCGCGAAACCGCCGACATTAAAAAACCAATTAATTAAGGAAATTGTGGATGGTATAGCTTATACAGAGACATCATATGAGGCAATCAGAAA
GCAAACTGCAAAACTATGGGATATTGCATTAGGTAAGGTGACCAAAGATAAAATCAATAAAGAAGATGCAGCAGCAGCTAGCAAATTTACTTTGAGTTATGAATATCACAGAT
TCAGGCTAATCAATATGGCAGACAATTTAATTGCTAGACTTATGGTGAAACCAAAATCAGATTGGCTAATATCAGTCATGAAGGGGCATCTTAACAGACTATATGAGCACTGGA
AAGTAATGAATGAAATTATCCTAAGTATGGACAACGATTATTCAATTGCAACAACGTTCGAATATTATGCACCATCATGTCTGTGTTTAGCTACGCAGACTTTCCTTATTGTGAG
GAATATGGAAATGGATGATGTCAAGATGATGGTTGCAGTATATAAAAGATTTCTTAACCTAGGAATGTTTCTGCAGAGTGCCAAAGTATGCAGCCTTGCCGATAGTCATACATT
CAGAGATTTTTCTAGATCTTTTTCCTTTATTACGATAATTTCAAGATTGATGATAATCGAATTTATGCAAATTAAAGAATTGACGAAGGTAGAGTTTATTGAGAAGTTTTCTGAAG

TATGCCCTGACCTTGCAGATCTACCTCCGATGCTTCTAGATCCAAACTCTTGCTTATATTTTTCATTGTTACAGCAGATTAAGAAATCTGGTTTTACGTTGTCATTCAAAAAAATTC
TTGAAGACGCTAGAATGATGGACTTCAATTACGACCGCAATTTGGACTCAGAGGCCATTAAAAAGTGCAATGGTGAATTTAGCAAGTCAATGCCTTCCTGTACCAATGTCTCAG
ATACCACCACCGCTGTTTCTGACAACAGTGCTAAGAAGAAAGCTTCAATGGGGTCGGCGAGGGTAAATTCAACTGATACACTAACTGCATCTCCCTTATCGGGCTTAAGGAATC
AAACGCAGTTGGATTCTAAAGACAGTGTTCCATCTCTCGAGGCTTATACACCAATTGATTCTGTCTCTGACGTACCCACTGGGGAGATCAACGTTCCATTCCCTCCTGTTTATAAT
CAAAATGGATTGGATCAGCAAACCACTTATAATTTGGGAACTTTAGATGAGTTTGTTAACAAGGGAGATTTGAATGAACTCTATAATAGCCTATGGGGTGACCTATTTTCTGAT
GTTTACTTGTGA

>4

ATGCAAACATTAGAAACTACATCAAAATCAAATCCAGGGGAAGTCAAAGCACAGAAGCCTAGTACAAGAAGAACAAAAGTTGGAAAAGCTTGTGATAGCTGTAGAAGGAGG
AAAATAAAATGTAATGGGCTAAAACCTTGTCCATCTTGTACAATCTATGGCTGTGAATGTACATATACTGATGCAAAATCGACAAAAAATCTCAAATCAAATGATGCAGGTAAA
TCAAAACCAACAGGGAGAGTATCAAAGAATAAAGAAACTACTAGAATCGACAAAGATATTAGGAAATCAGAGCAGCAGTATGTCCCTATTAATGCTAATATTCATGTTGGTCC
CAGGTTCCCCTCCGAGAATATATTGAATGGATATCCACAATGTGGAGCACCACAGAACAATGTTGTGGGTAATCCACTAGCGGTTAATACTCAATGCCATAGAGGTCTTTCTGA
AACTCCTATGTCCTCAACATTCAAAGAATCTAACTTAAGAGATGATCGGCTACTACAGTCATCAGATACAGATGATATGAGGAATGGTGACTCGGAAGAAAGGGACTTGAAAG
GGAGTGACAGCGAGAATGTCAAAAGTAAAGACAATAAAAGTGATCCTTTGATTATATACAAAGATGATACACATATTGAAAGCACGGTTAATAAACTAACACAGGCAGTTAAT
GAACTCAAATCACTTCAAAATGCACCCAGTTCGATAAAATCATCCATTAACGCCATTGAGTTACAACTTAGAAACATTTTAGACAACTGGAAACCAGAGGTAGATTTCGAGAAA
GCAAAGATTAATGAAAGTGCCACCACTAAGTCACTTGAAACAAACTTGCTGAGGAATAAATACACTAATCACGTTCATTTAACAAGATTTAGGATATGGATAGATTATAAAAAT
GCGAACAAAAACAATCATTTTATGGGAGAGTGTGGATTTAGTCTTGCAGAATCTTTTTTTGCTTCTAATCAGCCATTGGTCGATGAATTGTTTGGGTTGTATTCCCAGGTAGAGG
CCTTTTCTTTGCAAGGTCTTGGTTACTGTGTTCACCTTTATGAGCCATATATGAAAACTGAGGAAGCGATAAAACTGATGAAAGAGACCTTATATATTATACTACGGTTTATTGA
TATATGTGTTCACCATATCAATGAAGAGTCGATATCGATTGCCAACCCGTTAGAAACATATTTACGAAAAAAACATCTAATGCCTATGACTCCTACACCAAGGTCGTCCTATGGA
AGTCCACAAAGTGCTAGTACAAAGAGCTTGGTAAGTAAGATAATAGAGAGAATACCGCAACCGTTTATTGAGAGTGTAACTAATGTGTCGAGTCTTCAACTATTAGATCTTCGA
GATGACGAGTCAAAAATGTTTGGAACATTGCTGAACATGTGTAAGTCTATAAGGCGAAAATTTGACTCTGTTATGAGCGATTACGATTCCATTGTCACAGAAAAATCCGAAGG
CGAACAAAATGATGGTAAAGTAACTGTAGCTGAGTTCACATCTTTGTGTGAAGCGGAAGAAATGCTCTTAGCATTATGCTATAACTATTATAATCTGACGTTATACAGTTTCTTT
GAATTTGGGACTAATATTGAATACATGGAACATCTGTTGCTTCTTCTTGAAGAACAGCTTGCTCTCGACGAATACTATGGTTTTGAAAAGGTCTTGAATGTAGCTGTTGCAAATG
CTAAAAAAATGGGTTTCCACCGTTGGGAGTTTTACGTCGGCTATGAAGAGTCGACTGCTGAAAAGAGGCGGCTACTATGGTGGAAGTTATACAATTATGAAAAAGCCAGTACT
ATGAAGAAGGGTTTTTTTTCTGTGATTGATGATGCTACTGTCAACTGTTTATTACCTAAGATTTTTAGAAACTTTGGCTATCTGGATAGGGTGGAGTTTCTAGAAAATATTCAAA
AGCCAATGGATCTTAGTGTGTTTTCCGATGTTCCAATTTCTGTCCTTTGTAAATACGGTGAGTTGGCCCTTACAATAGTTACCAGTGAGTTTCATGAAAAATTTTTATATGCTGAT
AGATACACTTCTATTCGAAATTCCGCGAAACCGCCGACATTAAAAAACCAATTAATTAAGGAAATTGTGGATGGTATAGCTTATACAGAGACATCATATGAGGCAATCAGAAA
GCAAACTGCAAAACTATGGGATATTGCATTAGGTAAGGTGACCAAAGATAAAATCAATAAAGAAGATACAGCAGCAGCTAGCAAATTTACTTTGAGTTATGAATATCACAGAT
TCAGGCTAATCAATATGGCAGACAATTTAATTGCTAGACTAATGGTGAAACCAAAATCAGATTGGCTAATATCAGTCATGAAGGGGCATCTTAACAGACTATATGAGCACTGG
AAAGTAATGAATGAAATTATCCTAAGTATGGACAACGATTATTCAATTGCAACAACGTTCGAATATTATGCACCATCATGTCTGTGTTTAGCTACGCAGACTTTCCTTATTGTGA
GGAATATGGAAATGGATGATGTCAAGATGATGGTTGCAGTATATAAAAGATTTCTTAACCTAGGAATGTTTTTGCAGAGTGCCAAAGTATGCAGCCTTGCCGATAGTCATACAT

TCAGAGATTTTTCTAGATCTTTTTCCTTTATTACGATAATTTCAAGATTGATGATAATCGAATTTATGCAAATTAAAGAATTGACGAAGGTAGAGTTTATTGAGAAGTTTTCTGAA
GTATGCCCTGACCTTGCAGATCTACCTCCGATGCTTCTAGATCCAAACTCTTGCTTATATTTTTCATTGTTACAGCAGATTAAGAAATCTGGTTTTACGTTGTCATTCAAAAAAATT
CTTGAAGACGCTAGAATGATGGACTTCAATTACGACCGCAATTTGGACTCAGAGGCCATTAAAAAGTGCAATGGTGAATTTAGCAAGTCAATGCCTTCCTGTACCAATGTCTCA
GATACCACCACCGCTGTTTCTGACAATAGTGCTAAGAAGAAAGCTTCAATGGGGTCGGCGAGGGTAAATTCAACTGATACACTAACTGCATCTCCCTTATCGGGCTTAAGGAAT
CAAACGCAGTTGGATTCTAAAGACAGTGTTCCATCTCTCGAGGCTTATACACCAATTGATTCTGTCTCTGACGTGCCCACTGGGGAGATCAACGTTCCATTCCCTCCTGTTTATA
ATCAAAATGGATTGGATCAGCAAACCACTTATAATTTGGGAACTTTAGATGAGTTTGTTAACAAGGGAGATTTGAATGAACTCTATAATAGCCTATGGGGTGACCTATTTTCTG
ATGTTTACTTGTGA

>5

ATGCAAACATTAGAAACTACATCAAAATCAAATCCAGGGGAAGTCAAAGCACAGAAGCCTAGTACAAGAAGAACAAAAGTTGGAAAAGCTTGTGATAGCTGTAGAAGGAGG
AAAATAAAATGTAATGGGCTAAAACCTTGTCCATCTTGTACAATCTATGGTTGTGAATGTACATATACTGATGCAAAATCGACAAAAAATCTCAAATCAAATGATGCAGGTAAA
CCAAAACCAACAGGGAGAGTATCAAAGAATAAAGAAACTACTAGAATCGACAAAGATATTAGGAAATCAGAGCAGCAGTATGTCCCTATTAATGCTAATATTCATGTTGGTCC
CAGGTTCCCCTCCGAGAATATATTGAATGGATATCCACAATGTGGAGCACCACAGAACAATGTTGTGGGTAATCCACTAGCGGTTAATCCTCAATGCCATAGAGGTCTTTCTGA
AACTCCTATGTCCTCAACATTCAAAGAATCTAACTTAAGAGATGATCGGCTACTACAGTCATCAGATACAGATGATATGAGGAATGGTGACTCGGAAGAAAGGGACTTGAAAG
GGAGTGACAGCGAGAATGTCAAAAGTAAAGACAATAAAAGTGATCCTTTGATTATATACAAAGATGATACACATATTGAAAGCACGGTTAATAAACTAACACAGGCAGTTAAT
GAACTCAAATCACTTCAAAATGCACCTAGTTCGATAAAATCATCCATTGACGCCATTGAGTTACAACTTAGAAACATTTTAGACAATTGGAAACCAGAGGTAGATTTCGAGAAA
GCAAAGATTAATGAAAGTGCCACCACTAAGTCACTTGAAACAAACTTGCTGAGGAATAAATACACTAATCACGTTCATCTAACAAGATTTAGGATATGGATAGATTATAAAAAT
GCGAACAAAAACAATCATTTTATGGGAGAGTGTGGATTTAGTCTTGCAGAATCTTTTTTTGCTTCTAATCAGCCATTGGTCGATGAATTGTTTGGGTTGTATTCCCAGGTAGAGG
CCTTTTCTTTGCAAGGTCTTGGTTACTGTGTTCACCTTTATGAGCCATATATGAAAACTGAGGAAGCGATAAAACTGATGAAAGAGACCTTATATATTATACTACGGTTTATTGA
TATATGTGTTCACCATATCAATGAAGAGTCGATATCGATTGCCAACCCGTTAGAAACATATTTACGAAAAAAACATCTAATGCCTATGACTCCTACACCAAGGTCGTCCTATGGA
AGTCCACAAAGTGCTAGTACAAAGAGCTTGGTAAGTAAGATAATAGAGAGAATACCGCAACCGTTTATTGAGAGTGTAACTAATGTGTCGAGTCTTCAACTATTAGATCTTCGA
GATGACGAGTCAAAAATGTTTGGAACATTGCTGAACATGTGTAAGTCTATAAGGCGAAAATTTGACTCTGTTATGAGCGATTACGATTCCATTGTCACAGAAAAATCCGAAGG
CGAACAAAATGATGGTAAAGTAACTGTAGCTGAGTTCACATCTTTGTGTGAAGCGGAAGAAATGCTCTTAGCATTATGCTATAACTATTATAATCTGACGTTATACAGTTTCTTT
GAATTTGGGACTAATATTGAATACATGGAACATCTGTTGCTTCTTCTTGAAGAACAGCTTGCTCTCGACGAATACTATGGTTTTGAAAAGGTCTTGAATGTAGCTGTTGCAAATG
CTAAAAAAATGGGTTTCCACCGTTGGGAGTTTTACGTCGGTTATGAAGAGTCGACTGCTGAAAAGAGGCGGCTACTATGGTGGAAGTTATACAATTATGAAAAAGCCAGTACT
ATGAAGAAGGGTTTTTTTTCTGTGATTGATGATGCTACTGTCAACTGTTTATTACCTAAGATTTTTAGAAACTTTGGCTATCTGGATAGGGTGGAGTTTCTAGAAAATATTCAAA
AGCCAATGGATCTTAGTGTGTTTTCCGATGTTCCAATTTCTGTCCTTTGTAAATACGGTGAGTTGGCCCTTACAATAGTTACCAGTGAGTTTCATGAAAAATTTTTATATGCTGAT
AGATACACTTCTATTCGAAATTCCGCGAAACCGCCGACATTAAAAAACCAATTAATTAAGGAAATTGTGGATGGTATAGCTTATACAGAGACATCATATGAGGCAATCAGAAA
GCAAACTGCAAAACTATGGGATATTGCATTAGGTAAGGTGACCAAAGATAAAATCAATAAAGAAGATACAGCAGCAGCTAGCAAATTTACTTTGAGTTATGAATATCACAGAT
TCAGGCTAATCAATATGGCAGACAATTTAATTGCTAGACTTATGGTGAAACCAAAATCAGATTGGCTAATATCAGTCATGAAGGGGCATCTTAACAGACTATATGAGCACTGGA
AAGTAATGAATGAAATTATCCTAAGTATGGACAACGATTATTCAATTGCAACAACGTTCGAATATTATGCACCATCATGTCTGTGTTTAGCTACGCAGACTTTCCTTATTGTGAG

GAATATGGAAATGGATGATGTCAAGATGATGGTTGCAGTATATAAAAGATTTCTTAACCTAGGAATGTTTCTGCAGAGTGCCAAAGTATGCAGCCTTGCCGATAGTCATACATT
CAGAGATTTTTCTAGATCTTTTTCCTTTATTACGATAATTTCAAGATTGATGATAATCGAATTTATGCAAATTAAAGAATTGACGAAGGTAGAGTTTATTGAGAAGTTTTCTGAAG
TATGCCCTGACCTTGCAGATCTACCTCCGATGCTTCTAGATCCAAACTCTTGCTTATATTTTTCATTGTTACAGCAGATTAAGAAATCTGGTTTTACGTTGTCATTCAAAAAAATTC
TTGAAGACGCTAGAATGATGGACTTCAATTACGACCGCAATTTGGACTCAGAGGCCATTAAAAAGTGCAATGGTGAATTTAGCAAGTCAATGCCTTCCTGTACCAATGTCTCAG
ATACCACCACCGCTGTTTCTGACAACAGTGCTAAGAAGAAAGCTTCAATGGGGTCGGCGAGGGTAAATTCAACTGATACACTAACTGCATCTCCCTTATCGGGCTTAAGGAATC
AAACGCAGTTGGATTCTAAAGACAGTGTTCCATCTCTCGAGGCTTATACACCAATTGATTCTGTCTCTGACGTACCCACTGGGGAGATCAACGTTCCATTCCCTCCTGTTTATAAT
CAAAATGGATTGGATCAGCAAACCACTTATAATTTGGGAACTTTAGATGAGTTTGTTAACAAGGGAGATTTGAATGAACTCTATAATAGCCTATGGGGTGACCTATTTTCTGAT
GTTTACTTGTGA

>6

ATGCAAACATTAGAAACTACATCAAAATCAAATCCAGGGGAAGTCAAAGCACAGAAGCCTAGTACAAGAAGAACAAAAGTTGGAAAAGCTTGTGATAGCTGTAGAAGGAGG
AAAATAAAATGTAATGGGCTAAAACCTTGTCCATCTTGTACAATCTATGGTTGTGAATGTACATATACTGATGCAAAATCGACAAAAAATCTCAAATCAAATGATGCAGGTAAA
CCAAAACCAACAGGGAGAGTATCAAAGAATAAAGAAACTACTAGAATCGACAAAGATATTAGGAAATCAGAGCAGCAGTATGTACCTATTAATGCTAATATTCATGTTGGTCC
CAGGTTCCCCTCCGAGAATATATTGAATGGATATCCACAATGTGGAGCACCACAGAACAATGTTGTGGGTAATCCACTAGCGGTTAATCCTCAATGCCATAGAGGTCTTTCTGA
AACTCCTATGTCCTCAACATTCAAAGAATCTAACTTAAGAGATGATCGGCTACTACAGTCATCAGATACAGATGATATGAGGAATGGTGACTCGGAAGAAAGGGACTTGAAAG
GGAGTGACAGCGAGAATGTCAAAAGTAAAGACAATAAAAGTGATCCTTTGATTATATACAAAGATGATACACATATTGAAAGCACGGTTAATAAACTAACACAGGCAGTTAAT
GAACTCAAATCACTTCAAAATGCACCTAGTTCGATAAAATCATCCATTGACGCCATTGAGTTACAACTTAGAAACATTTTAGACAATTGGAAACCAGAGGTAGATTTCGAGAAA
GCAAAGATTAATGAAAGTGCCACCACTAAGTCACTTGAAACAAACTTGCTGAGGAATAAATACACTAATCACGTTCATCTAACAAGATTTAGGATATGGATAGATTATAAAAAT
GCGAACAAAAACAATCATTTTATGGGAGAGTGTGGATTTAGTCTTGCAGAATCTTTTTTTGCTTCTAATCAGCCATTGGTCGATGAATTGTTTGGGTTGTATTCCCAGGTAGAGG
CCTTTTCTTTGCAAGGTCTTGGTTACTGTGTTCACCTTTATGAGCCATATATGAAAACTGAGGAAGCGATAAAACTGATGAAAGAGACCTTATATATTATACTACGGTTTATTGA
TATATGTGTTCACCATATCAATGAAGAGTCGATATCGATTGCCAACCCGTTAGAAACATATTTACGAAAAAAACATCTAATGCCTATGACTCCTACACCAAGGTCGTCCTATGGA
AGTCCACAAAGTGCTAGTACAAAGAGCTTGGTAAGTAAGATAATAGAGAGAATACCGCAACCGTTTATTGAGAGTGTAACTAATGTGTCGAGTCTTCAACTATTAGATCTTCGA
GATGACGAGTCAAAAATGTTTGGAACATTGCTGAACATGTGTAAGTCTATAAGGCGAAAATTTGACTCTGTTATGAGCGATTACGATTCCATTGTCACAGAAAAATCCGAAGG
CGAACAAAATGATGGTAAAGTAACTGTAGCTGAGTTCACATCTTTGTGTGAAGCGGAAGAAATGCTCTTAGCATTATGCTATAACTATTATAATCTGACGTTATACAGTTTCTTT
GAATTTGGGACTAATATTGAATACATGGAACATCTGTTGCTTCTTCTTGAAGAACAGCTTGCTCTCGACGAATACTATGGTTTTGAAAAGGTCTTGAATGTAGCTGTTGCAAATG
CTAAAAAAATGGGTTTCCACCGTTGGGAGTTTTACGTCGGTTATGAAGAGTCGACTGCTGAAAAGAGGCGGCTACTATGGTGGAAGTTATACAATTATGAAAAAGCCAGTACT
ATGAAGAAGGGTTTTTTTTCTGTGATTGATGATGCTACTGTCAACTGTTTATTACCTAAGATTTTTAGAAACTTTGGCTATCTGGATAGGGTGGAGTTTCTAGAAAATATTCAAA
AGCCAATGGATCTTAGTGTGTTTTCCGATGTTCCAATTTCTGTCCTTTGTAAATACGGTGAGTTGGCCCTTACAATAGTTACCAGTGAGTTTCATGAAAAATTTTTATATGCTGAT
AGATACACTTCTATTCGAAATTCCGCGAAACCGCCGACATTAAAAAACCAATTAATTAAGGAAATTGTGGATGGTATAGCTTATACAGAGACATCATATGAGGCAATCAGAAA
GCAAACTGCAAAACTATGGGATATTGCATTAGGTAAGGTGACCAAAGATAAAATCAATAAAGAAGATGCAGCAGCAGCTAGCAAATTTACTTTGAGTTATGAATATCACAGAT
TCAGGCTAATCAATATGGCAGACAATTTAATTGCTAGACTTATGGTGAAACCAAAATCAGATTGGCTAATATCAGTCATGAAGGGGCATCTTAACAGACTATATGAGCACTGGA

AAGTAATGAATGAAATTATCCTAAGTATGGACAACGATTATTCAATTGCAACAACGTTCGAATATTATGCACCATCATGTCTGTGTTTAGCTACGCAGACTTTCCTTATTGTGAG
GAATATGGAAATGGATGATGTCAAGATGATGGTTGCAGTATATAAAAGATTTCTTAACCTAGGAATGTTTCTGCAGAGTGCCAAAGTATGCAGCCTTGCCGATAGTCATACATT
CAGAGATTTTTCTAGATCTTTTTCCTTTATTACGATAATTTCAAGATTGATGATAATCGAATTTATGCAAATTAAAGAATTGACGAAGGTAGAGTTTATTGAGAAGTTTTCTGAAG
TATGCCCTGACCTTGCAGATCTACCTCCGATGCTTCTAGATCCAAACTCTTGCTTATATTTTTCATTGTTACAGCAGATTAAGAAATCTGGTTTTACGTTGTCATTCAAAAAAATTC
TTGAAGACGCTAGAATGATGGACTTCAATTACGACCGCAATTTGGACTCAGAGGCCATTAAAAAGTGCAATGGTGAATTTAGCAAGTCAATGCCTTCCTGTACCAATGTCTCAG
ATACCACCACCGCTGTTTCTGACAACAGTGCTAAGAAGAAAGCTTCAATGGGGTCGGCGAGGGTAAATTCAACTGATACACTAACTGCATCTCCCTTATCGGGCTTAAGGAATC
AAACGCAGTTGGATTCTAAAGACAGTGTTCCATCTCTCGAGGCTTATACACCAATTGATTCTGTCTCTGACGTACCCACTGGGGAGATCAACGTTCCATTCCCTCCTGTTTATAAT
CAAAATGGATTGGATCAGCAAACCACTTATAATTTGGGAACTTTAGATGAGTTTGTTAACAAGGGAGATTTGAATGAACTCTATAATAGCCTATGGGGTGACCTATTTTCTGAT
GTTTACTTGTGA

>7

ATGCAAACATTAGAAACTACATCAAAATCAAATCCAGGGGAAGTCAAAGCACAGAAGCCTAGTACAAGAAGAACAAAAGTTGGAAAAGCTTGTGATAGCTGTAGAAGGAGG
AAAATAAAATGTAATGGGCTAAAACCTTGTCCATCTTGTACAATCTATGGTTGTGAATGTACATATACTGATGCAAAATCGACAAAAAATCTCAAATCAAATGATGCAGGTAAA
CCAAAACCAACAGGGAGAGTATCAAAGAATAAAGAAACTACTAGAATCGACAAAGATATTAGGAAATCAGAGCAGCAGTATGTACCTATTAATGCTAATATTCATGTTGGTCC
CAGGTTCCCCTCCGAGAATATATTGAATGGATATCCACAATGTGGAGCACCACAGAACAATGTTGTGGGTAATCCACTAGCGGTTAATCCTCAATGCCATAGAGGTCTTTCTGA
AACTCCTATGTCCTCAACATTCAAAGAATCTAACTTAAGAGATGATCGGCTACTACAGTCATCAGATACAGATGATATGAGGAATGGTGACTCGGAAGAAAGGGACTTGAAAG
GGAGTGACAGCGAGAATGTCAAAAGTAAAGACAATAAAAGTGATCCTTTGATTATATACAAAGATGATACACATATTGAAAGCACGGTTAATAAACTAACACAGGCAGTTAAT
GAACTCAAATCACTTCAAAATGCACCTAGTTCGATAAAATCATCCATTGACGCCATTGAGTTACAACTTAGAAACATTTTAGACAATTGGAAACCAGAGGTAGATTTCGAGAAA
GCAAAGATTAATGAAAGTGCCACCACTAAGTCACTTGAAACAAACTTGCTGAGGAATAAATACACTAATCACGTTCATCTAACAAGATTTAGGATATGGATAGATTATAAAAAT
GCGAACAAAAACAATCATTTTATGGGAGAGTGTGGATTTAGTCTTGCAGAATCTTTTTTTGCTTCTAATCAGCCATTGGTCGATGAATTGTTTGGGTTGTATTCCCAGGTAGAGG
CCTTTTCTTTGCAAGGTCTTGGTTACTGTGTTCACCTTTATGAGCCATATATGAAAACTGAGGAAGCGATAAAACTGATGAAAGAGACCTTATATATTATACTACGGTTTATTGA
TATATGTGTTCACCATATCAATGAAGAGTCGATATCGATTGCCAACCCGTTAGAAACATATTTACGAAAAAAACATCTAATGCCTATGACTCCTACACCAAGGTCGTCCTATGGA
AGTCCACAAAGTGCTAGTACAAAGAGCTTGGTAAGTAAGATAATAGAGAGAATACCGCAACCGTTTATTGAGAGTGTAACTAATGTGTCGAGTCTTCAACTATTAGATCTTCGA
GATGACGAGTCAAAAATGTTTGGAACATTGCTGAACATGTGTAAGTCTATAAGGCGAAAATTTGACTCTGTTATGAGCGATTACGATTCCATTGTCACAGAAAAATCCGAAGG
CGAACAAAATGATGGTAAAGTAACTGTAGCTGAGTTCACATCTTTGTGTGAAGCGGAAGAAATGCTCTTAGCATTATGCTATAACTATTATAATCTGACGTTATACAGTTTCTTT
GAATTTGGGACTAATATTGAATACATGGAACATCTGTTGCTTCTTCTTGAAGAACAGCTTGCTCTCGACGAATACTATGGTTTTGAAAAGGTCTTGAATGTAGCTGTTGCAAATG
CTAAAAAAATGGGTTTCCACCGTTGGGAGTTTTACGTCGGTTATGAAGAGTCGACTGCTGAAAAGAGGCGGCTACTATGGTGGAAGTTATACAATTATGAAAAAGCCAGTACT
ATGAAGAAGGGTTTTTTTTCTGTGATTGATGATGCTACTGTCAACTGTTTATTACCTAAGATTTTTAGAAACTTTGGCTATCTGGATAGGGTGGAGTTTCTAGAAAATATTCAAA
AGCCAATGGATCTTAGTGTGTTTTCCGATGTTCCAATTTCTGTCCTTTGTAAATACGGTGAGTTGGCCCTTACAATAGTTACCAGTGAGTTTCATGAAAAATTTTTATATGCTGAT
AGATACACTTCTATTCGAAATTCCGCGAAACCGCCGACATTAAAAAACCAATTAATTAAGGAAATTGTGGATGGTATAGCTTATACAGAGACATCATATGAGGCAATCAGAAA
GCAAACTGCAAAACTATGGGATATTGCATTAGGTAAGGTGACCAAAGATAAAATCAATAAAGAAGATGCAGCAGCAGCTAGCAAATTTACTTTGAGTTATGAATATCACAGAT

TCAGGCTAATCAATATGGCAGACAATTTAATTGCTAGACTTATGGTGAAACCAAAATCAGATTGGCTAATATCAGTCATGAAGGGGCATCTTAACAGACTATATGAGCACTGGA
AAGTAATGAATGAAATTATCCTAAGTATGGACAACGATTATTCAATTGCAACAACGTTCGAATATTATGCACCATCATGTCTGTGTTTAGCTACGCAGACTTTCCTTATTGTGAG
GAATATGGAAATGGATGATGTCAAGATGATGGTTGCAGTATATAAAAGATTTCTTAACCTAGGAATGTTTCTGCAGAGTGCCAAAGTATGCAGCCTTGCCGATAGTCATACATT
CAGAGATTTTTCTAGATCTTTTTCCTTTATTACGATAATTTCAAGATTGATGATAATCGAATTTATGCAAATTAAAGAATTGACGAAGGTAGAGTTTATTGAGAAGTTTTCTGAAG
TATGCCCTGACCTTGCAGATCTACCTCCGATGCTTCTAGATCCAAACTCTTGCTTATATTTTTCATTGTTACAGCAGATTAAGAAATCTGGTTTTACGTTGTCATTCAAAAAAATTC
TTGAAGACGCTAGAATGATGGACTTCAATTACGACCGCAATTTGGACTCAGAGGCCATTAAAAAGTGCAATGGTGAATTTAGCAAGTCAATGCCTTCCTGTACCAATGTCTCAG
ATACCACCACCGCTGTTTCTGACAACAGTGCTAAGAAGAAAGCTTCAATGGGGTCGGCGAGGGTAAATTCAACTGATACACTAACTGCATCTCCCTTATCGGGCTTAAGGAATC
AAACGCAGTTGGATTCTAAAGACAGTGTTCCATCTCTCGAGGCTTATACACCAATTGATTCTGTCTCTGACGTACCCACTGGGGAGATCAACGTTCCATTCCCTCCTGTTTATAAT
CAAAATGGATTGGATCAGCAAACCACTTATAATTTGGGAACTTTAGATGAGTTTGTTAACAAGGGAGATTTGAATGAACTCTATAATAGCCTATGGGGTGACCTATTTTCTGAT
GTTTACTTGTGA

>8

ATGCAAACATTAGAAACTACATCAAAATCAAATCCAGGGGAAGTCAAAGCACAGAAGCCTAGTACAAGAAGAACAAAAGTTGGAAAAGCTTGTGATAGCTGTAGAAGGAGG
AAAATAAAATGTAATGGGCTAAAACCTTGTCCATCTTGTACAATCTATGGTTGTGAATGTACATATACTGATGCAAAATCGACAAAAAATCTCAAATCAAATGATGCAGGTAAA
CCAAAACCAACAGGGAGAGTATCAAAGAATAAAGAAACTACTAGAATCGACAAAGATATTAGGAAATCAGAGCAGCAGTATGTACCTATTAATGCTAATATTCATGTTGGTCC
CAGGTTCCCCTCCGAGAATATATTGAATGGATATCCACAATGTGGAGCACCACAGAACAATGTTGTGGGTAATCCACTAGCGGTTAATCCTCAATGCCATAGAGGTCTTTCTGA
AACTCCTATGTCCTCAACATTCAAAGAATCTAACTTAAGAGATGATCGGCTACTACAGTCATCAGATACAGATGATATGAGGAATGGTGACTCGGAAGAAAGGGACTTGAAAG
GGAGTGACAGCGAGAATGTCAAAAGTAAAGACAATAAAAGTGATCCTTTGATTATATACAAAGATGATACACATATTGAAAGCACGGTTAATAAACTAACACAGGCAGTTAAT
GAACTCAAATCACTTCAAAATGCACCTAGTTCGATAAAATCATCCATTGACGCCATTGAGTTACAACTTAGAAACATTTTAGACAATTGGAAACCAGAGGTAGATTTCGAGAAA
GCAAAGATTAATGAAAGTGCCACCACTAAGTCACTTGAAACAAACTTGCTGAGGAATAAATACACTAATCACGTTCATCTAACAAGATTTAGGATATGGATAGATTATAAAAAT
GCGAACAAAAACAATCATTTTATGGGAGAGTGTGGATTTAGTCTTGCAGAATCTTTTTTTGCTTCTAATCAGCCATTGGTCGATGAATTGTTTGGGTTGTATTCCCAGGTAGAGG
CCTTTTCTTTGCAAGGTCTTGGTTACTGTGTTCACCTTTATGAGCCATATATGAAAACTGAGGAAGCGATAAAACTGATGAAAGAGACCTTATATATTATACTACGGTTTATTGA
TATATGTGTTCACCATATCAATGAAGAGTCGATATCGATTGCCAACCCGTTAGAAACATATTTACGAAAAAAACATCTAATGCCTATGACTCCTACACCAAGGTCGTCCTATGGA
AGTCCACAAAGTGCTAGTACAAAGAGCTTGGTAAGTAAGATAATAGAGAGAATACCGCAACCGTTTATTGAGAGTGTAACTAATGTGTCGAGTCTTCAACTATTAGATCTTCGA
GATGACGAGTCAAAAATGTTTGGAACATTGCTGAACATGTGTAAGTCTATAAGGCGAAAATTTGACTCTGTTATGAGCGATTACGATTCCATTGTCACAGAAAAATCCGAAGG
CGAACAAAATGATGGTAAAGTAACTGTAGCTGAGTTCACATCTTTGTGTGAAGCGGAAGAAATGCTCTTAGCATTATGCTATAACTATTATAATCTGACGTTATACAGTTTCTTT
GAATTTGGGACTAATATTGAATACATGGAACATCTGTTGCTTCTTCTTGAAGAACAGCTTGCTCTCGACGAATACTATGGTTTTGAAAAGGTCTTGAATGTAGCTGTTGCAAATG
CTAAAAAAATGGGTTTCCACCGTTGGGAGTTTTACGTCGGTTATGAAGAGTCGACTGCTGAAAAGAGGCGGCTACTATGGTGGAAGTTATACAATTATGAAAAAGCCAGTACT
ATGAAGAAGGGTTTTTTTTCTGTGATTGATGATGCTACTGTCAACTGTTTATTACCTAAGATTTTTAGAAACTTTGGCTATCTGGATAGGGTGGAGTTTCTAGAAAATATTCAAA
AGCCAATGGATCTTAGTGTGTTTTCCGATGTTCCAATTTCTGTCCTTTGTAAATACGGTGAGTTGGCCCTTACAATAGTTACCAGTGAGTTTCATGAAAAATTTTTATATGCTGAT
AGATACACTTCTATTCGAAATTCCGCGAAACCGCCGACATTAAAAAACCAATTAATTAAGGAAATTGTGGATGGTATAGCTTATACAGAGACATCATATGAGGCAATCAGAAA

GCAAACTGCAAAACTATGGGATATTGCATTAGGTAAGGTGACCAAAGATAAAATCAATAAAGAAGATGCAGCAGCAGCTAGCAAATTTACTTTGAGTTATGAATATCACAGAT
TCAGGCTAATCAATATGGCAGACAATTTAATTGCTAGACTTATGGTGAAACCAAAATCAGATTGGCTAATATCAGTCATGAAGGGGCATCTTAACAGACTATATGAGCACTGGA
AAGTAATGAATGAAATTATCCTAAGTATGGACAACGATTATTCAATTGCAACAACGTTCGAATATTATGCACCATCATGTCTGTGTTTAGCTACGCAGACTTTCCTTATTGTGAG
GAATATGGAAATGGATGATGTCAAGATGATGGTTGCAGTATATAAAAGATTTCTTAACCTAGGAATGTTTCTGCAGAGTGCCAAAGTATGCAGCCTTGCCGATAGTCATACATT
CAGAGATTTTTCTAGATCTTTTTCCTTTATTACGATAATTTCAAGATTGATGATAATCGAATTTATGCAAATTAAAGAATTGACGAAGGTAGAGTTTATTGAGAAGTTTTCTGAAG
TATGCCCTGACCTTGCAGATCTACCTCCGATGCTTCTAGATCCAAACTCTTGCTTATATTTTTCATTGTTACAGCAGATTAAGAAATCTGGTTTTACGTTGTCATTCAAAAAAATTC
TTGAAGACGCTAGAATGATGGACTTCAATTACGACCGCAATTTGGACTCAGAGGCCATTAAAAAGTGCAATGGTGAATTTAGCAAGTCAATGCCTTCCTGTACCAATGTCTCAG
ATACCACCACCGCTGTTTCTGACAACAGTGCTAAGAAGAAAGCTTCAATGGGGTCGGCGAGGGTAAATTCAACTGATACACTAACTGCATCTCCCTTATCGGGCTTAAGGAATC
AAACGCAGTTGGATTCTAAAGACAGTGTTCCATCTCTCGAGGCTTATACACCAATTGATTCTGTCTCTGACGTACCCACTGGGGAGATCAACGTTCCATTCCCTCCTGTTTATAAT
CAAAATGGATTGGATCAGCAAACCACTTATAATTTGGGAACTTTAGATGAGTTTGTTAACAAGGGAGATTTGAATGAACTCTATAATAGCCTATGGGGTGACCTATTTTCTGAT
GTTTACTTGTGA

>9

ATGCAAACATTAGAAACTACATCAAAATCAAATCCAGGGGAAGTCAAAGCACAGAAGCCTAGTACAAGAAGAACAAAAGTTGGAAAAGCTTGTGATAGCTGTAGAAGGAGG
AAAATAAAATGTAATGGGCTAAAACCTTGTCCATCTTGTACAATCTATGGTTGTGAATGTACATATACTGATGCAAAATCGACAAAAAATCTCAAATCAAATGATGCAGGTAAA
CCAAAACCAACAGGGAGAGTATCAAAGAATAAAGAAACTACTAGAATCGACAAAGATATTAGGAAATCAGAGCAGCAGTATGTACCTATTAATGCTAATATTCATGTTGGTCC
CAGGTTCCCCTCCGAGAATATATTGAATGGATATCCACAATGTGGAGCACCACAGAACAATGTTGTGGGTAATCCACTAGCGGTTAATCCTCAATGCCATAGAGGTCTTTCTGA
AACTCCTATGTCCTCAACATTCAAAGAATCTAACTTAAGAGATGATCGGCTACTACAGTCATCAGATACAGATGATATGAGGAATGGTGACTCGGAAGAAAGGGACTTGAAAG
GGAGTGACAGCGAGAATGTCAAAAGTAAAGACAATAAAAGTGATCCTTTGATTATATACAAAGATGATACACATATTGAAAGCACGGTTAATAAACTAACACAGGCAGTTAAT
GAACTCAAATCACTTCAAAATGCACCTAGTTCGATAAAATCATCCATTGACGCCATTGAGTTACAACTTAGAAACATTTTAGACAATTGGAAACCAGAGGTAGATTTCGAGAAA
GCAAAGATTAATGAAAGTGCCACCACTAAGTCACTTGAAACAAACTTGCTGAGGAATAAATACACTAATCACGTTCATCTAACAAGATTTAGGATATGGATAGATTATAAAAAT
GCGAACAAAAACAATCATTTTATGGGAGAGTGTGGATTTAGTCTTGCAGAATCTTTTTTTGCTTCTAATCAGCCATTGGTCGATGAATTGTTTGGGTTGTATTCCCAGGTAGAGG
CCTTTTCTTTGCAAGGTCTTGGTTACTGTGTTCACCTTTATGAGCCATATATGAAAACTGAGGAAGCGATAAAACTGATGAAAGAGACCTTATATATTATACTACGGTTTATTGA
TATATGTGTTCACCATATCAATGAAGAGTCGATATCGATTGCCAACCCGTTAGAAACATATTTACGAAAAAAACATCTAATGCCTATGACTCCTACACCAAGGTCGTCCTATGGA
AGTCCACAAAGTGCTAGTACAAAGAGCTTGGTAAGTAAGATAATAGAGAGAATACCGCAACCGTTTATTGAGAGTGTAACTAATGTGTCGAGTCTTCAACTATTAGATCTTCGA
GATGACGAGTCAAAAATGTTTGGAACATTGCTGAACATGTGTAAGTCTATAAGGCGAAAATTTGACTCTGTTATGAGCGATTACGATTCCATTGTCACAGAAAAATCCGAAGG
CGAACAAAATGATGGTAAAGTAACTGTAGCTGAGTTCACATCTTTGTGTGAAGCGGAAGAAATGCTCTTAGCATTATGCTATAACTATTATAATCTGACGTTATACAGTTTCTTT
GAATTTGGGACTAATATTGAATACATGGAACATCTGTTGCTTCTTCTTGAAGAACAGCTTGCTCTCGACGAATACTATGGTTTTGAAAAGGTCTTGAATGTAGCTGTTGCAAATG
CTAAAAAAATGGGTTTCCACCGTTGGGAGTTTTACGTCGGTTATGAAGAGTCGACTGCTGAAAAGAGGCGGCTACTATGGTGGAAGTTATACAATTATGAAAAAGCCAGTACT
ATGAAGAAGGGTTTTTTTTCTGTGATTGATGATGCTACTGTCAACTGTTTATTACCTAAGATTTTTAGAAACTTTGGCTATCTGGATAGGGTGGAGTTTCTAGAAAATATTCAAA
AGCCAATGGATCTTAGTGTGTTTTCCGATGTTCCAATTTCTGTCCTTTGTAAATACGGTGAGTTGGCCCTTACAATAGTTACCAGTGAGTTTCATGAAAAATTTTTATATGCTGAT

AGATACACTTCTATTCGAAATTCCGCGAAACCGCCGACATTAAAAAACCAATTAATTAAGGAAATTGTGGATGGTATAGCTTATACAGAGACATCATATGAGGCAATCAGAAA
GCAAACTGCAAAACTATGGGATATTGCATTAGGTAAGGTGACCAAAGATAAAATCAATAAAGAAGATGCAGCAGCAGCTAGCAAATTTACTTTGAGTTATGAATATCACAGAT
TCAGGCTAATCAATATGGCAGACAATTTAATTGCTAGACTTATGGTGAAACCAAAATCAGATTGGCTAATATCAGTCATGAAGGGGCATCTTAACAGACTATATGAGCACTGGA
AAGTAATGAATGAAATTATCCTAAGTATGGACAACGATTATTCAATTGCAACAACGTTCGAATATTATGCACCATCATGTCTGTGTTTAGCTACGCAGACTTTCCTTATTGTGAG
GAATATGGAAATGGATGATGTCAAGATGATGGTTGCAGTATATAAAAGATTTCTTAACCTAGGAATGTTTCTGCAGAGTGCCAAAGTATGCAGCCTTGCCGATAGTCATACATT
CAGAGATTTTTCTAGATCTTTTTCCTTTATTACGATAATTTCAAGATTGATGATAATCGAATTTATGCAAATTAAAGAATTGACGAAGGTAGAGTTTATTGAGAAGTTTTCTGAAG
TATGCCCTGACCTTGCAGATCTACCTCCGATGCTTCTAGATCCAAACTCTTGCTTATATTTTTCATTGTTACAGCAGATTAAGAAATCTGGTTTTACGTTGTCATTCAAAAAAATTC
TTGAAGACGCTAGAATGATGGACTTCAATTACGACCGCAATTTGGACTCAGAGGCCATTAAAAAGTGCAATGGTGAATTTAGCAAGTCAATGCCTTCCTGTACCAATGTCTCAG
ATACCACCACCGCTGTTTCTGACAACAGTGCTAAGAAGAAAGCTTCAATGGGGTCGGCGAGGGTAAATTCAACTGATACACTAACTGCATCTCCCTTATCGGGCTTAAGGAATC
AAACGCAGTTGGATTCTAAAGACAGTGTTCCATCTCTCGAGGCTTATACACCAATTGATTCTGTCTCTGACGTACCCACTGGGGAGATCAACGTTCCATTCCCTCCTGTTTATAAT
CAAAATGGATTGGATCAGCAAACCACTTATAATTTGGGAACTTTAGATGAGTTTGTTAACAAGGGAGATTTGAATGAACTCTATAATAGCCTATGGGGTGACCTATTTTCTGAT
GTTTACTTGTGA

>10

ATGCAAACATTAGAAACTACATCAAAATCAAATCCAGGGGAAGTCAAAGCACAGAAGCCTAGTACAAGAAGAACAAAAGTTGGAAAAGCTTGTGATAGCTGTAGAAGGAGG
AAAATAAAATGTAATGGGCTAAAACCTTGTCCATCTTGTACAATCTATGGCTGTGAATGTACATATACTGATGCAAAATCGACAAAAAATCTCAAATCAAATGATGCAGGTAAA
TCAAAACCAACAGGGAGAGTATCAAAGAATAAAGAAACTACTAGAATCGACAAAGATATTAGGAAATCAGAGCAGCAGTATGTCCCTATTAATGCTAATATTCATGTTGGTCC
CAGGTTCCCCTCCGAGAATATATTGAATGGATATCCACAATGTGGAGCACCACAGAACAATGTTGTGGGTAATCCACTAGCGGTTAATACTCAATGCCATAGAGGTCTTTCTGA
AACTCCTATGTCCTCAACATTCAAAGAATCTAACTTAAGAGATGATCGGCTACTACAGTCATCAGATACAGATGATATGAGGAATGGTGACTCGGAAGAAAGGGACTTGAAAG
GGAGTGACAGCGAGAATGTCAAAAGTAAAGACAATAAAAGTGATCCTTTGATTATATACAAAGATGATACACATATTGAAAGCACGGTTAATAAACTAACACAGGCAGTTAAT
GAACTCAAATCACTTCAAAATGCACCCAGTTCGATAAAATCATCCATTAACGCCATTGAGTTACAACTTAGAAACATTTTAGACAACTGGAAACCAGAGGTAGATTTCGAGAAA
GCAAAGATTAATGAAAGTGCCACCACTAAGTCACTTGAAACAAACTTGCTGAGGAATAAATACACTAATCACGTTCATTTAACAAGATTTAGGATATGGATAGATTATAAAAAT
GCGAACAAAAACAATCATTTTATGGGAGAGTGTGGATTTAGTCTTGCAGAATCTTTTTTTGCTTCTAATCAGCCATTGGTCGATGAATTGTTTGGGTTGTATTCCCAGGTAGAGG
CCTTTTCTTTGCAAGGTCTTGGTTACTGTGTTCACCTTTATGAGCCATATATGAAAACTGAGGAAGCGATAAAACTGATGAAAGAGACCTTATATATTATACTACGGTTTATTGA
TATATGTGTTCACCATATCAATGAAGAGTCGATATCGATTGCCAACCCGTTAGAAACATATTTACGAAAAAAACATCTAATGCCTATGACTCCTACACCAAGGTCGTCCTATGGA
AGTCCACAAAGTGCTAGTACAAAGAGCTTGGTAAGTAAGATAATAGAGAGAATACCGCAACCGTTTATTGAGAGTGTAACTAATGTGTCGAGTCTTCAACTATTAGATCTTCGA
GATGACGAGTCAAAAATGTTTGGAACATTGCTGAACATGTGTAAGTCTATAAGGCGAAAATTTGACTCTGTTATGAGCGATTACGATTCCATTGTCACAGAAAAATCCGAAGG
CGAACAAAATGATGGTAAAGTAACTGTAGCTGAGTTCACATCTTTGTGTGAAGCGGAAGAAATGCTCTTAGCATTATGCTATAACTATTATAATCTGACGTTATACAGTTTCTTT
GAATTTGGGACTAATATTGAATACATGGAACATCTGTTGCTTCTTCTTGAAGAACAGCTTGCTCTCGACGAATACTATGGTTTTGAAAAGGTCTTGAATGTAGCTGTTGCAAATG
CTAAAAAAATGGGTTTCCACCGTTGGGAGTTTTACGTCGGCTATGAAGAGTCGACTGCTGAAAAGAGGCGGCTACTATGGTGGAAGTTATACAATTATGAAAAAGCCAGTACT
ATGAAGAAGGGTTTTTTTTCTGTGATTGATGATGCTACTGTCAACTGTTTATTACCTAAGATTTTTAGAAACTTTGGCTATCTGGATAGGGTGGAGTTTCTAGAAAATATTCAAA

AGCCAATGGATCTTAGTGTGTTTTCCGATGTTCCAATTTCTGTCCTTTGTAAATACGGTGAGTTGGCCCTTACAATAGTTACCAGTGAGTTTCATGAAAAATTTTTATATGCTGAT
AGATACACTTCTATTCGAAATTCCGCGAAACCGCCGACATTAAAAAACCAATTAATTAAGGAAATTGTGGATGGTATAGCTTATACAGAGACATCATATGAGGCAATCAGAAA
GCAAACTGCAAAACTATGGGATATTGCATTAGGTAAGGTGACCAAAGATAAAATCAATAAAGAAGATACAGCAGCAGCTAGCAAATTTACTTTGAGTTATGAATATCACAGAT
TCAGGCTAATCAATATGGCAGACAATTTAATTGCTAGACTAATGGTGAAACCAAAATCAGATTGGCTAATATCAGTCATGAAGGGGCATCTTAACAGACTATATGAGCACTGG
AAAGTAATGAATGAAATTATCCTAAGTATGGACAACGATTATTCAATTGCAACAACGTTCGAATATTATGCACCATCATGTCTGTGTTTAGCTACGCAGACTTTCCTTATTGTGA
GGAATATGGAAATGGATGATGTCAAGATGATGGTTGCAGTATATAAAAGATTTCTTAACCTAGGAATGTTTTTGCAGAGTGCCAAAGTATGCAGCCTTGCCGATAGTCATACAT
TCAGAGATTTTTCTAGATCTTTTTCCTTTATTACGATAATTTCAAGATTGATGATAATCGAATTTATGCAAATTAAAGAATTGACGAAGGTAGAGTTTATTGAGAAGTTTTCTGAA
GTATGCCCTGACCTTGCAGATCTACCTCCGATGCTTCTAGATCCAAACTCTTGCTTATATTTTTCATTGTTACAGCAGATTAAGAAATCTGGTTTTACGTTGTCATTCAAAAAAATT
CTTGAAGACGCTAGAATGATGGACTTCAATTACGACCGCAATTTGGACTCAGAGGCCATTAAAAAGTGCAATGGTGAATTTAGCAAGTCAATGCCTTCCTGTACCAATGTCTCA
GATACCACCACCGCTGTTTCTGACAATAGTGCTAAGAAGAAAGCTTCAATGGGGTCGGCGAGGGTAAATTCAACTGATACACTAACTGCATCTCCCTTATCGGGCTTAAGGAAT
CAAACGCAGTTGGATTCTAAAGACAGTGTTCCATCTCTCGAGGCTTATACACCAATTGATTCTGTCTCTGACGTGCCCACTGGGGAGATCAACGTTCCATTCCCTCCTGTTTATA
ATCAAAATGGATTGGATCAGCAAACCACTTATAATTTGGGAACTTTAGATGAGTTTGTTAACAAGGGAGATTTGAATGAACTCTATAATAGCCTATGGGGTGACCTATTTTCTG
ATGTTTACTTGTGA

>11

ATGCAAACATTAGAAACTACATCAAAATCAAATCCAGGGGAAGTCAAAGCACAGAAGCCTAGTACAAGAAGAACAAAAGTTGGAAAAGCTTGTGATAGCTGTAGAAGGAGG
AAAATAAAATGTAATGGGCTAAAACCTTGTCCATCTTGTACAATCTATGGTTGTGAATGTACATATACTGATGCAAAATCGACAAAAAATCTCAAATCAAATGATGCAGGTAAA
CCAAAACCAACAGGGAGAGTATCAAAGAATAAAGAAACTACTAGAATCGACAAAGATATTAGGAAATCAGAGCAGCAGTATGTCCCTATTAATGCTAATATTCATGTTGGTCC
CAGGTTCCCCTCCGAGAATATATTGAATGGATATCCACAATGTGGAGCACCACAGAACAATGTTGTGGGTAATCCACTAGCGGTTAATCCTCAATGCCATAGAGGTCTTTCTGA
AACTCCTATGTCCTCAACATTCAAAGAATCTAACTTAAGAGATGATCGGCTACTACAGTCATCAGATACAGATGATATGAGGAATGGTGACTCGGAAGAAAGGGACTTGAAAG
GGAGTGACAGCGAGAATGTCAAAAGTAAAGACAATAAAAGTGATCCTTTGATTATATACAAAGATGATACACATATTGAAAGCACGGTTAATAAACTAACACAGGCAGTTAAT
GAACTCAAATCACTTCAAAATGCACCTAGTTCGATAAAATCATCCATTGACGCCATTGAGTTACAACTTAGAAACATTTTAGACAATTGGAAACCAGAGGTAGATTTCGAGAAA
GCAAAGATTAATGAAAGTGCCACCACTAAGTCACTTGAAACAAACTTGCTGAGGAATAAATACACTAATCACGTTCATCTAACAAGATTTAGGATATGGATAGATTATAAAAAT
GCGAACAAAAACAATCATTTTATGGGAGAGTGTGGATTTAGTCTTGCAGAATCTTTTTTTGCTTCTAATCAGCCATTGGTCGATGAATTGTTTGGGTTGTATTCCCAGGTAGAGG
CCTTTTCTTTGCAAGGTCTTGGTTACTGTGTTCACCTTTATGAGCCATATATGAAAACTGAGGAAGCGATAAAACTGATGAAAGAGACCTTATATATTATACTACGGTTTATTGA
TATATGTGTTCACCATATCAATGAAGAGTCGATATCGATTGCCAACCCGTTAGAAACATATTTACGAAAAAAACATCTAATGCCTATGACTCCTACACCAAGGTCGTCCTATGGA
AGTCCACAAAGTGCTAGTACAAAGAGCTTGGTAAGTAAGATAATAGAGAGAATACCGCAACCGTTTATTGAGAGTGTAACTAATGTGTCGAGTCTTCAACTATTAGATCTTCGA
GATGACGAGTCAAAAATGTTTGGAACATTGCTGAACATGTGTAAGTCTATAAGGCGAAAATTTGACTCTGTTATGAGCGATTACGATTCCATTGTCACAGAAAAATCCGAAGG
CGAACAAAATGATGGTAAAGTAACTGTAGCTGAGTTCACATCTTTGTGTGAAGCGGAAGAAATGCTCTTAGCATTATGCTATAACTATTATAATCTGACGTTATACAGTTTCTTT
GAATTTGGGACTAATATTGAATACATGGAACATCTGTTGCTTCTTCTTGAAGAACAGCTTGCTCTCGACGAATACTATGGTTTTGAAAAGGTCTTGAATGTAGCTGTTGCAAATG
CTAAAAAAATGGGTTTCCACCGTTGGGAGTTTTACGTCGGTTATGAAGAGTCGACTGCTGAAAAGAGGCGGCTACTATGGTGGAAGTTATACAATTATGAAAAAGCCAGTACT

ATGAAGAAGGGTTTTTTTTCTGTGATTGATGATGCTACTGTCAACTGTTTATTACCTAAGATTTTTAGAAACTTTGGCTATCTGGATAGGGTGGAGTTTCTAGAAAATATTCAAA
AGCCAATGGATCTTAGTGTGTTTTCCGATGTTCCAATTTCTGTCCTTTGTAAATACGGTGAGTTGGCCCTTACAATAGTTACCAGTGAGTTTCATGAAAAATTTTTATATGCTGAT
AGATACACTTCTATTCGAAATTCCGCGAAACCGCCGACATTAAAAAACCAATTAATTAAGGAAATTGTGGATGGTATAGCTTATACAGAGACATCATATGAGGCAATCAGAAA
GCAAACTGCAAAACTATGGGATATTGCATTAGGTAAGGTGACCAAAGATAAAATCAATAAAGAAGATACAGCAGCAGCTAGCAAATTTACTTTGAGTTATGAATATCACAGAT
TCAGGCTAATCAATATGGCAGACAATTTAATTGCTAGACTTATGGTGAAACCAAAATCAGATTGGCTAATATCAGTCATGAAGGGGCATCTTAACAGACTATATGAGCACTGGA
AAGTAATGAATGAAATTATCCTAAGTATGGACAACGATTATTCAATTGCAACAACGTTCGAATATTATGCACCATCATGTCTGTGTTTAGCTACGCAGACTTTCCTTATTGTGAG
GAATATGGAAATGGATGATGTCAAGATGATGGTTGCAGTATATAAAAGATTTCTTAACCTAGGAATGTTTCTGCAGAGTGCCAAAGTATGCAGCCTTGCCGATAGTCATACATT
CAGAGATTTTTCTAGATCTTTTTCCTTTATTACGATAATTTCAAGATTGATGATAATCGAATTTATGCAAATTAAAGAATTGACGAAGGTAGAGTTTATTGAGAAGTTTTCTGAAG
TATGCCCTGACCTTGCAGATCTACCTCCGATGCTTCTAGATCCAAACTCTTGCTTATATTTTTCATTGTTACAGCAGATTAAGAAATCTGGTTTTACGTTGTCATTCAAAAAAAATTC
TTGAAGACGCTAGAATGATGGACTTCAATTACGACCGCAATTTGGACTCAGAGGCCATTAAAAAGTGCAATGGTGAATTTAGCAAGTCAATGCCTTCCTGTACCAATGTCTCAG
ATACCACCACCGCTGTTTCTGACAACAGTGCTAAGAAGAAAGCTTCAATGGGGTCGGCGAGGGTAAATTCAACTGATACACTAACTGCATCTCCCTTATCGGGCTTAAGGAATC
AAACGCAGTTGGATTCTAAAGACAGTGTTCCATCTCTCGAGGCTTATACACCAATTGATTCTGTCTCTGACGTACCCACTGGGGAGATCAACGTTCCATTCCCTCCTGTTTATAAT
CAAAATGGATTGGATCAGCAAACCACTTATAATTTGGGAACTTTAGATGAGTTTGTTAACAAGGGAGATTTGAATGAACTCTATAATAGCCTATGGGGTGACCTATTTTCTGAT
GTTTACTTGTGA

>12

ATGCAAACATTAGAAACTACATCAAAATCAAATCCAGGGGAAGTCAAAGCACAGAAGCCTAGTACAAGAAGAACAAAAGTTGGAAAAGCTTGTGATAGCTGTAGAAGGAGG
AAAATAAAATGTAATGGGCTAAAACCTTGTCCATCTTGTACAATCTATGGCTGTGAATGTACATATACTGATGCAAAATCGACAAAAAATCTCAAATCAAATGATGCAGGTAAA
TCAAAACCAACAGGGAGAGTATCAAAGAATAAAGAAACTACTAGAATCGACAAAGATATTAGGAAATCAGAGCAGCAGTATGTCCCTATTAATGCTAATATTCATGTTGGTCC
CAGGTTCCCCTCCGAGAATATATTGAATGGATATCCACAATGTGGAGCACCACAGAACAATGTTGTGGGTAATCCACTAGCGGTTAATACTCAATGCCATAGAGGTCTTTCTGA
AACTCCTATGTCCTCAACATTCAAAGAATCTAACTTAAGAGATGATCGGCTACTACAGTCATCAGATACAGATGATATGAGGAATGGTGACTCGGAAGAAAGGGACTTGAAAG
GGAGTGACAGCGAGAATGTCAAAAGTAAAGACAATAAAAGTGATCCTTTGATTATATACAAAGATGATACACATATTGAAAGCACGGTTAATAAACTAACACAGGCAGTTAAT
GAACTCAAATCACTTCAAAATGCACCCAGTTCGATAAAATCATCCATTAACGCCATTGAGTTACAACTTAGAAACATTTTAGACAACTGGAAACCAGAGGTAGATTTCGAGAAA
GCAAAGATTAATGAAAGTGCCACCACTAAGTCACTTGAAACAAACTTGCTGAGGAATAAATACACTAATCACGTTCATTTAACAAGATTTAGGATATGGATAGATTATAAAAAT
GCGAACAAAAACAATCATTTTATGGGAGAGTGTGGATTTAGTCTTGCAGAATCTTTTTTTGCTTCTAATCAGCCATTGGTCGATGAATTGTTTGGGTTGTATTCCCAGGTAGAGG
CCTTTTCTTTGCAAGGTCTTGGTTACTGTGTTCACCTTTATGAGCCATATATGAAAACTGAGGAAGCGATAAAACTGATGAAAGAGACCTTATATATTATACTACGGTTTATTGA
TATATGTGTTCACCATATCAATGAAGAGTCGATATCGATTGCCAACCCGTTAGAAACATATTTACGAAAAAAACATCTAATGCCTATGACTCCTACACCAAGGTCGTCCTATGGA
AGTCCACAAAGTGCTAGTACAAAGAGCTTGGTAAGTAAGATAATAGAGAGAATACCGCAACCGTTTATTGAGAGTGTAACTAATGTGTCGAGTCTTCAACTATTAGATCTTCGA
GATGACGAGTCAAAAATGTTTGGAACATTGCTGAACATGTGTAAGTCTATAAGGCGAAAATTTGACTCTGTTATGAGCGATTACGATTCCATTGTCACAGAAAAATCCGAAGG
CGAACAAAATGATGGTAAAGTAACTGTAGCTGAGTTCACATCTTTGTGTGAAGCGGAAGAAATGCTCTTAGCATTATGCTATAACTATTATAATCTGACGTTATACAGTTTCTTT
GAATTTGGGACTAATATTGAATACATGGAACATCTGTTGCTTCTTCTTGAAGAACAGCTTGCTCTCGACGAATACTATGGTTTTGAAAAGGTCTTGAATGTAGCTGTTGCAAATG

CTAAAAAAATGGGTTTCCACCGTTGGGAGTTTTACGTCGGCTATGAAGAGTCGACTGCTGAAAAGAGGCGGCTACTATGGTGGAAGTTATACAATTATGAAAAAGCCAGTACT
ATGAAGAAGGGTTTTTTTTCTGTGATTGATGATGCTACTGTCAACTGTTTATTACCTAAGATTTTTAGAAACTTTGGCTATCTGGATAGGGTGGAGTTTCTAGAAAATATTCAAA
AGCCAATGGATCTTAGTGTGTTTTCCGATGTTCCAATTTCTGTCCTTTGTAAATACGGTGAGTTGGCCCTTACAATAGTTACCAGTGAGTTTCATGAAAAATTTTTATATGCTGAT
AGATACACTTCTATTCGAAATTCCGCGAAACCGCCGACATTAAAAAACCAATTAATTAAGGAAATTGTGGATGGTATAGCTTATACAGAGACATCATATGAGGCAATCAGAAA
GCAAACTGCAAAACTATGGGATATTGCATTAGGTAAGGTGACCAAAGATAAAATCAATAAAGAAGATACAGCAGCAGCTAGCAAATTTACTTTGAGTTATGAATATCACAGAT
TCAGGCTAATCAATATGGCAGACAATTTAATTGCTAGACTAATGGTGAAACCAAAATCAGATTGGCTAATATCAGTCATGAAGGGGCATCTTAACAGACTATATGAGCACTGG
AAAGTAATGAATGAAATTATCCTAAGTATGGACAACGATTATTCAATTGCAACAACGTTCGAATATTATGCACCATCATGTCTGTGTTTAGCTACGCAGACTTTCCTTATTGTGA
GGAATATGGAAATGGATGATGTCAAGATGATGGTTGCAGTATATAAAAGATTTCTTAACCTAGGAATGTTTTTGCAGAGTGCCAAAGTATGCAGCCTTGCCGATAGTCATACAT
TCAGAGATTTTTCTAGATCTTTTTCCTTTATTACGATAATTTCAAGATTGATGATAATCGAATTTATGCAAATTAAAGAATTGACGAAGGTAGAGTTTATTGAGAAGTTTTCTGAA
GTATGCCCTGACCTTGCAGATCTACCTCCGATGCTTCTAGATCCAAACTCTTGCTTATATTTTTCATTGTTACAGCAGATTAAGAAATCTGGTTTTACGTTGTCATTCAAAAAAATT
CTTGAAGACGCTAGAATGATGGACTTCAATTACGACCGCAATTTGGACTCAGAGGCCATTAAAAAGTGCAATGGTGAATTTAGCAAGTCAATGCCTTCCTGTACCAATGTCTCA
GATACCACCACCGCTGTTTCTGACAATAGTGCTAAGAAGAAAGCTTCAATGGGGTCGGCGAGGGTAAATTCAACTGATACACTAACTGCATCTCCCTTATCGGGCTTAAGGAAT
CAAACGCAGTTGGATTCTAAAGACAGTGTTCCATCTCTCGAGGCTTATACACCAATTGATTCTGTCTCTGACGTGCCCACTGGGGAGATCAACGTTCCATTCCCTCCTGTTTATA
ATCAAAATGGATTGGATCAGCAAACCACTTATAATTTGGGAACTTTAGATGAGTTTGTTAACAAGGGAGATTTGAATGAACTCTATAATAGCCTATGGGGTGACCTATTTTCTG
ATGTTTACTTGTGA

>13

ATGCAAACATTAGAAACTACATCAAAATCAAATCCAGGGGAAGTCAAAGCACAGAAGCCTAGTACAAGAAGAACAAAAGTTGGAAAAGCTTGTGATAGCTGTAGAAGGAGG
AAAATAAAATGTAATGGGCTAAAACCTTGTCCATCTTGTACAATCTATGGTTGTGAATGTACATATACTGATGCAAAATCGACAAAAAATCTCAAATCAAATGATGCAGGTAAA
CCAAAACCAACAGGGAGAGTATCAAAGAATAAAGAAACTACTAGAATCGACAAAGATATTAGGAAATCAGAGCAGCAGTATGTACCTATTAATGCTAATATTCATGTTGGTCC
CAGGTTCCCCTCCGAGAATATATTGAATGGATATCCACAATGTGGAGCACCACAGAACAATGTTGTGGGTAATCCACTAGCGGTTAATCCTCAATGCCATAGAGGTCTTTCTGA
AACTCCTATGTCCTCAACATTCAAAGAATCTAACTTAAGAGATGATCGGCTACTACAGTCATCAGATACAGATGATATGAGGAATGGTGACTCGGAAGAAAGGGACTTGAAAG
GGAGTGACAGCGAGAATGTCAAAAGTAAAGACAATAAAAGTGATCCTTTGATTATATACAAAGATGATACACATATTGAAAGCACGGTTAATAAACTAACACAGGCAGTTAAT
GAACTCAAATCACTTCAAAATGCACCTAGTTCGATAAAATCATCCATTGACGCCATTGAGTTACAACTTAGAAACATTTTAGACAATTGGAAACCAGAGGTAGATTTCGAGAAA
GCAAAGATTAATGAAAGTGCCACCACTAAGTCACTTGAAACAAACTTGCTGAGGAATAAATACACTAATCACGTTCATCTAACAAGATTTAGGATATGGATAGATTATAAAAAT
GCGAACAAAAACAATCATTTTATGGGAGAGTGTGGATTTAGTCTTGCAGAATCTTTTTTTGCTTCTAATCAGCCATTGGTCGATGAATTGTTTGGGTTGTATTCCCAGGTAGAGG
CCTTTTCTTTGCAAGGTCTTGGTTACTGTGTTCACCTTTATGAGCCATATATGAAAACTGAGGAAGCGATAAAACTGATGAAAGAGACCTTATATATTATACTACGGTTTATTGA
TATATGTGTTCACCATATCAATGAAGAGTCGATATCGATTGCCAACCCGTTAGAAACATATTTACGAAAAAAACATCTAATGCCTATGACTCCTACACCAAGGTCGTCCTATGGA
AGTCCACAAAGTGCTAGTACAAAGAGCTTGGTAAGTAAGATAATAGAGAGAATACCGCAACCGTTTATTGAGAGTGTAACTAATGTGTCGAGTCTTCAACTATTAGATCTTCGA
GATGACGAGTCAAAAATGTTTGGAACATTGCTGAACATGTGTAAGTCTATAAGGCGAAAATTTGACTCTGTTATGAGCGATTACGATTCCATTGTCACAGAAAAATCCGAAGG
CGAACAAAATGATGGTAAAGTAACTGTAGCTGAGTTCACATCTTTGTGTGAAGCGGAAGAAATGCTCTTAGCATTATGCTATAACTATTATAATCTGACGTTATACAGTTTCTTT

GAATTTGGGACTAATATTGAATACATGGAACATCTGTTGCTTCTTCTTGAAGAACAGCTTGCTCTCGACGAATACTATGGTTTTGAAAAGGTCTTGAATGTAGCTGTTGCAAATG
CTAAAAAAATGGGTTTCCACCGTTGGGAGTTTTACGTCGGTTATGAAGAGTCGACTGCTGAAAAGAGGCGGCTACTATGGTGGAAGTTATACAATTATGAAAAAGCCAGTACT
ATGAAGAAGGGTTTTTTTTCTGTGATTGATGATGCTACTGTCAACTGTTTATTACCTAAGATTTTTAGAAACTTTGGCTATCTGGATAGGGTGGAGTTTCTAGAAAATATTCAAA
AGCCAATGGATCTTAGTGTGTTTTCCGATGTTCCAATTTCTGTCCTTTGTAAATACGGTGAGTTGGCCCTTACAATAGTTACCAGTGAGTTTCATGAAAAATTTTTATATGCTGAT
AGATACACTTCTATTCGAAATTCCGCGAAACCGCCGACATTAAAAAACCAATTAATTAAGGAAATTGTGGATGGTATAGCTTATACAGAGACATCATATGAGGCAATCAGAAA
GCAAACTGCAAAACTATGGGATATTGCATTAGGTAAGGTGACCAAAGATAAAATCAATAAAGAAGATACAGCAGCAGCTAGCAAATTTACTTTGAGTTATGAATATCACAGAT
TCAGGCTAATCAATATGGCAGACAATTTAATTGCTAGACTTATGGTGAAACCAAAATCAGATTGGCTAATATCAGTCATGAAGGGGCATCTTAACAGACTATATGAGCACTGGA
AAGTAATGAATGAAATTATCCTAAGTATGGACAACGATTATTCAATTGCAACAACGTTCGAATATTATGCACCATCATGTCTGTGTTTAGCTACGCAGACTTTCCTTATTGTGAG
GAATATGGAAATGGATGATGTCAAGATGATGGTTGCAGTATATAAAAGATTTCTTAACCTAGGAATGTTTCTGCAGAGTGCCAAAGTATGCAGCCTTGCCGATAGTCATACATT
CAGAGATTTTTCTAGATCTTTTTCCTTTATTACGATAATTTCAAGATTGATGATAATCGAATTTATGCAAATTAAAGAATTGACGAAGGTAGAGTTTATTGAGAAGTTTTCTGAAG
TATGCCCTGACCTTGCAGATCTACCTCCGATGCTTCTAGATCCAAACTCTTGCTTATATTTTTCATTGTTACAGCAGATTAAGAAATCTGGTTTTACGTTGTCATTCAAAAAAAATTC
TTGAAGACGCTAGAATGATGGACTTCAATTACGACCGCAATTTGGACTCAGAGGCCATTAAAAAGTGCAATGGTGAATTTAGCAAGTCAATGCCTTCCTGTACCAATGTCTCAG
ATACCACCACCGCTGTTTCTGACAACAGTGCTAAGAAGAAAGCTTCAATGGGGTCGGCGAGGGTAAATTCAACTGATACACTAACTGCATCTCCCTTATCGGGCTTAAGGAATC
AAACGCAGTTGGATTCTAAAGACAGTGTTCCATCTCTCGAGGCTTATACACCAATTGATTCTGTCTCTGACGTACCCACTGGGGAGATCAACGTTCCATTCCCTCCTGTTTATAAT
CAAAATGGATTGGATCAGCAAACCACTTATAATTTGGGAACTTTAGATGAGTTTGTTAACAAGGGAGATTTGAATGAACTCTATAATAGCCTATGGGGTGACCTATTTTCTGAT
GTTTACTTGTGA

>14

ATGCAAACATTAGAAACTACATCAAAATCAAATCCAGGGGAAGTCAAAGCACAGAAGCCTAGTACAAGAAGAACAAAAGTTGGAAAAGCTTGTGATAGCTGTAGAAGGAGG
AAAATAAAATGTAATGGGCTAAAACCTTGTCCATCTTGTACAATCTATGGTTGTGAATGTACATATACTGATGCAAAATCGACAAAAAATCTCAAATCAAATGATGCAGGTAAA
CCAAAACCAACAGGGAGAGTATCAAAGAATAAAGAAACTACTAGAATCGACAAAGATATTAGGAAATCAGAGCAGCAGTATGTCCCTATTAATGCTAATATTCATGTTGGTCC
CAGGTTCCCCTCCGAGAATATATTGAATGGATATCCACAATGTGGAGCACCACAGAACAATGTTGTGGGTAATCCACTAGCGGTTAATCCTCAATGCCATAGAGGTCTTTCTGA
AACTCCTATGTCCTCAACATTCAAAGAATCTAACTTAAGAGATGATCGGCTACTACAGTCATCAGATACAGATGATATGAGGAATGGTGACTCGGAAGAAAGGGACTTGAAAG
GGAGTGACAGCGAGAATGTCAAAAGTAAAGACAATAAAAGTGATCCTTTGATTATATACAAAGATGATACACATATTGAAAGCACGGTTAATAAACTAACACAGGCAGTTAAT
GAACTCAAATCACTTCAAAATGCACCTAGTTCGATAAAATCATCCATTGACGCCATTGAGTTACAACTTAGAAACATTTTAGACAATTGGAAACCAGAGGTAGATTTCGAGAAA
GCAAAGATTAATGAAAGTGCCACCACTAAGTCACTTGAAACAAACTTGCTGAGGAATAAATACACTAATCACGTTCATCTAACAAGATTTAGGATATGGATAGATTATAAAAAT
GCGAACAAAAACAATCATTTTATGGGAGAGTGTGGATTTAGTCTTGCAGAATCTTTTTTTGCTTCTAATCAGCCATTGGTCGATGAATTGTTTGGGTTGTATTCCCAGGTAGAGG
CCTTTTCTTTGCAAGGTCTTGGTTACTGTGTTCACCTTTATGAGCCATATATGAAAACTGAGGAAGCGATAAAACTGATGAAAGAGACCTTATATATTATACTACGGTTTATTGA
TATATGTGTTCACCATATCAATGAAGAGTCGATATCGATTGCCAACCCGTTAGAAACATATTTACGAAAAAAACATCTAATGCCTATGACTCCTACACCAAGGTCGTCCTATGGA
AGTCCACAAAGTGCTAGTACAAAGAGCTTGGTAAGTAAGATAATAGAGAGAATACCGCAACCGTTTATTGAGAGTGTAACTAATGTGTCGAGTCTTCAACTATTAGATCTTCGA
GATGACGAGTCAAAAATGTTTGGAACATTGCTGAACATGTGTAAGTCTATAAGGCGAAAATTTGACTCTGTTATGAGCGATTACGATTCCATTGTCACAGAAAAATCCGAAGG

CGAACAAAATGATGGTAAAGTAACTGTAGCTGAGTTCACATCTTTGTGTGAAGCGGAAGAAATGCTCTTAGCATTATGCTATAACTATTATAATCTGACGTTATACAGTTTCTTT
GAATTTGGGACTAATATTGAATACATGGAACATCTGTTGCTTCTTCTTGAAGAACAGCTTGCTCTCGACGAATACTATGGTTTTGAAAAGGTCTTGAATGTAGCTGTTGCAAATG
CTAAAAAAATGGGTTTCCACCGTTGGGAGTTTTACGTCGGTTATGAAGAGTCGACTGCTGAAAAGAGGCGGCTACTATGGTGGAAGTTATACAATTATGAAAAAGCCAGTACT
ATGAAGAAGGGTTTTTTTTCTGTGATTGATGATGCTACTGTCAACTGTTTATTACCTAAGATTTTTAGAAACTTTGGCTATCTGGATAGGGTGGAGTTTCTAGAAAATATTCAAA
AGCCAATGGATCTTAGTGTGTTTTCCGATGTTCCAATTTCTGTCCTTTGTAAATACGGTGAGTTGGCCCTTACAATAGTTACCAGTGAGTTTCATGAAAAATTTTTATATGCTGAT
AGATACACTTCTATTCGAAATTCCGCGAAACCGCCGACATTAAAAAACCAATTAATTAAGGAAATTGTGGATGGTATAGCTTATACAGAGACATCATATGAGGCAATCAGAAA
GCAAACTGCAAAACTATGGGATATTGCATTAGGTAAGGTGACCAAAGATAAAATCAATAAAGAAGATACAGCAGCAGCTAGCAAATTTACTTTGAGTTATGAATATCACAGAT
TCAGGCTAATCAATATGGCAGACAATTTAATTGCTAGACTTATGGTGAAACCAAAATCAGATTGGCTAATATCAGTCATGAAGGGGCATCTTAACAGACTATATGAGCACTGGA
AAGTAATGAATGAAATTATCCTAAGTATGGACAACGATTATTCAATTGCAACAACGTTCGAATATTATGCACCATCATGTCTGTGTTTAGCTACGCAGACTTTCCTTATTGTGAG
GAATATGGAAATGGATGATGTCAAGATGATGGTTGCAGTATATAAAAGATTTCTTAACCTAGGAATGTTTCTGCAGAGTGCCAAAGTATGCAGCCTTGCCGATAGTCATACATT
CAGAGATTTTTCTAGATCTTTTTCCTTTATTACGATAATTTCAAGATTGATGATAATCGAATTTATGCAAATTAAAGAATTGACGAAGGTAGAGTTTATTGAGAAGTTTTCTGAAG
TATGCCCTGACCTTGCAGATCTACCTCCGATGCTTCTAGATCCAAACTCTTGCTTATATTTTTCATTGTTACAGCAGATTAAGAAATCTGGTTTTACGTTGTCATTCAAAAAAATTC
TTGAAGACGCTAGAATGATGGACTTCAATTACGACCGCAATTTGGACTCAGAGGCCATTAAAAAGTGCAATGGTGAATTTAGCAAGTCAATGCCTTCCTGTACCAATGTCTCAG
ATACCACCACCGCTGTTTCTGACAACAGTGCTAAGAAGAAAGCTTCAATGGGGTCGGCGAGGGTAAATTCAACTGATACACTAACTGCATCTCCCTTATCGGGCTTAAGGAATC
AAACGCAGTTGGATTCTAAAGACAGTGTTCCATCTCTCGAGGCTTATACACCAATTGATTCTGTCTCTGACGTACCCACTGGGGAGATCAACGTTCCATTCCCTCCTGTTTATAAT
CAAAATGGATTGGATCAGCAAACCACTTATAATTTGGGAACTTTAGATGAGTTTGTTAACAAGGGAGATTTGAATGAACTCTATAATAGCCTATGGGGTGACCTATTTTCTGAT
GTTTACTTGTGA

>15

ATGCAAACATTAGAAACTACATCAAAATCAAATCCAGGGGAAGTCAAAGCACAGAAGCCTAGTACAAGAAGAACAAAAGTTGGAAAAGCTTGTGATAGCTGTAGAAGGAGG
AAAATAAAATGTAATGGGCTAAAACCTTGTCCATCTTGTACAATCTATGGTTGTGAATGTACATATACTGATGCAAAATCGACAAAAAATCTCAAATCAAATGATGCAGGTAAA
CCAAAACCAACAGGGAGAGTATCAAAGAATAAAGAAACTACTAGAATCGACAAAGATATTAGGAAATCAGAGCAGCAGTATGTACCTATTAATGCTAATATTCATGTTGGTCC
CAGGTTCCCCTCCGAGAATATATTGAATGGATATCCACAATGTGGAGCACCACAGAACAATGTTGTGGGTAATCCACTAGCGGTTAATCCTCAATGCCATAGAGGTCTTTCTGA
AACTCCTATGTCCTCAACATTCAAAGAATCTAACTTAAGAGATGATCGGCTACTACAGTCATCAGATACAGATGATATGAGGAATGGTGACTCGGAAGAAAGGGACTTGAAAG
GGAGTGACAGCGAGAATGTCAAAAGTAAAGACAATAAAAGTGATCCTTTGATTATATACAAAGATGATACACATATTGAAAGCACGGTTAATAAACTAACACAGGCAGTTAAT
GAACTCAAATCACTTCAAAATGCACCTAGTTCGATAAAATCATCCATTGACGCCATTGAGTTACAACTTAGAAACATTTTAGACAATTGGAAACCAGAGGTAGATTTCGAGAAA
GCAAAGATTAATGAAAGTGCCACCACTAAGTCACTTGAAACAAACTTGCTGAGGAATAAATACACTAATCACGTTCATCTAACAAGATTTAGGATATGGATAGATTATAAAAAT
GCGAACAAAAACAATCATTTTATGGGAGAATGTGGATTTAGTCTTGCAGAATCTTTTTTTGCTTCTAATCAGCCATTGGTCGATGAATTGTTTGGGTTGTATTCCCAGGTAGAGG
CCTTTTCTTTGCAAGGTCTTGGTTACTGTGTTCACCTTTATGAGCCATATATGAAAACTGAGGAAGCGATAAAACTGATGAAAGAGACCTTATATATTATACTACGGTTTATTGA
TATATGTGTTCACCATATCAATGAAGAGTCGATATCGATTGCCAACCCGTTAGAAACATATTTACGAAAAAAACATCTAATGCCTATGACTCCTACACCAAGGTCGTCCTATGGA
AGTCCACAAAGTGCTAGTACAAAGAGCTTGGTAAGTAAGATAATAGAGAGAATACCGCAACCGTTTATTGAGAGTGTAACTAATGTGTCGAGTCTTCAACTATTAGATCTTCGA

GATGACGAGTCAAAAATGTTTGGAACATTGCTGAACATGTGTAAGTCTATAAGGCGAAAATTTGACTCTGTTATGAGCGATTACGATTCCATTGTCACAGAAAAATCCGAAGAC
GAACAAAATGATGGTAAAGTAACTGTAGCTGAGTTCACATCTTTGTGTGAAGCGGAAGAAATGCTCTTAGCATTATGCTATAACTATTATAATCTGACGTTATACAGTTTCTTTG
AATTTGGGACTAATATTGAATACATGGAACATCTGTTGCTTCTTCTTGAAGAACAGCTTGCTCTCGACGAATACTATGGTTTTGAAAAGGTCTTGAATGTAGCTGTTGCAAATGC
TAAAAAAATGGGTTTCCACCGTTGGGAGTTTTACGTCGGTTATGAAGAGTCGACTGCTGAAAAGAGGCGGCTACTATGGTGGAAGTTATACAATTATGAAAAAGCCAGTACTA
TGAAGAAGGGTTTTTTTTCTGTGATTGATGATGCTACTGTCAACTGTTTATTACCTAAGATTTTTAGAAACTTTGGCTATCTGGATAGGGTGGAGTTTCTAGAAAATATTCAAAA
GCCAATGGATCTTAGTGTGTTTTCCGATGTTCCAATTTCTGTCCTTTGTAAATATGGTGAGTTGGCCCTTACAATAGTTACCAGTGAGTTTCATGAAAAATTTTTATATGCTGATA
GATACACTTCTATTCGAAATTCCGCGAAACCGCCGACATTAAAAAACCAATTAATTAAGGAAATTGTGGATGGTATAGCTTATACAGAGACATCATATGAGGCAATCAGAAAGC
AAACTGCAAAACTATGGGATATTGCATTAGGTAAGGTGACCAAAGATAAAATCAATAAAGAAGATACAGCAGCAGCTAGCAAATTTACTTTGAGTTATGAATATCACAGATTC
AGGCTAATCAATATGGCAGACAATTTAATTGCTAGACTTATGGTGAAACCAAAATCAGATTGGCTAATATCAGTCATGAAGGGGCATCTTAACAGACTATATGAGCACTGGAA
AGTAATGAATGAAATTATCCTAAGTATGGACAACGATTATTCAATTGCAACAACGTTCGAATATTATGCACCATCATGTCTGTGTTTAGCTACGCAGACTTTCCTTATTGTGAGG
AATATGGAAATGGATGATGTCAAGATGATGGTTGCAGTATATAAAAGATTTCTTAACCTAGGAATGTTTCTGCAGAGTGCCAAAGTATGCAGCCTTGCCGATAGTCATACATTC
AGAGATTTTTCTAGATCTTTTTCCTTTATTACGATAATTTCAAGATTGATGATAATCGAATTTATGCAAATTAAAGAATTGACGAAGGTAGAGTTTATTGAGAAGTTTTCTGAAGT
ATGCCCTGACCTTGCAGATCTACCTCCGATGCTTCTAGATCCAAACTCTTGCTTATATTTTTCATTGTTACAGCAGATTAAGAAATCTGGTTTTACGTTGTCATTCAAAAAAATTCT
TGAAGACGCTAGAATGATGGACTTCAATTACGACCGCAATTTGGACTCAGAGGCCATTAAAAAGTGCAATGGTGAATTTAGCAAGTCAATGCCTTCCTGTACCAATGTCTCAGA
TACCACCACCGCTGTTTCTGACAACAGTGCTAAGAAGAAAGCTTCAATGGGGTCGGCGAGGGTAAATTCAACTGATACACTAACTGCATCTCCCTTATCGGGCTTAAGGAATCA
AACGCAGTTGGATTCTAAAGACAGTGTTCCATCTCTCGAGGCTTATACACCAATTGATTCTGTCTCTGACGTACCCACTGGGGAGATCAACGTTCCATTCCCTCCTGTTTATAATC
AAAATGGATTGGATCAGCAAACCACTTATAATTTGGGAACTTTAGATGAGTTTGTTAACAAGGGAGATTTGAATGAACTCTATAATAGCCTATGGGGTGACCTATTTTCTGATG
TTTACTTGTGA

>16

ATGCAAACATTAGAAACTACATCAAAATCAAATCCAGGGGAAGTCAAAGCACAGAAGCCTAGTACAAGAAGAACAAAAGTTGGAAAAGCTTGTGATAGCTGTAGAAGGAGG
AAAATAAAATGTAATGGGCTAAAACCTTGTCCATCTTGTACAATCTATGGTTGTGAATGTACATATACTGATGCAAAATCGACAAAAAATCTCAAATCAAATGATGCAGGTAAA
CCAAAACCAACAGGGAGAGTATCAAAGAATAAAGAAACTACTAGAATCGACAAAGATATTAGGAAATCAGAGCAGCAGTATGTCCCTATTAATGCTAATATTCATGTTGGTCC
CAGGTTCCCCTCCGAGAATATATTGAATGGATATCCACAATGTGGAGCACCACAGAACAATGTTGTGGGTAATCCACTAGCGGTTAATCCTCAATGCCATAGAGGTCTTTCTGA
AACTCCTATGTCCTCAACATTCAAAGAATCTAACTTAAGAGATGATCGGCTACTACAGTCATCAGATACAGATGATATGAGGAATGGTGACTCGGAAGAAAGGGACTTGAAAG
GGAGTGACAGCGAGAATGTCAAAAGTAAAGACAATAAAAGTGATCCTTTGATTATATACAAAGATGATACACATATTGAAAGCACGGTTAATAAACTAACACAGGCAGTTAAT
GAACTCAAATCACTTCAAAATGCACCTAGTTCGATAAAATCATCCATTGACGCCATTGAGTTACAACTTAGAAACATTTTAGACAATTGGAAACCAGAGGTAGATTTCGAGAAA
GCAAAGATTAATGAAAGTGCCACCACTAAGTCACTTGAAACAAACTTGCTGAGGAATAAATACACTAATCACGTTCATCTAACAAGATTTAGGATATGGATAGATTATAAAAAT
GCGAACAAAAACAATCATTTTATGGGAGAGTGTGGATTTAGTCTTGCAGAATCTTTTTTTGCTTCTAATCAGCCATTGGTCGATGAATTGTTTGGGTTGTATTCCCAGGTAGAGG
CCTTTTCTTTGCAAGGTCTTGGTTACTGTGTTCACCTTTATGAGCCATATATGAAAACTGAGGAAGCGATAAAACTGATGAAAGAGACCTTATATATTATACTACGGTTTATTGA
TATATGTGTTCACCATATCAATGAAGAGTCGATATCGATTGCCAACCCGTTAGAAACATATTTACGAAAAAAACATCTAATGCCTATGACTCCTACACCAAGGTCGTCCTATGGA

AGTCCACAAAGTGCTAGTACAAAGAGCTTGGTAAGTAAGATAATAGAGAGAATACCGCAACCGTTTATTGAGAGTGTAACTAATGTGTCGAGTCTTCAACTATTAGATCTTCGA
GATGACGAGTCAAAAATGTTTGGAACATTGCTGAACATGTGTAAGTCTATAAGGCGAAAATTTGACTCTGTTATGAGCGATTACGATTCCATTGTCACAGAAAAATCCGAAGG
CGAACAAAATGATGGTAAAGTAACTGTAGCTGAGTTCACATCTTTGTGTGAAGCGGAAGAAATGCTCTTAGCATTATGCTATAACTATTATAATCTGACGTTATACAGTTTCTTT
GAATTTGGGACTAATATTGAATACATGGAACATCTGTTGCTTCTTCTTGAAGAACAGCTTGCTCTCGACGAATACTATGGTTTTGAAAAGGTCTTGAATGTAGCTGTTGCAAATG
CTAAAAAAATGGGTTTCCACCGTTGGGAGTTTTACGTCGGTTATGAAGAGTCGACTGCTGAAAAGAGGCGGCTACTATGGTGGAAGTTATACAATTATGAAAAAGCCAGTACT
ATGAAGAAGGGTTTTTTTTCTGTGATTGATGATGCTACTGTCAACTGTTTATTACCTAAGATTTTTAGAAACTTTGGCTATCTGGATAGGGTGGAGTTTCTAGAAAATATTCAAA
AGCCAATGGATCTTAGTGTGTTTTCCGATGTTCCAATTTCTGTCCTTTGTAAATACGGTGAGTTGGCCCTTACAATAGTTACCAGTGAGTTTCATGAAAAATTTTTATATGCTGAT
AGATACACTTCTATTCGAAATTCCGCGAAACCGCCGACATTAAAAAACCAATTAATTAAGGAAATTGTGGATGGTATAGCTTATACAGAGACATCATATGAGGCAATCAGAAA
GCAAACTGCAAAACTATGGGATATTGCATTAGGTAAGGTGACCAAAGATAAAATCAATAAAGAAGATACAGCAGCAGCTAGCAAATTTACTTTGAGTTATGAATATCACAGAT
TCAGGCTAATCAATATGGCAGACAATTTAATTGCTAGACTTATGGTGAAACCAAAATCAGATTGGCTAATATCAGTCATGAAGGGGCATCTTAACAGACTATATGAGCACTGGA
AAGTAATGAATGAAATTATCCTAAGTATGGACAACGATTATTCAATTGCAACAACGTTCGAATATTATGCACCATCATGTCTGTGTTTAGCTACGCAGACTTTCCTTATTGTGAG
GAATATGGAAATGGATGATGTCAAGATGATGGTTGCAGTATATAAAAGATTTCTTAACCTAGGAATGTTTCTGCAGAGTGCCAAAGTATGCAGCCTTGCCGATAGTCATACATT
CAGAGATTTTTCTAGATCTTTTTCCTTTATTACGATAATTTCAAGATTGATGATAATCGAATTTATGCAAATTAAAGAATTGACGAAGGTAGAGTTTATTGAGAAGTTTTCTGAAG
TATGCCCTGACCTTGCAGATCTACCTCCGATGCTTCTAGATCCAAACTCTTGCTTATATTTTTCATTGTTACAGCAGATTAAGAAATCTGGTTTTACGTTGTCATTCAAAAAAAATTC
TTGAAGACGCTAGAATGATGGACTTCAATTACGACCGCAATTTGGACTCAGAGGCCATTAAAAAGTGCAATGGTGAATTTAGCAAGTCAATGCCTTCCTGTACCAATGTCTCAG
ATACCACCACCGCTGTTTCTGACAACAGTGCTAAGAAGAAAGCTTCAATGGGGTCGGCGAGGGTAAATTCAACTGATACACTAACTGCATCTCCCTTATCGGGCTTAAGGAATC
AAACGCAGTTGGATTCTAAAGACAGTGTTCCATCTCTCGAGGCTTATACACCAATTGATTCTGTCTCTGACGTACCCACTGGGGAGATCAACGTTCCATTCCCTCCTGTTTATAAT
CAAAATGGATTGGATCAGCAAACCACTTATAATTTGGGAACTTTAGATGAGTTTGTTAACAAGGGAGATTTGAATGAACTCTATAATAGCCTATGGGGTGACCTATTTTCTGAT
GTTTACTTGTGA

>17

ATGCAAACATTAGAAACTACATCAAAATCAAATCCAGGGGAAGTCAAAGCACAGAAGCCTAGTACAAGAAGAACAAAAGTTGGAAAAGCTTGTGATAGCTGTAGAAGGAGG
AAAATAAAATGTAATGGGCTAAAACCTTGTCCATCTTGTACAATCTATGGTTGTGAATGTACATATACTGATGCAAAATCGACAAAAAATCTCAAATCAAATGATGCAGGTAAA
CCAAAACCAACAGGGAGAGTATCAAAGAATAAAGAAACTACTAGAATCGACAAAGATATTAGGAAATCAGAGCAGCAGTATGTACCTATTAATGCTAATATTCATGTTGGTCC
CAGGTTCCCCTCCGAGAATATATTGAATGGATATCCACAATGTGGAGCACCACAGAACAATGTTGTGGGTAATCCACTAGCGGTTAATCCTCAATGCCATAGAGGTCTTTCTGA
AACTCCTATGTCCTCAACATTCAAAGAATCTAACTTAAGAGATGATCGGCTACTACAGTCATCAGATACAGATGATATGAGGAATGGTGACTCGGAAGAAAGGGACTTGAAAG
GGAGTGACAGCGAGAATGTCAAAAGTAAAGACAATAAAAGTGATCCTTTGATTATATACAAAGATGATACACATATTGAAAGCACGGTTAATAAACTAACACAGGCAGTTAAT
GAACTCAAATCACTTCAAAATGCACCTAGTTCGATAAAATCATCCATTGACGCCATTGAGTTACAACTTAGAAACATTTTAGACAATTGGAAACCAGAGGTAGATTTCGAGAAA
GCAAAGATTAATGAAAGTGCCACCACTAAGTCACTTGAAACAAACTTGCTGAGGAATAAATACACTAATCACGTTCATCTAACAAGATTTAGGATATGGATAGATTATAAAAAT
GCGAACAAAAACAATCATTTTATGGGAGAGTGTGGATTTAGTCTTGCAGAATCTTTTTTTGCTTCTAATCAGCCATTGGTCGATGAATTGTTTGGGTTGTATTCCCAGGTAGAGG
CCTTTTCTTTGCAAGGTCTTGGTTACTGTGTTCACCTTTATGAGCCATATATGAAAACTGAGGAAGCGATAAAACTGATGAAAGAGACCTTATATATTATACTACGGTTTATTGA

TATATGTGTTCACCATATCAATGAAGAGTCGATATCGATTGCCAACCCGTTAGAAACATATTTACGAAAAAAACATCTAATGCCTATGACTCCTACACCAAGGTCGTCCTATGGA
AGTCCACAAAGTGCTAGTACAAAGAGCTTGGTAAGTAAGATAATAGAGAGAATACCGCAACCGTTTATTGAGAGTGTAACTAATGTGTCGAGTCTTCAACTATTAGATCTTCGA
GATGACGAGTCAAAAATGTTTGGAACATTGCTGAACATGTGTAAGTCTATAAGGCGAAAATTTGACTCTGTTATGAGCGATTACGATTCCATTGTCACAGAAAAATCCGAAGG
CGAACAAAATGATGGTAAAGTAACTGTAGCTGAGTTCACATCTTTGTGTGAAGCGGAAGAAATGCTCTTAGCATTATGCTATAACTATTATAATCTGACGTTATACAGTTTCTTT
GAATTTGGGACTAATATTGAATACATGGAACATCTGTTGCTTCTTCTTGAAGAACAGCTTGCTCTCGACGAATACTATGGTTTTGAAAAGGTCTTGAATGTAGCTGTTGCAAATG
CTAAAAAAATGGGTTTCCACCGTTGGGAGTTTTACGTCGGTTATGAAGAGTCGACTGCTGAAAAGAGGCGGCTACTATGGTGGAAGTTATACAATTATGAAAAAGCCAGTACT
ATGAAGAAGGGTTTTTTTTCTGTGATTGATGATGCTACTGTCAACTGTTTATTACCTAAGATTTTTAGAAACTTTGGCTATCTGGATAGGGTGGAGTTTCTAGAAAATATTCAAA
AGCCAATGGATCTTAGTGTGTTTTCCGATGTTCCAATTTCTGTCCTTTGTAAATACGGTGAGTTGGCCCTTACAATAGTTACCAGTGAGTTTCATGAAAAATTTTTATATGCTGAT
AGATACACTTCTATTCGAAATTCCGCGAAACCGCCGACATTAAAAAACCAATTAATTAAGGAAATTGTGGATGGTATAGCTTATACAGAGACATCATATGAGGCAATCAGAAA
GCAAACTGCAAAACTATGGGATATTGCATTAGGTAAGGTGACCAAAGATAAAATCAATAAAGAAGATGCAGCAGCAGCTAGCAAATTTACTTTGAGTTATGAATATCACAGAT
TCAGGCTAATCAATATGGCAGACAATTTAATTGCTAGACTTATGGTGAAACCAAAATCAGATTGGCTAATATCAGTCATGAAGGGGCATCTTAACAGACTATATGAGCACTGGA
AAGTAATGAATGAAATTATCCTAAGTATGGACAACGATTATTCAATTGCAACAACGTTCGAATATTATGCACCATCATGTCTGTGTTTAGCTACGCAGACTTTCCTTATTGTGAG
GAATATGGAAATGGATGATGTCAAGATGATGGTTGCAGTATATAAAAGATTTCTTAACCTAGGAATGTTTCTGCAGAGTGCCAAAGTATGCAGCCTTGCCGATAGTCATACATT
CAGAGATTTTTCTAGATCTTTTTCCTTTATTACGATAATTTCAAGATTGATGATAATCGAATTTATGCAAATTAAAGAATTGACGAAGGTAGAGTTTATTGAGAAGTTTTCTGAAG
TATGCCCTGACCTTGCAGATCTACCTCCGATGCTTCTAGATCCAAACTCTTGCTTATATTTTTCATTGTTACAGCAGATTAAGAAATCTGGTTTTACGTTGTCATTCAAAAAAATTC
TTGAAGACGCTAGAATGATGGACTTCAATTACGACCGCAATTTGGACTCAGAGGCCATTAAAAAGTGCAATGGTGAATTTAGCAAGTCAATGCCTTCCTGTACCAATGTCTCAG
ATACCACCACCGCTGTTTCTGACAACAGTGCTAAGAAGAAAGCTTCAATGGGGTCGGCGAGGGTAAATTCAACTGATACACTAACTGCATCTCCCTTATCAGGCTTAAGGAATC
AAACGCAGTTGGATTCTAAAGACAGTGTTCCATCTCTCGAGGCTTATACACCAATTGATTCTGTCTCTGACGTACCCACTGGGGAGATCAACGTTCCATTCCCTCCTGTTTATAAT
CAAAATGGATTGGATCAGCAAACCACTTATAATTTGGGAACTTTAGATGAGTTTGTTAACAAGGGAGATTTGAATGAACTCTATAATAGCCTATGGGGTGACCTATTTTCTGAT
GTTTACTTGTGA

>18

ATGCAAACATTAGAAACTACATCAAAATCAAATCCAGGGGAAGTCAAAGCACAGAAGCCTAGTACAAGAAGAACAAAAGTTGGAAAAGCTTGTGATAGCTGTAGAAGGAGG
AAAATAAAATGTAATGGGCTAAAACCTTGTCCATCTTGTACAATCTATGGTTGTGAATGTACATATACTGATGCAAAATCGACAAAAAATCTCAAATCAAATGATGCAGGTAAA
CCAAAACCAACAGGGAGAGTATCAAAGAATAAAGAAACTACTAGAATCGACAAAGATATTAGGAAATCAGAGCAGCAGTATGTCCCTATTAATGCTAATATTCATGTTGGTCC
CAGGTTCCCCTCCGAGAATATATTGAATGGATATCCACAATGTGGAGCACCACAGAACAATGTTGTGGGTAATCCACTAGCGGTTAATCCTCAATGCCATAGAGGTCTTTCTGA
AACTCCTATGTCCTCAACATTCAAAGAATCTAACTTAAGAGATGATCGGCTACTACAGTCATCAGATACAGATGATATGAGGAATGGTGACTCGGAAGAAAGGGACTTGAAAG
GGAGTGACAGCGAGAATGTCAAAAGTAAAGACAATAAAAGTGATCCTTTGATTATATACAAAGATGATACACATATTGAAAGCACGGTTAATAAACTAACACAGGCAGTTAAT
GAACTCAAATCACTTCAAAATGCACCTAGTTCGATAAAATCATCCATTGACGCCATTGAGTTACAACTTAGAAACATTTTAGACAATTGGAAACCAGAGGTAGATTTCGAGAAA
GCAAAGATTAATGAAAGTGCCACCACTAAGTCACTTGAAACAAACTTGCTGAGGAATAAATACACTAATCACGTTCATCTAACAAGATTTAGGATATGGATAGATTATAAAAAT
GCGAACAAAAACAATCATTTTATGGGAGAGTGTGGATTTAGTCTTGCAGAATCTTTTTTTGCTTCTAATCAGCCATTGGTCGATGAATTGTTTGGGTTGTATTCCCAGGTAGAGG

CCTTTTCTTTGCAAGGTCTTGGTTACTGTGTTCACCTTTATGAGCCATATATGAAAACTGAGGAAGCGATAAAACTGATGAAAGAGACCTTATATATTATACTACGGTTTATTGA
TATATGTGTTCACCATATCAATGAAGAGTCGATATCGATTGCCAACCCGTTAGAAACATATTTACGAAAAAAACATCTAATGCCTATGACTCCTACACCAAGGTCGTCCTATGGA
AGTCCACAAAGTGCTAGTACAAAGAGCTTGGTAAGTAAGATAATAGAGAGAATACCGCAACCGTTTATTGAGAGTGTAACTAATGTGTCGAGTCTTCAACTATTAGATCTTCGA
GATGACGAGTCAAAAATGTTTGGAACATTGCTGAACATGTGTAAGTCTATAAGGCGAAAATTTGACTCTGTTATGAGCGATTACGATTCCATTGTCACAGAAAAATCCGAAGG
CGAACAAAATGATGGTAAAGTAACTGTAGCTGAGTTCACATCTTTGTGTGAAGCGGAAGAAATGCTCTTAGCATTATGCTATAACTATTATAATCTGACGTTATACAGTTTCTTT
GAATTTGGGACTAATATTGAATACATGGAACATCTGTTGCTTCTTCTTGAAGAACAGCTTGCTCTCGACGAATACTATGGTTTTGAAAAGGTCTTGAATGTAGCTGTTGCAAATG
CTAAAAAAATGGGTTTCCACCGTTGGGAGTTTTACGTCGGTTATGAAGAGTCGACTGCTGAAAAGAGGCGGCTACTATGGTGGAAGTTATACAATTATGAAAAAGCCAGTACT
ATGAAGAAGGGTTTTTTTTCTGTGATTGATGATGCTACTGTCAACTGTTTATTACCTAAGATTTTTAGAAACTTTGGCTATCTGGATAGGGTGGAGTTTCTAGAAAATATTCAAA
AGCCAATGGATCTTAGTGTGTTTTCCGATGTTCCAATTTCTGTCCTTTGTAAATACGGTGAGTTGGCCCTTACAATAGTTACCAGTGAGTTTCATGAAAAATTTTTATATGCTGAT
AGATACACTTCTATTCGAAATTCCGCGAAACCGCCGACATTAAAAAACCAATTAATTAAGGAAATTGTGGATGGTATAGCTTATACAGAGACATCATATGAGGCAATCAGAAA
GCAAACTGCAAAACTATGGGATATTGCATTAGGTAAGGTGACCAAAGATAAAATCAATAAAGAAGATACAGCAGCAGCTAGCAAATTTACTTTGAGTTATGAATATCACAGAT
TCAGGCTAATCAATATGGCAGACAATTTAATTGCTAGACTTATGGTGAAACCAAAATCAGATTGGCTAATATCAGTCATGAAGGGGCATCTTAACAGACTATATGAGCACTGGA
AAGTAATGAATGAAATTATCCTAAGTATGGACAACGATTATTCAATTGCAACAACGTTCGAATATTATGCACCATCATGTCTGTGTTTAGCTACGCAGACTTTCCTTATTGTGAG
GAATATGGAAATGGATGATGTCAAGATGATGGTTGCAGTATATAAAAGATTTCTTAACCTAGGAATGTTTCTGCAGAGTGCCAAAGTATGCAGCCTTGCCGATAGTCATACATT
CAGAGATTTTTCTAGATCTTTTTCCTTTATTACGATAATTTCAAGATTGATGATAATCGAATTTATGCAAATTAAAGAATTGACGAAGGTAGAGTTTATTGAGAAGTTTTCTGAAG
TATGCCCTGACCTTGCAGATCTACCTCCGATGCTTCTAGATCCAAACTCTTGCTTATATTTTTCATTGTTACAGCAGATTAAGAAATCTGGTTTTACGTTGTCATTCAAAAAAATTC
TTGAAGACGCTAGAATGATGGACTTCAATTACGACCGCAATTTGGACTCAGAGGCCATTAAAAAGTGCAATGGTGAATTTAGCAAGTCAATGCCTTCCTGTACCAATGTCTCAG
ATACCACCACCGCTGTTTCTGACAACAGTGCTAAGAAGAAAGCTTCAATGGGGTCGGCGAGGGTAAATTCAACTGATACACTAACTGCATCTCCCTTATCGGGCTTAAGGAATC
AAACGCAGTTGGATTCTAAAGACAGTGTTCCATCTCTCGAGGCTTATACACCAATTGATTCTGTCTCTGACGTACCCACTGGGGAGATCAACGTTCCATTCCCTCCTGTTTATAAT
CAAAATGGATTGGATCAGCAAACCACTTATAATTTGGGAACTTTAGATGAGTTTGTTAACAAGGGAGATTTGAATGAACTCTATAATAGCCTATGGGGTGACCTATTTTCTGAT
GTTTACTTGTGA

>19

ATGCAAACATTAGAAACTACATCAAAATCAAATCCAGGGGAAGTCAAAGCACAGAAGCCTAGTACAAGAAGAACAAAAGTTGGAAAAGCTTGTGATAGCTGTAGAAGGAGG
AAAATAAAATGTAATGGGCTAAAACCTTGTCCATCTTGTACAATCTATGGTTGTGAATGTACATATACTGATGCAAAATCGACAAAAAATCTCAAATCAAATGATGCAGGTAAA
CCAAAACCAACAGGGAGAGTATCAAAGAATAAAGAAACTACTAGAATCGACAAAGATATTAGGAAATCAGAGCAGCAGTATGTCCCTATTAATGCTAATATTCATGTTGGTCC
CAGGTTCCCCTCCGAGAATATATTGAATGGATATCCACAATGTGGAGCACCACAGAACAATGTTGTGGGTAATCCACTAGCGGTTAATCCTCAATGCCATAGAGGTCTTTCTGA
AACTCCTATGTCCTCAACATTCAAAGAATCTAACTTAAGAGATGATCGGCTACTACAGTCATCAGATACAGATGATATGAGGAATGGTGACTCGGAAGAAAGGGACTTGAAAG
GGAGTGACAGCGAGAATGTCAAAAGTAAAGACAATAAAAGTGATCCTTTGATTATATACAAAGATGATACACATATTGAAAGCACGGTTAATAAACTAACACAGGCAGTTAAT
GAACTCAAATCACTTCAAAATGCACCTAGTTCGATAAAATCATCCATTGACGCCATTGAGTTACAACTTAGAAACATTTTAGACAATTGGAAACCAGAGGTAGATTTCGAGAAA
GCAAAGATTAATGAAAGTGCCACCACTAAGTCACTTGAAACAAACTTGCTGAGGAATAAATACACTAATCACGTTCATCTAACAAGATTTAGGATATGGATAGATTATAAAAAT

GCGAACAAAAACAATCATTTTATGGGAGAGTGTGGATTTAGTCTTGCAGAATCTTTTTTTGCTTCTAATCAGCCATTGGTCGATGAATTGTTTGGGTTGTATTCCCAGGTAGAGG
CCTTTTCTTTGCAAGGTCTTGGTTACTGTGTTCACCTTTATGAGCCATATATGAAAACTGAGGAAGCGATAAAACTGATGAAAGAGACCTTATATATTATACTACGGTTTATTGA
TATATGTGTTCACCATATCAATGAAGAGTCGATATCGATTGCCAACCCGTTAGAAACATATTTACGAAAAAAACATCTAATGCCTATGACTCCTACACCAAGGTCGTCCTATGGA
AGTCCACAAAGTGCTAGTACAAAGAGCTTGGTAAGTAAGATAATAGAGAGAATACCGCAACCGTTTATTGAGAGTGTAACTAATGTGTCGAGTCTTCAACTATTAGATCTTCGA
GATGACGAGTCAAAAATGTTTGGAACATTGCTGAACATGTGTAAGTCTATAAGGCGAAAATTTGACTCTGTTATGAGCGATTACGATTCCATTGTCACAGAAAAATCCGAAGG
CGAACAAAATGATGGTAAAGTAACTGTAGCTGAGTTCACATCTTTGTGTGAAGCGGAAGAAATGCTCTTAGCATTATGCTATAACTATTATAATCTGACGTTATACAGTTTCTTT
GAATTTGGGACTAATATTGAATACATGGAACATCTGTTGCTTCTTCTTGAAGAACAGCTTGCTCTCGACGAATACTATGGTTTTGAAAAGGTCTTGAATGTAGCTGTTGCAAATG
CTAAAAAAATGGGTTTCCACCGTTGGGAGTTTTACGTCGGTTATGAAGAGTCGACTGCTGAAAAGAGGCGGCTACTATGGTGGAAGTTATACAATTATGAAAAAGCCAGTACT
ATGAAGAAGGGTTTTTTTTCTGTGATTGATGATGCTACTGTCAACTGTTTATTACCTAAGATTTTTAGAAACTTTGGCTATCTGGATAGGGTGGAGTTTCTAGAAAATATTCAAA
AGCCAATGGATCTTAGTGTGTTTTCCGATGTTCCAATTTCTGTCCTTTGTAAATACGGTGAGTTGGCCCTTACAATAGTTACCAGTGAGTTTCATGAAAAATTTTTATATGCTGAT
AGATACACTTCTATTCGAAATTCCGCGAAACCGCCGACATTAAAAAACCAATTAATTAAGGAAATTGTGGATGGTATAGCTTATACAGAGACATCATATGAGGCAATCAGAAA
GCAAACTGCAAAACTATGGGATATTGCATTAGGTAAGGTGACCAAAGATAAAATCAATAAAGAAGATACAGCAGCAGCTAGCAAATTTACTTTGAGTTATGAATATCACAGAT
TCAGGCTAATCAATATGGCAGACAATTTAATTGCTAGACTTATGGTGAAACCAAAATCAGATTGGCTAATATCAGTCATGAAGGGGCATCTTAACAGACTATATGAGCACTGGA
AAGTAATGAATGAAATTATCCTAAGTATGGACAACGATTATTCAATTGCAACAACGTTCGAATATTATGCACCATCATGTCTGTGTTTAGCTACGCAGACTTTCCTTATTGTGAG
GAATATGGAAATGGATGATGTCAAGATGATGGTTGCAGTATATAAAAGATTTCTTAACCTAGGAATGTTTCTGCAGAGTGCCAAAGTATGCAGCCTTGCCGATAGTCATACATT
CAGAGATTTTTCTAGATCTTTTTCCTTTATTACGATAATTTCAAGATTGATGATAATCGAATTTATGCAAATTAAAGAATTGACGAAGGTAGAGTTTATTGAGAAGTTTTCTGAAG
TATGCCCTGACCTTGCAGATCTACCTCCGATGCTTCTAGATCCAAACTCTTGCTTATATTTTTCATTGTTACAGCAGATTAAGAAATCTGGTTTTACGTTGTCATTCAAAAAAAATTC
TTGAAGACGCTAGAATGATGGACTTCAATTACGACCGCAATTTGGACTCAGAGGCCATTAAAAAGTGCAATGGTGAATTTAGCAAGTCAATGCCTTCCTGTACCAATGTCTCAG
ATACCACCACCGCTGTTTCTGACAACAGTGCTAAGAAGAAAGCTTCAATGGGGTCGGCGAGGGTAAATTCAACTGATACACTAACTGCATCTCCCTTATCGGGCTTAAGGAATC
AAACGCAGTTGGATTCTAAAGACAGTGTTCCATCTCTCGAGGCTTATACACCAATTGATTCTGTCTCTGACGTACCCACTGGGGAGATCAACGTTCCATTCCCTCCTGTTTATAAT
CAAAATGGATTGGATCAGCAAACCACTTATAATTTGGGAACTTTAGATGAGTTTGTTAACAAGGGAGATTTGAATGAACTCTATAATAGCCTATGGGGTGACCTATTTTCTGAT
GTTTACTTGTGA

>20

ATGCAAACATTAGAAACTACATCAAAATCAAATCCAGGGGAAGTCAAAGCACAGAAGCCTAGTACAAGAAGAACAAAAGTTGGAAAAGCTTGTGATAGCTGTAGAAGGAGG
AAAATAAAATGTAATGGGCTAAAACCTTGTCCATCTTGTACAATCTATGGTTGTGAATGTACATATACTGATGCAAAATCGACAAAAAATCTCAAATCAAATGATGCAGGTAAA
CCAAAACCAACAGGGAGAGTATCAAAGAATAAAGAAACTACTAGAATCGACAAAGATATTAGGAAATCAGAGCAGCAGTATGTCCCTATTAATGCTAATATTCATGTTGGTCC
CAGGTTCCCCTCCGAGAATATATTGAATGGATATCCACAATGTGGAGCACCACAGAACAATGTTGTGGGTAATCCACTAGCGGTTAATCCTCAATGCCATAGAGGTCTTTCTGA
AACTCCTATGTCCTCAACATTCAAAGAATCTAACTTAAGAGATGATCGGCTACTACAGTCATCAGATACAGATGATATGAGGAATGGTGACTCGGAAGAAAGGGACTTGAAAG
GGAGTGACAGCGAGAATGTCAAAAGTAAAGACAATAAAAGTGATCCTTTGATTATATACAAAGATGATACACATATTGAAAGCACGGTTAATAAACTAACACAGGCAGTTAAT
GAACTCAAATCACTTCAAAATGCACCTAGTTCGATAAAATCATCCATTGACGCCATTGAGTTACAACTTAGAAACATTTTAGACAATTGGAAACCAGAGGTAGATTTCGAGAAA

GCAAAGATTAATGAAAGTGCCACCACTAAGTCACTTGAAACAAACTTGCTGAGGAATAAATACACTAATCACGTTCATCTAACAAGATTTAGGATATGGATAGATTATAAAAAT
GCGAACAAAAACAATCATTTTATGGGAGAGTGTGGATTTAGTCTTGCAGAATCTTTTTTTGCTTCTAATCAGCCATTGGTCGATGAATTGTTTGGGTTGTATTCCCAGGTAGAGG
CCTTTTCTTTGCAAGGTCTTGGTTACTGTGTTCACCTTTATGAGCCATATATGAAAACTGAGGAAGCGATAAAACTGATGAAAGAGACCTTATATATTATACTACGGTTTATTGA
TATATGTGTTCACCATATCAATGAAGAGTCGATATCGATTGCCAACCCGTTAGAAACATATTTACGAAAAAAACATCTAATGCCTATGACTCCTACACCAAGGTCGTCCTATGGA
AGTCCACAAAGTGCTAGTACAAAGAGCTTGGTAAGTAAGATAATAGAGAGAATACCGCAACCGTTTATTGAGAGTGTAACTAATGTGTCGAGTCTTCAACTATTAGATCTTCGA
GATGACGAGTCAAAAATGTTTGGAACATTGCTGAACATGTGTAAGTCTATAAGGCGAAAATTTGACTCTGTTATGAGCGATTACGATTCCATTGTCACAGAAAAATCCGAAGG
CGAACAAAATGATGGTAAAGTAACTGTAGCTGAGTTCACATCTTTGTGTGAAGCGGAAGAAATGCTCTTAGCATTATGCTATAACTATTATAATCTGACGTTATACAGTTTCTTT
GAATTTGGGACTAATATTGAATACATGGAACATCTGTTGCTTCTTCTTGAAGAACAGCTTGCTCTCGACGAATACTATGGTTTTGAAAAGGTCTTGAATGTAGCTGTTGCAAATG
CTAAAAAAATGGGTTTCCACCGTTGGGAGTTTTACGTCGGTTATGAAGAGTCGACTGCTGAAAAGAGGCGGCTACTATGGTGGAAGTTATACAATTATGAAAAAGCCAGTACT
ATGAAGAAGGGTTTTTTTTCTGTGATTGATGATGCTACTGTCAACTGTTTATTACCTAAGATTTTTAGAAACTTTGGCTATCTGGATAGGGTGGAGTTTCTAGAAAATATTCAAA
AGCCAATGGATCTTAGTGTGTTTTCCGATGTTCCAATTTCTGTCCTTTGTAAATACGGTGAGTTGGCCCTTACAATAGTTACCAGTGAGTTTCATGAAAAATTTTTATATGCTGAT
AGATACACTTCTATTCGAAATTCCGCGAAACCGCCGACATTAAAAAACCAATTAATTAAGGAAATTGTGGATGGTATAGCTTATACAGAGACATCATATGAGGCAATCAGAAA
GCAAACTGCAAAACTATGGGATATTGCATTAGGTAAGGTGACCAAAGATAAAATCAATAAAGAAGATACAGCAGCAGCTAGCAAATTTACTTTGAGTTATGAATATCACAGAT
TCAGGCTAATCAATATGGCAGACAATTTAATTGCTAGACTTATGGTGAAACCAAAATCAGATTGGCTAATATCAGTCATGAAGGGGCATCTTAACAGACTATATGAGCACTGGA
AAGTAATGAATGAAATTATCCTAAGTATGGACAACGATTATTCAATTGCAACAACGTTCGAATATTATGCACCATCATGTCTGTGTTTAGCTACGCAGACTTTCCTTATTGTGAG
GAATATGGAAATGGATGATGTCAAGATGATGGTTGCAGTATATAAAAGATTTCTTAACCTAGGAATGTTTCTGCAGAGTGCCAAAGTATGCAGCCTTGCCGATAGTCATACATT
CAGAGATTTTTCTAGATCTTTTTCCTTTATTACGATAATTTCAAGATTGATGATAATCGAATTTATGCAAATTAAAGAATTGACGAAGGTAGAGTTTATTGAGAAGTTTTCTGAAG
TATGCCCTGACCTTGCAGATCTACCTCCGATGCTTCTAGATCCAAACTCTTGCTTATATTTTTCATTGTTACAGCAGATTAAGAAATCTGGTTTTACGTTGTCATTCAAAAAAAATTC
TTGAAGACGCTAGAATGATGGACTTCAATTACGACCGCAATTTGGACTCAGAGGCCATTAAAAAGTGCAATGGTGAATTTAGCAAGTCAATGCCTTCCTGTACCAATGTCTCAG
ATACCACCACCGCTGTTTCTGACAACAGTGCTAAGAAGAAAGCTTCAATGGGGTCGGCGAGGGTAAATTCAACTGATACACTAACTGCATCTCCCTTATCGGGCTTAAGGAATC
AAACGCAGTTGGATTCTAAAGACAGTGTTCCATCTCTCGAGGCTTATACACCAATTGATTCTGTCTCTGACGTACCCACTGGGGAGATCAACGTTCCATTCCCTCCTGTTTATAAT
CAAAATGGATTGGATCAGCAAACCACTTATAATTTGGGAACTTTAGATGAGTTTGTTAACAAGGGAGATTTGAATGAACTCTATAATAGCCTATGGGGTGACCTATTTTCTGAT
GTTTACTTGTGA

>21

ATGCAAACATTAGAAACTACATCAAAATCAAATCCAGGGGAAGTCAAAGCACAGAAGCCTAGTACAAGAAGAACAAAAGTTGGAAAAGCTTGTGATAGCTGTAGAAGGAGG
AAAATAAAATGTAATGGGCTAAAACCTTGTCCATCTTGTACAATCTATGGTTGTGAATGTACATATACTGATGCAAAATCGACAAAAAATCTCAAATCAAATGATGCAGGTAAA
CCAAAACCAACAGGGAGAGTATCAAAGAATAAAGAAACTACTAGAATCGACAAAGATATTAGGAAATCAGAGCAGCAGTATGTCCCTATTAATGCTAATATTCATGTTGGTCC
CAGGTTCCCCTCCGAGAATATATTGAATGGATATCCACAATGTGGAGCACCACAGAACAATGTTGTGGGTAATCCACTAGCGGTTAATCCTCAATGCCATAGAGGTCTTTCTGA
AACTCCTATGTCCTCAACATTCAAAGAATCTAACTTGAGAGATGATCGGCTACTACAGTCATCAGATACAGATGATATGAGGAATGGTGACTCGGAAGAAAGGGACTTGAAAG
GGAGTGACAGCGAGAATGTCAAAAGTAAAGACAATAAAAGTGATCCTTTGATTATATACAAAGATGATACACATATTGAAAGCACGGTTAATAAACTAACACAGGCAGTTAAT

GAACTCAAATCACTTCAAAATGCACCTAGTTCGATAAAATCATCCATTGACGCCATTGAGTTACAACTTAGAAACATTTTAGACAATTGGAAACCAGAGGTAGATTTCGAGAAA
GCAAAGATTAATGAAAGTGCCACCACTAAGTCACTTGAAACAAACTTGCTGAGGAATAAATACACTAATCACGTTCATCTAACAAGATTTAGGATATGGATAGATTATAAAAAT
GCGAACAAAAACAATCATTTTATGGGAGAGTGTGGATTTAGTCTTGCAGAATCTTTTTTTGCTTCTAATCAGCCATTGGTCGATGAATTGTTTGGGTTGTATTCCCAGGTAGAGG
CCTTTTCTTTGCAAGGTCTTGGTTACTGTGTTCACCTTTATGAGCCATATATGAAAACTGAGGAAGCGATAAAACTGATGAAAGAGACCTTATATATTATACTACGGTTTATTGA
TATATGTGTTCACCATATCAATGAAGAGTCGATATCGATTGCCAACCCGTTAGAAACATATTTACGAAAAAAACATCTAATGCCTATGACTCCTACACCAAGGTCGTCCTATGGA
AGTCCACAAAGTGCTAGTACAAAGAGCTTGGTAAGTAAGATAATAGAGAGAATACCGCAACCGTTTATTGAGAGTGTAACTAATGTGTCGAGTCTTCAACTATTAGATCTTCGA
GATGACGAGTCAAAAATGTTTGGAACATTGCTGAACATGTGTAAGTCTATAAGGCGAAAATTTGACTCTGTTATGAGCGATTACGATTCCATTGTCACAGAAAAATCCGAAGG
CGAACAAAATGATGGTAAAGTAACTGTAGCTGAGTTCACATCTTTGTGTGAAGCGGAAGAAATGCTCTTAGCATTATGCTATAACTATTATAATCTGACGTTATACAGTTTCTTT
GAATTTGGGACTAATATTGAATACATGGAACATCTGTTGCTTCTTCTTGAAGAACAGCTTGCTCTCGACGAATACTATGGTTTTGAAAAGGTCTTGAATGTAGCTGTTGCAAATG
CTAAAAAAATGGGTTTCCACCGTTGGGAGTTTTACGTCGGCTATGAAGAGTCGACTGCTGAAAAGAGGCGGCTACTATGGTGGAAGTTATACAATTATGAAAAAGCCAGTACT
ATGAAGAAGGGTTTTTTTTCTGTGATTGATGATGCTACTGTCAACTGTTTATTACCTAAGATTTTTAGAAACTTTGGCTATCTGGATAGGGTGGAGTTTCTAGAAAATATTCAAA
AGCCAATGGATCTTAGTGTGTTTTCCGATGTTCCAATTTCTGTCCTTTGTAAATACGGTGAGTTGGCCCTTACAATAGTTACCAGTGAGTTTCATGAAAAATTTTTATATGCTGAT
AGATACACTTCTATTCGAAATTCCGCGAAACCGCCGACATTAAAAAACCAATTAATTAAGGAAATTGTGGATGGTATAGCTTATACAGAGACATCATATGAGGCAATCAGAAA
GCAAACTGCAAAACTATGGGATATTGCATTAGGTAAGGTGACCAAAGATAAAATCAATAAAGAAGATACAGCAGCAGCTAGCAAATTTACTTTGAGTTATGAATATCACAGAT
TCAGGCTAATCAATATGGCAGACAATTTAATTGCTAGACTTATGGTGAAACCAAAATCAGATTGGCTAATATCAGTCATGAAGGGGCATCTTAACAGACTATATGAGCACTGGA
AAGTAATGAATGAAATTATCCTAAGTATGGACAACGATTATTCAATTGCAACAACGTTCGAATATTATGCACCATCATGTCTGTGTTTAGCTACGCAGACTTTCCTTATTGTGAG
GAATATGGAAATGGATGATGTCAAGATGATGGTTGCAGTATATAAAAGATTTCTTAACCTAGGAATGTTTCTGCAGAGTGCCAAAGTATGCAGCCTTGCCGATAGTCATACATT
CAGAGATTTTTCTAGATCTTTTTCCTTTATTACGATAATTTCAAGATTGATGATAATCGAATTTATGCAAATTAAAGAATTGACGAAGGTAGAGTTTATTGAGAAGTTTTCTGAAG
TATGCCCTGACCTTGCAGATCTACCTCCGATGCTTCTAGATCCAAACTCTTGCTTATATTTTTCATTGTTACAGCAGATTAAGAAATCTGGTTTTACGTTGTCATTCAAAAAAATTC
TTGAAGACGCTAGAATGATGGACTTCAATTACGACCGCAATTTGGACTCAGAGGCCATTAAAAAGTGCAATGGTGAATTTAGCAAGTCAATGCCTTCCTGTACCAATGTCTCAG
ATACCACCACCGCTGTTTCTGACAACAGTGCTAAGAAGAAATCTTCAATGGGGTCGGCGAGGGTAAATTCAACTGATACACTAACTGCATCTCCCTTATCGGGCTTAAGGAATC
AAACGCAGTTGGATTCTAAAGACAGTGTTCCATCTCTCGAGGCTTATACACCAATTGATTCTGTCTCTGACGTACCCACTGGGGAGATCAACGTTCCATTCCCTCCTGTTTATAAT
CAAAATGGATTGGATCAGCAAACCACTTATAATTTGGGAACTTTAGATGAGTTTGTTAACAAGGGAGATTTGAATGAACTCTATAATAGCCTATGGGGTGACCTATTTTCTGAT
GTTTACTTGTGA

>22

ATGCAAACATTAGAAACTACATCAAAATCAAATCCAGGGGAAGTCAAAGCACAGAAGCCTAGTACAAGAAGAACAAAAGTTGGAAAAGCTTGTGATAGCTGTAGAAGGAGG
AAAATAAAATGTAATGGGCTAAAACCTTGTCCATCTTGTACAATCTATGGTTGTGAATGTACATATACTGATGCAAAATCGACAAAAAATCTCAAATCAAATGATGCAGGTAAA
CCAAAACCAACAGGGAGAGTATCAAAGAATAAAGAAACTACTAGAATCGACAAAGATATTAGGAAATCAGAGCAGCAGTATGTCCCTATTAATGCTAATATTCATGTTGGTCC
CAGGTTCCCCTCCGAGAATATATTGAATGGATATCCACAATGTGGAGCACCACAGAACAATGTTGTGGGTAATCCACTAGCGGTTAATCCTCAATGCCATAGAGGTCTTTCTGA
AACTCCTATGTCCTCAACATTCAAAGAATCTAACTTGAGAGATGATCGGCTACTACAGTCATCAGATACAGATGATATGAGGAATGGTGACTCGGAAGAAAGGGACTTGAAAG

GGAGTGACAGCGAGAATGTCAAAAGTAAAGACAATAAAAGTGATCCTTTGATTATATACAAAGATGATACACATATTGAAAGCACGGTTAATAAACTAACACAGGCAGTTAAT
GAACTCAAATCACTTCAAAATGCACCTAGTTCGATAAAATCATCCATTGACGCCATTGAGTTACAACTTAGAAACATTTTAGACAATTGGAAACCAGAGGTAGATTTCGAGAAA
GCAAAGATTAATGAAAGTGCCACCACTAAGTCACTTGAAACAAACTTGCTGAGGAATAAATACACTAATCACGTTCATCTAACAAGATTTAGGATATGGATAGATTATAAAAAT
GCGAACAAAAACAATCATTTTATGGGAGAGTGTGGATTTAGTCTTGCAGAATCTTTTTTTGCTTCTAATCAGCCATTGGTCGATGAATTGTTTGGGTTGTATTCCCAGGTAGAGG
CCTTTTCTTTGCAAGGTCTTGGTTACTGTGTTCACCTTTATGAGCCATATATGAAAACTGAGGAAGCGATAAAACTGATGAAAGAGACCTTATATATTATACTACGGTTTATTGA
TATATGTGTTCACCATATCAATGAAGAGTCGATATCGATTGCCAACCCGTTAGAAACATATTTACGAAAAAAACATCTAATGCCTATGACTCCTACACCAAGGTCGTCCTATGGA
AGTCCACAAAGTGCTAGTACAAAGAGCTTGGTAAGTAAGATAATAGAGAGAATACCGCAACCGTTTATTGAGAGTGTAACTAATGTGTCGAGTCTTCAACTATTAGATCTTCGA
GATGACGAGTCAAAAATGTTTGGAACATTGCTGAACATGTGTAAGTCTATAAGGCGAAAATTTGACTCTGTTATGAGCGATTACGATTCCATTGTCACAGAAAAATCCGAAGG
CGAACAAAATGATGGTAAAGTAACTGTAGCTGAGTTCACATCTTTGTGTGAAGCGGAAGAAATGCTCTTAGCATTATGCTATAACTATTATAATCTGACGTTATACAGTTTCTTT
GAATTTGGGACTAATATTGAATACATGGAACATCTGTTGCTTCTTCTTGAAGAACAGCTTGCTCTCGACGAATACTATGGTTTTGAAAAGGTCTTGAATGTAGCTGTTGCAAATG
CTAAAAAAATGGGTTTCCACCGTTGGGAGTTTTACGTCGGTTATGAAGAGTCGACTGCTGAAAAGAGGCGGCTACTATGGTGGAAGTTATACAATTATGAAAAAGCCAGTACT
ATGAAGAAGGGTTTTTTTTCTGTGATTGATGATGCTACTGTCAACTGTTTATTACCTAAGATTTTTAGAAACTTTGGCTATCTGGATAGGGTGGAGTTTCTAGAAAATATTCAAA
AGCCAATGGATCTTAGTGTGTTTTCCGATGTTCCAATTTCTGTCCTTTGTAAATACGGTGAGTTGGCCCTTACAATAGTTACCAGTGAGTTTCATGAAAAATTTTTATATGCTGAT
AGATACACTTCTATTCGAAATTCCGCGAAACCGCCGACATTAAAAAACCAATTAATTAAGGAAATTGTGGATGGTATAGCTTATACAGAGACATCATATGAGGCAATCAGAAA
GCAAACTGCAAAACTATGGGATATTGCATTAGGTAAGGTGACCAAAGATAAAATCAATAAAGAAGATACAGCAGCAGCTAGCAAATTTACTTTGAGTTATGAATATCACAGAT
TCAGGCTAATCAATATGGCAGACAATTTAATTGCTAGACTTATGGTGAAACCAAAATCAGATTGGCTAATATCAGTCATGAAGGGGCATCTTAACAGACTATATGAGCACTGGA
AAGTAATGAATGAAATTATCCTAAGTATGGACAACGATTATTCAATTGCAACAACGTTCGAATATTATGCACCATCATGTCTGTGTTTAGCTACGCAGACTTTCCTTATTGTGAG
GAATATGGAAATGGATGATGTCAAGATGATGGTTGCAGTATATAAAAGATTTCTTAACCTAGGAATGTTTCTGCAGAGTGCCAAAGTATGCAGCCTTGCCGATAGTCATACATT
CAGAGATTTTTCTAGATCTTTTTCCTTTATTACGATAATTTCAAGATTGATGATAATCGAATTTATGCAAATTAAAGAATTGACGAAGGTAGAGTTTATTGAGAAGTTTTCTGAAG
TATGCCCTGACCTTGCAGATCTACCTCCGATGCTTCTAGATCCAAACTCTTGCTTATATTTTTCATTGTTACAGCAGATTAAGAAATCTGGTTTTACGTTGTCATTCAAAAAAATTC
TTGAAGACGCTAGAATGATGGACTTCAATTACGACCGCAATTTGGACTCAGAGGCCATTAAAAAGTGCAATGGTGAATTTAGCAAGTCAATGCCTTCCTGTACCAATGTCTCAG
ATACCACCACCGCTGTTTCTGACAACAGTGCTAAGAAGAAAGCTTCAATGGGGTCGGCGAGGGTAAATTCAACTGATACACTAACTGCATCTCCCTTATCGGGCTTAAGGAATC
AAACGCAGTTGGATTCTAAAGACAGTGTTCCATCTCTCGAGGCTTATACACCAATTGATTCTGTCTCTGACGTACCCACTGGGGAGATCAACGTTCCATTCCCTCCTGTTTATAAT
CAAAATGGATTGGATCAGCAAACCACTTATAATTTGGGAACTTTAGATGAGTTTGTTAACAAGGGAGATTTGAATGAACTCTATAATAGCCTATGGGGTGACCTATTTTCTGAT
GTTTACTTGTGA

>23

ATGCAAACATTAGAAACTACATCAAAATCAAATCCAGGGGAAGTCAAAGCACAGAAGCCTAGTACAAGAAGAACAAAAGTTGGAAAAGCTTGTGATAGCTGTAGAAGGAGG
AAAATAAAATGTAATGGGCTAAAACCTTGTCCATCTTGTACAATCTATGGTTGTGAATGTACATATACTGATGCAAAATCGACAAAAAATCTCAAATCAAATGATGCAGGTAAA
CCAAAACCAACAGGGAGAGTATCAAAGAATAAAGAAACTACTAGAATCGACAAAGATATTAGGAAATCAGAGCAGCAGTATGTCCCTATTAATGCTAATATTCATGTTGGTCC
CAGGTTCCCCTCCGAGAATATATTGAATGGATATCCACAATGTGGAGCACCACAGAACAATGTTGTGGGTAATCCACTAGCGGTTAATCCTCAATGCCATAGAGGTCTTTCTGA

AACTCCTATGTCCTCAACATTCAAAGAATCTAACTTGAGAGATGATCGGCTACTACAGTCATCAGATACAGATGATATGAGGAATGGTGACTCGGAAGAAAGGGACTTGAAAG
GGAGTGACAGCGAGAATGTCAAAAGTAAAGACAATAAAAGTGATCCTTTGATTATATACAAAGATGATACACATATTGAAAGCACGGTTAATAAACTAACACAGGCAGTTAAT
GAACTCAAATCACTTCAAAATGCACCTAGTTCGATAAAATCATCCATTGACGCCATTGAGTTACAACTTAGAAACATTTTAGACAATTGGAAACCAGAGGTAGATTTCGAGAAA
GCAAAGATTAATGAAAGTGCCACCACTAAGTCACTTGAAACAAACTTGCTGAGGAATAAATACACTAATCACGTTCATCTAACAAGATTTAGGATATGGATAGATTATAAAAAT
GCGAACAAAAACAATCATTTTATGGGAGAGTGTGGATTTAGTCTTGCAGAATCTTTTTTTGCTTCTAATCAGCCATTGGTCGATGAATTGTTTGGGTTGTATTCCCAGGTAGAGG
CCTTTTCTTTGCAAGGTCTTGGTTACTGTGTTCACCTTTATGAGCCATATATGAAAACTGAGGAAGCGATAAAACTGATGAAAGAGACCTTATATATTATACTACGGTTTATTGA
TATATGTGTTCACCATATCAATGAAGAGTCGATATCGATTGCCAACCCGTTAGAAACATATTTACGAAAAAAACATCTAATGCCTATGACTCCTACACCAAGGTCGTCCTATGGA
AGTCCACAAAGTGCTAGTACAAAGAGCTTGGTAAGTAAGATAATAGAGAGAATACCGCAACCGTTTATTGAGAGTGTAACTAATGTGTCGAGTCTTCAACTATTAGATCTTCGA
GATGACGAGTCAAAAATGTTTGGAACATTGCTGAACATGTGTAAGTCTATAAGGCGAAAATTTGACTCTGTTATGAGCGATTACGATTCCATTGTCACAGAAAAATCCGAAGG
CGAACAAAATGATGGTAAAGTAACTGTAGCTGAGTTCACATCTTTGTGTGAAGCGGAAGAAATGCTCTTAGCATTATGCTATAACTATTATAATCTGACGTTATACAGTTTCTTT
GAATTTGGGACTAATATTGAATACATGGAACATCTGTTGCTTCTTCTTGAAGAACAGCTTGCTCTCGACGAATACTATGGTTTTGAAAAGGTCTTGAATGTAGCTGTTGCAAATG
CTAAAAAAATGGGTTTCCACCGTTGGGAGTTTTACGTCGGTTATGAAGAGTCGACTGCTGAAAAGAGGCGGCTACTATGGTGGAAGTTATACAATTATGAAAAAGCCAGTACT
ATGAAGAAGGGTTTTTTTTCTGTGATTGATGATGCTACTGTCAACTGTTTATTACCTAAGATTTTTAGAAACTTTGGCTATCTGGATAGGGTGGAGTTTCTAGAAAATATTCAAA
AGCCAATGGATCTTAGTGTGTTTTCCGATGTTCCAATTTCTGTCCTTTGTAAATACGGTGAGTTGGCCCTTACAATAGTTACCAGTGAGTTTCATGAAAAATTTTTATATGCTGAT
AGATACACTTCTATTCGAAATTCCGCGAAACCGCCGACATTAAAAAACCAATTAATTAAGGAAATTGTGGATGGTATAGCTTATACAGAGACATCATATGAGGCAATCAGAAA
GCAAACTGCAAAACTATGGGATATTGCATTAGGTAAGGTGACCAAAGATAAAATCAATAAAGAAGATACAGCAGCAGCTAGCAAATTTACTTTGAGTTATGAATATCACAGAT
TCAGGCTAATCAATATGGCAGACAATTTAATTGCTAGACTTATGGTGAAACCAAAATCAGATTGGCTAATATCAGTCATGAAGGGGCATCTTAACAGACTATATGAGCACTGGA
AAGTAATGAATGAAATTATCCTAAGTATGGACAACGATTATTCAATTGCAACAACGTTCGAATATTATGCACCATCATGTCTGTGTTTAGCTACGCAGACTTTCCTTATTGTGAG
GAATATGGAAATGGATGATGTCAAGATGATGGTTGCAGTATATAAAAGATTTCTTAACCTAGGAATGTTTCTGCAGAGTGCCAAAGTATGCAGCCTTGCCGATAGTCATACATT
CAGAGATTTTTCTAGATCTTTTTCCTTTATTACGATAATTTCAAGATTGATGATAATCGAATTTATGCAAATTAAAGAATTGACGAAGGTAGAGTTTATTGAGAAGTTTTCTGAAG
TATGCCCTGACCTTGCAGATCTACCTCCGATGCTTCTAGATCCAAACTCTTGCTTATATTTTTCATTGTTACAGCAGATTAAGAAATCTGGTTTTACGTTGTCATTCAAAAAAAATTC
TTGAAGACGCTAGAATGATGGACTTCAATTACGACCGCAATTTGGACTCAGAGGCCATTAAAAAGTGCAATGGTGAATTTAGCAAGTCAATGCCTTCCTGTACCAATGTCTCAG
ATACCACCACCGCTGTTTCTGACAACAGTGCTAAGAAGAAAGCTTCAATGGGGTCGGCGAGGGTAAATTCAACTGATACACTAACTGCATCTCCCTTATCGGGCTTAAGGAATC
AAACGCAGTTGGATTCTAAAGACAGTGTTCCATCTCTCGAGGCTTATACACCAATTGATTCTGTCTCTGACGTACCCACTGGGGAGATCAACGTTCCATTCCCTCCTGTTTATAAT
CAAAATGGATTGGATCAGCAAACCACTTATAATTTGGGAACTTTAGATGAGTTTGTTAACAAGGGAGATTTGAATGAACTCTATAATAGCCTATGGGGTGACCTATTTTCTGAT
GTTTACTTGTGA

>24

ATGCAAACATTAGAAACTACATCAAAATCAAATCCAGGGGAAGTCAAAGCACAGAAGCCTAGTACAAGAAGAACAAAAGTTGGAAAAGCTTGTGATAGCTGTAGAAGGAGG
AAAATAAAATGTAATGGGCTAAAACCTTGTCCATCTTGTACAATCTATGGCTGTGAATGTACATATACTGATGCAAAATCGACAAAAAATCTCAAATCAAATGATGCAGGTAAA
TCAAAACCAACAGGGAGAGTATCAAAGAATAAAGAAACTACTAGAATCGACAAAGATATTAGGAAATCAGAGCAGCAGTATGTCCCTATTAATGCTAATATTCATGTTGGTCC

CAGGTTCCCCTCCGAGAATATATTGAATGGATATCCACAATGTGGAGCACCACAGAACAATGTTGTGGGTAATCCACTAGCGGTTAATACTCAATGCCATAGAGGTCTTTCTGA
AACTCCTATGTCCTCAACATTCAAAGAATCTAACTTAAGAGATGATCGGCTACTACAGTCATCAGATACAGATGATATGAGGAATGGTGACTCGGAAGAAAGGGACTTGAAAG
GGAGTGACAGCGAGAATGTCAAAAGTAAAGACAATAAAAGTGATCCTTTGATTATATACAAAGATGATACACATATTGAAAGCACGGTTAATAAACTAACACAGGCAGTTAAT
GAACTCAAATCACTTCAAAATGCACCCAGTTCGATAAAATCATCCATTAACGCCATTGAGTTACAACTTAGAAACATTTTAGACAACTGGAAACCAGAGGTAGATTTCGAGAAA
GCAAAGATTAATGAAAGTGCCACCACTAAGTCACTTGAAACAAACTTGCTGAGGAATAAATACACTAATCACGTTCATTTAACAAGATTTAGGATATGGATAGATTATAAAAAT
GCGAACAAAAACAATCATTTTATGGGAGAGTGTGGATTTAGTCTTGCAGAATCTTTTTTTGCTTCTAATCAGCCATTGGTCGATGAATTGTTTGGGTTGTATTCCCAGGTAGAGG
CCTTTTCTTTGCAAGGTCTTGGTTACTGTGTTCACCTTTATGAGCCATATATGAAAACTGAGGAAGCGATAAAACTGATGAAAGAGACCTTATATATTATACTACGGTTTATTGA
TATATGTGTTCACCATATCAATGAAGAGTCGATATCGATTGCCAACCCGTTAGAAACATATTTACGAAAAAAACATCTAATGCCTATGACTCCTACACCAAGGTCGTCCTATGGA
AGTCCACAAAGTGCTAGTACAAAGAGCTTGGTAAGTAAGATAATAGAGAGAATACCGCAACCGTTTATTGAGAGTGTAACTAATGTGTCGAGTCTTCAACTATTAGATCTTCGA
GATGACGAGTCAAAAATGTTTGGAACATTGCTGAACATGTGTAAGTCTATAAGGCGAAAATTTGACTCTGTTATGAGCGATTACGATTCCATTGTCACAGAAAAATCCGAAGG
CGAACAAAATGATGGTAAAGTAACTGTAGCTGAGTTCACATCTTTGTGTGAAGCGGAAGAAATGCTCTTAGCATTATGCTATAACTATTATAATCTGACGTTATACAGTTTCTTT
GAATTTGGGACTAATATTGAATACATGGAACATCTGTTGCTTCTTCTTGAAGAACAGCTTGCTCTCGACGAATACTATGGTTTTGAAAAGGTCTTGAATGTAGCTGTTGCAAATG
CTAAAAAAATGGGTTTCCACCGTTGGGAGTTTTACGTCGGCTATGAAGAGTCGACTGCTGAAAAGAGGCGGCTACTATGGTGGAAGTTATACAATTATGAAAAAGCCAGTACT
ATGAAGAAGGGTTTTTTTTCTGTGATTGATGATGCTACTGTCAACTGTTTATTACCTAAGATTTTTAGAAACTTTGGCTATCTGGATAGGGTGGAGTTTCTAGAAAATATTCAAA
AGCCAATGGATCTTAGTGTGTTTTCCGATGTTCCAATTTCTGTCCTTTGTAAATACGGTGAGTTGGCCCTTACAATAGTTACCAGTGAGTTTCATGAAAAATTTTTATATGCTGAT
AGATACACTTCTATTCGAAATTCCGCGAAACCGCCGACATTAAAAAACCAATTAATTAAGGAAATTGTGGATGGTATAGCTTATACAGAGACATCATATGAGGCAATCAGAAA
GCAAACTGCAAAACTATGGGATATTGCATTAGGTAAGGTGACCAAAGATAAAATCAATAAAGAAGATACAGCAGCAGCTAGCAAATTTACTTTGAGTTATGAATATCACAGAT
TCAGGCTAATCAATATGGCAGACAATTTAATTGCTAGACTAATGGTGAAACCAAAATCAGATTGGCTAATATCAGTCATGAAGGGGCATCTTAACAGACTATATGAGCACTGG
AAAGTAATGAATGAAATTATCCTAAGTATGGACAACGATTATTCAATTGCAACAACGTTCGAATATTATGCACCATCATGTCTGTGTTTAGCTACGCAGACTTTCCTTATTGTGA
GGAATATGGAAATGGATGATGTCAAGATGATGGTTGCAGTATATAAAAGATTTCTTAACCTAGGAATGTTTTTGCAGAGTGCCAAAGTATGCAGCCTTGCCGATAGTCATACAT
TCAGAGATTTTTCTAGATCTTTTTCCTTTATTACGATAATTTCAAGATTGATGATAATCGAATTTATGCAAATTAAAGAATTGACGAAGGTAGAGTTTATTGAGAAGTTTTCTGAA
GTATGCCCTGACCTTGCAGATCTACCTCCGATGCTTCTAGATCCAAACTCTTGCTTATATTTTTCATTGTTACAGCAGATTAAGAAATCTGGTTTTACGTTGTCATTCAAAAAAATT
CTTGAAGACGCTAGAATGATGGACTTCAATTACGACCGCAATTTGGACTCAGAGGCCATTAAAAAGTGCAATGGTGAATTTAGCAAGTCAATGCCTTCCTGTACCAATGTCTCA
GATACCACCACCGCTGTTTCTGACAATAGTGCTAAGAAGAAAGCTTCAATGGGGTCGGCGAGGGTAAATTCAACTGATACACTAACTGCATCTCCCTTATCGGGCTTAAGGAAT
CAAACGCAGTTGGATTCTAAAGACAGTGTTCCATCTCTCGAGGCTTATACACCAATTGATTCTGTCTCTGACGTGCCCACTGGGGAGATCAACGTTCCATTCCCTCCTGTTTATA
ATCAAAATGGATTGGATCAGCAAACCACTTATAATTTGGGAACTTTAGATGAGTTTGTTAACAAGGGAGATTTGAATGAACTCTATAATAGCCTATGGGGTGACCTATTTTCTG
ATGTTTACTTGTGA

>25

ATGCAAACATTAGAAACTACATCAAAATCAAATCCAGGGGAAGTCAAAGCACAGAAGCCTAGTACAAGAAGAACAAAAGTTGGAAAAGCTTGTGATAGCTGTAGAAGGAGG
AAAATAAAATGTAATGGGCTAAAACCTTGTCCATCTTGTACAATCTATGGCTGTGAATGTACATATACTGATGCAAAATCGACAAAAAATCTCAAATCAAATGATGCAGGTAAA

TCAAAACCAACAGGGAGAGTATCAAAGAATAAAGAAACTACTAGAATCGACAAAGATATTAGGAAATCAGAGCAGCAGTATGTCCCTATTAATGCTAATATTCATGTTGGTCC
CAGGTTCCCCTCCGAGAATATATTGAATGGATATCCACAATGTGGAGCACCACAGAACAATGTTGTGGGTAATCCACTAGCGGTTAATACTCAATGCCATAGAGGTCTTTCTGA
AACTCCTATGTCCTCAACATTCAAAGAATCTAACTTAAGAGATGATCGGCTACTACAGTCATCAGATACAGATGATATGAGGAATGGTGACTCGGAAGAAAGGGACTTGAAAG
GGAGTGACAGCGAGAATGTCAAAAGTAAAGACAATAAAAGTGATCCTTTGATTATATACAAAGATGATACACATATTGAAAGCACGGTTAATAAACTAACACAGGCAGTTAAT
GAACTCAAATCACTTCAAAATGCACCCAGTTCGATAAAATCATCCATTAACGCCATTGAGTTACAACTTAGAAACATTTTAGACAACTGGAAACCAGAGGTAGATTTCGAGAAA
GCAAAGATTAATGAAAGTGCCACCACTAAGTCACTTGAAACAAACTTGCTGAGGAATAAATACACTAATCACGTTCATTTAACAAGATTTAGGATATGGATAGATTATAAAAAT
GCGAACAAAAACAATCATTTTATGGGAGAGTGTGGATTTAGTCTTGCAGAATCTTTTTTTGCTTCTAATCAGCCATTGGTCGATGAATTGTTTGGGTTGTATTCCCAGGTAGAGG
CCTTTTCTTTGCAAGGTCTTGGTTACTGTGTTCACCTTTATGAGCCATATATGAAAACTGAGGAAGCGATAAAACTGATGAAAGAGACCTTATATATTATACTACGGTTTATTGA
TATATGTGTTCACCATATCAATGAAGAGTCGATATCGATTGCCAACCCGTTAGAAACATATTTACGAAAAAAACATCTAATGCCTATGACTCCTACACCAAGGTCGTCCTATGGA
AGTCCACAAAGTGCTAGTACAAAGAGCTTGGTAAGTAAGATAATAGAGAGAATACCGCAACCGTTTATTGAGAGTGTAACTAATGTGTCGAGTCTTCAACTATTAGATCTTCGA
GATGACGAGTCAAAAATGTTTGGAACATTGCTGAACATGTGTAAGTCTATAAGGCGAAAATTTGACTCTGTTATGAGCGATTACGATTCCATTGTCACAGAAAAATCCGAAGG
CGAACAAAATGATGGTAAAGTAACTGTAGCTGAGTTCACATCTTTGTGTGAAGCGGAAGAAATGCTCTTAGCATTATGCTATAACTATTATAATCTGACGTTATACAGTTTCTTT
GAATTTGGGACTAATATTGAATACATGGAACATCTGTTGCTTCTTCTTGAAGAACAGCTTGCTCTCGACGAATACTATGGTTTTGAAAAGGTCTTGAATGTAGCTGTTGCAAATG
CTAAAAAAATGGGTTTCCACCGTTGGGAGTTTTACGTCGGCTATGAAGAGTCGACTGCTGAAAAGAGGCGGCTACTATGGTGGAAGTTATACAATTATGAAAAAGCCAGTACT
ATGAAGAAGGGTTTTTTTTCTGTGATTGATGATGCTACTGTCAACTGTTTATTACCTAAGATTTTTAGAAACTTTGGCTATCTGGATAGGGTGGAGTTTCTAGAAAATATTCAAA
AGCCAATGGATCTTAGTGTGTTTTCCGATGTTCCAATTTCTGTCCTTTGTAAATACGGTGAGTTGGCCCTTACAATAGTTACCAGTGAGTTTCATGAAAAATTTTTATATGCTGAT
AGATACACTTCTATTCGAAATTCCGCGAAACCGCCGACATTAAAAAACCAATTAATTAAGGAAATTGTGGATGGTATAGCTTATACAGAGACATCATATGAGGCAATCAGAAA
GCAAACTGCAAAACTATGGGATATTGCATTAGGTAAGGTGACCAAAGATAAAATCAATAAAGAAGATACAGCAGCAGCTAGCAAATTTACTTTGAGTTATGAATATCACAGAT
TCAGGCTAATCAATATGGCAGACAATTTAATTGCTAGACTAATGGTGAAACCAAAATCAGATTGGCTAATATCAGTCATGAAGGGGCATCTTAACAGACTATATGAGCACTGG
AAAGTAATGAATGAAATTATCCTAAGTATGGACAACGATTATTCAATTGCAACAACGTTCGAATATTATGCACCATCATGTCTGTGTTTAGCTACGCAGACTTTCCTTATTGTGA
GGAATATGGAAATGGATGATGTCAAGATGATGGTTGCAGTATATAAAAGATTTCTTAACCTAGGAATGTTTTTGCAGAGTGCCAAAGTATGCAGCCTTGCCGATAGTCATACAT
TCAGAGATTTTTCTAGATCTTTTTCCTTTATTACGATAATTTCAAGATTGATGATAATCGAATTTATGCAAATTAAAGAATTGACGAAGGTAGAGTTTATTGAGAAGTTTTCTGAA
GTATGCCCTGACCTTGCAGATCTACCTCCGATGCTTCTAGATCCAAACTCTTGCTTATATTTTTCATTGTTACAGCAGATTAAGAAATCTGGTTTTACGTTGTCATTCAAAAAAATT
CTTGAAGACGCTAGAATGATGGACTTCAATTACGACCGCAATTTGGACTCAGAGGCCATTAAAAAGTGCAATGGTGAATTTAGCAAGTCAATGCCTTCCTGTACCAATGTCTCA
GATACCACCACCGCTGTTTCTGACAATAGTGCTAAGAAGAAAGCTTCAATGGGGTCGGCGAGGGTAAATTCAACTGATACACTAACTGCATCTCCCTTATCGGGCTTAAGGAAT
CAAACGCAGTTGGATTCTAAAGACAGTGTTCCATCTCTCGAGGCTTATACACCAATTGATTCTGTCTCTGACGTGCCCACTGGGGAGATCAACGTTCCATTCCCTCCTGTTTATA
ATCAAAATGGATTGGATCAGCAAACCACTTATAATTTGGGAACTTTAGATGAGTTTGTTAACAAGGGAGATTTGAATGAACTCTATAATAGCCTATGGGGTGACCTATTTTCTG
ATGTTTACTTGTGA

>26

ATGCAAACATTAGAAACTACATCAAAATCAAATCCAGGGGAAGTCAAAGCACAGAAGCCTAGTACAAGAAGAACAAAAGTTGGAAAAGCTTGTGATAGCTGTAGAAGGAGG
AAAATAAAATGTAATGGGCTAAAACCTTGTCCATCTTGTACAATCTATGGTTGTGAATGTACATATACTGATGCAAAATCGACAAAAAATCTCAAATCAAATGATGCAGGTAAA
CCAAAACCAACAGGGAGAGTATCAAAGAATAAAGAAACTACTAGAATCGACAAAGATATTAGGAAATCAGAGCAGCAGTATGTCCCTATTAATGCTAATATTCATGTTGGTCC
CAGGTTCCCCTCCGAGAATATATTGAATGGATATCCACAATGTGGAGCACCACAGAACAATGTTGTGGGTAATCCACTAGCGGTTAATCCTCAATGCCATAGAGGTCTTTCTGA
AACTCCTATGTCCTCAACATTCAAAGAATCTAACTTAAGAGATGATCGGCTACTACAGTCATCAGATACAGATGATATGAGGAATGGTGACTCGGAAGAAAGGGACTTGAAAG
GGAGTGACAGCGAGAATGTCAAAAGTAAAGACAATAAAAGTGATCCTTTGATTATATACAAAGATGATACACATATTGAAAGCACGGTTAATAAACTAACACAGGCAGTTAAT
GAACTCAAATCACTTCAAAATGCACCTAGTTCGATAAAATCATCCATTGACGCCATTGAGTTACAACTTAGAAACATTTTAGACAATTGGAAACCAGAGGTAGATTTCGAGAAA
GCAAAGATTAATGAAAGTGCCACCACTAAGTCACTTGAAACAAACTTGCTGAGGAATAAATACACTAATCACGTTCATCTAACAAGATTTAGGATATGGATAGATTATAAAAAT
GCGAACAAAAACAATCATTTTATGGGAGAGTGTGGATTTAGTCTTGCAGAATCTTTTTTTGCTTCTAATCAGCCATTGGTCGATGAATTGTTTGGGTTGTATTCCCAGGTAGAGG
CCTTTTCTTTGCAAGGTCTTGGTTACTGTGTTCACCTTTATGAGCCATATATGAAAACTGAGGAAGCGATAAAACTGATGAAAGAGACCTTATATATTATACTACGGTTTATTGA
TATATGTGTTCACCATATCAATGAAGAGTCGATATCGATTGCCAACCCGTTAGAAACATATTTACGAAAAAAACATCTAATGCCTATGACTCCTACACCAAGGTCGTCCTATGGA
AGTCCACAAAGTGCTAGTACAAAGAGCTTGGTAAGTAAGATAATAGAGAGAATACCGCAACCGTTTATTGAGAGTGTAACTAATGTGTCGAGTCTTCAACTATTAGATCTTCGA
GATGACGAGTCAAAAATGTTTGGAACATTGCTGAACATGTGTAAGTCTATAAGGCGAAAATTTGACTCTGTTATGAGCGATTACGATTCCATTGTCACAGAAAAATCCGAAGG
CGAACAAAATGATGGTAAAGTAACTGTAGCTGAGTTCACATCTTTGTGTGAAGCGGAAGAAATGCTCTTAGCATTATGCTATAACTATTATAATCTGACGTTATACAGTTTCTTT
GAATTTGGGACTAATATTGAATACATGGAACATCTGTTGCTTCTTCTTGAAGAACAGCTTGCTCTCGACGAATACTATGGTTTTGAAAAGGTCTTGAATGTAGCTGTTGCAAATG
CTAAAAAAATGGGTTTCCACCGTTGGGAGTTTTACGTCGGTTATGAAGAGTCGACTGCTGAAAAGAGGCGGCTACTATGGTGGAAGTTATACAATTATGAAAAAGCCAGTACT
ATGAAGAAGGGTTTTTTTTCTGTGATTGATGATGCTACTGTCAACTGTTTATTACCTAAGATTTTTAGAAACTTTGGCTATCTGGATAGGGTGGAGTTTCTAGAAAATATTCAAA
AGCCAATGGATCTTAGTGTGTTTTCCGATGTTCCAATTTCTGTCCTTTGTAAATACGGTGAGTTGGCCCTTACAATAGTTACCAGTGAGTTTCATGAAAAATTTTTATATGCTGAT
AGATACACTTCTATTCGAAATTCCGCGAAACCGCCGACATTAAAAAACCAATTAATTAAGGAAATTGTGGATGGTATAGCTTATACAGAGACATCATATGAGGCAATCAGAAA
GCAAACTGCAAAACTATGGGATATTGCATTAGGTAAGGTGACCAAAGATAAAATCAATAAAGAAGATACAGCAGCAGCTAGCAAATTTACTTTGAGTTATGAATATCACAGAT
TCAGGCTAATCAATATGGCAGACAATTTAATTGCTAGACTTATGGTGAAACCAAAATCAGATTGGCTAATATCAGTCATGAAGGGGCATCTTAACAGACTATATGAGCACTGGA
AAGTAATGAATGAAATTATCCTAAGTATGGACAACGATTATTCAATTGCAACAACGTTCGAATATTATGCACCATCATGTCTGTGTTTAGCTACGCAGACTTTCCTTATTGTGAG
GAATATGGAAATGGATGATGTCAAGATGATGGTTGCAGTATATAAAAGATTTCTTAACCTAGGAATGTTTCTGCAGAGTGCCAAAGTATGCAGCCTTGCCGATAGTCATACATT
CAGAGATTTTTCTAGATCTTTTTCCTTTATTACGATAATTTCAAGATTGATGATAATCGAATTTATGCAAATTAAAGAATTGACGAAGGTAGAGTTTATTGAGAAGTTTTCTGAAG
TATGCCCTGACCTTGCAGATCTACCTCCGATGCTTCTAGATCCAAACTCTTGCTTATATTTTTCATTGTTACAGCAGATTAAGAAATCTGGTTTTACGTTGTCATTCAAAAAAAATTC
TTGAAGACGCTAGAATGATGGACTTCAATTACGACCGCAATTTGGACTCAGAGGCCATTAAAAAGTGCAATGGTGAATTTAGCAAGTCAATGCCTTCCTGTACCAATGTCTCAG
ATACCACCACCGCTGTTTCTGACAACAGTGCTAAGAAGAAAGCTTCAATGGGGTCGGCGAGGGTAAATTCAACTGATACACTAACTGCATCTCCCTTATCGGGCTTAAGGAATC
AAACGCAGTTGGATTCTAAAGACAGTGTTCCATCTCTCGAGGCTTATACACCAATTGATTCTGTCTCTGACGTACCCACTGGGGAGATCAACGTTCCATTCCCTCCTGTTTATAAT
CAAAATGGATTGGATCAGCAAACCACTTATAATTTGGGAACTTTAGATGAGTTTGTTAACAAGGGAGATTTGAATGAACTCTATAATAGCCTATGGGGTGACCTATTTTCTGAT
GTTTACTTGTGA

>27

ATGCAAACATTAGAAACTACATCAAAATCAAATCCAGGGGAAGTCAAAGCACAGAAGCCTAGTACAAGAAGAACAAAAGTTGGAAAAGCTTGTGATAGCTGTAGAAGGAGG
AAAATAAAATGTAATGGGCTAAAACCTTGTCCATCTTGTACAATCTATGGTTGTGAATGTACATATACTGATGCAAAATCGACAAAAAATCTCAAATCAAATGATGCAGGTAAA
CCAAAACCAACAGGGAGAGTATCAAAGAATAAAGAAACTACTAGAATCGACAAAGATATTAGGAAATCAGAGCAGCAGTATGTACCTATTAATGCTAATATTCATGTTGGTCC
CAGGTTCCCCTCCGAGAATATATTGAATGGATATCCACAATGTGGAGCACCACAGAACAATGTTGTGGGTAATCCACTAGCGGTTAATCCTCAATGCCATAGAGGTCTTTCTGA
AACTCCTATGTCCTCAACATTCAAAGAATCTAACTTAAGAGATGATCGGCTACTACAGTCATCAGATACAGATGATATGAGGAATGGTGACTCGGAAGAAAGGGACTTGAAAG
GGAGTGACAGCGAGAATGTCAAAAGTAAAGACAATAAAAGTGATCCTTTGATTATATACAAAGATGATACACATATTGAAAGCACGGTTAATAAACTAACACAGGCAGTTAAT
GAACTCAAATCACTTCAAAATGCACCTAGTTCGATAAAATCATCCATTGACGCCATTGAGTTACAACTTAGAAACATTTTAGACAATTGGAAACCAGAGGTAGATTTCGAGAAA
GCAAAGATTAATGAAAGTGCCACCACTAAGTCACTTGAAACAAACTTGCTGAGGAATAAATACACTAATCACGTTCATCTAACAAGATTTAGGATATGGATAGATTATAAAAAT
GCGAACAAAAACAATCATTTTATGGGAGAGTGTGGATTTAGTCTTGCAGAATCTTTTTTTGCTTCTAATCAGCCATTGGTCGATGAATTGTTTGGGTTGTATTCCCAGGTAGAGG
CCTTTTCTTTGCAAGGTCTTGGTTACTGTGTTCACCTTTATGAGCCATATATGAAAACTGAGGAAGCGATAAAACTGATGAAAGAGACCTTATATATTATACTACGGTTTATTGA
TATATGTGTTCACCATATCAATGAAGAGTCGATATCGATTGCCAACCCGTTAGAAACATATTTACGAAAAAAACATCTAATGCCTATGACTCCTACACCAAGGTCGTCCTATGGA
AGTCCACAAAGTGCTAGTACAAAGAGCTTGGTAAGTAAGATAATAGAGAGAATACCGCAACCGTTTATTGAGAGTGTAACTAATGTGTCGAGTCTTCAACTATTAGATCTTCGA
GATGACGAGTCAAAAATGTTTGGAACATTGCTGAACATGTGTAAGTCTATAAGGCGAAAATTTGACTCTGTTATGAGCGATTACGATTCCATTGTCACAGAAAAATCCGAAGG
CGAACAAAATGATGGTAAAGTAACTGTAGCTGAGTTCACATCTTTGTGTGAAGCGGAAGAAATGCTCTTAGCATTATGCTATAACTATTATAATCTGACGTTATACAGTTTCTTT
GAATTTGGGACTAATATTGAATACATGGAACATCTGTTGCTTCTTCTTGAAGAACAGCTTGCTCTCGACGAATACTATGGTTTTGAAAAGGTCTTGAATGTAGCTGTTGCAAATG
CTAAAAAAATGGGTTTCCACCGTTGGGAGTTTTACGTCGGTTATGAAGAGTCGACTGCTGAAAAGAGGCGGCTACTATGGTGGAAGTTATACAATTATGAAAAAGCCAGTACT
ATGAAGAAGGGTTTTTTTTCTGTGATTGATGATGCTACTGTCAACTGTTTATTACCTAAGATTTTTAGAAACTTTGGCTATCTGGATAGGGTGGAGTTTCTAGAAAATATTCAAA
AGCCAATGGATCTTAGTGTGTTTTCCGATGTTCCAATTTCTGTCCTTTGTAAATACGGTGAGTTGGCCCTTACAATAGTTACCAGTGAGTTTCATGAAAAATTTTTATATGCTGAT
AGATACACTTCTATTCGAAATTCCGCGAAACCGCCGACATTAAAAAACCAATTAATTAAGGAAATTGTGGATGGTATAGCTTATACAGAGACATCATATGAGGCAATCAGAAA
GCAAACTGCAAAACTATGGGATATTGCATTAGGTAAGGTGACCAAAGATAAAATCAATAAAGAAGATACAGCAGCAGCTAGCAAATTTACTTTGAGTTATGAATATCACAGAT
TCAGGCTAATCAATATGGCAGACAATTTAATTGCTAGACTTATGGTGAAACCAAAATCAGATTGGCTAATATCAGTCATGAAGGGGCATCTTAACAGACTATATGAGCACTGGA
AAGTAATGAATGAAATTATCCTAAGTATGGACAACGATTATTCAATTGCAACAACGTTCGAATATTATGCACCATCATGTCTGTGTTTAGCTACGCAGACTTTCCTTATTGTGAG
GAATATGGAAATGGATGATGTCAAGATGATGGTTGCAGTATATAAAAGATTTCTTAACCTAGGAATGTTTCTGCAGAGTGCCAAAGTATGCAGCCTTGCCGATAGTCATACATT
CAGAGATTTTTCTAGATCTTTTTCCTTTATTACGATAATTTCAAGATTGATGATAATCGAATTTATGCAAATTAAAGAATTGACGAAGGTAGAGTTTATTGAGAAGTTTTCTGAAG
TATGCCCTGACCTTGCAGATCTACCTCCGATGCTTCTAGATCCAAACTCTTGCTTATATTTTTCATTGTTACAGCAGATTAAGAAATCTGGTTTTACGTTGTCATTCAAAAAAAATTC
TTGAAGACGCTAGAATGATGGACTTCAATTACGACCGCAATTTGGACTCAGAGGCCATTAAAAAGTGCAATGGTGAATTTAGCAAGTCAATGCCTTCCTGTACCAATGTCTCAG
ATACCACCACCGCTGTTTCTGACAACAGTGCTAAGAAGAAAGCTTCAATGGGGTCGGCGAGGGTAAATTCAACTGATACACTAACTGCATCTCCCTTATCGGGCTTAAGGAATC
AAACGCAGTTGGATTCTAAAGACAGTGTTCCATCTCTCGAGGCTTATACACCAATTGATTCTGTCTCTGACGTACCCACTGGGGAGATCAACGTTCCATTCCCTCCTGTTTATAAT

CAAAATGGATTGGATCAGCAAACCACTTATAATTTGGGAACTTTAGATGAGTTTGTTAACAAGGGAGATTTGAATGAACTCTATAATAGCCTATGGGGTGACCTATTTTCTGAT
GTTTACTTGTGA

>28

ATGCAAACATTAGAAACTACATCAAAATCAAATCCAGGGGAAGTCAAAGCACAGAAGCCTAGTACAAGAAGAACAAAAGTTGGAAAAGCTTGTGATAGCTGTAGAAGGAGG
AAAATAAAATGTAATGGGCTAAAACCTTGTCCATCTTGTACAATCTATGGTTGTGAATGTACATATACTGATGCAAAATCGACAAAAAATCTCAAATCAAATGATGCAGGTAAA
CCAAAACCAACAGGGAGAGTATCAAAGAATAAAGAAACTACTAGAATCGACAAAGATATTAGGAAATCAGAGCAGCAGTATGTACCTATTAATGCTAATATTCATGTTGGTCC
CAGGTTCCCCTCCGAGAATATATTGAATGGATATCCACAATGTGGAGCACCACAGAACAATGTTGTGGGTAATCCACTAGCGGTTAATCCTCAATGCCATAGAGGTCTTTCTGA
AACTCCTATGTCCTCAACATTCAAAGAATCTAACTTAAGAGATGATCGGCTACTACAGTCATCAGATACAGATGATATGAGGAATGGTGACTCGGAAGAAAGGGACTTGAAAG
GGAGTGACAGCGAGAATGTCAAAAGTAAAGACAATAAAAGTGATCCTTTGATTATATACAAAGATGATACACATATTGAAAGCACGGTTAATAAACTAACACAGGCAGTTAAT
GAACTCAAATCACTTCAAAATGCACCTAGTTCGATAAAATCATCCATTGACGCCATTGAGTTACAACTTAGAAACATTTTAGACAATTGGAAACCAGAGGTAGATTTCGAGAAA
GCAAAGATTAATGAAAGTGCCACCACTAAGTCACTTGAAACAAACTTGCTGAGGAATAAATACACTAATCACGTTCATCTAACAAGATTTAGGATATGGATAGATTATAAAAAT
GCGAACAAAAACAATCATTTTATGGGAGAGTGTGGATTTAGTCTTGCAGAATCTTTTTTTGCTTCTAATCAGCCATTGGTCGATGAATTGTTTGGGTTGTATTCCCAGGTAGAGG
CCTTTTCTTTGCAAGGTCTTGGTTACTGTGTTCACCTTTATGAGCCATATATGAAAACTGAGGAAGCGATAAAACTGATGAAAGAGACCTTATATATTATACTACGGTTTATTGA
TATATGTGTTCACCATATCAATGAAGAGTCGATATCGATTGCCAACCCGTTAGAAACATATTTACGAAAAAAACATCTAATGCCTATGACTCCTACACCAAGGTCGTCCTATGGA
AGTCCACAAAGTGCTAGTACAAAGAGCTTGGTAAGTAAGATAATAGAGAGAATACCGCAACCGTTTATTGAGAGTGTAACTAATGTGTCGAGTCTTCAACTATTAGATCTTCGA
GATGACGAGTCAAAAATGTTTGGAACATTGCTGAACATGTGTAAGTCTATAAGGCGAAAATTTGACTCTGTTATGAGCGATTACGATTCCATTGTCACAGAAAAATCCGAAGG
CGAACAAAATGATGGTAAAGTAACTGTAGCTGAGTTCACATCTTTGTGTGAAGCGGAAGAAATGCTCTTAGCATTATGCTATAACTATTATAATCTGACGTTATACAGTTTCTTT
GAATTTGGGACTAATATTGAATACATGGAACATCTGTTGCTTCTTCTTGAAGAACAGCTTGCTCTCGACGAATACTATGGTTTTGAAAAGGTCTTGAATGTAGCTGTTGCAAATG
CTAAAAAAATGGGTTTCCACCGTTGGGAGTTTTACGTCGGTTATGAAGAGTCGACTGCTGAAAAGAGGCGGCTACTATGGTGGAAGTTATACAATTATGAAAAAGCCAGTACT
ATGAAGAAGGGTTTTTTTTCTGTGATTGATGATGCTACTGTCAACTGTTTATTACCTAAGATTTTTAGAAACTTTGGCTATCTGGATAGGGTGGAGTTTCTAGAAAATATTCAAA
AGCCAATGGATCTTAGTGTGTTTTCCGATGTTCCAATTTCTGTCCTTTGTAAATACGGTGAGTTGGCCCTTACAATAGTTACCAGTGAGTTTCATGAAAAATTTTTATATGCTGAT
AGATACACTTCTATTCGAAATTCCGCGAAACCGCCGACATTAAAAAACCAATTAATTAAGGAAATTGTGGATGGTATAGCTTATACAGAGACATCATATGAGGCAATCAGAAA
GCAAACTGCAAAACTATGGGATATTGCATTAGGTAAGGTGACCAAAGATAAAATCAATAAAGAAGATGCAGCAGCAGCTAGCAAATTTACTTTGAGTTATGAATATCACAGAT
TCAGGCTAATCAATATGGCAGACAATTTAATTGCTAGACTTATGGTGAAACCAAAATCAGATTGGCTAATATCAGTCATGAAGGGGCATCTTAACAGACTATATGAGCACTGGA
AAGTAATGAATGAAATTATCCTAAGTATGGACAACGATTATTCAATTGCAACAACGTTCGAATATTATGCACCATCATGTCTGTGTTTAGCTACGCAGACTTTCCTTATTGTGAG
GAATATGGAAATGGATGATGTCAAGATGATGGTTGCAGTATATAAAAGATTTCTTAACCTAGGAATGTTTCTGCAGAGTGCCAAAGTATGCAGCCTTGCCGATAGTCATACATT
CAGAGATTTTTCTAGATCTTTTTCCTTTATTACGATAATTTCAAGATTGATGATAATCGAATTTATGCAAATTAAAGAATTGACGAAGGTAGAGTTTATTGAGAAGTTTTCTGAAG
TATGCCCTGACCTTGCAGATCTACCTCCGATGCTTCTAGATCCAAACTCTYGCTTATATTTTTCATTGTTACAGCAGATTAAGAAATCTGGTTTTACGTTGTCATTCAAAAAAATTC
TTGAAGACGCTAGAATGATGGACTTCAATTACGACCGCAATTTGGACTCAGAGGCCATTAAAAAGTGCAATGGTGAATTTAGCAAGTCAATGCCTTCCTGTACCAATGTCTCAG
ATACCACCACCGCTGTTTCTGACAACAGTGCTAAGAAGAAAGCTTCAATGGGGTCGGCGAGGGTAAATTCAACTGATACACTAACTGCATCTCCCTTATCGGGCTTAAGGAATC

AAACGCAGTTGGATTCTAAAGACAGTGTTCCATCTCTCGAGGCTTATACACCAATTGATTCTGTCTCTGACGTACCCACTGGGGAGATCAACGTTCCATTCCCTCCTGTTTATAAT
CAAAATGGATTGGATCAGCAAACCACTTATAATTTGGGAACTTTAGATGAGTTTGTTAACAAGGGAGATTTGAATGAACTCTATAATAGCCTATGGGGTGACCTATTTTCTGAT
GTTTACTTGTGA

>29

ATGCAAACATTAGAAACTACATCAAAATCAAATCCAGGGGAAGTCAAAGCACAGAAGCCTAGTACAAGAAGAACAAAAGTTGGAAAAGCTTGTGATAGCTGTAGAAGGAGG
AAAATAAAATGTAATGGGCTAAAACCTTGTCCATCTTGTACAATCTATGGTTGTGAATGTACATATACTGATGCAAAATCGACAAAAAATCTCAAATCAAATGATGCAGGTAAA
CCAAAACCAACAGGGAGAGTATCAAAGAATAAAGAAACTACTAGAATCGACAAAGATATTAGGAAATCAGAGCAGCAGTATGTCCCTATTAATGCTAATATTCATGTTGGTCC
CAGGTTCCCCTCCGAGAATATATTGAATGGATATCCACAATGTGGAGCACCACAGAACAATGTTGTGGGTAATCCACTAGCGGTTAATCCTCAATGCCATAGAGGTCTTTCTGA
AACTCCTATGTCCTCAACATTCAAAGAATCTAACTTAAGAGATGATCGGCTACTACAGTCATCAGATACAGATGATATGAGGAATGGTGACTCGGAAGAAAGGGACTTGAAAG
GGAGTGACAGCGAGAATGTCAAAAGTAAAGACAATAAAAGTGATCCTTTGATTATATACAAAGATGATACACATATTGAAAGCACGGTTAATAAACTAACACAGGCAGTTAAT
GAACTCAAATCACTTCAAAATGCACCTAGTTCGATAAAATCATCCATTGACGCCATTGAGTTACAACTTAGAAACATTTTAGACAATTGGAAACCAGAGGTAGATTTCGAGAAA
GCAAAGATTAATGAAAGTGCCACCACTAAGTCACTTGAAACAAACTTGCTGAGGAATAAATACACTAATCACGTTCATCTAACAAGATTTAGGATATGGATAGATTATAAAAAT
GCGAACAAAAACAATCATTTTATGGGAGAGTGTGGATTTAGTCTTGCAGAATCTTTTTTTGCTTCTAATCAGCCATTGGTCGATGAATTGTTTGGGTTGTATTCCCAGGTAGAGG
CCTTTTCTTTGCAAGGTCTTGGTTACTGTGTTCACCTTTATGAGCCATATATGAAAACTGAGGAAGCGATAAAACTGATGAAAGAGACCTTATATATTATACTACGGTTTATTGA
TATATGTGTTCACCATATCAATGAAGAGTCGATATCGATTGCCAACCCGTTAGAAACATATTTACGAAAAAAACATCTAATGCCTATGACTCCTACACCAAGGTCGTCCTATGGA
AGTCCACAAAGTGCTAGTACAAAGAGCTTGGTAAGTAAGATAATAGAGAGAATACCGCAACCGTTTATTGAGAGTGTAACTAATGTGTCGAGTCTTCAACTATTAGATCTTCGA
GATGACGAGTCAAAAATGTTTGGAACATTGCTGAACATGTGTAAGTCTATAAGGCGAAAATTTGACTCTGTTATGAGCGATTACGATTCCATTGTCACAGAAAAATCCGAAGG
CGAACAAAATGATGGTAAAGTAACTGTAGCTGAGTTCACATCTTTGTGTGAAGCGGAAGAAATGCTCTTAGCATTATGCTATAACTATTATAATCTGACGTTATACAGTTTCTTT
GAATTTGGGACTAATATTGAATACATGGAACATCTGTTGCTTCTTCTTGAAGAACAGCTTGCTCTCGACGAATACTATGGTTTTGAAAAGGTCTTGAATGTAGCTGTTGCAAATG
CTAAAAAAATGGGTTTCCACCGTTGGGAGTTTTACGTCGGTTATGAAGAGTCGACTGCTGAAAAGAGGCGGCTACTATGGTGGAAGTTATACAATTATGAAAAAGCCAGTACT
ATGAAGAAGGGTTTTTTTTCTGTGATTGATGATGCTACTGTCAACTGTTTATTACCTAAGATTTTTAGAAACTTTGGCTATCTGGATAGGGTGGAGTTTCTAGAAAATATTCAAA
AGCCAATGGATCTTAGTGTGTTTTCCGATGTTCCAATTTCTGTCCTTTGTAAATACGGTGAGTTGGCCCTTACAATAGTTACCAGTGAGTTTCATGAAAAATTTTTATATGCTGAT
AGATACACTTCTATTCGAAATTCCGCGAAACCGCCGACATTAAAAAACCAATTAATTAAGGAAATTGTGGATGGTATAGCTTATACAGAGACATCATATGAGGCAATTAGAAAG
CAAACTGCAAAACTATGGGATATTGCATTAGGTAAGGTGACCAAAGATAAAATCAATAAAGAAGATACAGCAGCAGCTAGCAAATTTACTTTGAGTTATGAATATCACAGATT
CAGGCTAATCAATATGGCAGACAATTTAATTGCTAGACTTATGGTGAAACCAAAATCAGATTGGCTAATATCAGTCATGAAGGGGCATCTTAACAGACTATATGAGCACTGGA
AAGTAATGAATGAAATTATCCTAAGTATGGACAACGATTATTCAATTGCAACAACGTTCGAATATTATGCACCATCATGTCTGTGTTTAGCTACGCAGACTTTCCTTATTGTGAG
GAATATGGAAATGGATGATGTCAAGATGATGGTTGCAGTATATAAAAGATTTCTTAACCTAGGAATGTTTCTGCAGAGTGCCAAAGTATGCAGCCTTGCCGATAGTCATACATT
CAGAGATTTTTCTAGATCTTTTTCCTTTATTACGATAATTTCAAGATTGATGATAATCGAATTTATGCAAATTAAAGAATTGACGAAGGTAGAGTTTATTGAGAAGTTTTCTGAAG
TATGCCCTGACCTTGCAGATCTACCTCCGATGCTTCTAGATCCAAACTCTTGCTTATATTTTTCATTGTTACAGCAGATTAAGAAATCTGGTTTTACGTTGTCATTCAAAAAAAATTC
TTGAAGACGCTAGAATGATGGACTTCAATTACGACCGCAATTTGGACTCAGAGGCCATTAAAAAGTGCAATGGTGAATTTAGCAAGTCAATGCCTTCCTGTACCAATGTCTCAG

ATACCACCACCGCTGTTTCTGACAACAGTGCTAAGAAGAAAGCTTCAATGGGGTCGGCGAGGGTAAATTCAACTGATACACTAACTGCATCTCCCTTATCGGGCTTAAGGAATC
AAACGCAGTTGGATTCTAAAGACAGTGTTCCATCTCTCGAGGCTTATACACCAATTGATTCTGTCTCTGACGTACCCACTGGGGAGATCAACGTTCCATTCCCTCCTGTTTATAAT
CAAAATGGATTGGATCAGCAAACCACTTATAATTTGGGAACTTTAGATGAGTTTGTTAACAAGGGAGATTTGAATGAACTCTATAATAGCCTATGGGGTGACCTATTTTCTGAT
GTTTACTTGTGA

>30

ATGCAAACATTAGAAACTACATCAAAATCAAATCCAGGGGAAGTCAAAGCACAGAAGCCTAGTACAAGAAGAACAAAAGTTGGAAAAGCTTGTGATAGCTGTAGAAGGAGG
AAAATAAAATGTAATGGGCTAAAACCTTGTCCATCTTGTACAATCTATGGTTGTGAATGTACATATACTGATGCAAAATCGACAAAAAATCTCAAATCAAATGATGCAGGTAAA
CCAAAACCAACAGGGAGAGTATCAAAGAATAAAGAAACTACTAGAATCGACAAAGATATTAGGAAATCAGAGCAGCAGTATGTCCCTATTAATGCTAATATTCATGTTGGTCC
CAGGTTCCCCTCCGAGAATATATTGAATGGATATCCACAATGTGGAGCACCACAGAACAATGTTGTGGGTAATCCACTAGCGGTTAATCCTCAATGCCATAGAGGTCTTTCTGA
AACTCCTATGTCCTCAACATTCAAAGAATCTAACTTAAGAGATGATCGGCTACTACAGTCATCAGATACAGATGATATGAGGAATGGTGACTCGGAAGAAAGGGACTTGAAAG
GGAGTGACAGCGAGAATGTCAAAAGTAAAGACAATAAAAGTGATCCTTTGATTATATACAAAGATGATACACATATTGAAAGCACGGTTAATAAACTAACACAGGCAGTTAAT
GAACTCAAATCACTTCAAAATGCACCTAGTTCGATAAAATCATCCATTGACGCCATTGAGTTACAACTTAGAAACATTTTAGACAATTGGAAACCAGAGGTAGATTTCGAGAAA
GCAAAGATTAATGAAAGTGCCACCACTAAGTCACTTGAAACAAACTTGCTGAGGAATAAATACACTAATCACGTTCATCTAACAAGATTTAGGATATGGATAGATTATAAAAAT
GCGAACAAAAACAATCATTTTATGGGAGAGTGTGGATTTAGTCTTGCAGAATCTTTTTTTGCTTCTAATCAGCCATTGGTCGATGAATTGTTTGGGTTGTATTCCCAGGTAGAGG
CCTTTTCTTTGCAAGGTCTTGGTTACTGTGTTCACCTTTATGAGCCATATATGAAAACTGAGGAAGCGATAAAACTGATGAAAGAGACCTTATATATTATACTACGGTTTATTGA
TATATGTGTTCACCATATCAATGAAGAGTCGATATCGATTGCCAACCCGTTAGAAACATATTTACGAAAAAAACATCTAATGCCTATGACTCCTACACCAAGGTCGTCCTATGGA
AGTCCACAAAGTGCTAGTACAAAGAGCTTGGTAAGTAAGATAATAGAGAGAATACCGCAACCGTTTATTGAGAGTGTAACTAATGTGTCGAGTCTTCAACTATTAGATCTTCGA
GATGACGAGTCAAAAATGTTTGGAACATTGCTGAACATGTGTAAGTCTATAAGGCGAAAATTTGACTCTGTTATGAGCGATTACGATTCCATTGTCACAGAAAAATCCGAAGG
CGAACAAAATGATGGTAAAGTAACTGTAGCTGAGTTCACATCTTTGTGTGAAGCGGAAGAAATGCTCTTAGCATTATGCTATAACTATTATAATCTGACGTTATACAGTTTCTTT
GAATTTGGGACTAATATTGAATACATGGAACATCTGTTGCTTCTTCTTGAAGAACAGCTTGCTCTCGACGAATACTATGGTTTTGAAAAGGTCTTGAATGTAGCTGTTGCAAATG
CTAAAAAAATGGGTTTCCACCGTTGGGAGTTTTACGTCGGTTATGAAGAGTCGACTGCTGAAAAGAGGCGGCTACTATGGTGGAAGTTATACAATTATGAAAAAGCCAGTACT
ATGAAGAAGGGTTTTTTTTCTGTGATTGATGATGCTACTGTCAACTGTTTATTACCTAAGATTTTTAGAAACTTTGGCTATCTGGATAGGGTGGAGTTTCTAGAAAATATTCAAA
AGCCAATGGATCTTAGTGTGTTTTCCGATGTTCCAATTTCTGTCCTTTGTAAATACGGTGAGTTGGCCCTTACAATAGTTACCAGTGAGTTTCATGAAAAATTTTTATATGCTGAT
AGATACACTTCTATTCGAAATTCCGCGAAACCGCCGACATTAAAAAACCAATTAATTAAGGAAATTGTGGATGGTATAGCTTATACAGAGACATCATATGAGGCAATCAGAAA
GCAAACTGCAAAACTATGGGATATTGCATTAGGTAAGGTGACCAAAGATAAAATCAATAAAGAAGATACAGCAGCAGCTAGCAAATTTACTTTGAGTTATGAATATCACAGAT
TCAGGCTAATCAATATGGCAGACAATTTAATTGCTAGACTTATGGTGAAACCAAAATCAGATTGGCTAATATCAGTCATGAAGGGGCATCTTAACAGACTATATGAGCACTGGA
AAGTAATGAATGAAATTATCCTAAGTATGGACAACGATTATTCAATTGCAACAACGTTCGAATATTATGCACCATCATGTCTGTGTTTAGCTACGCAGACTTTCCTTATTGTGAG
GAATATGGAAATGGATGATGTCAAGATGATGGTTGCAGTATATAAAAGATTTCTTAACCTAGGAATGTTTCTGCAGAGTGCCAAAGTATGCAGCCTTGCCGATAGTCATACATT
CAGAGATTTTTCTAGATCTTTTTCCTTTATTACGATAATTTCAAGATTGATGATAATCGAATTTATGCAAATTAAAGAATTGACGAAGGTAGAGTTTATTGAGAAGTTTTCTGAAG
TATGCCCTGACCTTGCAGATCTACCTCCGATGCTTCTAGATCCAAACTCTTGCTTATATTTTTCATTGTTACAGCAGATTAAGAAATCTGGTTTTACGTTGTCATTCAAAAAAATTC

TTGAAGACGCTAGAATGATGGACTTCAATTACGACCGCAATTTGGACTCAGAGGCCATTAAAAAGTGCAATGGTGAATTTAGCAAGTCAATGCCTTCCTGTACCAATGTCTCAG
ATACCACCACCGCTGTTTCTGACAACAGTGCTAAGAAGAAAGCTTCAATGGGGTCGGCGAGGGTAAATTCAACTGATACACTAACTGCATCTCCCTTATCGGGCTTAAGGAATC
AAACGCAGTTGGATTCTAAAGACAGTGTTCCATCTCTCGAGGCTTATACACCAATTGATTCTGTCTCTGACGTACCCACTGGGGAGATCAACGTTCCATTCCCTCCTGTTTATAAT
CAAAATGGATTGGATCAGCAAACCACTTATAATTTGGGAACTTTAGATGAGTTTGTTAACAAGGGAGATTTGAATGAACTCTATAATAGCCTATGGGGTGACCTATTTTCTGAT
GTTTACTTGTGA

>31

ATGCAAACATTAGAAACTACATCAAAATCAAATCCAGGGGAAGTCAAAGCACAGAAGCCTAGTACAAGAAGAACAAAAGTTGGAAAAGCTTGTGATAGCTGTAGAAGGAGG
AAAATAAAATGTAATGGGCTAAAACCTTGTCCATCTTGTACAATCTATGGTTGTGAATGTACATATACTGATGCAAAATCGACAAAAAATCTCAAATCAAATGATGCAGGTAAA
CCAAAACCAACAGGGAGAGTATCAAAGAATAAAGAAACTACTAGAATCGACAAAGATATTAGGAAATCAGAGCAGCAGTATGTCCCTATTAATGCTAATATTCATGTTGGTCC
CAGGTTCCCCTCCGAGAATATATTGAATGGATATCCACAATGTGGAGCACCACAGAACAATGTTGTGGGTAATCCACTAGCGGTTAATCCTCAATGCCATAGAGGTCTTTCTGA
AACTCCTATGTCCTCAACATTCAAAGAATCTAACTTAAGAGATGATCGGCTACTACAGTCATCAGATACAGATGATATGAGGAATGGTGACTCGGAAGAAAGGGACTTGAAAG
GGAGTGACAGCGAGAATGTCAAAAGTAAAGACAATAAAAGTGATCCTTTGATTATATACAAAGATGATACACATATTGAAAGCACGGTTAATAAACTAACACAGGCAGTTAAT
GAACTCAAATCACTTCAAAATGCACCTAGTTCGATAAAATCATCCATTGACGCCATTGAGTTACAACTTAGAAACATTTTAGACAATTGGAAACCAGAGGTAGATTTCGAGAAA
GCAAAGATTAATGAAAGTGCCACCACTAAGTCACTTGAAACAAACTTGCTGAGGAATAAATACACTAATCACGTTCATCTAACAAGATTTAGGATATGGATAGATTATAAAAAT
GCGAACAAAAACAATCATTTTATGGGAGAGTGTGGATTTAGTCTTGCAGAATCTTTTTTTGCTTCTAATCAGCCATTGGTCGATGAATTGTTTGGGTTGTATTCCCAGGTAGAGG
CCTTTTCTTTGCAAGGTCTTGGTTACTGTGTTCACCTTTATGAGCCATATATGAAAACTGAGGAAGCGATAAAACTGATGAAAGAGACCTTATATATTATACTACGGTTTATTGA
TATATGTGTTCACCATATCAATGAAGAGTCGATATCGATTGCCAACCCGTTAGAAACATATTTACGAAAAAAACATCTAATGCCTATGACTCCTACACCAAGGTCGTCCTATGGA
AGTCCACAAAGTGCTAGTACAAAGAGCTTGGTAAGTAAGATAATAGAGAGAATACCGCAACCGTTTATTGAGAGTGTAACTAATGTGTCGAGTCTTCAACTATTAGATCTTCGA
GATGACGAGTCAAAAATGTTTGGAACATTGCTGAACATGTGTAAGTCTATAAGGCGAAAATTTGACTCTGTTATGAGCGATTACGATTCCATTGTCACAGAAAAATCCGAAGG
CGAACAAAATGATGGTAAAGTAACTGTAGCTGAGTTCACATCTTTGTGTGAAGCGGAAGAAATGCTCTTAGCATTATGCTATAACTATTATAATCTGACGTTATACAGTTTCTTT
GAATTTGGGACTAATATTGAATACATGGAACATCTGTTGCTTCTTCTTGAAGAACAGCTTGCTCTCGACGAATACTATGGTTTTGAAAAGGTCTTGAATGTAGCTGTTGCAAATG
CTAAAAAAATGGGTTTCCACCGTTGGGAGTTTTACGTCGGTTATGAAGAGTCGACTGCTGAAAAGAGGCGGCTACTATGGTGGAAGTTATACAATTATGAAAAAGCCAGTACT
ATGAAGAAGGGTTTTTTTTCTGTGATTGATGATGCTACTGTCAACTGTTTATTACCTAAGATTTTTAGAAACTTTGGCTATCTGGATAGGGTGGAGTTTCTAGAAAATATTCAAA
AGCCAATGGATCTTAGTGTGTTTTCCGATGTTCCAATTTCTGTCCTTTGTAAATACGGTGAGTTGGCCCTTACAATAGTTACCAGTGAGTTTCATGAAAAATTTTTATATGCTGAT
AGATACACTTCTATTCGAAATTCCGCGAAACCGCCGACATTAAAAAACCAATTAATTAAGGAAATTGTGGATGGTATAGCTTATACAGAGACATCATATGAGGCAATCAGAAA
GCAAACTGCAAAACTATGGGATATTGCATTAGGTAAGGTGACCAAAGATAAAATCAATAAAGAAGATACAGCAGCAGCTAGCAAATTTACTTTGAGTTATGAATATCACAGAT
TCAGGCTAATCAATATGGCAGACAATTTAATTGCTAGACTTATGGTGAAACCAAAATCAGATTGGCTAATATCAGTCATGAAGGGGCATCTTAACAGACTATATGAGCACTGGA
AAGTAATGAATGAAATTATCCTAAGTATGGACAACGATTATTCAATTGCAACAACGTTCGAATATTATGCACCATCATGTCTGTGTTTAGCTACGCAGACTTTCCTTATTGTGAG
GAATATGGAAATGGATGATGTCAAGATGATGGTTGCAGTATATAAAAGATTTCTTAACCTAGGAATGTTTCTGCAGAGTGCCAAAGTATGCAGCCTTGCCGATAGTCATACATT
CAGAGATTTTTCTAGATCTTTTTCCTTTATTACGATAATTTCAAGATTGATGATAATCGAATTTATGCAAATTAAAGAATTGACGAAGGTAGAGTTTATTGAGAAGTTTTCTGAAG

TATGCCCTGACCTTGCAGATCTACCTCCGATGCTTCTAGATCCAAACTCTTGCTTATATTTTTCATTGTTACAGCAGATTAAGAAATCTGGTTTTACGTTGTCATTCAAAAAAATTC
TTGAAGACGCTAGAATGATGGACTTCAATTACGACCGCAATTTGGACTCAGAGGCCATTAAAAAGTGCAATGGTGAATTTAGCAAGTCAATGCCTTCCTGTACCAATGTCTCAG
ATACCACCACCGCTGTTTCTGACAACAGTGCTAAGAAGAAAGCTTCAATGGGGTCGGCGAGGGTAAATTCAACTGATACACTAACTGCATCTCCCTTATCGGGCTTAAGGAATC
AAACGCAGTTGGATTCTAAAGACAGTGTTCCATCTCTCGAGGCTTATACACCAATTGATTCTGTCTCTGACGTACCCACTGGGGAGATCAACGTTCCATTCCCTCCTGTTTATAAT
CAAAATGGATTGGATCAGCAAACCACTTATAATTTGGGAACTTTAGATGAGTTTGTTAACAAGGGAGATTTGAATGAACTCTATAATAGCCTATGGGGTGACCTATTTTCTGAT
GTTTACTTGTGA

>32

ATGCAAACATTAGAAACTACATCAAAATCAAATCCAGGGGAAGTCAAAGCACAGAAGCCTAGTACAAGAAGAACAAAAGTTGGAAAAGCTTGTGATAGCTGTAGAAGGAGG
AAAATAAAATGTAATGGGCTAAAACCTTGTCCATCTTGTACAATCTATGGTTGTGAATGTACATATACTGATGCAAAATCGACAAAAAATCTCAAATCAAATGATGCAGGTAAA
CCAAAACCAACAGGGAGAGTATCAAAGAATAAAGAAACTACTAGAATCGACAAAGATATTAGGAAATCAGAGCAGCAGTATGTCCCTATTAATGCTAATATTCATGTTGGTCC
CAGGTTCCCCTCCGAGAATATATTGAATGGATATCCACAATGTGGAGCACCACAGAACAATGTTGTGGGTAATCCACTAGCGGTTAATCCTCAATGCCATAGAGGTCTTTCTGA
AACTCCTATGTCCTCAACATTCAAAGAATCTAGCTTAAGAGATGATCGGCTACTACAGTCATCAGATACAGATGATATGAGGAATGGTGACTCGGAAGAAAGGGACTTGAAAG
GGAGTGACAGCGAGAATGTCAAAAGTAAAGACAATAAAAGTGATCCTTTGATTATATACAAAGATGATACACATATTGAAAGCACGGTTAATAAACTAACACAGGCAGTTAAT
GAACTCAAATCACTTCAAAATGCACCTAGTTCGATAAAATCATCCATTGACGCCATTGAGTTACAACTTAGAAACATTTTAGACAATTGGAAACCAGAGGTAGATTTCGAGAAA
GCAAAGATTAATGAAAGTGCCACCACTAAGTCACTTGAAACAAACTTGCTGAGGAATAAATACACTAATCACGTTCATCTAACAAGATTTAGGATATGGATAGATTATAAAAAT
GCGAACAAAAACAATCATTTTATGGGAGAGTGTGGATTTAGTCTTGCAGAATCTTTTTTTGCTTCTAATCAGCCATTGGTCGATGAATTGTTTGGGTTGTATTCCCAGGTAGAGG
CCTTTTCTTTGCAAGGTCTTGGTTACTGTGTTCACCTTTATGAGCCATATATGAAAACCGAGGAAGCGATAAAACTGATGAAAGAGACCTTATATATTATACTACGGTTTATTGA
TATATGTGTTCACCATATCAATGAAGAGTCGATATCGATTGCCAACCCGTTAGAAACATATTTACGAAAAAAACATCTAATGCCTATGACTCCTACACCAAGGTCGTCCTATGGA
AGTCCACAAAGTGCTAGTACAAAGAGCTTGGTAAGTAAGATAATAGAGAGAATACCGCAACCGTTTATTGAGAGTGTAACTAATGTGTCGAGTCTTCAACTATTAGATCTTCGA
GATGACGAGTCAAAAATGTTTGGAACATTGCTGAACATGTGTAAGTCTATAAGGCGAAAATTTGACTCTGTTATGAGCGATTACGATTCCATTGTCACAGAAAAATCCGAAGG
CGAACAAAATGATGGTAAAGTAACTGTAGCTGAGTTCACATCTTTGTGTGAAGCGGAAGAAATGCTCTTAGCATTATGCTATAACTATTATAATCTGACGTTATACAGTTTCTTT
GAATTTGGGACTAATATTGAATACATGGAACATCTGTTGCTTCTTCTTGAAGAACAGCTTGCTCTCGACGAATACTATGGTTTTGAAAAGGTCTTGAATGTAGCTGTTGCAAATG
CTAAAAAAATGGGTTTCCACCGTTGGGAGTTTTACGTCGGTTATGAAGAGTCGACTGCTGAAAAGAGGCGGCTACTATGGTGGAAGTTATACAATTATGAAAAAGCCAGTACT
ATGAAGAAGGGTTTTTTTTCTGTGATTGATGATGCTACTGTCAACTGTTTATTACCTAAGATTTTTAGAAACTTTGGCTATCTGGATAGGGTGGAGTTTCTAGAAAATATTCAAA
AGCCAATGGATCTTAGTGTGTTTTCCGATGTTCCAATTTCTGTCCTTTGTAAATACGGTGAGTTGGCCCTTACAATAGTTACCAGTGAGTTTCATGAAAAATTTTTATATGCTGAT
AGATACACTTCTATTCGAAATTCCGCGAAACCGCCGACATTAAAAAACCAATTAATTAAGGAAATTGTGGATGGTATAGCTTATACAGAGACATCATATGAGGCAATCAGAAA
GCAAACTGCAAAACTATGGGATATTGCATTAGGTAAGGTGACCAAAGATAAAATCAATAAAGAAGATACAGCAGCAGCTAGCAAATTTACTTTGAGTTATGAATATCACAGAT
TCAGGCTAATCAATATGGCAGACAATTTAATTGCTAGACTTATGGTGAAACCAAAATCAGATTGGCTAATATCAGTCATGAAGGGGCATCTTAACAGACTATATGAGCACTGGA
AAGTAATGAATGAAATTATCCTAAGTATGGACAACGATTATTCAATTGCAACAACGTTCGAATATTATGCACCATCATGTCTGTGTTTAGCTACGCAGACTTTCCTTATTGTGAG
GAATATGGAAATGGATGATGTCAAGATGATGGTTGCAGTATATAAAAGATTTCTTAACCTAGGAATGTTTCTGCAGAGTGCCAAAGTATGCAGCCTTGCCGATAGTCATACATT

CAGAGATTTTTCTAGATCTTTTTCCTTTATTACGATAATTTCAAGATTGATGATAATCGAATTTATGCAAATTAAAGAATTGACGAAGGTAGAGTTTATTGAGAAGTTTTCTGAAG
TATGCCCTGACCTTGCAGATCTACCTCCGATGCTTCTAGATCCAAACTCTTGCTTATATTTTTCATTGTTACAGCAGATTAAGAAATCTGGTTYTACGTTGTCATTCAAAAAAATTC
TTGAAGACGCTAGAATGATGGACTTCAATTACGACCGCAATTTGGACTCAGAGGCCATTAAAAAGTGCAATGGTGAATTTAGCAAGTCAATGCCTTCCTGTACCAATGTCTCAG
ATACCACCACCGCTGTTTCTGACAACAGTGCTAAGAAGAAAGCTTCAATGGGGTCGGCGAGGGTAAATTCAACTGATACACTAACTGCATCTCCCTTATCGGGCTTAAGGAATC
AAACGCAGTTGGATTCTAAAGACAGTGTTCCATCTCTCGAGGCTTATACACCAATTGATTCTGTCTCTGACGTACCCACTGGGGAGATCAACGTTCCATTCCCTCCTGTTTATAAT
CAAAATGGATTGGATCAGCAAACCACTTATAATTTGGGAACTTTAGATGAGTTTGTTAACAAGGGAGATTTGAATGAACTCTATAATAGCCTATGGGGTGACCTATTTTCTGAT
GTTTACTTGTGA

>33

ATGCAAACATTAGAAACTACATCAAAATCAAATCCAGGGGAAGTCAAAGCACAGAAGCCTAGTACAAGAAGAACAAAAGTTGGAAAAGCTTGTGATAGCTGTAGAAGGAGG
AAAATAAAATGTAATGGGCTAAAACCTTGTCCATCTTGTACAATCTATGGTTGTGAATGTACATATACTGATGCAAAATCGACAAAAAATCTCAAATCAAATGATGCAGGTAAA
CCAAAACCAACAGGGAGAGTATCAAAGAATAAAGAAACTACTAGAATCGACAAAGATATTAGGAAATCAGAGCAGCAGTATGTCCCTATTAATGCTAATATTCATGTTGGTCC
CAGGTTCCCCTCCGAGAATATATTGAATGGATATCCACAATGTGGAGCACCACAGAACAATGTTGTGGGTAATCCACTAGCGGTTAATCCTCAATGCCATAGAGGTCTTTCTGA
AACTCCTATGTCCTCAACATTCAAAGAATCTAACTTAAGAGATGATCGGCTACTACAGTCATCAGATACAGATGATATGAGGAATGGTGACTCGGAAGAAAGGGACTTGAAAG
GGAGTGACAGCGAGAATGTCAAAAGTAAAGACAATAAAAGTGATCCTTTGATTATATACAAAGATGATACACATATTGAAAGCACGGTTAATAAACTAACACAGGCAGTTAAT
GAACTCAAATCACTTCAAAATGCACCTAGTTCGATAAAATCATCCATTGACGCCATTGAGTTACAACTTAGAAACATTTTAGACAATTGGAAACCAGAGGTAGATTTCGAGAAA
GCAAAGATTAATGAAAGTGCCACCACTAAGTCACTTGAAACAAACTTGCTGAGGAATAAATACACTAATCACGTTCATCTAACAAGATTTAGGATATGGATAGATTATAAAAAT
GCGAACAAAAACAATCATTTTATGGGAGAGTGTGGATTTAGTCTTGCAGAATCTTTTTTTGCTTCTAATCAGCCATTGGTCGATGAATTGTTTGGGTTGTATTCCCAGGTAGAGG
CCTTTTCTTTGCAAGGTCTTGGTTACTGTGTTCACCTTTATGAGCCATATATGAAAACTGAGGAAGCGATAAAACTGATGAAAGAGACCTTATATATTATACTACGGTTTATTGA
TATATGTGTTCACCATATCAATGAAGAGTCGATATCGATTGCCAACCCGTTAGAAACATATTTACGAAAAAAACATCTAATGCCTATGACTCCTACACCAAGGTCGTCCTATGGA
AGTCCACAAAGTGCTAGTACAAAGAGCTTGGTAAGTAAGATAATAGAGAGAATACCGCAACCGTTTATTGAGAGTGTAACTAATGTGTCGAGTCTTCAACTATTAGATCTTCGA
GATGACGAGTCAAAAATGTTTGGAACATTGCTGAACATGTGTAAGTCTATAAGGCGAAAATTTGACTCTGTTATGAGCGATTACGATTCCATTGTCACAGAAAAATCCGAAGG
CGAACAAAATGATGGTAAAGTAACTGTAGCTGAGTTCACATCTTTGTGTGAAGCGGAAGAAATGCTCTTAGCATTATGCTATAACTATTATAATCTGACGTTATACAGTTTCTTT
GAATTTGGGACTAATATTGAATACATGGAACATCTGTTGCTTCTTCTTGAAGAACAGCTTGCTCTCGACGAATACTATGGTTTTGAAAAGGTCTTGAATGTAGCTGTTGCAAATG
CTAAAAAAATGAGTTTCCACCGTTGGGAGTTTTACGTCGGTTATGAAGAGTCGACTGCTGAAAAGAGGCGGCTACTATGGTGGAAGTTATACAATTATGAAAAAGCCAGTACT
ATGAAGAAGGGTTTTTTTTCTGTGATTGATGATGCTACTGTCAACTGTTTATTACCTAAGATTTTTAGAAACTTTGGCTATCTGGATAGGGTGGAGTTTCTAGAAAATATTCAAA
AGCCAATGGATCTTAGTGTGTTTTCCGATGTTCCAATTTCTGTCCTTTGTAAATACGGTGAGTTGGCCCTTACAATAGTTACCAGTGAGTTTCATGAAAAATTTTTATATGCTGAT
AGATACACTTCTATTCGAAATTCCGCGAAACCGCCGACATTAAAAAACCAATTAATTAAGGAAATTGTGGATGGTATAGCTTATACAGAGACATCATATGAGGCAATCAGAAA
GCAAACTGCAAAACTATGGGATATTGCATTAGGTAAGGTGACCAAAGATAAAATCAATAAAGAAGATACAGCAGCAGCTAGCAAATTTACTTTGAGTTATGAATATCACAGAT
TCAGGCTAATCAATATGGCAGACAATTTAATTGCTAGACTTATGGTGAAACCAAAATCAGATTGGCTAATATCAGTCATGAAGGGGCATCTTAACAGACTATATGAGCACTGGA
AAGTAATGAATGAAATTATCCTAAGTATGGACAACGATTATTCAATTGCAACAACGTTCGAATATTATGCACCATCATGTCTGTGTTTAGCTACGCAGACTTTCCTTATTGTGAG

GAATATGGAAATGGATGATGTCAAGATGATGGTTGCAGTATATAAAAGATTTCTTAACCTAGGAATGTTTCTGCAGAGTGCCAAAGTATGCAGCCTTGCCGATAGTCATACATT
CAGAGATTTTTCTAGATCTTTTTCCTTTATTACGATAATTTCAAGATTGATGATAATCGAATTTATGCAAATTAAAGAATTGACGAAGGTAGAGTTTATTGAGAAGTTTTCTGAAG
TATGCCCTGACCTTGCAGATCTACCTCCGATGCTTCTAGATCCAAACTCTTGCTTATATTTTTCATTGTTACAGCAGATTAAGAAATCTGGTTTTACGTTGTCATTCAAAAAAATTC
TTGAAGACGCTAGAATGATGGACTTCAATTACGACCGCAATTTGGACTCAGAGGCCATTAAAAAGTGCAATGGTGAATTTAGCAAGTCAATGCCTTCCTGTACCAATGTCTCAG
ATACCACCACCGCTGTTTCTGACAACAGTGCTAAGAAGAAAGCTTCAATGGGGTCGGCGAGGGTAAATTCAACTGATACACTAACTGCATCTCCCTTATCGGGCTTAAGGAATC
AAACGCAGTTGGATTCTAAAGACAGTGTTCCATCTCTCGAGGCTTATACACCAATTGATTCTGTCTCTGACGTACCCACTGGGGAGATCAACGTTCCATTCCCTCCTGTTTATAAT
CAAAATGGATTGGATCAGCAAACCACTTATAATTTGGGAACTTTAGATGAGTTTGTTAACAAGGGAGATTTGAATGAACTCTATAATAGCCTATGGGGTGACCTATTTTCTGAT
GTTTACTTGTGA

>34

ATGCAAACATTAGAAACTACATCAAAATCAAATCCAGGGGAAGTCAAAGCACAGAAGCCTAGTACAAGAAGAACAAAAGTTGGAAAAGCTTGTGATAGCTGTAGAAGGAGG
AAAATAAAATGTAATGGGCTAAAACCTTGTCCATCTTGTACAATCTATGGCTGTGAATGTACATATACTGATGCAAAATCGACAAAAAATCTCAAATCAAATGATGCAGGTAAA
TCAAAACCAACAGGGAGAGTATCAAAGAATAAAGAAACTACTAGAATCGACAAAGATATTAGGAAATCAGAGCAGCAGTATGTCCCTATTAATGCTAATATTCATGTTGGTCC
CAGGTTCCCCTCCGAGAATATATTGAATGGATATCCACAATGTGGAGCACCACAGAACAATGTTGTGGGTAATCCACTAGCGGTTAATACTCAATGCCATAGAGGTCTTTCTGA
AACTCCTATGTCCTCAACATTCAAAGAATCTAACTTAAGAGATGATCGGCTACTACAGTCATCAGATACAGATGATATGAGGAATGGTGACTCGGAAGAAAGGGACTTGAAAG
GGAGTGACAGCGAGAATGTCAAAAGTAAAGACAATAAAAGTGATCCTTTGATTATATACAAAGATGATACACATATTGAAAGCACGGTTAATAAACTAACACAGGCAGTTAAT
GAACTCAAATCACTTCAAAATGCACCCAGTTCGATAAAATCATCCATTAACGCCATTGAGTTACAACTTAGAAACATTTTAGACAACTGGAAACCAGAGGTAGATTTCGAGAAA
GCAAAGATTAATGAAAGTGCCACCACTAAGTCACTTGAAACAAACTTGCTGAGGAATAAATACACTAATCACGTTCATTTAACAAGATTTAGGATATGGATAGATTATAAAAAT
GCGAACAAAAACAATCATTTTATGGGAGAGTGTGGATTTAGTCTTGCAGAATCTTTTTTTGCTTCTAATCAGCCATTGGTCGATGAATTGTTTGGGTTGTATTCCCAGGTAGAGG
CCTTTTCTTTGCAAGGTCTTGGTTACTGTGTTCACCTTTATGAGCCATATATGAAAACTGAGGAAGCGATAAAACTGATGAAAGAGACCTTATATATTATACTACGGTTTATTGA
TATATGTGTTCACCATATCAATGAAGAGTCGATATCGATTGCCAACCCGTTAGAAACATATTTACGAAAAAAACATCTAATGCCTATGACTCCTACACCAAGGTCGTCCTATGGA
AGTCCACAAAGTGCTAGTACAAAGAGCTTGGTAAGTAAGATAATAGAGAGAATACCGCAACCGTTTATTGAGAGTGTAACTAATGTGTCGAGTCTTCAACTATTAGATCTTCGA
GATGACGAGTCAAAAATGTTTGGAACATTGCTGAACATGTGTAAGTCTATAAGGCGAAAATTTGACTCTGTTATGAGCGATTACGATTCCATTGTCACAGAAAAATCCGAAGG
CGAACAAAATGATGGTAAAGTAACTGTAGCTGAGTTCACATCTTTGTGTGAAGCGGAAGAAATGCTCTTAGCATTATGCTATAACTATTATAATCTGACGTTATACAGTTTCTTT
GAATTTGGGACTAATATTGAATACATGGAACATCTGTTGCTTCTTCTTGAAGAACAGCTTGCTCTCGACGAATACTATGGTTTTGAAAAGGTCTTGAATGTAGCTGTTGCAAATG
CTAAAAAAATGGGTTTCCACCGTTGGGAGTTTTACGTCGGCTATGAAGAGTCGACTGCTGAAAAGAGGCGGCTACTATGGTGGAAGTTATACAATTATGAAAAAGCCAGTACT
ATGAAGAAGGGTTTTTTTTCTGTGATTGATGATGCTACTGTCAACTGTTTATTACCTAAGATTTTTAGAAACTTTGGCTATCTGGATAGGGTGGAGTTTCTAGAAAATATTCAAA
AGCCAATGGATCTTAGTGTGTTTTCCGATGTTCCAATTTCTGTCCTTTGTAAATACGGTGAGTTGGCCCTTACAATAGTTACCAGTGAGTTTCATGAAAAATTTTTATATGCTGAT
AGATACACTTCTATTCGAAATTCCGCGAAACCGCCGACATTAAAAAACCAATTAATTAAGGAAATTGTGGATGGTATAGCTTATACAGAGACATCATATGAGGCAATCAGAAA
GCAAACTGCAAAACTATGGGATATTGCATTAGGTAAGGTGACCAAAGATAAAATCAATAAAGAAGATACAGCAGCAGCTAGCAAATTTACTTTGAGTTATGAATATCACAGAT
TCAGGCTAATCAATATGGCAGACAATTTAATTGCTAGACTAATGGTGAAACCAAAATCAGATTGGCTAATATCAGTCATGAAGGGGCATCTTAACAGACTATATGAGCACTGG

AAAGTAATGAATGAAATTATCCTAAGTATGGACAACGATTATTCAATTGCAACAACGTTCGAATATTATGCACCATCATGTCTGTGTTTAGCTACGCAGACTTTCCTTATTGTGA
GGAATATGGAAATGGATGATGTCAAGATGATGGTTGCAGTATATAAAAGATTTCTTAACCTAGGAATGTTTTTGCAGAGTGCCAAAGTATGCAGCCTTGCCGATAGTCATACAT
TCAGAGATTTTTCTAGATCTTTTTCCTTTATTACGATAATTTCAAGATTGATGATAATCGAATTTATGCAAATTAAAGAATTGACGAAGGTAGAGTTTATTGAGAAGTTTTCTGAA
GTATGCCCTGACCTTGCAGATCTACCTCCGATGCTTCTAGATCCAAACTCTTGCTTATATTTTTCATTGTTACAGCAGATTAAGAAATCTGGTTTTACGTTGTCATTCAAAAAAATT
CTTGAAGACGCTAGAATGATGGACTTCAATTACGACCGCAATTTGGACTCAGAGGCCATTAAAAAGTGCAATGGTGAATTTAGCAAGTCAATGCCTTCCTGTACCAATGTCTCA
GATACCACCACCGCTGTTTCTGACAATAGTGCTAAGAAGAAAGCTTCAATGGGGTCGGCGAGGGTAAATTCAACTGATACACTAACTGCATCTCCCTTATCGGGCTTAAGGAAT
CAAACGCAGTTGGATTCTAAAGACAGTGTTCCATCTCTCGAGGCTTATACACCAATTGATTCTGTCTCTGACGTGCCCACTGGGGAGATCAACGTTCCATTCCCTCCTGTTTATA
ATCAAAATGGATTGGATCAGCAAACCACTTATAATTTGGGAACTTTAGATGAGTTTGTTAACAAGGGAGATTTGAATGAACTCTATAATAGCCTATGGGGTGACCTATTTTCTG
ATGTTTACTTGTGA

>35

ATGCAAACATTAGAAACTACATCAAAATCAAATCCAGGGGAAGTCAAAGCACAGAAGCCTAGTACAAGAAGAACAAAAGTTGGAAAAGCTTGTGATAGCTGTAGAAGGAGG
AAAATAAAATGTAATGGGCTAAAACCTTGTCCATCTTGTACAATCTATGGYTGTGAATGTACATATACTGATGCAAAATCGACAAAAAATCTCAAATCAAATGATGCAGGTAAA
YCAAAACCAACAGGGAGAGTATCAAAGAATAAAGAAACTACTAGAATCGACAAAGATATTAGGAAATCAGAGCAGCAGTATGTCCCTATTAATGCTAATATTCATGTTGGTCC
CAGGTTCCCCTCCGAGAATATATTGAATGGATATYCACAATGTGGAGCACCACAGAACAATGTTGTGGGTAATCCACTAGCGGTTAATCCTCAATGCCATAGAGGTCTTTCTGA
AACTCCTATGTCCTCAACATTCAAAGAATCTAACTTGAGAGATGATCGGCTACTACAGTCATCAGATACAGATGATATGAGGAATGGTGACTCGGAAGAAAGGGACTTGAAAG
GGAGTGACAGCGAGAATGTCAAAAGTAAAGACAATAAAAGTGATCCTTTGATTATATACAAAGATGATACACATATTGAAAGCACGGTTAATAAACTAACACAGGCAGTTAAT
GAACTCAAATCACTTCAAAATGCACCYAGTTCGATAAAATCATCCATTRACGCCATTGAGTTACAACTTAGAAACATTTTAGACAAYTGGAAACCAGAGGTAGATTTCGAGAAA
GCAAAGATTAATGAAAGTGCCACCACTAAGTCACTTGAAACAAACTTGCTGAGGAATAAATACACTAATCACGTTCATCTAACAAGATTTAGGATATGGATAGATTATAAAAAT
GCGAACAAAAACAATCATTTTATGGGAGAGTGTGGATTTAGTCTTGCAGAATCTTTTTTTGCTTCTAATCAGCCATTGGTCGATGAATTGTTTGGGTTGTATTCCCAGGTAGAGG
CCTTTTCTTTGCAAGGTCTTGGTTACTGTGTTCACCTTTATGAGCCATATATGAAAACTGAGGAAGCGATAAAACTGATGAAAGAGACCTTATATATTATACTACGGTTTATTGA
TATATGTGTTCACCATATCAATGAAGAGTCGATATCGATTGCCAACCCGTTAGAAACATATTTACGAAAAAAACATCTAATGCCTATGACTCCTACACCAAGGTCGTCCTATGGA
AGTCCACAAAGTGCTAGTACAAAGAGCTTGGTAAGTAAGATAATAGAGAGAATACCGCAACCGTTTATTGAGAGTGTAACTAATGTGTCGAGTCTTCAACTATTAGATCTTCGA
GATGACGAGTCAAAAATGTTTGGAACATTGCTGAACATGTGTAAGTCTATAAGGCGAAAATTTGACTCTGTTATGAGCGATTACGATTCCATTGTCACAGAAAAATCCGAAGG
CGAACAAAATGATGGTAAAGTAACTGTAGCTGAGTTCACATCTTTGTGTGAAGCGGAAGAAATGCTCTTAGCATTATGCTATAACTATTATAATCTGACGTTATACAGTTTCTTT
GAATTTGGGACTAATATTGAATACATGGAACATCTGTTGCTTCTTCTTGAAGAACAGCTTGCTCTCGACGAATACTATGGTTTTGAAAAGGTCTTGAATGTAGCTGTTGCAAATG
CTAAAAAAATGGGTTTCCACCGTTGGGAGTTTTACGTCGGYTATGAAGAGTCGACTGCTGAAAAGAGGCGGCTACTATGGTGGAAGTTATACAATTATGAAAAAGCCAGTACT
ATGAAGAAGGGTTTTTTTTCTGTGATTGATGATGCTACTGTCAACTGTTTATTACCTAAGATTTTTAGAAACTTTGGCTATCTGGATAGGGTGGAGTTTCTAGAAAATATTCAAA
AGCCAATGGATCTTAGTGTGTTTTCCGATGTTCCAATTTCTGTCCTTTGTAAATACGGTGAGTTGGCCCTTACAATAGTTACCAGTGAGTTTCATGAAAAATTTTTATATGCTGAT
AGATACACTTCTATTCGAAATTCCGCGAAACCGCCGACATTAAAAAACCAATTAATTAAGGAAATTGTGGATGGTATAGCTTATACAGAGACATCATATGAGGCAATCAGAAA
GCAAACTGCAAAACTATGGGATATTGCATTAGGTAAGGTGACCAAAGATAAAATCAATAAAGAAGATACAGCAGCAGCTAGCAAATTTACTTTGAGTTATGAATATCACAGAT

TCAGGCTAATCAATATGGCAGACAATTTAATTGCTAGACTTATGGTGAAACCAAAATCAGATTGGCTAATATCAGTCATGAAGGGGCATCTTAACAGACTATATGAGCACTGGA
AAGTAATGAATGAAATTATCCTAAGTATGGACAACGATTATTCAATTGCAACAACGTTCGAATATTATGCACCATCATGTCTGTGTTTAGCTACGCAGACTTTCCTTATTGTGAG
GAATATGGAAATGGATGATGTCAAGATGATGGTTGCAGTATATAAAAGATTTCTTAACCTAGGAATGTTTCTGCAGAGTGCCAAAGTATGCAGCCTTGCCGATAGTCATACATT
CAGAGATTTTTCTAGATCTTTTTCCTTTATTACGATAATTTCAAGATTGATGATAATCGAATTTATGCAAATTAAAGAATTGACGAAGGTAGAGTTTATTGAGAAGTTTTCTGAAG
TATGCCCTGACCTTGCAGATCTACCTCCGATGCTTCTAGATCCAAACTCTTGCTTATATTTTTCATTGTTACAGCAGATTAAGAAATCTGGTTTTACGTTGTCATTCAAAAAAATTC
TTGAAGACGCTAGAATGATGGACTTCAATTACGACCGCAATTTGGACTCAGAGGCCATTAAAAAGTGCAATGGTGAATTTAGCAAGTCAATGCCTTCCTGTACCAATGTCTCAG
ATACCACCACCGCTGTTTCTGACAACAGTGCTAAGAAGAAAGCTTCAATGGGGTCGGCGAGGGTAAATTCAACTGATACACTAACTGCATCTCCCTTATCGGGCTTAAGGAATC
AAACGCAGTTGGATTCTAAAGACAGTGTTCCATCTCTCGAGGCTTATACACCAATTGATTCTGTCTCTGACGTRCCCACTGGGGAGATCAACGTTCCATTCCCTCCTGTTTATAAT
CAAAATGGATTGGATCAGCAAACCACTTATAATTTGGGAACTTTAGATGAGTTTGTTAACAAGGGAGATTTGAATGAACTCTATAATAGCCTATGGGGTGACCTATTTTCTGAT
GTTTACTTGTGA

>36

ATGCAAACATTAGAAACTACATCAAAATCAAATCCAGGGGAAGTCAAAGCACAGAAGCCTAGTACAAGAAGAACAAAAGTTGGAAAAGCTTGTGATAGCTGTAGAAGGAGG
AAAATAAAATGTAATGGGCTAAAACCTTGTCCATCTTGTACAATCTATGGTTGTGAATGTACATATACTGATGCAAAATCGACAAAAAATCTCAAATCAAATGATGCAGGTAAA
CCAAAACCAACAGGGAGAGTATCAAAGAATAAAGAAACTACTAGAATCGACAAAGATATTAGGAAATCAGAGCAGCAGTATGTACCTATTAATGCTAATATTCATGTTGGTCC
CAGGTTCCCCTCCGAGAATATATTGAATGGATATCCACAATGTGGAGCACCACAGAACAATGTTGTGGGTAATCCACTAGCGGTTAATCCTCAATGCCATAGAGGTCTTTCTGA
AACTCCTATGTCCTCAACATTCAAAGAATCTAACTTAAGAGATGATCGGCTACTACAGTCATCAGATACAGATGATATGAGGAATGGTGACTCGGAAGAAAGGGACTTGAAAG
GGAGTGACAGCGAGAATGTCAAAAGTAAAGACAATAAAAGTGATCCTTTGATTATATACAAAGATGATACACATATTGAAAGCACGGTTAATAAACTAACACAGGCAGTTAAT
GAACTCAAATCACTTCAAAATGCACCTAGTTCGATAAAATCATCCATTGACGCCATTGAGTTACAACTTAGAAACATTTTAGACAATTGGAAACCAGAGGTAGATTTCGAGAAA
GCAAAGATTAATGAAAGTGCCACCACTAAGTCACTTGAAACAAACTTGCTGAGGAATAAATACACTAATCACGTTCATCTAACAAGATTTAGGATATGGATAGATTATAAAAAT
GCGAACAAAAACAATCATTTTATGGGAGAGTGTGGATTTAGTCTTGCAGAATCTTTTTTTGCTTCTAATCAGCCATTGGTCGATGAATTGTTTGGGTTGTATTCCCAGGTAGAGG
CCTTTTCTTTGCAAGGTCTTGGTTACTGTGTTCACCTTTATGAGCCATATATGAAAACTGAGGAAGCGATAAAACTGATGAAAGAGACCTTATATATTATACTACGGTTTATTGA
TATATGTGTTCACCATATCAATGAAGAGTCGATATCGATTGCCAACCCGTTAGAAACATATTTACGAAAAAAACATCTAATGCCTATGACTCCTACACCAAGGTCGTCCTATGGA
AGTCCACAAAGTGCTAGTACAAAGAGCTTGGTAAGTAAGATAATAGAGAGAATACCGCAACCGTTTATTGAGAGTGTAACTAATGTGTCGAGTCTTCAACTATTAGATCTTCGA
GATGACGAGTCAAAAATGTTTGGAACATTGCTGAACATGTGTAAGTCTATAAGGCGAAAATTTGACTCTGTTATGAGCGATTACGATTCCATTGTCACAGAAAAATCCGAAGG
CGAACAAAATGATGGTAAAGTAACTGTAGCTGAGTTCACATCTTTGTGTGAAGCGGAAGAAATGCTCTTAGCATTATGCTATAACTATTATAATCTGACGTTATACAGTTTCTTT
GAATTTGGGACTAATATTGAATACATGGAACATCTGTTGCTTCTTCTTGAAGAACAGCTTGCTCTCGACGAATACTATGGTTTTGAAAAGGTCTTGAATGTAGCTGTTGCAAATG
CTAAAAAAATGGGTTTCCACCGTTGGGAGTTTTACGTCGGTTATGAAGAGTCGACTGCTGAAAAGAGGCGGCTACTATGGTGGAAGTTATACAATTATGAAAAAGCCAGTACT
ATGAAGAAGGGTTTTTTTTCTGTGATTGATGATGCTACTGTCAACTGTTTATTACCTAAGATTTTTAGAAACTTTGGCTATCTGGATAGGGTGGAGTTTCTAGAAAATATTCAAA
AGCCAATGGATCTTAGTGTGTTTTCCGATGTTCCAATTTCTGTCCTTTGTAAATACGGTGAGTTGGCCCTTACAATAGTTACCAGTGAGTTTCATGAAAAATTTTTATATGCTGAT
AGATACACTTCTATTCGAAATTCCGCGAAACCGCCGACATTAAAAAACCAATTAATTAAGGAAATTGTGGATGGTATAGCTTATACAGAGACATCATATGAGGCAATCAGAAA

GCAAACTGCAAAACTATGGGATATTGCATTAGGTAAGGTGACCAAAGATAAAATCAATAAAGAAGATACAGCAGCAGCTAGCAAATTTACTTTGAGTTATGAATATCACAGAT
TCAGGCTAATCAATATGGCAGACAATTTAATTGCTAGACTTATGGTGAAACCAAAATCAGATTGGCTAATATCAGTCATGAAGGGGCATCTTAACAGACTATATGAGCACTGGA
AAGTAATGAATGAAATTATCCTAAGTATGGACAACGATTATTCAATTGCAACAACGTTCGAATATTATGCACCATCATGTCTGTGTTTAGCTACGCAGACTTTCCTTATTGTGAG
GAATATGGAAATGGATGATGTCAAGATGATGGTTGCAGTATATAAAAGATTTCTTAACCTAGGAATGTTTCTGCAGAGTGCCAAAGTATGCAGCCTTGCCGATAGTCATACATT
CAGAGATTTTTCTAGATCTTTTTCCTTTATTACGATAATTTCAAGATTGATGATAATCGAATTTATGCAAATTAAAGAATTGACGAAGGTAGAGTTTATTGAGAAGTTTTCTGAAG
TATGCCCTGACCTTGCAGATCTACCTCCGATGCTTCTAGATCCAAACTCTTGCTTATATTTTTCATTGTTACAGCAGATTAAGAAATCTGGTTTTACGTTGTCATTCAAAAAAATTC
TTGAAGACGCTAGAATGATGGACTTCAATTACGACCGCAATTTGGACTCAGAGGCCATTAAAAAGTGCAATGGTGAATTTAGCAAGTCAATGCCTTCCTGTACCAATGTCTCAG
ATACCACCACCGCTGTTTCTGACAACAGTGCTAAGAAGAAAGCTTCAATGGGGTCGGCGAGGGTAAATTCAACTGATACACTAACTGCATCTCCCTTATCGGGCTTAAGGAATC
AAACGCAGTTGGATTCTAAAGACAGTGTTCCATCTCTCGAGGCTTATACACCAATTGATTCTGTCTCTGACGTACCCACTGGGGAGATCAACGTTCCATTCCCTCCTGTTTATAAT
CAAAATGGATTGGATCAGCAAACCACTTATAATTTGGGAACTTTAGATGAGTTTGTTAACAAGGGAGATTTGAATGAACTCTATAATAGCCTATGGGGTGACCTATTTTCTGAT
GTTTACTTGTGA

>37

ATGCAAACATTAGAAACTACATCAAAATCAAATCCAGGGGAAGTCAAAGCACAGAAGCCTAGTACAAGAAGAACAAAAGTTGGAAAAGCTTGTGATAGCTGTAGAAGGAGG
AAAATAAAATGTAATGGGCTAAAACCTTGTCCATCTTGTACAATCTATGGTTGTGAATGTACATATACTGATGCAAAATCGACAAAAAATCTCAAATCAAATGATGCAGGTAAA
CCAAAACCAACAGGGAGAGTATCAAAGAATAAAGAAACTACTAGAATCGACAAAGATATTAGGAAATCAGAGCAGCAGTATGTCCCTATTAATGCTAATATTCATGTTGGTCC
CAGGTTCCCCTCCGAGAATATATTGAATGGATATCCACAATGTGGAGCACCACAGAACAATGTTGTGGGTAATCCACTAGCGGTTAATCCTCAATGCCATAGAGGTCTTTCTGA
AACTCCTATGTCCTCAACATTCAAAGAATCTAACTTAAGAGATGATCGGCTACTACAGTCATCAGATACAGATGATATGAGGAATGGTGACTCGGAAGAAAGGGACTTGAAAG
GGAGTGACAGCGAGAATGTCAAAAGTAAAGACAATAAAAGTGATCCTTTGATTATATACAAAGATGATACACATATTGAAAGCACGGTTAATAAACTAACACAGGCAGTTAAT
GAACTCAAATCACTTCAAAATGCACCTAGTTCGATAAAATCATCCATTGACGCCATTGAGTTACAACTTAGAAACATTTTAGACAATTGGAAACCAGAGGTAGATTTCGAGAAA
GCAAAGATTAATGAAAGTGCCACCACTAAGTCACTTGAAACAAACTTGCTGAGGAATAAATACACTAATCACGTTCATCTAACAAGATTTAGGATATGGATAGATTATAAAAAT
GCGAACAAAAACAATCATTTTATGGGAGAGTGTGGATTTAGTCTTGCAGAATCTTTTTTTGCTTCTAATCAGCCATTGGTCGATGAATTGTTTGGGTTGTATTCCCAGGTAGAGG
CCTTTTCTTTGCAAGGTCTTGGTTACTGTGTTCACCTTTATGAGCCATATATGAAAACTGAGGAAGCGATAAAACTGATGAAAGAGACCTTATATATTATACTACGGTTTATTGA
TATATGTGTTCACCATATCAATGAAGAGTCGATATCGATTGCCAACCCGTTAGAAACATATTTACGAAAAAAACATCTAATGCCTATGACTCCTACACCAAGGTCGTCCTATGGA
AGTCCACAAAGTGCTAGTACAAAGAGCTTGGTAAGTAAGATAATAGAGAGAATACCGCAACCGTTTATTGAGAGTGTAACTAATGTGTCGAGTCTTCAACTATTAGATCTTCGA
GATGACGAGTCAAAAATGTTTGGAACATTGCTGAACATGTGTAAGTCTATAAGGCGAAAATTTGACTCTGTTATGAGCGATTACGATTCCATTGTCACAGAAAAATCCGAAGG
CGAACAAAATGATGGTAAAGTAACTGTAGCTGAGTTCACATCTTTGTGTGAAGCGGAAGAAATGCTCTTAGCATTATGCTATAACTATTATAATCTGACGTTATACAGTTTCTTT
GAATTTGGGACTAATATTGAATACATGGAACATCTGTTGCTTCTTCTTGAAGAACAGCTTGCTCTCGACGAATACTATGGTTTTGAAAAGGTCTTGAATGTAGCTGTTGCAAATG
CTAAAAAAATGGGTTTCCACCGTTGGGAGTTTTACGTCGGTTATGAAGAGTCGACTGCTGAAAAGAGGCGGCTACTATGGTGGAAGTTATACAATTATGAAAAAGCCAGTACT
ATGAAGAAGGGTTTTTTTTCTGTGATTGATGATGCTACTGTCAACTGTTTATTACCTAAGATTTTTAGAAACTTTGGCTATCTGGATAGGGTGGAGTTTCTAGAAAATATTCAAA
AGCCAATGGATCTTAGTGTGTTTTCCGATGTTCCAATTTCTGTCCTTTGTAAATACGGTGAGTTGGCCCTTACAATAGTTACCAGTGAGTTTCATGAAAAATTTTTATATGCTGAT

AGATACACTTCTATTCGAAATTCCGCGAAACCGCCGACATTAAAAAACCAATTAATTAAGGAAATTGTGGATGGTATAGCTTATACAGAGACATCATATGAGGCAATCAGAAA
GCAAACTGCAAAACTATGGGATATTGCATTAGGTAAGGTGACCAAAGATAAAATCAATAAAGAAGATACAGCAGCAGCTAGCAAATTTACTTTGAGTTATGAATATCACAGAT
TCAGGCTAATCAATATGGCAGACAATTTAATTGCTAGACTTATGGTGAAACCAAAATCAGATTGGCTAATATCAGTCATGAAGGGGCATCTTAACAGACTATATGAGCACTGGA
AAGTAATGAATGAAATTATCCTAAGTATGGACAACGATTATTCAATTGCAACAACGTTCGAATATTATGCACCATCATGTCTGTGTTTAGCTACGCAGACTTTCCTTATTGTGAG
GAATATGGAAATGGATGATGTCAAGATGATGGTTGCAGTATATAAAAGATTTCTTAACCTAGGAATGTTTCTGCAGAGTGCCAAAGTATGCAGCCTTGCCGATAGTCATACATT
CAGAGATTTTTCTAGATCTTTTTCCTTTATTACGATAATTTCAAGATTGATGATAATCGAATTTATGCAAATTAAAGAATTGACGAAGGTAGAGTTTATTGAGAAGTTTTCTGAAG
TATGCCCTGACCTTGCAGATCTACCTCCGATGCTTCTAGATCCAAACTCTTGCTTATATTTTTCATTGTTACAGCAGATTAAGAAATCTGGTTTTACGTTGTCATTCAAAAAAATTC
TTGAAGACGCTAGAATGATGGACTTCAATTACGACCGCAATTTGGACTCAGAGGCCATTAAAAAGTGCAATGGTGAATTTAGCAAGTCAATGCCTTCCTGTACCAATGTCTCAG
ATACCACCACCGCTGTTTCTGACAACAGTGCTAAGAAGAAAGCTTCAATGGGGTCGGCGAGGGTAAATTCAACTGATACACTAACTGCATCTCCCTTATCGGGCTTAAGGAATC
AAACGCAGTTGGATTCTAAAGACAGTGTTCCATCTCTCGAGGCTTATACACCAATTGATTCTGTCTCTGACGTACCCACTGGGGAGATCAACGTTCCATTCCCTCCTGTTTATAAT
CAAAATGGATTGGATCAGCAAACCACTTATAATTTGGGAACTTTAGATGAGTTTGTTAACAAGGGAGATTTGAATGAACTCTATAATAGCCTATGGGGTGACCTATTTTCTGAT
GTTTACTTGTGA

>38

ATGCAAACATTAGAAACTACATCAAAATCAAATCCAGGGGAAGTCAAAGCACAGAAGCCTAGTACAAGAAGAACAAAAGTTGGAAAAGCTTGTGATAGCTGTAGAAGGAGG
AAAATAAAATGTAATGGGCTAAAACCTTGTCCATCTTGTACAATCTATGGTTGTGAATGTACATATACTGATGCAAAATCGACAAAAAATCTCAAATCAAATGATGCAGGTAAA
CCAAAACCAACAGGGAGAGTATCAAAGAATAAAGAAACTACTAGAATCGACAAAGATATTAGGAAATCAGAGCAGCAGTATGTCCCTATTAATGCTAATATTCATGTTGGTCC
CAGGTTCCCCTCCGAGAATATATTGAATGGATATCCACAATGTGGAGCACCACAGAACAATGTTGTGGGTAATCCACTAGCGGTTAATCCTCAATGCCATAGAGGTCTTTCTGA
AACTCCTATGTCCTCAACATTCAAAGAATCTAACTTAAGAGATGATCGGCTACTACAGTCATCAGATACAGATGATATGAGGAATGGTGACTCGGAAGAAAGGGACTTGAAAG
GGAGTGACAGCGAGAATGTCAAAAGTAAAGACAATAAAAGTGATCCTTTGATTATATACAAAGATGATACACATATTGAAAGCACGGTTAATAAACTAACACAGGCAGTTAAT
GAACTCAAATCACTTCAAAATGCACCTAGTTCGATAAAATCATCCATTGACGCCATTGAGTTACAACTTAGAAACATTTTAGACAATTGGAAACCAGAGGTAGATTTCGAGAAA
GCAAAGATTAATGAAAGTGCCACCACTAAGTCACTTGAAACAAACTTGCTGAGGAATAAATACACTAATCACGTTCATCTAACAAGATTTAGGATATGGATAGATTATAAAAAT
GCGAACAAAAACAATCATTTTATGGGAGAGTGTGGATTTAGTCTTGCAGAATCTTTTTTTGCTTCTAATCAGCCATTGGTCGATGAATTGTTTGGGTTGTATTCCCAGGTAGAGG
CCTTTTCTTTGCAAGGTCTTGGTTACTGTGTTCACCTTTATGAGCCATATATGAAAACTGAGGAAGCGATAAAACTGATGAAAGAGACCTTATATATTATACTACGGTTTATTGA
TATATGTGTTCACCATATCAATGAAGAGTCGATATCGATTGCCAACCCGTTAGAAACATATTTACGAAAAAAACATCTAATGCCTATGACTCCTACACCAAGGTCGTCCTATGGA
AGTCCACAAAGTGCTAGTACAAAGAGCTTGGTAAGTAAGATAATAGAGAGAATACCGCAACCGTTTATTGAGAGTGTAACTAATGTGTCGAGTCTTCAACTATTAGATCTTCGA
GATGACGAGTCAAAAATGTTTGGAACATTGCTGAACATGTGTAAGTCTATAAGGCGAAAATTTGACTCTGTTATGAGCGATTACGATTCCATTGTCACAGAAAAATCCGAAGG
CGAACAAAATGATGGTAAAGTAACTGTAGCTGAGTTCACATCTTTGTGTGAAGCGGAAGAAATGCTCTTAGCATTATGCTATAACTATTATAATCTGACGTTATACAGTTTCTTT
GAATTTGGGACTAATATTGAATACATGGAACATCTGTTGCTTCTTCTTGAAGAACAGCTTGCTCTCGACGAATACTATGGTTTTGAAAAGGTCTTGAATGTAGCTGTTGCAAATG
CTAAAAAAATGGGTTTCCACCGTTGGGAGTTTTACGTCGGTTATGAAGAGTCGACTGCTGAAAAGAGGCGGCTACTATGGTGGAAGTTATACAATTATGAAAAAGCCAGTACT
ATGAAGAAGGGTTTTTTTTCTGTGATTGATGATGCTACTGTCAACTGTTTATTACCTAAGATTTTTAGAAACTTTGGCTATCTGGATAGGGTGGAGTTTCTAGAAAATATTCAAA

AGCCAATGGATCTTAGTGTGTTTTCCGATGTTCCAATTTCTGTCCTTTGTAAATACGGTGAGTTGGCCCTTACAATAGTTACCAGTGAGTTTCATGAAAAATTTTTATATGCTGAT
AGATACACTTCTATTCGAAATTCCGCGAAACCGCCGACATTAAAAAACCAATTAATTAAGGAAATTGTGGATGGTATAGCTTATACAGAGACATCATATGAGGCAATCAGAAA
GCAAACTGCAAAACTATGGGATATTGCATTAGGTAAGGTGACCAAAGATAAAATCAATAAAGAAGATACAGCAGCAGCTAGCAAATTTACTTTGAGTTATGAATATCACAGAT
TCAGGCTAATCAATATGGCAGACAATTTAATTGCTAGACTTATGGTGAAACCAAAATCAGATTGGCTAATATCAGTCATGAAGGGGCATCTTAACAGACTATATGAGCACTGGA
AAGTAATGAATGAAATTATCCTAAGTATGGACAACGATTATTCAATTGCAACAACGTTCGAATATTATGCACCATCATGTCTGTGTTTAGCTACGCAGACTTTCCTTATTGTGAG
GAATATGGAAATGGATGATGTCAAGATGATGGTTGCAGTATATAAAAGATTTCTTAACCTAGGAATGTTTCTGCAGAGTGCCAAAGTATGCAGCCTTGCCGATAGTCATACATT
CAGAGATTTTTCTAGATCTTTTTCCTTTATTACGATAATTTCAAGATTGATGATAATCGAATTTATGCAAATTAAAGAATTGACGAAGGTAGAGTTTATTGAGAAGTTTTCTGAAG
TATGCCCTGACCTTGCAGATCTACCTCCGATGCTTCTAGATCCAAACTCTYGCTTATATTTTTCATTGTTACAGCAGATTAAGAAATCTGGTTTTACGTTGTCATTCAAAAAAATTC
TTGAAGACGCTAGAATGATGGACTTCAATTACGACCGCAATTTGGACTCAGAGGCCATTAAAAAGTGCAATGGTGAATTTAGCAAGTCAATGCCTTCCTGTACCAATGTCTCAG
ATACCACCACCGCTGTTTCTGACAACAGTGCTAAGAAGAAAGCTTCAATGGGGTCGGCGAGGGTAAATTCAACTGATACACTAACTGCATCTCCCTTATCGGGCTTAAGGAATC
AAACGCAGTTGGATTCTAAAGACAGTGTTCCATCTCTCGAGGCTTATACACCAATTGATTCTGTCTCTGACGTACCCACTGGGGAGATCAACGTTCCATTCCCTCCTGTTTATAAT
CAAAATGGATTGGATCAGCAAACCACTTATAATTTGGGAACTTTAGATGAGTTTGTTAACAAGGGAGATTTGAATGAACTCTATAATAGCCTATGGGGTGACCTATTTTCTGAT
GTTTACTTGTGA

>39

ATGCAAACATTAGAAACTACATCAAAATCAAATCCAGGGGAAGTCAAAGCACAGAAGCCTAGTACAAGAAGAACAAAAGTTGGAAAAGCTTGTGATAGCTGTAGAAGGAGG
AAAATAAAATGTAATGGGCTAAAACCTTGTCCATCTTGTACAATCTATGGTTGTGAATGTACATATACTGATGCAAAATCGACAAAAAATCTCAAATCAAATGATGCAGGTAAA
CCAAAACCAACAGGGAGAGTATCAAAGAATAAAGAAACTACTAGAATCGACAAAGATATTAGGAAATCAGAGCAGCAGTATGTCCCTATTAATGCTAATATTCATGTTGGTCC
CAGGTTCCCCTCCGAGAATATATTGAATGGATATCCACAATGTGGAGCACCACAGAACAATGTTGTGGGTAATCCACTAGCGGTTAATCCTCAATGCCATAGAGGTCTTTCTGA
AACTCCTATGTCCTCAACATTCAAAGAATCTAACTTAAGAGATGATCGGCTACTACAGTCATCAGATACAGATGATATGAGGAATGGTGACTCGGAAGAAAGGGACTTGAAAG
GGAGTGACAGCGAGAATGTCAAAAGTAAAGACAATAAAAGTGATCCTTTGATTATATACAAAGATGATACACATATTGAAAGCACGGTTAATAAACTAACACAGGCAGTTAAT
GAACTCAAATCACTTCAAAATGCACCTAGTTCGATAAAATCATCCATTGACGCCATTGAGTTACAACTTAGAAACATTTTAGACAATTGGAAACCAGAGGTAGATTTCGAGAAA
GCAAAGATTAATGAAAGTGCCACCACTAAGTCACTTGAAACAAACTTGCTGAGGAATAAATACACTAATCACGTTCATCTAACAAGATTTAGGATATGGATAGATTATAAAAAT
GCGAACAAAAACAATCATTTTATGGGAGAGTGTGGATTTAGTCTTGCAGAATCTTTTTTTGCTTCTAATCAGCCATTGGTCGATGAATTGTTTGGGTTGTATTCCCAGGTAGAGG
CCTTTTCTTTGCAAGGTCTTGGTTACTGTGTTCACCTTTATGAGCCATATATGAAAACTGAGGAAGCGATAAAACTGATGAAAGAGACCTTATATATTATACTACGGTTTATTGA
TATATGTGTTCACCATATCAATGAAGAGTCGATATCGATTGCCAACCCGTTAGAAACATATTTACGAAAAAAACATCTAATGCCTATGACTCCTACACCAAGGTCGTCCTATGGA
AGTCCACAAAGTGCTAGTACAAAGAGCTTGGTAAGTAAGATAATAGAGAGAATACCGCAACCGTTTATTGAGAGTGTAACTAATGTGTCGAGTCTTCAACTATTAGATCTTCGA
GATGACGAGTCAAAAATGTTTGGAACATTGCTGAACATGTGTAAGTCTATAAGGCGAAAATTTGACTCTGTTATGAGCGATTACGATTCCATTGTCACAGAAAAATCCGAAGG
CGAACAAAATGATGGTAAAGTAACTGTAGCTGAGTTCACATCTTTGTGTGAAGCGGAAGAAATGCTCTTAGCATTATGCTATAACTATTATAATCTGACGTTATACAGTTTCTTT
GAATTTGGGACTAATATTGAATACATGGAACATCTGTTGCTTCTTCTTGAAGAACAGCTTGCTCTCGACGAATACTATGGTTTTGAAAAGGTCTTGAATGTAGCTGTTGCAAATG
CTAAAAAAATGGGTTTCCACCGTTGGGAGTTTTACGTCGGTTATGAAGAGTCGACTGCTGAAAAGAGGCGGCTACTATGGTGGAAGTTATACAATTATGAAAAAGCCAGTACT

ATGAAGAAGGGTTTTTTTTCTGTGATTGATGATGCTACTGTCAACTGTTTATTACCTAAGATTTTTAGAAACTTTGGCTATCTGGATAGGGTGGAGTTTCTAGAAAATATTCAAA
AGCCAATGGATCTTAGTGTGTTTTCCGATGTTCCAATTTCTGTCCTTTGTAAATACGGTGAGTTGGCCCTTACAATAGTTACCAGTGAGTTTCATGAAAAATTTTTATATGCTGAT
AGATACACTTCTATTCGAAATTCCGCGAAACCGCCGACATTAAAAAACCAATTAATTAAGGAAATTGTGGATGGTATAGCTTATACAGAGACATCATATGAGGCAATCAGAAA
GCAAACTGCAAAACTATGGGATATTGCATTAGGTAAGGTGACCAAAGATAAAATCAATAAAGAAGATACAGCAGCAGCTAGCAAATTTACTTTGAGTTATGAATATCACAGAT
TCAGGCTAATCAATATGGCAGACAATTTAATTGCTAGACTTATGGTGAAACCAAAATCAGATTGGCTAATATCAGTCATGAAGGGGCATCTTAACAGACTATATGAGCACTGGA
AAGTAATGAATGAAATTATCCTAAGTATGGACAACGATTATTCAATTGCAACAACGTTCGAATATTATGCACCATCATGTCTGTGTTTAGCTACGCAGACTTTCCTTATTGTGAG
GAATATGGAAATGGATGATGTCAAGATGATGGTTGCAGTATATAAAAGATTTCTTAACCTAGGAATGTTTCTGCAGAGTGCCAAAGTATGCAGCCTTGCCGATAGTCATACATT
CAGAGATTTTTCTAGATCTTTTTCCTTTATTACGATAATTTCAAGATTGATGATAATCGAATTTATGCAAATTAAAGAATTGACGAAGGTAGAGTTTATTGAGAAGTTTTCTGAAG
TATGCCCTGACCTTGCAGATCTACCTCCGATGCTTCTAGATCCAAACTCTYGCTTATATTTTTCATTGTTACAGCAGATTAAGAAATCTGGTTTTACGTTGTCATTCAAAAAAATTC
TTGAAGACGCTAGAATGATGGACTTCAATTACGACCGCAATTTGGACTCAGAGGCCATTAAAAAGTGCAATGGTGAATTTAGCAAGTCAATGCCTTCCTGTACCAATGTCTCAG
ATACCACCACCGCTGTTTCTGACAACAGTGCTAAGAAGAAAGCTTCAATGGGGTCGGCGAGGGTAAATTCAACTGATACACTAACTGCATCTCCCTTATCGGGCTTAAGGAATC
AAACGCAGTTGGATTCTAAAGACAGTGTTCCATCTCTCGAGGCTTATACACCAATTGATTCTGTCTCTGACGTACCCACTGGGGAGATCAACGTTCCATTCCCTCCTGTTTATAAT
CAAAATGGATTGGATCAGCAAACCACTTATAATTTGGGAACTTTAGATGAGTTTGTTAACAAGGGAGATTTGAATGAACTCTATAATAGCCTATGGGGTGACCTATTTTCTGAT
GTTTACTTGTGA

>40

ATGCAAACATTAGAAACTACATCAAAATCAAATCCAGGGGAAGTCAAAGCACAGAAGCCTAGTACAAGAAGAACAAAAGTTGGAAAAGCTTGTGATAGCTGTAGAAGGAGG
AAAATAAAATGTAATGGGCTAAAACCTTGTCCATCTTGTACAATCTATGGTTGTGAATGTACATATACTGATGCAAAATCGACAAAAAATCTCAAATCAAATGATGCAGGTAAA
CCAAAACCAACAGGGAGAGTATCAAAGAATAAAGAAACTACTAGAATCGACAAAGATATTAGGAAATCAGAGCAGCAGTATGTCCCTATTAATGCTAATATTCATGTTGGTCC
CAGGTTCCCCTCCGAGAATATATTGAATGGATATCCACAATGTGGAGCACCACAGAACAATGTTGTGGGTAATCCACTAGCGGTTAATCCTCAATGCCATAGAGGTCTTTCTGA
AACTCCTATGTCCTCAACATTCAAAGAATCTAACTTAAGAGATGATCGGCTACTACAGTCATCAGATACAGATGATATGAGGAATGGTGACTCGGAAGAAAGGGACTTGAAAG
GGAGTGACAGCGAGAATGTCAAAAGTAAAGACAATAAAAGTGATCCTTTGATTATATACAAAGATGATACACATATTGAAAGCACGGTTAATAAACTAACACAGGCAGTTAAT
GAACTCAAATCACTTCAAAATGCACCTAGTTCGATAAAATCATCCATTGACGCCATTGAGTTACAACTTAGAAACATTTTAGACAATTGGAAACCAGAGGTAGATTTCGAGAAA
GCAAAGATTAATGAAAGTGCCACCACTAAGTCACTTGAAACAAACTTGCTGAGGAATAAATACACTAATCACGTTCATCTAACAAGATTTAGGATATGGATAGATTATAAAAAT
GCGAACAAAAACAATCATTTTATGGGAGAGTGTGGATTTAGTCTTGCAGAATCTTTTTTTGCTTCTAATCAGCCATTGGTCGATGAATTGTTTGGGTTGTATTCCCAGGTAGAGG
CCTTTTCTTTGCAAGGTCTTGGTTACTGTGTTCACCTTTATGAGCCATATATGAAAACTGAGGAAGCGATAAAACTGATGAAAGAGACCTTATATATTATACTACGGTTTATTGA
TATATGTGTTCACCATATCAATGAAGAGTCGATATCGATTGCCAACCCGTTAGAAACATATTTACGAAAAAAACATCTAATGCCTATGACTCCTACACCAAGGTCGTCCTATGGA
AGTCCACAAAGTGCTAGTACAAAGAGCTTGGTAAGTAAGATAATAGAGAGAATACCGCAACCGTTTATTGAGAGTGTAACTAATGTGTCGAGTCTTCAACTATTAGATCTTCGA
GATGACGAGTCAAAAATGTTTGGAACATTGCTGAACATGTGTAAGTCTATAAGGCGAAAATTTGACTCTGTTATGAGCGATTACGATTCCATTGTCACAGAAAAATCCGAAGG
CGAACAAAATGATGGTAAAGTAACTGTAGCTGAGTTCACATCTTTGTGTGAAGCGGAAGAAATGCTCTTAGCATTATGCTATAACTATTATAATCTGACGTTATACAGTTTCTTT
GAATTTGGGACTAATATTGAATACATGGAACATCTGTTGCTTCTTCTTGAAGAACAGCTTGCTCTCGACGAATACTATGGTTTTGAAAAGGTCTTGAATGTAGCTGTTGCAAATG

CTAAAAAAATGGGTTTCCACCGTTGGGAGTTTTACGTCGGTTATGAAGAGTCGACTGCTGAAAAGAGGCGGCTACTATGGTGGAAGTTATACAATTATGAAAAAGCCAGTACT
ATGAAGAAGGGTTTTTTTTCTGTGATTGATGATGCTACTGTCAACTGTTTATTACCTAAGATTTTTAGAAACTTTGGCTATCTGGATAGGGTGGAGTTTCTAGAAAATATTCAAA
AGCCAATGGATCTTAGTGTGTTTTCCGATGTTCCAATTTCTGTCCTTTGTAAATACGGTGAGTTGGCCCTTACAATAGTTACCAGTGAGTTTCATGAAAAATTTTTATATGCTGAT
AGATACACTTCTATTCGAAATTCCGCGAAACCGCCGACATTAAAAAACCAATTAATTAAGGAAATTGTGGATGGTATAGCTTATACAGAGACATCATATGAGGCAATCAGAAA
GCAAACTGCAAAACTATGGGATATTGCATTAGGTAAGGTGACCAAAGATAAAATCAATAAAGAAGATACAGCAGCAGCTAGCAAATTTACTTTGAGTTATGAATATCACAGAT
TCAGGCTAATCAATATGGCAGACAATTTAATTGCTAGACTTATGGTGAAACCAAAATCAGATTGGCTAATATCAGTCATGAAGGGGCATCTTAACAGACTATATGAGCACTGGA
AAGTAATGAATGAAATTATCCTAAGTATGGACAACGATTATTCAATTGCAACAACGTTCGAATATTATGCACCATCATGTCTGTGTTTAGCTACGCAGACTTTCCTTATTGTGAG
GAATATGGAAATGGATGATGTCAAGATGATGGTTGCAGTATATAAAAGATTTCTTAACCTAGGAATGTTTCTGCAGAGTGCCAAAGTATGCAGCCTTGCCGATAGTCATACATT
CAGAGATTTTTCTAGATCTTTTTCCTTTATTACGATAATTTCAAGATTGATGATAATCGAATTTATGCAAATTAAAGAATTGACGAAGGTAGAGTTTATTGAGAAGTTTTCTGAAG
TATGCCCTGACCTTGCAGATCTACCTCCGATGCTTCTAGATCCAAACTCTYGCTTATATTTTTCATTGTTACAGCAGATTAAGAAATCTGGTTTTACGTTGTCATTCAAAAAAATTC
TTGAAGACGCTAGAATGATGGACTTCAATTACGACCGCAATTTGGACTCAGAGGCCATTAAAAAGTGCAATGGTGAATTTAGCAAGTCAATGCCTTCCTGTACCAATGTCTCAG
ATACCACCACCGCTGTTTCTGACAACAGTGCTAAGAAGAAAGCTTCAATGGGGTCGGCGAGGGTAAATTCAACTGATACACTAACTGCATCTCCCTTATCGGGCTTAAGGAATC
AAACGCAGTTGGATTCTAAAGACAGTGTTCCATCTCTCGAGGCTTATACACCAATTGATTCTGTCTCTGACGTACCCACTGGGGAGATCAACGTTCCATTCCCTCCTGTTTATAAT
CAAAATGGATTGGATCAGCAAACCACTTATAATTTGGGAACTTTAGATGAGTTTGTTAACAAGGGAGATTTGAATGAACTCTATAATAGCCTATGGGGTGACCTATTTTCTGAT
GTTTACTTGTGA

>41

ATGCAAACATTAGAAACTACATCAAAATCAAATCCAGGGGAAGTCAAAGCACAGAAGCCTAGTACAAGAAGAACAAAAGTTGGAAAAGCTTGTGATAGCTGTAGAAGGAGG
AAAATAAAATGTAATGGGCTAAAACCTTGTCCATCTTGTACAATCTATGGTTGTGAATGTACATATACTGATGCAAAATCGACAAAAAATCTCAAATCAAATGATGCAGGTAAA
CCAAAACCAACAGGGAGAGTATCAAAGAATAAAGAAACTACTAGAATCGACAAAGATATTAGGAAATCAGAGCAGCAGTATGTCCCTATTAATGCTAATATTCATGTTGGTCC
CAGGTTCCCCTCCGAGAATATATTGAATGGATATYCACAATGTGGAGCACCACAGAACAATGTTGTGGGTAATCCACTAGCGGTTAATCCTCAATGCCATAGAGGTCTTTCTGA
AACTCCTATGTCCTCAACATTCAAAGAATCTAACTTGAGAGATGATCGGCTACTACAGTCATCAGATACAGATGATATGAGGAATGGTGACTCGGAAGAAAGGGACTTGAAAG
GGAGTGACAGCGAGAATGTCAAAAGTAAAGACAATAAAAGTGATCCTTTGATTATATACAAAGATGATACACATATTGAAAGCACGGTTAATAAACTAACACAGGCAGTTAAT
GAACTCAAATCACTTCAAAATGCACCTAGTTCGATAAAATCATCCATTGACGCCATTGAGTTACAACTTAGAAACATTTTAGACAATTGGAAACCAGAGGTAGATTTCGAGAAA
GCAAAGATTAATGAAAGTGCCACCACTAAGTCACTTGAAACAAACTTGCTGAGGAATAAATACACTAATCACGTTCATCTAACAAGATTTAGGATATGGATAGATTATAAAAAT
GCGAACAAAAACAATCATTTTATGGGAGAGTGTGGATTTAGTCTTGCAGAATCTTTTTTTGCTTCTAATCAGCCATTGGTCGATGAATTGTTTGGGTTGTATTCCCAGGTAGAGG
CCTTTTCTTTGCAAGGTCTTGGTTACTGTGTTCACCTTTATGAGCCATATATGAAAACTGAGGAAGCGATAAAACTGATGAAAGAGACCTTATATATTATACTACGGTTTATTGA
TATATGTGTTCACCATATCAATGAAGAGTCGATATCGATTGCCAACCCGTTAGAAACATATTTACGAAAAAAACATCTAATGCCTATGACTCCTACACCAAGGTCGTCCTATGGA
AGTCCACAAAGTGCTAGTACAAAGAGCTTGGTAAGTAAGATAATAGAGAGAATACCGCAACCGTTTATTGAGAGTGTAACTAATGTGTCGAGTCTTCAACTATTAGATCTTCGA
GATGACGAGTCAAAAATGTTTGGAACATTGCTGAACATGTGTAAGTCTATAAGGCGAAAATTTGACTCTGTTATGAGCGATTACGATTCCATTGTCACAGAAAAATCCGAAGG
CGAACAAAATGATGGTAAAGTAACTGTAGCTGAGTTCACATCTTTGTGTGAAGCGGAAGAAATGCTCTTAGCATTATGCTATAACTATTATAATCTGACGTTATACAGTTTCTTT

GAATTTGGGACTAATATTGAATACATGGAACATCTGTTGCTTCTTCTTGAAGAACAGCTTGCTCTCGACGAATACTATGGTTTTGAAAAGGTCTTGAATGTAGCTGTTGCAAATG
CTAAAAAAATGGGTTTCCACCGTTGGGAGTTTTACGTCGGTTATGAAGAGTCGACTGCTGAAAAGAGGCGGCTACTATGGTGGAAGTTATACAATTATGAAAAAGCCAGTACT
ATGAAGAAGGGTTTTTTTTCTGTGATTGATGATGCTACTGTCAACTGTTTATTACCTAAGATTTTTAGAAACTTTGGCTATCTGGATAGGGTGGAGTTTCTAGAAAATATTCAAA
AGCCAATGGATCTTAGTGTGTTTTCCGATGTTCCAATTTCTGTCCTTTGTAAATACGGTGAGTTGGCCCTTACAATAGTTACCAGTGAGTTTCATGAAAAATTTTTATATGCTGAT
AGATACACTTCTATTCGAAATTCCGCGAAACCGCCGACATTAAAAAACCAATTAATTAAGGAAATTGTGGATGGTATAGCTTATACAGAGACATCATATGAGGCAATCAGAAA
GCAAACTGCAAAACTATGGGATATTGCATTAGGTAAGGTGACCAAAGATAAAATCAATAAAGAAGATACAGCAGCAGCTAGCAAATTTACTTTGAGTTATGAATATCACAGAT
TCAGGCTAATCAATATGGCAGACAATTTAATTGCTAGACTTATGGTGAAACCAAAATCAGATTGGCTAATATCAGTCATGAAGGGGCATCTTAACAGACTATATGAGCACTGGA
AAGTAATGAATGAAATTATCCTAAGTATGGACAACGATTATTCAATTGCAACAACGTTCGAATATTATGCACCATCATGTCTGTGTTTAGCTACGCAGACTTTCCTTATTGTGAG
GAATATGGAAATGGATGATGTCAAGATGATGGTTGCAGTATATAAAAGATTTCTTAACCTAGGAATGTTTCTGCAGAGTGCCAAAGTATGCAGCCTTGCCGATAGTCATACATT
CAGAGATTTTTCTAGATCTTTTTCCTTTATTACGATAATTTCAAGATTGATGATAATCGAATTTATGCAAATTAAAGAATTGACGAAGGTAGAGTTTATTGAGAAGTTTTCTGAAG
TATGCCCTGACCTTGCAGATCTACCTCCGATGCTTCTAGATCCAAACTCTTGCTTATATTTTTCATTGTTACAGCAGATTAAGAAATCTGGTTTTACGTTGTCATTCAAAAAAATTC
TTGAAGACGCTAGAATGATGGACTTCAATTACGACCGCAATTTGGACTCAGAGGCCATTAAAAAGTGCAATGGTGAATTTAGCAAGTCAATGCCTTCCTGTACCAATGTCTCAG
ATACCACCACCGCTGTTTCTGACAACAGTGCTAAGAAGAAAGCTTCAATGGGGTCGGCGAGGGTAAATTCAACTGATACACTAACTGCATCTCCCTTATCGGGCTTAAGGAATC
AAACGCAGTTGGATTCTAAAGACAGTGTTCCATCTCTCGAGGCTTATACACCAATTGATTCTGTCTCTGACGTACCCACTGGGGAGATCAACGTTCCATTCCCTCCTGTTTATAAT
CAAAATGGATTGGATCAGCAAACCACTTATAATTTGGGAACTTTAGATGAGTTTGTTAACAAGGGAGATTTGAATGAACTCTATAATAGCCTATGGGGTGACCTATTTTCTGAT
GTTTACTTGTGA

>42

ATGCAAACATTAGAAACTACATCAAAATCAAATCCAGGGGAAGTCAAAGCACAGAAGCCTAGTACAAGAAGAACAAAAGTTGGAAAAGCTTGTGATAGCTGTAGAAGGAGG
AAAATAAAATGTAATGGGCTAAAACCTTGTCCATCTTGTACAATCTATGGTTGTGAATGTACATATACTGATGCAAAATCGACAAAAAATCTCAAATCAAATGATGCAGGTAAA
CCAAAACCAACAGGGAGAGTATCAAAGAATAAAGAAACTACTAGAATCGACAAAGATATTAGGAAATCAGAGCAGCAGTATGTCCCTATTAATGCTAATATTCATGTTGGTCC
CAGGTTCCCCTCCGAGAATATATTGAATGGATATCCACAATGTGGAGCACCACAGAACAATGTTGTGGGTAATCCACTAGCGGTTAATCCTCAATGCCATAGAGGTCTTTCTGA
AACTCCTATGTCCTCAACATTCAAAGAATCTAACTTAAGAGATGATCGGCTACTACAGTCATCAGATACAGATGATATGAGGAATGGTGACTCGGAAGAAAGGGACTTGAAAG
GGAGTGACAGCGAGAATGTCAAAAGTAAAGACAATAAAAGTGATCCTTTGATTATATACAAAGATGATACACATATTGAAAGCACGGTTAATAAACTAACACAGGCAGTTAAT
GAACTCAAATCACTTCAAAATGCACCTAGTTCGATAAAATCATCCATTGACGCCATTGAGTTACAACTTAGAAACATTTTAGACAATTGGAAACCAGAGGTAGATTTCGAGAAA
GCAAAGATTAATGAAAGTGCCACCACTAAGTCACTTGAAACAAACTTGCTGAGGAATAAATACACTAATCACGTTCATCTAACAAGATTTAGGATATGGATAGATTATAAAAAT
GCGAACAAAAACAATCATTTTATGGGAGAGTGTGGATTTAGTCTTGCAGAATCTTTTTTTGCTTCTAATCAGCCATTGGTCGATGAATTGTTTGGGTTGTATTCCCAGGTAGAGG
CCTTTTCTTTGCAAGGTCTTGGTTACTGTGTTCACCTTTATGAGCCATATATGAAAACTGAGGAAGCGATAAAACTGATGAAAGAGACCTTATATATTATACTACGGTTTATTGA
TATATGTGTTCACCATATCAATGAAGAGTCGATATCGATTGCCAACCCGTTAGAAACATATTTACGAAAAAAACATCTAATGCCTATGACTCCTACACCAAGGTCGTCCTATGGA
AGTCCACAAAGTGCTAGTACAAAGAGCTTGGTAAGTAAGATAATAGAGAGAATACCGCAACCGTTTATTGAGAGTGTAACTAATGTGTCGAGTCTTCAACTATTAGATCTTCGA
GATGACGAGTCAAAAATGTTTGGAACATTGCTGAACATGTGTAAGTCTATAAGGCGAAAATTTGACTCTGTTATGAGCGATTACGATTCCATTGTCACAGAAAAATCCGAAGG

CGAACAAAATGATGGTAAAGTAACTGTAGCTGAGTTCACATCTTTGTGTGAAGCGGAAGAAATGCTCTTAGCATTATGCTATAACTATTATAATCTGACGTTATACAGTTTCTTT
GAATTTGGGACTAATATTGAATACATGGAACATCTGTTGCTTCTTCTTGAAGAACAGCTTGCTCTCGACGAATACTATGGTTTTGAAAAGGTCTTGAATGTAGCTGTTGCAAATG
CTAAAAAAATGGGTTTCCACCGTTGGGAGTTTTACGTCGGTTATGAAGAGTCGACTGCTGAAAAGAGGCGGCTACTATGGTGGAAGTTATACAATTATGAAAAAGCCAGTACT
ATGAAGAAGGGTTTTTTTTCTGTGATTGATGATGCTACTGTCAACTGTTTATTACCTAAGATTTTTAGAAACTTTGGCTATCTGGATAGGGTGGAGTTTCTAGAAAATATTCAAA
AGCCAATGGATCTTAGTGTGTTTTCCGATGTTCCAATTTCTGTCCTTTGTAAATACGGTGAGTTGGCCCTTACAATAGTTACCAGTGAGTTTCATGAAAAATTTTTATATGCTGAT
AGATACACTTCTATTCGAAATTCCGCGAAACCGCCGACATTAAAAAACCAATTAATTAAGGAAATTGTGGATGGTATAGCTTATACAGAGACATCATATGAGGCAATCAGAAA
GCAAACTGCAAAACTATGGGATATTGCATTAGGTAAGGTGACCAAAGATAAAATCAATAAAGAAGATACAGCAGCAGCTAGCAAATTTACTTTGAGTTATGAATATCACAGAT
TCAGGCTAATCAATATGGCAGACAATTTAATTGCTAGACTTATGGTGAAACCAAAATCAGATTGGCTAATATCAGTCATGAAGGGGCATCTTAACAGACTATATGAGCACTGGA
AAGTAATGAATGAAATTATCCTAAGTATGGACAACGATTATTCAATTGCAACAACGTTCGAATATTATGCACCATCATGTCTGTGTTTAGCTACGCAGACTTTCCTTATTGTGAG
GAATATGGAAATGGATGATGTCAAGATGATGGTTGCAGTATATAAAAGATTTCTTAACCTAGGAATGTTTCTGCAGAGTGCCAAAGTATGCAGCCTTGCCGATAGTCATACATT
CAGAGATTTTTCTAGATCTTTTTCCTTTATTACGATAATTTCAAGATTGATGATAATCGAATTTATGCAAATTAAAGAATTGACGAAGGTAGAGTTTATTGAGAAGTTTTCTGAAG
TATGCCCTGACCTTGCAGATCTACCTCCGATGCTTCTAGATCCAAACTCTTGCTTATATTTTTCATTGTTACAGCAGATTAAGAAATCTGGTTTTACGTTGTCATTCAAAAAAAATTC
TTGAAGACGCTAGAATGATGGACTTCAATTACGACCGCAATTTGGACTCAGAGGCCATTAAAAAGTGCAATGGTGAATTTAGCAAGTCAATGCCTTCCTGTACCAATGTCTCAG
ATACCACCACCGCTGTTTCTGACAACAGTGCTAAGAAGAAAGCTTCAATGGGGTCGGCGAGGGTAAATTCAACTGATACACTAACTGCATCTCCCTTATCGGGCTTAAGGAATC
AAACGCAGTTGGATTCTAAAGACAGTGTTCCATCTCTCGAGGCTTATACACCAATTGATTCTGTCTCTGACGTACCCACTGGGGAGATCAACGTTCCATTCCCTCCTGTTTATAAT
CAAAATGGATTGGATCAGCAAACCACTTATAATTTGGGAACTTTAGATGAGTTTGTTAACAAGGGAGATTTGAATGAACTCTATAATAGCCTATGGGGTGACCTATTTTCTGAT
GTTTACTTGTGA

>43

ATGCAAACATTAGAAACTACATCAAAATCAAATCCAGGGGAAGTCAAAGCACAGAAGCCTAGTACAAGAAGAACAAAAGTTGGAAAAGCTTGTGATAGCTGTAGAAGGAGG
AAAATAAAATGTAATGGGCTAAAACCTTGTCCATCTTGTACAATCTATGGCTGTGAATGTACATATACTGATGCAAAATCGACAAAAAATCTCAAATCAAATGATGCAGGTAAA
TCAAAACCAACAGGGAGAGTATCAAAGAATAAAGAAACTACTAGAATCGACAAAGATATTAGGAAATCAGAGCAGCAGTATGTCCCTATTAATGCTAATATTCATGTTGGTCC
CAGGTTCCCCTCCGAGAATATATTGAATGGATATCCACAATGTGGAGCACCACAGAACAATGTTGTGGGTAATCCACTAGCGGTTAATACTCAATGCCATAGAGGTCTTTCTGA
AACTCCTATGTCCTCAACATTCAAAGAATCTAACTTAAGAGATGATCGGCTACTACAGTCATCAGATACAGATGATATGAGGAATGGTGACTCGGAAGAAAGGGACTTGAAAG
GGAGTGACAGCGAGAATGTCAAAAGTAAAGACAATAAAAGTGATCCTTTGATTATATACAAAGATGATACACATATTGAAAGCACGGTTAATAAACTAACACAGGCAGTTAAT
GAACTCAAATCACTTCAAAATGCACCCAGTTCGATAAAATCATCCATTAACGCCATTGAGTTACAACTTAGAAACATTTTAGACAACTGGAAACCAGAGGTAGATTTCGAGAAA
GCAAAGATTAATGAAAGTGCCACCACTAAGTCACTTGAAACAAACTTGCTGAGGAATAAATACACTAATCACGTTCATTTAACAAGATTTAGGATATGGATAGATTATAAAAAT
GCGAACAAAAACAATCATTTTATGGGAGAGTGTGGATTTAGTCTTGCAGAATCTTTTTTTGCTTCTAATCAGCCATTGGTCGATGAATTGTTTGGGTTGTATTCCCAGGTAGAGG
CCTTTTCTTTGCAAGGTCTTGGTTACTGTGTTCACCTTTATGAGCCATATATGAAAACTGAGGAAGCGATAAAACTGATGAAAGAGACCTTATATATTATACTACGGTTTATTGA
TATATGTGTTCACCATATCAATGAAGAGTCGATATCGATTGCCAACCCGTTAGAAACATATTTACGAAAAAAACATCTAATGCCTATGACTCCTACACCAAGGTCGTCCTATGGA
AGTCCACAAAGTGCTAGTACAAAGAGCTTGGTAAGTAAGATAATAGAGAGAATACCGCAACCGTTTATTGAGAGTGTAACTAATGTGTCGAGTCTTCAACTATTAGATCTTCGA

GATGACGAGTCAAAAATGTTTGGAACATTGCTGAACATGTGTAAGTCTATAAGGCGAAAATTTGACTCTGTTATGAGCGATTACGATTCCATTGTCACAGAAAAATCCGAAGG
CGAACAAAATGATGGTAAAGTAACTGTAGCTGAGTTCACATCTTTGTGTGAAGCGGAAGAAATGCTCTTAGCATTATGCTATAACTATTATAATCTGACGTTATACAGTTTCTTT
GAATTTGGGACTAATATTGAATACATGGAACATCTGTTGCTTCTTCTTGAAGAACAGCTTGCTCTCGACGAATACTATGGTTTTGAAAAGGTCTTGAATGTAGCTGTTGCAAATG
CTAAAAAAATGGGTTTCCACCGTTGGGAGTTTTACGTCGGCTATGAAGAGTCGACTGCTGAAAAGAGGCGGCTACTATGGTGGAAGTTATACAATTATGAAAAAGCCAGTACT
ATGAAGAAGGGTTTTTTTTCTGTGATTGATGATGCTACTGTCAACTGTTTATTACCTAAGATTTTTAGAAACTTTGGCTATCTGGATAGGGTGGAGTTTCTAGAAAATATTCAAA
AGCCAATGGATCTTAGTGTGTTTTCCGATGTTCCAATTTCTGTCCTTTGTAAATACGGTGAGTTGGCCCTTACAATAGTTACCAGTGAGTTTCATGAAAAATTTTTATATGCTGAT
AGATACACTTCTATTCGAAATTCCGCGAAACCGCCGACATTAAAAAACCAATTAATTAAGGAAATTGTGGATGGTATAGCTTATACAGAGACATCATATGAGGCAATCAGAAA
GCAAACTGCAAAACTATGGGATATTGCATTAGGTAAGGTGACCAAAGATAAAATCAATAAAGAAGATACAGCAGCAGCTAGCAAATTTACTTTGAGTTATGAATATCACAGAT
TCAGGCTAATCAATATGGCAGACAATTTAATTGCTAGACTAATGGTGAAACCAAAATCAGATTGGCTAATATCAGTCATGAAGGGGCATCTTAACAGACTATATGAGCACTGG
AAAGTAATGAATGAAATTATCCTAAGTATGGACAACGATTATTCAATTGCAACAACGTTCGAATATTATGCACCATCATGTCTGTGTTTAGCTACGCAGACTTTCCTTATTGTGA
GGAATATGGAAATGGATGATGTCAAGATGATGGTTGCAGTATATAAAAGATTTCTTAACCTAGGAATGTTTTTGCAGAGTGCCAAAGTATGCAGCCTTGCCGATAGTCATACAT
TCAGAGATTTTTCTAGATCTTTTTCCTTTATTACGATAATTTCAAGATTGATGATAATCGAATTTATGCAAATTAAAGAATTGACGAAGGTAGAGTTTATTGAGAAGTTTTCTGAA
GTATGCCCTGACCTTGCAGATCTACCTCCGATGCTTCTAGATCCAAACTCTTGCTTATATTTTTCATTGTTACAGCAGATTAAGAAATCTGGTTTTACGTTGTCATTCAAAAAAATT
CTTGAAGACGCTAGAATGATGGACTTCAATTACGACCGCAATTTGGACTCAGAGGCCATTAAAAAGTGCAATGGTGAATTTAGCAAGTCAATGCCTTCCTGTACCAATGTCTCA
GATACCACCACCGCTGTTTCTGACAATAGTGCTAAGAAGAAAGCTTCAATGGGGTCGGCGAGGGTAAATTCAACTGATACACTAACTGCATCTCCCTTATCGGGCTTAAGGAAT
CAAACGCAGTTGGATTCTAAAGACAGTGTTCCATCTCTCGAGGCTTATACACCAATTGATTCTGTCTCTGACGTGCCCACTGGGGAGATCAACGTTCCATTCCCTCCTGTTTATA
ATCAAAATGGATTGGATCAGCAAACCACTTATAATTTGGGAACTTTAGATGAGTTTGTTAACAAGGGAGATTTGAATGAACTCTATAATAGCCTATGGGGTGACCTATTTTCTG
ATGTTTACTTGTGA

>44

ATGCAAACATTAGAAACTACATCAAAATCAAATCCAGGGGAAGTCAAAGCACAGAAGCCTAGTACAAGAAGAACAAAAGTTGGAAAAGCTTGTGATAGCTGTAGAAGGAGG
AAAATAAAATGTAATGGGCTAAAACCTTGTCCATCTTGTACAATCTATGGTTGTGAATGTACATATACTGATGCAAAATCGACAAAAAATCTCAAATCAAATGATGCAGGTAAA
CCAAAACCAACAGGGAGAGTATCAAAGAATAAAGAAACTACTAGAATCGACAAAGATATTAGGAAATCAGAGCAGCAGTATGTCCCTATTAATGCTAATATTCATGTTGGTCC
CAGGTTCCCCTCCGAGAATATATTGAATGGATATCCACAATGTGGAGCACCACAGAACAATGTTGTGGGTAATCCACTAGCGGTTAATCCTCAATGCCATAGAGGTCTTTCTGA
AACTCCTATGTCCTCAACATTCAAAGAATCTAACTTAAGAGATGATCGGCTACTACAGTCATCAGATACAGATGATATGAGGAATGGTGACTCGGAAGAAAGGGACTTGAAAG
GGAGTGACAGCGAGAATGTCAAAAGTAAAGACAATAAAAGTGATCCTTTGATTATATACAAAGATGATACACATATTGAAAGCACGGTTAATAAACTAACACAGGCAGTTAAT
GAACTCAAATCACTTCAAAATGCACCTAGTTCGATAAAATCATCCATTGACGCCATTGAGTTACAACTTAGAAACATTTTAGACAATTGGAAACCAGAGGTAGATTTCGAGAAA
GCAAAGATTAATGAAAGTGCCACCACTAAGTCACTTGAAACAAACTTGCTGAGGAATAAATACACTAATCACGTTCATCTAACAAGATTTAGGATATGGATAGATTATAAAAAT
GCGAACAAAAACAATCATTTTATGGGAGAGTGTGGATTTAGTCTTGCAGAATCTTTTTTTGCTTCTAATCAGCCATTGGTCGATGAATTGTTTGGGTTGTATTCCCAGGTAGAGG
CCTTTTCTTTGCAAGGTCTTGGTTACTGTGTTCACCTTTATGAGCCATATATGAAAACTGAGGAAGCGATAAAACTGATGAAAGAGACCTTATATATTATACTACGGTTTATTGA
TATATGTGTTCACCATATCAATGAAGAGTCGATATCGATTGCCAACCCGTTAGAAACATATTTACGAAAAAAACATCTAATGCCTATGACTCCTACACCAAGGTCGTCCTATGGA

AGTCCACAAAGTGCTAGTACAAAGAGCTTGGTAAGTAAGATAATAGAGAGAATACCGCAACCGTTTATTGAGAGTGTAACTAATGTGTCGAGTCTTCAACTATTAGATCTTCGA
GATGACGAGTCAAAAATGTTTGGAACATTGCTGAACATGTGTAAGTCTATAAGGCGAAAATTTGACTCTGTTATGAGCGATTACGATTCCATTGTCACAGAAAAATCCGAAGG
CGAACAAAATGATGGTAAAGTAACTGTAGCTGAGTTCACATCTTTGTGTGAAGCGGAAGAAATGCTCTTAGCATTATGCTATAACTATTATAATCTGACGTTATACAGTTTCTTT
GAATTTGGGACTAATATTGAATACATGGAACATCTGTTGCTTCTTCTTGAAGAACAGCTTGCTCTCGACGAATACTATGGTTTTGAAAAGGTCTTGAATGTAGCTGTTGCAAATG
CTAAAAAAATGGGTTTCCACCGTTGGGAGTTTTACGTCGGTTATGAAGAGTCGACTGCTGAAAAGAGGCGGCTACTATGGTGGAAGTTATACAATTATGAAAAAGCCAGTACT
ATGAAGAAGGGTTTTTTTTCTGTGATTGATGATGCTACTGTCAACTGTTTATTACCTAAGATTTTTAGAAACTTTGGCTATCTGGATAGGGTGGAGTTTCTAGAAAATATTCAAA
AGCCAATGGATCTTAGTGTGTTTTCCGATGTTCCAATTTCTGTCCTTTGTAAATACGGTGAGTTGGCCCTTACAATAGTTACCAGTGAGTTTCATGAAAAATTTTTATATGCTGAT
AGATACACTTCTATTCGAAATTCCGCGAAACCGCCGACATTAAAAAACCAATTAATTAAGGAAATTGTGGATGGTATAGCTTATACAGAGACATCATATGAGGCAATCAGAAA
GCAAACTGCAAAACTATGGGATATTGCATTAGGTAAGGTGACCAAAGATAAAATCAATAAAGAAGATACAGCAGCAGCTAGCAAATTTACTTTGAGTTATGAATATCACAGAT
TCAGGCTAATCAATATGGCAGACAATTTAATTGCTAGACTTATGGTGAAACCAAAATCAGATTGGCTAATATCAGTCATGAAGGGGCATCTTAACAGACTATATGAGCACTGGA
AAGTAATGAATGAAATTATCCTAAGTATGGACAACGATTATTCAATTGCAACAACGTTCGAATATTATGCACCATCATGTCTGTGTTTAGCTACGCAGACTTTCCTTATTGTGAG
GAATATGGAAATGGATGATGTCAAGATGATGGTTGCAGTATATAAAAGATTTCTTAACCTAGGAATGTTTCTGCAGAGTGCCAAAGTATGCAGCCTTGCCGATAGTCATACATT
CAGAGATTTTTCTAGATCTTTTTCCTTTATTACGATAATTTCAAGATTGATGATAATCGAATTTATGCAAATTAAAGAATTGACGAAGGTAGAGTTTATTGAGAAGTTTTCTGAAG
TATGCCCTGACCTTGCAGATCTACCTCCGATGCTTCTAGATCCAAACTCTYGCTTATATTTTTCATTGTTACAGCAGATTAAGAAATCTGGTTTTACGTTGTCATTCAAAAAAAATTC
TTGAAGACGCTAGAATGATGGACTTCAATTACGACCGCAATTTGGACTCAGAGGCCATTAAAAAGTGCAATGGTGAATTTAGCAAGTCAATGCCTTCCTGTACCAATGTCTCAG
ATACCACCACCGCTGTTTCTGACAACAGTGCTAAGAAGAAAGCTTCAATGGGGTCGGCGAGGGTAAATTCAACTGATACACTAACTGCATCTCCCTTATCGGGCTTAAGGAATC
AAACGCAGTTGGATTCTAAAGACAGTGTTCCATCTCTCGAGGCTTATACACCAATTGATTCTGTCTCTGACGTACCCACTGGGGAGATCAACGTTCCATTCCCTCCTGTTTATAAT
CAAAATGGATTGGATCAGCAAACCACTTATAATTTGGGAACTTTAGATGAGTTTGTTAACAAGGGAGATTTGAATGAACTCTATAATAGCCTATGGGGTGACCTATTTTCTGAT
GTTTACTTGTGA

>45

ATGCAAACATTAGAAACTACATCAAAATCAAATCCAGGGGAAGTCAAAGCACAGAAGCCTAGTACAAGAAGAACAAAAGTTGGAAAAGCTTGTGATAGCTGTAGAAGGAGG
AAAATAAAATGTAATGGGCTAAAACCTTGTCCATCTTGTACAATCTATGGTTGTGAATGTACATATACTGATGCAAAATCGACAAAAAATCTCAAATCAAATGATGCAGGTAAA
CCAAAACCAACAGGGAGAGTATCAAAGAATAAAGAAACTACTAGAATCGACAAAGATATTAGGAAATCAGAGCAGCAGTATGTCCCTATTAATGCTAATATTCATGTTGGTCC
CAGGTTCCCCTCCGAGAATATATTGAATGGATATCCACAATGTGGAGCACCACAGAACAATGTTGTGGGTAATCCACTAGCGGTTAATCCTCAATGCCATAGAGGTCTTTCTGA
AACTCCTATGTCCTCAACATTCAAAGAATCTAACTTAAGAGATGATCGGCTACTACAGTCATCAGATACAGATGATATGAGGAATGGTGACTCGGAAGAAAGGGACTTGAAAG
GGAGTGACAGCGAGAATGTCAAAAGTAAAGACAATAAAAGTGATCCTTTGATTATATACAAAGATGATACACATATTGAAAGCACGGTTAATAAACTAACACAGGCAGTTAAT
GAACTCAAATCACTTCAAAATGCACCTAGTTCGATAAAATCATCCATTGACGCCATTGAGTTACAACTTAGAAACATTTTAGACAATTGGAAACCAGAGGTAGATTTCGAGAAA
GCAAAGATTAATGAAAGTGCCACCACTAAGTCACTTGAAACAAACTTGCTGAGGAATAAATACACTAATCACGTTCATCTAACAAGATTTAGGATATGGATAGATTATAAAAAT
GCGAACAAAAACAATCATTTTATGGGAGAGTGTGGATTTAGTCTTGCAGAATCTTTTTTTGCTTCTAATCAGCCATTGGTCGATGAATTGTTTGGGTTGTATTCCCAGGTAGAGG
CCTTTTCTTTGCAAGGTCTTGGTTACTGTGTTCACCTTTATGAGCCATATATGAAAACTGAGGAAGCGATAAAACTGATGAAAGAGACCTTATATATTATACTACGGTTTATTGA

TATATGTGTTCACCATATCAATGAAGAGTCGATATCGATTGCCAACCCGTTAGAAACATATTTACGAAAAAAACATCTAATGCCTATGACTCCTACACCAAGGTCGTCCTATGGA
AGTCCACAAAGTGCTAGTACAAAGAGCTTGGTAAGTAAGATAATAGAGAGAATACCGCAACCGTTTATTGAGAGTGTAACTAATGTGTCGAGTCTTCAACTATTAGATCTTCGA
GATGACGAGTCAAAAATGTTTGGAACATTGCTGAACATGTGTAAGTCTATAAGGCGAAAATTTGACTCTGTTATGAGCGATTACGATTCCATTGTCACAGAAAAATCCGAAGG
CGAACAAAATGATGGTAAAGTAACTGTAGCTGAGTTCACATCTTTGTGTGAAGCGGAAGAAATGCTCTTAGCATTATGCTATAACTATTATAATCTGACGTTATACAGTTTCTTT
GAATTTGGGACTAATATTGAATACATGGAACATCTGTTGCTTCTTCTTGAAGAACAGCTTGCTCTCGACGAATACTATGGTTTTGAAAAGGTCTTGAATGTAGCTGTTGCAAATG
CTAAAAAAATGGGTTTCCACCGTTGGGAGTTTTACGTCGGTTATGAAGAGTCGACTGCTGAAAAGAGGCGGCTACTATGGTGGAAGTTATACAATTATGAAAAAGCCAGTACT
ATGAAGAAGGGTTTTTTTTCTGTGATTGATGATGCTACTGTCAACTGTTTATTACCTAAGATTTTTAGAAACTTTGGCTATCTGGATAGGGTGGAGTTTCTAGAAAATATTCAAA
AGCCAATGGATCTTAGTGTGTTTTCCGATGTTCCAATTTCTGTCCTTTGTAAATACGGTGAGTTGGCCCTTACAATAGTTACCAGTGAGTTTCATGAAAAATTTTTATATGCTGAT
AGATACACTTCTATTCGAAATTCCGCGAAACCGCCGACATTAAAAAACCAATTAATTAAGGAAATTGTGGATGGTATAGCTTATACAGAGACATCATATGAGGCAATCAGAAA
GCAAACTGCAAAACTATGGGATATTGCATTAGGTAAGGTGACCAAAGATAAAATCAATAAAGAAGATACAGCAGCAGCTAGCAAATTTACTTTGAGTTATGAATATCACAGAT
TCAGGCTAATCAATATGGCAGACAATTTAATTGCTAGACTTATGGTGAAACCAAAATCAGATTGGCTAATATCAGTCATGAAGGGGCATCTTAACAGACTATATGAGCACTGGA
AAGTAATGAATGAAATTATCCTAAGTATGGACAACGATTATTCAATTGCAACAACGTTCGAATATTATGCACCATCATGTCTGTGTTTAGCTACGCAGACTTTCCTTATTGTGAG
GAATATGGAAATGGATGATGTCAAGATGATGGTTGCAGTATATAAAAGATTTCTTAACCTAGGAATGTTTCTGCAGAGTGCCAAAGTATGCAGCCTTGCCGATAGTCATACATT
CAGAGATTTTTCTAGATCTTTTTCCTTTATTACGATAATTTCAAGATTGATGATAATCGAATTTATGCAAATTAAAGAATTGACGAAGGTAGAGTTTATTGAGAAGTTTTCTGAAG
TATGCCCTGACCTTGCAGATCTACCTCCGATGCTTCTAGATCCAAACTCTYGCTTATATTTTTCATTGTTACAGCAGATTAAGAAATCTGGTTTTACGTTGTCATTCAAAAAAAATTC
TTGAAGACGCTAGAATGATGGACTTCAATTACGACCGCAATTTGGACTCAGAGGCCATTAAAAAGTGCAATGGTGAATTTAGCAAGTCAATGCCTTCCTGTACCAATGTCTCAG
ATACCACCACCGCTGTTTCTGACAACAGTGCTAAGAAGAAAGCTTCAATGGGGTCGGCGAGGGTAAATTCAACTGATACACTAACTGCATCTCCCTTATCGGGCTTAAGGAATC
AAACGCAGTTGGATTCTAAAGACAGTGTTCCATCTCTCGAGGCTTATACACCAATTGATTCTGTCTCTGACGTACCCACTGGGGAGATCAACGTTCCATTCCCTCCTGTTTATAAT
CAAAATGGATTGGATCAGCAAACCACTTATAATTTGGGAACTTTAGATGAGTTTGTTAACAAGGGAGATTTGAATGAACTCTATAATAGCCTATGGGGTGACCTATTTTCTGAT
GTTTACTTGTGA

>46

ATGCAAACATTAGAAACTACATCAAAATCAAATCCAGGGGAAGTCAAAGCACAGAAGCCTAGTACAAGAAGAACAAAAGTTGGAAAAGCTTGTGATAGCTGTAGAAGGAGG
AAAATAAAATGTAATGGGCTAAAACCTTGTCCATCTTGTACAATCTATGGTTGTGAATGTACATATACTGATGCAAAATCGACAAAAAATCTCAAATCAAATGATGCAGGTAAA
CCAAAACCAACAGGGAGAGTATCAAAGAATAAAGAAACTACTAGAATCGACAAAGATATTAGGAAATCAGAGCAGCAGTATGTACCTATTAATGCTAATATTCATGTTGGTCC
CAGGTTCCCCTCCGAGAATATATTGAATGGATATCCACAATGTGGAGCACCACAGAACAATGTTGTGGGTAATCCACTAGCGGTTAATCCTCAATGCCATAGAGGTCTTTCTGA
AACTCCTATGTCCTCAACATTCAAAGAATCTAACTTAAGAGATGATCGGCTACTACAGTCATCAGATACAGATGATATGAGGAATGGTGACTCGGAAGAAAGGGACTTGAAAG
GGAGTGACAGCGAGAATGTCAAAAGTAAAGACAATAAAAGTGATCCTTTGATTATATACAAAGATGATACACATATTGAAAGCACGGTTAATAAACTAACACAGGCAGTTAAT
GAACTCAAATCACTTCAAAATGCACCTAGTTCGATAAAATCATCCATTGACGCCATTGAGTTACAACTTAGAAACATTTTAGACAATTGGAAACCAGAGGTAGATTTCGAGAAA
GCAAAGATTAATGAAAGTGCCACCACTAAGTCACTTGAAACAAACTTGCTGAGGAATAAATACACTAATCACGTTCATCTAACAAGATTTAGGATATGGATAGATTATAAAAAT
GCGAACAAAAACAATCATTTTATGGGAGAGTGTGGATTTAGTCTTGCAGAATCTTTTTTTGCTTCTAATCAGCCATTGGTCGATGAATTGTTTGGGTTGTATTCCCAGGTAGAGG

CCTTTTCTTTGCAAGGTCTTGGTTACTGTGTTCACCTTTATGAGCCATATATGAAAACTGAGGAAGCGATAAAACTGATGAAAGAGACCTTATATATTATACTACGGTTTATTGA
TATATGTGTTCACCATATCAATGAAGAGTCGATATCGATTGCCAACCCGTTAGAAACATATTTACGAAAAAAACATCTAATGCCTATGACTCCTACACCAAGGTCGTCCTATGGA
AGTCCACAAAGTGCTAGTACAAAGAGCTTGGTAAGTAAGATAATAGAGAGAATACCGCAACCGTTTATTGAGAGTGTAACTAATGTGTCGAGTCTTCAACTATTAGATCTTCGA
GATGACGAGTCAAAAATGTTTGGAACATTGCTGAACATGTGTAAGTCTATAAGGCGAAAATTTGACTCTGTTATGAGCGATTACGATTCCATTGTCACAGAAAAATCCGAAGG
CGAACAAAATGATGGTAAAGTAACTGTAGCTGAGTTCACATCTTTGTGTGAAGCGGAAGAAATGCTCTTAGCATTATGCTATAACTATTATAATCTGACGTTATACAGTTTCTTT
GAATTTGGGACTAATATTGAATACATGGAACATCTGTTGCTTCTTCTTGAAGAACAGCTTGCTCTCGACGAATACTATGGTTTTGAAAAGGTCTTGAATGTAGCTGTTGCAAATG
CTAAAAAAATGGGTTTCCACCGTTGGGAGTTTTACGTCGGTTATGAAGAGTCGACTGCTGAAAAGAGGCGGCTACTATGGTGGAAGTTATACAATTATGAAAAAGCCAGTACT
ATGAAGAAGGGTTTTTTTTCTGTGATTGATGATGCTACTGTCAACTGTTTATTACCTAAGATTTTTAGAAACTTTGGCTATCTGGATAGGGTGGAGTTTCTAGAAAATATTCAAA
AGCCAATGGATCTTAGTGTGTTTTCCGATGTTCCAATTTCTGTCCTTTGTAAATACGGTGAGTTGGCCCTTACAATAGTTACCAGTGAGTTTCATGAAAAATTTTTATATGCTGAT
AGATACACTTCTATTCGAAATTCCGCGAAACCGCCGACATTAAAAAACCAATTAATTAAGGAAATTGTGGATGGTATAGCTTATACAGAGACATCATATGAGGCAATCAGAAA
GCAAACTGCAAAACTATGGGATATTGCATTAGGTAAGGTGACCAAAGATAAAATCAATAAAGAAGATACAGCAGCAGCTAGCAAATTTACTTTGAGTTATGAATATCACAGAT
TCAGGCTAATCAATATGGCAGACAATTTAATTGCTAGACTTATGGTGAAACCAAAATCAGATTGGCTAATATCAGTCATGAAGGGGCATCTTAACAGACTATATGAGCACTGGA
AAGTAATGAATGAAATTATCCTAAGTATGGACAACGATTATTCAATTGCAACAACGTTCGAATATTATGCACCATCATGTCTGTGTTTAGCTACGCAGACTTTCCTTATTGTGAG
GAATATGGAAATGGATGATGTCAAGATGATGGTTGCAGTATATAAAAGATTTCTTAACCTAGGAATGTTTCTGCAGAGTGCCAAAGTATGCAGCCTTGCCGATAGTCATACATT
CAGAGATTTTTCTAGATCTTTTTCCTTTATTACGATAATTTCAAGATTGATGATAATCGAATTTATGCAAATTAAAGAATTGACGAAGGTAGAGTTTATTGAGAAGTTTTCTGAAG
TATGCCCTGACCTTGCAGATCTACCTCCGATGCTTCTAGATCCAAACTCTTGCTTATATTTTTCATTGTTACAGCAGATTAAGAAATCTGGTTTTACGTTGTCATTCAAAAAAAATTC
TTGAAGACGCTAGAATGATGGACTTCAATTACGACCGCAATTTGGACTCAGAGGCCATTAAAAAGTGCAATGGTGAATTTAGCAAGTCAATGCCTTCCTGTACCAATGTCTCAG
ATACCACCACCGCTGTTTCTGACAACAGTGCTAAGAAGAAAGCTTCAATGGGGTCGGCGAGGGTAAATTCAACTGATACACTAACTGCATCTCCCTTATCGGGCTTAAGGAATC
AAACGCAGTTGGATTCTAAAGACAGTGTTCCATCTCTCGAGGCTTATACACCAATTGATTCTGTCTCTGACGTACCCACTGGGGAGATCAACGTTCCATTCCCTCCTGTTTATAAT
CAAAATGGATTGGATCAGCAAACCACTTATAATTTGGGAACTTTAGATGAGTTTGTTAACAAGGGAGATTTGAATGAACTCTATAATAGCCTATGGGGTGACCTATTTTCTGAT
GTTTACTTGTGA

>47

ATGCAAACATTAGAAACTACATCAAAATCAAATCCAGGGGAAGTCAAAGCACAGAAGCCTAGTACAAGAAGAACAAAAGTTGGAAAAGCTTGTGATAGCTGTAGAAGGAGG
AAAATAAAATGTAATGGGCTAAAACCTTGTCCATCTTGTACAATCTATGGTTGTGAATGTACATATACTGATGCAAAATCGACAAAAAATCTCAAATCAAATGATGCAGGTAAA
CCAAAACCAACAGGGAGAGTATCAAAGAATAAAGAAACTACTAGAATCGACAAAGATATTAGGAAATCAGAGCAGCAGTATGTCCCTATTAATGCTAATATTCATGTTGGTCC
CAGGTTCCCCTCCGAGAATATATTGAATGGATATCCACAATGTGGAGCACCACAGAACAATGTTGTGGGTAATCCACTAGCGGTTAATCCTCAATGCCATAGAGGTCTTTCTGA
AACTCCTATGTCCTCAACATTCAAAGAATCTAACTTAAGAGATGATCGGCTACTACAGTCATCAGATACAGATGATATGAGGAATGGTGACTCGGAAGAAAGGGACTTGAAAG
GGAGTGACAGCGAGAATGTCAAAAGTAAAGACAATAAAAGTGATCCTTTGATTATATACAAAGATGATACACATATTGAAAGCACGGTTAATAAACTAACACAGGCAGTTAAT
GAACTCAAATCACTTCAAAATGCACCTAGTTCGATAAAATCATCCATTGACGCCATTGAGTTACAACTTAGAAACATTTTAGACAATTGGAAACCAGAGGTAGATTTCGAGAAA
GCAAAGATTAATGAAAGTGCCACCACTAAGTCACTTGAAACAAACTTGCTGAGGAATAAATACACTAATCACGTTCATCTAACAAGATTTAGGATATGGATAGATTATAAAAAT

GCGAACAAAAACAATCATTTTATGGGAGAGTGTGGATTTAGTCTTGCAGAATCTTTTTTTGCTTCTAATCAGCCATTGGTCGATGAATTGTTTGGGTTGTATTCCCAGGTAGAGG
CCTTTTCTTTGCAAGGTCTTGGTTACTGTGTTCACCTTTATGAGCCATATATGAAAACTGAGGAAGCGATAAAACTGATGAAAGAGACCTTATATATTATACTACGGTTTATTGA
TATATGTGTTCACCATATCAATGAAGAGTCGATATCGATTGCCAACCCGTTAGAAACATATTTACGAAAAAAACATCTAATGCCTATGACTCCTACACCAAGGTCGTCCTATGGA
AGTCCACAAAGTGCTAGTACAAAGAGCTTGGTAAGTAAGATAATAGAGAGAATACCGCAACCGTTTATTGAGAGTGTAACTAATGTGTCGAGTCTTCAACTATTAGATCTTCGA
GATGACGAGTCAAAAATGTTTGGAACATTGCTGAACATGTGTAAGTCTATAAGGCGAAAATTTGACTCTGTTATGAGCGATTACGATTCCATTGTCACAGAAAAATCCGAAGG
CGAACAAAATGATGGTAAAGTAACTGTAGCTGAGTTCACATCTTTGTGTGAAGCGGAAGAAATGCTCTTAGCATTATGCTATAACTATTATAATCTGACGTTATACAGTTTCTTT
GAATTTGGGACTAATATTGAATACATGGAACATCTGTTGCTTCTTCTTGAAGAACAGCTTGCTCTCGACGAATACTATGGTTTTGAAAAGGTCTTGAATGTAGCTGTTGCAAATG
CTAAAAAAATGGGTTTCCACCGTTGGGAGTTTTACGTCGGTTATGAAGAGTCGACTGCTGAAAAGAGGCGGCTACTATGGTGGAAGTTATACAATTATGAAAAAGCCAGTACT
ATGAAGAAGGGTTTTTTTTCTGTGATTGATGATGCTACTGTCAACTGTTTATTACCTAAGATTTTTAGAAACTTTGGCTATCTGGATAGGGTGGAGTTTCTAGAAAATATTCAAA
AGCCAATGGATCTTAGTGTGTTTTCCGATGTTCCAATTTCTGTCCTTTGTAAATACGGTGAGTTGGCCCTTACAATAGTTACCAGTGAGTTTCATGAAAAATTTTTATATGCTGAT
AGATACACTTCTATTCGAAATTCCGCGAAACCGCCGACATTAAAAAACCAATTAATTAAGGAAATTGTGGATGGTATAGCTTATACAGAGACATCATATGAGGCAATTAGAAAG
CAAACTGCAAAACTATGGGATATTGCATTAGGTAAGGTGACCAAAGATAAAATCAATAAAGAAGATACAGCAGCAGCTAGCAAATTTACTTTGAGTTATGAATATCACAGATT
CAGGCTAATCAATATGGCAGACAATTTAATTGCTAGACTTATGGTGAAACCAAAATCAGATTGGCTAATATCAGTCATGAAGGGGCATCTTAACAGACTATATGAGCACTGGA
AAGTAATGAATGAAATTATCCTAAGTATGGACAACGATTATTCAATTGCAACAACGTTCGAATATTATGCACCATCATGTCTGTGTTTAGCTACGCAGACTTTCCTTATTGTGAG
GAATATGGAAATGGATGATGTCAAGATGATGGTTGCAGTATATAAAAGATTTCTTAACCTAGGAATGTTTCTGCAGAGTGCCAAAGTATGCAGCCTTGCCGATAGTCATACATT
CAGAGATTTTTCTAGATCTTTTTCCTTTATTACGATAATTTCAAGATTGATGATAATCGAATTTATGCAAATTAAAGAATTGACGAAGGTAGAGTTTATTGAGAAGTTTTCTGAAG
TATGCCCTGACCTTGCAGATCTACCTCCGATGCTTCTAGATCCAAACTCTTGCTTATATTTTTCATTGTTACAGCAGATTAAGAAATCTGGTTTTACGTTGTCATTCAAAAAAATTC
TTGAAGACGCTAGAATGATGGACTTCAATTACGACCGCAATTTGGACTCAGAGGCCATTAAAAAGTGCAATGGTGAATTTAGCAAGTCAATGCCTTCCTGTACCAATGTCTCAG
ATACCACCACCGCTGTTTCTGACAACAGTGCTAAGAAGAAAGCTTCAATGGGGTCGGCGAGGGTAAATTCAACTGATACACTAACTGCATCTCCCTTATCGGGCTTAAGGAATC
AAACGCAGTTGGATTCTAAAGACAGTGTTCCATCTCTCGAGGCTTATACACCAATTGATTCTGTCTCTGACGTACCCACTGGGGAGATCAACGTTCCATTCCCTCCTGTTTATAAT
CAAAATGGATTGGATCAGCAAACCACTTATAATTTGGGAACTTTAGATGAGTTTGTTAACAAGGGAGATTTGAATGAACTCTATAATAGCCTATGGGGTGACCTATTTTCTGAT
GTTTACTTGTGA

>48

ATGCAAACATTAGAAACTACATCAAAATCAAATCCAGGGGAAGTCAAAGCACAGAAGCCTAGTACAAGAAGAACAAAAGTTGGAAAAGCTTGTGATAGCTGTAGAAGGAGG
AAAATAAAATGTAATGGGCTAAAACCTTGTCCATCTTGTACAATCTATGGTTGTGAATGTACATATACTGATGCAAAATCGACAAAAAATCTCAAATCAAATGATGCAGGTAAA
CCAAAACCAACAGGGAGAGTATCAAAGAATAAAGAAACTACTAGAATCGACAAAGATATTAGGAAATCAGAGCAGCAGTATGTCCCTATTAATGCTAATATTCATGTTGGTCC
CAGGTTCCCCTCCGAGAATATATTGAATGGATATCCACAATGTGGAGCACCACAGAACAATGTTGTGGGTAATCCACTAGCGGTTAATCCTCAATGCCATAGAGGTCTTTCTGA
AACTCCTATGTCCTCAACATTCAAAGAATCTAACTTAAGAGATGATCGGCTACTACAGTCATCAGATACAGATGATATGAGGAATGGTGACTCGGAAGAAAGGGACTTGAAAG
GGAGTGACAGCGAGAATGTCAAAAGTAAAGACAATAAAAGTGATCCTTTGATTATATACAAAGATGATACACATATTGAAAGCACGGTTAATAAACTAACACAGGCAGTTAAT
GAACTCAAATCACTTCAAAATGCACCTAGTTCGATAAAATCATCCATTGACGCCATTGAGTTACAACTTAGAAACATTTTAGACAATTGGAAACCAGAGGTAGATTTCGAGAAA

GCAAAGATTAATGAAAGTGCCACCACTAAGTCACTTGAAACAAACTTGCTGAGGAATAAATACACTAATCACGTTCATCTAACAAGATTTAGGATATGGATAGATTATAAAAAT
GCGAACAAAAACAATCATTTTATGGGAGAGTGTGGATTTAGTCTTGCAGAATCTTTTTTTGCTTCTAATCAGCCATTGGTCGATGAATTGTTTGGGTTGTATTCCCAGGTAGAGG
CCTTTTCTTTGCAAGGTCTTGGTTACTGTGTTCACCTTTATGAGCCATATATGAAAACTGAGGAAGCGATAAAACTGATGAAAGAGACCTTATATATTATACTACGGTTTATTGA
TATATGTGTTCACCATATCAATGAAGAGTCGATATCGATTGCCAACCCGTTAGAAACATATTTACGAAAAAAACATCTAATGCCTATGACTCCTACACCAAGGTCGTCCTATGGA
AGTCCACAAAGTGCTAGTACAAAGAGCTTGGTAAGTAAGATAATAGAGAGAATACCGCAACCGTTTATTGAGAGTGTAACTAATGTGTCGAGTCTTCAACTATTAGATCTTCGA
GATGACGAGTCAAAAATGTTTGGAACATTGCTGAACATGTGTAAGTCTATAAGGCGAAAATTTGACTCTGTTATGAGCGATTACGATTCCATTGTCACAGAAAAATCCGAAGG
CGAACAAAATGATGGTAAAGTAACTGTAGCTGAGTTCACATCTTTGTGTGAAGCGGAAGAAATGCTCTTAGCATTATGCTATAACTATTATAATCTGACGTTATACAGTTTCTTT
GAATTTGGGACTAATATTGAATACATGGAACATCTGTTGCTTCTTCTTGAAGAACAGCTTGCTCTCGACGAATACTATGGTTTTGAAAAGGTCTTGAATGTAGCTGTTGCAAATG
CTAAAAAAATGGGTTTCCACCGTTGGGAGTTTTACGTCGGTTATGAAGAGTCGACTGCTGAAAAGAGGCGGCTACTATGGTGGAAGTTATACAATTATGAAAAAGCCAGTACT
ATGAAGAAGGGTTTTTTTTCTGTGATTGATGATGCTACTGTCAACTGTTTATTACCTAAGATTTTTAGAAACTTTGGCTATCTGGATAGGGTGGAGTTTCTAGAAAATATTCAAA
AGCCAATGGATCTTAGTGTGTTTTCCGATGTTCCAATTTCTGTCCTTTGTAAATACGGTGAGTTGGCCCTTACAATAGTTACCAGTGAGTTTCATGAAAAATTTTTATATGCTGAT
AGATACACTTCTATTCGAAATTCCGCGAAACCGCCGACATTAAAAAACCAATTAATTAAGGAAATTGTGGATGGTATAGCTTATACAGAGACATCATATGAGGCAATTAGAAAG
CAAACTGCAAAACTATGGGATATTGCATTAGGTAAGGTGACCAAAGATAAAATCAATAAAGAAGATACAGCAGCAGCTAGCAAATTTACTTTGAGTTATGAATATCACAGATT
CAGGCTAATCAATATGGCAGACAATTTAATTGCTAGACTTATGGTGAAACCAAAATCAGATTGGCTAATATCAGTCATGAAGGGGCATCTTAACAGACTATATGAGCACTGGA
AAGTAATGAATGAAATTATCCTAAGTATGGACAACGATTATTCAATTGCAACAACGTTCGAATATTATGCACCATCATGTCTGTGTTTAGCTACGCAGACTTTCCTTATTGTGAG
GAATATGGAAATGGATGATGTCAAGATGATGGTTGCAGTATATAAAAGATTTCTTAACCTAGGAATGTTTCTGCAGAGTGCCAAAGTATGCAGCCTTGCCGATAGTCATACATT
CAGAGATTTTTCTAGATCTTTTTCCTTTATTACGATAATTTCAAGATTGATGATAATCGAATTTATGCAAATTAAAGAATTGACGAAGGTAGAGTTTATTGAGAAGTTTTCTGAAG
TATGCCCTGACCTTGCAGATCTACCTCCGATGCTTCTAGATCCAAACTCTTGCTTATATTTTTCATTGTTACAGCAGATTAAGAAATCTGGTTTTACGTTGTCATTCAAAAAAATTC
TTGAAGACGCTAGAATGATGGACTTCAATTACGACCGCAATTTGGACTCAGAGGCCATTAAAAAGTGCAATGGTGAATTTAGCAAGTCAATGCCTTCCTGTACCAATGTCTCAG
ATACCACCACCGCTGTTTCTGACAACAGTGCTAAGAAGAAAGCTTCAATGGGGTCGGCGAGGGTAAATTCAACTGATACACTAACTGCATCTCCCTTATCGGGCTTAAGGAATC
AAACGCAGTTGGATTCTAAAGACAGTGTTCCATCTCTCGAGGCTTATACACCAATTGATTCTGTCTCTGACGTACCCACTGGGGAGATCAACGTTCCATTCCCTCCTGTTTATAAT
CAAAATGGATTGGATCAGCAAACCACTTATAATTTGGGAACTTTAGATGAGTTTGTTAACAAGGGAGATTTGAATGAACTCTATAATAGCCTATGGGGTGACCTATTTTCTGAT
GTTTACTTGTGA

>49

ATGCAAACATTAGAAACTACATCAAAATCAAATCCAGGGGAAGTCAAAGCACAGAAGCCTAGTACAAGAAGAACAAAAGTTGGAAAAGCTTGTGATAGCTGTAGAAGGAGG
AAAATAAAATGTAATGGGCTAAAACCTTGTCCATCTTGTACAATCTATGGTTGTGAATGTACATATACTGATGCAAAATCGACAAAAAATCTCAAATCAAATGATGCAGGTAAA
CCAAAACCAACAGGGAGAGTATCAAAGAATAAAGAAACTACTAGAATCGACAAAGATATTAGGAAATCAGAGCAGCAGTATGTCCCTATTAATGCTAATATTCATGTTGGTCC
CAGGTTCCCCTCCGAGAATATATTGAATGGATATCCACAATGTGGAGCACCACAGAACAATGTTGTGGGTAATCCACTAGCGGTTAATCCTCAATGCCATAGAGGTCTTTCTGA
AACTCCTATGTCCTCAACATTCAAAGAATCTAACTTAAGAGATGATCGGCTACTACAGTCATCAGATACAGATGATATGAGGAATGGTGACTCGGAAGAAAGGGACTTGAAAG
GGAGTGACAGCGAGAATGTCAAAAGTAAAGACAATAAAAGTGATCCTTTGATTATATACAAAGATGATACACATATTGAAAGCACGGTTAATAAACTAACACAGGCAGTTAAT

GAACTCAAATCACTTCAAAATGCACCTAGTTCGATAAAATCATCCATTGACGCCATTGAGTTACAACTTAGAAACATTTTAGACAATTGGAAACCAGAGGTAGATTTCGAGAAA
GCAAAGATTAATGAAAGTGCCACCACTAAGTCACTTGAAACAAACTTGCTGAGGAATAAATACACTAATCACGTTCATCTAACAAGATTTAGGATATGGATAGATTATAAAAAT
GCGAACAAAAACAATCATTTTATGGGAGAGTGTGGATTTAGTCTTGCAGAATCTTTTTTTGCTTCTAATCAGCCATTGGTCGATGAATTGTTTGGGTTGTATTCCCAGGTAGAGG
CCTTTTCTTTGCAAGGTCTTGGTTACTGTGTTCACCTTTATGAGCCATATATGAAAACTGAGGAAGCGATAAAACTGATGAAAGAGACCTTATATATTATACTACGGTTTATTGA
TATATGTGTTCACCATATCAATGAAGAGTCGATATCGATTGCCAACCCGTTAGAAACATATTTACGAAAAAAACATCTAATGCCTATGACTCCTACACCAAGGTCGTCCTATGGA
AGTCCACAAAGTGCTAGTACAAAGAGCTTGGTAAGTAAGATAATAGAGAGAATACCGCAACCGTTTATTGAGAGTGTAACTAATGTGTCGAGTCTTCAACTATTAGATCTTCGA
GATGACGAGTCAAAAATGTTTGGAACATTGCTGAACATGTGTAAGTCTATAAGGCGAAAATTTGACTCTGTTATGAGCGATTACGATTCCATTGTCACAGAAAAATCCGAAGG
CGAACAAAATGATGGTAAAGTAACTGTAGCTGAGTTCACATCTTTGTGTGAAGCGGAAGAAATGCTCTTAGCATTATGCTATAACTATTATAATCTGACGTTATACAGTTTCTTT
GAATTTGGGACTAATATTGAATACATGGAACATCTGTTGCTTCTTCTTGAAGAACAGCTTGCTCTCGACGAATACTATGGTTTTGAAAAGGTCTTGAATGTAGCTGTTGCAAATG
CTAAAAAAATGGGTTTCCACCGTTGGGAGTTTTACGTCGGTTATGAAGAGTCGACTGCTGAAAAGAGGCGGCTACTATGGTGGAAGTTATACAATTATGAAAAAGCCAGTACT
ATGAAGAAGGGTTTTTTTTCTGTGATTGATGATGCTACTGTCAACTGTTTATTACCTAAGATTTTTAGAAACTTTGGCTATCTGGATAGGGTGGAGTTTCTAGAAAATATTCAAA
AGCCAATGGATCTTAGTGTGTTTTCCGATGTTCCAATTTCTGTCCTTTGTAAATACGGTGAGTTGGCCCTTACAATAGTTACCAGTGAGTTTCATGAAAAATTTTTATATGCTGAT
AGATACACTTCTATTCGAAATTCCGCGAAACCGCCGACATTAAAAAACCAATTAATTAAGGAAATTGTGGATGGTATAGCTTATACAGAGACATCATATGAGGCAATCAGAAA
GCAAACTGCAAAACTATGGGATATTGCATTAGGTAAGGTGACCAAAGATAAAATCAATAAAGAAGATACAGCAGCAGCTAGCAAATTTACTTTGAGTTATGAATATCACAGAT
TCAGGCTAATCAATATGGCAGACAATTTAATTGCTAGACTTATGGTGAAACCAAAATCAGATTGGCTAATATCAGTCATGAAGGGGCATCTTAACAGACTATATGAGCACTGGA
AAGTAATGAATGAAATTATCCTAAGTATGGACAACGATTATTCAATTGCAACAACGTTCGAATATTATGCACCATCATGTCTGTGTTTAGCTACGCAGACTTTCCTTATTGTGAG
GAATATGGAAATGGATGATGTCAAGATGATGGTTGCAGTATATAAAAGATTTCTTAACCTAGGAATGTTTCTGCAGAGTGCCAAAGTATGCAGCCTTGCCGATAGTCATACATT
CAGAGATTTTTCTAGATCTTTTTCCTTTATTACGATAATTTCAAGATTGATGATAATCGAATTTATGCAAATTAAAGAATTGACGAAGGTAGAGTTTATTGAGAAGTTTTCTGAAG
TATGCCCTGACCTTGCAGATCTACCTCCGATGCTTCTAGATCCAAACTCTTGCTTATATTTTTCATTGTTACAGCAGATTAAGAAATCTGGTTTTACGTTGTCATTCAAAAAAATTC
TTGAAGACGCTAGAATGATGGACTTCAATTACGACCGCAATTTGGACTCAGAGGCCATTAAAAAGTGCAATGGTGAATTTAGCAAGTCAATGCCTTCCTGTACCAATGTCTCAG
ATACCACCACCGCTGTTTCTGACAACAGTGCTAAGAAGAAAGCTTCAATGGGGTCGGCGAGGGTAAATTCAACTGATACACTAACTGCATCTCCCTTATCGGGCTTAAGGAATC
AAACGCAGTTGGATTCTAAAGACAGTGTTCCATCTCTCGAGGCTTATACACCAATTGATTCTGTCTCTGACGTACCCACTGGGGAGATCAACGTTCCATTCCCTCCTGTTTATAAT
CAAAATGGATTGGATCAGCAAACCACTTATAATTTGGGAACTTTAGATGAGTTTGTTAACAAGGGAGATTTGAATGAACTCTATAATAGCCTATGGGGTGACCTATTTTCTGAT
GTTTACTTGTGA

>50

ATGCAAACATTAGAAACTACATCAAAATCAAATCCAGGGGAAGTCAAAGCACAGAAGCCTAGTACAAGAAGAACAAAAGTTGGAAAAGCTTGTGATAGCTGTAGAAGGAGG
AAAATAAAATGTAATGGGCTAAAACCTTGTCCATCTTGTACAATCTATGGTTGTGAATGTACATATACTGATGCAAAATCGACAAAAAATCTCAAATCAAATGATGCAGGTAAA
CCAAAACCAACAGGGAGAGTATCAAAGAATAAAGAAACTACTAGAATCGACAAAGATATTAGGAAATCAGAGCAGCAGTATGTCCCTATTAATGCTAATATTCATGTTGGTCC
CAGGTTCCCCTCCGAGAATATATTGAATGGATATCCACAATGTGGAGCACCACAGAACAATGTTGTGGGTAATCCACTAGCGGTTAATCCTCAATGCCATAGAGGTCTTTCTGA
AACTCCTATGTCCTCAACATTCAAAGAATCTAACTTAAGAGATGATCGGCTACTACAGTCATCAGATACAGATGATATGAGGAATGGTGACTCGGAAGAAAGGGACTTGAAAG

GGAGTGACAGCGAGAATGTCAAAAGTAAAGACAATAAAAGTGATCCTTTGATTATATACAAAGATGATACACATATTGAAAGCACGGTTAATAAACTAACACAGGCAGTTAAT
GAACTCAAATCACTTCAAAATGCACCTAGTTCGATAAAATCATCCATTGACGCCATTGAGTTACAACTTAGAAACATTTTAGACAATTGGAAACCAGAGGTAGATTTCGAGAAA
GCAAAGATTAATGAAAGTGCCACCACTAAGTCACTTGAAACAAACTTGCTGAGGAATAAATACACTAATCACGTTCATCTAACAAGATTTAGGATATGGATAGATTATAAAAAT
GCGAACAAAAACAATCATTTTATGGGAGAGTGTGGATTTAGTCTTGCAGAATCTTTTTTTGCTTCTAATCAGCCATTGGTCGATGAATTGTTTGGGTTGTATTCCCAGGTAGAGG
CCTTTTCTTTGCAAGGTCTTGGTTACTGTGTTCACCTTTATGAGCCATATATGAAAACTGAGGAAGCGATAAAACTGATGAAAGAGACCTTATATATTATACTACGGTTTATTGA
TATATGTGTTCACCATATCAATGAAGAGTCGATATCGATTGCCAACCCGTTAGAAACATATTTACGAAAAAAACATCTAATGCCTATGACTCCTACACCAAGGTCGTCCTATGGA
AGTCCACAAAGTGCTAGTACAAAGAGCTTGGTAAGTAAGATAATAGAGAGAATACCGCAACCGTTTATTGAGAGTGTAACTAATGTGTCGAGTCTTCAACTATTAGATCTTCGA
GATGACGAGTCAAAAATGTTTGGAACATTGCTGAACATGTGTAAGTCTATAAGGCGAAAATTTGACTCTGTTATGAGCGATTACGATTCCATTGTCACAGAAAAATCCGAAGG
CGAACAAAATGATGGTAAAGTAACTGTAGCTGAGTTCACATCTTTGTGTGAAGCGGAAGAAATGCTCTTAGCATTATGCTATAACTATTATAATCTGACGTTATACAGTTTCTTT
GAATTTGGGACTAATATTGAATACATGGAACATCTGTTGCTTCTTCTTGAAGAACAGCTTGCTCTCGACGAATACTATGGTTTTGAAAAGGTCTTGAATGTAGCTGTTGCAAATG
CTAAAAAAATGGGTTTCCACCGTTGGGAGTTTTACGTCGGTTATGAAGAGTCGACTGCTGAAAAGAGGCGGCTACTATGGTGGAAGTTATACAATTATGAAAAAGCCAGTACT
ATGAAGAAGGGTTTTTTTTCTGTGATTGATGATGCTACTGTCAACTGTTTATTACCTAAGATTTTTAGAAACTTTGGCTATCTGGATAGGGTGGAGTTTCTAGAAAATATTCAAA
AGCCAATGGATCTTAGTGTGTTTTCCGATGTTCCAATTTCTGTCCTTTGTAAATACGGTGAGTTGGCCCTTACAATAGTTACCAGTGAGTTTCATGAAAAATTTTTATATGCTGAT
AGATACACTTCTATTCGAAATTCCGCGAAACCGCCGACATTAAAAAACCAATTAATTAAGGAAATTGTGGATGGTATAGCTTATACAGAGACATCATATGAGGCAATCAGAAA
GCAAACTGCAAAACTATGGGATATTGCATTAGGTAAGGTGACCAAAGATAAAATCAATAAAGAAGATACAGCAGCAGCTAGCAAATTTACTTTGAGTTATGAATATCACAGAT
TCAGGCTAATCAATATGGCAGACAATTTAATTGCTAGACTTATGGTGAAACCAAAATCAGATTGGCTAATATCAGTCATGAAGGGGCATCTTAACAGACTATATGAGCACTGGA
AAGTAATGAATGAAATTATCCTAAGTATGGACAACGATTATTCAATTGCAACAACGTTCGAATATTATGCACCATCATGTCTGTGTTTAGCTACGCAGACTTTCCTTATTGTGAG
GAATATGGAAATGGATGATGTCAAGATGATGGTTGCAGTATATAAAAGATTTCTTAACCTAGGAATGTTTCTGCAGAGTGCCAAAGTATGCAGCCTTGCCGATAGTCATACATT
CAGAGATTTTTCTAGATCTTTTTCCTTTATTACGATAATTTCAAGATTGATGATAATCGAATTTATGCAAATTAAAGAATTGACGAAGGTAGAGTTTATTGAGAAGTTTTCTGAAG
TATGCCCTGACCTTGCAGATCTACCTCCGATGCTTCTAGATCCAAACTCTTGCTTATATTTTTCATTGTTACAGCAGATTAAGAAATCTGGTTTTACGTTGTCATTCAAAAAAATTC
TTGAAGACGCTAGAATGATGGACTTCAATTACGACCGCAATTTGGACTCAGAGGCCATTAAAAAGTGCAATGGTGAATTTAGCAAGTCAATGCCTTCCTGTACCAATGTCTCAG
ATACCACCACCGCTGTTTCTGACAACAGTGCTAAGAAGAAAGCTTCAATGGGGTCGGCGAGGGTAAATTCAACTGATACACTAACTGCATCTCCCTTATCGGGCTTAAGGAATC
AAACGCAGTTGGATTCTAAAGACAGTGTTCCATCTCTCGAGGCTTATACACCAATTGATTCTGTCTCTGACGTACCCACTGGGGAGATCAACGTTCCATTCCCTCCTGTTTATAAT
CAAAATGGATTGGATCAGCAAACCACTTATAATTTGGGAACTTTAGATGAGTTTGTTAACAAGGGAGATTTGAATGAACTCTATAATAGCCTATGGGGTGACCTATTTTCTGAT
GTTTACTTGTGA

>51

ATGCAAACATTAGAAACTACATCAAAATCAAATCCAGGGGAAGTCAAAGCACAGAAGCCTAGTACAAGAAGAACAAAAGTTGGAAAAGCTTGTGATAGCTGTAGAAGGAGG
AAAATAAAATGTAATGGGCTAAAACCTTGTCCATCTTGTACAATCTATGGTTGTGAATGTACATATACTGATGCAAAATCGACAAAAAATCTCAAATCAAATGATGCAGGTAAA
CCAAAACCAACAGGGAGAGTATCAAAGAATAAAGAAACTACTAGAATCGACAAAGATATTAGGAAATCAGAGCAGCAGTATGTCCCTATTAATGCTAATATTCATGTTGGTCC
CAGGTTCCCCTCCGAGAATATATTGAATGGATATCCACAATGTGGAGCACCACAGAACAATGTTGTGGGTAATCCACTAGCGGTTAATCCTCAATGCCATAGAGGTCTTTCTGA

AACTCCTATGTCCTCAACATTCAAAGAATCTAACTTGAGAGATGATCGGCTACTACAGTCATCAGATACAGATGATATGAGGAATGGTGACTCGGAAGAAAGGGACTTGAAAG
GGAGTGACAGCGAGAATGTCAAAAGTAAAGACAATAAAAGTGATCCTTTGATTATATACAAAGATGATACACATATTGAAAGCACGGTTAATAAACTAACACAGGCAGTTAAT
GAACTCAAATCACTTCAAAATGCACCTAGTTCGATAAAATCATCCATTGACGCCATTGAGTTACAACTTAGAAACATTTTAGACAATTGGAAACCAGAGGTAGATTTCGAGAAA
GCAAAGATTAATGAAAGTGCCACCACTAAGTCACTTGAAACAAACTTGCTGAGGAATAAATACACTAATCACGTTCATCTAACAAGATTTAGGATATGGATAGATTATAAAAAT
GCGAACAAAAACAATCATTTTATGGGAGAGTGTGGATTTAGTCTTGCAGAATCTTTTTTTGCTTCTAATCAGCCATTGGTCGATGAATTGTTTGGGTTGTATTCCCAGGTAGAGG
CCTTTTCTTTGCAAGGTCTTGGTTACTGTGTTCACCTTTATGAGCCATATATGAAAACTGAGGAAGCGATAAAACTGATGAAAGAGACCTTATATATTATACTACGGTTTATTGA
TATATGTGTTCACCATATCAATGAAGAGTCGATATCGATTGCCAACCCGTTAGAAACATATTTACGAAAAAAACATCTAATGCCTATGACTCCTACACCAAGGTCGTCCTATGGA
AGTCCACAAAGTGCTAGTACAAAGAGCTTGGTAAGTAAGATAATAGAGAGAATACCGCAACCGTTTATTGAGAGTGTAACTAATGTGTCGAGTCTTCAACTATTAGATCTTCGA
GATGACGAGTCAAAAATGTTTGGAACATTGCTGAACATGTGTAAGTCTATAAGGCGAAAATTTGACTCTGTTATGAGCGATTACGATTCCATTGTCACAGAAAAATCCGAAGG
CGAACAAAATGATGGTAAAGTAACTGTAGCTGAGTTCACATCTTTGTGTGAAGCGGAAGAAATGCTCTTAGCATTATGCTATAACTATTATAATCTGACGTTATACAGTTTCTTT
GAATTTGGGACTAATATTGAATACATGGAACATCTGTTGCTTCTTCTTGAAGAACAGCTTGCTCTCGACGAATACTATGGTTTTGAAAAGGTCTTGAATGTAGCTGTTGCAAATG
CTAAAAAAATGGGTTTCCACCGTTGGGAGTTTTACGTCGGTTATGAAGAGTCGACTGCTGAAAAGAGGCGGCTACTATGGTGGAAGTTATACAATTATGAAAAAGCCAGTACT
ATGAAGAAGGGTTTTTTTTCTGTGATTGATGATGCTACTGTCAACTGTTTATTACCTAAGATTTTTAGAAACTTTGGCTATCTGGATAGGGTGGAGTTTCTAGAAAATATTCAAA
AGCCAATGGATCTTAGTGTGTTTTCCGATGTTCCAATTTCTGTCCTTTGTAAATACGGTGAGTTGGCCCTTACAATAGTTACCAGTGAGTTTCATGAAAAATTTTTATATGCTGAT
AGATACACTTCTATTCGAAATTCCGCGAAACCGCCGACATTAAAAAACCAATTAATTAAGGAAATTGTGGATGGTATAGCTTATACAGAGACATCATATGAGGCAATCAGAAA
GCAAACTGCAAAACTATGGGATATTGCATTAGGTAAGGTGACCAAAGATAAAATCAATAAAGAAGATACAGCAGCAGCTAGCAAATTTACTTTGAGTTATGAATATCACAGAT
TCAGGCTAATCAATATGGCAGACAATTTAATTGCTAGACTTATGGTGAAACCAAAATCAGATTGGCTAATATCAGTCATGAAGGGGCATCTTAACAGACTATATGAGCACTGGA
AAGTAATGAATGAAATTATCCTAAGTATGGACAAYGATTATTCAATTGCAACAACGTTCGAATATTATGCACCATCATGTCTGTGTTTAGCTACGCAGACTTTCCTTATTGTGAG
GAATATGGAAATGGATGATGTCAAGATGATGGTTGCAGTATATAAAAGATTTCTTAACCTAGGAATGTTTCTGCAGAGTGCCAAAGTATGCAGCCTTGCCGATAGTCATACATT
CAGAGATTTTTCTAGATCTTTTTCCTTTATTACGATAATTTCAAGATTGATGATAATCGAATTTATGCAAATTAAAGAATTGACGAAGGTAGAGTTTATTGAGAAGTTTTCTGAAG
TATGCCCTGACCTTGCAGATCTACCTCCGATGCTTCTAGATCCAAACTCTYGCTTATATTTTTCATTGTTACAGCAGATTAAGAAATCTGGTTTTACGTTGTCATTCAAAAAAATTC
TTGAAGACGCTAGAATGATGGACTTCAATTACGACCGCAATTTGGACTCAGAGGCCATTAAAAAGTGCAATGGTGAATTTAGCAAGTCAATGCCTTCCTGTACCAATGTCTCAG
ATACCACCACCGCTGTTTCTGACAACAGTGCTAAGAAGAAAGCTTCAATGGGGTCGGCGAGGGTAAATTCAACTGATACACTAACTGCATCTCCCTTATCGGGCTTAAGGAATC
AAACGCAGTTGGATTCTAAAGACAGTGTTCCATCTCTCGAGGCTTATACACCAATTGATTCTGTCTCTGACGTACCCACTGGGGAGATCAACGTTCCATTCCCTCCTGTTTATAAT
CAAAATGGATTGGATCAGCAAACCACTTATAATTTGGGAACTTTAGATGAGTTTGTTAACAAGGGAGATTTGAATGAACTCTATAATAGCCTATGGGGTGACCTATTTTCTGAT
GTTTACTTGTGA

>52

ATGCAAACATTAGAAACTACATCAAAATCAAATCCAGGGGAAGTCAAAGCACAGAAGCCTAGTACAAGAAGAACAAAAGTTGGAAAAGCTTGTGATAGCTGTAGAAGGAGG
AAAATAAAATGTAATGGGCTAAAACCTTGTCCATCTTGTACAATCTATGGTTGTGAATGTACATATACTGATGCAAAATCGACAAAAAATCTCAAATCAAATGATGCAGGTAAA
CCAAAACCAACAGGGAGAGTATCAAAGAATAAAGAAACTACTAGAATCGACAAAGATATTAGGAAATCAGAGCAGCAGTATGTCCCTATTAATGCTAATATTCATGTTGGTCC

CAGGTTCCCCTCCGAGAATATATTGAATGGATATCCACAATGTGGAGCACCACAGAACAATGTTGTGGGTAATCCACTAGCGGTTAATCCTCAATGCCATAGAGGTCTTTCTGA
AACTCCTATGTCCTCAACATTCAAAGAATCTAACTTGAGAGATGATCGGCTACTACAGTCATCAGATACAGATGATATGAGGAATGGTGACTCGGAAGAAAGGGACTTGAAAG
GGAGTGACAGCGAGAATGTCAAAAGTAAAGACAATAAAAGTGATCCTTTGATTATATACAAAGATGATACACATATTGAAAGCACGGTTAATAAACTAACACAGGCAGTTAAT
GAACTCAAATCACTTCAAAATGCACCTAGTTCGATAAAATCATCCATTGACGCCATTGAGTTACAACTTAGAAACATTTTAGACAATTGGAAACCAGAGGTAGATTTCGAGAAA
GCAAAGATTAATGAAAGTGCCACCACTAAGTCACTTGAAACAAACTTGCTGAGGAATAAATACACTAATCACGTTCATCTAACAAGATTTAGGATATGGATAGATTATAAAAAT
GCGAACAAAAACAATCATTTTATGGGAGAGTGTGGATTTAGTCTTGCAGAATCTTTTTTTGCTTCTAATCAGCCATTGGTCGATGAATTGTTTGGGTTGTATTCCCAGGTAGAGG
CCTTTTCTTTGCAAGGTCTTGGTTACTGTGTTCACCTTTATGAGCCATATATGAAAACTGAGGAAGCGATAAAACTGATGAAAGAGACCTTATATATTATACTACGGTTTATTGA
TATATGTGTTCACCATATCAATGAAGAGTCGATATCGATTGCCAACCCGTTAGAAACATATTTACGAAAAAAACATCTAATGCCTATGACTCCTACACCAAGGTCGTCCTATGGA
AGTCCACAAAGTGCTAGTACAAAGAGCTTGGTAAGTAAGATAATAGAGAGAATACCGCAACCGTTTATTGAGAGTGTAACTAATGTGTCGAGTCTTCAACTATTAGATCTTCGA
GATGACGAGTCAAAAATGTTTGGAACATTGCTGAACATGTGTAAGTCTATAAGGCGAAAATTTGACTCTGTTATGAGCGATTACGATTCCATTGTCACAGAAAAATCCGAAGG
CGAACAAAATGATGGTAAAGTAACTGTAGCTGAGTTCACATCTTTGTGTGAAGCGGAAGAAATGCTCTTAGCATTATGCTATAACTATTATAATCTGACGTTATACAGTTTCTTT
GAATTTGGGACTAATATTGAATACATGGAACATCTGTTGCTTCTTCTTGAAGAACAGCTTGCTCTCGACGAATACTATGGTTTTGAAAAGGTCTTGAATGTAGCTGTTGCAAATG
CTAAAAAAATGGGTTTCCACCGTTGGGAGTTTTACGTCGGTTATGAAGAGTCGACTGCTGAAAAGAGGCGGCTACTATGGTGGAAGTTATACAATTATGAAAAAGCCAGTACT
ATGAAGAAGGGTTTTTTTTCTGTGATTGATGATGCTACTGTCAACTGTTTATTACCTAAGATTTTTAGAAACTTTGGCTATCTGGATAGGGTGGAGTTTCTAGAAAATATTCAAA
AGCCAATGGATCTTAGTGTGTTTTCCGATGTTCCAATTTCTGTCCTTTGTAAATACGGTGAGTTGGCCCTTACAATAGTTACCAGTGAGTTTCATGAAAAATTTTTATATGCTGAT
AGATACACTTCTATTCGAAATTCCGCGAAACCGCCGACATTAAAAAACCAATTAATTAAGGAAATTGTGGATGGTATAGCTTATACAGAGACATCATATGAGGCAATCAGAAA
GCAAACTGCAAAACTATGGGATATTGCATTAGGTAAGGTGACCAAAGATAAAATCAATAAAGAAGATACAGCAGCAGCTAGCAAATTTACTTTGAGTTATGAATATCACAGAT
TCAGGCTAATCAATATGGCAGACAATTTAATTGCTAGACTTATGGTGAAACCAAAATCAGATTGGCTAATATCAGTCATGAAGGGGCATCTTAACAGACTATATGAGCACTGGA
AAGTAATGAATGAAATTATCCTAAGTATGGACAACGATTATTCAATTGCAACAACGTTCGAATATTATGCACCATCATGTCTGTGTTTAGCTACGCAGACTTTCCTTATTGTGAG
GAATATGGAAATGGATGATGTCAAGATGATGGTTGCAGTATATAAAAGATTTCTTAACCTAGGAATGTTTCTGCAGAGTGCCAAAGTATGCAGCCTTGCCGATAGTCATACATT
CAGAGATTTTTCTAGATCTTTTTCCTTTATTACGATAATTTCAAGATTGATGATAATCGAATTTATGCAAATTAAAGAATTGACGAAGGTAGAGTTTATTGAGAAGTTTTCTGAAG
TATGCCCTGACCTTGCAGATCTACCTCCGATGCTTCTAGATCCAAACTCTTGCTTATATTTTTCATTGTTACAGCAGATTAAGAAATCTGGTTTTACGTTGTCATTCAAAAAAAATTC
TTGAAGACGCTAGAATGATGGACTTCAATTACGACCGCAATTTGGACTCAGAGGCCATTAAAAAGTGCAATGGTGAATTTAGCAAGTCAATGCCTTCCTGTACCAATGTCTCAG
ATACCACCACCGCTGTTTCTGACAACAGTGCTAAGAAGAAAGCTTCAATGGGGTCGGCGAGGGTAAATTCAACTGATACACTAACTGCATCTCCCTTATCGGGCTTAAGGAATC
AAACGCAGTTGGATTCTAAAGACAGTGTTCCATCTCTCGAGGCTTATACACCAATTGATTCTGTCTCTGACGTACCCACTGGGGAGATCAACGTTCCATTCCCTCCTGTTTATAAT
CAAAATGGATTGGATCAGCAAACCACTTATAATTTGGGAACTTTAGATGAGTTTGTTAACAAGGGAGATTTGAATGAACTCTATAATAGCCTATGGGGTGACCTATTTTCTGAT
GTTTACTTGTGA

>53

ATGCAAACATTAGAAACTACATCAAAATCAAATCCAGGGGAAGTCAAAGCACAGAAGCCTAGTACAAGAAGAACAAAAGTTGGAAAAGCTTGTGATAGCTGTAGAAGGAGG
AAAATAAAATGTAATGGGCTAAAACCTTGTCCATCTTGTACAATCTATGGTTGTGAATGTACATATACTGATGCAAAATCGACAAAAAATCTCAAATCAAATGATGCAGGTAAA

CCAAAACCAACAGGGAGAGTATCAAAGAATAAAGAAACTACTAGAATCGACAAAGATATTAGGAAATCAGAGCAGCAGTATGTCCCTATTAATGCTAATATTCATGTTGGTCC
CAGGTTCCCCTCCGAGAATATATTGAATGGATATCCACAATGTGGAGCACCACAGAACAATGTTGTGGGTAATCCACTAGCGGTTAATCCTCAATGCCATAGAGGTCTTTCTGA
AACTCCTATGTCCTCAACATTCAAAGAATCTAACTTAAGAGATGATCGGCTACTACAGTCATCAGATACAGATGATATGAGGAATGGTGACTCGGAAGAAAGGGACTTGAAAG
GGAGTGACAGCGAGAATGTCAAAAGTAAAGACAATAAAAGTGATCCTTTGATTATATACAAAGATGATACACATATTGAAAGCACGGTTAATAAACTAACACAGGCAGTTAAT
GAACTCAAATCACTTCAAAATGCACCTAGTTCGATAAAATCATCCATTGACGCCATTGAGTTACAACTTAGAAACATTTTAGACAATTGGAAACCAGAGGTAGATTTCGAGAAA
GCAAAGATTAATGAAAGTGCCACCACTAAGTCACTTGAAACAAATTTGCTGAGGAATAAATACACTAATCACGTTCATCTAACAAGATTTAGGATATGGATAGATTATAAAAAT
GCGAACAAAAACAATCATTTTATGGGAGAGTGTGGATTTAGTCTTGCAGAATCTTTTTTTGCTTCTAATCAGCCATTGGTCGATGAATTGTTTGGGTTGTATTCCCAGGTAGAGG
CCTTTTCTTTGCAAGGTCTTGGTTACTGTGTTCACCTTTATGAGCCATATATGAAAACTGAGGAAGCGATAAAACTGATGAAAGAGACCTTATATATTATACTACGGTTTATTGA
TATATGTGTTCACCATATCAATGAAGAGTCGATATCGATTGCCAACCCGTTAGAAACATATTTACGAAAAAAACATCTAATGCCTATGACTCCTACACCAAGGTCGTCCTATGGA
AGTCCACAAAGTGCTAGTACAAAGAGCTTGGTAAGTAAGATAATAGAGAGAATACCGCAACCGTTTATTGAGAGTGTAACTAATGTGTCGAGTCTTCAACTATTAGATCTTCGA
GATGACGAGTCAAAAATGTTTGGAACATTGCTGAACATGTGTAAGTCTATAAGGCGAAAATTTGACTCTGTTATGAGCGATTACGATTCCATTGTCACAGAAAAATCCGAAGG
CGAACAAAATGATGGTAAAGTAACTGTAGCTGAGTTCACATCTTTGTGTGAAGCGGAAGAAATGCTCTTAGCATTATGCTATAACTATTATAATCTGACGTTATACAGTTTCTTT
GAATTTGGGACTAATATTGAATACATGGAACATCTGTTGCTTCTTCTTGAAGAACAGCTTGCTCTCGACAAATACTATGGTTTTGAAAAGGTCTTGAATGTAGCTGTTGCAAATG
CTAAAAAAATGGGTTTCCACCGTTGGGAGTTTTACGTCGGTTATGAAGAGTCGACTGCTGAAAAGAGGCGGCTACTATGGTGGAAGTTATACAATTATGAAAAAGCCAGTACT
ATGAAGAAGGGTTTTTTTTCTGTGATTGATGATGCTACTGTCAACTGTTTATTACCTAAGATTTTTAGAAACTTTGGCTATCTGGATAGGGTGGAGTTTCTAGAAAATATTCAAA
AGCCAATGGATCTTAGTGTGTTTTCCGATGTTCCAATTTCTGTCCTTTGTAAATACGGTGAGTTGGCCCTTACAATAGTTACCAGTGAGTTTCATGAAAAATTTTTATATGCTGAT
AGATACACTTCTATTCGAAATTCCGCGAAACCGCCGACATTAAAAAACCAATTAATTAAGGAAATTGTGGATGGTATAGCTTATACAGAGACATCATATGAGGCAATCAGAAA
GCAAACTGCAAAACTATGGGATATTGCATTAGGTAAGGTGACCAAAGATAAAATCAATAAAGAAGATACAGCAGCAGCTAGCAAATTTACTTTGAGTTATGAATATCACAGAT
TCAGGCTAATCAATATGGCAGACAATTTAATTGCTAGACTTATGGTGAAACCAAAATCAGATTGGCTAATATCAGTCATGAAGGGGCATCTTAACAGACTATATGAGCACTGGA
AAGTAATGAATGAAATTATCCTAAGTATGGACAACGATTATTCAATTGCAACAACGTTCGAATATTATGCACCATCATGTCTGTGTTTAGCTACGCAGACTTTCCTTATTGTGAG
GAATATGGAAATGGATGATGTCAAGATGATGGTTGCAGTATATAAAAGATTTCTTAACCTAGGAATGTTTCTGCAGAGTGCCAAAGTATGCAGCCTTGCCGATAGTCATACATT
CAGAGATTTTTCTAGATCTTTTTCCTTTATTACGATAATTTCAAGATTGATGATAATCGAATTTATGCAAATTAAAGAATTGACGAAGGTAGAGTTTATTGAGAAGTTTTCTGAAG
TATGCCCTGACCTTGCAGATCTACCTCCGATGCTTCTAGATCCAAACTCTTGCTTATATTTTTCATTGTTACAGCAGATTAAGAAATCTGGTTTTACGTTGTCATTCAAAAAAATTC
TTGAAGACGCTAGAATGATGGACTTCAATTACGACCGCAATTTGGACTCAGAGGCCATTAAAAAGTGCAATGGTGAATTTAGCAAGTCAATGCCTTCCTGTACCAATGTCTCAG
ATACCACCACCGCTGTTTCTGACAACAGTGCTAAGAAGAAAGCTTCAATGGGGTCGGCGAGGGTAAATTCAACTGATACACTAACTGCATCTCCCTTATCGGGCTTAAGGAATC
AAACGCAGTTGGATTCTAAAGACAGTGTTCCATCTCTCGAGGCTTATACACCAATTGATTCTGTCTCTGACGTACCCACTGGGGAGATCAACGTTCCATTCCCTCCTGTTTATAAT
CAAAATGGATTGGATCAGCAAACCACTTATAATTTGGGAACTTTAGATGAGTTTGTTAACAAGGGAGATTTGAATGAACTCTATAATAGCCTATGGGGTGACCTATTTTCTGAT
GTTTACTTGTGA

>54

ATGCAAACATTAGAAACTACATCAAAATCAAATCCAGGGGAAGTCAAAGCACAGAAGCCTAGTACAAGAAGAACAAAAGTTGGAAAAGCTTGTGATAGCTGTAGAAGGAGG
AAAATAAAATGTAATGGGCTAAAACCTTGTCCATCTTGTACAATCTATGGTTGTGAATGTACATATACTGATGCAAAATCGACAAAAAATCTCAAATCAAATGATGCAGGTAAA
CCAAAACCAACAGGGAGAGTATCAAAGAATAAAGAAACTACTAGAATCGACAAAGATATTAGGAAATCAGAGCAGCAGTATGTCCCTATTAATGCTAATATTCATGTTGGTCC
CAGGTTCCCCTCCGAGAATATATTGAATGGATATCCACAATGTGGAGCACCACAGAACAATGTTGTGGGTAATCCACTAGCGGTTAATCCTCAATGCCATAGAGGTCTTTCTGA
AACTCCTATGTCCTCAACATTCAAAGAATCTAACTTAAGAGATGATCGGCTACTACAGTCATCAGATACAGATGATATGAGGAATGGTGACTCGGAAGAAAGGGACTTGAAAG
GGAGTGACAGCGAGAATGTCAAAAGTAAAGACAATAAAAGTGATCCTTTGATTATATACAAAGATGATACACATATTGAAAGCACGGTTAATAAACTAACACAGGCAGTTAAT
GAACTCAAATCACTTCAAAATGCACCTAGTTCGATAAAATCATCCATTGACGCCATTGAGTTACAACTTAGAAACATTTTAGACAATTGGAAACCAGAGGTAGATTTCGAGAAA
GCAAAGATTAATGAAAGTGCCACCACTAAGTCACTTGAAACAAACTTGCTGAGGAATAAATACACTAATCACGTTCATCTAACAAGATTTAGGATATGGATAGATTATAAAAAT
GCGAACAAAAACAATCATTTTATGGGAGAGTGTGGATTTAGTCTTGCAGAATCTTTTTTTGCTTCTAATCAGCCATTGGTCGATGAATTGTTTGGGTTGTATTCCCAGGTAGAGG
CCTTTTCTTTGCAAGGTCTTGGTTACTGTGTTCACCTTTATGAGCCATATATGAAAACTGAGGAAGCGATAAAACTGATGAAAGAGACCTTATATATTATACTACGGTTTATTGA
TATATGTGTTCACCATATCAATGAAGAGTCGATATCGATTGCCAACCCGTTAGAAACATATTTACGAAAAAAACATCTAATGCCTATGACTCCTACACCAAGGTCGTCCTATGGA
AGTCCACAAAGTGCTAGTACAAAGAGCTTGGTAAGTAAGATAATAGAGAGAATACCGCAACCGTTTATTGAGAGTGTAACTAATGTGTCGAGTCTTCAACTATTAGATCTTCGA
GATGACGAGTCAAAAATGTTTGGAACATTGCTGAACATGTGTAAGTCTATAAGGCGAAAATTTGACTCTGTTATGAGCGATTACGATTCCATTGTCACAGAAAAATCCGAAGG
CGAACAAAATGATGGTAAAGTAACTGTAGCTGAGTTCACATCTTTGTGTGAAGCGGAAGAAATGCTCTTAGCATTATGCTATAACTATTATAATCTGACGTTATACAGTTTCTTT
GAATTTGGGACTAATATTGAATACATGGAACATCTGTTGCTTCTTCTTGAAGAACAGCTTGCTCTCGACGAATACTATGGTTTTGAAAAGGTCTTGAATGTAGCTGTTGCAAATG
CTAAAAAAATGGGTTTCCACCGTTGGGAGTTTTACGTCGGTTATGAAGAGTCGACTGCTGAAAAGAGGCGGCTACTATGGTGGAAGTTATACAATTATGAAAAAGCCAGTACT
ATGAAGAAGGGTTTTTTTTCTGTGATTGATGATGCTACTGTCAACTGTTTATTACCTAAGATTTTTAGAAACTTTGGCTATCTGGATAGGGTGGAGTTTCTAGAAAATATTCAAA
AGCCAATGGATCTTAGTGTGTTTTCCGATGTTCCAATTTCTGTCCTTTGTAAATACGGTGAGTTGGCCCTTACAATAGTTACCAGTGAGTTTCATGAAAAATTTTTATATGCTGAT
AGATACACTTCTATTCGAAATTCCGCGAAACCGCCGACATTAAAAAACCAATTAATTAAGGAAATTGTGGATGGTATAGCTTATACAGAGACATCATATGAGGCAATCAGAAA
GCAAACTGCAAAACTATGGGATATTGCATTAGGTAAGGTGACCAAAGATAAAATCAATAAAGAAGATACAGCAGCAGCTAGCAAATTTACTTTGAGTTATGAATATCACAGAT
TCAGGCTAATCAATATGGCAGACAATTTAATTGCTAGACTTATGGTGAAACCAAAATCAGATTGGCTAATATCAGTCATGAAGGGGCATCTTAACAGACTATATGAGCACTGGA
AAGTAATGAATGAAATTATCCTAAGTATGGACAACGATTATTCAATTGCAACAACGTTCGAATATTATGCACCATCATGTCTGTGTTTAGCTACGCAGACTTTCCTTATTGTGAG
GAATATGGAAATGGATGATGTCAAGATGATGGTTGCAGTATATAAAAGATTTCTTAACCTAGGAATGTTTCTGCAGAGTGCCAAAGTATGCAGCCTTGCCGATAGTCATACATT
CAGAGATTTTTCTAGATCTTTTTCCTTTATTACGATAATTTCAAGATTGATGATAATCGAATTTATGCAAATTAAAGAATTGACGAAGGTAGAGTTTATTGAGAAGTTTTCTGAAG
TATGCCCTGACCTTGCAGATCTACCTCCGATGCTTCTAGATCCAAACTCTTGCTTATATTTTTCATTGTTACAGCAGATTAAGAAATCTGGTTTTACGTTGTCATTCAAAAAAAATTC
TTGAAGACGCTAGAATGATGGACTTCAATTACGACCGCAATTTGGACTCAGAGGCCATTAAAAAGTGCAATGGTGAATTTAGCAAGTCAATGCCTTCCTGTACCAATGTCTCAG
ATACCACCACCGCTGTTTCTGACAACAGTGCTAAGAAGAAAGCTTCAATGGGGTCGGCGAGGGTAAATTCAACTGATACACTAACTGCATCTCCCTTATCGGGCTTAAGGAATC
AAACGCAGTTGGATTCTAAAGACAGTGTTCCATCTCTCGAGGCTTATACACCAATTGATTCTGTCTCTGACGTACCCACTGGGGAGATCAACGTTCCATTCCCTCCTGTTTATAAT
CAAAATGGATTGGATCAGCAAACCACTTATAATTTGGGAACTTTAGATGAGTTTGTTAACAAGGGAGATTTGAATGAACTCTATAATAGCCTATGGGGTGACCTATTTTCTGAT
GTTTACTTGTGA

>55

ATGCAAACATTAGAAACTACATCAAAATCAAATCCAGGGGAAGTCAAAGCACAGAAGCCTAGTACAAGAAGAACAAAAGTTGGAAAAGCTTGTGATAGCTGTAGAAGGAGG
AAAATAAAATGTAATGGGCTAAAACCTTGTCCATCTTGTACAATCTATGGTTGTGAATGTACATATACTGATGCAAAATCGACAAAAAATCTCAAATCAAATGATGCAGGTAAC
CCAAAACCAACAGGGAGAGTATCAAAGAATAAAGAAACTACTAGAATCGACAAAGATATTAGGAAATCAGAGCAGCAGTATGTCCCTATTAATGCTAATATTCATGTTGGTCC
CAGGTTCCCCTCCGAGAATATATTGAATGGATATCCACAATGTGGAGCACCACAGAACAATGTTGTGGGTAATCCACTAGCGGTTAATCCTCAATGCCATAGAGGTCTTTCTGA
AACTCCTATGTCCTCAACATTCAAAGAATCTAACTTGAGAGATGATCGGCTACTACAGTCATCAGATACAGATGATATGAGGAATGGTGACTCGGAAGAAAGGGACTTGAAAG
GGAGTGACAGCGAGAATGTCAAAAGTAAAGACAATAAAAGTGATCCTTTGATTATATACAAAGATGATACACATATTGAAAGCACGGTTAATAAACTAACACAGGCAGTTAAT
GAACTCAAATCACTTCAAAATGCACCTAGTTCGATAAAATCATCCATTGACGCCATTGAGTTACAACTTAGAAACATTTTAGACAATTGGAAACCAGAGGTAGATTTCGAGAAA
GCAAAGATTAATGAAAGTGCCACCACTAAGTCACTTGAAACAAACTTGCTGAGGAATAAATACACTAATCACGTTCATCTAACAAGATTTAGGATATGGATAGATTATAAAAAT
GCGAACAAAAACAATCATTTTATGGGAGAGTGTGGATTTAGTCTTGCAGAATCTTTTTTTGCTTCTAATCAGCCATTGGTCGATGAATTGTTTGGGTTGTATTCCCAGGTAGAGG
CCTTTTCTTTGCAAGGTCTTGGTTACTGTGTTCACCTTTATGAGCCATATATGAAAACTGAGGAAGCGATAAAACTGATGAAAGAGACCTTATATATTATACTACGGTTTATTGA
TATATGTGTTCACCATATCAATGAAGAGTCGATATCGATTGCCAACCCGTTAGAAACATATTTACGAAAAAAACATCTAATGCCTATGACTCCTACACCAAGGTCGTCCTATGGA
AGTCCACAAAGTGCTAGTACAAAGAGCTTGGTAAGTAAGATAATAGAGAGAATACCGCAACCGTTTATTGAGAGTGTAACTAATGTGTCGAGTCTTCAACTATTAGATCTTCGA
GATGACGAGTCAAAAATGTTTGGAACATTGCTGAACATGTGTAAGTCTATAAGGCGAAAATTTGACTCTGTTATGAGCGATTACGATTCCATTGTCACAGAAAAATCCGAAGG
CGAACAAAATGATGGTAAAGTAACTGTAGCTGAGTTCACATCTTTGTGTGAAGCGGAAGAAATGCTCTTAGCATTATGCTATAACTATTATAATCTGACGTTATACAGTTTCTTT
GAATTTGGGACTAATATTGAATACATGGAACATCTGTTGCTTCTTCTTGAAGAACAGCTTGCTCTCGACGAATACTATGGTTTTGAAAAGGTCTTGAATGTAGCTGTTGCAAATG
CTAAAAAAATGGGTTTCCACCGTTGGGAGTTTTACGTCGGTTATGAAGAGTCGACTGCTGAAAAGAGGCGGCTACTATGGTGGAAGTTATACAATTATGAAAAAGCCAGTACT
ATGAAGAAGGGTTTTTTTTCTGTGATTGATGATGCTACTGTCAACTGTTTATTACCTAAGATTTTTAGAAACTTTGGCTATCTGGATAGGGTGGAGTTTCTAGAAAATATTCAAA
AGCCAATGGATCTTAGTGTGTTTTCCGATGTTCCAATTTCTGTCCTTTGTAAATACGGTGAGTTGGCCCTTACAATAGTTACCAGTGAGTTTCATGAAAAATTTTTATATGCTGAT
AGATACACTTCTATTCGAAATTCCGCGAAACCGCCGACATTAAAAAACCAATTAATTAAGGAAATTGTGGATGGTATAGCTTATACAGAGACATCATATGAGGCAATCAGAAA
GCAAACTGCAAAACTATGGGATATTGCATTAGGTAAGGTGACCAAAGATAAAATCAATAAAGAAGATACAGCAGCAGCTAGCAAATTTACTTTGAGTTATGAATATCACAGAT
TCAGGCTAATCAATATGGCAGACAATTTAATTGCTAGACTTATGGTGAAACCAAAATCAGATTGGCTAATATCAGTCATGAAGGGGCATCTTAACAGACTATATGAGCACTGGA
AAGTAATGAATGAAATTATCCTAAGTATGGACAACGATTATTCAATTGCAACAACGTTCGAATATTATGCACCATCATGTCTGTGTTTAGCTACGCAGACTTTCCTTATTGTGAG
GAATATGGAAATGGATGATGTCAAGATGATGGTTGCAGTATATAAAAGATTTCTTAACCTAGGAATGTTTCTGCAGAGTGCCAAAGTATGCAGCCTTGCCGATAGTCATACATT
CAGAGATTTTTCTAGATCTTTTTCCTTTATTACGATAATTTCAAGATTGATGATAATCGAATTTATGCAAATTAAAGAATTGACGAAGGTAGAGTTTATTGAGAAGTTTTCTGAAG
TATGCCCTGACCTTGCAGATCTACCTCCGATGCTTCTAGATCCAAACTCTTGCTTATATTTTTCATTGTTACAGCAGATTAAGAAATCTGGTTTTACGTTGTCATTCAAAAAAAATTC
TTGAAGACGCTAGAATGATGGACTTCAATTACGACCGCAATTTGGACTCAGAGGCCATTAAAAAGTGCAATGGTGAATTTAGCAAGTCAATGCCTTCCTGTACCAATGTCTCAG
ATACCACCACCGCTGTTTCTGACAACAGTGCTAAGAAGAAAGCTTCAATGGGGTCGGCGAGGGTAAATTCAACTGATACACTAACTGCATCTCCCTTATCGGGCTTAAGGAATC
AAACGCAGTTGGATTCTAAAGACAGTGTTCCATCTCTCGAGGCTTATACACCAATTGATTCTGTCTCTGACGTACCCACTGGGGAGATCAACGTTCCATTCCCTCCTGTTTATAAT

CAAAATGGATTGGATCAGCAAACCACTTATAATTTGGGAACTTTAGATGAGTTTGTTAACAAGGGAGATTTGAATGAACTCTATAATAGCCTATGGGGTGACCTATTTTCTGAT
GTTTACTTGTGA

>56

ATGCAAACATTAGAAACTACATCAAAATCAAATCCAGGGGAAGTCAAAGCACAGAAGCCTAGTACAAGAAGAACAAAAGTTGGAAAAGCTTGTGATAGCTGTAGAAGGAGG
AAAATAAAATGTAATGGGCTAAAACCTTGTCCATCTTGTACAATCTATGGTTGTGAATGTACATATACTGATGCAAAATCGACAAAAAATCTCAAATCAAATGATGCAGGTAAA
CCAAAACCAACAGGGAGAGTATCAAAGAATAAAGAAACTACTAGAATCGACAAAGATATTAGGAAATCAGAGCAGCAGTATGTCCCTATTAATGCTAATATTCATGTTGGTCC
CAGGTTCCCCTCCGAGAATATATTGAATGGATATCCACAATGTGGAGCACCACAGAACAATGTTGTGGGTAATCCACTAGCGGTTAATCCTCAATGCCATAGAGGTCTTTCTGA
AACTCCTATGTCCTCAACATTCAAAGAATCTAACTTAAGAGATGATCGGCTACTACAGTCATCAGATACAGATGATATGAGGAATGGTGACTCGGAAGAAAGGGACTTGAAAG
GGAGTGACAGCGAGAATGTCAAAAGTAAAGACAATAAAAGTGATCCTTTGATTATATACAAAGATGATACACATATTGAAAGCACGGTTAATAAACTAACACAGGCAGTTAAT
GAACTCAAATCACTTCAAAATGCACCTAGTTCGATAAAATCATCCATTGACGCCATTGAGTTACAACTTAGAAACATTTTAGACAATTGGAAACCAGAGGTAGATTTCGAGAAA
GCAAAGATTAATGAAAGTGCCACCACTAAGTCACTTGAAACAAACTTGCTGAGGAATAAATACACTAATCACGTTCATCTAACAAGATTTAGGATATGGATAGATTATAAAAAT
GCGAACAAAAACAATCATTTTATGGGAGAGTGTGGATTTAGTCTTGCAGAATCTTTTTTTGCTTCTAATCAGCCATTGGTCGATGAATTGTTTGGGTTGTATTCCCAGGTAGAGG
CCTTTTCTTTGCAAGGTCTTGGTTACTGTGTTCACCTTTATGAGCCATATATGAAAACTGAGGAAGCGATAAAACTGATGAAAGAGACCTTATATATTATACTACGGTTTATTGA
TATATGTGTTCACCATATCAATGAAGAGTCGATATCGATTGCCAACCCGTTAGAAACATATTTACGAAAAAAACATCTAATGCCTATGACTCCTACACCAAGGTCGTCCTATGGA
AGTCCACAAAGTGCTAGTACAAAGAGCTTGGTAAGTAAGATAATAGAGAGAATACCGCAACCGTTTATTGAGAGTGTAACTAATGTGTCGAGTCTTCAACTATTAGATCTTCGA
GATGACGAGTCAAAAATGTTTGGAACATTGCTGAACATGTGTAAGTCTATAAGGCGAAAATTTGACTCTGTTATGAGCGATTACGATTCCATTGTCACAGAAAAATCCGAAGG
CGAACAAAATGATGGTAAAGTAACTGTAGCTGAGTTCACATCTTTGTGTGAAGCGGAAGAAATGCTCTTAGCATTATGCTATAACTATTATAATCTGACGTTATACAGTTTCTTT
GAATTTGGGACTAATATTGAATACATGGAACATCTGTTGCTTCTTCTTGAAGAACAGCTTGCTCTCGACGAATACTATGGTTTTGAAAAGGTCTTGAATGTAGCTGTTGCAAATG
CTAAAAAAATGGGTTTCCACCGTTGGGAGTTTTACGTCGGTTATGAAGAGTCGACTGCTGAAAAGAGGCGGCTACTATGGTGGAAGTTATACAATTATGAAAAAGCCAGTACT
ATGAAGAAGGGTTTTTTTTCTGTGATTGATGATGCTACTGTCAACTGTTTATTACCTAAGATTTTTAGAAACTTTGGCTATCTGGATAGGGTGGAGTTTCTAGAAAATATTCAAA
AGCCAATGGATCTTAGTGTGTTTTCCGATGTTCCAATTTCTGTCCTTTGTAAATACGGTGAGTTGGCCCTTACAATAGTTACCAGTGAGTTTCATGAAAAATTTTTATATGCTGAT
AGATACACTTCTATTCGAAATTCCGCGAAACCGCCGACATTAAAAAACCAATTAATTAAGGAAATTGTGGATGGTATAGCTTATACAGAGACATCATATGAGGCAATCAGAAA
GCAAACTGCAAAACTATGGGATATTGCATTAGGTAAGGTGACCAAAGATAAAATCAATAAAGAAGATACAGCAGCAGCTAGCAAATTTACTTTGAGTTATGAATATCACAGAT
TCAGGCTAATCAATATGGCAGACAATTTAATTGCTAGACTTATGGTGAAACCAAAATCAGATTGGCTAATATCAGTCATGAAGGGGCATCTTAACAGACTATATGAGCACTGGA
AAGTAATGAATGAAATTATCCTAAGTATGGACAACGATTATTCAATTGCAACAACGTTCGAATATTATGCACCATCATGTCTGTGTTTAGCTACGCAGACTTTCCTTATTGTGAG
GAATATGGAAATGGATGATGTCAAGATGATGGTTGCAGTATATAAAAGATTTCTTAACCTAGGAATGTTTCTGCAGAGTGCCAAAGTATGCAGCCTTGCCGATAGTCATACATT
CAGAGATTTTTCTAGATCTTTTTCCTTTATTACGATAATTTCAAGATTGATGATAATCGAATTTATGCAAATTAAAGAATTGACGAAGGTAGAGTTTATTGAGAAGTTTTCTGAAG
TATGCCCTGACCTTGCAGATCTACCTCCGATGCTTCTAGATCCAAACTCTTGCTTATATTTTTCATTGTTACAGCAGATTAAGAAATCTGGTTTTACGTTGTCATTCAAAAAAAATTC
TTGAAGACGCTAGAATGATGGACTTCAATTACGACCGCAATTTGGACTCAGAGGCCATTAAAAAGTGCAATGGTGAATTTAGCAAGTCAATGCCTTCCTGTACCAATGTCTCAG
ATACCACCACCGCTGTTTCTGACAACAGTGCTAAGAAGAAAGCTTCAATGGGGTCGGCGAGGGTAAATTCAACTGATACACTAACTGCATCTCCCTTATCGGGCTTAAGGAATC

AAACGCAGTTGGATTCTAAAGACAGTGTTCCATCTCTCGAGGCTTATACACCAATTGATTCTGTCTCTGACGTACCCACTGGGGAGATCAACGTTCCATTCCCTCCTGTTTATAAT
CAAAATGGATTGGATCAGCAAACCACTTATAATTTGGGAACTTTAGATGAGTTTGTTAACAAGGGAGATTTGAATGAACTCTATAATAGCCTATGGGGTGACCTATTTTCTGAT
GTTTACTTGTGA

>57

ATGCAAACATTAGAAACTACATCAAAATCAAATCCAGGGGAAGTCAAAGCACAGAAGCCTAGTACAAGAAGAACAAAAGTTGGAAAAGCTTGTGATAGCTGTAGAAGGAGG
AAAATAAAATGTAATGGGCTAAAACCTTGTCCATCTTGTACAATCTATGGTTGTGAATGTACATATACTGATGCAAAATCGACAAAAAATCTCAAATCAAATGATGCAGGTAAA
CCAAAACCAACAGGGAGAGTATCAAAGAATAAAGAAACTACTAGAATCGACAAAGATATTAGGAAATCAGAGCAGCAGTATGTCCCTATTAATGCTAATATTCATGTTGGTCC
CAGGTTCCCCTCCGAGAATATATTGAATGGATATCCACAATGTGGAGCACCACAGAACAATGTTGTGGGTAATCCACTAGCGGTTAATCCTCAATGCCATAGAGGTCTTTCTGA
AACTCCTATGTCCTCAACATTCAAAGAATCTAACTTAAGAGATGATCGGCTACTACAGTCATCAGATACAGATGATATGAGGAATGGTGACTCGGAAGAAAGGGACTTGAAAG
GGAGTGACAGCGAGAATGTCAAAAGTAAAGACAATAAAAGTGATCCTTTGATTATATACAAAGATGATACACATATTGAAAGCACGGTTAATAAACTAACACAGGCAGTTAAT
GAACTCAAATCACTTCAAAATGCACCTAGTTCGATAAAATCATCCATTGACGCCATTGAGTTACAACTTAGAAACATTTTAGACAATTGGAAACCAGAGGTAGATTTCGAGAAA
GCAAAGATTAATGAAAGTGCCACCACTAAGTCACTTGAAACAAACTTGCTGAGGAATAAATACACTAATCACGTTCATCTAACAAGATTTAGGATATGGATAGATTATAAAAAT
GCGAACAAAAACAATCATTTTATGGGAGAGTGTGGATTTAGTCTTGCAGAATCTTTTTTTGCTTCTAATCAGCCATTGGTCGATGAATTGTTTGGGTTGTATTCCCAGGTAGAGG
CCTTTTCTTTGCAAGGTCTTGGTTACTGTGTTCACCTTTATGAGCCATATATGAAAACTGAGGAAGCGATAAAACTGATGAAAGAGACCTTATATATTATACTACGGTTTATTGA
TATATGTGTTCACCATATCAATGAAGAGTCGATATCGATTGCCAACCCGTTAGAAACATATTTACGAAAAAAACATCTAATGCCTATGACTCCTACACCAAGGTCGTCCTATGGA
AGTCCACAAAGTGCTAGTACAAAGAGCTTGGTAAGTAAGATAATAGAGAGAATACCGCAACCGTTTATTGAGAGTGTAACTAATGTGTCGAGTCTTCAACTATTAGATCTTCGA
GATGACGAGTCAAAAATGTTTGGAACATTGCTGAACATGTGTAAGTCTATAAGGCGAAAATTTGACTCTGTTATGAGCGATTACGATTCCATTGTCACAGAAAAATCCGAAGG
CGAACAAAATGATGGTAAAGTAACTGTAGCTGAGTTCACATCTTTGTGTGAAGCGGAAGAAATGCTCTTAGCATTATGCTATAACTATTATAATCTGACGTTATACAGTTTCTTT
GAATTTGGGACTAATATTGAATACATGGAACATCTGTTGCTTCTTCTTGAAGAACAGCTTGCTCTCGACGAATACTATGGTTTTGAAAAGGTCTTGAATGTAGCTGTTGCAAATG
CTAAAAAAATGGGTTTCCACCGTTGGGAGTTTTACGTCGGTTATGAAGAGTCGACTGCTGAAAAGAGGCGGCTACTATGGTGGAAGTTATACAATTATGAAAAAGCCAGTACT
ATGAAGAAGGGTTTTTTTTCTGTGATTGATGATGCTACTGTCAACTGTTTATTACCTAAGATTTTTAGAAACTTTGGCTATCTGGATAGGGTGGAGTTTCTAGAAAATATTCAAA
AGCCAATGGATCTTAGTGTGTTTTCCGATGTTCCAATTTCTGTCCTTTGTAAATACGGTGAGTTGGCCCTTACAATAGTTACCAGTGAGTTTCATGAAAAATTTTTATATGCTGAT
AGATACACTTCTATTCGAAATTCCGCGAAACCGCCGACATTAAAAAACCAATTAATTAAGGAAATTGTGGATGGTATAGCTTATACAGAGACATCATATGAGGCAATCAGAAA
GCAAACTGCAAAACTATGGGATATTGCATTAGGTAAGGTGACCAAAGATAAAATCAATAAAGAAGATACAGCAGCAGCTAGCAAATTTACTTTGAGTTATGAATATCACAGAT
TCAGGCTAATCAATATGGCAGACAATTTAATTGCTAGACTTATGGTGAAACCAAAATCAGATTGGCTAATATCAGTCATGAAGGGGCATCTTAACAGACTATATGAGCACTGGA
AAGTAATGAATGAAATTATCCTAAGTATGGACAACGATTATTCAATTGCAACAACGTTCGAATATTATGCACCATCATGTCTGTGTTTAGCTACGCAGACTTTCCTTATTGTGAG
GAATATGGAAATGGATGATGTCAAGATGATGGTTGCAGTATATAAAAGATTTCTTAACCTAGGAATGTTTCTGCAGAGTGCCAAAGTATGCAGCCTTGCCGATAGTCATACATT
CAGAGATTTTTCTAGATCTTTTTCCTTTATTACGATAATTTCAAGATTGATGATAATCGAATTTATGCAAATTAAAGAATTGACGAAGGTAGAGTTTATTGAGAAGTTTTCTGAAG
TATGCCCTGACCTTGCAGATCTACCTCCGATGCTTCTAGATCCAAACTCTTGCTTATATTTTTCATTGTTACAGCAGATTAAGAAATCTGGTTTTACGTTGTCATTCAAAAAAATTC
TTGAAGACGCTAGAATGATGGACTTCAATTACGACCGCAATTTGGACTCAGAGGCCATTAAAAAGTGCAATGGTGAATTTAGCAAGTCAATGCCTTCCTGTACCAATGTCTCAG

ATACCACCACCGCTGTTTCTGACAACAGTGCTAAGAAGAAAGCTTCAATGGGGTCGGCGAGGGTAAATTCAACTGATACACTAACTGCATCTCCCTTATCGGGCTTAAGGAATC
AAACGCAGTTGGATTCTAAAGACAGTGTTCCATCTCTCGAGGCTTATACACCAATTGATTCTGTCTCTGACGTACCCACTGGGGAGATCAACGTTCCATTCCCTCCTGTTTATAAT
CAAAATGGATTGGATCAGCAAACCACTTATAATTTGGGAACTTTAGATGAGTTTGTTAACAAGGGAGATTTGAATGAACTCTATAATAGCCTATGGGGTGACCTATTTTCTGAT
GTTTACTTGTGA

>58

ATGCAAACATTAGAAACTACATCAAAATCAAATCCAGGGGAAGTCAAAGCACAGAAGCCTAGTACAAGAAGAACAAAAGTTGGAAAAGCTTGTGATAGCTGTAGAAGGAGG
AAAATAAAATGTAATGGGCTAAAACCTTGTCCATCTTGTACAATCTATGGTTGTGAATGTACATATACTGATGCAAAATCGACAAAAAATCTCAAATCAAATGATGCAGGTAAA
CCAAAACCAACAGGGAGAGTATCAAAGAATAAAGAAACTACTAGAATCGACAAAGATATTAGGAAATCAGAGCAGCAGTATGTCCCTATTAATGCTAATATTCATGTTGGTCC
CAGGTTCCCCTCCGAGAATATATTGAATGGATATCCACAATGTGGAGCACCACAGAACAATGTTGTGGGTAATCCACTAGCGGTTAATCCTCAATGCCATAGAGGTCTTTCTGA
AACTCCTATGTCCTCAACATTCAAAGAATCTAACTTAAGAGATGATCGGCTACTACAGTCATCAGATACAGATGATATGAGGAATGGTGACTCGGAAGAAAGGGACTTGAAAG
GGAGTGACAGCGAGAATGTCAAAAGTAAAGACAATAAAAGTGATCCTTTGATTATATACAAAGATGATACACATATTGAAAGCACGGTTAATAAACTAACACAGGCAGTTAAT
GAACTCAAATCACTTCAAAATGCACCTAGTTCGATAAAATCATCCATTGACGCCATTGAGTTACAACTTAGAAACATTTTAGACAATTGGAAACCAGAGGTAGATTTCGAGAAA
GCAAAGATTAATGAAAGTGCCACCACTAAGTCACTTGAAACAAACTTGCTGAGGAATAAATACACTAATCACGTTCATCTAACAAGATTTAGGATATGGATAGATTATAAAAAT
GCGAACAAAAACAATCATTTTATGGGAGAGTGTGGATTTAGTCTTGCAGAATCTTTTTTTGCTTCTAATCAGCCATTGGTCGATGAATTGTTTGGGTTGTATTCCCAGGTAGAGG
CCTTTTCTTTGCAAGGTCTTGGTTACTGTGTTCACCTTTATGAGCCATATATGAAAACTGAGGAAGCGATAAAACTGATGAAAGAGACCTTATATATTATACTACGGTTTATTGA
TATATGTGTTCACCATATCAATGAAGAGTCGATATCGATTGCCAACCCGTTAGAAACATATTTACGAAAAAAACATCTAATGCCTATGACTCCTACACCAAGGTCGTCCTATGGA
AGTCCACAAAGTGCTAGTACAAAGAGCTTGGTAAGTAAGATAATAGAGAGAATACCGCAACCGTTTATTGAGAGTGTAACTAATGTGTCGAGTCTTCAACTATTAGATCTTCGA
GATGACGAGTCAAAAATGTTTGGAACATTGCTGAACATGTGTAAGTCTATAAGGCGAAAATTTGACTCTGTTATGAGCGATTACGATTCCATTGTCACAGAAAAATCCGAAGG
CGAACAAAATGATGGTAAAGTAACTGTAGCTGAGTTCACATCTTTGTGTGAAGCGGAAGAAATGCTCTTAGCATTATGCTATAACTATTATAATCTGACGTTATACAGTTTCTTT
GAATTTGGGACTAATATTGAATACATGGAACATCTGTTGCTTCTTCTTGAAGAACAGCTTGCTCTCGACGAATACTATGGTTTTGAAAAGGTCTTGAATGTAGCTGTTGCAAATG
CTAAAAAAATGGGTTTCCACCGTTGGGAGTTTTACGTCGGTTATGAAGAGTCGACTGCTGAAAAGAGGCGGCTACTATGGTGGAAGTTATACAATTATGAAAAAGCCAGTACT
ATGAAGAAGGGTTTTTTTTCTGTGATTGATGATGCTACTGTCAACTGTTTATTACCTAAGATTTTTAGAAACTTTGGCTATCTGGATAGGGTGGAGTTTCTAGAAAATATTCAAA
AGCCAATGGATCTTAGTGTGTTTTCCGATGTTCCAATTTCTGTCCTTTGTAAATACGGTGAGTTGGCCCTTACAATAGTTACCAGTGAGTTTCATGAAAAATTTTTATATGCTGAT
AGATACACTTCTATTCGAAATTCCGCGAAACCGCCGACATTAAAAAACCAATTAATTAAGGAAATTGTGGATGGTATAGCTTATACAGAGACATCATATGAGGCAATCAGAAA
GCAAACTGCAAAACTATGGGATATTGCATTAGGTAAGGTGACCAAAGATAAAATCAATAAAGAAGATACAGCAGCAGCTAGCAAATTTACTTTGAGTTATGAATATCACAGAT
TCAGGCTAATCAATATGGCAGACAATTTAATTGCTAGACTTATGGTGAAACCAAAATCAGATTGGCTAATATCAGTCATGAAGGGGCATCTTAACAGACTATATGAGCACTGGA
AAGTAATGAATGAAATTATCCTAAGTATGGACAACGATTATTCAATTGCAACAACGTTCGAATATTATGCACCATCATGTCTGTGTTTAGCTACGCAGACTTTCCTTATTGTGAG
GAATATGGAAATGGATGATGTCAAGATGATGGTTGCAGTATATAAAAGATTTCTTAACCTAGGAATGTTTCTGCAGAGTGCCAAAGTATGCAGCCTTGCCGATAGTCATACATT
CAGAGATTTTTCTAGATCTTTTTCCTTTATTACGATAATTTCAAGATTGATGATAATCGAATTTATGCAAATTAAAGAATTGACGAAGGTAGAGTTTATTGAGAAGTTTTCTGAAG
TATGCCCTGACCTTGCAGATCTACCTCCGATGCTTCTAGATCCAAACTCTTGCTTATATTTTTCATTGTTACAGCAGATTAAGAAATCTGGTTTTACGTTGTCATTCAAAAAAAATTC

TTGAAGACGCTAGAATGATGGACTTCAATTACGACCGCAATTTGGACTCAGAGGCCATTAAAAAGTGCAATGGTGAATTTAGCAAGTCAATGCCTTCCTGTACCAATGTCTCAG
ATACCACCACCGCTGTTTCTGACAACAGTGCTAAGAAGAAAGCTTCAATGGGGTCGGCGAGGGTAAATTCAACTGATACACTAACTGCATCTCCCTTATCGGGCTTAAGGAATC
AAACGCAGTTGGATTCTAAAGACAGTGTTCCATCTCTCGAGGCTTATACACCAATTGATTCTGTCTCTGACGTACCCACTGGGGAGATCAACGTTCCATTCCCTCCTGTTTATAAT
CAAAATGGATTGGATCAGCAAACCACTTATAATTTGGGAACTTTAGATGAGTTTGTTAACAAGGGAGATTTGAATGAACTCTATAATAGCCTATGGGGTGACCTATTTTCTGAT
GTTTACTTGTGA

>71

ATGCAAACATTAGAAACTACATCAAAATCAAATCCAGGGGAAGTCAAAGCACAGAAGCCTAGTACAAGAAGAACAAAAGTTGGAAAAGCTTGTGATAGCTGTAGAAGGAGG
AAAATAAAATGTAATGGGCTAAAACCTTGTCCATCTTGTACAATCTATGGTTGTGAATGTACATATACTGATGCAAAATCGACAAAAAATCTCAAATCAAATGATGCAGGTAAA
CCAAAACCAACAGGGAGAGTATCAAAGAATAAAGAAACTACTAGAATCGACAAAGATATTAGGAAATCAGAGCAGCAGTATGTCCCTATTAATGCTAATATTCATGTTGGTCC
CAGGTTCCCCTCCGAGAATATATTGAATGGATATCCACAATGTGGAGCACCACAGAACAATGTTGTGGGTAATCCACTAGCGGTTAATCCTCAATGCCATAGAGGTCTTTCTGA
AACTCCTATGTCCTCAACATTCAAAGAATCTAACTTGAGAGATGATCGGCTACTACAGTCATCAGATACAGATGATATGAGGAATGGTGACTCGGAAGAAAGGGACTTGAAAG
GGAGTGACAGCGAGAATGTCAAAAGTAAAGACAATAAAAGTGATCCTTTGATTATATACAAAGATGATACACATATTGAAAGCACGGTTAATAAACTAACACAGGCAGTTAAT
GAACTCAAATCACTTCAAAATGCACCTAGTTCGATAAAATCATCCATTGACGCCATTGAGTTACAACTTAGAAACATTTTAGACAATTGGAAACCAGAGGTAGATTTCGAGAAA
GCAAAGATTAATGAAAGTGCCACCACTAAGTCACTTGAAACAAACTTGCTGAGGAATAAATACACTAATCACGTTCATCTAACAAGATTTAGGATATGGATAGATTATAAAAAT
GCGAACAAAAACAATCATTTTATGGGAGAGTGTGGATTTAGTCTTGCAGAATCTTTTTTTGCTTCTAATCAGCCATTGGTCGATGAATTGTTTGGGTTGTATTCCCAGGTAGAGG
CCTTTTCTTTGCAAGGTCTTGGTTACTGTGTTCACCTTTATGAGCCATATATGAAAACTGAGGAAGCGATAAAACTGATGAAAGAGACCTTATATATTATACTACGGTTTATTGA
TATATGTGTTCACCATATCAATGAAGAGTCGATATCGATTGCCAACCCGTTAGAAACATATTTACGAAAAAAACATCTAATGCCTATGACTCCTACACCAAGGTCGTCCTATGGA
AGTCCACAAAGTGCTAGTACAAAGAGCTTGGTAAGTAAGATAATAGAGAGAATACCGCAACCGTTTATTGAGAGTGTAACTAATGTGTCGAGTCTTCAACTATTAGATCTTCGA
GATGACGAGTCAAAAATGTTTGGAACATTGCTGAACATGTGTAAGTCTATAAGGCGAAAATTTGACTCTGTTATGAGCGATTACGATTCCATTGTCACAGAAAAATCCGAAGG
CGAACAAAATGATGGTAAAGTAACTGTAGCTGAGTTCACATCTTTGTGTGAAGCGGAAGAAATGCTCTTAGCATTATGCTATAACTATTATAATCTGACGTTATACAGTTTCTTT
GAATTTGGGACTAATATTGAATACATGGAACATCTGTTGCTTCTTCTTGAAGAACAGCTTGCTCTCGACGAATACTATGGTTTTGAAAAGGTCTTGAATGTAGCTGTTGCAAATG
CTAAAAAAATGGGTTTCCACCGTTGGGAGTTTTACGTCGGTTATGAAGAGTCGACTGCTGAAAAGAGGCGGCTACTATGGTGGAAGTTATACAATTATGAAAAAGCCAGTACT
ATGAAGAAGGGTTTTTTTTCTGTGATTGATGATGCTACTGTCAACTGTTTATTACCTAAGATTTTTAGAAACTTTGGCTATCTGGATAGGGTGGAGTTTCTAGAAAATATTCAAA
AGCCAATGGATCTTAGTGTGTTTTCCGATGTTCCAATTTCTGTCCTTTGTAAATACGGTGAGTTGGCCCTTACAATAGTTACCAGTGAGTTTCATGAAAAATTTTTATATGCTGAT
AGATACACTTCTATTCGAAATTCCGCGAAACCGCCGACATTAAAAAACCAATTAATTAAGGAAATTGTGGATGGTATAGCTTATACAGAGACATCATATGAGGCAATCAGAAA
GCAAACTGCAAAACTATGGGATATTGCATTAGGTAAGGTGACCAAAGATAAAATCAATAAAGAAGATACAGCAGCAGCTAGCAAATTTACTTTGAGTTATGAATATCACAGAT
TCAGGCTAATCAATATGGCAGACAATTTAATTGCTAGACTTATGGTGAAACCAAAATCAGATTGGCTAATATCAGTCATGAAGGGGCATCTTAACAGACTATATGAGCACTGGA
AAGTAATGAATGAAATTATCCTAAGTATGGACAACGATTATTCAATTGCAACAACGTTCGAATATTATGCACCATCATGTCTGTGTTTAGCTACGCAGACTTTCCTTATTGTGAG
GAATATGGAAATGGATGATGTCAAGATGATGGTTGCAGTATATAAAAGATTTCTTAACCTAGGAATGTTTCTGCAGAGTGCCAAAGTATGCAGCCTTGCCGATAGTCATACATT
CAGAGATTTTTCTAGATCTTTTTCCTTTATTACGATAATTTCAAGATTGATGATAATCGAATTTATGCAAATTAAAGAATTGACGAAGGTAGAGTTTATTGAGAAGTTTTCTGAAG

TATGCCCTGACCTTGCAGATCTACCTCCGATGCTTCTAGATCCAAACTCTTGCTTATATTTTTCATTGTTACAGCAGATTAAGAAATCTGGTTTTACGTTGTCATTCAAAAAAATTC
TTGAAGACGCTAGAATGATGGACTTCAATTACGACCGCAATTTGGACTCAGAGGCCATTAAAAAGTGCAATGGTGAATTTAGCAAGTCAATGCCTTCCTGTACCAATGTCTCAG
ATACCACCACCGCTGTTTCTGACAACAGTGCTAAGAAGAAAGCTTCAATGGGGTCGGCGAGGGTAAATTCAACTGATACACTAACTGCATCTCCCTTATCGGGCTTAAGGAATC
AAACGCAGTTGGATTCTAAAGACAGTGTTCCATCTCTCGAGGCTTATACACCAATTGATTCTGTCTCTGACGTACCCACTGGGGAGATCAACGTTCCATTCCCTCCTGTTTATAAT
CAAAATGGATTGGATCAGCAAACCACTTATAATTTGGGAACTTTAGATGAGTTTGTTAACAAGGGAGATTTGAATGAACTCTATAATAGCCTATGGGGTGACCTATTTTCTGAT
GTTTACTTGTGA

>72

ATGCAAACATTAGAAACTACATCAAAATCAAATCCAGGGGAAGTCAAAGCACAGAAGCCTAGTACAAGAAGAACAAAAGTTGGAAAAGCTTGTGATAGCTGTAGAAGGAGG
AAAATAAAATGTAATGGGCTAAAACCTTGTCCATCTTGTACAATCTATGGTTGTGAATGTACATATACTGATGCAAAATCGACAAAAAATCTCAAATCAAATGATGCAGGTAAR
CCAAAACCAACAGGGAGAGTATCAAAGAATAAAGAAACTACTAGAATCGACAAAGATATTAGGAAATCAGAGCAGCAGTATGTCCCTATTAATGCTAATATTCATGTTGGTCC
CAGGTTCCCCTCCGAGAATATATTGAATGGATATCCACAATGTGGAGCACCACAGAACAATGTTGTGGGTAATCCACTAGCGGTTAATCCTCAATGCCATAGAGGTCTTTCTGA
AACTCCTATGTCCTCAACATTCAAAGAATCTAACTTAAGAGATGATCGGCTACTACAGTCATCAGATACAGATGATATGAGGAATGGTGACTCGGAAGAAAGGGACTTGAAAG
TGAGTGACAGCGAGAATGTCAAAAGTAAAGACAATAAAAGTGATCCTTTGATTATATACAAAGATGATACACATATTGAAAGCACGGTTAATAAACTAACACAGGCAGTTAAT
GAACTCAAATCACTTCAAAATGCACCTAGTTCGATAAAATCATCCATTGACGCCATTGAGTTACAACTTAGAAACATTTTAGACAATTGGAARCCAGAGGTAGATTTCGAGAAA
GCAAAGATTAATGAAAGTGCCACCACTAAGTCACTTGAAACAAACTTGCTGAGGAATAAATACACTAATCACGTTCATCTAACAAGATTTAGGATATGGATAGATTATAAAAAT
GCGAACAAAAACAATCATTTTATGGGAGAGTGTGGATTTAGTCTTGCAGAATCTTTTTTTGCTTCTAATCAGCCATTGGTCGATGAATTGTTTGGGTTGTATTCCCAGGTAGAGG
CCTTTTCTTTGCAAGGTCTTGGTTACTGTGTTCACCTTTATGAGCCATATATGAAAACTGAGGAAGCGATAAAACTGATGAAAGAGACCTTATATATTATACTACGGTTTATTGA
TATATGTGTTCACCATATCAATGAAGAGTCGATATCGATTGCCAACCCGTTAGAAACATATTTACGAAAAAAACATCTAATGCCTATGACTCCTACACCAAGGTCGTCCTATGGA
AGTCCACAAAGTGCTAGTACAAAGAGCTTGGTAAGTAAGATAATAGAGAGAATACCGCAACCGTTTATTGAGAGTGTAACTAATGTGTCGAGTCTTCAACTATTAGATCTTCGA
GATGACGAGTCAAAAATGTTTGGAACATTGCTGAACATGTGTAAGTCTATAAGGCGAAAATTTGACTCTGTTATGAGCGATTACGATTCCATTGTCACAGAAAAATCCGAAGG
CGAACAAAATGATGGTAAAGTAACTGTAGCTGAGTTCACATCTTTGTGTGAAGCGGAAGAAATGCTCTTAGCATTATGCTATAACTATTATAATCTGACGTTATACAGTTTCTTT
GAATTTGGGACTAATATTGAATACATGGAACATCTGTTGCTTCTTCTTGAAGAACAGCTTGCTCTCGACGAATACTATGGTTTTGAAAAGGTCTTGAATGTAGCTGTTGCAAATG
CTAAAAAAATGGGTTTCCACCGTTGGGAGTTTTACGTCGGTTATGAAGAGTCGACTGCTGAAAAGAGGCGGCTACTATGGTGGAAGTTATACAATTATGAAAAAGCCAGTACT
ATGAAGAAGGGTTTTTTTTCTGTGATTGATGATGCTACTGTCAACTGTTTATTACCTAAGATTTTTAGAAACTTTGGCTATCTGGATAGGGTGGAGTTTCTAGAAAATATTCAAA
AGCCAATGGATCTTAGTGTGTTTTCCGATGTTCCAATTTCTGTCCTTTGTAAATACGGTGAGTTGGCCCTTACAATAGTTACCAGTGAGTTTCATGAAAAATTTTTATATGCTGAT
AGATACACTTCTATTCGAAATTCCGCGAAACCGCCGACATTAAAAAACCAATTAATTAAGGAAATTGTGGATGGTATAGCTTATACAGAGACATCATATGAGGCAATCAGAAA
GCAAACTGCAAAACTATGGGATATTGCATTAGGTAAGGTGACCAAAGATAAAATCAATAAAGAAGATACAGCAGCAGCTAGCAAATTTACTTTGAGTTATGAATATCACAGAT
TCAGGCTAATCAATATGGCAGACAATTTAATTGCTAGACTTATGGTGAAACCAAAATCAGATTGGCTAATATCAGTCATGAAGGGGCATCTTAACAGACTATATGAGCACTGGA
AAGTAATGAATGAAATTATCCTAAGTATGGACAACGATTATTCAATTGCAACAACGTTCGAATATTATGCACCATCATGTCTGTGTTTAGCTACGCAGACTTTCCTTATTGTGAG
GAATATGGAAATGGATGATGTCAAGATGATGGTTGCAGTATATAAAAGATTTCTTAACCTAGGAATGTTTCTGCAGAGTGCCAAAGTATGCAGCCTTGCCGATAGTCATACATT

CAGAGATTTTTCTAGATCTTTTTCCTTTATTACGATAATTTCAAGATTGATGATAATCGAATTTATGCAAATTAAAGAATTGACGAAGGTAGAGTTTATTGAGAAGTTTTCTGAAG
TATGCCCTGACCTTGCAGATCTACCTCCGATGCTTCTAGATCCAAACTCTTGCTTATATTTTTCATTGTTACAGCAGATTAAGAAATCTGGTTTTACGTTGTCATTCAAAAAAATTC
TTGAAGACGCTAGAATGATGGACTTCAATTACGACCGCAATTTGGACTCAGAGGCCATTAAAAAGTGCAATGGTGAATTTAGCAAGTCAATGCCTTCCTGTACCAATGTCTCAG
ATACCACCACCGCTGTTTCTGACAACAGTGCTAAGAAGAAAGCTTCAATGGGGTCGGCGAGGGTAAATTCAACTGATACACTAACTGCATCTCCCTTATCGGGCTTAAGGAATC
AAACGCAGTTGGATTCTAAAGACAGTGTTCCATCTCTCGAGGCTTATACACCAATTGATTCTGTCTCTGACGTACCCACTGGGGAGATCAACGTTCCATTCCCTCCTGTTTATAAT
CAAAATGGATTGGATCAGCAAACCACTTATAATTTGGGAACTTTAGATGAGTTTGTTAACAAGGGAGATTTGAATGAACTCTATAATAGCCTATGGGGTGACCTATTTTCTGAT
GTTTACTTGTGA

>73

ATGCAAACATTAGAAACTACATCAAAATCAAATCCAGGGGAAGTCAAAGCACAGAAGCCTAGTACAAGAAGAACAAAAGTTGGAAAAGCTTGTGATAGCTGTAGAAGGAGG
AAAATAAAATGTAATGGGCTAAAACCTTGTCCATCTTGTACAATCTATGGTTGTGAATGTACATATACTGATGCAAAATCGACAAAAAATCTCAAATCAAATGATGCAGGTAAA
CCAAAACCAACAGGGAGAGTATCAAAGAATAAAGAAACTACTAGAATCGACAAAGATATTAGGAAATCAGAGCAGCAGTATGTCCCTATTAATGCTAATATTCATGTTGGTCC
CAGGTTCCCCTCCGAGAATATATTGAATGGATATCCACAATGTGGAGCACCACAGAACAATGTTGTGGGTAATCCACTAGCGGTTAATCCTCAATGCCATAGAGGTCTTTCTGA
AACTCCTATGTCCTCAACATTCAAAGAATCTAACTTAAGAGATGATCGGCTACTACAGTCATCAGATACAGATGATATGAGGAATGGTGACTCGGAAGAAAGGGACTTGAAAG
GGAGTGACAGCGAGAATGTCAAAAGTAAAGACAATAAAAGTGATCCTTTGATTATATACAAAGATGATACACATATTGAAAGCACGGTTAATAAACTAACACAGGCAGTTAAT
GAACTCAAATCACTTCAAAATGCACCTAGTTCGATAAAATCATCCATTGACGCCATTGAGTTACAACTTAGAAACATTTTAGACAATTGGAAACCAGAGGTAGATTTCGAGAAA
GCAAAGATTAATGAAAGTGCCACCACTAAGTCACTTGAAACAAATTTGCTGAGGAATAAATACACTAATCACGTTCATCTAACAAGATTTAGGATATGGATAGATTATAAAAAT
GCGAACAAAAACAATCATTTTATGGGAGAGTGTGGATTTAGTCTTGCAGAATCTTTTTTTGCTTCTAATCAGCCATTGGTCGATGAATTGTTTGGGTTGTATTCCCAGGTAGAGG
CCTTTTCTTTGCAAGGTCTTGGTTACTGTGTTCACCTTTATGAGCCATATATGAAAACTGAGGAAGCGATAAAACTGATGAAAGAGACCTTATATATTATACTACGGTTTATTGA
TATATGTGTTCACCATATCAATGAAGAGTCGATATCGATTGCCAACCCGTTAGAAACATATTTACGAAAAAAACATCTAATGCCTATGACTCCTACACCAAGGTCGTCCTATGGA
AGTCCACAAAGTGCTAGTACAAAGAGCTTGGTAAGTAAGATAATAGAGAGAATACCGCAACCGTTTATTGAGAGTGTAACTAATGTGTCGAGTCTTCAACTATTAGATCTTCGA
GATGACGAGTCAAAAATGTTTGGAACATTGCTGAACATGTGTAAGTCTATAAGGCGAAAATTTGACTCTGTTATGAGCGATTACGATTCCATTGTCACAGAAAAATCCGAAGG
CGAACAAAATGATGGTAAAGTAACTGTAGCTGAGTTCACATCTTTGTGTGAAGCGGAAGAAATGCTCTTAGCATTATGCTATAATTATTATAATCTGACGTTATACAGTTTCTTT
GAATTTGGGACTAATATTGAATACATGGAACATCTGTTGCTTCTTCTTGAAGAACAGCTTGCTCTCGACGAATACTATGGTTTTGAAAAGGTCTTGAATGTAGCTGTTGCAAATG
CTAAAAAAATGGGTTTCCACCGTTGGGAGTTTTACGTCGGTTATGAAGAGTCGACTGCTGAAAAGAGGCGGCTACTATGGTGGAAGTTATACAATTATGAAAAAGCCAGTACT
ATGAAGAAGGGTTTTTTTTCTGTGATTGATGATGCTACTGTCAACTGTTTATTACCTAAGATTTTTAGAAACTTTGGCTATCTGGATAGGGTGGAGTTTCTAGAAAATATTCAAA
AGCCAATGGATCTTAGTGTGTTTTCCGATGTTCCAATTTCTGTCCTTTGTAAATACGGTGAGTTGGCCCTTACAATAGTTACCAGTGAGTTTCATGAAAAATTTTTATATGCTGAT
AGATACACTTCTATTCGAAATTCCGCGAAACCGCCGACATTAAAAAACCAATTAATTAAGGAAATTGTGGATGGTATAGCTTATACAGAGACATCATATGAGGCAATCAGAAA
GCAAACTGCAAAACTATGGGATATTGCATTAGGTAAGGTGACCAAAGATAAAATCAATAAAGAAGATACAGCAGCAGCTAGCAAATTTACTTTGAGTTATGAATATCACAGAT
TCAGGCTAATCAATATGGCAGACAATTTAATTGCTAGACTTATGGTGAAACCAAAATCAGATTGGCTAATATCAGTCATGAAGGGGCATCTTAACAGACTATATGAGCACTGGA
AAGTAATGAATGAAATTATCCTAAGTATGGACAACGATTATTCAATTGCAACAACGTTCGAATATTATGCACCATCATGTCTGTGTTTAGCTACGCAGACTTTCCTTATTGTGAG

GAATATGGAAATGGATGATGTCAAGATGATGGTTGCAGTATATAAAAGATTTCTTAACCTAGGAATGTTTCTGCAGAGTGCCAAAGTATGCAGCCTTGCCGATAGTCATACATT
CAGAGATTTTTCTAGATCTTTTTCCTTTATTACGATAATTTCAAGATTGATGATAATCGAATTTATGCAAATTAAAGAATTGACGAAGGTAGAGTTTATTGAGAAGTTTTCTGAAG
TATGCCCTGACCTTGCAGATCTACCTCCGATGCTTCTAGATCCAAACTCTTGCTTATATTTTTCATTGTTACAGCAGATTAAGAAATCTGGTTTTACGTTGTCATTCAAAAAAATTC
TTGAAGACGCTAGAATGATGGACTTCAATTACGACCGCAATTTGGACTCAGAGGCCATTAAAAAGTGCAATGGTGAATTTAGCAAGTCAATGCCTTCCTGTACCAATGTCTCAG
ATACCACCACCGCTGTTTCTGACAACAGTGCTAAGAAGAAAGCTTCAATGGGGTCGGCGAGGGTAAATTCAACTGATACACTAACTGCATCTCCCTTATCGGGCTTAAGGAATC
AAACGCAGTTGGATTCTAAAGACAGTGTTCCATCTCTCGAGGCTTATACACCAATTGATTCTGTCTCTGACGTGCCCACTGGGGAGATCAACGTTCCATTCCCTCCTGTTTATAAT
CAAAATGGATTGGATCAGCAAACCACTTACAATTTGGGAACTTTAGATGAGTTTGTTAACAAGGGAGATTTGAATGAACTCTATAATAGCCTATGGGGTGACCTATTTTCTGAT
GTTTACTTGTGA

>74

ATGCAAACATTAGAAACTACATCAAAATCAAATCCAGGGGAAGTCAAAGCACAGAAGCCTAGTACAAGAAGAACAAAAGTTGGAAAAGCTTGTGATAGCTGTAGAAGGAGG
AAAATAAAATGTAATGGGCTAAAACCTTGTCCATCTTGTACAATCTATGGTTGTGAATGTACATATACTGATGCAAAATCGACAAAAAATCTCAAATCAAATGATGCAGGTAAA
CCAAAACCAACAGGGAGAGTATCAAAGAATAAAGAAACTACTAGAATCGACAAAGATATTAGGAAATCAGAGCAGCAGTATGTCCCTATTAATGCTAATATTCATGTTGGTCC
CAGGTTCCCCTCCGAGAATATATTGAATGGATATCCACAATGTGGAGCACCACAGAACAATGTTGTGGGTAATCCACTAGCGGTTAATCCTCAATGCCATAGAGGTCTTTCTGA
AACTCCTATGTCCTCAACATTCAAAGAATCTAACTTAAGAGATGATCGGCTACTACAGTCATCAGATACAGATGATATGAGGAATGGTGACTCGGAAGAAAGGGACTTGAAAG
GGAGTGACAGCGAGAATGTCAAAAGTAAAGACAATAAAAGTGATCCTTTGATTATATACAAAGATGATACACATATTGAAAGCACGGTTAATAAACTAACACAGGCAGTTAAT
GAACTCAAATCACTTCAAAATGCACCTAGTTCGATAAAATCATCCATTGACGCCATTGAGTTACAACTTAGAAACATTTTAGACAATTGGAAACCAGAGGTAGATTTCGAGAAA
GCAAAGATTAATGAAAGTGCCACCACTAAGTCACTTGAAACAAACTTGCTGAGGAATAAATACACTAATCACGTTCATCTAACAAGATTTAGGATATGGATAGATTATAAAAAT
GCGAACAAAAACAATCATTTTATGGGAGAGTGTGGATTTAGTCTTGCAGAATCTTTTTTTGCTTCTAATCAGCCATTGGTCGATGAATTGTTTGGGTTGTATTCCCAGGTAGAGG
CCTTTTCTTTGCAAGGTCTTGGTTACTGTGTTCACCTTTATGAGCCATATATGAAAACTGAGGAAGCGATAAAACTGATGAAAGAGACCTTATATATTATACTACGGTTTATTGA
TATATGTGTTCACCATATCAATGAAGAGTCGATATCGATTGCCAACCCGTTAGAAACATATTTACGAAAAAAACATCTAATGCCTATGACTCCTACACCAAGGTCGTCCTATGGA
AGTCCACAAAGTGCTAGTACAAAGAGCTTGGTAAGTAWGATAATAGAGAGAATACCGCAACCGTTTATTGAGAGTGTAACTAATGTGTCGAGTCTTCAACTATTAGATCTTCG
AGATGACGAGTCAAAAATGTTTGGAACATTGCTGAACATGTGTAAGTCTATAAGGCGAAAATTTGACTCTGTTATGAGCGATTACGATTCCATTGTCACAGAAAAATCCGAAG
GCGAACAAAATGATGGTAAAGTAACTGTAGCTGAGTTCACATCTTTGTGTGAAGCGGAAGAAATGCTCTTAGCATTATGCTATAACTATTATAATCTGACGTTATACAGTTTCTT
TGAATTTGGGACTAATATTGAATACATGGAACATCTGTTGCTTCTTCTTGAAGAACAGCTTGCTCTCGACGAATACTATGGTTTTGAAAAGGTCTTGAATGTAGCTGTTGCAAAT
GCTAAAAAAATGGGTTTCCACCGTTGGGAGTTTTACGTCGGTTATGAAGAGTCGACTGCTGAAAAGAGGCGGCTACTATGGTGGAAGTTATACAATTATGAAAAAGCCAGTAC
TATGAAGAAGGGTTTTTTTTCTGTGATTGATGATGCTACTGTCAACTGTTTATTACCTAAGATTTTTAGAAACTTTGGCTATCTGGATAGGGTGGAGTTTCTAGAAAATATTCAA
AAGCCAATGGATCTTAGTGTGTTTTCCGATGTTCCAATTTCTGTCCTTTGTAAATACGGTGAGTTGGCCCTTACAATAGTTACCAGTGAGTTTCATGAAAAATTTTTATATGCTGA
TAGATACACTTCTATTCGAAATTCCGCGAAACCGCCGACATTAAAAAACCAATTAATTAAGGAAATTGTGGATGGTATAGCTTATACAGAGACATCATATGAGGCAATCAGAAA
GCAAACTGCAAAACTATGGGATATTGCATTAGGTAAGGTGACCAAAGATAAAATCAATAAAGAAGATACAGCAGCAGCTAGCAAATTTACTTTGAGTTATGAATATCACAGAT
TCAGGCTAATCAATATGGCAGACAATTTAATTGCTAGACTTATGGTGAAACCAAAATCAGATTGGCTAATATCAGTCATGAAGGGGCATCTTAACAGACTATATGAGCACTGGA

AAGTAATGAATGAAATTATCCTAAGTATGGACAACGATTATTCAATTGCAACAACGTTCGAATATTATGCACCATCATGTCTGTGTTTAGCTACGCAGACTTTCCTTATTGTGAG
GAATATGGAAATGGATGATGTCAAGATGATGGTTGCAGTATATAAAAGATTTCTTAACCTAGGAATGTTTCTGCAGAGTGCCAAAGTATGCAGCCTTGCCGATAGTCATACATT
CAGAGATTTTTCTAGATCTTTTTCCTTTATTACGATAATTTCAAGATTGATGATAATCGAATTTATGCAAATTAAAGAATTGACGAAGGTAGAGTTTATTGAGAAGTTTTCTGAAG
TATGCCCTGACCTTGCAGATCTACCTCCGATGCTTCTAGATCCAAACTCTTGCTTATATTTTTCATTGTTACAGCAGATTAAGAAATCTGGTTTTACGTTGTCATTCAAAAAAATTC
TTGAAGACGCTAGAATGATGGACTTCAATTACGACCGCAATTTGGACTCAGAGGCCATTAAAAAGTGCAATGGTGAATTTAGCAAGTCAATGCCTTCCTGTACCAATGTCTCAG
ATACCACCACCGCTGTTTCTGACAACAGTGCTAAGAAGAAAGCTTCAATGGGGTCGGCGAGGGTAAATTCAACTGATACACTAACTGCATCTCCCTTATCGGGCTTAAGGAATC
AAACGCAGTTGGATTCTAAAGACAGTGTTCCATCTCTCGAGGCTTATACACCAATTGATTCTGTCTCTGACGTACCCACTGGGGAGATCAACGTTCCATTCCCTCCTGTTTATAAT
CAAAATGGATTGGATCAGCAAACCACTTATAATTTGGGAACTTTAGATGAGTTTGTTAACAAGGGAGATTTGAATGAACTCTATAATAGCCTATGGGGTGACCTATTTTCTGAT
GTTTACTTGTGA

>75

ATGCAAACATTAGAAACTACATCAAAATCAAATCCAGGGGAaGTCAAAGCACAGAAGCCTAGTACAAGAAGAACAAAAGTTGGAAAAGCTTGTGATAGCTGTAGAAGGAGGA
AAATAAAATGTAATGGGCTAAAACCTTGTCCATCTTGTACAATCTATGGTTGTGAATGTACATATACTGATGCAAAATCGACAAAAAATCTCAAATCAAATGATGCAGGTAAAC
CAAAACCAACAGGGAGAGTATCAAAGAATAAAGAAACTACTAGAATCGACAAAGATATTAGGAAATCAGAGCAGCAGTATGTACCTATTAATGCTAATATTCATGTTGGTCCC
AGGTTCCCCTCCGAGAATATATTGAATGGATATCCACAATGTGRAGCACCACAGAACAATGTTGTGGGTAATCCACTAGCGGTTAATCCTCAATGCCATAGAGGTCTTTCTGAA
ACTCCTATGTCCTCAACATTCAAAGAATCTAACTTAAGAGATGATCGGCTACTACAGTCATCAGATACAGATGATATGAGGAATGGTGACTCGGAAGAAAGGGACTTGAAAGG
GAGTGACAGCGAGAATGTCAAAAGTAAAGACAATAAAAGTGATCCTTTGATTATATACAAAGATGATACACATATTGAAAGCACGGTTAATAAACTAACACAGGCAGTTAATG
AACTCAAATCACTTCAAAATGCACCTAGTTCGATAAAATCATCCATTGACGCCATTGAGTTACAACTTAGAAACATTTTAGACAATTGGAAACCAGAGGTAGATTTCGAGAAAG
CAAAGATTAATGAAAGTGCCACCACTAAGTCACTTGAAACAAACTTGCTGAGGAATAAATACACTAATCACGTTCATCTAACAAGATTTAGGATATGGATAGATTATAAAAATG
CGAACAAAAACAATCATTTTATGGGAGAGTGTGGATTTAGTCTTGCAGAATCTTTTTTTGCTTCTAATCAGCCATTGGTCGATGAATTGTTTGGGTTGTATTCCCAGGTAGAGGC
CTTTTCTTTGCAAGGTCTTGGTTACTGTGTTCACCTTTATGAGCCATATATGAAAACTGAGGAAGCGATAAAACTGATGAAAGAGACCTTATATATTATACTACGGTTTATTGAT
ATATGTGTTCACCATATCAATGAAGAGTCGATATCGATTGCCAACCCGTTAGAAACATATTTACGAAAAAAACATCTAATGCCTATGACTCCTACACCAAGGTCGTCCTATGGAA
GTCCACAAAGTGCTAGTACAAAGAGCTTGGTAAGTAAGATAATAGAGAGAATACCGCAACCGTTTATTGAGAGTGTAACTAATGTGTCGAGTCTTCAACTATTAGATCTTCGA
GATGACGAGTCAAAAATGTTTGGAACATTGCTGAACATGTGTAAGTCTATAAGGCGAAAATTTGACTCTGTTATGAGCGATTACGATTCCATTGTCACAGAAAAATCCGAAGSC
GAACAAAATGATGGTAAAGTAACTGTAGCTGAGTTCACATCTTTGTGTGAAGCGGAAGAAATGCTCTTAGCATTATGCTATAACTATTATAATCTGACGTTATACAGTTTCTTTG
AATTTGGGACTAATATTGAATACATGGAACATCTGTTGCTTCTTCTTGAAGAACAGCTTGCTCTCGACGAATACTATGGTTTTGAAAAGGTCTTGAATGTAGCTGTTGCAAATGC
TAAAAAAATGGGTTTCCACCGTTGGGAGTTTTACGTCGGTTATGAAGAGTCGACTGCTGAAAAGAGGCGGCTACTATGGTGGAAGTTATACAATTATGAAAAAGCCAGTACTA
TGAAGAAGGGTTTTTTTTCTGTGATTGATGATGCTACTGTCAACTGTTTATTACCTAAGATTTTTAGAAACTTTGGCTATCTGGATAGGGTGGAGTTTCTAGAAAATATTCAAAA
GCCAATGGATCTTAGTGTGTTTTCCGATGTTCCAATTTCTGTCCTTTGTAAATACGGTGAGTTGGCCCTTACAATAGTTACCAGTGAGTTTCATGAAAAATTTTTATATGCTGATA
GATACACTTCTATTCGAAATTCCGCGAAACCGCCGACATTAAAAAACCAATTAATTAAGGAAATTGTGGATGGTATAGCTTATACAGAGACATCATATGAGGCAATCAGAAAGC
AAACTGCAAAACTATGGGATATTGCATTAGGTAAGGTGACCAAAGATAAAATCAATAAAGAAGATACAGCAGCAGCTAGCAAATTTACTTTGAGTTATGAATATCACAGATTC

AGGCTAATCAATATGGCAGACAATTTAATTGCTAGACTTATGGTGAAACCAAAATCAGATTGGCTAATATCAGTCATGAAGGGGCATCTTAACAGACTATATGAGCACTGGAA
AGTAATGAATGAAATTATCCTAAGTATGGACAACGATTATTCAATTGCAACAACGTTCGAATATTATGCACCATCATGTCTGTGTTTAGCTACGCAGACTTTCCTTATTGTGAGG
AATATGGAAATGGATGATGTCAAGATGATGGTTGCAGTATATAAAAGATTTCTTAACCTAGGAATGTTTCTGCAGAGTGCCAAAGTATGCAGCCTTGCCGATAGTCATACATTC
AGAGATTTTTCTAGATCTTTTTCCTTTATTACGATAATTTCAAGATTGATGATAATCGAATTTATGCAAATTAAAGAATTGACGAAGGTAGAGTTTATTGAGAAGTTTTCTGAAGT
ATGCCCTGACCTTGCAGATCTACCTCCGATGCTTCTAGATCCAAACTCTTGCTTATATTTTTCATTGTTACAGCAGATTAAGAAATCTGGTTTTACGTTGTCATTCAAAAAAATTCT
TGAAGACGCTAGAATGATGGACTTCAATTACGACCGCAATTTGGACTCAGAGGCCATTAAAAAGTGCAATGGTGAATTTAGCAAGTCAATGCCTTCCTGTACCAATGTCTCAGA
TACCACCACCGCTGTTTCTGACAACAGTGCTAAGAAGAAAGCTTCAATGGGGTCGGCGAGGGTAAATTCAACTGATACACTAACTGCATCTCCCTTATCGGGCTTAAGGAATCA
AACGCAGTTGGATTCTAAAGACAGTGTTCCATCTCTCGAGGCTTATACACCAATTGATTCTGTCTCTGACGTACCCACTGGGGAGATCAACGTTCCATTCCCTCCTGTTTATAATC
AAAATGGATTGGATCAGCAAACCACTTATAATTTGGGAACTTTAGATGAGTTTGTTAACAAGGGAGATTTGAATGAACTCTATAATAGCCTATGGGGTGACCTATTTTCTGATG
TTTACTTGTGA

>76

ATGCAAACATTAGAAACTACATCAAAATCAAATCCAGGGGAAGTCAAAGCACAGAAGCCTAGTACAAGAAGAACAAAAGTTGGAAAAGCTTGTGATAGCTGTAGAAGGAGG
AAAATAAAATGTAATGGGCTAAAACCTTGTCCATCTTGTACAATCTATGGTTGTGAATGTACATATACTGATGCAAAATCGACAAAAAATCTCAAATCAAATGATGCAGGTAAA
CCAAAACCAACAGGGAGAGTATCAAAGAATAAAGAAACTACTAGAATCGACAAAGATATTAGGAAATCAGAGCAGCAGTATGTACCTATTAATGCTAATATTCATGTTGGTCC
CAGGTTCCCCTCCGAGAATATATTGAATGGATATCCACAATGTGGAGCACCACAGAACAATGTTGTGGGTAATCCACTAGCGGTTAATCCTCAATGCCATAGAGGTCTTTCTGA
AACTCCTATGTCCTCAACATTCAAAGAATCTAACTTAAGAGATGATCGGCTACTACAGTCATCAGATACAGATGATATGAGGAATGGTGACTCGGAAGAAAGGGACTTGAAAG
GGAGTGACAGCGAGAATGTCAAAAGTAAAGACAATAAAAGTGATCCTTTGATTATATACAAAGATGATACACATATTGAAAGCACGGTTAATAAACTAACACAGGCAGTTAAT
GAACTCAAATCACTTCAAAATGCACCTAGTTCGATAAAATCATCCATTGACGCCATTGAGTTACAACTTAGAAACATTTTAGACAATTGGAAACCAGAGGTAGATTTCGAGAAA
GCAAAGATTAATGAAAGTGCCACCACTAAGTCACTTGAAACAAACTTGCTGAGGAATAAATACACTAATCACGTTCATCTAACAAGATTTAGGATATGGATAGATTATAAAAAT
GCGAACAAAAACAATCATTTTATGGGAGAGTGTGGATTTAGTCTTGCAGAATCTTTTTTTGCTTCTAATCAGCCATTGGTCGATGAATTGTTTGGGTTGTATTCCCAGGTAGAGG
CCTTTTCTTTGCAAGGTCTTGGTTACTGTGTTCACCTTTATGAGCCATATATGAAAACTGAGGAAGCGATAAAACTGATGAAAGAGACCTTATATATTATACTACGGTTTATTGA
TATATGTGTTCACCATATCAATGAAGAGTCGATATCGATTGCCAACCCGTTAGAAACATATTTACGAAAAAAACATCTAATGCCTATGACTCCTACACCAAGGTCGTCCTATGGA
AGTCCACAAAGTGCTAGTACAAAGAGCTTGGTAAGTATGATAATAGAGAGAATACCGCAACCGTTTATTGAGAGTGTAACTAATGTGTCGAGTCTTCAACTATTAGATCTTCGA
GATGACGAGTCAAAAATGTTTGGAACATTGCTGAACATGTGTAAGTCTATAAGGCGAAAATTTGACTCTGTTATGAGCGATTACGATTCCATTGTCACAGAAAAATCCGAAGG
CGAACAAAATGATGGTAAAGTAACTGTAGCTGAGTTCACATCTTTGTGTGAAGCGGAAGAAATGCTCTTAGCATTATGCTATAACTATTATAATCTGACGTTATACAGTTTCTTT
GAATTTGGGACTAATATTGAATACATGGAACATCTGTTGCTTCTTCTTGAAGAACAGCTTGCTCTCGACGAATACTATGGTTTTGAAAAGGTCTTGAATGTAGCTGTTGCAAATG
CTAAAAAAATGGGTTTCCACCGTTGGGAGTTTTACGTCGGTTATGAAGAGTCGACTGCTGAAAAGAGGCGGCTACTATGGTGGAAGTTATACAATTATGAAAAAGCCAGTACT
ATGAAGAAGGGTTTTTTTTCTGTGATTGATGATGCTACTGTCAACTGTTTATTACCTAAGATTTTTAGAAACTTTGGCTATCTGGATAGGGTGGAGTTTCTAGAAAATATTCAAA
AGCCAATGGATCTTAGTGTGTTTTCCGATGTTCCAATTTCTGTCCTTTGTAAATACGGTGAGTTGGCCCTTACAATAGTTACCAGTGAGTTTCATGAAAAATTTTTATATGCTGAT
AGATACACTTCTATTCGAAATTCCGCGAAACCGCCGACATTAAAAAACCAATTAATTAAGGAAATTGTGGATGGTATAGCTTATACAGAGACATCATATGAGGCAATCAGAAA

GCAAACTGCAAAACTATGGGATATTGCATTAGGTAAGGTGACCAAAGATAAAATCAATAAAGAAGATGCAGCAGCAGCTAGCAAATTTACTTTGAGTTATGAATATCACAGAT
TCAGGCTAATCAATATGGCAGACAATTTAATTGCTAGACTTATGGTGAAACCAAAATCAGATTGGCTAATATCAGTCATGAAGGGGCATCTTAACAGACTATATGAGCACTGGA
AAGTAATGAATGAAATTATCCTAAGTATGGACAACGATTATTCAATTGCAACAACGTTCGAATATTATGCACCATCATGTCTGTGTTTAGCTACGCAGACTTTCCTTATTGTGAG
GAATATGGAAATGGATGATGTCAAGATGATGGTTGCAGTATATAAAAGATTTCTTAACCTAGGAATGTTTCTGCAGAGTGCCAAAGTATGCAGCCTTGCCGATAGTCATACATT
CAGAGATTTTTCTAGATCTTTTTCCTTTATTACGATAATTTCAAGATTGATGATAATCGAATTTATGCAAATTAAAGAATTGACGAAGGTAGAGTTTATTGAGAAGTTTTCTGAAG
TATGCCCTGACCTTGCAGATCTACCTCCGATGCTTCTAGATCCAAACTCTTGCTTATATTTTTCATTGTTACAGCAGATTAAGAAATCTGGTTTTACGTTGTCATTCAAAAAAATTC
TTGAAGACGCTAGAATGATGGACTTCAATTACGACCGCAATTTGGACTCAGAGGCCATTAAAAAGTGCAATGGTGAATTTAGCAAGTCAATGCCTTCCTGTACCAATGTCTCAG
ATACCACCACCGCTGTTTCTGACAACAGTGCTAAGAAGAAAGCTTCAATGGGGTCGGCGAGGGTAAATTCAACTGATACACTAACTGCATCTCCCTTATCAGGCTTAAGGAATC
AAACGCAGTTGGATTCTAAAGACAGTGTTCCATCTCTCGAGGCTTATACACCAATTGATTCTGTCTCTGACGTACCCACTGGGGAGATCAACGTTCCATTCCCTCCTGTTTATAAT
CAAAATGGATTGGATCAGCAAACCACTTATAATTTGGGAACTTTAGATGAGTTTGTTAACAAGGGAGATTTGAATGAACTCTATAATAGCCTATGGGGTGACCTATTTTCTGAT
GTTTACTTGTGA

>77

ATGCAAACATTAGAAACTACATCAAAATCAAATCCAGGGGAAGTCAAAGCACAGAAGCCTAGTACAAGAAGAACAAAAGTTGGAAAAGCTTGTGATAGCTGTAGAAGGAGG
AAAATAAAATGTAATGGGCTAAAACCTTGTCCATCTTGTACAATCTATGGTTGTGAATGTACATATACTGATGCAAAATCGACAAAAAATCTCAAATCAAATGATGCAGGTAAA
CCAAAACCAACAGGGAGAGTATCAAAGAATAAAGAAACTACTAGAATCGACAAAGATATTAGGAAATCAGAGCAGCAGTATGTACCTATTAATGCTAATATTCATGTTGGTCC
CAGGTTCCCCTCCGAGAATATATTGAATGGATATCCACAATGTGRAGCACCACAGAACAATGTTGTGGGTAATCCACTAGCGGTTAATCCTCAATGCCATAGAGGTCTTTCTGA
AACTCCTATGTCCTCAACATTCAAAGAATCTAACTTAAGAGATGATCGGCTACTACAGTCATCAGATACAGATGATATGAGGAATGGTGACTCGGAAGAAAGGGACTTGAAAG
GGAGTGACAGCGAGAATGTCAAAAGTAAAGACAATAAAAGTGATCCTTTGATTATATACAAAGATGATACACATATTGAAAGCACGGTTAATAAACTAACACAGGCAGTTAAT
GAACTCAAATCACTTCAAAATGCACCTAGTTCGATAAAATCATCCATTGACGCCATTGAGTTACAACTTAGAAACATTTTAGACAATTGGAAACCAGAGGTAGATTTCGAGAAA
GCAAAGATTAATGAAAGTGCCACCACTAAGTCACTTGAAACAAACTTGCTGAGGAATAAATACACTAATCACGTTCATCTAACAAGATTTAGGATATGGATAGATTATAAAAAT
GCGAACAAAAACAATCATTTTATGGGAGAGTGTGGATTTAGTCTTGCAGAATCTTTTTTTGCTTCTAATCAGCCATTGGTCGATGAATTGTTTGGGTTGTATTCCCAGGTAGAGG
CCTTTTCTTTGCAAGGTCTTGGTTACTGTGTTCACCTTTATGAGCCATATATGAAAACTGAGGAAGCGATAAAACTGATGAAAGAGACCTTATATATTATACTACGGTTTATTGA
TATATGTGTTCACCATATCAATGAAGAGTCGATATCGATTGCCAACCCGTTAGAAACATATTTACGAAAAAAACATCTAATGCCTATGACTCCTACACCAAGGTCGTCCTATGGA
AGTCCACAAAGTGCTAGTACAAAGAGCTTGGTAAGTAAGATAATAGAGAGAATACCGCAACCGTTTATTGAGAGTGTAACTAATGTGTCGAGTCTTCAACTATTAGATCTTCGA
GATGACGAGTCAAAAATGTTTGGAACATTGCTGAACATGTGTAAGTCTATAAGGCGAAAATTTGACTCTGTTATGAGCGATTACGATTCCATTGTCACAGAAAAATCCGAAGG
CGAACAAAATGATGGTAAAGTAACTGTAGCTGAGTTCACATCTTTGTGTGAAGCGGAAGAAATGCTCTTAGCATTATGCTATAACTATTATAATCTGACGTTATACAGTTTCTTT
GAATTTGGGACTAATATTGAATACATGGAACATCTGTTGCTTCTTCTTGAAGAACAGCTTGCTCTCGACGAATACTATGGTTTTGAAAAGGTCTTGAATGTAGCTGTTGCAAATG
CTAAAAAAATGGGTTTCCACCGTTGGGAGTTTTACGTCGGTTATGAAGAGTCGACTGCTGAAAAGAGGCGGCTACTATGGTGGAAGTTATACAATTATGAAAAAGCCAGTACT
ATGAAGAAGGGTTTTTTTTCTGTGATTGATGATGCTACTGTCAACTGTTTATTACCTAAGATTTTTAGAAACTTTGGCTATCTGGATAGGGTGGAGTTTCTAGAAAATATTCAAA
AGCCAATGGATCTTAGTGTGTTTTCCGATGTTCCAATTTCTGTCCTTTGTAAATACGGTGAGTTGGCCCTTACAATAGTTACCAGTGAGTTTCATGAAAAATTTTTATATGCTGAT

AGATACACTTCTATTCGAAATTCCGCGAAACCGCCGACATTAAAAAACCAATTAATTAAGGAAATTGTGGATGGTATAGCTTATACAGAGACATCATATGAGGCAATCAGAAA
GCAAACTGCAAAACTATGGGATATTGCATTAGGTAAGGTGACCAAAGATAAAATCAATAAAGAAGATACAGCAGCAGCTAGCAAATTTACTTTGAGTTATGAATATCACAGAT
TCAGGCTAATCAATATGGCAGACAATTTAATTGCTAGACTTATGGTGAAACCAAAATCAGATTGGCTAATATCAGTCATGAAGGGGCATCTTAACAGACTATATGAGCACTGGA
AAGTAATGAATGAAATTATCCTAAGTATGGACAACGATTATTCAATTGCAACAACGTTCGAATATTATGCACCATCATGTCTGTGTTTAGCTACGCAGACTTTCCTTATTGTGAG
GAATATGGAAATGGATGATGTCAAGATGATGGTTGCAGTATATAAAAGATTTCTTAACCTAGGAATGTTTCTGCAGAGTGCCAAAGTATGCAGCCTTGCCGATAGTCATACATT
CAGAGATTTTTCTAGATCTTTTTCCTTTATTACGATAATTTCAAGATTGATGATAATCGAATTTATGCAAATTAAAGAATTGACGAAGGTAGAGTTTATTGAGAAGTTTTCTGAAG
TATGCCCTGACCTTGCAGATCTACCTCCGATGCTTCTAGATCCAAACTCTTGCTTATATTTTTCATTGTTACAGCAGATTAAGAAATCTGGTTTTACGTTGTCATTCAAAAAAATTC
TTGAAGACGCTAGAATGATGGACTTCAATTACGACCGCAATTTGGACTCAGAGGCCATTAAAAAGTGCAATGGTGAATTTAGCAAGTCAATGCCTTCCTGTACCAATGTCTCAG
ATACCACCACCGCTGTTTCTGACAACAGTGCTAAGAAGAAAGCTTCAATGGGGTCGGCGAGGGTAAATTCAACTGATACACTAACTGCATCTCCCTTATCGGGCTTAAGGAATC
AAACGCAGTTGGATTCTAAAGACAGTGTTCCATCTCTCGAGGCTTATACACCAATTGATTCTGTCTCTGACGTACCCACTGGGGAGATCAACGTTCCATTCCCTCCTGTTTATAAT
CAAAATGGATTGGATCAGCAAACCACTTATAATTTGGGAACTTTAGATGAGTTTGTTAACAAGGGAGATTTGAATGAACTCTATAATAGCCTATGGGGTGACCTATTTTCTGAT
GTTTACTTGTGA

>78

ATGCAAACATTAGAAACTACATCAAAATCAAATCCAGGGGAAGTCAAAGCACAGAAGCCTAGTACAAGAAGAACAAAAGTTGGAAAAGCTTGTGATAGCTGTAGAAGGAGG
AAAATAAAATGTAATGGGCTAAAACCTTGTCCATCTTGTACAATCTATGGTTGTGAATGTACATATACTGATGCAAAATCGACAAAAAATCTCAAATCAAATGATGCAGGTAAA
CCAAAACCAACAGGGAGAGTATCAAAGAATAAAGAAACTACTAGAATCGACAAAGATATTAGGAAATCAGAGCAGCAGTATGTACCTATTAATGCTAATATTCATGTTGGTCC
CAGGTTCCCCTCCGAGAATATATTGAATGGATATCCACAATGTGGAGCACCACAGAACAATGTTGTGGGTAATCCACTAGCGGTTAATCCTCAATGCCATAGAGGTCTTTCTGA
AACTCCTATGTCCTCAACATTCAAAGAATCTAACTTAAGAGATGATCGGCTACTACAGTCATCAGATACAGATGATATGAGGAATGGTGACTCGGAAGAAAGGGACTTGAAAG
GGAGTGACAGCGAGAATGTCAAAAGTAAAGACAATAAAAGTGATCCTTTGATTATATACAAAGATGATACACATATTGAAAGCACGGTTAATAAACTAACACAGGCAGTTAAT
GAACTCAAATCACTTCAAAATGCACCTAGTTCGATAAAATCATCCATTGACGCCATTGAGTTACAACTTAGAAACATTTTAGACAATTGGAAACCAGAGGTAGATTTCGAGAAA
GCAAAGATTAATGAAAGTGCCACCACTAAGTCACTTGAAACAAACTTGCTGAGGAATAAAWACRCTAATCACGTTCATCTAACAAGATTTAGGATATGGATAGATTATAAAAA
TGCGAACAAAAACAATCATTTTATGGGAGAGTGTGGATTTAGTCTTGCAGAATCTTTTTTTGCTTCTAATCAGCCATTGGTCGATGAATTGTTTGGGTTGTATTCCCAGGTAGAG
GCCTTTTCTTTGCAAGGTCTTGGTTACTGTGTTCACCTTTATGAGCCATATATGAAAACTGAGGAAGCGATAAAACTGATGAAAGAGACCTTATATATTATACTACGGTTTATTG
ATATATGTGTTCACCATATCAATGAAGAGTCGATATCGATTGCCAACCCGTTAGAAACATATTTACGAAAAAAACATCTAATGCCTATGACTCCTACACCAAGGTCGTCCTATGG
AAGTCCACAAAGTGCTAGTACAAAGAGCTTGGTAAGTAWGATAATAGAGAGAATACCGCAACCGTTTATTGAGAGTGTAACTAATGTGTCGAGTCTTCAACTATTAGATCTTC
GAGATGACGAGTCAAAAATGTTTGGAACATTGCTGAACATGTGTAAGTCTATAAGGCGAAAATTTGACTCTGTTATGAGCGATTACGATTCCATTGTCACAGAAAAATCCGAA
GGCGAACAAAATGATGGTAAAGTAACTGTAGCTGAGTTCACATCTTTGTGTGAAGCGGAAGAAATGCTCTTAGCATTATGCTATAACTATTATAATCTGACGTTATACAGTTTCT
TTGAATTTGGGACTAATATTGAATACATGGAACATCTGTTGCTTCTTCTTGAAGAACAGCTTGCTCTCGACGAATACTATGGTTTTGAAAAGGTCTTGAATGTAGCTGTTGCAAA
TGCTAAAAAAATGGGTTTCCACCGTTGGGAGTTTTACGTCGGTTATGAAGAGTCGACTGCTGAAAAGAGGCGGCTACTATGGTGGAAGTTATACAATTATGAAAAAGCCAGTA
CTATGAAGAAGGGTTTTTTTTCTGTGATTGATGATGCTACTGTCAACTGTTTATTACCTAAGATTTTTAGAAACTTTGGCTATCTGGATAGGGTGGAGTTTCTAGAAAATATTCAA

AAGCCAATGGATCTTAGTGTGTTTTCCGATGTTCCAATTTCTGTCCTTTGTAAATACGGTGAGTTGGCCCTTACAATAGTTACCAGTGAGTTTCATGAAAAATTTTTATATGCTGA
TAGATACACTTCTATTCGAAATTCCGCGAAACCGCCGACATTAAAAAACCAATTAATTAAGGAAATTGTGGATGGTATAGCTTATACAGAGACATCATATGAGGCAATCAGAAA
GCAAACTGCAAAACTATGGGATATTGCATTAGGTAAGGTGACCAAAGATAAAATCAATAAAGAAGATGCAGCAGCAGCTAGCAAATTTACTTTGAGTTATGAATATCACAGAT
TCAGGCTAATCAATATGGCAGACAATTTAATTGCTAGACTTATGGTGAAACCAAAATMAGATTGGCTAATATCAGTCATGAAGGGGCATCTTAACAGACTATATGAGCACTGG
AAAGTAATGAATGAAATTATCCTAAGTATGGACAACGATTATTCAATTGCAACAACGTTCGAATATTATGCACCATCATGTCTGTGTTTAGCTACGCAGACTTTCCTTATTGTGA
GGAATATGGAAATGGATGATGTCAAGATGATGGTTGCAGTATATAAAAGATTTCTTAACCTAGGAATGTTTCTGCAGAGTGCCAAAGTATGCAGCCTTGCCGATAGTCATACA
TTCAGAGATTTTTCTAGATCTTTTTCCTTTATTACGATAATTTCAAGATTGATGATAATCGAATTTATGCAAATTAAAGAATTGACGAAGGTAGAGTTTATTGAGAAGTTTTCTGA
AGTATGCCCTGACCTTGCAGATCTACCTCCGATGCTTCTAGATCCAAACTCTTGCTTATATTTTTCATTGTTACAGCAGATTAAGAAATCTGGTTTTACGTTGTCATTCAAAAAAAT
TCTTGAAGACGCTAGAATGATGGACTTCAATTACGACCGCAATTTGGACTCAGAGGCCATTAAAAAGTGCAATGGTGAATTTAGCAAGTCAATGCCTTCCTGTACCAATGTCTC
AGATACCACCACCGCTGTTTCTGACAACAGTGCTAAGAAGAAAGCTTCAATGGGGTCGGCGAGGGTAAATTCAACTGATACACTAACTGCATCTCCCTTATCAGGCTTAAGGA
ATCAAACGCAGTTGGATTCTAAAGACAGTGTTCCATCTCTCGAGGCTTATACACCAATTGATTCTGTCTCTGACGTACCCACTGGGGAGATCAACGTTCCATTCCCTCCTGTTTAT
AATCAAAATGGATTGGATCAGCAAACCACTTATAATTTGGGAACTTTAGATGAGTTTGTTAACAAGGGAGATTTGAATGAACTCTATAATAGCCTATGGGGTGACCTATTTTCT
GATGTTTACTTGTGA

>80

ATGCAAACATTAGAAACTACATCAAAATCAAATCCAGGGGAAGTCAAAGCACAGAAGCCTAGTACAAGAAGAACAAAAGTTGGAAAAGCTTGTGATAGCTGTAGAAGGAGG
AAAATAAAATGTAATGGGCTAAAACCTTGTCCATCTTGTACAATCTATGGTTGTGAATGTACATATACTGATGCAAAATCGACAAAAAATCTCAAATCAAATGATGCAGGTAAA
CCAAAACCAACAGGGAGAGTATCAAAGAATAAAGAAACTACTAGAATCGACAAAGATATTAGGAAATCAGAGCAGCAGTATGTCCCTATTAATGCTAATATTCATGTTGGTCC
CAGGTTCCCCTCCGAGAATATATTGAATGGATATCCACAATGTGGAGCACCACAGAACAATGTTGTGGGTAATCCACTAGCGGTTAATCCTCAATGCCATAGAGGTCTTTCTGA
AACTCCTATGTCCTCAACATTCAAAGAATCTAACTTGAGAGATGATCGGCTACTACAGTCATCAGATACAGATGATATGAGGAATGGTGACTCGGAAGAAAGGGACTTGAAAG
GGAGTGACAGCGAGAATGTCAAAAGTAAAGACAATAAAAGTGATCCTTTGATTATATACAAAGATGATACACATATTGAAAGCACGGTTAATAAACTAACACAGGCAGTTAAT
GAACTCAAATCACTTCAAAATGCACCTAGTTCGATAAAATCATCCATTGACGCCATTGAGTTACAACTTAGAAACATTTTAGACAATTGGAAACCAGAGGTAGATTTCGAGAAA
GCAAAGATTAATGAAAGTGCCACCACTAAGTCACTTGAAACAAACTTGCTGAGGAATAAATACACTAATCACGTTCATCTAACAAGATTTAGGATATGGATAGATTATAAAAAT
GCGAACAAAAACAATCATTTTATGGGAGAGTGTGGATTTAGTCTTGCAGAATCTTTTTTTGCTTCTAATCAGCCATTGGTCGATGAATTGTTTGGGTTGTATTCCCAGGTAGAGG
CCTTTTCTTTGCAAGGTCTTGGTTACTGTGTTCACCTTTATGAGCCATATATGAAAACTGAGGAAGCGATAAAACTGATGAAAGAGACCTTATATATTATACTACGGTTTATTGA
TATATGTGTTCACCATATCAATGAAGAGTCGATATCGATTGCCAACCCGTTAGAAACATATTTACGAAAAAAACATCTAATGCCTATGACTCCTACACCAAGGTCGTCCTATGGA
AGTCCACAAAGTGCTAGTACAAAGAGCTTGGTAAGTAWGATAATAGAGAGAATACCGCAACCGTTTATTGAGAGTGTAACTAATGTGTCGAGTCTTCAACTATTAGATCYTCG
AGATGACGAGTCAAAAATGTTTGGAACATTGCTGAACATGTGTAAGTCTATAAGGCGAAAATTTGACTCTGTTATGAGCGATTACGATTCCATTGTCACAGAAAAATCCGAAG
GCGAACAAAATGATGGTAAAGTAACTGTAGCTGAGTTCACATCTTTGTGTGAAGCGGAAGAAATGCTCTTAGCATTATGCTATAACTATTATAATCTGACGTTATACAGTTTCTT
TGAATTTGGGACTAATATTGAATACATGGAACATCTGTTGCTTCTTCTTGAAGAACAGCTTGCTCTCGACGAATACTATGGTTTTGAAAAGGTCTTGAATGTAGCTGTTGCAAAT
GCTAAAAAAATGGGTTTCCACCGTTGGGAGTTTTACGTCGGTTATGAAGAGTCGACTGCTGAAAAGAGGCGGCTACTATGGTGGAAGTTATACAATTATGAAAAAGCCAGTAC

TATGAAGAAGGGTTTTTTTTCTGTGATTGATGATGCTACTGTCAACTGTTTATTACCTAAGATTTTTAGAAACTTTGGCTATCTGGATAGGGTGGAGTTTCTAGAAAATATTCAA
AAGCCAATGGATCTTAGTGTGTTTTCCGATGTTCCAATTTCTGTCCTTTGTAAATACGGTGAGTTGGCCCTTACAATAGTTACCAGTGAGTTTCATGAAAAATTTTTATATGCTGA
TAGATACACTTCTATTCGAAATTCCGCGAAACCGCCGACATTAAAAAACCAATTAATTAAGGAAATTGTGGATGGTATAGCTTATACAGAGACATCATATGAGGCAATCAGAAA
GCAAACTGCAAAACTATGGGATATTGCATTAGGTAAGGTGACCAAAGATAAAATCAATAAAGAAGATACAGCAGCAGCTAGCAAATTTACTTTGAGTTATGAATATCACAGAT
TCAGGCTAATCAATATGGCAGACAATTTAATTGCTAGACTTATGGTGAAACCAAAATCAGATTGGCTAATATCAGTCATGAAGGGGCATCTTAACAGACTATATGAGCACTGGA
AAGTAATGAATGAAATTATCCTAAGTATGGACAACGATTATTCAATTGCAACAACGTTCGAATATTATGCACCATCATGTCTGTGTTTAGCTACGCAGACTTTCCTTATTGTGAG
GAATATGGAAATGGATGATGTCAAGATGATGGTTGCAGTATATAAAAGATTTCTTAACCTAGGAATGTTTCTGCAGAGTGCCAAAGTATGCAGCCTTGCCGATAGTCATACATT
CAGAGATTTTTCTAGATCTTTTTCCTTTATTACGATAATTTCAAGATTGATGATAATCGAATTTATGCAAATTAAAGAATTGACGAAGGTAGAGTTTATTGAGAAGTTTTCTGAAG
TATGCCCTGACCTTGCAGATCTACCTCCGATGCTTCTAGATCCAAACTCTTGCTTATATTTTTCATTGTTACAGCAGATTAAGAAATCTGGTTTTACGTTGTCATTCAAAAAAAATTC
TTGAAGACGCTAGAATGATGGACTTCAATTACGACCGCAATTTGGACTCAGAGGCCATTAAAAAGTGCAATGGTGAATTTAGCAAGTCAATGCCTTCCTGTACCAATGTCTCAG
ATACCACCACCGCTGTTTCTGACAACAGTGCTAAGAAGAAAGCTTCAATGGGGTCGGCGAGGGTAAATTCAACTGATACACTAACTGCATCTCCCTTATCGGGCTTAAGGAATC
AAACGCAGTTGGATTCTAAAGACAGTGTTCCATCTCTCGAGGCTTATACACCAATTGATTCTGTCTCTGACGTACCCACTGGGGAGATCAACGTTCCATTCCCTCCTGTTTATAAT
CAAAATGGATTGGATCAGCAAACCACTTATAATTTGGGAACTTTAGATGAGTTTGTTAACAAGGGAGATTTGAATGAACTCTATAATAGCCTATGGGGTGACCTATTTTCTGAT
GTTTACTTGTGA

>81

ATGCAAACATTAGAAACTACATCAAAATCAAATCCAGGGGAAGTCAAAGCACAGAAGCCTAGTACAAGAAGAACAAAAGTTGGAAAAGCTTGTGATAGCTGTAGAAGGAGG
AAAATAAAATGTAATGGGCTAAAACCTTGTCCATCTTGTACAATCTATGGTTGTGAATGTACATATACTGATGCAAAATCGACAAAAAATCTCAAATCAAATGATGCAGGTAAA
CCAAAACCAACAGGGAGAGTATCAAAGAATAAAGAAACTACTAGAATCGACAAAGATATTAGGAAATCAGAGCAGCAGTATGTCCCTATTAATGCTAATATTCATGTTGGTCC
CAGGTTCCCCTCCGAGAATATATTGAATGGATATCCACAATGTGGAGCACCACAGAACAATGTTGTGGGTAATCCACTAGCGGTTAATCCTCAATGCCATAGAGGTCTTTCTGA
AACTCCTATGTCCTCAACATTCAAAGAATCTAACTTAAGAGATGATCGGCTACTACAGTCATCAGATACAGATGATATGAGGAATGGTGACTCGGAAGAAAGGGACTTGAAAG
GGAGTGACAGCGAGAATGTCAAAAGTAAAGACAATAAAAGTGATCCTTTGATTATATACAAAGATGATACACATATTGAAAGCACGGTTAATAAACTAACACAGGCAGTTAAT
GAACTCAAATCACTTCAAAATGCACCTAGTTCGATAAAATCATCCATTGACGCCATTGAGTTACAACTTAGAAACATTTTAGACAATTGGAAACCAGAGGTAGATTTCGAGAAA
GCAAAGATTAATGAAAGTGCCACCACTAAGTCACTTGAAACAAACTTGCTGAGGAATAAATACACTAATCACGTTCATCTAACAAGATTTAGGATATGGATAGATTATAAAAAT
GCGAACAAAAACAATCATTTTATGGGAGAGTGTGGATTTAGTCTTGCAGAATCTTTTTTTGCTTCTAATCAGCCATTGGTCGATGAATTGTTTGGGTTGTATTCCCAGGTAGAGG
CCTTTTCTTTGCAAGGTCTTGGTTACTGTGTTCACCTTTATGAGCCATATATGAAAACTGAGGAAGCGATAAAACTGATGAAAGAGACCTTATATATTATACTACGGTTTATTGA
TATATGTGTTCACCATATCAATGAAGAGTCGATATCGATTGCCAACCCGTTAGAAACATATTTACGAAAAAAACATCTAATGCCTATGACTCCTACACCAAGGTCGTCCTATGGA
AGTCCACAAAGTGCTAGTACAAAGAGCTTGGTAAGTATGATAATAGAGAGAATACCGCAACCGTTTATWRAGAGTGTAACTAATGTGTCGAGTCTTCAACTATTAGATCTTCG
AGATGACGAGTCAAAAATGTTTGGAACATTGCTGAACATGTGTAAGTCTATAAGGCGAAAATTTGACTCTGTTATGAGCGATTACGATTCCATTGTCACAGAAAAATCCGAAG
GCGAACAAAATGATGGTAAAGTAACTGTAGCTGAGTTCACATCTTTGTGTGAAGCGGAAGAAATGCTCTTAGCATTATGCTATAACTATTATAATCTGACGTTATACAGTTTCTT
TGAATTTGGGACTAATATTGAATACATGGAACATCTGTTGCTTCTTCTTGAAGAACAGCTTGCTCTCGACGAATACTATGGTTTTGAAAAGGTCTTGAATGTAGCTGTTGCAAAT

GCTAAAAAAATGGGTTTCCACCGTTGGGAGTTTTACGTCGGTTATGAAGAGTCGACTGCTGAAAAGAGGCGGCTACTATGGTGGAAGTTATACAATTATGAAAAAGCCAGTAC
TATGAAGAAGGGTTTTTTTTCTGTGATTGATGATGCTACTGTCAACTGTTTATTACCTAAGATTTTTAGAAACTTTGGCTATCTGGATAGGGTGGAGTTTCTAGAAAATATTCAA
AAGCCAATGGATCTTAGTGTGTTTTCCGATGTTCCAATTTCTGTCCTTTGTAAATACGGTGAGTTGGCCCTTACAATAGTTACCAGTGAGTTTCATGAAAAATTTTTATATGCTGA
TAGATACACTTCTATTCGAAATTCCGCGAAACCGCCGACATTAAAAAACCAATTAATTAAGGAAATTGTGGATGGTATAGCTTATACAGAGACATCATATGAGGCAATCAGAAA
GCAAACTGCAAAACTATGGGATATTGCATTAGGTAAGGTGACCAAAGATAAAATCAATAAAGAAGATACAGCAGCAGCTAGCAAATTTACTTTGAGTTATGAATATCACAGAT
TCAGGCTAATCAATATGGCAGACAATTTAATTGCTAGACTTATGGTGAAACCAAAATCMGATTGGCTAATATCAGTCATGAAGGGGCATCTTAACAGACTATATGAGCACTGG
AAAGTAATGAATGAAATTATCCTAAGTATGGACAACGATTATTCAATTGCAACAACGTTCGAATATTATGCACCATCATGTCTGTGTTTAGCTACGCAGACTTTCCTTATTGTGA
GGAATATGGAAATGGATGATGTCAAGATGATGGTTGCAGTATATAAAAGATTTCTTAACCTAGGAATGTTTCTGCAGAGTGCCAAAGTATGCAGCCTTGCCGATAGTCATACA
TTCAGAGATTTTTCTAGATCTTTTTCCTTTATTACGATAATTTCAAGATTGATGATAATCGAATTTATGCAAATTAAAGAATTGACGAAGGTAGAGTTTATTGAGAAGTTTTCTGA
AGTATGCCCTGACCTTGCAGATCTACCTCCGATGCTTCTAGATCCAAACTCTTGCTTATATTTTTCATTGTTACAGCAGATTAAGAAATCTGGTTTTACGTTGTCATTCAAAAAAAT
TCTTGAAGACGCTAGAATGATGGACTTCAATTACGACCGCAATTTGGACTCAGAGGCCATTAAAAAGTGCAATGGTGAATTTAGCAAGTCAATGCCTTCCTGTACCAATGTCTC
AGATACCACCACCGCTGTTTCTGACAACAGTGCTAAGAAGAAAGCTTCAATGGGGTCGGCGAGGGTAAATTCAACTGATACACTAACTGCATCTCCCTTATCGGGCTTAAGGA
ATCAAACGCAGTTGGATTCTAAAGACAGTGTTCCATCTCTCGAGGCTTATACACCAATTGATTCTGTCTCTGACGTACCCACTGGGGAGATCAACGTTCCATTCCCTCCTGTTTAT
AATCAAAATGGATTGGATCAGCAAACCACTTATAATTTGGGAACTTTAGATGAGTTTGTTAACAAGGGAGATTTGAATGAACTCTATAATAGCCTATGGGGTGACCTATTTTCT
GATGTTTACTTGTGA

>79

ATGCAAACATTAGAAACTACATCAAAATCAAATCCAGGGGAAGTCAAAGCACAGAAGCCTAGTACAAGAAGAACAAAAGTTGGAAAAGCTTGTGATAGCTGTAGAAGGAGG
AAAATAAAATGTAATGGGCTAAAACCTTGTCCATCTTGTACAATCTATGGTTGTGAATGTACATATACTGATGCAAAATCGACAAAAAATCTCAAATCAAATGATGCAGGTAAA
CCAAAACCAACAGGGAGAGTATCAAAGAATAAAGAAACTACTAGAATCGACAAAGATATTAGGAAATCAGAGCAGCAGTATGTACCTATTAATGCTAATATTCATGTTGGTCC
CAGGTTCCCCTCCGAGAATATATTGAATGGATATCCACAATGTGGAGCACCACAGAACAATGTTGTGGGTAATCCACTAGCGGTTAATCCTCAATGCCATAGAGGTCTTTCTGA
AACTCCTATGTCCTCAACATTCAAAGAATCTAACTTAAGAGATGATCGGCTACTACAGTCATCAGATACAGATGATATGAGGAATGGTGACTCGGAAGAAAGGGACTTGAAAG
GGAGTGACAGCGAGAATGTCAAAAGTAAAGACAATAAAAGTGATCCTTTGATTATATACAAAGATGATACACATATTGAAAGCACGGTTAATAAACTAACACAGGCAGTTAAT
GAACTCAAATCACTTCAAAATGCACCTAGTTCGATAAAATCATCCATTGACGCCATTGAGTTACAACTTAGAAACATTTTAGACAATTGGAAACCAGAGGTAGATTTCGAGAAA
GCAAAGATTAATGAAAGTGCCACCACTAAGTCACTTGAAACAAACTTGCTGAGGAATAAATACACTAATCACGTTCATCTAACAAGATTTAGGATATGGATAGATTATAAAAAT
GCGAACAAAAACAATCATTTTATGGGAGAGTGTGGATTTAGTCTTGCAGAATCTTTTTTTGCTTCTAATCAGCCATTGGTCGATGAATTGTTTGGGTTGTATTCCCAGGTAGAGG
CCTTTTCTTTGCAAGGTCTTGGTTACTGTGTTCACCTTTATGAGCCATATATGAAAACTGAGGAAGCGATAAAACTGATGAAAGAGACCTTATATATTATACTACGGTTTATTGA
TATATGTGTTCACCATATCAATGAAGAGTCGATATCGATTGCCAACCCGTTAGAAACATATTTACGAAAAAAACATCTAATGCCTATGACTCCTACACCAAGGTCGTCCTATGGA
AGTCCACAAAGTGCTAGTACAAAGAGCTTGGTAAGTAWGATAATAGAGAGAATACCGCAACCGTTTATTGAGAGTGTAACTAATGTGTCGAGTCTTCAACTATTAGATCTTCG
AGATGACGAGTCAAAAATGTTTGGAACATTGCTGAACATGTGTAAGTCTATAAGGCGAAAATTTGACTCTGTTATGAGCGATTACGATTCCATTGTCACAGAAAAATCCGAAGS
CGAACAAAATGATGGTAAAGTAACTGTAGCTGAGTTCACATCTTTGTGTGAAGCGGAAGAAATGCTCTTAGCATTATGCTATAACTATTATAATCTGACGTTATACAGTTTCTTT

GAATTTGGGACTAATATTGAATACATGGAACATCTGTTGCTTCTTCTTGAAGAACAGCTTGCTCTCGACGAATACTATGGTTTTGAAAAGGTCTTGAATGTAGCTGTTGCAAATG
CTAAAAAAATGGGTTTCCACCGTTGGGAGTTTTACGTCGGTTATGAAGAGTCGACTGCTGAAAAGAGGCGGCTACTATGGTGGAAGTTATACAATTATGAAAAAGCCAGTACT
ATGAAGAAGGGTTTTTTTTCTGTGATTGATGATGCTACTGTCAACTGTTTATTACCTAAGATTTTTAGAAACTTTGGCTATCTGGATAGGGTGGAGTTTCTAGAAAATATTCAAA
AGCCAATGGATCTTAGTGTGTTTTCCGATGTTCCAATTTCTGTCCTTTGTAAATACGGTGAGTTGGCCCTTACAATAGTTACCAGTGAGTTTCATGAAAAATTTTTATATGCTGAT
AGATACACTTCTATTCGAAATTCCGCGAAACCGCCGACATTAAAAAACCAATTAATTAAGGAAATTGTGGATGGTATAGCTTATACAGAGACATCATATGAGGCAATCAGAAA
GCAAACTGCAAAACTATGGGATATTGCATTAGGTAAGGTGACCAAAGATAAAATCAATAAAGAAGATGCAGCAGCAGCTAGCAAATTTACTTTGAGTTATGAATATCACAGAT
TCAGGCTAATCAATATGGCAGACAATTTAATTGCTAGACTTATGGTGAAACCAAAATCAGATTGGCTAATATCAGTCATGAAGGGGCATCTTAACAGACTATATGAGCACTGGA
AAGTAATGAATGAAATTATCCTAAGTATGGACAACGATTATTCAATTGCAACAACGTTCGAATATTATGCACCATCATGTCTGTGTTTAGCTACGCAGACTTTCCTTATTGTGAG
GAATATGGAAATGGATGATGTCAAGATGATGGTTGCAGTATATAAAAGATTTCTTAACCTAGGAATGTTTCTGCAGAGTGCCAAAGTATGCAGCCTTGCCGATAGTCATACATT
CAGAGATTTTTCTAGATCTTTTTCCTTTATTACGATAATTTCAAGATTGATGATAATCGAATTTATGCAAATTAAAGAATTGACGAAGGTAGAGTTTATTGAGAAGTTTTCTGAAG
TATGCCCTGACCTTGCAGATCTACCTCCGATGCTTCTAGATCCAAACTCTTGCTTATATTTTTCATTGTTACAGCAGATTAAGAAATCTGGTTTTACGTTGTCATTCAAAAAAATTC
TTGAAGACGCTAGAATGATGGACTTCAATTACGACCGCAATTTGGACTCAGAGGCCATTAAAAAGTGCAATGGTGAATTTAGCAAGTCAATGCCTTCCTGTACCAATGTCTCAG
ATACCACCACCGCTGTTTCTGACAACAGTGCTAAGAAGAAAGCTTCAATGGGGTCGGCGAGGGTAAATTCAACTGATACACTAACTGCATCTCCCTTATCAGGCTTAAGGAATC
AAACGCAGTTGGATTCTAAAGACAGTGTTCCATCTCTCGAGGCTTATACACCAATTGATTCTGTCTCTGACGTACCCACTGGGGAGATCAACGTTCCATTCCCTCCTGTTTATAAT
CAAAATGGATTGGATCAGCAAACCACTTATAATTTGGGAACTTTAGATGAGTTTGTTAACAAGGGAGATTTGAATGAACTCTATAATAGCCTATGGGGTGACCTATTTTCTGAT
GTTTACTTGTGA

>82

ATGCAAACATTAGAAACTACATCAAAATCAAATCCAGGGGAAGTCAAAGCACAGAAGCCTAGTACAAGAAGAACAAAAGTTGGAAAAGCTTGTGATAGCTGTAGAAGGAGG
AAAATAAAATGTAATGGGCTAAAACCTTGTCCATCTTGTACAATCTATGGTTGTGAATGTACATATACTGATGCAAAATCGACAAAAAATCTCAAATCAAATGATGCAGGTAAA
CCAAAACCAACAGGGAGAGTATCAAAGAATAAAGAAACTACTAGAATCGACAAAGATATTAGGAAATCAGAGCAGCAGTATGTCCCTATTAATGCTAATATTCATGTTGGTCC
CAGGTTCCCCTCCGAGAATATATTGAATGGATATCCACAATGTGGAGCACCACAGAACAATGTTGTGGGTAATCCACTAGCGGTTAATCCTCAATGCCATAGAGGTCTTTCTGA
AACTCCTATGTCCTCAACATTCAAAGAATCTAACTTAAGAGATGATCGGCTACTACAGTCATCAGATACAGATGATATGAGGAATGGTGACTCGGAAGAAAGGGACTTGAAAG
GGAGTGACAGCGAGAATGTCAAAAGTAAAGACAATAAAAGTGATCCTTTGATTATATACAAAGATGATACACATATTGAAAGCACGGTTAATAAACTAACACAGGCAGTTAAT
GAACTCAAATCACTTCAAAATGCACCTAGTTCGATAAAATCATCCATTGACGCCATTGAGTTACAACTTAGAAACATTTTAGACAATTGGAAACCAGAGGTAGATTTCGAGAAA
GCAAAGATTAATGAAAGTGCCACCACTAAGTCACTTGAAACAAACTTGCTGAGGAATAAATACACTAATCACGTTCATCTAACAAGATTTAGGATATGGATAGATTATAAAAAT
GCGAACAAAAACAATCATTTTATGGGAGAGTGTGGATTTAGTCTTGCAGAATCTTTTTTTGCTTCTAATCAGCCATTGGTCGATGAATTGTTTGGGTTGTATTCCCAGGTAGAGG
CCTTTTCTTTGCAAGGTCTTGGTTACTGTGTTCACCTTTATGAGCCATATATGAAAACTGAGGAAGCGATAAAACTGATGAAAGAGACCTTATATATTATACTACGGTTTATTGA
TATATGTGTTCACCATATCAATGAAGAGTCGATATCGATTGCCAACCCGTTAGAAACATATTTACGAAAAAAACATCTAATGCCTATGACTCCTACACCAAGGTCGTCCTATGGA
AGTCCACAAAGTGCTAGTACAAAGAGCTTGGTAAGTAWGATAATAGAGAGAATACCGCAACCGTTTATTGAGAGTGTAACTAATGTGTCGAGTCTTCAACTATTAGATCTTCG
AGATGACGAGTCAAAAATGTTTGGAAYATTGCTGAACATGTGTAAGTCTATAAGGCGAAAATTTGACTCTGTTATGAGCGATTACGATTCCATTGTCACAGAAAATCCGAAG

GCGAACAAAATGATGGTAAAGTAACTGTAGCTGAGTTCACATCTTTGTGTGAAGCGGAAGAAATGCTCTTAGCATTATGCTATAACTATTATAATCTGACGTTATACAGTTTCTT
TGAATTTGGGACTAATATTGAATACATGGAACATCTGTTGCTTCTTCTTGAAGAACAGCTTGCTCTCGACGAATACTATGGTTTTGAAAAGGTCTTGAATGTAGCTGTTGCAAAT
GCTAAAAAAATGGGTTTCCACCGTTGGGAGTTTTACGTCGGTTATGAAGAGTCGACTGCTGAAAAGAGGCGGCTACTATGGTGGAAGTTATACAATTATGAAAAAGCCAGTAC
TATGAAGAAGGGTTTTTTTTCTGTGATTGATGATGCTACTGTCAACTGTTTATTACCTAAGATTTTTAGAAACTTTGGCTATCTGGATAGGGTGGAGTTTCTAGAAAATATTCAA
AAGCCAATGGATCTTAGTGTGTTTTCCGATGTTCCAATTTCTGTCCTTTGTAAATACGGTGAGTTGGCCCTTACAATAGTTACCAGTGAGTTTCATGAAAAATTTTTATATGCTGA
TAGATACACTTCTATTCGAAATTCCGCGAAACCGCCGACATTAAAAAACCAATTAATTAAGGAAATTGTGGATGGTATAGCTTATACAGAGACATCATATGAGGCAATCAGAAA
GCAAACTGCAAAACTATGGGATATTGCATTAGGTAAGGTGACCAAAGATAAAATCAATAAAGAAGATACAGCAGCAGCTAGCAAATTTACTTTGAGTTATGAATATCACAGAT
TCAGGCTAATCAATATGGCAGACAATTTAATTGCTAGACTTATGGTGAAACCAAAATCAGATTGGCTAATATCAGTCATGAAGGGGCATCTTAACAGACTATATGAGCACTGGA
AAGTAATGAATGAAATTATCCTAAGTATGGACAAYGATTATTCAATTGCAACAACGTTCGAATATTATGCACCATCATGTCTGTGTTTAGCTACGCAGACTTTCCTTATTGTGAG
GAATATGGAAATGGATGATGTCAAGATGATGGTTGCAGTATATAAAAGATTTCTTAACCTAGGAATGTTTCTGCAGAGTGCCAAAGTATGCAGCCTTGCCGATAGTCATACATT
CAGAGATTTTTCTAGATCTTTTTCCTTTATTACGATAATTTCAAGATTGATGATAATCGAATTTATGCAAATTAAAGAATTGACGAAGGTAGAGTTTATTGAGAAGTTTTCTGAAG
TATGCCCTGACCTTGCAGATCTACCTCCGATGCTTCTAGATCCAAACTCTTGCTTATATTTTTCATTGTTACAGCAGATTAAGAAATCTGGTTTTACGTTGTCATTCAAAAAAAATTC
TTGAAGACGCTAGAATGATGGACTTCAATTACGACCGCAATTTGGACTCAGAGGCCATTAAAAAGTGCAATGGTGAATTTAGCAAGTCAATGCCTTCCTGTACCAATGTCTCAG
ATACCACCACCGCTGTTTCTGACAACAGTGCTAAGAAGAAAGCTTCAATGGGGTCGGCGAGGGTAAATTCAACTGATACACTAACTGCATCTCCCTTATCGGGCTTAAGGAATC
AAACGCAGTTGGATTCTAAAGACAGTGTTCCATCTCTCGAGGCTTATACACCAATTGATTCTGTCTCTGACGTACCCACTGGGGAGATCAACGTTCCATTCCCTCCTGTTTATAAT
CAAAATGGATTGGATCAGCAAACCACTTATAATTTGGGAACTTTAGATGAGTTTGTTAACAAGGGAGATTTGAATGAACTCTATAATAGCCTATGGGGTGACCTATTTTCTGAT
GTTTACTTGTGA

>83

ATGCAAACATTAGAAACTACATCAAAATCAAATCCAGGGGAAGTCAAAGCACAGAAGCCTAGTACAAGAAGAACAAAAGTTGGAAAAGCTTGTGATAGCTGTAGAAGGAGG
AAAATAAAATGTAATGGGCTAAAACCTTGTCCATCTTGTACAATCTATGGTTGTGAATGTACATATACTGATGCAAAATCGACAAAAAATCTCAAATCAAATGATGCAGGTAAA
CCAAAACCAACAGGGAGAGTATCAAAGAATAAAGAAACTACTAGAATCGACAAAGATATTAGGAAATCAGAGCAGCAGTATGTCCCTATTAATGCTAATATTCATGTTGGTCC
CAGGTTCCCCTCCGAGAATATATTGAATGGATATCCACAATGTGGAGCACCACAGAACAATGTTGTGGGTAATCCACTAGCGGTTAATCCTCAATGCCATAGAGGTCTTTCTGA
AACTCCTATGTCCTCAACATTCAAAGAATCTAACTTAAGAGATGATCGGCTACTACAGTCATCAGATACAGATGATATGAGGAATGGTGACTCGGAAGAAAGGGACTTGAAAG
GGAGTGACAGCGAGAATGTCAAAAGTAAAGACAATAAAAGTGATCCTTTGATTATATACAAAGATGATACACATATTGAAAGCACGGTTAATAAACTAACACAGGCAGTTAAT
GAACTCAAATCACTTCAAAATGCACCTAGTTCGATAAAATCATCCATTGACGCCATTGAGTTACAACTTAGAAACATTTTAGACAATTGGAAACCAGAGGTAGATTTCGAGAAA
GCAAAGATTAATGAAAGTGCCACCACTAAGTCACTTGAAACAAACTTGCTGAGGAATAAATACACTAATCACGTTCATCTAACAAGATTTAGGATATGGATAGATTATAAAAAT
GCGAACAAAAACAATCATTTTATGGGAGAGTGTGGATTTAGTCTTGCAGAATCTTTTTTTGCTTCTAATCAGCCATTGGTCGATGAATTGTTTGGGTTGTATTCCCAGGTAGAGG
CCTTTTCTTTGCAAGGTCTTGGTTACTGTGTTCACCTTTATGAGCCATATATGAAAACTGAGGAAGCGATAAAACTGATGAAAGAGACCTTATATATTATACTACGGTTTATTGA
TATATGTGTTCACCATATCAATGAAGAGTCGATATCGATTGCCAACCCGTTAGAAACATATTTACGAAAAAAACATCTAATGCCTATGACTCCTACACCAAGGTCGTCCTATGGA
AGTCCACAAAGTGCTAGTACAAAGAGCTTGGTAAGTAAGATAATAGAGAGAATACCGCAACCGTTTATTGAGAGTGTAACTAATGTGTCGAGTCTTCAACTATTAGATCTTCGA

GATGACGAGTCAAAAATGTTTGGAACATTGCTGAACATGTGTAAGTCTATAAGGCGAAAATTTGACTCTGTTATGAGCGATTACGATTCCATTGTCACAGAAAAATCCGAAGG
CGAACAAAATGATGGTAAAGTAACTGTAGCTGAGTTCACATCTTTGTGTGAAGCGGAAGAAATGCTCTTAGCATTATGCTATAACTATTATAATCTGACGTTATACAGTTTCTTT
GAATTTGGGACTAATATTGAATACATGGAACATCTGTTGCTTCTTCTTGAAGAACAGCTTGCTCTCGACGAATACTATGGTTTTGAAAAGGTCTTGAATGTAGCTGTTGCAAATG
CTAAAAAAATGGGTTTCCACCGTTGGGAGTTTTACGTCGGTTATGAAGAGTCGACTGCTGAAAAGAGGCGGCTACTATGGTGGAAGTTATACAATTATGAAAAAGCCAGTACT
ATGAAGAAGGGTTTTTTTTCTGTGATTGATGATGCTACTGTCAACTGTTTATTACCTAAGATTTTTAGAAACTTTGGCTATCTGGATAGGGTGGAGTTTCTAGAAAATATTCAAA
AGCCAATGGATCTTAGTGTGTTTTCCGATGTTCCAATTTCTGTCCTTTGTAAATACGGTGAGTTGGCCCTTACAATAGTTACCAGTGAGTTTCATGAAAAATTTTTATATGCTGAT
AGATACACTTCTATTCGAAATTCCGCGAAACCGCCGACATTAAAAAACCAATTAATTAAGGAAATTGTGGATGGTATAGCTTATACAGAGACATCATATGAGGCAATCAGAAA
GCAAACTGCAAAACTATGGGATATTGCATTAGGTAAGGTGACCAAAGATAAAATCAATAAAGAAGATACAGCAGCAGCTAGCAAATTTACTTTGAGTTATGAATATCACAGAT
TCAGGCTAATCAATATGGCAGACAATTTAATTGCTAGACTTATGGTGAAACCAAAATCAGATTGGCTAATATCAGTCATGAAGGGGCATCTTAACAGACTATATGAGCACTGGA
AAGTAATGAATGAAATTATCCTAAGTATGGACAACGATTATTCAATTGCAACAACGTTCGAATATTATGCACCATCATGTCTGTGTTTARCTACGCAGACTTTCCTTATTGTGAG
GAATATGGAAATGGATGATGTCAAGATGATGGTTGCAGTATATAAAAGATTTCTTAACCTAGGAATGTTTCTGCAGAGTGCCAAAGTATGCAGCCTTGCCGATAGTCATACATT
CAGAGATTTTTCTAGATCTTTTTCCTTTATTACGATAATTTCAAGATTGATGATAATCGAATTTATGCAAATTAAAGAATTGACGAAGGTAGAGTTTATTGAGAAGTTTTCTGAAG
TATGCCCTGACCTTGCAGATCTACCTCCGATGCTTCTAGATCCAAACTCTTGCTTATATTTTTCATTGTTACAGCAGATTAAGAAATCTGGTTTTACGTTGTCATTCAAAAAAATTC
TTGAAGACGCTAGAATGATGGACTTCAATTACGACCGCAATTTGGACTCAGAGGCCATTAAAAAGTGCAATGGTGAATTTAGCAAGTCAATGCCTTCCTGTACCAATGTCTCAG
ATACCACCACCGCTGTTTCTGACAACAGTGCTAAGAAGAAAGCTTCAATGGGGTCGGCGAGGGTAAATTCAACTGATACACTAACTGCATCTCCCTTATCGGGCTTAAGGAATC
AAACGCAGTTGGATTCTAAAGACAGTGTTCCATCTCTCGAGGCTTATACACCAATTGATTCTGTCTCTGACGTACCCACTGGGGAGATCAACGTTCCATTCCCTCCTGTTTATAAT
CAAAATGGATTGGATCAGCAAACCACTTATAATTTGGGAACTTTAGATGAGTTTGTTAACAAGGGAGATTTGAATGAACTCTATAATAGCCTATGGGGTGACCTATTTTCTGAT
GTTTACTTGTGA

HS1 FKS1 sequences

>1

TTCACTGCCTCCTTTGCACCTTTGCATGGTCTTGACAGATGGTTGTCTTACCTGGTTTGGGTTACTGTTTTTGCTGCTAAGTACGCTGAATCATACTACTTCTTGATTCTATCTCTA
AGAGATCCAATCAGAATTTTGTCTACCACTACCATGAGATGTACTGGTGAATACTGGTGGGGTTCAAAGCTATGTAGACATCAATCAAAGATTGTTTTGGGTTTGATGATTGCT
ACCGATTTTATTCTGTTCTTCTTGGATACCTATCTGTGGTACATTGTTGTCAACACTGTCTTTTCCGTTGGTAAATCTTTCTACTTGGGTATTTCT

>2

TTCACTGCCTCCTTTGCACCTTTGCATGGTCTTGACAGATGGTTGTCTTACCTGGTTTGGGTTACTGTTTTTGCTGCTAAGTACGCTGAATCATACTACTTCTTGATTCTATCTCTA
AGAGATCCAATCAGAATTTTGTCTACCACTACCATGAGATGTACTGGTGAATACTGGTGGGGTTCAAAGCTATGTAGACATCAATCAAAGATTGTTTTGGGTTTGATGATTGCT
ACCGATTTTATTCTGTTCTTCTTGGATACCTATCTGTGGTACATTGTTGTCAACACTGTCTTTTCCGTTGGTAAATCTTTCTACTTGGGTATTTCT

>3

TTCACTGCCTCCTTTGCACCTTTGCATGGTCTTGACAGATGGTTGTCTTACCTGGTTTGGGTTACTGTTTTTGCTGCTAAGTACGCTGAATCATACTACTTCTTGATTCTATCTCTA
AGAGATCCAATCAGAATTTTGTCTACCACTACCATGAGATGTACTGGTGAATACTGGTGGGGTTCAAAGCTATGTAGACATCAATCAAAGATTGTTTTGGGTTTGATGATTGCT
ACCGATTTTATTCTGTTCTTCTTGGATACCTATCTGTGGTACATTGTTGTCAACACTGTCTTTTCCGTTGGTAAATCTTTCTACTTGGGTATTTCT

>4

TTCACTGCCTCCTTTGCACCTTTGCATGGTCTTGACAGATGGTTGTCTTACCTGGTTTGGGTTACTGTTTTTGCTGCTAAGTACGCTGAATCATACTACTTTTTGATTCTATCTCTA
AGAGATCCAATCAGAATTTTGTCTACCACTACCATGAGATGTACTGGTGAATACTGGTGGGGTTCAAAGCTATGTAGACATCAATCAAAGATTGTTTTGGGTTTGATGATTGCT
ACCGATTTTATTCTGTTCTTCTTGGATACCTATCTGTGGTACATTGTTGTCAACACTGTCTTTTCCGTTGGTAAATCTTTCTACTTGGGTATTTCT

>5

TTCACTGCCTCCTTTGCACCTTTGCATGGTCTTGACAGATGGTTGTCTTACCTGGTTTGGGTTACTGTTTTTGCTGCTAAGTACGCTGAATCATACTACTTCTTGATTCTATCTCTA
AGAGATCCAATCAGAATTTTGTCTACCACTACCATGAGATGTACTGGTGAATACTGGTGGGGTTCAAAGCTATGTAGACATCAATCAAAGATTGTTTTGGGTTTGATGATTGCT
ACCGATTTTATTCTGTTCTTCTTGGATACCTATCTGTGGTACATTGTTGTCAACACTGTCTTTTCCGTTGGTAAATCTTTCTACTTGGGTATTTCT

>6

TTCACTGCCTCCTTTGCACCTTTGCATGGTCTTGACAGATGGTTGTCTTACCTGGTTTGGGTTACTGTTTTTGCTGCTAAGTACGCTGAATCATACTACTTCTTGATTCTATCTCTA
AGAGATCCAATCAGAATTTTGTCTACCACTACCATGAGATGTACTGGTGAATACTGGTGGGGTTCAAAGCTATGTAGACATCAATCAAAGATTGTTTTGGGTTTGATGATTGCT
ACCGATTTTATTCTGTTCTTCTTGGATACCTATCTGTGGTACATTGTTGTCAACACTGTCTTTTCCGTTGGTAAATCTTTCTACTTGGGTATTTCT

>7

TTCACTGCCTCCTTTGCACCTTTGCATGGTCTTGACAGATGGTTGTCTTACCTGGTTTGGGTTACTGTTTTTGCTGCTAAGTACGCTGAATCATACTACTTCTTGATTCTATCTCTA
AGAGATCCAATCAGAATTTTGTCTACCACTACCATGAGATGTACTGGTGAATACTGGTGGGGTTCAAAGCTATGTAGACATCAATCAAAGATTGTTTTGGGTTTGATGATTGCT
ACCGATTTTATTCTGTTCTTCTTGGATACCTATCTGTGGTACATTGTTGTCAACACTGTCTTTTCCGTTGGTAAATCTTTCTACTTGGGTATTTCT

>8

TTCACTGCCTCCTTTGCACCTTTGCATGGTCTTGACAGATGGTTGTCTTACCTGGTTTGGGTTACTGTTTTTGCTGCTAAGTACGCTGAATCATACTACTTCTTGATTCTATCTCTA
AGAGATCCAATCAGAATTTTGTCTACCACTACCATGAGATGTACTGGTGAATACTGGTGGGGTTCAAAGCTATGTAGACATCAATCAAAGATTGTTTTGGGTTTGATGATTGCT
ACCGATTTTATTCTGTTCTTCTTGGATACCTATCTGTGGTACATTGTTGTCAACACTGTCTTTTCCGTTGGTAAATCTTTCTACTTGGGTATTTCT

>9

TTCACTGCCTCCTTTGCACCTTTGCATGGTCTTGACAGATGGTTGTCTTACCTGGTTTGGGTTACTGTTTTTGCTGCTAAGTACGCTGAATCATACTACTTCTTGATTCTATCTCTA
AGAGATCCAATCAGAATTTTGTCTACCACTACCATGAGATGTACTGGTGAATACTGGTGGGGTTCAAAGCTATGTAGACATCAATCAAAGATTGTTTTGGGTTTGATGATTGCT
ACCGATTTTATTCTGTTCTTCTTGGATACCTATCTGTGGTACATTGTTGTCAACACTGTCTTTTCCGTTGGTAAATCTTTCTACTTGGGTATTTCT

>10

TTCACTGCCTCCTTTGCACCTTTGCATGGTCTTGACAGATGGTTGTCTTACCTGGTTTGGGTTACTGTTTTTGCTGCTAAGTACGCTGAATCATACTACTTTTTGATTCTATCTCTA
AGAGATCCAATCAGAATTTTGTCTACCACTACCATGAGATGTACTGGTGAATACTGGTGGGGTTCAAAGCTATGTAGACATCAATCAAAGATTGTTTTGGGTTTGATGATTGCT
ACCGATTTTATTCTGTTCTTCTTGGATACCTATCTGTGGTACATTGTTGTCAACACTGTCTTTTCCGTTGGTAAATCTTTCTACTTGGGTATTTCT

>11

TTCACTGCCTCCTTTGCACCTTTGCATGGTCTTGACAGATGGTTGTCTTACCTGGTTTGGGTTACTGTTTTTGCTGCTAAGTACGCTGAATCATACTACTTCTTGATTCTATCTCTA
AGAGATCCAATCAGAATTTTGTCTACCACTACCATGAGATGTACTGGTGAATACTGGTGGGGTTCAAAGCTATGTAGACATCAATCAAAGATTGTTTTGGGTTTGATGATTGCT
ACCGATTTTATTCTGTTCTTCTTGGATACCTATCTGTGGTACATTGTTGTCAACACTGTCTTTTCCGTTGGTAAATCTTTCTACTTGGGTATTTCT

>12

TTCACTGCCTCCTTTGCACCTTTGCATGGTCTTGACAGATGGTTGTCTTACCTGGTTTGGGTTACTGTTTTTGCTGCTAAGTACGCTGAATCATACTACTTTTTGATTCTATCTCTA
AGAGATCCAATCAGAATTTTGTCTACCACTACCATGAGATGTACTGGTGAATACTGGTGGGGTTCAAAGCTATGTAGACATCAATCAAAGATTGTTTTGGGTTTGATGATTGCT
ACCGATTTTATTCTGTTCTTCTTGGATACCTATCTGTGGTACATTGTTGTCAACACTGTCTTTTCCGTTGGTAAATCTTTCTACTTGGGTATTTCT

>13

TTCACTGCCTCCTTTGCACCTTTGCATGGTCTTGACAGATGGTTGTCTTACCTGGTTTGGGTTACTGTTTTTGCTGCTAAGTACGCTGAATCATACTACTTCTTGATTCTATCTCTA
AGAGATCCAATCAGAATTTTGTCTACCACTACCATGAGATGTACTGGTGAATACTGGTGGGGTTCAAAGCTATGTAGACATCAATCAAAGATTGTTTTGGGTTTGATGATTGCT
ACCGATTTTATTCTGTTCTTCTTGGATACCTATCTGTGGTACATTGTTGTCAACACTGTCTTTTCCGTTGGTAAATCTTTCTACTTGGGTATTTCT

>14

TTCACTGCCTCCTTTGCACCTTTGCATGGTCTTGACAGATGGTTGTCTTACCTGGTTTGGGTTACTGTTTTTGCTGCTAAGTACGCTGAATCATACTACTTCTTGATTCTATCTCTA
AGAGATCCAATCAGAATTTTGTCTACCACTACCATGAGATGTACTGGTGAATACTGGTGGGGTTCAAAGCTATGTAGACATCAATCAAAGATTGTTTTGGGTTTGATGATTGCT
ACCGATTTTATTCTGTTCTTCTTGGATACCTATCTGTGGTACATTGTTGTCAACACTGTCTTTTCCGTTGGTAAATCTTTCTACTTGGGTATTTCT

>15

TTCACTGCCTCCTTTGCACCTTTGCATGGTCTTGACAGATGGTTGTCTTACCTGGTTTGGGTTACTGTTTTTGCTGCTAAGTACGCTGAATCATACTACTTCTTGATTCTATCTCTA
AGAGATCCAATCAGAATTTTGTCTACCACTACCATGAGATGTACTGGTGAATACTGGTGGGGTTCAAAGCTATGTAGACATCAATCAAAGATTGTTTTGGGTTTGATGATTGCT
ACCGATTTTATTCTGTTCTTCTTGGATACCTATCTGTGGTACATTGTTGTCAACACTGTCTTTTCCGTTGGTAAATCTTTCTACTTGGGTATTTCT

>16

TTCACTGCCTCCTTTGCACCTTTGCATGGTCTTGACAGATGGTTGTCTTACCTGGTTTGGGTTACTGTTTTTGCTGCTAAGTACGCTGAATCATACTACTTCTTGATTCTATCTCTA
AGAGATCCAATCAGAATTTTGTCTACCACTACCATGAGATGTACTGGTGAATACTGGTGGGGTTCAAAGCTATGTAGACATCAATCAAAGATTGTTTTGGGTTTGATGATTGCT
ACCGATTTTATTCTGTTCTTCTTGGATACCTATCTGTGGTACATTGTTGTCAACACTGTCTTTTCCGTTGGTAAATCTTTCTACTTGGGTATTTCT

>17

TTCACTGCCTCCTTTGCACCTTTGCATGGTCTTGACAGATGGTTGTCTTACCTGGTTTGGGTTACTGTTTTTGCTGCTAAGTACGCTGAATCATACTACTTCTTGATTCTATCTCTA
AGAGATCCAATCAGAATTTTGTCTACCACTACCATGAGATGTACTGGTGAATACTGGTGGGGTTCAAAGCTATGTAGACATCAATCAAAGATTGTTTTGGGTTTGATGATTGCT
ACCGATTTTATTCTGTTCTTCTTGGATACCTATCTGTGGTACATTGTTGTCAACACTGTCTTTTCCGTTGGTAAATCTTTCTACTTGGGTATTTCT

>18

TTCACTGCCTCCTTTGCACCTTTGCATGGTCTTGACAGATGGTTGTCTTACCTGGTTTGGGTTACTGTTTTTGCTGCTAAGTACGCTGAATCATACTACTTCTTGATTCTATCTCTA
AGAGATCCAATCAGAATTTTGTCTACCACTACCATGAGATGTACTGGTGAATACTGGTGGGGTTCAAAGCTATGTAGACATCAATCAAAGATTGTTTTGGGTTTGATGATTGCT
ACCGATTTTATTCTGTTCTTCTTGGATACCTATCTGTGGTACATTGTTGTCAACACTGTCTTTTCCGTTGGTAAATCTTTCTACTTGGGTATTTCT

>19

TTCACTGCCTCCTTTGCACCTTTGCATGGTCTTGACAGATGGTTGTCTTACCTGGTTTGGGTTACTGTTTTTGCTGCTAAGTACGCTGAATCATACTACTTCTTGATTCTATCTCTA
AGAGATCCAATCAGAATTTTGTCTACCACTACCATGAGATGTACTGGTGAATACTGGTGGGGTTCAAAGCTATGTAGACATCAATCAAAGATTGTTTTGGGTTTGATGATTGCT
ACCGATTTTATTCTGTTCTTCTTGGATACCTATCTGTGGTACATTGTTGTCAACACTGTCTTTTCCGTTGGTAAATCTTTCTACTTGGGTATTTCT

>20

TTCACTGCCTCCTTTGCACCTTTGCATGGTCTTGACAGATGGTTGTCTTACCTGGTTTGGGTTACTGTTTTTGCTGCTAAGTACGCTGAATCATACTACTTCTTGATTCTATCTCTA
AGAGATCCAATCAGAATTTTGTCTACCACTACCATGAGATGTACTGGTGAATACTGGTGGGGTTCAAAGCTATGTAGACATCAATCAAAGATTGTTTTGGGTTTGATGATTGCT
ACCGATTTTATTCTGTTCTTCTTGGATACCTATCTGTGGTACATTGTTGTCAACACTGTCTTTTCCGTTGGTAAATCTTTCTACTTGGGTATTTCT

>21

TTCACTGCCTCCTTTGCACCTTTGCATGGTCTTGACAGATGGTTGTCTTACCTGGTTTGGGTTACTGTTTTTGCTGCTAAGTACGCTGAATCATACTACTTCTTGATTCTATCTCTA
AGAGATCCAATCAGAATTTTGTCTACCACTACCATGAGATGTACTGGTGAATACTGGTGGGGTTCAAAGCTATGTAGACATCAATCAAAGATTGTTTTGGGTTTGATGATTGCT
ACCGATTTTATTCTGTTCTTCTTGGATACCTATCTGTGGTACATTGTTGTCAACACTGTCTTTTCCGTTGGTAAATCTTTCTACTTGGGTATTTCT

>22

TTCACTGCCTCCTTTGCACCTTTGCATGGTCTTGACAGATGGTTGTCTTACCTGGTTTGGGTTACTGTTTTTGCTGCTAAGTACGCTGAATCATACTACTTCTTGATTCTATCTCTA
AGAGATCCAATCAGAATTTTGTCTACCACTACCATGAGATGTACTGGTGAATACTGGTGGGGTTCAAAGCTATGTAGACATCAATCAAAGATTGTTTTGGGTTTGATGATTGCT
ACCGATTTTATTCTGTTCTTCTTGGATACCTATCTGTGGTACATTGTTGTCAACACTGTCTTTTCCGTTGGTAAATCTTTCTACTTGGGTATTTCT

>23

TTCACTGCCTCCTTTGCACCTTTGCATGGTCTTGACAGATGGTTGTCTTACCTGGTTTGGGTTACTGTTTTTGCTGCTAAGTACGCTGAATCATACTACTTCTTGATTCTATCTCTA
AGAGATCCAATCAGAATTTTGTCTACCACTACCATGAGATGTACTGGTGAATACTGGTGGGGTTCAAAGCTATGTAGACATCAATCAAAGATTGTTTTGGGTTTGATGATTGCT
ACCGATTTTATTCTGTTCTTCTTGGATACCTATCTGTGGTACATTGTTGTCAACACTGTCTTTTCCGTTGGTAAATCTTTCTACTTGGGTATTTCT

>24

TTCACTGCCTCCTTTGCACCTTTGCATGGTCTTGACAGATGGTTGTCTTACCTGGTTTGGGTTACTGTTTTTGCTGCTAAGTACGCTGAATCATACTACTTTTTGATTCTATCTCTA
AGAGATCCAATCAGAATTTTGTCTACCACTACCATGAGATGTACTGGTGAATACTGGTGGGGTTCAAAGCTATGTAGACATCAATCAAAGATTGTTTTGGGTTTGATGATTGCT
ACCGATTTTATTCTGTTCTTCTTGGATACCTATCTGTGGTACATTGTTGTCAACACTGTCTTTTCCGTTGGTAAATCTTTCTACTTGGGTATTTCT

>25

TTCACTGCCTCCTTTGCACCTTTGCATGGTCTTGACAGATGGTTGTCTTACCTGGTTTGGGTTACTGTTTTTGCTGCTAAGTACGCTGAATCATACTACTTTTTGATTCTATCTCTA
AGAGATCCAATCAGAATTTTGTCTACCACTACCATGAGATGTACTGGTGAATACTGGTGGGGTTCAAAGCTATGTAGACATCAATCAAAGATTGTTTTGGGTTTGATGATTGCT
ACCGATTTTATTCTGTTCTTCTTGGATACCTATCTGTGGTACATTGTTGTCAACACTGTCTTTTCCGTTGGTAAATCTTTCTACTTGGGTATTTCT

>26

TTCACTGCCTCCTTTGCACCTTTGCATGGTCTTGACAGATGGTTGTCTTACCTGGTTTGGGTTACTGTTTTTGCTGCTAAGTACGCTGAATCATACTACTTCTTGATTCTATCTCTA
AGAGATCCAATCAGAATTTTGTCTACCACTACCATGAGATGTACTGGTGAATACTGGTGGGGTTCAAAGCTATGTAGACATCAATCAAAGATTGTTTTGGGTTTGATGATTGCT
ACCGATTTTATTCTGTTCTTCTTGGATACCTATCTGTGGTACATTGTTGTCAACACTGTCTTTTCCGTTGGTAAATCTTTCTACTTGGGTATTTCT

>27

TTCACTGCCTCCTTTGCACCTTTGCATGGTCTTGACAGATGGTTGTCTTACCTGGTTTGGGTTACTGTTTTTGCTGCTAAGTACGCTGAATCATACTACTTCTTGATTCTATCTCTA
AGAGATCCAATCAGAATTTTGTCTACCACTACCATGAGATGTACTGGTGAATACTGGTGGGGTTCAAAGCTATGTAGACATCAATCAAAGATTGTTTTGGGTTTGATGATTGCT
ACCGATTTTATTCTGTTCTTCTTGGATACCTATCTGTGGTACATTGTTGTCAACACTGTCTTTTCCGTTGGTAAATCTTTCTACTTGGGTATTTCT

>28

TTCACTGCCTCCTTTGCACCTTTGCATGGTCTTGACAGATGGTTGTCTTACCTGGTTTGGGTTACTGTTTTTGCTGCTAAGTACGCTGAATCATACTACTTCTTGATTCTATCTCTA
AGAGATCCAATCAGAATTTTGTCTACCACTACCATGAGATGTACTGGTGAATACTGGTGGGGTTCAAAGCTATGTAGACATCAATCAAAGATTGTTTTGGGTTTGATGATTGCT
ACCGATTTTATTCTGTTCTTCTTGGATACCTATCTGTGGTACATTGTTGTCAACACTGTCTTTTCCGTTGGTAAATCTTTCTACTTGGGTATTTCT

>29

TTCACTGCCTCCTTTGCACCTTTGCATGGTCTTGACAGATGGTTGTCTTACCTGGTTTGGGTTACTGTTTTTGCTGCTAAGTACGCTGAATCATACTACTTCTTGATTCTATCTCTA
AGAGATCCAATCAGAATTTTGTCTACCACTACCATGAGATGTACTGGTGAATACTGGTGGGGTTCAAAGCTATGTAGACATCAATCAAAGATTGTTTTGGGTTTGATGATTGCT
ACCGATTTTATTCTGTTCTTCTTGGATACCTATCTGTGGTACATTGTTGTCAACACTGTCTTTTCCGTTGGTAAATCTTTCTACTTGGGTATTTCT

>30

TTCACTGCCTCCTTTGCACCTTTGCATGGTCTTGACAGATGGTTGTCTTACCTGGTTTGGGTTACTGTTTTTGCTGCTAAGTACGCTGAATCATACTACTTCTTGATTCTATCTCTA
AGAGATCCAATCAGAATTTTGTCTACCACTACCATGAGATGTACTGGTGAATACTGGTGGGGTTCAAAGCTATGTAGACATCAATCAAAGATTGTTTTGGGTTTGATGATTGCT
ACCGATTTTATTCTGTTCTTCTTGGATACCTATCTGTGGTACATTGTTGTCAACACTGTCTTTTCCGTTGGTAAATCTTTCTACTTGGGTATTTCT

>31

TTCACTGCCTCCTTTGCACCTTTGCATGGTCTTGACAGATGGTTGTCTTACCTGGTTTGGGTTACTGTTTTTGCTGCTAAGTACGCTGAATCATACTACTTCTTGATTCTATCTCTA
AGAGATCCAATCAGAATTTTGTCTACCACTACCATGAGATGTACTGGTGAATACTGGTGGGGTTCAAAGCTATGTAGACATCAATCAAAGATTGTTTTGGGTTTGATGATTGCT
ACCGATTTTATTCTGTTCTTCTTGGATACCTATCTGTGGTACATTGTTGTCAACACTGTCTTTTCCGTTGGTAAATCTTTCTACTTGGGTATTTCT

>32

TTCACTGCCTCCTTTGCACCTTTGCATGGTCTTGACAGATGGTTGTCTTACCTGGTTTGGGTTACTGTTTTTGCTGCTAAGTACGCTGAATCATACTACTTCTTGATTCTATCTCTA
AGAGATCCAATCAGAATTTTGTCTACCACTACCATGAGATGTACTGGTGAATACTGGTGGGGTTCAAAGCTATGTAGACATCAATCAAAGATTGTTTTGGGTTTGATGATTGCT
ACCGATTTTATTCTGTTCTTCTTGGATACCTATCTGTGGTACATTGTTGTCAACACTGTCTTTTCCGTTGGTAAATCTTTCTACTTGGGTATTTCT

>33

TTCACTGCCTCCTTTGCACCTTTGCATGGTCTTGACAGATGGTTGTCTTACCTGGTTTGGGTTACTGTTTTTGCTGCTAAGTACGCTGAATCATACTACTTCTTGATTCTATCTCTA
AGAGATCCAATCAGAATTTTGTCTACCACTACCATGAGATGTACTGGTGAATACTGGTGGGGTTCAAAGCTATGTAGACATCAATCAAAGATTGTTTTGGGTTTGATGATTGCT
ACCGATTTTATTCTGTTCTTCTTGGATACCTATCTGTGGTACATTGTTGTCAACACTGTCTTTTCCGTTGGTAAATCTTTCTACTTGGGTATTTCT

>34

TTCACTGCCTCCTTTGCACCTTTGCATGGTCTTGACAGATGGTTGTCTTACCTGGTTTGGGTTACTGTTTTTGCTGCTAAGTACGCTGAATCATACTACTTTTTGATTCTATCTCTA
AGAGATCCAATCAGAATTTTGTCTACCACTACCATGAGATGTACTGGTGAATACTGGTGGGGTTCAAAGCTATGTAGACATCAATCAAAGATTGTTTTGGGTTTGATGATTGCT
ACCGATTTTATTCTGTTCTTCTTGGATACCTATCTGTGGTACATTGTTGTCAACACTGTCTTTTCCGTTGGTAAATCTTTCTACTTGGGTATTTCT

>35

TTCACTGCCTCCTTTGCACCTTTGCATGGTCTTGACAGATGGTTGTCTTACCTGGTTTGGGTTACTGTTTTTGCTGCTAAGTACGCTGAATCATACTACTTTTTGATTCTATCTCTA
AGAGATCCAATCAGAATTTTGTCTACCACTACCATGAGATGTACTGGTGAATACTGGTGGGGTTCAAAGCTATGTAGACATCAATCAAAGATTGTTTTGGGTTTGATGATTGCT
ACCGATTTTATTCTGTTCTTCTTGGATACCTATCTGTGGTACATTGTTGTCAACACTGTCTTTTCCGTTGGTAAATCTTTCTACTTGGGTATTTCT

>36

TTCACTGCCTCCTTTGCACCTTTGCATGGTCTTGACAGATGGTTGTCTTACCTGGTTTGGGTTACTGTTTTTGCTGCTAAGTACGCTGAATCATACTACTTCTTGATTCTATCTCTA
AGAGATCCAATCAGAATTTTGTCTACCACTACCATGAGATGTACTGGTGAATACTGGTGGGGTTCAAAGCTATGTAGACATCAATCAAAGATTGTTTTGGGTTTGATGATTGCT
ACCGATTTTATTCTGTTCTTCTTGGATACCTATCTGTGGTACATTGTTGTCAACACTGTCTTTTCCGTTGGTAAATCTTTCTACTTGGGTATTTCT

>37

TTCACTGCCTCCTTTGCACCTTTGCATGGTCTTGACAGATGGTTGTCTTACCTGGTTTGGGTTACTGTTTTTGCTGCTAAGTACGCTGAATCATACTACTTCTTGATTCTATCTCTA
AGAGATCCAATCAGAATTTTGTCTACCACTACCATGAGATGTACTGGTGAATACTGGTGGGGTTCAAAGCTATGTAGACATCAATCAAAGATTGTTTTGGGTTTGATGATTGCT
ACCGATTTTATTCTGTTCTTCTTGGATACCTATCTGTGGTACATTGTTGTCAACACTGTCTTTTCCGTTGGTAAATCTTTCTACTTGGGTATTTCT

>38

TTCACTGCCTCCTTTGCACCTTTGCATGGTCTTGACAGATGGTTGTCTTACCTGGTTTGGGTTACTGTTTTTGCTGCTAAGTACGCTGAATCATACTACTTCTTGATTCTATCTCTA
AGAGATCCAATCAGAATTTTGTCTACCACTACCATGAGATGTACTGGTGAATACTGGTGGGGTTCAAAGCTATGTAGACATCAATCAAAGATTGTTTTGGGTTTGATGATTGCT
ACCGATTTTATTCTGTTCTTCTTGGATACCTATCTGTGGTACATTGTTGTCAACACTGTCTTTTCCGTTGGTAAATCTTTCTACTTGGGTATTTCT

>39

TTCACTGCCTCCTTTGCACCTTTGCATGGTCTTGACAGATGGTTGTCTTACCTGGTTTGGGTTACTGTTTTTGCTGCTAAGTACGCTGAATCATACTACTTCTTGATTCTATCTCTA
AGAGATCCAATCAGAATTTTGTCTACCACTACCATGAGATGTACTGGTGAATACTGGTGGGGTTCAAAGCTATGTAGACATCAATCAAAGATTGTTTTGGGTTTGATGATTGCT
ACCGATTTTATTCTGTTCTTCTTGGATACCTATCTGTGGTACATTGTTGTCAACACTGTCTTTTCCGTTGGTAAATCTTTCTACTTGGGTATTTCT

>40

TTCACTGCCTCCTTTGCACCTTTGCATGGTCTTGACAGATGGTTGTCTTACCTGGTTTGGGTTACTGTTTTTGCTGCTAAGTACGCTGAATCATACTACTTCTTGATTCTATCTCTA
AGAGATCCAATCAGAATTTTGTCTACCACTACCATGAGATGTACTGGTGAATACTGGTGGGGTTCAAAGCTATGTAGACATCAATCAAAGATTGTTTTGGGTTTGATGATTGCT
ACCGATTTTATTCTGTTCTTCTTGGATACCTATCTGTGGTACATTGTTGTCAACACTGTCTTTTCCGTTGGTAAATCTTTCTACTTGGGTATTTCT

>41

TTCACTGCCTCCTTTGCACCTTTGCATGGTCTTGACAGATGGTTGTCTTACCTGGTTTGGGTTACTGTTTTTGCTGCTAAGTACGCTGAATCATACTACTTCTTGATTCTATCTCTA
AGAGATCCAATCAGAATTTTGTCTACCACTACCATGAGATGTACTGGTGAATACTGGTGGGGTTCAAAGCTATGTAGACATCAATCAAAGATTGTTTTGGGTTTGATGATTGCT
ACCGATTTTATTCTGTTCTTCTTGGATACCTATCTGTGGTACATTGTTGTCAACACTGTCTTTTCCGTTGGTAAATCTTTCTACTTGGGTATTTCT

>42

TTCACTGCCTCCTTTGCACCTTTGCATGGTCTTGACAGATGGTTGTCTTACCTGGTTTGGGTTACTGTTTTTGCTGCTAAGTACGCTGAATCATACTACTTCTTGATTCTATCTCTA
AGAGATCCAATCAGAATTTTGTCTACCACTACCATGAGATGTACTGGTGAATACTGGTGGGGTTCAAAGCTATGTAGACATCAATCAAAGATTGTTTTGGGTTTGATGATTGCT
ACCGATTTTATTCTGTTCTTCTTGGATACCTATCTGTGGTACATTGTTGTCAACACTGTCTTTTCCGTTGGTAAATCTTTCTACTTGGGTATTTCT

>43

TTCACTGCCTCCTTTGCACCTTTGCATGGTCTTGACAGATGGTTGTCTTACCTGGTTTGGGTTACTGTTTTTGCTGCTAAGTACGCTGAATCATACTACTTTTTGATTCTATCTCTA
AGAGATCCAATCAGAATTTTGTCTACCACTACCATGAGATGTACTGGTGAATACTGGTGGGGTTCAAAGCTATGTAGACATCAATCAAAGATTGTTTTGGGTTTGATGATTGCT
ACCGATTTTATTCTGTTCTTCTTGGATACCTATCTGTGGTACATTGTTGTCAACACTGTCTTTTCCGTTGGTAAATCTTTCTACTTGGGTATTTCT

>44

TTCACTGCCTCCTTTGCACCTTTGCATGGTCTTGACAGATGGTTGTCTTACCTGGTTTGGGTTACTGTTTTTGCTGCTAAGTACGCTGAATCATACTACTTCTTGATTCTATCTCTA
AGAGATCCAATCAGAATTTTGTCTACCACTACCATGAGATGTACTGGTGAATACTGGTGGGGTTCAAAGCTATGTAGACATCAGTCAAAGATTGTTTTGGGTTTGATGATTGCT
ACCGATTTTATTCTGTTCTTCTTGGATACCTATCTGTGGTACATTGTTGTCAACACTGTCTTTTCCGTTGGTAAATCTTTCTACTTGGGTATTTCT

>45

TTCACTGCCTCCTTTGCACCTTTGCATGGTCTTGACAGATGGTTGTCTTACCTGGTTTGGGTTACTGTTTTTGCTGCTAAGTACGCTGAATCATACTACTTCTTGATTCTATCTCTA
AGAGATCCAATCAGAATTTTGTCTACCACTACCATGAGATGTACTGGTGAATACTGGTGGGGTTCAAAGCTATGTAGACATCAATCAAAGATTGTTTTGGGTTTGATGATTGCT
ACCGATTTTATTCTGTTCTTCTTGGATACCTATCTGTGGTACATTGTTGTCAACACTGTCTTTTCCGTTGGTAAATCTTTCTACTTGGGTATTTCT

>46

TTCACTGCCTCCTTTGCACCTTTGCATGGTCTTGACAGATGGTTGTCTTACCTGGTTTGGGTTACTGTTTTTGCTGCTAAGTACGCTGAATCATACTACTTCTTGATTCTATCTCTA
AGAGATCCAATCAGAATTTTGTCTACCACTACCATGAGATGTACTGGTGAATACTGGTGGGGTTCAAAGCTATGTAGACATCAATCAAAGATTGTTTTGGGTTTGATGATTGCT
ACCGATTTTATTCTGTTCTTCTTGGATACCTATCTGTGGTACATTGTTGTCAACACTGTCTTTTCCGTTGGTAAATCTTTCTACTTGGGTATTTCT

>47

TTCACTGCCTCCTTTGCACCTTTGCATGGTCTTGACAGATGGTTGTCTTACCTGGTTTGGGTTACTGTTTTTGCTGCTAAGTACGCTGAATCATACTACTTCTTGATTCTATCTCTA
AGAGATCCAATCAGAATTTTGTCTACCACTACCATGAGATGTACTGGTGAATACTGGTGGGGTTCAAAGCTATGTAGACATCAATCAAAGATTGTTTTGGGTTTGATGATTGCT
ACCGATTTTATTCTGTTCTTCTTGGATACCTATCTGTGGTACATTGTTGTCAACACTGTCTTTTCCGTTGGTAAATCTTTCTACTTGGGTATTTCT

>48

TTCACTGCCTCCTTTGCACCTTTGCATGGTCTTGACAGATGGTTGTCTTACCTGGTTTGGGTTACTGTTTTTGCTGCTAAGTACGCTGAATCATACTACTTCTTGATTCTATCTCTA
AGAGATCCAATCAGAATTTTGTCTACCACTACCATGAGATGTACTGGTGAATACTGGTGGGGTTCAAAGCTATGTAGACATCAATCAAAGATTGTTTTGGGTTTGATGATTGCT
ACCGATTTTATTCTGTTCTTCTTGGATACCTATCTGTGGTACATTGTTGTCAACACTGTCTTTTCCGTTGGTAAATCTTTCTACTTGGGTATTTCT

>49

TTCACTGCCTCCTTTGCACCTTTGCATGGTCTTGACAGATGGTTGTCTTACCTGGTTTGGGTTACTGTTTTTGCTGCTAAGTACGCTGAATCATACTACTTCTTGATTCTATCTCTA
AGAGATCCAATCAGAATTTTGTCTACCACTACCATGAGATGTACTGGTGAATACTGGTGGGGTTCAAAGCTATGTAGACATCAATCAAAGATTGTTTTGGGTTTGATGATTGCT
ACCGATTTTATTCTGTTCTTCTTGGATACCTATCTGTGGTACATTGTTGTCAACACTGTCTTTTCCGTTGGTAAATCTTTCTACTTGGGTATTTCT

>50

TTCACTGCCTCCTTTGCACCTTTGCATGGTCTTGACAGATGGTTGTCTTACCTGGTTTGGGTTACTGTTTTTGCTGCTAAGTACGCTGAATCATACTACTTCTTGATTCTATCTCTA
AGAGATCCAATCAGAATTTTGTCTACCACTACCATGAGATGTACTGGTGAATACTGGTGGGGTTCAAAGCTATGTAGACATCAATCAAAGATTGTTTTGGGTTTGATGATTGCT
ACCGATTTTATTCTGTTCTTCTTGGATACCTATCTGTGGTACATTGTTGTCAACACTGTCTTTTCCGTTGGTAAATCTTTCTACTTGGGTATTTCT

>51

TTCACTGCCTCCTTTGCACCTTTGCATGGTCTTGACAGATGGTTGTCTTACCTGGTTTGGGTTACTGTTTTTGCTGCTAAGTACGCTGAATCATACTACTTCTTGATTCTATCTCTA
AGAGATCCAATCAGAATTTTGTCTACCACTACCATGAGATGTACTGGTGAATACTGGTGGGGTTCAAAGCTATGTAGACATCAATCAAAGATTGTTTTGGGTTTGATGATTGCT
ACCGATTTTATTCTGTTCTTCTTGGATACCTATCTGTGGTACATTGTTGTCAACACTGTCTTTTCCGTTGGTAAATCTTTCTACTTGGGTATTTCT

>52

TTCACTGCCTCCTTTGCACCTTTGCATGGTCTTGACAGATGGTTGTCTTACCTGGTTTGGGTTACTGTTTTTGCTGCTAAGTACGCTGAATCATACTACTTCTTGATTCTATCTCTA
AGAGATCCAATCAGAATTTTGTCTACCACTACCATGAGATGTACTGGTGAATACTGGTGGGGTTCAAAGCTATGTAGACATCAATCAAAGATTGTTTTGGGTTTGATGATTGCT
ACCGATTTTATTCTGTTCTTCTTGGATACCTATCTGTGGTACATTGTTGTCAACACTGTCTTTTCCGTTGGTAAATCTTTCTACTTGGGTATTTCT

>53

TTCACTGCCTCCTTTGCACCTTTGCATGGTCTTGACAGATGGTTGTCTTACCTGGTTTGGGTTACTGTTTTTGCTGCTAAGTACGCTGAATCATACTACTTCTTGATTCTATCTCTA
AGAGATCCAATCAGAATTTTGTCTACCACTACCATGAGATGTACTGGTGAATACTGGTGGGGTTCAAAGCTATGTAGACATCAATCAAAGATTGTTTTGGGTTTGATGATTGCT
ACCGATTTTATTCTGTTCTTCTTGGATACCTATCTGTGGTACATTGTTGTCAACACTGTCTTTTCCGTTGGTAAATCTTTCTACTTGGGTATTTCT

>54

TTCACTGCCTCCTTTGCACCTTTGCATGGTCTTGACAGATGGTTGTCTTACCTGGTTTGGGTTACTGTTTTTGCTGCTAAGTACGCTGAATCATACTACTTCTTGATTCTATCTCTA
AGAGATCCAATCAGAATTTTGTCTACCACTACCATGAGATGTACTGGTGAATACTGGTGGGGTTCAAAGCTATGTAGACATCAATCAAAGATTGTTTTGGGTTTGATGATTGCT
ACCGATTTTATTCTGTTCTTCTTGGATACCTATCTGTGGTACATTGTTGTCAACACTGTCTTTTCCGTTGGTAAATCTTTCTACTTGGGTATTTCT

>55

TTCACTGCCTCCTTTGCACCTTTGCATGGTCTTGACAGATGGTTGTCTTACCTGGTTTGGGTTACTGTTTTTGCTGCTAAGTACGCTGAATCATACTACTTCTTGATTCTATCTCTA
AGAGATCCAATCAGAATTTTGTCTACCACTACCATGAGATGTACTGGTGAATACTGGTGGGGTTCAAAGCTATGTAGACATCAATCAAAGATTGTTTTGGGTTTGATGATTGCT
ACCGATTTTATTCTGTTCTTCTTGGATACCTATCTGTGGTACATTGTTGTCAACACTGTCTTTTCCGTTGGTAAATCTTTCTACTTGGGTATTTCT

>56

TTCACTGCCTCCTTTGCACCTTTGCATGGTCTTGACAGATGGTTGTCTTACCTGGTTTGGGTTACTGTTTTTGCTGCTAAGTACGCTGAATCATACTACTTCTTGATTCTATCTCTA
AGAGATCCAATCAGAATTTTGTCTACCACTACCATGAGATGTACTGGTGAATACTGGTGGGGTTCAAAGCTATGTAGACATCAATCAAAGATTGTTTTGGGTTTGATGATTGCT
ACCGATTTTATTCTGTTCTTCTTGGATACCTATCTGTGGTACATTGTTGTCAACACTGTCTTTTCCGTTGGTAAATCTTTCTACTTGGGTATTTCT

>57

TTCACTGCCTCCTTTGCACCTTTGCATGGTCTTGACAGATGGTTGTCTTACCTGGTTTGGGTTACTGTTTTTGCTGCTAAGTACGCTGAATCATACTACTTCTTGATTCTATCTCTA
AGAGATCCAATCAGAATTTTGTCTACCACTACCATGAGATGTACTGGTGAATACTGGTGGGGTTCAAAGCTATGTAGACATCAATCAAAGATTGTTTTGGGTTTGATGATTGCT
ACCGATTTTATTCTGTTCTTCTTGGATACCTATCTGTGGTACATTGTTGTCAACACTGTCTTTTCCGTTGGTAAATCTTTCTACTTGGGTATTTCT

>58

TTCACTGCCTCCTTTGCACCTTTGCATGGTCTTGACAGATGGTTGTCTTACCTGGTTTGGGTTACTGTTTTTGCTGCTAAGTACGCTGAATCATACTACTTCTTGATTCTATCTCTA
AGAGATCCAATCAGAATTTTGTCTACCACTACCATGAGATGTACTGGTGAATACTGGTGGGGTTCAAAGCTATGTAGACATCAATCAAAGATTGTTTTGGGTTTGATGATTGCT
ACCGATTTTATTCTGTTCTTCTTGGATACCTATCTGTGGTACATTGTTGTCAACACTGTCTTTTCCGTTGGTAAATCTTTCTACTTGGGTATTTCT

>71

TTCACTGCCTCCTTTGCACCTTTGCATGGTCTTGACAGATGGTTGTCTTACCTGGTTTGGGTTACTGTTTTTGCTGCTAAGTACGCTGAATCATACTACTTCTTGATTCTATCTCTA
AGAGATCCAATCAGAATTTTGTCTACCACTACCATGAGATGTACTGGTGAATACTGGTGGGGTTCAAAGCTATGTAGACATCAATCAAAGATTGTTTTGGGTTTGATGATTGCT
ACCGATTTTATTCTGTTCTTCTTGGATACCTATCTGTGGTACATTGTTGTCAACACTGTCTTTTCCGTTGGTAAATCTTTCTACTTGGGTATTTCT

>72

TTCACTGCCTCCTTTGCACCTTTGCATGGTCTTGACAGATGGTTGTCTTACCTGGTTTGGGTTACTGTTTTTGCTGCTAAGTACGCTGAATCATACTACTTCTTGATTCTATCTCTA
AGAGATCCAATCAGAATTTTGTCTACCACTACCATGAGATGTACTGGTGAATACTGGTGGGGTTCAAAGCTATGTAGACATCAATCAAAGATTGTTTTGGGTTTGATGATTGCT
ACCGATTTTATTCTGTTCTTCTTGGATACCTATCTGTGGTACATTGTTGTCAACACTGTCTTTTCCGTTGGTAAATCTTTCTACTTGGGTATTTCT

>73

TTCACTGCCTCCTTTGCACCTTTGCATGGTCTTGACAGATGGTTGTCTTACCTGGTTTGGGTTACTGTTTTTGCTGCTAAGTACGCTGAATCATACTACTTCTTGATTCTATCTCTA
AGAGATCCAATCAGAATTTTGTCTACCACTACCATGAGATGTACTGGTGAATACTGGTGGGGTTCAAAGCTATGTAGACATCAATCAAAGATTGTTTTGGGTTTGATGATTGCT
ACCGATTTTATTCTGTTCTTCTTGGATACCTATCTGTGGTACATTGTTGTCAACACTGTCTTTTCCGTTGGTAAATCTTTCTACTTGGGTATTTCT

>74

TTCACTGCCTCCTTTGCACCTTTGCATGGTCTTGACAGATGGTTGTCTTACCTGGTTTGGGTTACTGTTTTTGCTGCTAAGTACGCTGAATCATACTACTTCTTGATTCTATCTCTA
AGAGATCCAATCAGAATTTTGTCTACCACTACCATGAGATGTACTGGTGAATACTGGTGGGGTTCAAAGCTATGTAGACATCAATCAAAGATTGTTTTGGGTTTGATGATTGCT
ACCGATTTTATTCTGTTCTTCTTGGATACCTATCTGTGGTACATTGTTGTCAACACTGTCTTTTCCGTTGGTAAATCTTTCTACTTGGGTATTTCT

>75

TTCACTGCCTCCTTTGCACCTTTGCATGGTCTTGACAGATGGTTGTCTTACCTGGTTTGGGTTACTGTTTTTGCTGCTAAGTACGCTGAATCATACTACTTCTTGATTCTATCTCTA
AGAGATCCAATCAGAATTTTGTCTACCACTACCATGAGATGTACTGGTGAATACTGGTGGGGTTCAAAGCTATGTAGACATCAATCAAAGATTGTTTTGGGTTTGATGATTGCT
ACCGATTTTATTCTGTTCTTCTTGGATACCTATCTGTGGTACATTGTTGTCAACACTGTCTTTTCCGTTGGTAAATCTTTCTACTTGGGTATTTCT

>76

TTCACTGCCTCCTTTGCACCTTTGCATGGTCTTGACAGATGGTTGTCTTACCTGGTTTGGGTTACTGTTTTTGCTGCTAAGTACGCTGAATCATACTACTTCTTGATTCTATCTCTA
AGAGATCCAATCAGAATTTTGTCTACCACTACCATGAGATGTACTGGTGAATACTGGTGGGGTTCAAAGCTATGTAGACATCAATCAAAGATTGTTTTGGGTTTGATGATTGCT
ACCGATTTTATTCTGTTCTTCTTGGATACCTATCTGTGGTACATTGTTGTCAACACTGTCTTTTCCGTTGGTAAATCTTTCTACTTGGGTATTTCT

>77

TTCACTGCCTCCTTTGCACCTTTGCATGGTCTTGACAGATGGTTGTCTTACCTGGTTTGGGTTACTGTTTTTGCTGCTAAGTACGCTGAATCATACTACTTCTTGATTCTATCTCTA
AGAGATCCAATCAGAATTTTGTCTACCACTACCATGAGATGTACTGGTGAATACTGGTGGGGTTCAAAGCTATGTAGACATCAATCAAAGATTGTTTTGGGTTTGATGATTGCT
ACCGATTTTATTCTGTTCTTCTTGGATACCTATCTGTGGTACATTGTTGTCAACACTGTCTTTTCCGTTGGTAAATCTTTCTACTTGGGTATTTCT

>78

TTCACTGCCTCCTTTGCACCTTTGCATGGTCTTGACAGATGGTTGTCTTACCTGGTTTGGGTTACTGTTTTTGCTGCTAAGTACGCTGAATCATACTACTTCTTGATTCTATCTCTA
AGAGATCCAATCAGAATTTTGTCTACCACTACCATGAGATGTACTGGTGAATACTGGTGGGGTTCAAAGCTATGTAGACATCAATCAAAGATTGTTTTGGGTTTGATGATTGCT
ACCGATTTTATTCTGTTCTTCTTGGATACCTATCTGTGGTACATTGTTGTCAACACTGTCTTTTCCGTTGGTAAATCTTTCTACTTGGGTATTTCT

>79

TTCACTGCCTCCTTTGCACCTTTGCATGGTCTTGACAGATGGTTGTCTTACCTGGTTTGGGTTACTGTTTTTGCTGCTAAGTACGCTGAATCATACTACTTCTTGATTCTATCTCTA
AGAGATCCAATCAGAATTTTGTCTACCACTACCATGAGATGTACTGGTGAATACTGGTGGGGTTCAAAGCTATGTAGACATCAATCAAAGATTGTTTTGGGTTTGATGATTGCT
ACCGATTTTATTCTGTTCTTCTTGGATACCTATCTGTGGTACATTGTTGTCAACACTGTCTTTTCCGTTGGTAAATCTTTCTACTTGGGTATTTCT

>80

TTCACTGCCTCCTTTGCACCTTTGCATGGTCTTGACAGATGGTTGTCTTACCTGGTTTGGGTTACTGTTTTTGCTGCTAAGTACGCTGAATCATACTACTTCTTGATTCTATCTCTA
AGAGATCCAATCAGAATTTTGTCTACCACTACCATGAGATGTACTGGTGAATACTGGTGGGGTTCAAAGCTATGTAGACATCAATCAAAGATTGTTTTGGGTTTGATGATTGCT
ACCGATTTTATTCTGTTCTTCTTGGATACCTATCTGTGGTACATTGTTGTCAACACTGTCTTTTCCGTTGGTAAATCTTTCTACTTGGGTATTTCT

>81

TTCACTGCCTCCTTTGCACCTTTGCATGGTCTTGACAGATGGTTGTCTTACCTGGTTTGGGTTACTGTTTTTGCTGCTAAGTACGCTGAATCATACTACTTCTTGATTCTATCTCTA
AGAGATCCAATCAGAATTTTGTCTACCACTACCATGAGATGTACTGGTGAATACTGGTGGGGTTCAAAGCTATGTAGACATCAATCAAAGATTGTTTTGGGTTTGATGATTGCT
ACCGATTTTATTCTGTTCTTCTTGGATACCTATCTGTGGTACATTGTTGTCAACACTGTCTTTTCCGTTGGTAAATCTTTCTACTTGGGTATTTCT

>82

TTCACTGCCTCCTTTGCACCTTTGCATGGTCTTGACAGATGGTTGTCTTACCTGGTTTGGGTTACTGTTTTTGCTGCTAAGTACGCTGAATCATACTACTTCTTGATTCTATCTCTA
AGAGATCCAATCAGAATTTTGTCTACCACTACCATGAGATGTACTGGTGAATACTGGTGGGGTTCAAAGCTATGTAGACATCAATCAAAGATTGTTTTGGGTTTGATGATTGCT
ACCGATTTTATTCTGTTCTTCTTGGATACCTATCTGTGGTACATTGTTGTCAACACTGTCTTTTCCGTTGGTAAATCTTTCTACTTGGGTATTTCT

>83 (SU-261)

TTCACTGCCTCCTTTGCACCTTTGCATGGTCTTGACAGATGGTTGTCTTACCTGGTTTGGGTTACTGTTTTTGCTGCTAAGTACGCTGAATCATACTACTTCTTGATTCTATCTCTA
AGAGATCCAATCAGAATTTTGTCTACCACTACCATGAGATGTACTGGTGAATACTGGTGGGGTTCAAAGCTATGTAGACATCAATCAAAGATTGTTTTGGGTTTGATGATTGCT
ACCGATTTTATTCTGTTCTTCTTGGATACCTATCTGTGGTACATTGTTGTCAACACTGTCTTTTCCGTTGGTAAATCTTTCTACTTGGGTATTTCT

HS1 FKS2 sequences

>1

GTGGCCACTGTTTTATTCTTCTCGATTATGCCATTAGGTGGTCTTTTCACCTCATATATGCAAAAATCAAGTAGAAGATATGTTGCTTCTCAGACTTTCACCGCATCTTTTGCCCCA
TTACAAGGTTTGGATAGATGGTTATCTTATTTAGTTTGGGTTACTGTTTTTGCTGCCAAGTACTCTGAATCGTACTTCTTCTTGATTTTGTCTCTAAGAGACCCTATCAGAATTTTA
TCAACTACTACCATGAGATGTACTGGTGAGTATTGGTGGGGTTCAAAGTTATGTAGACATCAATCGAAGATTGTTTTAGGTTTCATGATTGCTACAGATTTCATTCTGTTCTTCCT
TGATACTTATTTGTGGTACATTGTTGTCAACACTGTCTTCTCTGTTGGTAAATCCTTCTATTTAGGTATTTCCATCTTAACTCCTTGGAGAAATATCTTTACAAGATTACCAAAGAG
AATTTACTCAAAGATTTTGGCCACAACTGATATGGAGATTAAA

>2

GTGGCCACTGTTTTATTCTTCTCGATTATGCCATTAGGTGGTCTTTTCACCTCATATATGCAAAAATCAAGTAGAAGATATGTTGCTTCTCAGACTTTCACCGCATCTTTTGCCCCA
TTACAAGGTTTGGATAGATGGTTATCTTATTTAGTTTGGGTTACTGTTTTTGCTGCCAAGTACTCTGAATCGTACTTCTTCTTGATTTTGTCTCTAAGAGACCCTATCAGAATTTTA

TCAACTACTACCATGAGATGTACTGGTGAGTATTGGTGGGGTTCAAAGTTATGTAGACATCAATCGAAGATTGTTTTAGGTTTCATGATTGCTACAGATTTCATTCTGTTCTTCCT
TGATACTTATTTGTGGTACATTGTTGTCAACACTGTCTTCTCTGTTGGTAAATCCTTCTATTTAGGTATTTCCATCTTAACTCCTTGGAGAAATATCTTTACAAGATTACCAAAGAG
AATTTACTCAAAGATTTTGGCCACAACTGATATGGAGATTAAA

>3

GTGGCCACTGTTTTATTCTTCTCGATTATGCCATTAGGTGGTCTTTTCACCTCATATATGCAAAAATCAAGTAGAAGATATGTTGCTTCTCAGACTTTCACCGCATCTTTTGCCCCA
TTACAAGGTTTGGATAGATGGTTATCTTATTTAGTTTGGGTTACTGTTTTTGCTGCCAAGTACTCTGAATCGTACTTCTTCTTGATTTTGTCTCTAAGAGACCCTATCAGAATTTTA
TCAACTACTACCATGAGATGTACTGGTGAGTATTGGTGGGGTTCAAAGTTATGTAGACATCAATCGAAGATTGTTTTAGGTTTCATGATTGCTACAGATTTCATTCTGTTCTTCCT
TGATACTTATTTGTGGTACATTGTTGTCAACACTGTCTTCTCTGTTGGTAAATCCTTCTATTTAGGTATTTCCATCTTAACTCCTTGGAGAAATATCTTTACAAGATTACCAAAGAG
AATTTACTCAAAGATTTTGGCCACAACTGATATGGAGATTAAA

>4

GTGGCCACTGTTTTATTCTTCTCGATTATGCCATTAGGTGGTCTTTTCACCTCATATATGCAAAAATCAAGTAGAAGATATGTTGCTTCTCAGACTTTCACCGCATCTTTTGCCCCA
TTACAAGGTTTGGATAGATGGTTATCTTATTTAGTTTGGGTTACAGTTTTTGCTGCCAAGTACTCTGAATCGTACTTCTTCTTGATTTTGTCTCTAAGAGACCCTATCAGAATATTA
TCAACTACTACTATGAGATGTACTGGTGAGTATTGGTGGGGTTCAAAGTTATGTAGACATCAATCGAAGATTGTTTTAGGTTTCATGATTGCTACAGATTTCATTCTGTTCTTCCT
TGATACTTATTTGTGGTACATTGTTGTCAACACTGTCTTCTCTGTTGGTAAATCCTTCTATTTAGGTATTTCCATCTTAACTCCTTGGAGAAATATCTTTACAAGATTACCAAAGAG
AATTTACTCAAAGATTTTGGCCACAACTGATATGGAGATTAAA

>5

GTGGCCACTGTTTTATTCTTCTCGATTATGCCATTAGGTGGTCTTTTCACCTCATATATGCAAAAATCAAGTAGAAGATATGTTGCTTCTCAGACTTTCACCGCATCTTTTGCCCCA
TTACAAGGTTTGGATAGATGGTTATCTTATTTAGTTTGGGTTACTGTTTTTGCTGCCAAGTACTCTGAATCGTACTTCTTCTTGATTTTGTCTCTAAGAGACCCTATCAGAATTTTA
TCAACTACTACCATGAGATGTACTGGTGAGTATTGGTGGGGTTCAAAGTTATGTAGACATCAATCGAAGATTGTTTTAGGTTTCATGATTGCTACAGATTTCATTCTGTTCTTCCT
TGATACTTATTTGTGGTACATTGTTGTCAACACTGTCTTCTCTGTTGGTAAATCCTTCTATTTAGGTATTTCCATCTTAACTCCTTGGAGAAATATCTTTACAAGATTACCAAAGAG
AATTTACTCAAAGATTTTGGCCACAACTGATATGGAGATTAAA

>6

GTGGCCACTGTTTTATTCTTCTCGATTATGCCATTAGGTGGTCTTTTCACCTCATATATGCAAAAATCAAGTAGAAGATATGTTGCTTCTCAGACTTTCACCGCATCTTTTGCCCCA
TTACAAGGTTTGGATAGATGGTTATCTTATTTAGTTTGGGTTACTGTTTTTGCTGCCAAGTACTCTGAATCGTACTTCTTCTTGATTTTGTCTCTAAGAGACCCTATCAGAATTTTA
TCAACTACTACCATGAGATGTACTGGTGAGTATTGGTGGGGTTCAAAGTTATGTAGACATCAATCGAAGATTGTTTTAGGTTTCATGATTGCTACAGATTTCATTCTGTTCTTCCT

TGATACTTATTTGTGGTACATTGTTGTCAACACTGTCTTCTCTGTTGGTAAATCCTTCTATTTAGGTATTTCCATCTTAACTCCTTGGAGAAATATCTTTACAAGATTACCAAAGAG
AATTTACTCAAAGATTTTGGCCACAACTGATATGGAGATTAAA

>7

GTGGCCACTGTTTTATTCTTCTCGATTATGCCATTAGGTGGTCTTTTCACCTCATATATGCAAAAATCAAGTAGAAGATATGTTGCTTCTCAGACTTTCACCGCATCTTTTGCCCCA
TTACAAGGTTTGGATAGATGGTTATCTTATTTAGTTTGGGTTACTGTTTTTGCTGCCAAGTACTCTGAATCGTACTTCTTCTTGATTTTGTCTCTAAGAGACCCTATCAGAATTTTA
TCAACTACTACCATGAGATGTACTGGTGAGTATTGGTGGGGTTCAAAGTTATGTAGACATCAATCGAAGATTGTTTTAGGTTTCATGATTGCTACAGATTTCATTCTGTTCTTCCT
TGATACTTATTTGTGGTACATTGTTGTCAACACTGTCTTCTCTGTTGGTAAATCCTTCTATTTAGGTATTTCCATCTTAACTCCTTGGAGAAATATCTTTACAAGATTACCAAAGAG
AATTTACTCAAAGATTTTGGCCACAACTGATATGGAGATTAAA

>8

GTGGCCACTGTTTTATTCTTCTCGATTATGCCATTAGGTGGTCTTTTCACCTCATATATGCAAAAATCAAGTAGAAGATATGTTGCTTCTCAGACTTTCACCGCATCTTTTGCCCCA
TTACAAGGTTTGGATAGATGGTTATCTTATTTAGTTTGGGTTACTGTTTTTGCTGCCAAGTACTCTGAATCGTACTTCTTCTTGATTTTGTCTCTAAGAGACCCTATCAGAATTTTA
TCAACTACTACCATGAGATGTACTGGTGAGTATTGGTGGGGTTCAAAGTTATGTAGACATCAATCGAAGATTGTTTTAGGTTTCATGATTGCTACAGATTTCATTCTGTTCTTCCT
TGATACTTATTTGTGGTACATTGTTGTCAACACTGTCTTCTCTGTTGGTAAATCCTTCTATTTAGGTATTTCCATCTTAACTCCTTGGAGAAATATCTTTACAAGATTACCAAAGAG
AATTTACTCAAAGATTTTGGCCACAACTGATATGGAGATTAAA

>9

GTGGCCACTGTTTTATTCTTCTCGATTATGCCATTAGGTGGTCTTTTCACCTCATATATGCAAAAATCAAGTAGAAGATATGTTGCTTCTCAGACTTTCACCGCATCTTTTGCCCCA
TTACAAGGTTTGGATAGATGGTTATCTTATTTAGTTTGGGTTACTGTTTTTGCTGCCAAGTACTCTGAATCGTACTTCTTCTTGATTTTGTCTCTAAGAGACCCTATCAGAATTTTA
TCAACTACTACCATGAGATGTACTGGTGAGTATTGGTGGGGTTCAAAGTTATGTAGACATCAATCGAAGATTGTTTTAGGTTTCATGATTGCTACAGATTTCATTCTGTTCTTCCT
TGATACTTATTTGTGGTACATTGTTGTCAACACTGTCTTCTCTGTTGGTAAATCCTTCTATTTAGGTATTTCCATCTTAACTCCTTGGAGAAATATCTTTACAAGATTACCAAAGAG
AATTTACTCAAAGATTTTGGCCACAACTGATATGGAGATTAAA

>10

GTGGCCACTGTTTTATTCTTCTCGATTATGCCATTAGGTGGTCTTTTCACCTCATATATGCAAAAATCAAGTAGAAGATATGTTGCTTCTCAGACTTTCACCGCATCTTTTGCCCCA
TTACAAGGTTTGGATAGATGGTTATCTTATTTAGTTTGGGTTACAGTTTTTGCTGCCAAGTACTCTGAATCGTACTTCTTCTTGATTTTGTCTCTAAGAGACCCTATCAGAATATTA
TCAACTACTACTATGAGATGTACTGGTGAGTATTGGTGGGGTTCAAAGTTATGTAGACATCAATCGAAGATTGTTTTAGGTTTCATGATTGCTACAGATTTCATTCTGTTCTTCCT
TGATACTTATTTGTGGTACATTGTTGTCAACACTGTCTTCTCTGTTGGTAAATCCTTCTATTTAGGTATTTCCATCTTAACTCCTTGGAGAAATATCTTTACAAGATTACCAAAGAG
AATTTACTCAAAGATTTTGGCCACAACTGATATGGAGATTAAA

>11

GTGGCCACTGTTTTATTCTTCTCGATTATGCCATTAGGTGGTCTTTTCACCTCATATATGCAAAAATCAAGTAGAAGATATGTTGCTTCTCAGACTTTCACCGCATCTTTTGCCCCA
TTACAAGGTTTGGATAGATGGTTATCTTATTTAGTTTGGGTTACTGTTTTTGCTGCCAAGTACTCTGAATCGTACTTCTTCTTGATTTTGTCTCTAAGAGACCCTATCAGAATTTTA
TCAACTACTACCATGAGATGTACTGGTGAGTATTGGTGGGGTTCAAAGTTATGTAGACATCAATCGAAGATTGTTTTAGGTTTCATGATTGCTACAGATTTCATTCTGTTCTTCCT
TGATACTTATTTGTGGTACATTGTTGTCAACACTGTCTTCTCTGTTGGTAAATCCTTCTATTTAGGTATTTCCATCTTAACTCCTTGGAGAAATATCTTTACAAGATTACCAAAGAG
AATTTACTCAAAGATTTTGGCCACAACTGATATGGAGATTAAA

>12

GTGGCCACTGTTTTATTCTTCTCGATTATGCCATTAGGTGGTCTTTTCACCTCATATATGCAAAAATCAAGTAGAAGATATGTTGCTTCTCAGACTTTCACCGCATCTTTTGCCCCA
TTACAAGGTTTGGATAGATGGTTATCTTATTTAGTTTGGGTTACAGTTTTTGCTGCCAAGTACTCTGAATCGTACTTCTTCTTGATTTTGTCTCTAAGAGACCCTATCAGAATATTA
TCAACTACTACTATGAGATGTACTGGTGAGTATTGGTGGGGTTCAAAGTTATGTAGACATCAATCGAAGATTGTTTTAGGTTTCATGATTGCTACAGATTTCATTCTGTTCTTCCT
TGATACTTATTTGTGGTACATTGTTGTCAACACTGTCTTCTCTGTTGGTAAATCCTTCTATTTAGGTATTTCCATCTTAACTCCTTGGAGAAATATCTTTACAAGATTACCAAAGAG
AATTTACTCAAAGATTTTGGCCACAACTGATATGGAGATTAAA

>13

GTGGCCACTGTTTTATTCTTCTCGATTATGCCATTAGGTGGTCTTTTCACCTCATATATGCAAAAATCAAGTAGAAGATATGTTGCTTCTCAGACTTTCACCGCATCTTTTGCCCCA
TTACAAGGTTTGGATAGATGGCTATCTTATTTAGTTTGGGTTACAGTTTTTGCTGCCAAATACTCTGAATCGTACTTCTTCTTGATTTTGTCTCTAAGAGACCCTATCAGAATTTTA
TCAACTACTACCATGAGATGTACTGGTGAGTATTGGTGGGGTTCAAAGTTATGTAGACATCAATCGAAGATTGTTTTAGGTTTCATGATTGCTACAGATTTCATTTTGTTCTTCCT
TGATACTTATTTGTGGTACATTGTTGTCAACACTGTCTTCTCTGTTGGTAAATCCTTCTATCTAGGTATTTCCATCTTAACTCCTTGGAGAAATATCTTTACAAGATTACCAAAGAG
AATTTACTCAAAGATTTTGGCCACAACTGATATGGAGATTAAA

>14

GTGGCCACTGTTTTATTCTTCTCGATTATGCCATTAGGTGGTCTTTTCACCTCATATATGCAAAAATCAAGTAGAAGATATGTTGCTTCTCAGACTTTCACCGCATCTTTTGCCCCA
TTACAAGGTTTGGATAGATGGTTATCTTATTTAGTTTGGGTTACTGTTTTTGCTGCCAAGTACTCTGAATCGTACTTCTTCTTGATTTTGTCTCTAAGAGACCCTATCAGAATTTTA
TCAACTACTACCATGAGATGTACTGGTGAGTATTGGTGGGGTTCAAAGTTATGTAGACATCAATCGAAGATTGTTTTAGGTTTCATGATTGCTACAGATTTCATTCTGTTCTTCCT
TGATACTTATTTGTGGTACATTGTTGTCAACACTGTCTTCTCTGTTGGTAAATCCTTCTATTTAGGTATTTCCATCTTAACTCCTTGGAGAAATATCTTTACAAGATTACCAAAGAG
AATTTACTCAAAGATTTTGGCCACAACTGATATGGAGATTAAA

>15

GTGGCCACTGTTTTATTCTTCTCGATTATGCCATTAGGTGGTCTTTTCACCTCATATATGCAAAAATCAAGTAGAAGATATGTTGCTTCTCAGACTTTCACCGCATCTTTTGCCCCA
TTACAAGGTTTGGATAGATGGTTATCTTATTTAGTTTGGGTTACAGTTTTTGCTGCCAAGTACTCTGAATCGTACTTCTTCTTGATTTTGTCTCTAAGAGACCCTATCAGAATATTA
TCAACTACTACTATGAGATGTACTGGTGAGTATTGGTGGGGTTCAAAGTTATGTAGACATCAATCGAAGATTGTTTTAGGTTTCATGATTGCTACAGATTTCATTCTGTTCTTCCT
TGATACTTATTTGTGGTACATTGTTGTCAACACTGTCTTCTCTGTTGGTAAATCCTTCTATTTAGGTATTTCCATCTTAACTCCTTGGAGAAATATCTTTACAAGATTACCAAAGAG
AATTTACTCAAAGATTTTGGCCACAACTGATATGGAGATTAAA

>16

GTGGCCACTGTTTTATTCTTCTCGATTATGCCATTAGGTGGTCTTTTCACCTCATATATGCAAAAATCAAGTAGAAGATATGTTGCTTCTCAGACTTTCACCGCATCTTTTGCCCCA
TTACAAGGTTTGGATAGATGGTTATCTTATTTAGTTTGGGTTACTGTTTTTGCTGCCAAGTACTCTGAATCGTACTTCTTCTTGATTTTGTCTCTAAGAGACCCTATCAGAATTTTA
TCAACTACTACCATGAGATGTACTGGTGAGTATTGGTGGGGTTCAAAGTTATGTAGACATCAATCGAAGATTGTTTTAGGTTTCATGATTGCTACAGATTTCATTCTGTTCTTCCT
TGATACTTATTTGTGGTACATTGTTGTCAACACTGTCTTCTCTGTTGGTAAATCCTTCTATTTAGGTATTTCCATCTTAACTCCTTGGAGAAATATCTTTACAAGATTACCAAAGAG
AATTTACTCAAAGATTTTGGCCACAACTGATATGGAGATTAAA

>17

GTGGCCACTGTTTTATTCTTCTCGATTATGCCATTAGGTGGTCTTTTCACCTCATATATGCAAAAATCAAGTAGAAGATATGTTGCTTCTCAGACTTTCACCGCATCTTTTGCCCCA
TTACAAGGTTTGGATAGATGGTTATCTTATTTAGTTTGGGTTACAGTTTTTGCTGCCAAGTACTCTGAATCGTACTTCTTCTTGATTTTGTCTCTAAGAGACCCTATCAGAATATTA
TCAACTACTACTATGAGATGTACTGGTGAGTATTGGTGGGGTTCAAAGTTATGTAGACATCAATCGAAGATTGTTTTAGGTTTCATGATTGCTACAGATTTCATTCTGTTCTTCCT
TGATACTTATTTGTGGTACATTGTTGTCAACACTGTCTTCTCTGTTGGTAAATCCTTCTATTTAGGTATTTCCATCTTAACTCCTTGGAGAAATATCTTTACAAGATTACCAAAGAG
AATTTACTCAAAGATTTTGGCCACAACTGATATGGAGATTAAA

>18

GTGGCCACTGTTTTATTCTTCTCGATTATGCCATTAGGTGGTCTTTTCACCTCATATATGCAAAAATCAAGTAGAAGATATGTTGCTTCTCAGACTTTCACCGCATCTTTTGCCCCA
TTACAAGGTTTGGATAGATGGTTATCTTATTTAGTTTGGGTTACTGTTTTTGCTGCCAAGTACTCTGAATCGTACTTCTTCTTGATTTTGTCTCTAAGAGACCCTATCAGAATTTTA
TCAACTACTACCATGAGATGTACTGGTGAGTATTGGTGGGGTTCAAAGTTATGTAGACATCAATCGAAGATTGTTTTAGGTTTCATGATTGCTACAGATTTCATTCTGTTCTTCCT
TGATACTTATTTGTGGTACATTGTTGTCAACACTGTCTTCTCTGTTGGTAAATCCTTCTATTTAGGTATTTCCATCTTAACTCCTTGGAGAAATATCTTTACAAGATTACCAAAGAG
AATTTACTCAAAGATTTTGGCCACAACTGATATGGAGATTAAA

>19

GTGGCCACTGTTTTATTCTTCTCGATTATGCCATTAGGTGGTCTTTTCACCTCATATATGCAAAAATCAAGTAGAAGATATGTTGCTTCTCAGACTTTCACCGCATCTTTTGCCCCA
TTACAAGGTTTGGATAGATGGTTATCTTATTTAGTTTGGGTTACTGTTTTTGCTGCCAAGTACTCTGAATCGTACTTCTTCTTGATTTTGTCTCTAAGAGACCCTATCAGAATTTTA

TCAACTACTACCATGAGATGTACTGGTGAGTATTGGTGGGGTTCAAAGTTATGTAGACATCAATCGAAGATTGTTTTAGGTTTCATGATTGCTACAGATTTCATTCTGTTCTTCCT
TGATACTTATTTGTGGTACATTGTTGTCAACACTGTCTTCTCTGTTGGTAAATCCTTCTATTTAGGTATTTCCATCTTAACTCCTTGGAGAAATATCTTTACAAGATTACCAAAGAG
AATTTACTCAAAGATTTTGGCCACAACTGATATGGAGATTAAA

>20

GTGGCCACTGTTTTATTCTTCTCGATTATGCCATTAGGTGGTCTTTTCACCTCATATATGCAAAAATCAAGTAGAAGATATGTTGCTTCTCAGACTTTCACCGCATCTTTTGCCCCA
TTACAAGGTTTGGATAGATGGTTATCTTATTTAGTTTGGGTTACTGTTTTTGCTGCCAAGTACTCTGAATCGTACTTCTTCTTGATTTTGTCTCTAAGAGACCCTATCAGAATTTTA
TCAACTACTACCATGAGATGTACTGGTGAGTATTGGTGGGGTTCAAAGTTATGTAGACATCAATCGAAGATTGTTTTAGGTTTCATGATTGCTACAGATTTCATTCTGTTCTTCCT
TGATACTTATTTGTGGTACATTGTTGTCAACACTGTCTTCTCTGTTGGTAAATCCTTCTATTTAGGTATTTCCATCTTAACTCCTTGGAGAAATATCTTTACAAGATTACCAAAGAG
AATTTACTCAAAGATTTTGGCCACAACTGATATGGAGATTAAA

>21

GTGGCCACTGTTTTATTCTTCTCGATTATGCCATTAGGTGGTCTTTTCACCTCATATATGCAAAAATCAAGTAGAAGATATGTTGCTTCTCAGACTTTCACCGCATCTTTTGCCCCA
TTACAAGGTTTGGATAGATGGTTATCTTATTTAGTTTGGGTTACTGTTTTTGCTGCCAAGTACTCTGAATCGTACTTCTTCTTGATTTTGTCTCTAAGAGACCCTATCAGAATTTTA
TCAACTACTACCATGAGATGTACTGGTGAGTATTGGTGGGGTTCAAAGTTATGTAGACATCAATCGAAGATTGTTTTAGGTTTCATGATTGCTACAGATTTCATTCTGTTCTTCCT
TGATACTTATTTGTGGTACATTGTTGTCAACACTGTCTTCTCTGTTGGTAAATCCTTCTATTTAGGTATTTCCATCTTAACTCCTTGGAGAAATATCTTTACAAGATTACCAAAGAG
AATTTACTCAAAGATTTTGGCCACAACTGATATGGAGATTAAA

>22

GTGGCCACTGTTTTATTCTTCTCGATTATGCCATTAGGTGGTCTTTTCACCTCATATATGCAAAAATCAAGTAGAAGATATGTTGCTTCTCAGACTTTCACCGCATCTTTTGCCCCA
TTACAAGGTTTGGATAGATGGTTATCTTATTTAGTTTGGGTTACAGTTTTTGCTGCCAAGTACTCTGAATCGTACTTCTTCTTGATTTTGTCTCTAAGAGACCCTATCAGAATTTTA
TCAACTACTACCATGAGATGTACTGGTGAGTACTGGTGGGGTTCAAAGTTATGTAGACATCAATCGAAGATTGTTTTAGGTTTCATGATTGCTACAGATTTCATTTTGTTCTTCCT
TGATACTTATTTGTGGTACATTGTTGTCAACACTGTCTTCTCTGTTGGTAAATCCTTCTATCTAGGTATTTCCATCTTAACTCCTTGGAGAAATATCTTTACAAGATTACCAAAGAG
AATTTACTCAAAGATTTTGGCCACAACTGATATGGAGATTAAA

>23

GTGGCCACTGTTTTATTCTTCTCGATTATGCCATTAGGTGGTCTTTTCACCTCATATATGCAAAAATCAAGTAGAAGATATGTTGCTTCTCAGACTTTCACCGCATCTTTTGCCCCA
TTACAAGGTTTGGATAGATGGTTATCTTATTTAGTTTGGGTTACAGTTTTTGCTGCCAAGTACTCTGAATCGTACTTCTTCTTGATTTTGTCTCTAAGAGACCCTATCAGAATTTTA
TCAACTACTACCATGAGATGTACTGGTGAGTACTGGTGGGGTTCAAAGTTATGTAGACATCAATCGAAGATTGTTTTAGGTTTCATGATTGCTACAGATTTCATTTTGTTCTTCCT

TGATACTTATTTGTGGTACATTGTTGTCAACACTGTCTTCTCTGTTGGTAAATCCTTCTATCTAGGTATTTCCATCTTAACTCCTTGGAGAAATATCTTTACAAGATTACCAAAGAG
AATTTACTCAAAGATTTTGGCCACAACTGATATGGAGATTAAA

>24

GTGGCCACTGTTTTATTCTTCTCGATTATGCCATTAGGTGGTCTTTTCACCTCATATATGCAAAAATCAAGTAGAAGATATGTTGCTTCTCAGACTTTCACCGCATCTTTTGCCCCA
TTACAAGGTTTGGATAGATGGTTATCTTATTTAGTTTGGGTTACAGTTTTTGCTGCCAAGTACTCTGAATCGTACTTCTTCTTGATTTTGTCTCTAAGAGACCCTATCAGAATATTA
TCAACTACTACTATGAGATGTACTGGTGAGTATTGGTGGGGTTCAAAGTTATGTAGACATCAATCGAAGATTGTTTTAGGTTTCATGATTGCTACAGATTTCATTCTGTTCTTCCT
TGATACTTATTTGTGGTACATTGTTGTCAACACTGTCTTCTCTGTTGGTAAATCCTTCTATTTAGGTATTTCCATCTTAACTCCTTGGAGAAATATCTTTACAAGATTACCAAAGAG
AATTTACTCAAAGATTTTGGCCACAACTGATATGGAGATTAAA

>25

GTGGCCACTGTTTTATTCTTCTCGATTATGCCATTAGGTGGTCTTTTCACCTCATATATGCAAAAATCAAGTAGAAGATATGTTGCTTCTCAGACTTTCACCGCATCTTTTGCCCCA
TTACAAGGTTTGGATAGATGGTTATCTTATTTAGTTTGGGTTACAGTTTTTGCTGCCAAGTACTCTGAATCGTACTTCTTCTTGATTTTGTCTCTAAGAGACCCTATCAGAATATTA
TCAACTACTACTATGAGATGTACTGGTGAGTATTGGTGGGGTTCAAAGTTATGTAGACATCAATCGAAGATTGTTTTAGGTTTCATGATTGCTACAGATTTCATTCTGTTCTTCCT
TGATACTTATTTGTGGTACATTGTTGTCAACACTGTCTTCTCTGTTGGTAAATCCTTCTATTTAGGTATTTCCATCTTAACTCCTTGGAGAAATATCTTTACAAGATTACCAAAGAG
AATTTACTCAAAGATTTTGGCCACAACTGATATGGAGATTAAA

>26

GTGGCCACTGTTTTATTCTTCTCGATTATGCCATTAGGTGGTCTTTTCACCTCATATATGCAAAAATCAAGTAGAAGATATGTTGCTTCTCAGACTTTCACCGCATCTTTTGCCCCA
TTACAAGGTTTGGATAGATGGTTATCTTATTTAGTTTGGGTTACAGTTTTTGCTGCCAAGTATTCTGAATCGTACTTCTTCTTGATTTTGTCTCTAAGAGACCCTATCAGAATTTTA
TCAACTACTACCATGAGATGTACTGGTGAGTACTGGTGGGGTTCAAAGTTATGTAGACATCAATCGAAGATTGTTTTAGGTTTCATGATTGCTACAGATTTCATTCTGTTCTTCCT
TGATACTTATTTGTGGTACATTGTTGTCAACACTGTCTTCTCTGTTGGTAAATCCTTCTATTTAGGTATTTCCATCTTAACTCCTTGGAGAAATATCTTTACAAGATTACCAAAGAG
AATTTACTCAAAGATTTTGGCCACAACTGATATGGAGATTAAA

>27

GTGGCCACTGTTTTATTCTTCTCGATTATGCCATTAGGTGGTCTTTTCACCTCATATATGCAAAAATCAAGTAGAAGATATGTTGCTTCTCAGACTTTCACCGCATCTTTTGCCCCA
TTACAAGGTTTGGATAGATGGCTATCTTATTTAGTTTGGGTTACAGTTTTTGCTGCCAAATACTCTGAATCGTACTTCTTCTTGATTTTGTCTCTAAGAGACCCTATCAGAATTTTA
TCAACTACTACCATGAGATGTACTGGTGAGTATTGGTGGGGTTCAAAGTTATGTAGACATCAATCGAAGATTGTTTTAGGTTTCATGATTGCTACAGATTTCATTTTGTTCTTCCT
TGATACTTATTTGTGGTACATTGTTGTCAACACTGTCTTCTCTGTTGGTAAATCCTTCTATCTAGGTATTTCCATCTTAACTCCTTGGAGAAATATCTTTACAAGATTACCAAAGAG
AATTTACTCAAAGATTTTGGCCACAACTGATATGGAGATTAAA

>28

GTGGCCACTGTTTTATTCTTCTCGATTATGCCATTAGGTGGTCTTTTCACCTCATATATGCAAAAATCAAGTAGAAGATATGTTGCTTCTCAGACTTTCACCGCATCTTTTGCCCCA
TTACAAGGTTTGGATAGATGGTTATCTTATTTAGTTTGGGTTACTGTTTTTGCTGCCAAGTACTCTGAATCGTACTTCTTCTTGATTTTGTCTCTAAGAGACCCTATCAGAATTTTA
TCAACTACTACCATGAGATGTACTGGTGAGTATTGGTGGGGTTCAAAGTTATGTAGACATCAATCGAAGATTGTTTTAGGTTTCATGATTGCTACAGATTTCATTCTGTTCTTCCT
TGATACTTATTTGTGGTACATTGTTGTCAACACTGTCTTCTCTGTTGGTAAATCCTTCTATTTAGGTATTTCCATCTTAACTCCTTGGAGAAATATCTTTACAAGATTACCAAAGAG
AATTTACTCAAAGATTTTGGCCACAACTGATATGGAGATTAAA

>29

GTGGCCACTGTTTTATTCTTCTCGATTATGCCATTAGGTGGTCTTTTCACCTCATATATGCAAAAATCAAGTAGAAGATATGTTGCTTCTCAGACTTTCACCGCATCTTTTGCCCCA
TTACAAGGTTTGGATAGATGGTTATCTTATTTAGTTTGGGTTACAGTTTTTGCTGCCAAGTACTCTGAATCGTACTTCTTCTTGATTTTGTCTCTAAGAGACCCTATCAGAATATTA
TCAACTACTACTATGAGATGTACTGGTGAGTATTGGTGGGGTTCAAAGTTATGTAGACATCAATCGAAGATTGTTTTAGGTTTCATGATTGCTACAGATTTCATTCTGTTCTTCCT
TGATACTTATTTGTGGTACATTGTTGTCAACACTGTCTTCTCTGTTGGTAAATCCTTCTATTTAGGTATTTCCATCTTAACTCCTTGGAGAAATATCTTTACAAGATTACCAAAGAG
AATTTACTCAAAGATTTTGGCCACAACTGATATGGAGATTAAA

>30

GTGGCCACTGTTTTATTCTTCTCGATTATGCCATTAGGTGGTCTTTTCACCTCATATATGCAAAAATCAAGTAGAAGATATGTTGCTTCTCAGACTTTCACCGCATCTTTTGCCCCA
TTACAAGGTTTGGATAGATGGTTATCTTATTTAGTTTGGGTTACAGTTTTTGCTGCCAAGTATTCTGAATCGTACTTCTTCTTGATTTTGTCTCTAAGAGACCCTATCAGAATTTTA
TCAACTACTACCATGAGATGTACTGGTGAGTACTGGTGGGGTTCAAAGTTATGTAGACATCAATCGAAGATTGTTTTAGGTTTCATGATTGCTACAGATTTCATTCTGTTCTTCCT
TGATACTTATTTGTGGTACATTGTTGTCAACACTGTCTTCTCTGTTGGTAAATCCTTCTATTTAGGTATTTCCATCTTAACTCCTTGGAGAAATATCTTTACAAGATTACCAAAGAG
AATTTACTCAAAGATTTTGGCCACAACTGATATGGAGATTAAA

>31

GTGGCCACTGTTTTATTCTTCTCGATTATGCCATTAGGTGGTCTTTTCACCTCATATATGCAAAAATCAAGTAGAAGATATGTTGCTTCTCAGACTTTCACCGCATCTTTTGCCCCA
TTACAAGGTTTGGATAGATGGTTATCTTATTTAGTTTGGGTTACTGTTTTTGCTGCCAAGTACTCTGAATCGTACTTCTTCTTGATTTTGTCTCTAAGAGACCCTATCAGAATTTTA
TCAACTACTACCATGAGATGTACTGGTGAGTATTGGTGGGGTTCAAAGTTATGTAGACATCAATCGAAGATTGTTTTAGGTTTCATGATTGCTACAGATTTCATTCTGTTCTTCCT
TGATACTTATTTGTGGTACATTGTTGTCAACACTGTCTTCTCTGTTGGTAAATCCTTCTATTTAGGTATTTCCATCTTAACTCCTTGGAGAAATATCTTTACAAGATTACCAAAGAG
AATTTACTCAAAGATTTTGGCCACAACTGATATGGAGATTAAA

>32

GTGGCCACTGTTTTATTCTTCTCGATTATGCCATTAGGTGGTCTTTTCACCTCATATATGCAAAAATCAAGTAGAAGATATGTTGCTTCTCAGACTTTCACCGCATCTTTTGCCCCA
TTACAAGGTTTGGATAGATGGTTATCTTATTTAGTTTGGGTTACTGTTTTTGCTGCCAAGTACTCTGAATCGTACTTCTTCTTGATTTTGTCTCTAAGAGACCCTATCAGAATTTTA
TCAACTACTACCATGAGATGTACTGGTGAGTATTGGTGGGGTTCAAAGTTATGTAGACATCAATCGAAGATTGTTTTAGGTTTCATGATTGCTACAGATTTCATTCTGTTCTTCCT
TGATACTTATTTGTGGTACATTGTTGTCAACACTGTCTTCTCTGTTGGTAAATCCTTCTATTTAGGTATTTCCATCTTAACTCCTTGGAGAAATATCTTTACAAGATTACCAAAGAG
AATTTACTCAAAGATTTTGGCCACAACTGATATGGAGATTAAA

>33

GTGGCCACTGTTTTATTCTTCTCGATTATGCCATTAGGTGGTCTTTTCACCTCATATATGCAAAAATCAAGTAGAAGATATGTTGCTTCTCAGACTTTCACCGCATCTTTTGCCCCA
TTACAAGGTTTGGATAGATGGTTATCTTATTTAGTTTGGGTTACTGTTTTTGCTGCCAAGTACTCTGAATCGTACTTCTTCTTGATTTTGTCTCTAAGAGACCCTATCAGAATTTTA
TCAACTACTACCATGAGATGTACTGGTGAGTATTGGTGGGGTTCAAAGTTATGTAGACATCAATCGAAGATTGTTTTAGGTTTCATGATTGCTACAGATTTCATTCTGTTCTTCCT
TGATACTTATTTGTGGTACATTGTTGTCAACACTGTCTTCTCTGTTGGTAAATCCTTCTATTTAGGTATTTCCATCTTAACTCCTTGGAGAAATATCTTTACAAGATTACCAAAGAG
AATTTACTCAAAGATTTTGGCCACAACTGATATGGAGATTAAA

>34

GTGGCCACTGTTTTATTCTTCTCGATTATGCCATTAGGTGGTCTTTTCACCTCATATATGCAAAAATCAAGTAGAAGATATGTTGCTTCTCAGACTTTCACCGCATCTTTTGCCCCA
TTACAAGGTTTGGATAGATGGTTATCTTATTTAGTTTGGGTTACAGTTTTTGCTGCCAAGTACTCTGAATCGTACTTCTTCTTGATTTTGTCTCTAAGAGACCCTATCAGAATATTA
TCAACTACTACTATGAGATGTACTGGTGAGTATTGGTGGGGTTCAAAGTTATGTAGACATCAATCGAAGATTGTTTTAGGTTTCATGATTGCTACAGATTTCATTCTGTTCTTCCT
TGATACTTATTTGTGGTACATTGTTGTCAACACTGTCTTCTCTGTTGGTAAATCCTTCTATTTAGGTATTTCCATCTTAACTCCTTGGAGAAATATCTTTACAAGATTACCAAAGAG
AATTTACTCAAAGATTTTGGCCACAACTGATATGGAGATTAAA

>35

GTGGCCACTGTTTTATTCTTCTCGATTATGCCATTAGGTGGTCTTTTCACCTCATATATGCAAAAATCAAGTAGAAGATATGTTGCTTCTCAGACTTTCACCGCATCTTTTGCCCCA
TTACAAGGTTTGGATAGATGGTTATCTTATTTAGTTTGGGTTACAGTTTTTGCTGCCAAGTACTCTGAATCGTACTTCTTCTTGATTTTGTCTCTAAGAGACCCTATCAGAATATTA
TCAACTACTACTATGAGATGTACTGGTGAGTATTGGTGGGGTTCAAAGTTATGTAGACATCAATCGAAGATTGTTTTAGGTTTCATGATTGCTACAGATTTCATTCTGTTCTTCCT
TGATACTTATTTGTGGTACATTGTTGTCAACACTGTCTTCTCTGTTGGTAAATCCTTCTATTTAGGTATTTCCATCTTAACTCCTTGGAGAAATATCTTTACAAGATTACCAAAGAG
AATTTACTCAAAGATTTTGGCCACAACTGATATGGAGATTAAA

>36

GTGGCCACTGTTTTATTCTTCTCGATTATGCCATTAGGTGGTCTTTTCACCTCATATATGCAAAAATCAAGTAGAAGATATGTTGCTTCTCAGACTTTCACCGCATCTTTTGCCCCA
TTACAAGGTTTGGATAGATGGCTATCTTATTTAGTTTGGGTTACAGTTTTTGCTGCCAAATACTCTGAATCGTACTTCTTCTTGATTTTGTCTCTAAGAGACCCTATCAGAATTTTA

TCAACTACTACCATGAGATGTACTGGTGAGTATTGGTGGGGTTCAAAGTTATGTAGACATCAATCGAAGATTGTTTTAGGTTTCATGATTGCTACAGATTTCATTTTGTTCTTCCT
TGATACTTATTTGTGGTACATTGTTGTCAACACTGTCTTCTCTGTTGGTAAATCCTTCTATCTAGGTATTTCCATCTTAACTCCTTGGAGAAATATCTTTACAAGATTACCAAAGAG
AATTTACTCAAAGATTTTGGCCACAACTGATATGGAGATTAAA

>37

GTGGCCACTGTTTTATTCTTCTCGATTATGCCATTAGGTGGTCTTTTCACCTCATATATGCAAAAATCAAGTAGAAGATATGTTGCTTCTCAGACTTTCACCGCATCTTTTGCCCCA
TTACAAGGTTTGGATAGATGGCTATCTTATTTAGTTTGGGTTACAGTTTTTGCTGCCAAATACTCTGAATCGTACTTCTTCTTGATTTTGTCTCTAAGAGACCCTATCAGAATTTTA
TCAACTACTACCATGAGATGTACTGGTGAGTATTGGTGGGGTTCAAAGTTATGTAGACATCAATCGAAGATTGTTTTAGGTTTCATGATTGCTACAGATTTCATTTTGTTCTTCCT
TGATACTTATTTGTGGTACATTGTTGTCAACACTGTCTTCTCTGTTGGTAAATCCTTCTATCTAGGTATTTCCATCTTAACTCCTTGGAGAAATATCTTTACAAGATTACCAAAGAG
AATTTACTCAAAGATTTTGGCCACAACTGATATGGAGATTAAA

>38

GTGGCCACTGTTTTATTCTTCTCGATTATGCCATTAGGTGGTCTTTTCACCTCATATATGCAAAAATCAAGTAGAAGATATGTTGCTTCTCAGACTTTCACCGCATCTTTTGCCCCA
TTACAAGGTTTGGATAGATGGTTATCTTATTTAGTTTGGGTTACAGTTTTTGCTGCCAAGTATTCTGAATCGTACTTCTTCTTGATTTTGTCTCTAAGAGACCCTATCAGAATTTTA
TCAACTACTACCATGAGATGTACTGGTGAGTACTGGTGGGGTTCAAAGTTATGTAGACATCAATCGAAGATTGTTTTAGGTTTCATGATTGCTACAGATTTCATTCTGTTCTTCCT
TGATACTTATTTGTGGTACATTGTTGTCAACACTGTCTTCTCTGTTGGTAAATCCTTCTATTTAGGTATTTCCATCTTAACTCCTTGGAGAAATATCTTTACAAGATTACCAAAGAG
AATTTACTCAAAGATTTTGGCCACAACTGATATGGAGATTAAA

>39

GTGGCCACTGTTTTATTCTTCTCGATTATGCCATTAGGTGGTCTTTTCACCTCATATATGCAAAAATCAAGTAGAAGATATGTTGCTTCTCAGACTTTCACCGCATCTTTTGCCCCA
TTACAAGGTTTGGATAGATGGTTATCTTATTTAGTTTGGGTTACTGTTTTTGCTGCCAAGTACTCTGAATCGTACTTCTTCTTGATTTTGTCTCTAAGAGACCCTATCAGAATTTTA
TCAACTACTACCATGAGATGTACTGGTGAGTATTGGTGGGGTTCAAAGTTATGTAGACATCAATCGAAGATTGTTTTAGGTTTCATGATTGCTACAGATTTCATTCTGTTCTTCCT
TGATACTTATTTGTGGTACATTGTTGTCAACACTGTCTTCTCTGTTGGTAAATCCTTCTATTTAGGTATTTCCATCTTAACTCCTTGGAGAAATATCTTTACAAGATTACCAAAGAG
AATTTACTCAAAGATTTTGGCCACAACTGATATGGAGATTAAA

>40

GTGGCCACTGTTTTATTCTTCTCGATTATGCCATTAGGTGGTCTTTTCACCTCATATATGCAAAAATCAAGTAGAAGATATGTTGCTTCTCAGACTTTCACCGCATCTTTTGCCCCA
TTACAAGGTTTGGATAGATGGTTATCTTATTTAGTTTGGGTTACTGTTTTTGCTGCCAAGTACTCTGAATCGTACTTCTTCTTGATTTTGTCTCTAAGAGACCCTATCAGAATTTTA
TCAACTACTACCATGAGATGTACTGGTGAGTATTGGTGGGGTTCAAAGTTATGTAGACATCAATCGAAGATTGTTTTAGGTTTCATGATTGCTACAGATTTCATTCTGTTCTTCCT

TGATACTTATTTGTGGTACATTGTTGTCAACACTGTCTTCTCTGTTGGTAAATCCTTCTATTTAGGTATTTCCATCTTAACTCCTTGGAGAAATATCTTTACAAGATTACCAAAGAG
AATTTACTCAAAGATTTTGGCCACAACTGATATGGAGATTAAA

>41

GTGGCCACTGTTTTATTCTTCTCGATTATGCCATTAGGTGGTCTTTTCACCTCATATATGCAAAAATCAAGTAGAAGATATGTTGCTTCTCAGACTTTCACCGCATCTTTTGCCCCA
TTACAAGGTTTGGATAGATGGTTATCTTATTTAGTTTGGGTTACAGTTTTTGCTGCCAAGTACTCTGAATCGTACTTCTTCTTGATTTTGTCTCTAAGAGACCCTATCAGAATTTTA
TCAACTACTACCATGAGATGTACTGGTGAGTACTGGTGGGGTTCAAAGTTATGTAGACATCAATCGAAGATTGTTTTAGGTTTCATGATTGCTACAGATTTCATTTTGTTCTTCCT
TGATACTTATTTGTGGTACATTGTTGTCAACACTGTCTTCTCTGTTGGTAAATCCTTCTATCTAGGTATTTCCATCTTAACTCCTTGGAGAAATATCTTTACAAGATTACCAAAGAG
AATTTACTCAAAGATTTTGGCCACAACTGATATGGAGATTAAA

>42

GTGGCCACTGTTTTATTCTTCTCGATTATGCCATTAGGTGGTCTTTTCACCTCATATATGCAAAAATCAAGTAGAAGATATGTTGCTTCTCAGACTTTCACCGCATCTTTTGCCCCA
TTACAAGGTTTGGATAGATGGTTATCTTATTTAGTTTGGGTTACAGTTTTTGCTGCCAAGTACTCTGAATCGTACTTCTTCTTGATTTTGTCTCTAAGAGACCCTATCAGAATTTTA
TCAACTACTACCATGAGATGTACTGGTGAGTACTGGTGGGGTTCAAAGTTATGTAGACATCAATCGAAGATTGTTTTAGGTTTCATGATTGCTACAGATTTCATTTTGTTCTTCCT
TGATACTTATTTGTGGTACATTGTTGTCAACACTGTCTTCTCTGTTGGTAAATCCTTCTATCTAGGTATTTCCATCTTAACTCCTTGGAGAAATATCTTTACAAGATTACCAAAGAG
AATTTACTCAAAGATTTTGGCCACAACTGATATGGAGATTAAA

>43

GTGGCCACTGTTTTATTCTTCTCGATTATGCCATTAGGTGGTCTTTTCACCTCATATATGCAAAAATCAAGTAGAAGATATGTTGCTTCTCAGACTTTCACCGCATCTTTTGCCCCA
TTACAAGGTTTGGATAGATGGTTATCTTATTTAGTTTGGGTTACAGTTTTTGCTGCCAAGTACTCTGAATCGTACTTCTTCTTGATTTTGTCTCTAAGAGACCCTATCAGAATATTA
TCAACTACTACTATGAGATGTACTGGTGAGTATTGGTGGGGTTCAAAGTTATGTAGACATCAATCGAAGATTGTTTTAGGTTTCATGATTGCTACAGATTTCATTCTGTTCTTCCT
TGATACTTATTTGTGGTACATTGTTGTCAACACTGTCTTCTCTGTTGGTAAATCCTTCTATTTAGGTATTTCCATCTTAACTCCTTGGAGAAATATCTTTACAAGATTACCAAAGAG
AATTTACTCAAAGATTTTGGCCACAACTGATATGGAGATTAAA

>44

GTGGCCACTGTTTTATTCTTCTCGATTATGCCATTAGGTGGTCTTTTCACCTCATATATGCAAAAATCAAGTAGAAGATATGTTGCTTCTCAGACTTTCACCGCATCTTTTGCCCCA
TTACAAGGTTTGGATAGATGGTTATCTTATTTAGTTTGGGTTACTGTTTTTGCTGCCAAGTACTCTGAATCGTACTTCTTCTTGATTTTGTCTCTAAGAGACCCTATCAGAATTTTA
TCAACTACTACCATGAGATGTACTGGTGAGTATTGGTGGGGTTCAAAGTTATGTAGACATCAATCGAAGATTGTTTTAGGTTTCATGATTGCTACAGATTTCATTCTGTTCTTCCT
TGATACTTATTTGTGGTACATTGTTGTCAACACTGTCTTCTCTGTTGGTAAATCCTTCTATTTAGGTATTTCCATCTTAACTCCTTGGAGAAATATCTTTACAAGATTACCAAAGAG
AATTTACTCAAAGATTTTGGCCACAACTGATATGGAGATTAAA

>45

GTGGCCACTGTTTTATTCTTCTCGATTATGCCATTAGGTGGTCTTTTCACCTCATATATGCAAAAATCAAGTAGAAGATATGTTGCTTCTCAGACTTTCACCGCATCTTTTGCCCCA
TTACAAGGTTTGGATAGATGGTTATCTTATTTAGTTTGGGTTACAGTTTTTGCTGCCAAGTATTCTGAATCGTACTTCTTCTTGATTTTGTCTCTAAGAGACCCTATCAGAATTTTA
TCAACTACTACCATGAGATGTACTGGTGAGTACTGGTGGGGTTCAAAGTTATGTAGACATCAATCGAAGATTGTTTTAGGTTTCATGATTGCTACAGATTTCATTCTGTTCTTCCT
TGATACTTATTTGTGGTACATTGTTGTCAACACTGTCTTCTCTGTTGGTAAATCCTTCTATTTAGGTATTTCCATCTTAACTCCTTGGAGAAATATCTTTACAAGATTACCAAAGAG
AATTTACTCAAAGATTTTGGCCACAACTGATATGGAGATTAAA

>46

GTGGCCACTGTTTTATTCTTCTCGATTATGCCATTAGGTGGTCTTTTCACCTCATATATGCAAAAATCAAGTAGAAGATATGTTGCTTCTCAGACTTTCACCGCATCTTTTGCCCCA
TTACAAGGTTTGGATAGATGGTTATCTTATTTAGTTTGGGTTACAGTTTTTGCTGCCAAGTATTCTGAATCGTACTTCTTCTTGATTTTGTCTCTAAGAGACCCTATCAGAATTTTA
TCAACTACTACCATGAGATGTACTGGTGAGTACTGGTGGGGTTCAAAGTTATGTAGACATCAATCGAAGATTGTTTTAGGTTTCATGATTGCTACAGATTTCATTCTGTTCTTCCT
TGATACTTATTTGTGGTACATTGTTGTCAACACTGTCTTCTCTGTTGGTAAATCCTTCTATTTAGGTATTTCCATCTTAACTCCTTGGAGAAATATCTTTACAAGATTACCAAAGAG
AATTTACTCAAAGATTTTGGCCACAACTGATATGGAGATTAAA

>47

GTGGCCACTGTTTTATTCTTCTCGATTATGCCATTAGGTGGTCTTTTCACCTCATATATGCAAAAATCAAGTAGAAGATATGTTGCTTCTCAGACTTTCACCGCATCTTTTGCCCCA
TTACAAGGTTTGGATAGATGGTTATCTTATTTAGTTTGGGTTACAGTTTTTGCTGCCAAGTACTCTGAATCGTACTTCTTCTTGATTTTGTCTCTAAGAGACCCTATCAGAATATTA
TCAACTACTACTATGAGATGTACTGGTGAGTATTGGTGGGGTTCAAAGTTATGTAGACATCAATCGAAGATTGTTTTAGGTTTCATGATTGCTACAGATTTCATTCTGTTCTTCCT
TGATACTTATTTGTGGTACATTGTTGTCAACACTGTCTTCTCTGTTGGTAAATCCTTCTATTTAGGTATTTCCATCTTAACTCCTTGGAGAAATATCTTTACAAGATTACCAAAGAG
AATTTACTCAAAGATTTTGGCCACAACTGATATGGAGATTAAA

>48

GTGGCCACTGTTTTATTCTTCTCGATTATGCCATTAGGTGGTCTTTTCACCTCATATATGCAAAAATCAAGTAGAAGATATGTTGCTTCTCAGACTTTCACCGCATCTTTTGCCCCA
TTACAAGGTTTGGATAGATGGTTATCTTATTTAGTTTGGGTTACAGTTTTTGCTGCCAAGTACTCTGAATCGTACTTCTTCTTGATTTTGTCTCTAAGAGACCCTATCAGAATATTA
TCAACTACTACTATGAGATGTACTGGTGAGTATTGGTGGGGTTCAAAGTTATGTAGACATCAATCGAAGATTGTTTTAGGTTTCATGATTGCTACAGATTTCATTCTGTTCTTCCT
TGATACTTATTTGTGGTACATTGTTGTCAACACTGTCTTCTCTGTTGGTAAATCCTTCTATTTAGGTATTTCCATCTTAACTCCTTGGAGAAATATCTTTACAAGATTACCAAAGAG
AATTTACTCAAAGATTTTGGCCACAACTGATATGGAGATTAAA

>49

GTGGCCACTGTTTTATTCTTCTCGATTATGCCATTAGGTGGTCTTTTCACCTCATATATGCAAAAATCAAGTAGAAGATATGTTGCTTCTCAGACTTTCACCGCATCTTTTGCCCCA
TTACAAGGTTTGGATAGATGGTTATCTTATTTAGTTTGGGTTACAGTTTTTGCTGCCAAGTATTCTGAATCGTACTTCTTCTTGATTTTGTCTCTAAGAGACCCTATCAGAATTTTA
TCAACTACTACCATGAGATGTACTGGTGAGTACTGGTGGGGTTCAAAGTTATGTAGACATCAATCGAAGATTGTTTTAGGTTTCATGATTGCTACAGATTTCATTCTGTTCTTCCT
TGATACTTATTTGTGGTACATTGTTGTCAACACTGTCTTCTCTGTTGGTAAATCCTTCTATTTAGGTATTTCCATCTTAACTCCTTGGAGAAATATCTTTACAAGATTACCAAAGAG
AATTTACTCAAAGATTTTGGCCACAACTGATATGGAGATTAAA

>50

GTGGCCACTGTTTTATTCTTCTCGATTATGCCATTAGGTGGTCTTTTCACCTCATATATGCAAAAATCAAGTAGAAGATATGTTGCTTCTCAGACTTTCACCGCATCTTTTGCCCCA
TTACAAGGTTTGGATAGATGGTTATCTTATTTAGTTTGGGTTACAGTTTTTGCTGCCAAGTATTCTGAATCGTACTTCTTCTTGATTTTGTCTCTAAGAGACCCTATCAGAATTTTA
TCAACTACTACCATGAGATGTACTGGTGAGTACTGGTGGGGTTCAAAGTTATGTAGACATCAATCGAAGATTGTTTTAGGTTTCATGATTGCTACAGATTTCATTCTGTTCTTCCT
TGATACTTATTTGTGGTACATTGTTGTCAACACTGTCTTCTCTGTTGGTAAATCCTTCTATTTAGGTATTTCCATCTTAACTCCTTGGAGAAATATCTTTACAAGATTACCAAAGAG
AATTTACTCAAAGATTTTGGCCACAACTGATATGGAGATTAAA

>51

GTGGCCACTGTTTTATTCTTCTCGATTATGCCATTAGGTGGTCTTTTCACCTCATATATGCAAAAATCAAGTAGAAGATATGTTGCTTCTCAGACTTTCACCGCATCTTTTGCCCCA
TTACAAGGTTTGGATAGATGGTTATCTTATTTAGTTTGGGTTACAGTTTTTGCTGCCAAGTACTCTGAATCGTACTTCTTCTTGATTTTGTCTCTAAGAGACCCTATCAGAATTTTA
TCAACTACTACCATGAGATGTACTGGTGAGTACTGGTGGGGTTCAAAGTTATGTAGACATCAATCGAAGATTGTTTTAGGTTTCATGATTGCTACAGATTTCATTTTGTTCTTCCT
TGATACTTATTTGTGGTACATTGTTGTCAACACTGTCTTCTCTGTTGGTAAATCCTTCTATCTAGGTATTTCCATCTTAACTCCTTGGAGAAATATCTTTACAAGATTACCAAAGAG
AATTTACTCAAAGATTTTGGCCACAACTGATATGGAGATTAAA

>52

GTGGCCACTGTTTTATTCTTCTCGATTATGCCATTAGGTGGTCTTTTCACCTCATATATGCAAAAATCAAGTAGAAGATATGTTGCTTCTCAGACTTTCACCGCATCTTTTGCCCCA
TTACAAGGTTTGGATAGATGGTTATCTTATTTAGTTTGGGTTACAGTTTTTGCTGCCAAGTACTCTGAATCGTACTTCTTCTTGATTTTGTCTCTAAGAGACCCTATCAGAATTTTA
TCAACTACTACCATGAGATGTACTGGTGAGTACTGGTGGGGTTCAAAGTTATGTAGACATCAATCGAAGATTGTTTTAGGTTTCATGATTGCTACAGATTTCATTTTGTTCTTCCT
TGATACTTATTTGTGGTACATTGTTGTCAACACTGTCTTCTCTGTTGGTAAATCCTTCTATCTAGGTATTTCCATCTTAACTCCTTGGAGAAATATCTTTACAAGATTACCAAAGAG
AATTTACTCAAAGATTTTGGCCACAACTGATATGGAGATTAAA

>53

GTGGCCACTGTTTTATTCTTCTCGATTATGCCATTAGGTGGTCTTTTCACCTCATATATGCAAAAATCAAGTAGAAGATATGTTGCTTCTCAGACTTTCACCGCATCTTTTGCCCCA
TTACAAGGTTTGGATAGATGGTTATCTTATTTAGTTTGGGTTACTGTTTTTGCTGCCAAGTACTCTGAATCGTACTTCTTCTTGATTTTGTCTCTAAGAGACCCTATCAGAATTTTA

TCAACTACTACCATGAGATGTACTGGTGAGTATTGGTGGGGTTCAAAGTTATGTAGACATCAATCGAAGATTGTTTTAGGTTTCATGATTGCTACAGATTTCATTCTGTTCTTCCT
TGATACTTATTTGTGGTACATTGTTGTCAACACTGTCTTCTCTGTTGGTAAATCCTTCTATTTAGGTATTTCCATCTTAACTCCTTGGAGAAATATCTTTACAAGATTACCAAAGAG
AATTTACTCAAAGATTTTGGCCACAACTGATATGGAGATTAAA

>54

GTGGCCACTGTTTTATTCTTCTCGATTATGCCATTAGGTGGTCTTTTCACCTCATATATGCAAAAATCAAGTAGAAGATATGTTGCTTCTCAGACTTTCACCGCATCTTTTGCCCCA
TTACAAGGTTTGGATAGATGGTTATCTTATTTAGTTTGGGTTACAGTTTTTGCTGCCAAGTATTCTGAATCGTACTTCTTCTTGATTTTGTCTCTAAGAGACCCTATCAGAATTTTA
TCAACTACTACCATGAGATGTACTGGTGAGTACTGGTGGGGTTCAAAGTTATGTAGACATCAATCGAAGATTGTTTTAGGTTTCATGATTGCTACAGATTTCATTCTGTTCTTCCT
TGATACTTATTTGTGGTACATTGTTGTCAACACTGTCTTCTCTGTTGGTAAATCCTTCTATTTAGGTATTTCCATCTTAACTCCTTGGAGAAATATCTTTACAAGATTACCAAAGAG
AATTTACTCAAAGATTTTGGCCACAACTGATATGGAGATTAAA

>55

GTGGCCACTGTTTTATTCTTCTCGATTATGCCATTAGGTGGTCTTTTCACCTCATATATGCAAAAATCAAGTAGAAGATATGTTGCTTCTCAGACTTTCACCGCATCTTTTGCCCCA
TTACAAGGTTTGGATAGATGGTTATCTTATTTAGTTTGGGTTACAGTTTTTGCTGCCAAGTACTCTGAATCGTACTTCTTCTTGATTTTGTCTCTAAGAGACCCTATCAGAATTTTA
TCAACTACTACCATGAGATGTACTGGTGAGTACTGGTGGGGTTCAAAGTTATGTAGACATCAATCGAAGATTGTTTTAGGTTTCATGATTGCTACAGATTTCATTTTGTTCTTCCT
TGATACTTATTTGTGGTACATTGTTGTCAACACTGTCTTCTCTGTTGGTAAATCCTTCTATCTAGGTATTTCCATCTTAACTCCTTGGAGAAATATCTTTACAAGATTACCAAAGAG
AATTTACTCAAAGATTTTGGCCACAACTGATATGGAGATTAAA

>56

GTGGCCACTGTTTTATTCTTCTCGATTATGCCATTAGGTGGTCTTTTCACCTCATATATGCAAAAATCAAGTAGAAGATATGTTGCTTCTCAGACTTTCACCGCATCTTTTGCCCCA
TTACAAGGTTTGGATAGATGGTTATCTTATTTAGTTTGGGTTACAGTTTTTGCTGCCAAGTATTCTGAATCGTACTTCTTCTTGATTTTGTCTCTAAGAGACCCTATCAGAATTTTA
TCAACTACTACCATGAGATGTACTGGTGAGTACTGGTGGGGTTCAAAGTTATGTAGACATCAATCGAAGATTGTTTTAGGTTTCATGATTGCTACAGATTTCATTCTGTTCTTCCT
TGATACTTATTTGTGGTACATTGTTGTCAACACTGTCTTCTCTGTTGGTAAATCCTTCTATTTAGGTATTTCCATCTTAACTCCTTGGAGAAATATCTTTACAAGATTACCAAAGAG
AATTTACTCAAAGATTTTGGCCACAACTGATATGGAGATTAAA

>57

GTGGCCACTGTTTTATTCTTCTCGATTATGCCATTAGGTGGTCTTTTCACCTCATATATGCAAAAATCAAGTAGAAGATATGTTGCTTCTCAGACTTTCACCGCATCTTTTGCCCCA
TTACAAGGTTTGGATAGATGGTTATCTTATTTAGTTTGGGTTACAGTTTTTGCTGCCAAGTATTCTGAATCGTACTTCTTCTTGATTTTGTCTCTAAGAGACCCTATCAGAATTTTA
TCAACTACTACCATGAGATGTACTGGTGAGTACTGGTGGGGTTCAAAGTTATGTAGACATCAATCGAAGATTGTTTTAGGTTTCATGATTGCTACAGATTTCATTCTGTTCTTCCT

TGATACTTATTTGTGGTACATTGTTGTCAACACTGTCTTCTCTGTTGGTAAATCCTTCTATTTAGGTATTTCCATCTTAACTCCTTGGAGAAATATCTTTACAAGATTACCAAAGAG
AATTTACTCAAAGATTTTGGCCACAACTGATATGGAGATTAAA

>58

GTGGCCACTGTTTTATTCTTCTCGATTATGCCATTAGGTGGTCTTTTCACCTCATATATGCAAAAATCAAGTAGAAGATATGTTGCTTCTCAGACTTTCACCGCATCTTTTGCCCCA
TTACAAGGTTTGGATAGATGGTTATCTTATTTAGTTTGGGTTACAGTTTTTGCTGCCAAGTATTCTGAATCGTACTTCTTCTTGATTTTGTCTCTAAGAGACCCTATCAGAATTTTA
TCAACTACTACCATGAGATGTACTGGTGAGTACTGGTGGGGTTCAAAGTTATGTAGACATCAATCGAAGATTGTTTTAGGTTTCATGATTGCTACAGATTTCATTCTGTTCTTCCT
TGATACTTATTTGTGGTACATTGTTGTCAACACTGTCTTCTCTGTTGGTAAATCCTTCTATTTAGGTATTTCCATCTTAACTCCTTGGAGAAATATCTTTACAAGATTACCAAAGAG
AATTTACTCAAAGATTTTGGCCACAACTGATATGGAGATTAAA

>71

GTGGCCACTGTTTTATTCTTCTCGATTATGCCATTAGGTGGTCTTTTCACCTCATATATGCAAAAATCAAGTAGAAGATATGTTGCTTCTCAGACTTTCACCGCATCTTTTGCCCCA
TTACAAGGTTTGGATAGATGGTTATCTTATTTAGTTTGGGTTACAGTTTTTGCTGCCAAGTACTCTGAATCGTACTTCTTCTTGATTTTGTCTCTAAGAGACCCTATCAGAATTTTA
TCAACTACTACCATGAGATGTACTGGTGAGTACTGGTGGGGTTCAAAGTTATGTAGACATCAATCGAAGATTGTTTTAGGTTTCATGATTGCTACAGATTTCATTTTGTTCTTCCT
TGATACTTATTTGTGGTACATTGTTGTCAACACTGTCTTCTCTGTTGGTAAATCCTTCTATCTAGGTATTTCCATCTTAACTCCTTGGAGAAATATCTTTACAAGATTACCAAAGAG
AATTTACTCAAAGATTTTGGCCACAACTGATATGGAGATTAAA

>72

GTGGCCACTGTTTTATTCTTCTCGATTATGCCATTAGGTGGTCTTTTCACCTCATATATGCAAAAATCAAGTAGAAGATATGTTGCTTCTCAGACTTTCACCGCATCTTTTGCCCCA
TTACAAGGTTTGGATAGATGGTTATCTTATTTAGTTTGGGTTACTGTTTTTGCTGCCAAGTACTCTGAATCGTACTTCTTCTTGATTTTGTCTCTAAGAGACCCTATCAGAATTTTA
TCAACTACTACCATGAGATGTACTGGTGAGTATTGGTGGGGTTCAAAGTTATGTAGACATCAATCGAAGATTGTTTTAGGTTTCATGATTGCTACAGATTTCATTCTGTTCTTCCT
TGATACTTATTTGTGGTACATTGTTGTCAACACTGTCTTCTCTGTTGGTAAATCCTTCTATTTAGGTATTTCCATCTTAACTCCTTGGAGAAATATCTTTACAAGATTACCAAAGAG
AATTTACTCAAAGATTTTGGCCACAACTGATATGGAGATTAAA

>73

GTGGCCACTGTTTTATTCTTCTCGATTATGCCATTAGGTGGTCTTTTCACCTCATATATGCAAAAATCAAGTAGAAGATATGTTGCTTCTCAGACTTTCACCGCATCTTTTGCCCCA
TTACAAGGTTTGGATAGATGGCTATCTTATTTAGTTTGGGTTACAGTTTTTGCTGCCAAATACTCTGAATCGTACTTCTTCTTGATTTTGTCTCTAAGAGACCCTATCAGAATTTTA
TCAACTACTACCATGAGATGTACTGGTGAGTATTGGTGGGGTTCAAAGTTATGTAGACATCAATCGAAGATTGTTTTAGGTTTCATGATTGCTACAGATTTCATTTTGTTCTTCCT
TGATACTTATTTGTGGTACATTGTTGTCAACACTGTCTTCTCTGTTGGTAAATCCTTCTATCTAGGTATTTCCATCTTAACTCCTTGGAGAAATATCTTTACAAGATTACCAAAGAG
AATTTACTCAAAGATTTTGGCCACAACTGATATGGAGATTAAA

>74

GTGGCCACTGTTTTATTCTTCTCGATTATGCCATTAGGTGGTCTTTTCACCTCATATATGCAAAAATCAAGTAGAAGATATGTTGCTTCTCAGACTTTCACCGCATCTTTTGCCCCA
TTACAAGGTTTGGATAGATGGTTATCTTATTTAGTTTGGGTTACTGTTTTTGCTGCCAAGTACTCTGAATCGTACTTCTTCTTGATTTTGTCTCTAAGAGACCCTATCAGAATTTTA
TCAACTACTACCATGAGATGTACTGGTGAGTATTGGTGGGGTTCAAAGTTATGTAGACATCAATCGAAGATTGTTTTAGGTTTCATGATTGCTACAGATTTCATTCTGTTCTTCCT
TGATACTTATTTGTGGTACATTGTTGTCAACACTGTCTTCTCTGTTGGTAAATCCTTCTATTTAGGTATTTCCATCTTAACTCCTTGGAGAAATATCTTTACAAGATTACCAAAGAG
AATTTACTCAAAGATTTTGGCCACAACTGATATGGAGATTAAA

>75

GTGGCCACTGTTTTATTCTTCTCGATTATGCCATTAGGTGGTCTTTTCACCTCATATATGCAAAAATCAAGTAGAAGATATGTTGCTTCTCAGACTTTCACCGCATCTTTTGCCCCA
TTACAAGGTTTGGATAGATGGCTATCTTATTTAGTTTGGGTTACAGTTTTTGCTGCCAAATACTCTGAATCGTACTTCTTCTTGATTTTGTCTCTAAGAGACCCTATCAGAATTTTA
TCAACTACTACCATGAGATGTACTGGTGAGTATTGGTGGGGTTCAAAGTTATGTAGACATCAATCGAAGATTGTTTTAGGTTTCATGATTGCTACAGATTTCATTTTGTTCTTCCT
TGATACTTATTTGTGGTACATTGTTGTCAACACTGTCTTCTCTGTTGGTAAATCCTTCTATCTAGGTATTTCCATCTTAACTCCTTGGAGAAATATCTTTACAAGATTACCAAAGAG
AATTTACTCAAAGATTTTGGCCACAACTGATATGGAGATTAAA

>76

GTGGCCACTGTTTTATTCTTCTCGATTATGCCATTAGGTGGTCTTTTCACCTCATATATGCAAAAATCAAGTAGAAGATATGTTGCTTCTCAGACTTTCACCGCATCTTTTGCCCCA
TTACAAGGTTTGGATAGATGGTTATCTTATTTAGTTTGGGTTACAGTTTTTGCTGCCAAGTACTCTGAATCGTACTTCTTCTTGATTTTGTCTCTAAGAGACCCTATCAGAATATTA
TCAACTACTACTATGAGATGTACTGGTGAGTATTGGTGGGGTTCAAAGTTATGTAGACATCAATCGAAGATTGTTTTAGGTTTCATGATTGCTACAGATTTCATTCTGTTCTTCCT
TGATACTTATTTGTGGTACATTGTTGTCAACACTGTCTTCTCTGTTGGTAAATCCTTCTATTTAGGTATTTCCATCTTAACTCCTTGGAGAAATATCTTTACAAGATTACCAAAGAG
AATTTACTCAAAGATTTTGGCCACAACTGATATGGAGATTAAA

>77

GTGGCCACTGTTTTATTCTTCTCGATTATGCCATTAGGTGGTCTTTTCACCTCATATATGCAAAAATCAAGTAGAAGATATGTTGCTTCTCAGACTTTCACCGCATCTTTTGCCCCA
TTACAAGGTTTGGATAGATGGCTATCTTATTTAGTTTGGGTTACAGTTTTTGCTGCCAAATACTCTGAATCGTACTTCTTCTTGATTTTGTCTCTAAGAGACCCTATCAGAATTTTA
TCAACTACTACCATGAGATGTACTGGTGAGTATTGGTGGGGTTCAAAGTTATGTAGACATCAATCGAAGATTGTTTTAGGTTTCATGATTGCTACAGATTTCATTTTGTTCTTCCT
TGATACTTATTTGTGGTACATTGTTGTCAACACTGTCTTCTCTGTTGGTAAATCCTTCTATCTAGGTATTTCCATCTTAACTCCTTGGAGAAATATCTTTACAAGATTACCAAAGAG
AATTTACTCAAAGATTTTGGCCACAACTGATATGGAGATTAAA

>78

GTGGCCACTGTTTTATTCTTCTCGATTATGCCATTAGGTGGTCTTTTCACCTCATATATGCAAAAATCAAGTAGAAGATATGTTGCTTCTCAGACTTTCACCGCATCTTTTGCCCCA
TTACAAGGTTTGGATAGATGGTTATCTTATTTAGTTTGGGTTACAGTTTTTGCTGCCAAGTACTCTGAATCGTACTTCTTCTTGATTTTGTCTCTAAGAGACCCTATCAGAATATTA
TCAACTACTACTATGAGATGTACTGGTGAGTATTGGTGGGGTTCAAAGTTATGTAGACATCAATCGAAGATTGTTTTAGGTTTCATGATTGCTACAGATTTCATTCTGTTCTTCCT
TGATACTTATTTGTGGTACATTGTTGTCAACACTGTCTTCTCTGTTGGTAAATCCTTCTATTTAGGTATTTCCATCTTAACTCCTTGGAGAAATATCTTTACAAGATTACCAAAGAG
AATTTACTCAAAGATTTTGGCCACAACTGATATGGAGATTAAA

>79

GTGGCCACTGTTTTATTCTTCTCGATTATGCCATTAGGTGGTCTTTTCACCTCATATATGCAAAAATCAAGTAGAAGATATGTTGCTTCTCAGACTTTCACCGCATCTTTTGCCCCA
TTACAAGGTTTGGATAGATGGTTATCTTATTTAGTTTGGGTTACAGTTTTTGCTGCCAAGTACTCTGAATCGTACTTCTTCTTGATTTTGTCTCTAAGAGACCCTATCAGAATATTA
TCAACTACTACTATGAGATGTACTGGTGAGTATTGGTGGGGTTCAAAGTTATGTAGACATCAATCGAAGATTGTTTTAGGTTTCATGATTGCTACAGATTTCATTCTGTTCTTCCT
TGATACTTATTTGTGGTACATTGTTGTCAACACTGTCTTCTCTGTTGGTAAATCCTTCTATTTAGGTATTTCCATCTTAACTCCTTGGAGAAATATCTTTACAAGATTACCAAAGAG
AATTTACTCAAAGATTTTGGCCACAACTGATATGGAGATTAAA

>80

GTGGCCACTGTTTTATTCTTCTCGATTATGCCATTAGGTGGTCTTTTCACCTCATATATGCAAAAATCAAGTAGAAGATATGTTGCTTCTCAGACTTTCACCGCATCTTTTGCCCCA
TTACAAGGTTTGGATAGATGGTTATCTTATTTAGTTTGGGTTACAGTTTTTGCTGCCAAGTACTCTGAATCGTACTTCTTCTTGATTTTGTCTCTAAGAGACCCTATCAGAATTTTA
TCAACTACTACCATGAGATGTACTGGTGAGTACTGGTGGGGTTCAAAGTTATGTAGACATCAATCGAAGATTGTTTTAGGTTTCATGATTGCTACAGATTTCATTTTGTTCTTCCT
TGATACTTATTTGTGGTACATTGTTGTCAACACTGTCTTCTCTGTTGGTAAATCCTTCTATCTAGGTATTTCCATCTTAACTCCTTGGAGAAATATCTTTACAAGATTACCAAAGAG
AATTTACTCAAAGATTTTGGCCACAACTGATATGGAGATTAAA

>81

GTGGCCACTGTTTTATTCTTCTCGATTATGCCATTAGGTGGTCTTTTCACCTCATATATGCAAAAATCAAGTAGAAGATATGTTGCTTCTCAGACTTTCACCGCATCTTTTGCCCCA
TTACAAGGTTTGGATAGATGGTTATCTTATTTAGTTTGGGTTACTGTTTTTGCTGCCAAGTACTCTGAATCGTACTTCTTCTTGATTTTGTCTCTAAGAGACCCTATCAGAATTTTA
TCAACTACTACCATGAGATGTACTGGTGAGTATTGGTGGGGTTCAAAGTTATGTAGACATCAATCGAAGATTGTTTTAGGTTTCATGATTGCTACAGATTTCATTCTGTTCTTCCT
TGATACTTATTTGTGGTACATTGTTGTCAACACTGTCTTCTCTGTTGGTAAATCCTTCTATTTAGGTATTTCCATCTTAACTCCTTGGAGAAATATCTTTACAAGATTACCAAAGAG
AATTTACTCAAAGATTTTGGCCACAACTGATATGGAGATTAAA

>82

GTGGCCACTGTTTTATTCTTCTCGATTATGCCATTAGGTGGTCTTTTCACCTCATATATGCAAAAATCAAGTAGAAGATATGTTGCTTCTCAGACTTTCACCGCATCTTTTGCCCCA
TTACAAGGTTTGGATAGATGGTTATCTTATTTAGTTTGGGTTACAGTTTTTGCTGCCAAGTATTCTGAATCGTACTTCTTCTTGATTTTGTCTCTAAGAGACCCTATCAGAATTTTA

TCAACTACTACCATGAGATGTACTGGTGAGTACTGGTGGGGTTCAAAGTTATGTAGACATCAATCGAAGATTGTTTTAGGTTTCATGATTGCTACAGATTTCATTCTGTTCTTCCT
TGATACTTATTTGTGGTACATTGTTGTCAACACTGTCTTCTCTGTTGGTAAATCCTTCTATTTAGGTATTTCCATCTTAACTCCTTGGAGAAATATCTTTACAAGATTACCAAAGAG
AATTTACTCAAAGATTTTGGCCACAACTGATATGGAGATTAAA

>83

GTGGCCACTGTTTTATTCTTCTCGATTATGCCATTAGGTGGTCTTTTCACCTCATATATGCAAAAATCAAGTAGAAGATATGTTGCTTCTCAGACTTTCACCGCATCTTTTGCCCCA
TTACAAGGGTTGGATGGATGGTTATCTTATTTAGTTTGGGTTACTGTTTTTGCTGCCAAGTACTCTGAATCGTACTTCTTCTTGATTTTGTCTCTTAGAGACCCTATCAGAATTTTA
TCAACTACTACCATGAGATGTACTGGTGAGTATTGGTGGGGTTCAAAGTTATGTAGACATCAATCGAAGATTGTTTTAGGTTTCATGATTGCTACAGATTTCATTCTGTTCTTCCT
TGATACTTATTTGTGGTACATTGTTGTCAACACTGTCTTCTCTGTTGGTAAATCCTTCTATTTAGGTATTTCCATCTTAACTCCCTGGAGAAATATCTTTACAAGATTACCAAAGAG
AATTTACTCAAAGATTTTGGCCACAACTGATATGGAGATTAAA
